# Identification of candidate chemosensory genes in the antennal transcriptome of *Monolepta signata*

**DOI:** 10.1371/journal.pone.0301177

**Published:** 2024-06-07

**Authors:** Wanjie He, Hanying Meng, Yu Zhang, Ge Zhang, Mengting Zhi, Guangwei Li, Jing Chen

**Affiliations:** 1 College of Agriculture / Key Laboratory of Oasis Agricultural Pest Management and Plant Protection Resources Utilization, Xinjiang Uygur Autonomous Region, Shihezi University, Shihezi, Xinjiang, China; 2 Yuli Industry Development Service Center of *Apocynum venetum*, Xinjiang, China; 3 Plant Protection Station of Xinjiang Uygur Autonomous Region, Urumqi, Xinjiang, China; 4 Xinjiang Uygur Autonomous Region Science and Technology Development Strategy Research Institute, Urumqi, Xinjiang, China; 5 Shaanxi Province Key Laboratory of Jujube, College of Life Science, Yan’an University, Yan’an, Shaanxi, China; Monell Chemical Senses Center, UNITED STATES

## Abstract

In the polyphagous insect *Monolepta signata* (*M*. *signata*) (Coleoptera: Chrysomelidae), antennae are important for olfactory reception used during feeding, mating, and finding a suitable oviposition site. Based on NextSeq 6000 Illumina sequencing, we assembled the antennal transcriptome of mated *M*. *signata* and described the first chemosensory gene repertoire expressed in this species. The relative expression levels of some significant chemosensory genes were conducted by quantitative real-time PCR. We identified 114 olfactory-related genes based on the antennal transcriptome database of *M*. *signata*, including 21 odorant binding proteins (OBPs), six chemosensory proteins (CSPs), 46 odorant receptors (ORs), 15 ionotropic receptors (IRs), 23 gustatory receptors (GRs) and three sensory neuron membrane proteins (SNMPs). Blastp best hit and phylogenetic analyses showed that most of the chemosensory genes had a close relationship with orthologs from other Coleoptera species. Overall, this study provides a foundation for elucidating the molecular mechanism of olfactory recognition in *M*. *signata* as well as a reference for the study of chemosensory genes in other species of Coleoptera.

## 1. Introduction

*Monolepta signata* (*M*. *signata*) belongs to the subfamily Galerucinae of the family Chrysomelidae of Coleoptera and is mainly distributed in East Asia and Southeast Asia [1]. The species is distributed in most areas of China, and it occurs at high densities in northern China [2]. *M*. *signata* is an herbivorous pest, with adults feeding on the leaves and flowers of host plants and causing damage to a variety of cash crops, such as corn, cotton, sunflowers, soybeans, rice, grains, sorghum, peanuts, potatoes, and cruciferous vegetables [3]. The beetle is characterized by an extensive damage period, high population density, and rapid spread within crop areas [4]. Similar to most insects of Chrysomelidae, *M*. *signata* identifies and locates host plants based on the volatile substances released by the plants, but research on this beetle has primarily focused on the biological characteristics and control effects [5–7]. The molecular mechanisms of interactions with host plants and their targets have not been reported. Identifying the olfactory-related genes of *M*. *signata* is a prerequisite for studying the olfactory recognition process of this beetle at the molecular level.

The co-evolution of herbivorous insects and plants has defined the range of host plants for herbivorous insects and the defenses of plants, and the insects can distinguish between host and non-host plants. This ensures the growth and reproduction of insects, and it prevents their poisoning and malnutrition from feeding on non-host plants [8]. The chemoreception process involves the reception, binding, transport, and inactivation of odorants, ultimately activating receptor neurons and converting chemical signals into electrical signals to the insect brain [9]. Interactions between different types of proteins determine the high sensitivity and specificity of the insect olfactory system [10]. Odorant binding proteins (OBPs) and chemosensory proteins (CSPs) are involved with binding, and transport of odorant substances. Odorant receptors (ORs), gustatory receptors (GRs), and ionotropic receptors (IRs) can recognize specific odor molecules in complex environments, then covert the recognized chemical signal into electrical signals through receptor-mediated signal transduction. Peripheral neuron depolarization is then transmitted through axonal processes to the central nervous system of the brain, and sensory neuronal membrane proteins (SNMPs) identify and transport lipophilic odor molecules [11]. With the application of high-throughput sequencing technology in insect functional gene mining, a variety of insect chemosensory genes has been identified. In total 26 OBP genes, 15 CSP genes, 37 OR genes, 10 IRs, and three SNMP genes were identified in the antennal transcriptome of *Leptinotarsa decemlineata* [12]. Twenty-six OBPs, 12 CSPs, 4 SNMPs, 43 ORs, 9 IRs, and 10 GRs were identified in the antennal transcriptome of *Colaphellus bowringi* [13]. Transcriptome sequencing and olfactory-related gene mining of the antenna and leg of *Ambrostoma quadriimpressum* Motschulsky yielded 16 OBPs, 10 CSPs, 34 ORs, 20 IRs, and two SNMPs [14].

Insect recognition of volatile substances emitted by plants is an important factor affecting insect feeding [15]. By measuring the electrophysiological responses of the antennae, it was found that certain concentrations of geraniol, 3-carene, β-pinene, α-erythro-myrcene, and leaf alcohol could produce strong stimulating effects in *M*. *signata* [16, 17]. Behavioral bioassays showed that 10 μg/mL β-pinacolone had a significant attraction effect on the females and a significant avoidance effect on the males, while γ-terpinene and D-limonene had significant attraction effects on the males and a significant avoidance effect on the females [18, 19]. Additionally, various types of antennal chemoreceptor sensilla (notably sensilla trichodea, sensilla basiconica, sensilla coeloclnica, and sensilla campaniformia) have been recently characterized in *M*. *signata* [20]. Thus, understanding the molecular basis of *M*. *signata* chemoreception, in particular the types and number of olfactory-related genes, is likely to provide new information that can be used to increase the biological control efficacy for this pest and further clarify the relationship between the pest and the host plant.

In this study, using the antenna transcriptome data of *M*. *signata* adults assembled in our laboratory (BioProject Accession Number: PRJNA960895), the chemosensory genes of *M*. *signata* were identified and examined through bioinformatic analyses. Moreover, the relative expression levels of some chemosensorygenes were analyzed by quantitative real-time PCR (RT-qPCR). The results provide theoretical support for future studies on the mechanism of chemoreception of *M*. *signata* and ultimately allow us to identify potential targets for disrupting odorant perception in *M*. *signata* that could lead to new pest management techniques.

## 2. Materials and methods

### 2.1. Insect rearing and antenna collection

Adults of *M*. *signata* were collected from a corn field in Shawan city, Xinjiang province, China (85°48′E, 44°03′N), before being cultured at the laboratory and fed with fresh cotton leaves every day. For transcriptome sequencing, three male and three female biological replicates were collected, each consisting of 100 pairs of antennae. For the antennal qRT-PCR study, three male and three female biological replicates were collected, each consisting of 100 pairs of antennae. These samples were immediately frozen in liquid nitrogen and stored at −80°C until they were used.

### 2.2. RNA extraction and cDNA library construction

Total RNA was extracted using Trizol reagent (Invitrogen, Carlsbad, CA, USA), the concentration of RNA was determined with a ND-2000 Spectrophotometer (Nanodrop Technologies, Wilmington, DE, USA), and UV absorption values were recorded at 260 / 280 nm to test the purity of RNA products. RNA integrity was monitored on 1% agarose gel electrophoresis. The Illumina sequencing of the samples was performed by Berry Genomics (Beijing, China). The cDNA library was synthetized with NEBNext® Ultra mRNA Library Prep Kit for Illumina (NEB, Ipswich, MA, USA) following manufacturer’s instructions. The mRNAs were enriched from total RNA using Oligo(dT)-attached magnetic beads and mRNAs were fragmented into short sequences within an RNA fragmentation buffer. Next, the first-strand cDNA was generated using random hexamer-primed reverse transcription, followed by synthesis of the second-strand cDNA using the buffer, dNTPs, RNaseH and DNA polymerase I. Then, end repair was performed on these double strands of cDNA with a dA-tail was added. After the end repair and ligation of adaptors, the PCR was performed to enrich the cDNA. Finally, cDNA library construction and Illumina sequencing of the samples were performed at Novogene Bioinformatics Technology Co., Ltd (Beijing, China).

### 2.3. De novo assembly and gene annotation

After sequencing, clean reads were obtained after the quality control of raw data and then spliced using Trinity software (version 2.6.6) to generate a set of transcripts. BUSCO was used to verify the completeness of the assemblage [21]. These transcripts were annotated according to the following databases: NCBI nonredundant protein sequences (NCBI-nr), NCBI nucleotide sequences (NCBI-nt), Gene Ontology (GO), Kyoto Encyclopedia of Genes and Genomes (KEGG-Ontology), Protein family database (Pfam), EuKaryotic Ortholog Groups / Clusters of Orthologous Groups (KOG / COG) and Swiss-Prot databases by Blast alignment with a cut-off *E*-value of 10^−5^.

### 2.4. Identification of chemosensory genes and bioinformatic analysis

Based on functional annotation information of the antennae transcriptome sequencing data of *M*. *signata*, preliminary candidate chemosensory gene nucleic acid sequences were obtained. The candidate chemosensory protein gene sequences were further compared and verified by Blastp at NCBI (www.ncbi.nlm.nih.gov), and the expected e-value was < 10^−5^. Open reading frames (ORFs) of candidate olfactory-related protein genes were predicted and verified by ORF finder in NCBI (https://www.ncbi.nlm.nih.gov/orffifinder). The default parameters of SignalP 5.0 Server online software (https://services.healthtech.dtu.dk) were utilized to predict signal peptides for candidate chemosensory protein genes. Transmembrane domains of both MsigORs, MsigIRs and MsigGRs were predicted with the TMHMM Server Version 2.0 (https://services.healthtech.dtu.dk/services/TMHMM-2.0/). The physical and chemical properties of proteins were predicted by Expasy online software (https://web.expasy.org/protparam). DNAMAN software (version 6.03) was used to analyze the protein sequence characteristics and sequence consistency. The nucleotide sequences of chemosensory gene transcripts in *M*. *signata* are listed in S1 File. The amino acid sequences of *M*. *signata* are listed in S2 File.

### 2.5. Phylogenetic analysis of olfactory-related genes from *M*. *signata* and other insects

The amino acid sequence alignment of the candidate OBPs, CSPs, ORs, GRs, and SNMPs of *M*. *signata* and other Coleopteran insects and *Drosophila melanogaster* was performed using the ClustalW [22] program implemented in the Mega V7.0 software package [23]. The phylogenetic tree was constructed using the neighbor-joining (NJ) method [24] with P-distance modeling [25] and pairwise deletion of gaps being performed, and a bootstrap procedure of 1000 replicates was used to evaluate the node support. Phylogenetic trees were visualized using Evolview (www.evolgenius.info/evolview-v2). The data sets of olfactory-related genes chosen from other species of Coleoptera are listed in S3 File.

### 2.6. RT-qPCR validation of MsigOBPs, MsigCSPs, and MsigORs

Based on FPKM values (S1 Table) and sequence analysis, expression profiles of nine OBPs, three ORs, and three CSPs were identified using RT-qPCR. The total RNA of each tissue sample of *M*. *signata* was extracted following the manufacturer’s protocol by using a Trizol reagent kit (Servicebio). First-stand cDNA was synthesized from 2 μg of total RNA using the Servicebio® RT First Strand cDNA Synthesis Kit (Servicebio). RT-qPCR was performed using 2 × SYBR Green qPCR Master Mix (Servicebio). RT-qPCR reactions were conducted in 15 μL reaction volumes containing 7.5 μl of 2× qPCR Mix, 1.5 μl of forward and reverse primers (2.5 μM), 2 μL of cDNA, and 4 μL Water Nuclease-Free. Gene-specific primers (S2 Table) were designed using the Primer designing tool (https://www. ncbi. nlm. nih. gov/ tools/ primer-blast/ index. cgi) and sequenced after PCR. RT-qPCR conditions were as follows: one cycle of 95°C for 30 s followed by 40 cycles of 95°C for 15 s and 60°C for 30 s. The RT-qPCR was performed on a CFX Connect Real-time PCR Detection System (Bio-Rad, Hercules, CA, USA). The glyceraldehyde-3-phosphate dehydrogenase (*GAPDH*) gene was selected as a reference gene to normalize the relative expression levels of OBP, CSP, and OR genes. The Ct values of divergent antennae from RT-qPCR were normalized to that of the endogenous control in the same tissue and presented as fold change relative to the expression level in the female antennae. Three biological replicates of each sample were analyzed, and relative expression levels of selected genes across the sample were measured using the 2^−ΔΔCT^ method [26]. The difference between antennae of males and females was established by independent sample *t*-tests using SPSS (SPSS Institute 20.0, SPSS Inc, Chicago, IL, USA).

## 3. Results

### 3.1. Transcriptome overview

We carried out next-generation sequencing on a cDNA library constructed from the adult antennae of *M*. *signata* using the Illumina NovaSeq 6000 platform. In total, each cDNA library was deep sequenced to yield 6.0 Gb of clean data. After clustering and redundancy filtering, we identified 73, 050 unigenes with the average length of 1140 bp. The length of N50 and N90 were 1896 bp and 440 bp, respectively. The Q20 and Q30 accounted for more than 97.75% and 93.23%, respectively. Through annotation by Blast using the NR, NT, GO, KEGG, Pfam, KOG/COG and Swiss-Prot databases, 34, 233 unigenes were matched to known proteins (S4 File). The raw data were deposited in the NCBI Short Read Archive (SRA) database with BioProject accession number: PRJNA960895.

### 3.2. Identification of candidate odorant binding proteins

**Fig 1:**
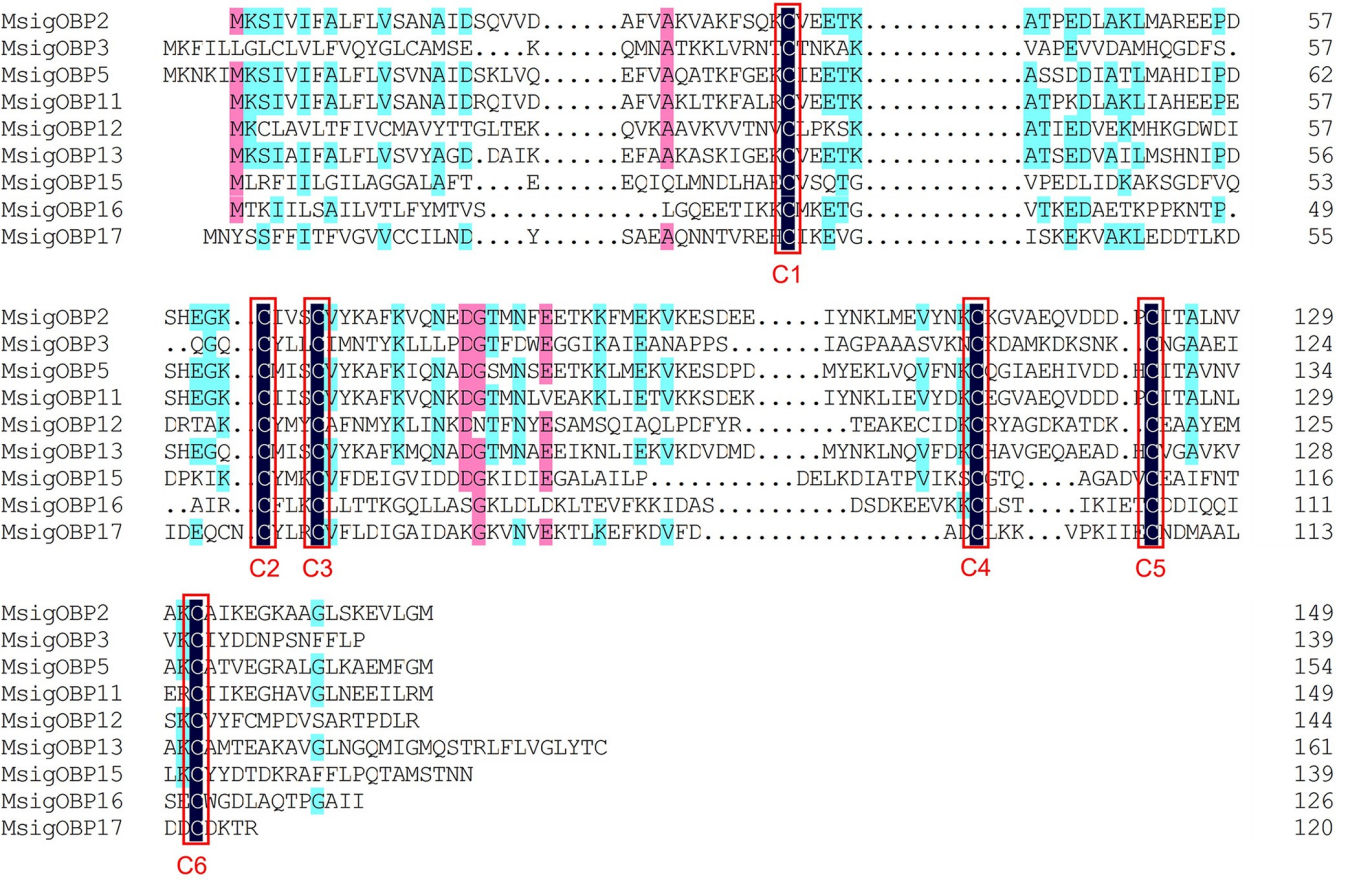
Multiple amino acid sequence alignment of Classic OBPs in *M*. *signata*. The six conserved cysteine residues are highlighted in black ground and red border. Amino acids that are more than 50% identical in all sequences are marked with cyan color highlights, and more than 75% identical are marked with pink highlights.

**Fig 2:**
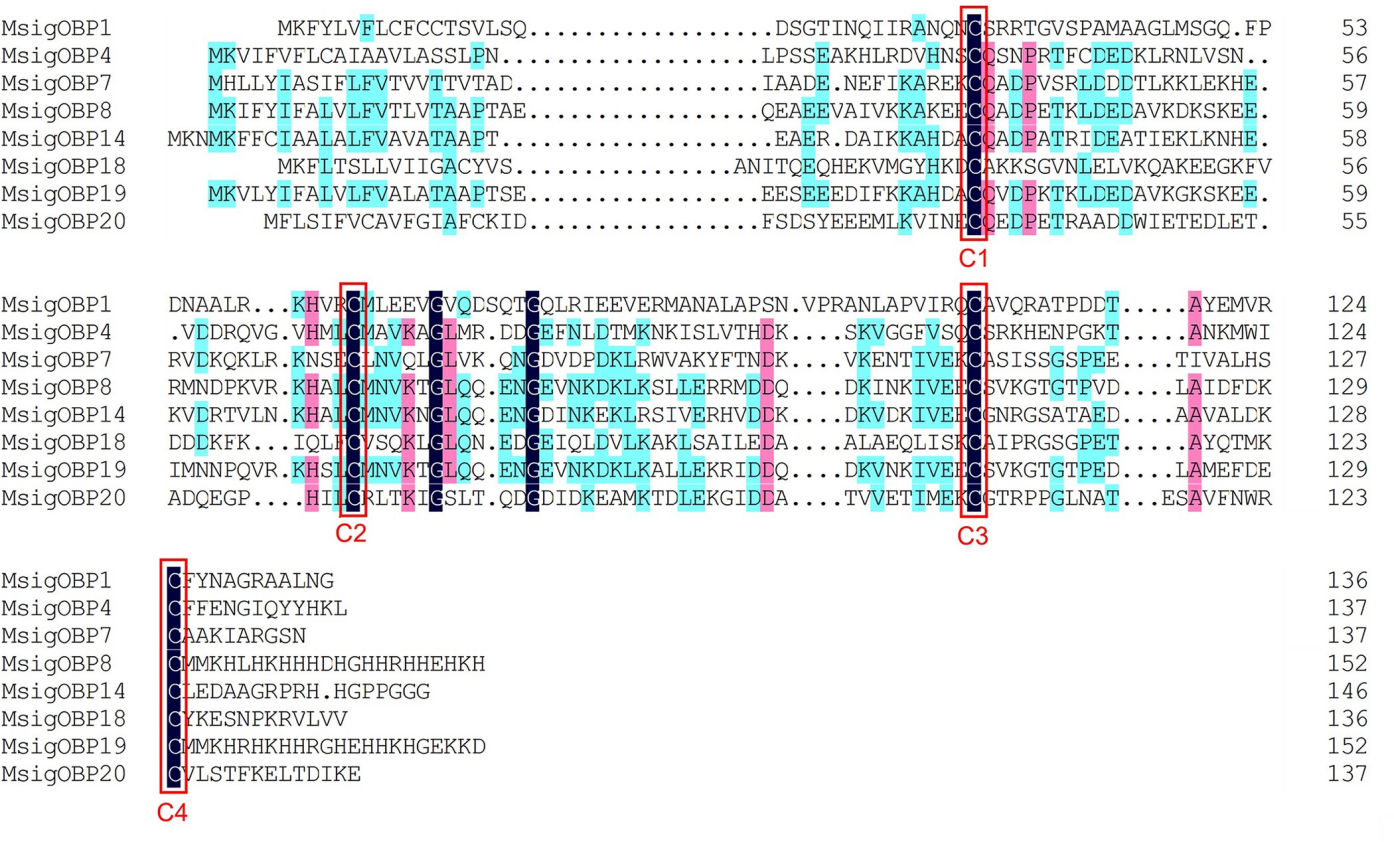
Multiple amino acid sequence alignment of Minus-C OBPs in *M*. *signata*. The four conserved cysteine residues are highlighted in black ground and red border. Amino acids that are more than 50% identical in all sequences are marked with cyan color highlights, and more than 75% identical are marked with pink highlights.

**Fig 3:**
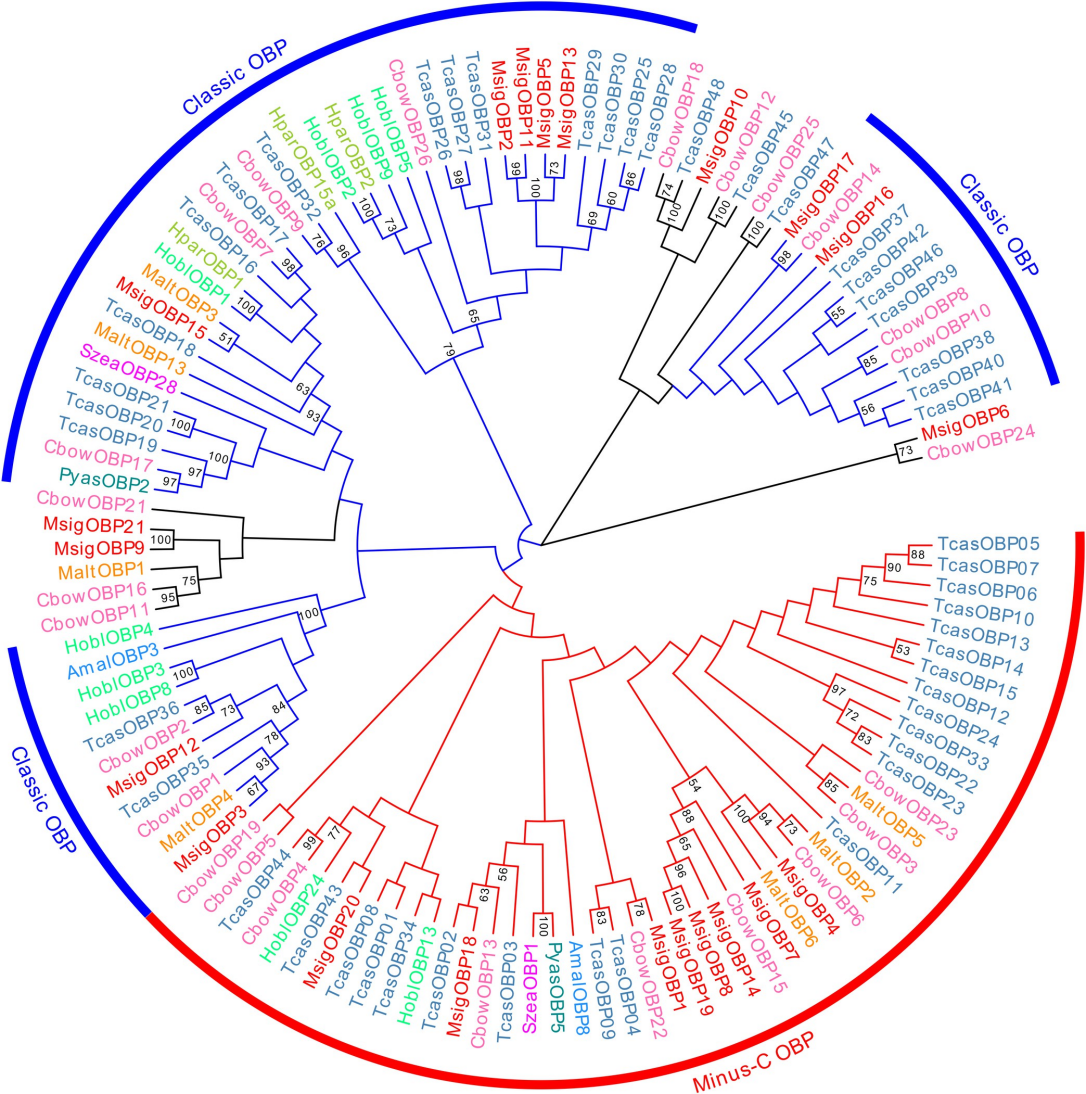
Phylogenetic tree of candidate MsigOBPs with known Coleopteran OBP sequences. Amal, *Agrilus mali* (N = 2); Cbow, Colaphellus bowringi (N = 25); Hobl, *Holotrichia oblita* (N = 9); Hpar, *Holotrichia parallela* (N = 3); Malt, *Monochamus alternatus* (N = 7); Pyas, *Pachyrhinus yasumatsui* (N = 2); Szea, *Sitophilus zeamais* (N = 2); Tcas, *Tribolium castaneum* (N = 48). Bootstrap values >50 are shown.

We identified 21 OBP candidate genes based on the antennal transcriptome data for *M*. *signata*; these were named *MsigOBP1*-*MsigOBP21*. Among the candidate genes, 20 unigenes were likely to represent full-length genes as they contained a complete ORF, and all of the unigenes had predicted signal peptide sequences. All of the candidate OBP sequence Blastp best hits were similar to known Coleoptera OBPs. The length of all of the putative full-length MsigOBPs ranged from 120 to 180 amino acids (S3 Table). Nine MsigOBPs (*MsigOBP2*, 3, 5, 11, 12, 13, 15, 16, and 17) contained six conserved cysteine residues, and the sequence features were C_1_-X_24-29_-C_2_-X_3_-C_3_-X_37-42_-C_4_-X_8-10_-X_8_-C_6_ (X represents any amino acid except cysteine), a pattern that belonged to the “Classic OBP” subfamily (Fig 1). Eight MsigOBPs (*MsigOBP1*, 4, 7, 8, 14, 18, 19, and 20) belonged to the “Minus-C OBP” subfamily (Fig 2). Compared with ORs, insect OBPs were highly conserved. *MsigOBP15* shared 86% identity with the OBP of *Diabrotica virgifera virgifera* (NCBI ID: XP_028129817.1), and *MsigOBP10* shared 73% identity with *PmacOBP31* in *Pyrrhalta maculicollis*. A phylogenetic tree was generated to infer the relationships between 21 OBPs of *M*. *signata* and 98 OBPs with known functions from seven Coleopteran insects. The results showed that *MsigOBP1* and *CbowOBP22*, *MsigOBP3* and *MaltOBP4*, *MsigOBP6* and *CbowOB*, and *MsigOBP17* and *CbowOBP14* were clustered together with a high degree of homology (Fig 3).

### 3.3. Identification of candidate chemosensory proteins

**Fig 4:**
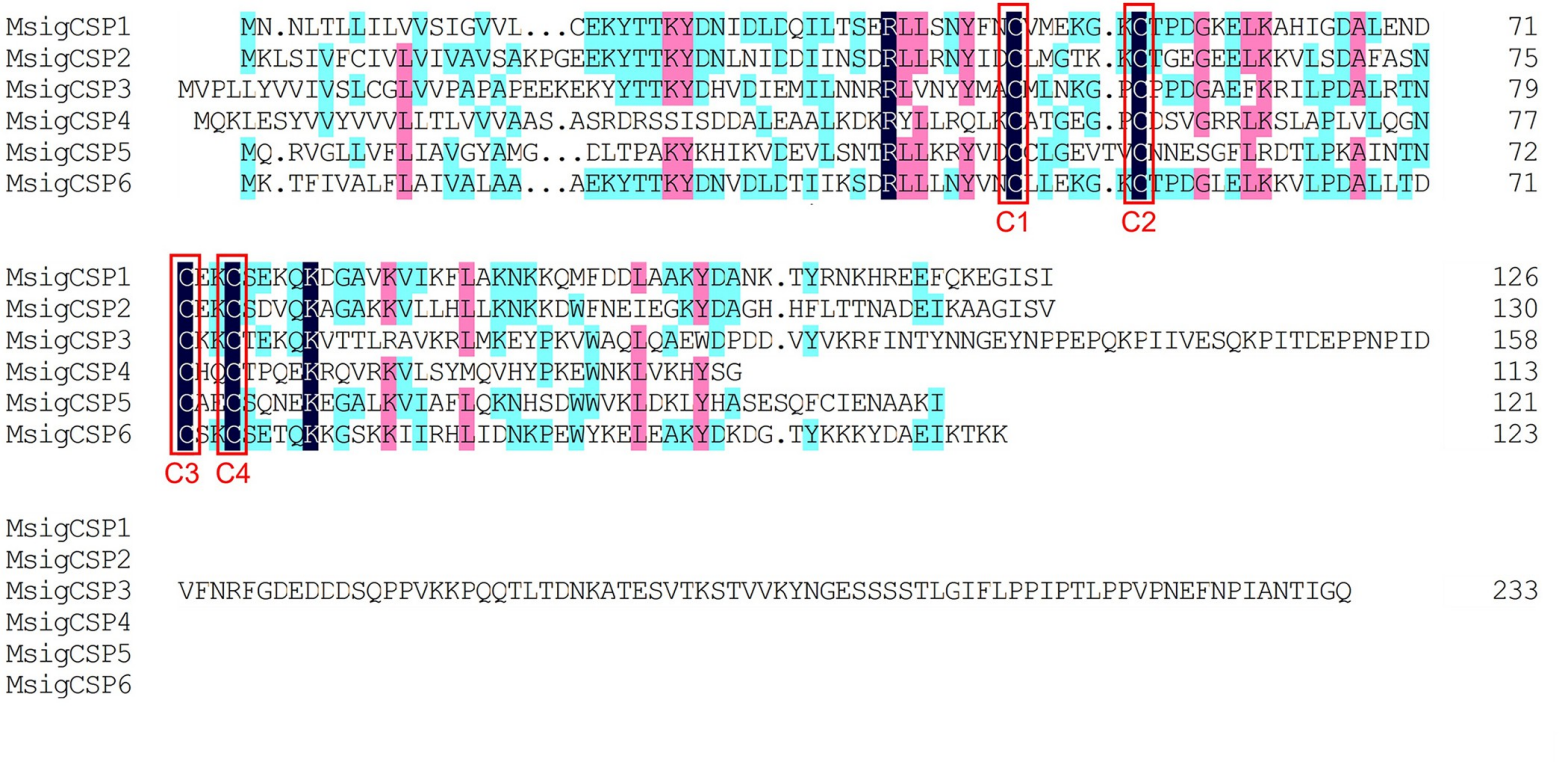
Multiple amino acid sequence alignment of CSPs in *M*. *signata*. The four conserved cysteine residues are highlighted in black ground and red border. Amino acids that are more than 50% identical in all sequences are marked with cyan color highlights, and more than 75% identical are marked with pink highlights.

**Fig 5:**
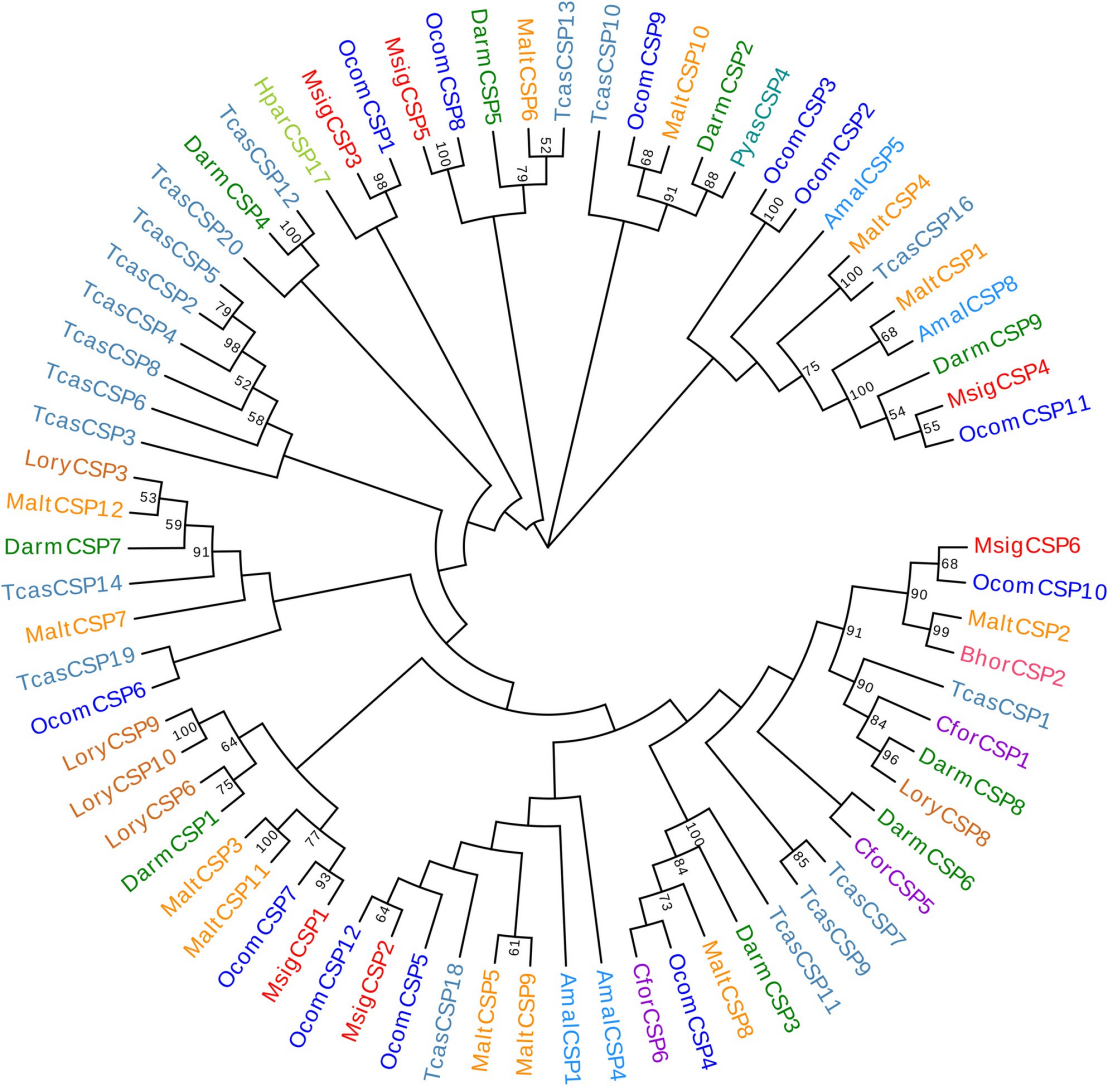
Phylogenetic tree of candidate MsigCSPs with known Coleopteran CSP sequences. Amal, *Agrilus mali* (N = 4); Bhor, *Batocera horsfieldi* (N = 1); Cfor, *Cylas formicarius* (N = 3); Darm, *Dendroctonus armandi* (N = 9); Hpar, *Holotrichia parallela* (N = 1); Lory, *Lissorhoptrus oryzophilus* (N = 5); Malt, *Monochamus alternatus* (N = 12); Ocom, *Ophraella communa* (N = 12); Pyas, *Pachyrhinus yasumatsui* (N = 1); Tcas, *Tribolium castaneum* (N = 18). Bootstrap values >50 are shown.

Bioinformatic analysis led to the identification of six different sequences of candidate CSPs in the *M*. *signata* antennal transcriptome; these were named *MsigCSP1*-*MsigCSP6*. Four sequences were predicted with a putative full-length ORF, and all of the unigenes had predicted signal peptide sequences. The lengths of all of the putative full-length MsigCSPs ranged from 113 to 130 amino acids (S3 Table). In addition, all of the MsigCSPs followed the highly conserved pattern with four cysteine residues arranged with an exact spacing of C_1_-X_6_-C_2_-X_18_-C_3_-X_2_-C_4_ (X represents any amino acid except cysteine) (Fig 4). Insect CSPs are more conserved than ORs or OBPs, and all of the MsigCSPs amino acid sequences have more than 48% identity with CSPs of *P*. *maculicollis*, *C*. *bowringi*, *Galeruca daurica*, and *Ophraella communa*. Notably, the identity between *MsigCSP6* and *PmacCSP6* was as high as 94%. A phylogenetic tree was generated to infer the relationships between six CSPs of *M*. *signata* and 66 CSPs from nine species of Coleoptera. Homology analysis showed that the MsigCSPs were dispersed in different branches of the phylogenetic tree. All of the MsigOBPs were orthologs of known OcomCSPs (*OcomCSP1*, 7, 8, 10, 11, and 12) (Fig 5).

### 3.4. Identification of candidate odorant receptors

**Fig 6:**
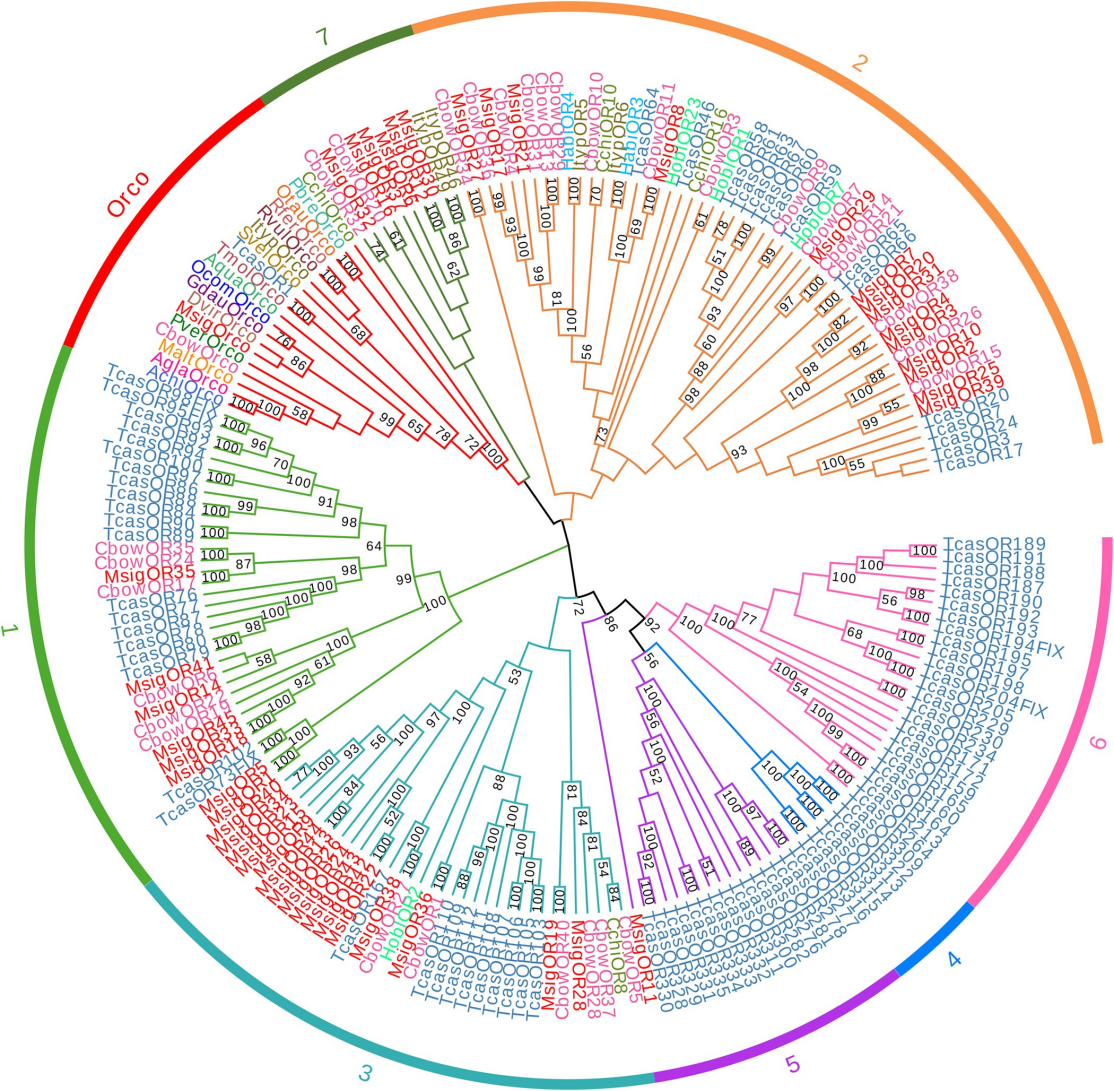
Phylogenetic tree of candidate MsigORs with known Coleopteran OR sequences. Achi, *Anoplophora chinensis* (N = 1); Agla, *Anoplophora glabripennis* (N = 1); Aqua, *Ambrostoma quadriimpressum* (N = 1); Cbow, *Colaphellus bowringi* (N = 31); Cchi, *Callosobruchus chinensis* (N = 4); Dvir, *Diabrotica virgifera virgifera* (N = 1); Gdau, *Galeruca daurica* (N = 1); Habi, *Hylobius abietis* (N = 2); Hobl, *Holotrichia oblita* (N = 4); Ityp, *Ips typographus* (N = 5); Malt, *Monochamus alternatus* (N = 1); Ocom, *Ophraella communa* (N = 1); Otau, *Onthophagus taurus* (N = 1);Pbre, *Protaetia brevitarsis* (N = 1); Pver, Plagiodera versicolora (N = 1); Rfer, *Rhynchophorus ferrugineus* (N = 1); Rvul, *Rhynchophorus vulneratus* (N = 1); Svel, *Sympiezomias velatus* (N = 1); Tcas, *Tribolium castaneum* (N = 91); Tmol, *Tenebrio molitor* (N = 1). Bootstrap values >50 are shown.

We found 46 candidate OR transcripts in the *M*. *signata* antennal transcriptome, including a highly conserved co-receptor (Orco) and 45 typical odorant receptors. Forty-two MsigORs contained a putative full-length ORF, with five to seven TMDs were predicted. The lengths of all of the putative full-length MsigORs ranged from 302 to 459 amino acids (S4 Table). The *MsigOrco* gene was easily identified because it had an intact ORF and seven transmembrane domains, features characteristic of typical insect ORs. The amino acid sequences of *MsigOrco* shared 91% identity with the odorant co-receptor of *O*. *communa*. The phylogenetic tree was generated to infer the relationships between the 46 MsigORs and 151 ORs from 20 species of Coleoptera. The results showed that the OR sequence were clustered into several subgroups according to previous studies. MsigORs were only present within the previously defined coleopteran OR subgroup 1, 2, 3, 5, and 7 as well as the Orco subgroup. Notably, *MsigOrco* was clustered with *DvirOrco*, *GdauOrco*, *OcomOrco*, *CbowOrco*, *MaltOrco*, *AchiOrco*, *AglaOrco*, *AquaOrco*, *TmolOrco*, *SvelOrco*, *ItypOrco*, *RvulOrco*, *RferOrco*, *OtauOrco*, *CchiOrco*, *PverOrco*, *PbreOrco*, and *TcasOR1* which are Orco homologs. In addition, *MsigOR4* and *CbowOR38*, *MsigOR8* and *CbowOR11*, *MsigOR10* and *CbowOR26*, *MsigOR17* and *CbowOR36*, *MsigOR19* and *CbowOR40*, *MsigOR27* and *CbowOR34*, *MsigOR28* and *CchiOR8*, *MsigOR29* and *HoblOR7*, *MsigOR32* and *CbowOR32*, *MsigOR35* and *CbowOR17*, *MsigOR36* and *CbowOR1*, *MsigOR38* and *CbowOR37*, were grouped with a high degree of homology (Fig 6).

### 3.5. Identification of candidate ionotropic receptors

**Fig 7:**
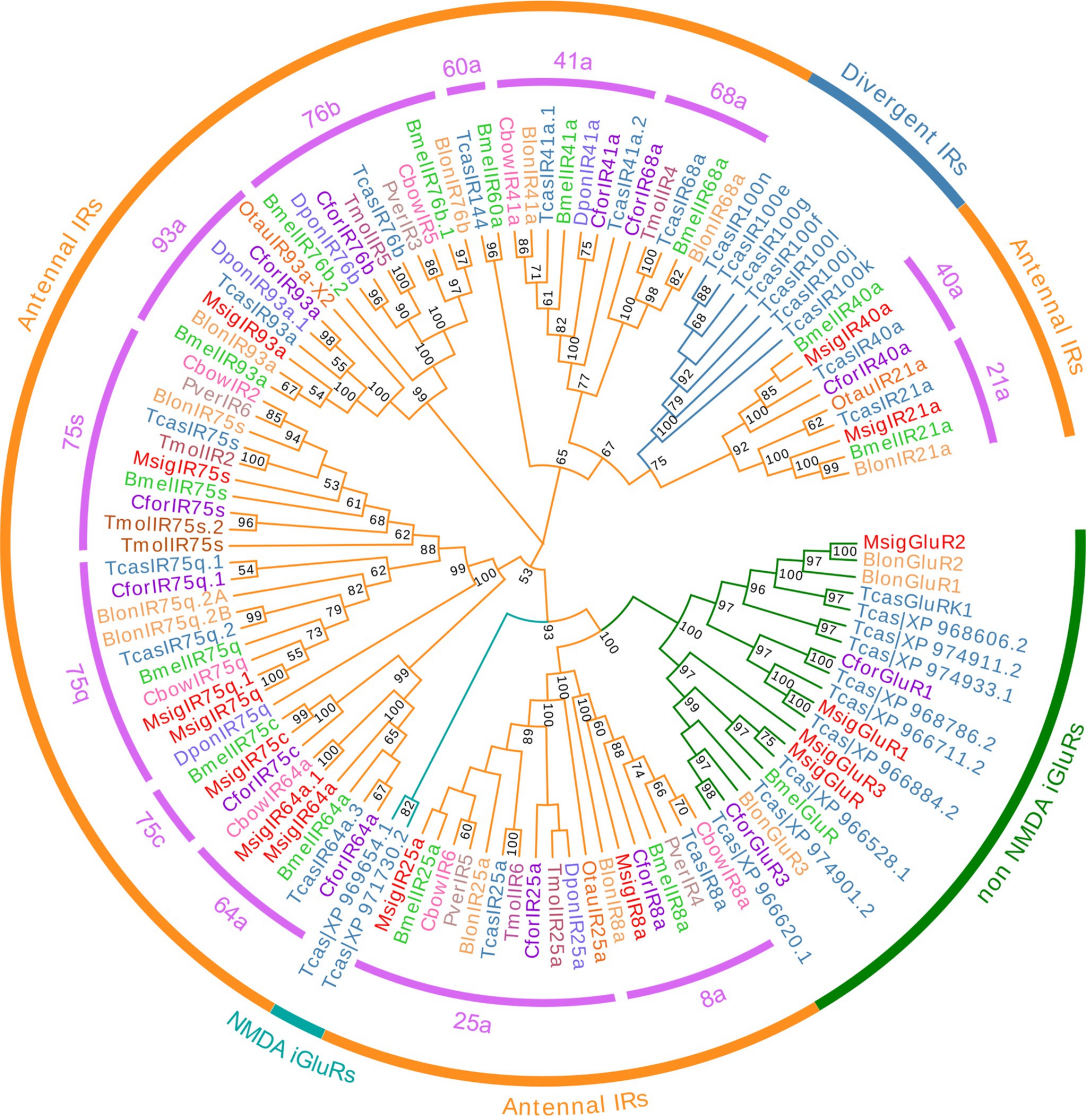
Phylogenetic tree of candidate MsigIRs with known Coleopteran IR sequences. Blon, *Brontispa longissima* (N = 13); Bmel, *Basilepta melanopus* (N = 15); Cbow, *Colaphellus bowringi* (N = 7); Cfor, *Cylas formicarius* (N = 13); Dpon, *Dendroctonus ponderosae* (N = 5); Otau, *Onthophagus taurus* (N = 3); Pver, *Plagiodera versicolora* (N = 4); Tcas, *Tribolium castaneum* (N = 33); Tmol, *Tenebrio molitor* (N = 7). Bootstrap values >50 are shown.

We found 15 candidate IR transcripts in the *M*. *signata* antennal transcriptome. 13 MsigIRs contained a putative full-length ORF, with three to four TMDs were predicted. Lengths of all of the candidate MsigIRs ranged from 283 to 947 amino acids (S4 Table). Based on the Blastp results, MsigIRs had 44–92% sequence homology with previously identified IRs from other Coleopteran insects. A phylogenetic tree was generated to infer the relationships between the 15 MsigIRs and 100 IRs from nine species of Coleoptera. The results showed that MsigIRs were grouped into different clades with high-level bootstrap values. Four iGluR genes were identified, these were named *MsigGluR*, *MsigGluR1*, *MsigGluR2*, and *MsigGluR3*. *MsigGluR2* and *BlonGluR2*, *MsigIR64a*, *MsigIR64a*.*1* and *BmelIR64a*, and *MsigIR75c* and *BmelIR75c* were clustered together with a high degree of homology (Fig 7).

### 3.6. Identification of candidate gustatory receptors

**Fig 8:**
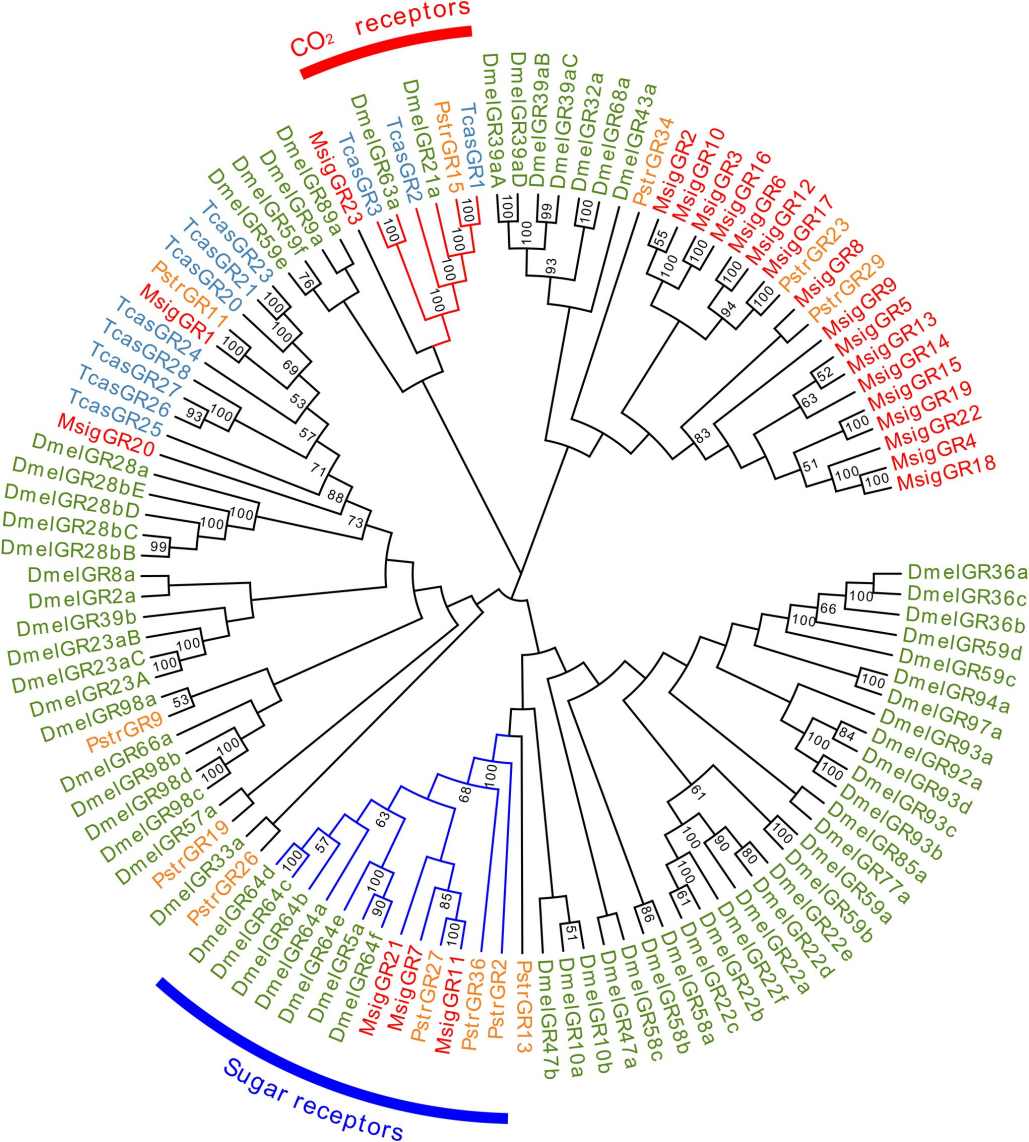
Phylogenetic tree of candidate MsigGRs with known Coleopteran GR sequences. Dmel, *Drosophila melanogaster*(N = 67); Pstr, *Phyllotreta striolata* (N = 12); Tcas, *Tribolium castaneum* (N = 11). Bootstrap values >50 are shown.

We found 23 candidate GR transcripts in the *M*. *signata* antennal transcriptome; these were named *MsigGR1*-*MsigGR23*. Eighteen MsigGRs contained a putative full-length ORF with five to eight TMDs. Lengths of all of the candidate MsigGRs ranged from 278 to 482 amino acids were predicted (S4 Table). Based on the Blastp results, six MsigGRs had more than 50% identity with GRs of *D*. *v*. *virgifera*, *Pyrrhalta aenescens*, *Leptinotarsa decemlineata*, and *P*. *maculicollis*. A phylogenetic tree was generated to infer the relationships between the 23 MsigGRs and 87 GRs of *Phyllotreta striolata*, *Tribolium castaneum* and *D*. *melanogaster*. Notably, three MsigGRs (*MsigGR7*, 11, and 21) were homologous to known sugar receptors, while no MsigGRs were homologous to other known carbon dioxide receptors (Fig 8).

### 3.7. Identification of candidate sensory neuron membrane proteins

**Fig 9:**
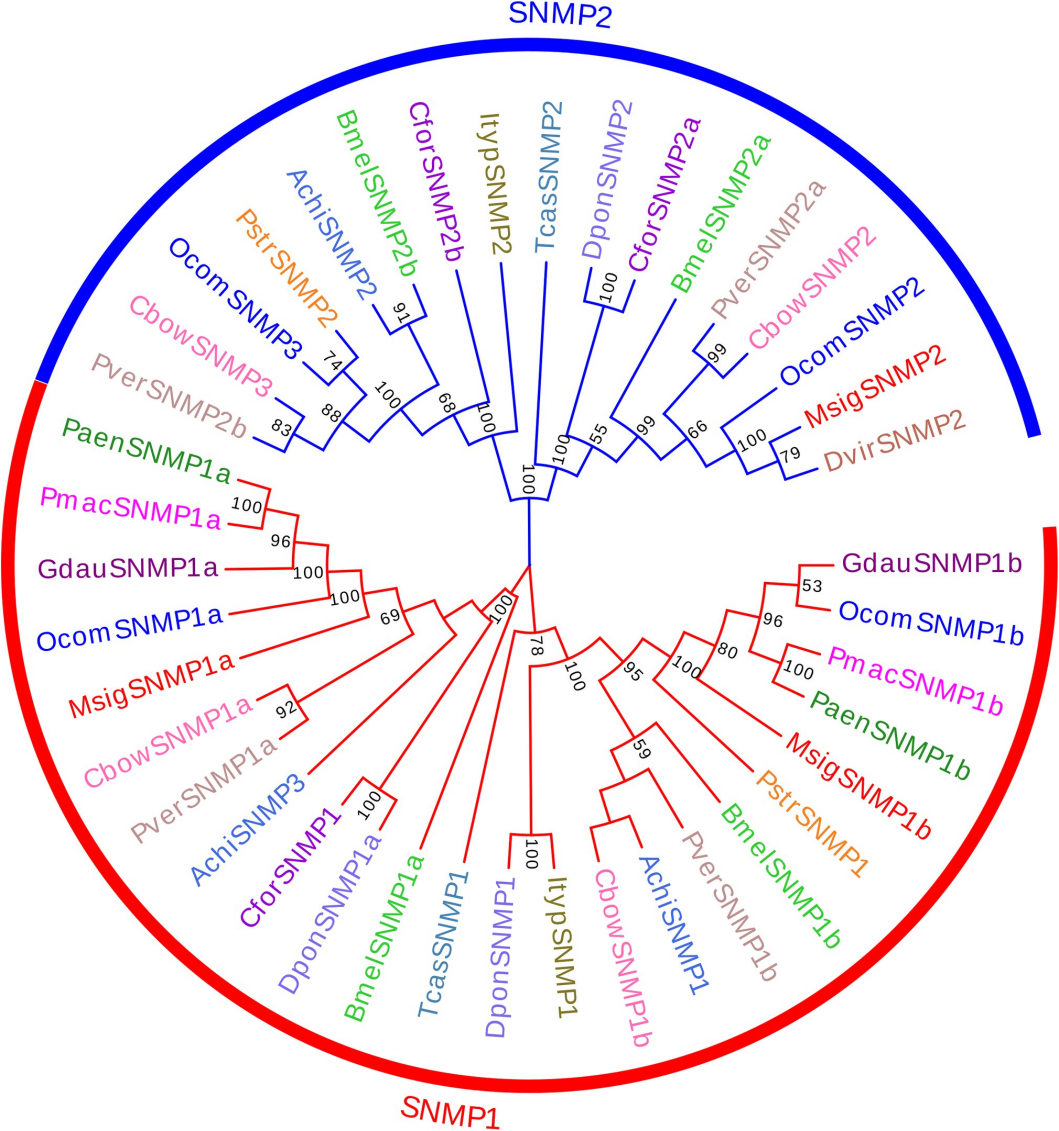
Phylogenetic tree of candidate MsigSNMPs with known Coleopteran SNMP sequences. Achi, *Anoplophora chinensis* (N = 3); Bmel, *Basilepta melanopus* (N = 4); Cbow, *Colaphellus bowringi* (N = 4); Cfor, *Cylas formicarius* (N = 3); Dpon, *Dendroctonus ponderosae* (N = 3); Dvir, *Diabrotica virgifera virgifera* (N = 1); Gdau, *Galeruca daurica* (N = 2); Ityp, *Ips typographus* (N = 2); Ocom, *Ophraella communa* (N = 4); Paen, *Pyrrhalta aenescens* (N = 2); Pmac, *Pyrrhalta maculicollis* (N = 2); Pstr, *Phyllotreta striolata* (N = 2); Pver, *Plagiodera versicolora* (N = 4); Tcas, *Tribolium castaneum* (N = 2).Bootstrap values > 50 are shown.

We identified three SNMP genes in the antennal transcriptome. The lengths of all of the candidate MsigSNMPs were over 522 amino acids, and all were predicted to have a putative full-length ORF (S3 Table). Furthermore, all of the MsigSNMPs had more than 61% identity with SNMPs of *D*. *v*. *virgifera*. A phylogenetic tree was generated to infer the relationships between the three MsigSNMPs and 38 SNMPs from 14 species of Coleoptera. The results showed that *MsigSNMP1a*, *MsigSNMP1b*, and *MsigSNMP2* were classified into SNMP1 and SNMP2, respectively (Fig 9).

### 3.8. Expression levels of MsigOBP, MsigCSP, and MsigOR genes by RT-qPCR

**Fig 10:**
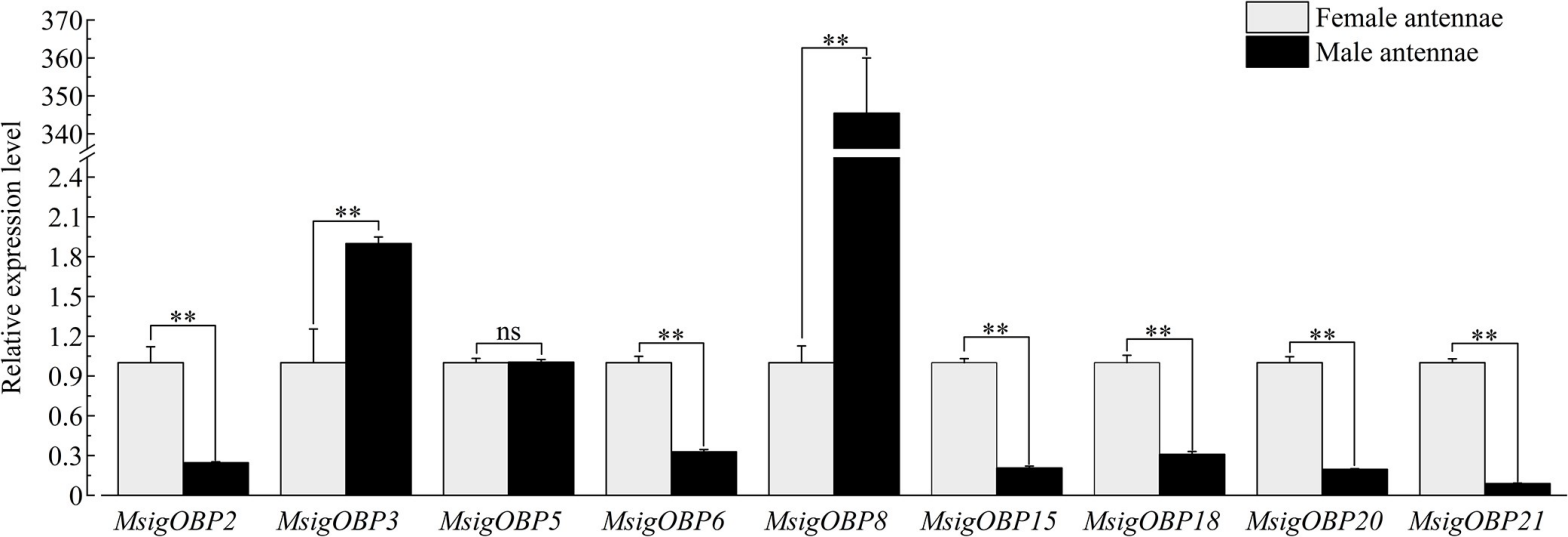
The relative expression levels of OBPs in male and female antennae of *M*. *signata* by RT-qPCR. FA: female antennae; MA: male antennae. The GAPDH gene was used to normalize expression levels in each sample, and the female antennae were selected as the calibrator to normalize the gene expression levels in various tissues. ** Indicates that the difference is extremely significant (*P*<0.01), * indicates that the difference is significant (*P*<0.05), ns means no significant difference (*P*>0.05). Standard errors are represented by the error bars.

**Fig 11:**
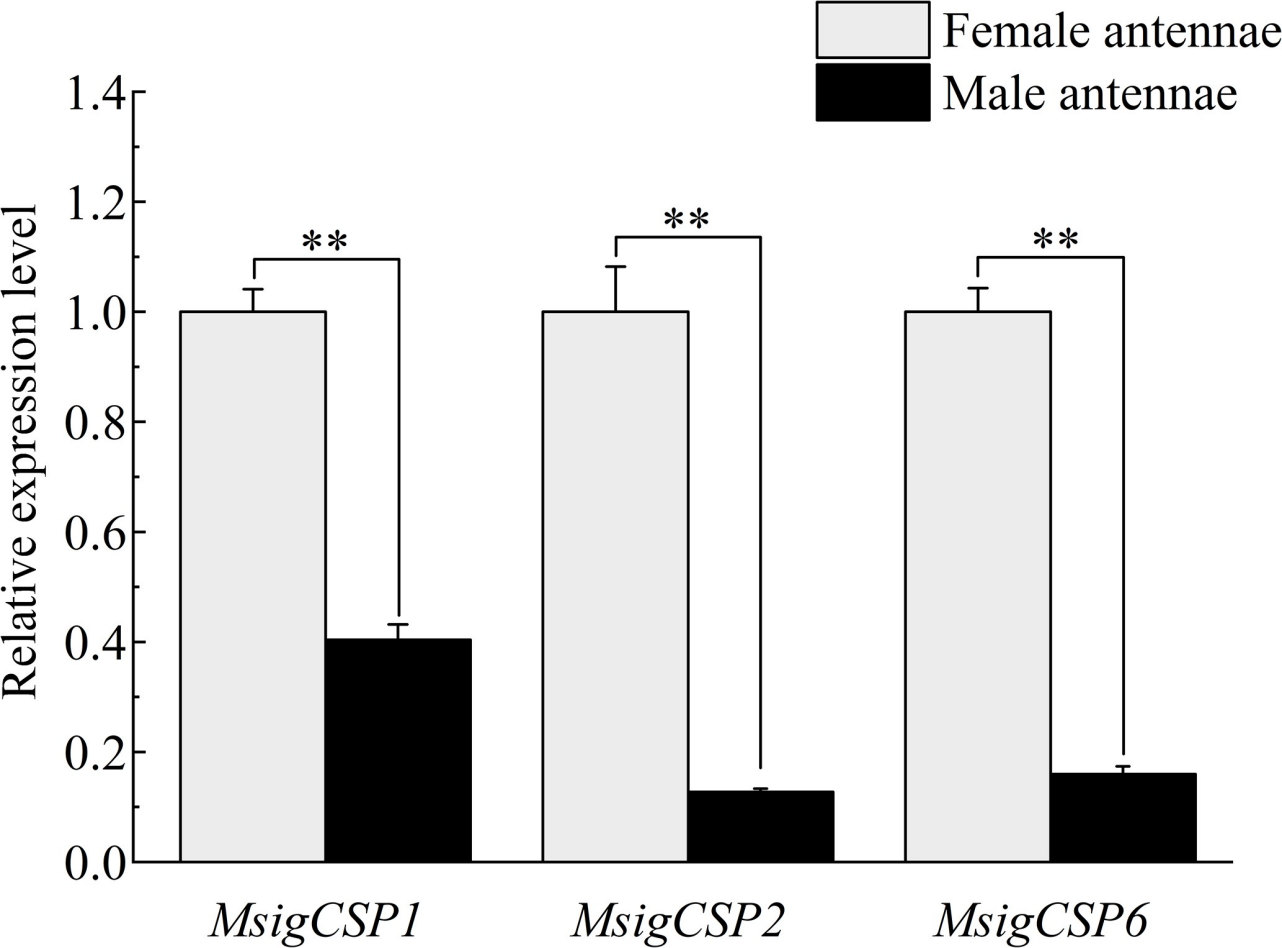
The relative expression levels of CSPs in male and female antennae of *M*. *signata* by RT-qPCR. FA: female antennae; MA: male antennae. The GAPDH gene was used to normalize expression levels in each sample, and the female antennae were selected as the calibrator to normalize the gene expression levels in various tissues. ** Indicates that the difference is extremely significant (*P*<0.01), * indicates that the difference is significant (*P*<0.05), ns means no significant difference (*P*>0.05). Standard errors are represented by the error bars.

**Fig 12:**
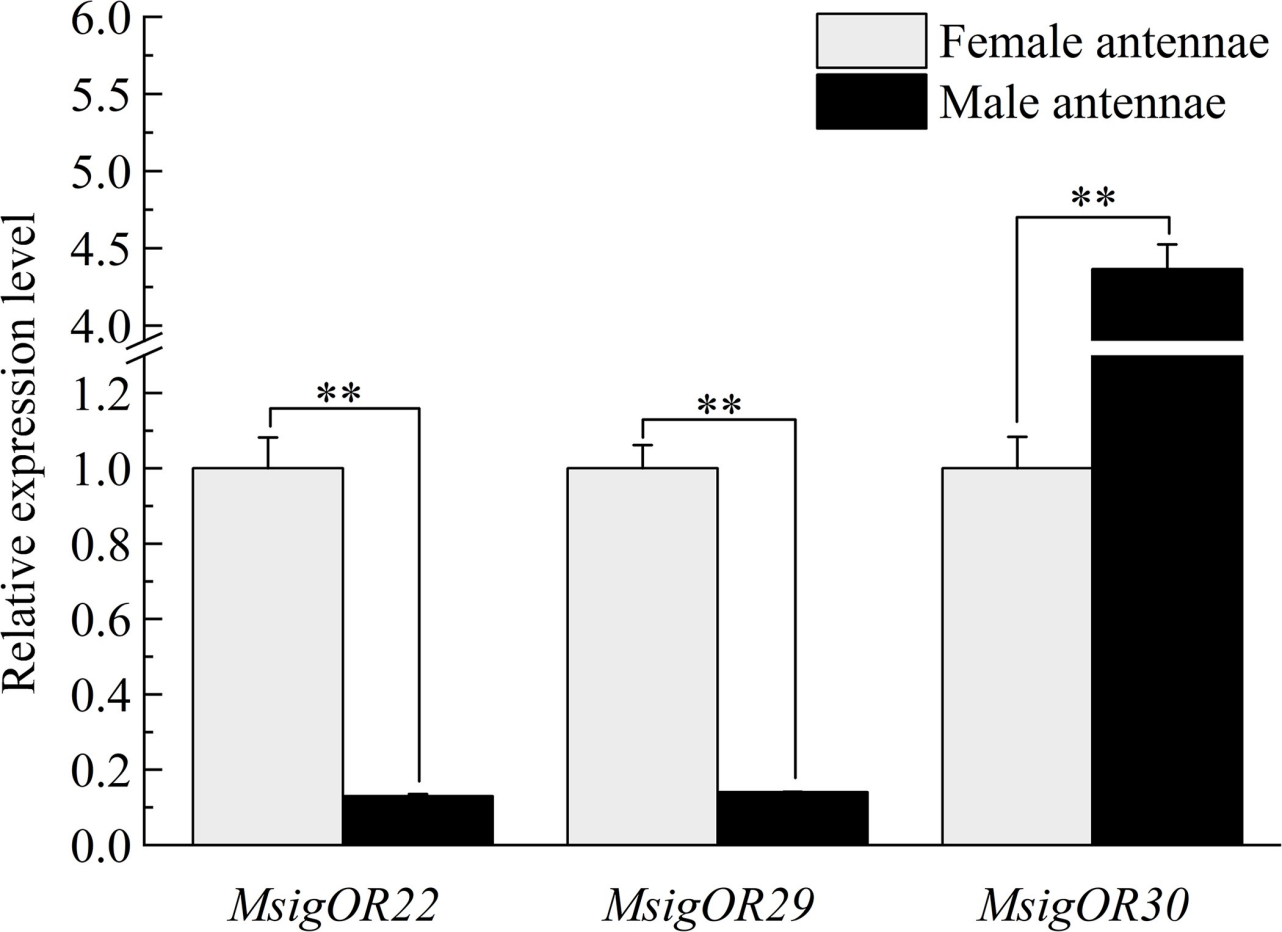
The relative expression levels of ORs in male and female antennae of *M*. *signata* by RT-qPCR. FA: female antennae; MA: male antennae. The GAPDH gene was used to normalize expression levels in each sample, and the female antennae were selected as the calibrator to normalize the gene expression levels in various tissues. ** Indicates that the difference is extremely significant (*P*<0.01), * indicates that the difference is significant (*P*<0.05), ns means no significant difference (*P*>0.05). Standard errors are represented by the error bars.

Some chemosensory genes were selected based on FPKM values and functional annotation for additional RT-qPCR analyses, including nine MsigOBPs (*MsigOBP2*, 3, 5, 6, 8, 15, 18, 20, and 21), three MsigCSPs (*MsigCSP1*, 2, and 6) and three MsigORs (*MsigOR22*, 29, and 30). The RT-qPCR results indicated that six MsigOBPs (*MsigOBP2*, 6, 15, 18, 20, and 21) were expressed at higher levels in the female antennae than male antennae, while only *MsigOBP3* and *MsigOBP8* were more highly expressed in the male antennae than in female antennae. Notably, *MsigOBP5* was not differentially expressed in male and female antennae (Fig 10). The expression levels of *MsigCSP1*, *MsigCSP2* and *MsigCSP6* in female antennae were higher than those in male antennae (Fig 11). *MsigOR22* and *MsigOR29* were highly expressed in the female, while *MsigOR30* was highly expressed in the male antennae (Fig 12).

## 4. Discussion

With the application of a new generation of transcriptome sequencing technology to functional gene mining in non-model insects [27], chemosensory protein genes of more than 30 species of Coleoptera have been identified. As an important agricultural pest, the identification, characterization, and expression analysis of chemosensory genes of *M*. *signata* facilitate a better understanding of the mechanism of the olfactory system in Chrysomelidae of Coleoptera. Based on the antennal transcriptome data, we screened 111 candidate olfactory-related genes and analyzed their sequence characteristics and phylogenetic relationships in *M*. *signata*. These included 21 OBPs, 6 CSPs, 46 ORs, 23 GRs, 15 IRs, and 3 SNMPs. The total number (114) of chemosensory protein genes in *M*. *signata* was greater than what has been reported in *Pyrrhalta aenescens* (92), *P*. *maculicollis* (87) [28], and *Ophraella communa* (105) [29]. This phenomenon may be explained by the evolution of divergent physiological behaviors of different insects during the process of adaptation to various environments [30]. Genetic and phylogenetic analyses were performed on these genes to examine similarities and differences between related genes. Tissue distribution patterns can provide key clues to the functions of olfactory proteins, and it is commonly thought that a high and specific antennal expression pattern suggests a chemosensory role of the genes. The results of this study provide a molecular foundation for further research on the olfactory system of *M*. *signata* as well as a reference for similar studies.

OBPs are a critical first step in the olfactory process of insects. Binding with fat-soluble odor molecules in the environment is the major biochemical mechanism of insect-specific odor recognition [31, 32]. To a certain extent, the differences in OBP reflect the evolutionary process of diverse insect chemosensory systems [33]. We identified 21 transcripts encoding OBP genes in the *M*. *signata* antennal transcriptome. The number of OBPs was clearly lower than in *Rhynchophorus palmarum* (37 OBPs) [34], *P*. *maculicollis* (36 OBPs), and *P*. *aenescens* (31 OBPs) [28] but higher than those of *A*. *quadriimpressum* (16 OBPs) [14] and *Callosobruchus maculatus* (12 OBPs) [27]. Analysis of the properties of the OBP amino acid sequences revealed eight Minus-C OBPs in *M*. *signata*, suggesting that these genes might play an important role in chemosensory function in Coleoptera. The phylogenetic analysis showed that *MsigOBP15* and *MaltOBP3* were clustered in same branch, suggesting that they may be involved in host plant selection [35]. *MsigOBP2*, *MsigOBP5*, *MsigOBP11*, and *MsigOBP13* were grouped in the same branch; *MsigOBP8*, *MsigOBP14*, and *MsigOBP19* were grouped in the same branch, indicating that they may be duplicated or have evolved from the same ancestral gene. According to the RT-qPCR results, the expression levels of the six MsigOBPs were higher in the female antennae than in the male antennae, indicating that they may play an important role in the identification of male pheromones or the oviposition process [36]. OBP genes have been developed as an important target for pest control due to their importance for insects to recognize odors in the external environment. Further functional studies of MsigOBPs would be helpful in providing target genes for green prevention and control of *M*. *signata*.

CSPs are involved in the first step of olfaction in insects as OBPs, and hence CSP gene families have received considerable attention [37, 38]. Our survey for these gene families revealed a total of six CSPs in *M*. *signata*. The high-level similarities found in Blastp best-hit results demonstrated that CSPs were highly conserved proteins among insects. Comparing MsigCSPs (six CSPs) gene numbers with those in other Coleopteran species, there were fewer than in *Leptinotarsa decemlineata* (15 CSPs) [12] and *Glenea cantor* Fabricius (14 CSPs) [39], and a similar number of CSP genes in *C*. *maculatus* (seven CSPs) [27]. In the phylogenetic tree, six MsigCSPs were distributed in different branches, suggesting that these genes may have undergone rapid evolution in adaptation to ecological changes. Notably, *MsigCSP2* and *OcomCSP12* were clustered in the same branch, indicating that they may be involved in mediating reproduction [40]. *MsigCSP1* was identified with significantly higher expression levels in female antennae than male antennae, suggesting that it may be involved in the detection of odors that regulate female-specific behaviors. In conclusion, the study of sequence characteristics and expression profiles of MsigCSP genes may contribute in further investigating their specific function.

ORs play an important role in the olfactory system of insects, as they determine the sensitivity and specificity of odorant reception, being the centerpiece of the peripheral olfactory reception in insects [11]. The number of MsigORs (46 ORs) was greater than in *G*. *daurica* (10 ORs) [41] but was similar to *Brontispa longissima* (48 ORs) [42], *Holotrichia parallela* (47 ORs) [43], *Holotrichia oblita* Faldermann (44 ORs) [44] and *C*. *bowringi* (43 ORs) [13]. *MsigOrco* was on the same branch and was highly homologous with Orco of other species. This was consistent with the fact that Orco genes are highly conserved in insects. *MsigOrco* shared 91% identity with *OcomOrco*, indicating that *MsigOrco* could help other MsigORs localize to the dendritic membrane or better associate with odor molecules [29]. Further functional work in *MsigOrco* would allow a better understanding of ion channel formation. The highly conserved sequence of Orco and its unique position in olfactory recognition indicates that it could potentially be used as a target gene for the future development of novel and effective control strategies [45–47]. RT-qPCR results showed that *MsigOR22* and *MsigOR30* were preferentially expressed in female antennae, which may be involved in the regulation of female oviposition.

Compared with ORs, IRs are another type of olfactory-related protein [48]. ORs bind and respond to pheromones and plant volatiles, regulating insect behaviors such as mating and host-plant selection, while IRs respond to acids and amines [49, 50]. A total of 15 IRs were identified in the antennal transcriptomes of *M*. *signata*. This is considerably lower than the numbers found in *R*. *palmarum* (28 IRs) [34], *Harmonia axyridis* (Pallas) (27 IRs) [51], and *Eucryptorrhynchus brandti* (Harild) (25 IRs) [52], but greater than those in *Plagiodera versicolora* (seven IRs) [53] and *Dendroctonus valens* (three IRs) [54]. All of the MsigIRs identified here have orthologs in other Coleoptera. IRs of insects have a variety of functions, including taste, olfactory, temperature, and humidity perception. Like Orco, both IR8a and IR25a were thought to act as co-receptors since they are co-expressed along with other IRs. The phylogenetic tree revealed that *MsigIR8a* and *MsigIR25a* belong to the co-expression IR group. The use of molecular biology, behavior and other methods, proved that the host-seeking behavior of *Anastatus japonicus* is related to peripheral olfactory receptors, and mainly related to the olfactory co-receptor Orco, and not related to ionotropic co-receptors IR8a and IR25a [55]. The molecular mechanisms and functions of most insect IR genes have not been reported, and thus MsigIR genes have a huge research space and potential for functional studies.

We found fewer GRs than ORs in antennae in *M*. *signata* because insect antennae are not primary gustatory organs. The maxillary palps may be responsible for expressing more GRs [56]. GRs are involved in the detection of sugars, bitter compounds, carbon dioxide, and contact pheromones [57–59]. The number of predicted MsigGRs (23 GRs) was higher than that in the antennal transcriptomes of *O*. *communa* (17 GRs) [29] and *P*. *versicolora* (13 GRs) [53]. In the phylogenetic tree, *MsigGR7*, *MsigGR11*, and *MsigGR21* were clustered with the sugar receptor families, indicating that they may play an important role in host plant selection.

SNMPs are transmembrane domain proteins, and their main function is the recognition and transport of lipophilic odor molecules [60]. SNMP genes are expressed in the dendrite membrane of olfactory receptor neurons, and they play an important role in pheromone recognition [61, 62]. Most studies have shown two SNMP genes in insects, namely, SNMP1 and SNMP2. However, multiple SNMPs have been identified in a variety of Coleopteran species. Three SNMPs were identified in the antennal transcriptomes of *M*. *signata*, the same as in *Dendroctonus ponderosae* and *Ips typographus* (three SNMPs) [63]. According to the phylogenetic tree of the SNMPs from *M*. *signata* and various Coleopterans, we observed that *MsigSNMP1a*, *MsigSNMP1b*, and *MsigSNMP2* were clustered separately in a branch, indicating that they may play a different role in *M*. *signata*.

## 5. Conclusions

In this study, to better understand the molecular mechanisms of the olfactory recognition process in *M*. *signata*, a total of 114 candidate olfactory-related genes were annotated and identified in the antennal transcriptome, including 21 OBPs, 6 CSPs, 46 ORs, 15 IRs, 23 GRs, and three SNMPs. As the first step towards understanding gene functions, we conducted a comprehensive and comparative phylogenetic analysis and examined the relative expression levels of some olfactory-related genes by RT-qPCR. Our study provided a foundation for the functional study of olfactory-related genes in *M*. *signata* and a basis for elucidating the molecular mechanism of olfactory recognition in insects as well as potential target genes for the development of novel behavior regulation techniques based on olfactory recognition.

## Supporting information

S1 Table The estimated expression levels (FPKM value) of chemosensory genes. (PDF)

S2 Table Primer for RT-qPCR of some MsigOBP, MsigCSP, and MsigOR genes in *M*. *signata*. (PDF)

S3 Table Blastp match of *M*. *signata* candidate OBP, CSP and SNMP genes. (PDF)

S4 Table Blastp match of *M*. *signata* candidate OR, IR and GR genes. (PDF)

S1 File The nucleotide sequences of olfactory-related genes in *M*. *signata*. (PDF)

S2 File The amino acid sequences of *M*. *signata* and other insects used in phylogenetic analyses. (PDF)

S3 File The amino acid sequences of Coleopetra insects were used in phylogenetic analyses. (PDF)

S4 File Overview of transcriptome data from the antennae of *M*. *signata*. (PDF)

S5 File (DOCX)
