# Supplementary material for: Identification of candidate chemosensory genes in the antennal transcriptome of Monolepta signata
Source: PLoS One. 2024 Jun 7;19(6):e0301177. doi: 10.1371/journal.pone.0301177 (PMC11161048; doi:10.1371/journal.pone.0301177)
Supplement: S1 Table — (PDF) [file pone.0301177.s001.pdf]

Table S1. The estimated expression levels (FPKM value) of olfactory-related genes.

Gene name	FA1	FA2	FA3	MA1	MA2	MA3
MsigOBP1	32.67	8.58	6.55	15.9	3.92	18.33
MsigOBP2	6965.55	8773.39	8338.09	6192	5917.29	5950.01
MsigOBP3	3096.25	5618.23	5230.9	25750.26	35692.09	28808.74
MsigOBP4	6.83	3.25	2.73	11.96	16.66	0
MsigOBP5	90.91	63.76	250.61	165.15	121.58	274.37
MsigOBP6	581.27	475.57	579.24	378.71	276.08	311.36
MsigOBP7	0	1.17	0	6.44	20.2	0
MsigOBP8	3.72	0.91	0	16.8	74.79	0
MsigOBP9	1.64	5.5	2.95	1.36	2.78	2.98
MsigOBP10	5.13	7.03	6.51	5.35	5.52	3.91
MsigOBP11	41.35	39.62	37.92	22.76	27.86	20.71
MsigOBP12	2299.42	2661.88	3201.12	2678.64	3537.41	3798.01
MsigOBP13	3.58	5.4	12.56	3.88	1.09	4.99
MsigOBP14	0	2.85	0	6.11	8.68	0
MsigOBP15	53.5	60.21	58.03	43.47	48.25	37.86
MsigOBP16	5.56	9.24	19.65	23.85	15.6	13.35
MsigOBP17	8.64	5.55	8.01	7.32	11.63	2.16
MsigOBP18	134.34	152.42	124.96	110.56	170.29	132.47
MsigOBP19	0	0	0	10.57	11.02	0
MsigOBP20	9365.12	8941.88	10583.78	7682.89	7579.62	9383.83
MsigOBP21	1061.26	967.03	1379.29	429.81	369.74	356.37
MsigCSP1	659.92	391.24	663.73	448.64	353.18	435.29
MsigCSP2	210.66	232.96	186.61	180.94	137.23	167.1
MsigCSP3	0.62	2.18	1.07	0.63	1.04	0.75
MsigCSP4	8.15	9.46	5.05	5.6	6.19	7.43
MsigCSP5	5.69	1.14	2.01	0	6.94	0.55
MsigCSP6	2136.67	1840.63	2099.73	1814.18	1571.73	2015.85
MsigOrco	19.81	43.2	24.93	47.21	9.9	38.13
MsigOR1	3.23	2.54	1.96	1.77	1.35	0.56
MsigOR2	0.41	1.03	0.85	0.24	0.79	0.53
MsigOR3	10.2	10.67	11.7	7.38	6.99	4.71
MsigOR4	3.42	3.54	3.96	2.79	2.25	1.84
MsigOR5	3.43	4.28	3.8	1.38	1.91	1.89
MsigOR6	1.38	2.92	2.05	2.13	2.47	1.77

MsigOR7	9.06	8.32	9.54	5.34	4.95	4.57
MsigOR8	4.49	4.07	3.45	1.99	1.57	1.45
MsigOR9	2.84	2.45	2.98	1.37	1.38	1.8
MsigOR10	2.56	1.59	1.91	1.02	0.97	1.37
MsigOR11	4.84	4.5	3	2.32	1.96	2.54
MsigOR12	7.95	8.44	8.2	8.15	11.93	7.97
MsigOR13	11.61	16.55	17.57	8.45	6.17	7.63
MsigOR14	14.33	14.51	14.28	7.96	6.34	5.14
MsigOR15	2.11	2.2	2.38	0	0	0
MsigOR16	4.28	3.47	4.31	2.14	3.66	2.37
MsigOR17	9.01	9.19	8.02	5.62	8.11	5.84
MsigOR18	5.93	6.8	9.52	4.96	4.36	5.05
MsigOR19	46.64	50.02	52.91	36.64	38.81	27.33
MsigOR20	20.76	17.57	20.04	14.77	13.08	14.78
MsigOR21	17.36	10.64	12.88	11.1	10.66	9.99
MsigOR22	70.78	71.36	80.34	48.53	36.55	35.86
MsigOR23	1.76	0.94	1.13	14.97	13.68	17.17
MsigOR24	3.84	3.74	3.64	2.16	2.62	1.97
MsigOR25	14.21	17.49	16.55	6.7	6.96	8
MsigOR26	10.01	9.09	11.61	2.23	4.73	5.54
MsigOR27	6.73	6.37	6.95	4.97	3.42	4.26
MsigOR28	8.11	11.62	13.38	2.34	3.83	3.62
MsigOR29	32.67	39.53	45.14	25.13	29.14	17.64
MsigOR30	39.95	43.01	60.45	509.72	519.21	465.32
MsigOR31	14.21	16.24	14.02	10.82	10.4	10.13
MsigOR32	4.78	3.91	4.27	3.15	2.88	2.17
MsigOR33	20.79	17.98	22.93	12.03	11.78	12.71
MsigOR34	5.66	5.81	6.44	1.39	1.17	1.26
MsigOR35	1.52	1.3	1.25	1.08	1.49	0.77
MsigOR36	4.53	3.05	3.1	1.67	1.95	1.78
MsigOR37	12.15	8.8	12.17	7.15	5.99	4.55
MsigOR38	7.81	6.33	7.36	6.25	3.83	4.68
MsigOR39	6.28	8.14	5.78	5.17	5.14	4.68
MsigOR40	5.92	5.5	6.37	38.37	31.84	31.38
MsigOR41	4.14	4.93	4.05	2.59	2.47	4.26
MsigOR42	0.98	0.67	0.39	0.83	0.42	0
MsigOR43	0.74	0.43	1.01	0.9	0.98	0.49

MsigOR44	2.2	2.11	2.72	1	1.34	1.76
MsigOR45	4.4	4.49	4.46	1.93	2.03	2.96
MsigGluR	1.64	1.82	1.08	1.17	0.75	0.52
MsigIR25a	20.82	34.37	35.27	35.58	21.94	25.45
MsigIR8a	4.9	3.7	6.87	3.18	1.47	2.27
MsigIR40a	1.19	0.44	1.06	0.95	0.79	0.25
MsigIR64a	15.46	21.48	20.83	14.01	11.38	10.46
MsigIR21a	5.21	4.34	5.72	2.93	2.96	2.07
MsigIR64a.1	11.45	12.25	13.41	7.91	7.5	6.2
MsigIR75s	40.57	30.25	42.46	32.29	30.2	22.24
MsigGluR2	18.91	9.03	11.32	8.56	6.34	4.47
MsigIR75q	26.7	37.8	43.92	27.69	22.27	17.05
MsigGluR1	1.35	0.83	1.13	1.19	1.6	0.88
MsigIR93a	8.72	6.42	7.85	5.8	4.44	4.55
MsigIR75q.1	9.54	12.38	11.54	8.95	7.8	5.6
MsigIR75c	4.24	3.72	4.55	1.86	2.6	1.66
MsigGluR3	6.98	3.87	3.46	3.34	2.12	2.2
MsigGR1	0.62	0.7	1.55	1.08	0.77	0.37
MsigGR2	2.04	1.77	1.95	1.36	0.76	0.9
MsigGR3	0.53	0.3	1.51	0.18	0.6	0.87
MsigGR4	3.46	3.95	2.46	2.07	3.86	2.29
MsigGR5	1.99	1.18	2.3	1.73	2.14	1.28
MsigGR6	4.65	3.87	4.32	2.5	3.09	0.99
MsigGR7	0.26	0.62	0.91	0.7	0.45	0.09
MsigGR8	1.65	1.27	2.13	0.75	0.6	0.58
MsigGR9	1.02	0.35	0.79	0.54	1	0.38
MsigGR10	0.8	0.75	0.77	0.81	0.76	0.8
MsigGR11	0.63	1.46	0.66	0.32	0.76	0.58
MsigGR12	2.91	3.19	2.85	2.81	2.76	1.85
MsigGR13	3.01	3.64	2.54	1.64	1.61	1.45
MsigGR14	1.78	1.47	2.15	1.17	1.53	1.17
MsigGR15	1.13	1.03	0.99	0.36	1.12	0.7
MsigGR16	1.48	1.44	1.8	0.27	0.69	0.93
MsigGR17	0.86	0.4	0.37	0.24	0.89	0
MsigGR18	2.14	2.04	1.34	1.35	1.75	1.61
MsigGR19	0.89	0.89	1.19	0.96	0.62	0.55
MsigGR20	2.4	3.05	1.84	1.25	1.76	3.18

MsigGR21	0.95	1.41	1.18	0.89	0.38	0.65
MsigGR22	0.79	1.08	1.2	0.61	0.27	0.66
MsigGR23	1.91	1.65	2.23	2.57	1.43	1.66
MsigSNMP1a	25.56	63.01	36.42	80.47	55.31	83.06
MsigSNMP1b	9.89	19.48	14.99	18.18	9.74	16.02
MsigSNMP2	12.22	21.9	23.92	13.58	9.12	10.05
