# Supplementary material for: Identification of candidate chemosensory genes in the antennal transcriptome of Monolepta signata
Source: PLoS One. 2024 Jun 7;19(6):e0301177. doi: 10.1371/journal.pone.0301177 (PMC11161048; doi:10.1371/journal.pone.0301177)
Supplement: S2 Table — (PDF) [file pone.0301177.s002.pdf]

Table S2. Primer for RT-qPCR of some MsigOBP, MsigCSP, and MsigOR genes in *M. signata*.

Gene name	Forward primer (5'-3')	Reverse primer (5'-3')
MsigOBP2	TGGTTTCAGCGAACGCAATC	TCCTTCATGACTGTCTGGTTCC
MsigOBP3	TAGAGGCAAATGCACCACCAT	TTGATGAGCTTCCTCTGGCTG
MsigOBP5	AAATGCGGATGGCTCCATGA	TCCCAGTGCTCGTCCTTCTA
MsigOBP6	GCAACCCATTTGTTGCCTCA	TGTACGTGTGTCGGACAGTG
MsigOBP8	CATCCATGCGCCTTTCAAGA	GCCAAAGAAGAGTGTCAAGCTG
MsigOBP15	TGGGAATCTTAGCTGGTGGC	CACACCAATTTCATCGAAAACGC
MsigOBP18	AGGACTCCAAAATGAAGACGGT	ACAAGGACACGTTTGGGGTT
MsigOBP20	ACGGTTGCGTCGTCTATTCC	AAGATCCAGAAACACGAGCGG
MsigOBP21	TGGACACCAAGGCATCTCAC	TCTGAGGCGCCTGAATAACA
MsigCSP1	TCGCAATCATTTTCCAATGCGT	TCGTCGTTTCGATAGGCGTT
MsigCSP2	TGGCGAGGGAGAAGAACTGA	AATGGTGGCCTGCATCGTAT
MsigCSP6	TGTTGCCGCAAAATGAAGACA	AATCCGTCCGGCGTACATTT
MsigOR22	TTGCAAATGTGCATCGTGCT	CACTCGCAAAGAACCCCTCA
MsigOR29	CGCATTCGTGCCAATTCGAT	TGGGATTTCAGTTGGCCGTT
MsigOR30	GCGATGTTCGTGCGTATTCC	CTTGCAATGCGTGGACTCTG
GAPDH	ATGCGTTGTTCATGGGTGGT	CGGCCGAAACCGTTAATTCC
