# Supplementary material for: Identification of candidate chemosensory genes in the antennal transcriptome of Monolepta signata
Source: PLoS One. 2024 Jun 7;19(6):e0301177. doi: 10.1371/journal.pone.0301177 (PMC11161048; doi:10.1371/journal.pone.0301177)
Supplement: S3 Table — (PDF) [file pone.0301177.s003.pdf]

Table S3. Blastp match of *M. signata* candidate OBP, CSP and SNMP genes.

Gene Name	ORF length(bp)	Complete ORF	Signal Peptide	Best Blastp-hit				
				Name	Acc-number	Species	E-Value	Identity (%)
MsigOBP1	136	Yes	Yes	odorant-binding protein 27	APC94275.1	*Pyrrhalta aenescens*	2×10^{-30}	39.10
MsigOBP2	149	Yes	Yes	odorant-binding protein 14	APC94205.1	*Pyrrhalta maculicollis*	1×10^{-48}	58.39
MsigOBP3	139	Yes	Yes	odorant-binding protein 72-like	XP_028137022.1	*Diabrotica virgifera virgifera*	4×10^{-66}	72.34
MsigOBP4	137	Yes	Yes	odorant-binding protein	AQY18985.1	*Galeruca daurica*	2×10^{-61}	62.77
MsigOBP5	154	Yes	Yes	odorant-binding protein 19d-like	XP_028134286.1	*Diabrotica virgifera virgifera*	5×10^{-67}	64.43
MsigOBP6	180	Yes	Yes	odorant-binding protein	AQY18976.1	*Galeruca daurica*	3×10^{-31}	37.02
MsigOBP7	137	Yes	Yes	odorant-binding protein	AQY18990.1	*Galeruca daurica*	3×10^{-17}	34.31
MsigOBP8	153	Yes	Yes	odorant-binding protein	AQY18990.1	*Galeruca daurica*	1×10^{-19}	41.05

MsigOBP9	148	Yes	Yes	odorant-binding protein 25	APC94185.1	*Pyrrhalta maculicollis*	1×10^{-24}	41.27
MsigOBP10	180	Yes	Yes	odorant-binding protein 31	APC94203.1	*Pyrrhalta maculicollis*	2×10^{-94}	73.18
MsigOBP11	149	Yes	Yes	odorant-binding protein 14	APC94205.1	*Pyrrhalta maculicollis*	1×10^{-50}	55.03
MsigOBP12	144	Yes	Yes	odorant-binding protein 16	APC94207.1	*Pyrrhalta maculicollis*	4×10^{-55}	68.64
MsigOBP13	148	Yes	Yes	odorant-binding protein 19d-like	XP_028134285.1	*Diabrotica virgifera virgifera*	9×10^{-50}	54.67
MsigOBP14	146	No	Yes	odorant-binding protein	AQY18990.1	*Galeruca daurica*	2×10^{-23}	52.94
MsigOBP15	131	Yes	Yes	odorant-binding protein 69a-like	XP_028129817.1	*Diabrotica virgifera virgifera*	1×10^{-52}	86.09
MsigOBP16	126	Yes	Yes	odorant-binding protein	AQY18972.1	*Galeruca daurica*	2×10^{-10}	29.46
MsigOBP17	120	Yes	Yes	odorant-binding protein 18	APC94209.1	*Pyrrhalta maculicollis*	2×10^{-26}	52.10
MsigOBP18	136	Yes	Yes	odorant-binding protein 28	APC94187.1	*Pyrrhalta maculicollis*	4×10^{-41}	52.21
MsigOBP19	153	Yes	Yes	odorant-binding protein	AQY18990.1	*Galeruca daurica*	2×10^{-19}	41.18
MsigOBP20	137	Yes	Yes	odorant-binding protein	AQY18978.1	*Galeruca daurica*	1×10^{-53}	63.85
MsigOBP21	144	Yes	Yes	odorant-binding protein 34	APC94181.1	*Pyrrhalta maculicollis*	2×10^{-22}	35.16

MsigCSP1	126	Yes	Yes	chemosensory protein 4	APC94217.1	*Pyrrhalta maculicollis*	$7×10^{-50}$	67.89
MsigCSP2	130	Yes	Yes	chemosensory protein 2, partial	APC94214.1	*Pyrrhalta maculicollis*	$5×10^{-28}$	62.31
MsigCSP3	234	No	Yes	chemosensory protein	ARM20146.1	*Galeruca daurica*	$4×10^{-64}$	72.09
MsigCSP4	113	Yes	Yes	chemosensory protein 11	ALR72525.1	*Colaphellus bowringi*	$6×10^{-41}$	82.05
MsigCSP5	121	No	Yes	chemosensory protein 8	UMT69260.1	*Ophraella communa*	$2×10^{-32}$	48.76
MsigCSP6	123	Yes	Yes	chemosensory protein 6	APC94220.1	*Pyrrhalta maculicollis*	$7×10^{-50}$	94.05
MsigSNMP1a	539	Yes	No	sensory neuron membrane protein 1-like	XP_028147205.1	*Diabrotica virgifera virgifera*	0	78.59
MsigSNMP1b	534	Yes	No	sensory neuron membrane protein 1-like isoform X1	XP_028147588.1	*Diabrotica virgifera virgifera*	0	82.40
MsigSNMP2	522	Yes	No	sensory neuron membrane protein 2-like isoform X2	XP_028134254.1	*Diabrotica virgifera virgifera*	0	61.13
