# Supplementary material for: Identification of candidate chemosensory genes in the antennal transcriptome of Monolepta signata
Source: PLoS One. 2024 Jun 7;19(6):e0301177. doi: 10.1371/journal.pone.0301177 (PMC11161048; doi:10.1371/journal.pone.0301177)
Supplement: S4 Table — (PDF) [file pone.0301177.s004.pdf]

Table S4. Blastp match of *M. signata* candidate OR, IR and GR genes.

Gene Name	ORF length (bp)	Complete ORF	TM number	Best Blastp-hit				
				Name	Acc-number	Species	E-Value	Identity(%)
MsigOrco	480	Yes	7	odorant co-receptor	QEE83332.1	*Ophraella communa*	0	91.88
MsigOR1	388	Yes	6	odorant receptor 4-like	XP_028146887.1	*Diabrotica virgifera virgifera*	1×10^{-99}	46.49
MsigOR2	302	Yes	5	odorant receptor OR26	ALR72569.1	*Colaphellus bowringi*	3×10^{-84}	47.54
MsigOR3	405	Yes	6	odorant receptor Or1-like	XP_028146119.1	*Diabrotica virgifera virgifera*	9×10^{-118}	58.33
MsigOR4	419	Yes	7	odorant receptor 7	APC94314.1	*Pyrrhalta aenescens*	3×10^{-120}	73.08
MsigOR5	390	Yes	6	odorant receptor 4-like	XP_028146887.1	*Diabrotica virgifera virgifera*	0	73.71
MsigOR6	399	Yes	8	odorant receptor Or2-like	XP_028138470.1	*Diabrotica virgifera virgifera*	2×10^{-110}	47.28
MsigOR7	378	Yes	7	odorant receptor 3, partial	APC94308.1	*Pyrrhalta aenescens*	8×10^{-156}	60.53
MsigOR8	417	Yes	7	odorant receptor 26, partial	APC94330.1	*Pyrrhalta aenescens*	0	80.39
MsigOR9	411	Yes	6	odorant receptor Or1-like	XP_028137519.1	*Diabrotica virgifera virgifera*	2×10^{-69}	35.61

MsigOR10	397	Yes	5	odorant receptor OR26	ALR72569.1	*Colaphellus bowringi*	7×10^{-149}	57.64
MsigOR11	382	Yes	6	odorant receptor 25	APC94326.1	*Pyrrhalta aenescens*	1×10^{-33}	28.07
MsigOR12	402	Yes	7	odorant receptor 49b-like	XP_028143789.1	*Diabrotica virgifera virgifera*	2×10^{-104}	39.44
MsigOR13	404	Yes	7	odorant receptor 30a-like isoform X1	XP_028137516.1	*Diabrotica virgifera virgifera*	2×10^{-81}	36.46
MsigOR14	393	Yes	8	odorant receptor 4-like	XP_023017287.1	*Leptinotarsa decemlineata*	4×10^{-84}	37.63
MsigOR15	403	Yes	6	odorant receptor 30a-like isoform X1	XP_028137516.1	*Diabrotica virgifera virgifera*	4×10^{-73}	33.74
MsigOR16	373	Yes	5	odorant receptor Or2-like	XP_028137025.1	*Diabrotica virgifera virgifera*	2×10^{-58}	49.18
MsigOR17	424	Yes	7	odorant receptor OR36	ALR72579.1	*Colaphellus bowringi*	0	68.32
MsigOR18	383	Yes	7	odorant receptor OR19	ALR72564.1	*Colaphellus bowringi*	5×10^{-91}	41.99
MsigOR19	375	Yes	5	odorant receptor 30a-like	XP_028129931.1	*Diabrotica virgifera virgifera*	0	80.00
MsigOR20	403	No	6	odorant receptor 3, partial	APC94308.1	*Pyrrhalta aenescens*	0	71.61
MsigOR21	426	No	7	odorant receptor OR36	ALR72579.1	*Colaphellus bowringi*	7×10^{-162}	58.38
MsigOR22	381	Yes	6	odorant receptor Or1-like	XP_028137519.1	*Diabrotica virgifera virgifera*	2×10^{-92}	40.60

MsigOR23	420	Yes	8	odorant receptor Or1-like	XP_028137519.1	*Diabrotica virgifera virgifera*	7×10^{-70}	31.41
MsigOR24	403	Yes	6	odorant receptor Or1-like	XP_028137519.1	*Diabrotica virgifera virgifera*	7×10^{-110}	42.37
MsigOR25	375	Yes	7	odorant receptor 13	APC94240.1	*Pyrrhalta maculicollis*	0	67.84
MsigOR26	394	Yes	6	odorant receptor 30a-like isoform X1	XP_028137516.1	*Diabrotica virgifera virgifera*	1×10^{-64}	35.88
MsigOR27	459	Yes	6	odorant receptor OR34	ALR72577.1	*Colaphellus bowringi*	4×10^{-118}	43.61
MsigOR28	380	Yes	5	odorant receptor 24a-like	XP_028145513.1	*Diabrotica virgifera virgifera*	1×10^{-81}	36.36
MsigOR29	325	Yes	6	odorant receptor 21, partial	APC94321.1	*Pyrrhalta aenescens*	7×10^{-119}	55.69
MsigOR30	402	Yes	6	odorant receptor 49b-like	XP_028143789.1	*Diabrotica virgifera virgifera*	1×10^{-112}	41.22
MsigOR31	399	No	6	odorant receptor 3, partial	APC94308.1	*Pyrrhalta aenescens*	2×10^{-163}	58.54
MsigOR32	374	Yes	6	odorant receptor Or2-like	XP_028133165.1	*Diabrotica virgifera virgifera*	3×10^{-87}	65.53
MsigOR33	384	Yes	7	odorant receptor OR19	ALR72564.1	*Colaphellus bowringi*	2×10^{-79}	37.56
MsigOR34	418	Yes	7	odorant receptor Or1-like	XP_028137519.1	*Diabrotica virgifera virgifera*	6×10^{-67}	31.58
MsigOR35	387	Yes	6	odorant receptor 19	APC94313.1	*Pyrrhalta aenescens*	0	73.90
MsigOR36	356	Yes	5	odorant receptor OR1	ALR72546.1	*Colaphellus bowringi*	2×10^{-50}	32.42

MsigOR37	391	Yes	6	odorant receptor 49b-like	XP_030759997.1	*Sitophilus oryzae*	3×10^{-50}	30.50
MsigOR38	384	Yes	5	odorant receptor 4, partial	APC94309.1	*Pyrrhalta aenescens*	1×10^{-115}	47.28
MsigOR39	385	Yes	6	odorant receptor OR15	ALR72560.1	*Colaphellus bowringi*	3×10^{-69}	37.27
MsigOR40	417	Yes	7	odorant receptor Or1-like	XP_028137519.1	*Diabrotica virgifera virgifera*	8×10^{-68}	32.95
MsigOR41	388	Yes	6	odorant receptor OR6	ALR72551.1	*Colaphellus bowringi*	4×10^{-64}	34.97
MsigOR42	360	No	6	odorant receptor Or1-like	XP_028137519.1	*Diabrotica virgifera virgifera*	1×10^{-102}	43.68
MsigOR43	401	Yes	5	odorant receptor Or1-like	XP_028137519.1	*Diabrotica virgifera virgifera*	4×10^{-128}	48.02
MsigOR44	433	Yes	6	odorant receptor 30a-like isoform X1	XP_028137516.1	*Diabrotica virgifera virgifera*	1×10^{-66}	35.86
MsigOR45	381	Yes	5	odorant receptor OR19	ALR72564.1	*Colaphellus bowringi*	3×10^{-76}	38.61
MsigGluR	947	Yes	3	ionotropic receptor 8	APC94353.1	*Pyrrhalta aenescens*	0	79.93
MsigIR25a	738	No	2	ionotropic receptor 25a	XP_050508442.1	*Diabrotica virgifera virgifera*	0	92.41
MsigIR8a	651	No	2	ionotropic receptor 8a	KAI2473903.1	*Diabrotica virgifera virgifera*	0	68.45
MsigIR40a	283	Yes	1	ionotropic receptor 40a	KAI2474156.1	*Diabrotica virgifera virgifera*	5×10^{-175}	79.15
MsigIR64a	622	Yes	4	ionotropic receptor 6	APC94350.1	*Pyrrhalta aenescens*	2×10^{-144}	44.95

MsigIR21a	789	Yes	3	ionotropic receptor 4, partial	APC94262.1	*Pyrrhalta maculicollis*	0	70.72
MsigIR64a.1	649	Yes	5	ionotropic receptor 75a-like	XP_044265374.1	*Tribolium madens*	0	55.80
MsigIR75s	559	Yes	3	ionotropic receptor IR2	ALR72541.1	*Colaphellus bowringi*	0	60.84
MsigGluR2	925	Yes	4	ionotropic receptor 1	ANQ46493.1	*Phyllotreta striolata*	0	75.43
MsigIR75q	628	Yes	3	ionotropic receptor 1	APC94347.1	*Pyrrhalta aenescens*	0	64.04
MsigGluR1	933	Yes	3	ionotropic receptor 3	APC94260.1	*Pyrrhalta maculicollis*	0	91.97
MsigIR93a	850	Yes	3	ionotropic receptor 93a isoform X1	XP_028134766.1	*Diabrotica virgifera virgifera*	0	82.51
MsigIR75q.1	574	Yes	3	ionotropic receptor 3	ANQ46495.1	*Phyllotreta striolata*	0	70.60
MsigIR75c	326	Yes	4	ionotropic receptor 75a-like	XP_028135898.1	*Diabrotica virgifera virgifera*	9×10^{-117}	56.75
MsigGluR3	891	Yes	4	ionotropic receptor 6	ANQ46498.1	*Phyllotreta striolata*	0	60.25
MsigGR1	396	No	7	gustatory receptor for sugar taste 43a-like isoform X1	XP_028135349.1	*Diabrotica virgifera virgifera*	0	71.97
MsigGR2	401	Yes	7	gustatory receptor 14	APC94341.1	*Pyrrhalta aenescens*	4×10^{-46}	40.00
MsigGR3	389	Yes	7	gustatory receptor 14	APC94341.1	*Pyrrhalta aenescens*	2×10^{-98}	43.98

MsigGR4	365	Yes	7	gustatory receptor 9, partial	APC94251.1	*Pyrrhalta maculicollis*	2×10^{-27}	29.70
MsigGR5	361	No	8	gustatory receptor 5	APC94250.1	*Pyrrhalta maculicollis*	1×10^{-28}	30.85
MsigGR6	370	Yes	7	gustatory receptor 3, partial	APC94248.1	*Pyrrhalta maculicollis*	6×10^{-60}	58.56
MsigGR7	305	Yes	6	gustatory receptor for sugar taste 64f-like	XP_023022939.1	*Leptinotarsa decemlineata*	1×10^{-112}	62.24
MsigGR8	333	Yes	7	gustatory receptor 9, partial	APC94251.1	*Pyrrhalta maculicollis*	5×10^{-17}	31.78
MsigGR9	394	Yes	7	gustatory receptor 9, partial	APC94251.1	*Pyrrhalta maculicollis*	1×10^{-23}	30.33
MsigGR10	333	Yes	5	putative gustatory receptor 28b	XP_028136855.1	*Diabrotica virgifera virgifera*	2×10^{-74}	44.55
MsigGR11	427	Yes	8	gustatory receptor for sugar taste 64a-like	XP_028144535.1	*Diabrotica virgifera virgifera*	7×10^{-111}	57.93
MsigGR12	245	Yes	6	gustatory and pheromone receptor 39a-like	XP_028143516.1	*Diabrotica virgifera virgifera*	3×10^{-97}	71.00
MsigGR13	362	Yes	7	gustatory receptor 9, partial	APC94251.1	*Pyrrhalta maculicollis*	1×10^{-43}	29.92
MsigGR14	278	Yes	5	gustatory receptor 9, partial	APC94251.1	*Pyrrhalta maculicollis*	1×10^{-7}	27.66
MsigGR15	366	Yes	6	gustatory receptor 5	APC94250.1	*Pyrrhalta maculicollis*	3×10^{-49}	33.97

MsigGR16	331	Yes	6	gustatory receptor 14	APC94341.1	*Pyrrhalta aenescens*	9×10^{-88}	45.99
MsigGR17	372	No	7	gustatory receptor 68a-like	XP_018567270.1	*Anoplophora glabripennis*	1×10^{-41}	31.13
MsigGR18	354	Yes	7	gustatory receptor 9, partial	APC94251.1	*Pyrrhalta maculicollis*	7×10^{-18}	27.97
MsigGR19	368	Yes	6	gustatory receptor 5	APC94250.1	*Pyrrhalta maculicollis*	2×10^{-44}	36.20
MsigGR20	482	No	6	gustatory receptor for sugar taste 43a-like	XP_028146875.1	*Diabrotica virgifera virgifera*	5×10^{-75}	40.00
MsigGR21	388	No	6	gustatory receptor 8, partial	APC94346.1	*Pyrrhalta aenescens*	9×10^{-108}	75.21
MsigGR22	356	Yes	7	gustatory receptor 9, partial	APC94251.1	*Pyrrhalta maculicollis*	3×10^{-28}	29.33
MsigGR23	360	Yes	6	gustatory receptor 123	EFA07620.2	*Tribolium castaneum*	2×10^{-24}	27.27
