# Supplementary material for: Identification of candidate chemosensory genes in the antennal transcriptome of Monolepta signata
Source: PLoS One. 2024 Jun 7;19(6):e0301177. doi: 10.1371/journal.pone.0301177 (PMC11161048; doi:10.1371/journal.pone.0301177)
Supplement: S1 File — (PDF) [file pone.0301177.s005.pdf]

File S1. The nucleotide sequences of olfactory-related genes in *M. signata.*

>Cluster-12689.26038 MsigOBP1

ACATTTGCAGTGGAAAATGAAATTTTATTTGGTATTTCTTTGTTTTTGTTGCACGAGTGT
GTTATCACAGGATTCAGGCACAATCAATCAAATTATAAGAGCTAATCAGAATTGTTCCA
GAAGGACGGGAGTATCACCAGCAATGGCAGCTGGCCTAATGTCAGGTCAATTTCCAGA
TAATGCAGCGTTAAGAAAACACGTTCGATGTATGCTTGAAGAAGTTGGTGTGCAAGAT
TCACAAACTGGTCAGTTACGAATAGAAGAAGTGGAAAGAATGGCGAACGCATTAGCA
CCTTCAAACGTCCCTAGAGCTAATTTAGCACCGGTTATCCGTCAGTGTGCAGTTCAGAG
AGCTACGCCAGACGATACAGCCTACGAAATGGTTAGATGTTTCTATAATGCAGGAAGAG
CAGCATTAAATGGATGACGACATTTAATTTTTAAATAATAATATTAATATTAATTTCGGAT
AAAAATATTGATATATCCGATTGTACTAATCAGAATATAATAGCGGTTGTAGTCCGTAGG
GATTTTTAGATCATTGGTTTTGTTGCTATGAACCGTTGCTTTCGTACATTTGGAGATTAC
AAAAGGCTTTTAAATATTTCACTGTTTTTCTTGACTACTGTCACATTGAGAACTTTCACA
CTCTGTAAAATATATTAAGAACAACTAATATACATTACTTATTTTTTAAGATTGCTTTATAA
ATTGAATGTCAATGTTAATGTAAACAAGACTTAATTAATATATTACGTTTATTA

>Cluster-12689.30761 MsigOBP2

TAAGTGTTAAAGTGTCAACAACTATTAAGAAGAACCATGAAATCCATCGTTATTTTTGC
CCTATTTTTGGTTTCAGCGAACGCAATCGACAGCCAGGTTGTAGATGCATTTGTAGCCA
AAGTAGCTAAATTTTCACAAAAATGTGTGGAAGAAACAAAGGCCACACCTGAGGATCT
AGCCAAACTTATGGCTCGCGAGGAACCAGACAGTCATGAAGGAAAATGTATTGTTTCT
TGTGTATACAAAGCTTTCAAAGTTCAAAATGAGGACGGCACTATGAATTTCGAAGAAA
CCAAGAAATTCATGGAGAAAGTGAAGGAGAGCGATGAGGAAATTTACAACAAACTTA
TGGAAGTTTATAACAAATGTAAAGGAGTAGCTGAACAAGTAGATGATGACCCTTGCATT
ACTGCACTTAATGTCGCCAAGTGTGCAATCAAAGAAGGAAAAGCAGCGGGACTCAGT
AAAGAAGTGTTAGGAATGTAAGCTGTAGAAATGTGATGATGAAGAATTGTGATTTGTTG
ACTAAGTACTTTGAATAAAAAATTACATTACGTCATAGAAAAAA

>Cluster-12689.31568 MsigOBP 3

GTTTTCTTCGAGAAAAATTGTGGTGTTTCTAATGTTTAGTTTTAAATTTAATTAAAACCA
AACCGATAATTTACGTAAACATTTCATATCGTTTCATTCATGCAACGAGGTAAATATAATT
GATTATGAAATTGTTTGTGAGGAAGTAATAATTACCCGTTTCATATAAAGGAAATCGTTT
AAAATGTTGGTCATTCATAACTTCAACGATGAAGTTTATTTGCTTGGGCTATGTTTAGT
TTTGTTTGTACAGTATGGACTTTGCGCGATGTCTGAAAAACAGATGAATGCAACGAAG
AAACTTGTCAGAAACACGTGTACTAATAAAGCAAAAGTAGCCCCAGAAGTTGTAGATG
CGATGCATCAAGGAGATTTTAGTCAGGGACAGTGTTATCTCCTGTGTATTATGAACACT
TATAAATTGCTATTACCTGATGGTACATTCGATTGGGAGGGAGGAATTAAAGCTATAGAG
GCAAATGCACCACCATCGATTGCTGGTCCAGCAGCGGCTAGCGTAAAAAATTGCAAAG
ATGCAATGAAAGACAAGAGCAATAAATGTAATGGCGCCGCAGAAATAGTTAAATGTATA

TATGACGACAATCCTTCCAATTTTTTTTTGCCGTAATTAAATTATTTCCAGCAGCCAGAG
GAAGCTCATCAATTATTATTATTATTTAATTTTTTTTATTACATATATACGATTTATAATGAT
GCCTGCGTAACTTATTTCATTTGTGTAAATAAATTAATGCAAAATAAAAAAAAAAAA
>Cluster-12689.36333 MsigOBP 4
TATTTTTGTATTATATTTTTAATGAAAATTAAAAAAAATGTGAAACACAATTCAATTTACA
ATTTGTGGTAATATTGAATGCCGTTTTCAAAGAAACAAATCCACATCTTGTTGGCAGTT
TTTCCAGGATTTTCATGTTTACGTGAACACTGGCTAACGAATCCACCTACTTTGGATTTG
TCGTGAGTAACCAAAGATATTTTATTCTTCATGGTATCCAAATTAAACTCTCCGTCGTCT
CTCATTAAACCAGCTTTAACTGCCATACATAACATGTGTACGCCAACTTGTCTATCGTCG
ACATTGCTAACAAGATTTCTTAATTTGTCTTCATCGCAAAAAGTACGAGGATTAGATTG
GCAGGAATTGTGAACATCCCGTAGATGTTTTGCTTCACTTGAAGGTAGGTTGGGTAAAC
TGCTAGCCAAAACTGCCGCAATCGCACACAAGAAAACGAAAATAACCTTCATTTTTA
AAAATTTTAAGTAGAGTTCTTTG
>Cluster-12689.26420 MsigOBP 5
GTCAACAAGCATGAAAAATAAAATCATGAAATCCATCGTAATTTTTGCCTTATTTTTGGT
GTCAGTTAACGCGATCGACAGCAAGTTAGTACAAGAATTTGTAGCCCAAGCAACTAAA
TTTGGAGAAAAATGTATTGAAGAAACGAAAGCCTCGAGTGATGATATTGCCACACTTAT
GGCTCATGACATACCTGATAGTCACGAAGGAAAATGTATGATTTCTTGTGTCTATAAAG
CCTTCAAAATCCAAAATGCGGATGGCTCCATGAATTCTGAAGAAACGAAAAAATTAAT
GGAAAAGGTTAAGGAAAGCGATCCAGATATGTACGAAAAACTCGTACAAGTTTTTAAT
AAATGCCAAGGAATTGCTGAACATATCGTTGACGATCACTGTATCACAGCAGTCAATGT
TGCCAAATGTGCCACGGTAGAAGGACGAGCACTGGGACTTAAAGCAGAAATGTTTGG
AATGTAAGCTCTAGAAATGTGATAAATTGTGATTCGTTGATAATGTAACTGTAACAAAAT
ATATTATTATATTACAAAAAAA
>Cluster-12689.31318 MsigOBP 6
CTTCAAATCAACACTGTCTTCAGACAAAAAGTATTTAACTATAACCGTCTATAAATAACT
AAATCTAAAATCAACCAATTACTGATGACTAAATCTACTTCAATTAAACTAATGTAAATT
TAAACTAAATACTAATAAATTGCGCTACCCCACACAGAAAATCTCCAACTCAGTACCTC
GATCCGACACCGACTACAACTGACTCGAAAATGACAATGTTTTCAGCTAAGTGTGTAA
AACTGTTCGGGGTATTCTACGCATAGATGACTCATAGTTCACGACCGATTCTGTCGACT
ATAATAATCGCTGGCGAAAGTTTTTATTAGGACTTGCAACCCATTTGTTGCCTCAGTTTA
CATATATTTGATAACAATCGCGATTACCTTATTTGTTTTAGTATAGTAGTTTCCCTTGCTTT
TCGGTCAACCCATTTTAAATTTGAATTTTATCATCAAAAATGCCTAAACAAAAATCAAC
ACTGTCCGACACACGTACACACGATCTACATACATAACCTGTTAGTTTTTACCCGCGCA
AATTTAGGCGCGCTACGTCAATGATCATGATCACAGACAGCTGTCAACAGAATTCTTTG
GGTTGCCTATCCCAGCACCGCGTAAAAACCATTATCCGGTACCTTTAGTTATTGGGAGA
TCTCTAGCAACGCTGTGGGCAGAGTCAGGGGTTTGACAGTGCTAATTAATGAAAAGAT

GTAGTTTACGCTCTGAATTTAGAACCCATCAAAAAGTAAAACTAAGCTTTACCTTCATT
TACTTCCTGGTAACACTGATCAACTGAATCTTGAAGAAAATTATGTAGTTCTCGAGCGG
ACGCCGTTCGAAGAATTGCTTCCAGTAGTTTAGAATGATCGGGCAGACCATTGGAATCC
AGCATTTGCATGTTATCTAATACGCAATGAATTATGCAATCTTGAGTTACATCTTCCACA
CTAGTTTCTTCCGTATCATCACTGGTATTGGTGTGTTCCGTGCTGTTGTCTGTTGGCGTT
ACTATGCGTGAATTTGTATCGGCGTCGACAGCTCTTTTGGTTTTGCTACTTGTTGATGTA
GAATTTATCGATGTCGGTTGGGAATCGTTTTCTTCAGAAGAATCACTTTCGTCAGAGTC
GTCTCCTTGAAGTAAATGCCATAATTTTTCCGTTTCATTATTTTTTACGCAATGACTTAAT
GCGTTTTTAATTTTTTCTCCATCCGCTTGTTGAAAATCACATTTCAGTGCCTCCATATATC
CTAAAACACAGGTCAAGACCAAAATAATACTACATATTGTCCTCATGTTTTCACAACTC
GCTTTAGACTTCGGATTCAGGTACTAAGAACAAAACTTTGGAAACATTGTTTTTATACT
AAAAGAGACAAATTCGTCCGTTATTTTAAACGATTAAATAATAGGATATATGCTGAATTA
AC
>Cluster-12689.2029 MsigOBP7
AAACGCTATAAACAAACAACACAGGAATATTATTTTTCTTGTATGCAATAATACAAAACT
TAAACTGTTAGTCAACTTGAAAAATTTTGGTTAATTGCTTCCTCTTGCAATTTTTGCAGC
ACAGCTGTGAAGTGCAACTATTGTCTCTTCAGGACTACCACTAGAAATGCTAGCGCATT
TTTCGACAATGGTATTTTCCTTTACTTTATCATTTGTGAAATATTTAGCAACCCATCTTAG
TTTATCTGGATCAACATCACCGTTCTGCTTTACAAGTCCAAGTTGCACGTTTAAGCATTC
AGAGTTTTTCCGAAGTTTTTGTTTATCAACGCGTTCATGTTTTTCAAGTTTTTTTAACGT
ATCGTCATCAAGACGTGAGACTGGATCAGCTTGGCATTTTTCGCGAGCTTTTATAAACT
CATTCTCATCTGCAGCTATGTCTGCAGTCACAGTGGTGACCACAGTGACAAATAAGAA
GATAGAGGCAATGTACAACAAGTGCATCTTTG
>Cluster-12689.8427 MsigOBP8
TGAAATTTGAGTTTAATTAAAGAACGCCATAGTTTAATCATGTTTATGTTCATGATGTCT
GTGATGTCCATGATCATGATGATGTTTATGCAGATGTTTCATCATACATTTATCGAAATCG
ATAGCAAGATCTACGGGAGTTCCAGTACCTTTAACGCTGCATTCTTCAACAATTTTGTT
GATTTTATCTTGATCATCCATGCGCCTTTCAAGAAGACTTTTTAGTTTATCTTTGTTAACT
TCTCCGTTTTCTTGTTGAAGTCCAGTTTTTACATTCATGCACAATGCATGTTTACGAACT
TTTGGATCGTTCATTCTTTCCTCTTTGCTCTTGTCTTTAACTGCATCTTCATCTAATTTTG
TTTCTGGATCAGCTTGACACTCTTCTTTGGCTTTCTTGACTATTGCAACTTCTTCTGCTT
CCTGTTCTGCGGTTGGTGCAGCAGTGACCAAAGTCACAAATAGGACGAGGGCAAAGA
TGTAGAAAATTTTCATGATTAG
>Cluster-12689.33620 MsigOBP9
CGACATTCCATGCGGTTTTTCAATCTTTTAATTATCTACTCAAATAAGCGAAATACTGTC
GGCATTCGATAACTTGGAACACCCAGTCTAAAATTTATTACAGACGAACTCGTCAAATC
TACGATTATCTTCAGTATAACCTTTGCCGTTGTAAAGTGCAAACTGTTGTGTCAGATAAA

TAGTAAACAAATCTTTGGATATTACAAGTTCTCGTTCCAGTATGAACAACTTTCCTTCCA
ATACACCGATAAGGATATTGTTGGCAGTTATGGTTGTTGTTAGCATTTGAAACTTAATAA
TGACTGTCATTTAAATTTCCGTAAATAATAGACGTCTAAAGTTAAATTTCTAATGAATAC
AAAATATTCAGATCATTACGTAACCGTTTATTCATTAAAATAAATTGAGTCTTGTTGACG
AGGTTACAAAATAATATTAGCAACAATATGAAGACGCTCTTATTGCTTTTTTTTACCACT
ATTATCTGTTCCGTTTTTTGCGACCTGAATGGAAAGGGTCTGGGGGATAGATTAAAGAA
AAAAGCGCATAAAATGCACAGCGATTGTCTATTGTATACAGGTGTAACAGATGAGCTAG
TAGAAGGAGTTTTACTTGGACAATTTCCAGACAACGAACCATCTATTCAGCGCTACAAC
TTTTGTATTTGGAACGTAGCTGAAGTGTTCGATGAAAACTACGAGATATCAGAATCAGT
GGCGAGGGAGAACTTGGGCGACTCTATGACGGACGAAGATGTTCTAACCCACGTGAA
GTGTAATAAGAAAGGGCGCGATTTAGGTGGAAACTCGCCGGTGAAGCTAGCCTGGATA
ATGAAAAAGTGTTTATATAAAAACATTCCTAAAGAGCGCTACGCTACGCTACGTTTGAA
TTAAACGCATGGAAACTGAAATACCAAAG

>Cluster-12689.21719   MsigOBP10

ATTTCTTTCGATTATGCGGTATTTTTAAATTAGCGGTGAACATTCAAACTTCAGTAGTTC
TTCGATATATTACACATTACCAGTTCACTGTGAAACATGTTTTGCGGGTTACTTATTTTGT
CTCTAATTAGTTTTGTGGCCGTTATCGGTAACGAACAAAACGAGACGAACTATACCAAC
AGATGTGAAATCCCTCCTTCAGCGCCCAAAAGAGTCGAAGAAGTCATTAACCAATGCC
AAGACGAAATTAAGTTGGCAATTCTTTCAGAAGCGTTGCAGTCATTAAACCTTAACGA
AAATGCACATAGCCGGGCGAAGAGAGCAGCATTTAGTGATGATGAAAGAAGAATTGCT
GGATGCTTGCTTCAATGCGTGTATAGGAAAATGAAGGCGGTCAACGAGACAGGATTTC
CAACAATAGATGGTTTAGTGGCCCTTTACACACAAGGTATTAATCAGAAAGAATATATTC
GCGCCACTATCGAATCTGTCCATGTTTGCCTACGAAGTGCGGAGAAGAAATTTGCGGT
GGTACCAAAAACTTTAGCAGAAGAGCATGGCAAAACATGTGACATTGCTTATGACGTA
TTCGATTGTGTATCCGAAGAAATTGGGAAATATTGTGGACAGTCGCCGTAGAGGAATTT
TGTAATTAGTTTTTCATGTTTTATTTATGTTAGAGTTAAGAAACGTCCTATGTAAAATATC
TATTTAATTTATGTTGTTAGTGTTAAAAAGATGTTAGATATA

>Cluster-12689.14423   MsigOBP11

AGAAGTCTAAGTGTTAAAGTGTCAACAACTATTAAGAAGAACCATGAAATCCATCGTT
ATTTTTGCTCTATTTTTGGTTTCAGCAAACGCAATCGACCGGCAGATAGTGGATGCATTT
GTAGCCAAATTAACTAAATTTGCACTACGTTGTGTGGAAGAAACGAAGGCCACACCTA
AGGATCTTGCTAAACTTATAGCTCACGAAGAACCAGAGAGTCATGAAGGAAAATGTAT
TATATCTTGTGTATACAAAGCTTTCAAAGTTCAAAATAAGGACGGCACTATGAATTTAGT
AGAAGCCAAGAAATTGATAGAGACAGTGAAGAAGAGCGATGAAAAAATTTACAACAA
ACTTATTGAAGTTTATGACAAATGTGAAGGAGTAGCTGAACAAGTAGATGATGACCCTT
GTATTACTGCACTCAATCTCGAAAGGTGTATAATCAAAGAAGGACATGCAGTGGGACT
CAATGAAGAAATATTAAGAATGTAAGCTCTAGAAATGTGATCATCAGGAATTGTGATTT

GTTGACTAAGTAGTTTGAATAAAAAATTAGATTACGTCATAAAATAAAAGTATTTTTCTT
TACAAATTCAATGATTAAGTTAATATAACATGTCAATT
>Cluster-12689.31616 MsigOBP12
CGAGCTCTCTGAGGTCCGGGGTAGGCCTCACTGGTAAGGATGGATAGTCCTAAAGCGG
CTGCATGACTTTGAGGTCCAGATTTCGGGTGATTCTGGTGAGGTTCCGGATTACGGCTG
GGTTCTGAGGTTCGGAGTACGGCTGGTTTCTGAGGTCCTCAGTACGGTTTATTGAAGTT
CGGAGTACGGCTTATTGAAGTTTTCAAAGTACGGCTAATTCTGAGGTGCGGAGTACGG
CTTATTGAAGTTGGGGTCCAAAGTACGGGTGATGCTGAGGTTCGGGGTACGGGCTATC
TGAGGTCCGGAGTACGGGCCGAAACATCTGGCATGCAGAAATATACACACTTTGACAT
TTCATAGGCAGCTTCGCATTTGTCGGTTGCCTTGTCACCTGCGTATCTGCATTTGTCTAT
ACATTCTTTTGCTTCAGTTCTATAAAAGTCGGGTAACTGAGCTATTTGACTCATCGCACT
TTCATAGTTAAAAGTGTTGTCTTTATTTATCAATTTATACATGTTGAATGCGCAATACATG
TAACACTTCGCAGTACGATCAATATCCCAATCTCCTTTGTGCATTTTCTCGACGTCCTCA
ATTGTTGCTTTGGATTTTGGTAGACAAACATTCGTAACCACTTTGACAGCCGCCTTTAC
TTGTTTTTCCGTTAACCCTGTAGTATAAACAGCCATACAGACTATAAAGGTCAACACGG
CAAGACATTTCATTTTACACAATAAACAAATCTATGAATGAGTCGTAGAATCTTACGAA
AAATGAATGAGAATCTCGATCCGCTGTCTATTTGAATACGATCGAGACCTGGAAAACGC
GTCGGCAATTATT
>Cluster-12689.54689 MsigOBP13
GAAACATCTAGTAATTTCAAAAATTAAATCATGAAATCGATTGCAATTTTTGCCTTATTT
TTGGTTTCAGTTTACGCAGGCGATGACGCAATTAAAGAATTTGCAGCCAAAGCAAGTA
AAATTGGGGAAAAATGTGTGGAAGAAACGAAAGCCACAAGTGAGGATGTTGCCATAC
TTATGTCTCATAACATACCCGACAGTCACGAAGGACAATGCATGATTTCTTGTGTCTAC
AAAGCTTTCAAAATGCAAAATGCGGATGGCACCATGAATGCTGAAGAAATCAAGAATT
TGATCGAGAAGGTAAAGGATGTCGATATGGACATGTACAACAAACTGAATCAAGTTTTT
GATAAATGTCACGCAGTTGGTGAACAAGCAGAGGCAGATCATTGTGTAGGAGCAGTAA
AAGTTGCCAAATGTGCAATGACAGAAGCAAAAGCAGTGGGACTCAACGGACAAATGA
TAGGAATGTAAGCTTTGAAAGTGTGTTTAATAAGCATTATTATTTGTTGACTATGAATTTT
TA
>Cluster-3334.0 MsigOBP14
AAGTTAATGAAAAACATGAAGTTCTTCTGCATTGCTGCCCTCGCTTTGTTTGTAGCTGT
GGCAACGGCTGCGCCAACAGAAGCTGAAAGGGACGCTATTAAGAAGGCTCACGATGC
GTGCCAAGCTGATCCAGCCACACGTATAGACGAAGCTACAATCGAAAAACTGAAAAA
CCACGAAAAAGTTGATCGCACCGTCCTTAACAAACACGCCCTGTGCATGAATGTAAAA
AATGGACTTCAACAAGAAAACGGGGACATTAATAAAGAAAAACTTAGAAGTATTGTCG
AAAGACATGTAGATGATAAGGACAAAGTCGACAAAATCGTCGAAGAATGTGGAAACA
GAGGCAGCGCAACCGCAGAAGACGCAGCTGTTGCTCTTGACAAATGTCTTGAGGACG

CAGCAGGACGACCACGTCATCACGGACCACCCGGAGGTGGA

>Cluster-12689.26435 MsigOBP15

AGAAGTTTAAAAAATGCTTAGGTTTATTATTTTGGGAATCTTAGCTGGTGGCGCACTTG
CTTTCACTGAAGAACAAATTCAGTTGATGAACGATCTGCATGCTGAATGTGTTTCACAG
ACTGGAGTTCCTGAAGACCTCATCGATAAAGCTAAATCTGGCGATTTTGTACAAGATCC
AAAAATAAAATGTTACATGAAATGCGTTTTCGATGAAATTGGTGTGATTGACGATGATG
GAAAAATTGACATTGAAGGTGCCTTGGCTATTTTACCTGATGAGTTGAAAGATATCGCA
ACTCCTGTAATTAAATCGTGCGGAACTCAAGCCGGTGCCGACGTTTGTGAAGCAATTTT
CAATACATTAAAATGTTATTACGATACCGATAAAAGGGCGTTCTTCTTGCCGTAGAGAA
ACAAAAATTAATCAAGATCGTCCCCTACTTCTAATAAGTTTTAATTCAAATGTATAGATA
GGTACAAAATATTCCATGTGGCATTGTTATAAATCGATTAATATGTTTTGTTATTAAATAA
TTAATTAACTGCTATTGCTATAATGAGTAGTCACTATACCTTAAAGATACCACTTTATACA
AAATCTGAGAATTATTGACTAACTAAAAATATAGTTCTGCTTTCCTTCCCATTGCAAACC
GACTGGACATCCTTTGACGACGTCAGATTTTCACAATTTGAAGAATTTCAGAATTCTTA
CTTGTAACCCTCAATTAACTGAAAATTCAAATGCATTCTCAGCAATGCCTATGACTAAA
AAGTTCAACTCCTTTTAGGTACTTGGGTAGCATAAAACTAATGATAATAATTAACAACAT
TTCCTTACACAAGAACTGGAGAAAGAAATAATAAATGCATCATAAAGATTTATTATTTTA
ACTCAAACCAAAACATTTTATATTTAATGACCCGTTAAAATTTAATGGTTAGTGGATTTA
AGTCACCCTAGTAAATAGATACCCACTTTCTTTCGTAGATGTCGCTGACTTTCAGTTTAT
TAAGGATTACAAGTACTTAGACTTAAATCAGTCTCAGTAATATTAACTGACACCCACGT
ATTAGTAATATAAAATTAAATTGGTTATAGTTGAAACAACTATTCCGACACTAGTCCAAG
TATGTGTTGTAGCAATCTGAAGAAAGCACAACCAACTCGAGAAAAGGAACAGAAATT
TATCTTGAACCAAATAAATGCCTGCACCAAATCTGAATAAACCTTATTTTGGCTCTAGTC
AGCAATAGGTAGGCAAATTCGATGCAAGGTATAATGCATACAGGACTTCTCAGTTCTCT
TTCTGTGCATGAATATACAATAATCCAAGTTCCACCAATTTGTCGCGTTGTTCATTTGTT
AAGCCG

>Cluster-12689.4464 MsigOBP16

TTAAACGAGCAGCATAAAATGACAAAAATAATTTTGTCAGCAATACTCGTTACACTTTT
TTATATGACTGTTTCACTGGGTCAAGAGGAAACAATAAAAAAATGTATGAAAGAAACT
GGAGTAACTAAAGAAGATGCGGAAACCAAACCACCCAAAAACACTCCGGCAATACGA
TGTTTTTTGAAATGCTTGCTGACTACAAAAGGACAACTATTGGCAAGCGGCAAATTAG
ATTTGGATAAACTCACGGAAGTTTTCAAGAAAATTGACGCATCTGACAGTGATAAAGA
AGAGGTCAAAAAATGTTTATCTACAATAAAAATTGAAACATGTGATGACATACAGCAA
ATTTCGGAATGTTGGGGTGATCTTGCGCAGACGCCTGGTGCAATAATTTAATTCAATTTT
AATTCTTTAAGTATTTTAAAATATAAAAGAACCTTTTACAGTAATAATAATATGTATTATTT
GAGTTAACGTTTTTGTTGTTTTAAATAATAAATTAGGTAGGAGAATAAAA

>Cluster-12689.36272 MsigOBP17

GAATAAACAAGTGCTAAAATGAATTACAGTTCTTTTTTTATTACTTTTGTTGGTGTAGTA
TGTTGTATTCTTAATGATTATTCTGCGGAAGCTCAAAATAATACCGTAAGAGAGCACTGT
ATCAAAGAAGTGGGCATTAGCAAAGAGAAAGTAGCCAAATTGGAAGATGACACCTTG
AAGGACATTGATGAACAGTGTAACTGTTACTTACGTTGTGTGTTTCTCGATATTGGAGC
TATAGATGCGAAAGGTAAGGTGAACGTAGAGAAAACTCTCAAGGAATTTAAAGACGTT
TTCGATGCAGATTGTTTAAAGAAAGTGCCGAAAATTATAGAATGTAATGATATGGCAGC
TCTGGATGACTGCGACAAAACACGTTGATTTATATATAATTATATATTTAGTAAACAGGTT
TACAATGTATAAGATCAAAATAAAAAATGTATAATATTTAAATAAAC
>Cluster-12689.34706 MsigOBP18
GCTGAATATCGGAAAATAATAATCATGAAATTTTTAACATCATTGTTGGTGATTATTGGT
GCTTGTTATGTCAGTGCTAATATCACACAAGAACAACATGAGAAAGTAATGGGATACCA
TAAAGATTGCGCTAAAAAATCTGGTGTTAATTTGGAACTGGTTAAACAAGCAAAAGAA
GAAGGAAAATTTGTCGACGACGATAAATTCAAAATCCAACTCTTCTGTGTTTCTCAAA
AACTAGGACTCCAAAATGAAGACGGTGAAATACAATTAGATGTTCTTAAAGCAAAACT
TTCTGCTATTCTCGAGGATGCAGCATTGGCGGAACAATTGATTAGCAAATGTGCAATTC
CACGAGGAAGTGGTCCTGAAACGGCTTACCAGACCATGAAGTGTTACAAGGAAAGTA
ACCCCAAACGTGTCCTTGTTGTTTAATTTTTTACTTTTTTGTATTCCATATTGATATTGAC
TTTTTGTTTTCATTAAAGAAAGTTTAGATCATTTTTCGTTATTTAAATGACTGTAAAGGC
TCTGATATAGCTTTAAAAAAATTATGACAACTGAAGAATAACGTTTAAGGTGTACATTTT
TTAAACTATTGACATATTGGTGTGGGAAATACATTAAACCAAATAATAATATAAATAATAT
TTTTGTTTGGCTATACCTGATATTAAAATAACTGTATCATGAGCTAATTCCCCGATTTCTA
GCTCATTACTGGCATTCAGGTACTTGTTAGAAAACTGAAACTGAAGTGAACTTGGAAA
TGAAACCTAAGGTTGAACAA
>Cluster-12689.53600 MsigOBP19
GCCACGCTTCCAATTTCCTTTCTAATTCCTCTGGGCACATTTCACAAAGAACAATGTCA
TCTGCAAACAACATACTCCAAGGTAACAAAAGTAATAACAAAAATTAAAGACAGTTTT
TATCTTCTTCGCATAAGTGACAGGTGTTTATCTAGTTGAACCCAGAAAATAAAAGTAGG
GGTTAAACTGGAAGTGCATTTAGGTAATATAAAAGCCGCTGATGTGAACTATTTCATTAC
AGAAGTTTAGTTTCAGAGTAATCATGAAAGTATTATACATCTTCGCCCTCGTCTTATTTG
TGGCTTTGGCCACCGCTGCACCAACATCAGAAGAAGAATCAGAGGAAGAAGACATATT
TAAGAAAGCCCATGATGCCTGTCAAGTAGATCCAAAAACAAAATTAGACGAAGATGCA
GTTAAAGGCAAGAGCAAAGAAGAGATAATGAATAATCCACAAGTTCGTAAACATTCAT
TGTGCATGAATGTGAAAACTGGACTTCAACAAGAAAATGGAGAAGTTAACAAAGATA
AACTAAAAGCTCTTCTGGAAAAGCGCATAGATGATCAAGACAAAGTCAACAAAATTGT
CGAAGAATGCAGTGTTAAAGGAACTGGAACTCCGGAAGACCTTGCTATGGAATTCGAT
GAATGTATGATGAAGCATCGTCATAAACATCATCGTGGTCACGAACATCACAAACATGG
TGAAAAGAAAGATGATTAGATTATGATAATAAAGTAAACTCTGATTTTTTTATATATTATT

AACTTAAGGGACTTCCAACAATAAATAATAA

>Cluster-12689.31354　　MsigOBP20

AAAAGAATTTATTTGAACAGAAGGTATCTAACATTAGATATAATATATGTATAACACACA
AGTGTACAATCGCTTACTCTTTTATATCAGTAAGTTCTTTGAAGGTTGAAAGTACGCATC
TCCAATTGAAAACCGCGCTTTCGGTTGCATTTAGGCCGGGAGGTCTTGTTCCACATTTT
TCCATAATTGTTTCTACTACGGTTGCGTCGTCTATTCCTTTCTCTAAATCTGTTTTCATAG
CTTCTTTATCAATATCTCCATCTTGTGTTAAACTACCTATTTTGGTGAGTCGACACAAAA
TGTGAGGACCTTCTTGATCAGCCGTTTCGAGATCTTCCGTTTCTATCCAATCGTCGGCC
GCTCGTGTTTCTGGATCTTCTTGGCATTCGTTAATAACTTTTAACATTTCTTCTTCATAGG
AATCGCTAAAGTCAATCTTACAAAACGCTATCCCAAACACAGCACATACAAAAATTGA
CAAAAACATTCGT

>Cluster-12689.30900　　MsigOBP21

GGGTATTCCGATATTGGACACCAAGGCATCTCACTTTTAAGTTTCAGGAACGAATAACA
ACAATATGAAAACGTTGGTATTAGGCGTTATCCTCACAATACTTATCTGCTTGGCGAATT
GCTATCTGGAACGAAAGGATTTTTCGGATGTGTTGAATAGCAAAGTCGATGATTTGCAC
GCTAATTGTAAATGTTATTCAGGCGCCTCAGAAAAACTTATAAACCAAGTTCGACTTGG
ACAATTTCCGGAAAACAACAGTTGCATTAAGCGATACAATTATTGCATTTGGAAAGATA
GTGGAGTGCTTGATGAAAACAACACTATTATCGAAAAAGTGCTGATGTCCCACCTAAG
CGACTGTAACTACGACGATGCACATATTTATATTGAGTGTAATAACAAAGTGGACTGTG
GAGGGACATGTCCTATAAACCCGATGTGGGAAATGCAGAAATGTATTGCCGAGAAGGT
TAAACCCGAGAATTATATTTACTTCTAATACCGGATTTGGTTAAACGTATGAAGAAATAT
TAAATGTTTCACCAGTTTTGTTCGCATTTATATATTTAATTGGTGTACAATAAAATAATAA
AAATAAAA

>Cluster-12689.31861　　MsigCSP1

CCTTCCACTCATTCTCTGAAGGACATATTTTCAGGGAGAAAAATTCTGCACGCGACCTG
ATGTCCAGGTTCCAGGGTTTTTCGGCATTTATTTACTGAACTATTAACATTTTAAACAAA
CATTATAAAAAAAAGTTTAAAAATAATTTTGTTTTCAAGGAAATTACATTAATTTAGTAT
GTACGTATCATTACATTAATTTGTTTAAAAGTAAAATCTTTCTTAGTATTTTTTATAACAA
ACGTATGAATTCCACCTTTCAAACGTAGCGAAATCGGATAGATATAATTTAACAATAATA
AGTGACATTTTTAGTATTGATTATGCAGATAGGTTATGAAATACCGTCTTTGTATTTGACA
TTCATGACCCTGAACTATGTAAAATCATTCGAGATATTTTATTATATGGATATCAAGAATA
CCAACCAACTTAAATTAAAAAAAGATGACCTACAGAAATTCTACATTTTATTTACTTGTT
ACACCAAAAATAAAATATTAAATAGACAATACACATTGTTTTATATCTGCCTAAATTGAA
ATTCCTTCTTTCTGAAACTCTTCTCTGTGTTTATTCCTGTATGTTTTGTTAGCATCATATTT
TGCAGCTAGGTCGTCAAACATTTGTTTCTTATTTTTGGCAAGAAATTTGATTACTTTCAC
GGCTCCATCTTTTTGCTTCTCACTGCATTTTTCGCAATCATTTTCCAATGCGTCACCAAT
ATGAGCTTTAAGTTCTTTTCCATCGGGAGTACACTTTCCTTTCTCCATCACACAATTAAA

GTAATTACTTAACAATCTTTCACTAGTTAAAATTTGATCGAGGTCGATGTTATCATATTTG
GTTGTATATTTTTCGCAAAGAACAACGCCTATCGAAACGACGAGAATCAATAAAGTAAG
ATTATTCATGTTTGACAAATGTTTC

>Cluster-12689.30764 MsigCSP2

TGAAGAATCGTGACAAAATCACAAAAATAAAAATGAAATTGTCAATTGTGTTTTGTATA
GTACTCGTTATAGTTGCAGTTTCTGCAAAACCCGGTGAAGAGAAATACACTACAAAATA
CGATAATTTGAATATTGACGATATTATTAACAGTGACAGGTTACTCAGAAATTACATTGA
TTGCCTTATGGGCACCAAAAAGTGCACTGGCGAGGGAGAAGAACTGAAAAAGGTGTT
AAGTGATGCATTCGCAAGCAACTGCGAAAAATGTAGCGATGTTCAAAAAGCGGGAGC
CAAAAAAGTCCTTTTACATTTGTTGAAGAATAAAAAAGATTGGTTTAACGAAATCGAA
GGCAAATACGATGCAGGCCACCATTTCCTAACCACCAACGCCGATGAAATAAAAGCTG
CTGGAATTTCCGTTTAAATTAGCGAGAACGTTGAGCCGAACAAAATTGTTATTTAATGA
TTTAATGATTGTTAATTCGATGATTTGTTACACTTTTAGTTACCGAAGTAAAATAGCATTA
CACTAAAAAA

>Cluster-7844.0 MsigCSP3

GCCTTGTCCGATCGTATTGGCGATGGGATTGAATTCGTTCGGAACCGGAGGTAGTGTTG
GTATTGGCGGTAAGAAGATTCCGAGCGTGCTGCTGCTGCTCTCACCATTATATTTGACTA
CTGTTGACTTCGTCACCGACTCCGTTGCCTTGTTGTCGGTCAAGGTTTGCTGTGGTTTC
TTCACCGGAGGCTGCGAATCGTCGTCTTCGTCGCCGAACCGATTGAAGACGTCAATCG
GATTCGGCGGTTCATCGGTAATCGGTTTTTGAGATTCGACGATAATCGGCTTTTGCGGTT
CAGGCGGATTGTATTCTCCGTTGTTGTAGGTGTTGATGAATCTTTTTACGTAGACATCAT
CGGGATCCCATTCGGCTTGCAGTTGAGCCCACACTTTGGGGTACTCTTTCATTAAACGT
TTTACTGCTCGTAGAGTGGTCACCTTTTGTTTTTCGGTGCACTTCTTGCAGTTGGTTCTG
AGGGCGTCTGGAAGAATTCTTTTAAATTCAGCGCCGTCCGGAGGACAAGGTCCTTTGT
TGAGCATGCAGGCCATATAGTAATTGACCAATCTGCGATTATTGAGGATCATCTCGATGT
CCACATGATCGTACTTCGTTGTGTAGTACTTTTCCTTTTCTTCGGGTGCAGGAGCCGGC
ACTACTAACCCACATAGGCTAACGATGACCACGTAAAGTAACGGTACCATTCTTTGCGT
GCCGAACGGTAAGTAAATAGTACCTCGAAGGAATCGAATCGACGGTAGTAGTACCGCT
TGCTATATGCGTGAAATGTATGTTGCTTCGACGGAAG

>Cluster-12689.19536 MsigCSP4

ATGGAGCAGACACGGAATGTGTCATCTAACTGTCACCAAGATCAAGTTCATGTCCAAG
GTTAAATTGATAATCCGCTAGTGCCGTTTTATTTTTCTCATCCCTATTCTTTTCCTCCCCA
GTCGTAAGACTGTTGGTACATCCTATTCTCTAAATACTGTGGTGTTGAGCCTATGTAGCA
CGACTGATAGCTTTTACAGTCAATTTTAAACATTAAATGGGATTTTTTAAATTTTGTTGA
CTTATCTATTTGGTAAAAATACTTTCAACTTGGTGACACGGATCCAAAGTTAACTTGCA
ATAATTATTTGCCTTGCTCCAGCTATTCCTAATCCATGTACTTAATGAAGGCGCTGTGTG
GTATCGTTTTTATATATAATTTCATAACGATTGAGTTAAAAGAACATATTATTTAGTCACC

CAAATTTTACTATTACACAAGTTTGCAAATAATTTAAATAATGTAAAAACCTATTTATATC
TGTAAGTTTATAATACAAAAGCTAACTGTACAGGTACATCGTAATTAACTAAGTATATTA
GATAAAATATGAGTAATAAATGACAACATTTGTTTTCTTCAACAGCGTTTTGTTCTTTGG
AAAATTTGATATAATAAATATAATAGTAATAATCTCTTCTCGGTTTTAATAATAATTTATCC
CGAATAGTGCTTCACAAGCTTATTCCATTCTTTGGGATAATGAACTTGCATGTAGCTTAA
TACTTTTCTAACTTGTCGTTTTTCTTGAGGTGTACATTGATGACAGTTTCCTTGAAGAAC
CAAAGGTGCTAAACTTTTTAGTCGACGGCCTACTGAATCACAAGGACCTTCTCCAGTG
GCACACTTCAATTGCCTCAATAGGTATCGTTTGTCTTTCAGCGCGGCTTCTAAGGCATC
ATCTGAAATGCTCGATCGGTCTCTGGAAGCAGATGCCGCTACGACGACCAAAGTAAGT
AATACAACCACATAAACAACGTAACTCTCAAGTTTCTGCATCTTGATCGATCGGTAGTC
ACTAAGAAAGTGTAATAAGTGAAAGTAAAAAACCGCTGCAACCAATAAACTCAGGGT
CTCGATTAATTCGTTGAATGCTTGACTTGTGATACATACGAAAAAGTGGAACCGTA
>Cluster-12689.28620 MsigCSP5
AATTTTCGCTGCATTTTCAATACAAAATTGAGATTCGGATGCATGGTACAGCTTATCGAG
CTTCACCCACCAGTCGCTGTGGTTTTTCTGTAAGAAAGCGATCACTTTGAGGGCACCC
TCTTTTTCGTTTTGACTACATTCCGCACAATTGGTGTTGATGGCTTTGGGAAGAGTATCT
CTCAGGAAACCTGACTCGTTGTTACACACAGTTACTTCACCCAAACAACAATCAACGT
ATCTCTTAAGCAATCTTGTATTACTGAGGACTTCGTCAACTTTGATATGCTTGTATTTAGC
TGGTGTTAAGTCACCCATGGCGTAGCCGACTGCGATCAAAAACACTAAAAGCCCAACT
CTCTGCATACTGACTGTTGTAGTTCTTCTGGCTAATTGGCG
>Cluster-12689.31619 MsigCSP6
GGGCCTCAATATTGATTACTTTCGGCATCGCTTTGGTGATAGAAATACGAAGACTACTAT
ATGTATATATATCTAGCAGGAAGAATTGTTATTGTTTATATATTTTATATATAAATAGAAGC
AGTTTTAGTTTTTAATATTAATTATTATTAACCACATTTCGTTTTTGTAACTATTAATCTTA
AATTCCAAGTAGTCAGTTAGGATTAATTTAAAACATAGTAATGTTAATATTACATTGGAT
CAGTAATTGTAAATACAAGAGAAACAGTACATTTCTTCGACTTTCTGTCCGATATCTGTC
CTTAAGGCTTACCTAGGCTGAGAACACCACAGGTGTACCCTACTTCCACAAGCTTAAA
GTGAGGAACACTGTCAAATGCCTTCTTATAGTCGACAGAGGCAGTAGATGTTTCTCTTT
TGCTTCTGGGCCTGTAGAAGGCTGACAGAATCTATAATTAGTTGTTGCTTGCATGCTATA
AGACCATTTTTGCAATATTTCTGTTCCTCGATCAATATTTTATTTTGATCCATATGTTTATG
AATTTTTTTAGTAATACAAGATGTTAGAAACGTTTCTTTTGTTGGTAAACAAGTAATGGG
CCAATATTGTGAAAGGACTTTCGGTTTGATTGCTTTTTGTATTTTTTGGATATTGCAGGT
TGTGTGTAAGCTGAACCTCTAGATTTTTGGTTGATTTAATTATTCTTAATATTATTATCATT
ATTATAAGATCCCGTTTTATAAAGAGTTTCAAACATAATTAAATAACGAGTAATCAAAAC
GTAGCATTAATTAAAATGAACGTTTTAATAAGAATAAACACTTTTTGTTAAATACAATCT
CTTTTCCGATCATTGTGTAAGAATCCAATTACTGGCGCAATAAAAATTATAACAAAGAA
AATAAATTATGTGCATAAAATGTGCAATTATCTATCATGATCGAGTTTATTATAAAAATA

TAGCAATTGTCTCCTACTTAATGGTGATATTTGCATAAAGATATATATACACTCCCTTTTAT
TTAAAGTTTTCTTGTTGCCGCAAAATGAAGACATTTATCGTCGCTTTATTTCTTGCAATT
GTAGCCTTGGCTGCCGCTGAGAAATACACGACAAAATACGATAATGTGGATCTTGATAC
CATCATAAAGAGTGACCGTTTGTTGTTGAATTATGTAAACTGCCTTTTGGAGAAAGGAA
AATGTACGCCGGACGGATTGGAACTGAAAAAGGTGCTTCCGGACGCGTTATTGACAGA
TTGTTCAAAATGCAGTGAGACCCAGAAAAAAGGAAGCAAGAAAATCATCCGCCACCT
TATCGACAACAAACCCGAATGGTACAAGGAGTTGGAAGCCAAATATGATAAAGACGGA
ACATATAAAAAGAAATATGATGCAGAAATAAAAACGAAAAAATAAGATCGTTACTGTAA
AATTTTTCATTTTAATATACATTGTGATATTTATCTATGTTGTAATAATAACTGGTTTAAGA
GTGTCGCAAAATTATTTCTTTAATAAATACCGAAAGTACGTAGTAAAAAA
>Cluster-12689.31574 MsigOrco
GTCGACTTGAGTCCGAGCAGCGACTTGGACTTACTGCGTTTTGAGTTGGTTAATTATTG
GACTTGTAATCGGTGCGGTTATGCGCGTGTTTGAGATTGTGCCGCGAGTAAATTTTTGA
AAATTCATTTTGAATGGAACAGTGGATCCAGTGATTGTTGTTTTTATGAAATTGCGGGA
CCTCTAGTTATCCTTCAAGATGATGAAATTCAAGGTATCCGGCCTTGTGGCCGATCTGAT
GCCCAACATCAGACTTATTCAAGCTTCTGGGCATTTCATGTTCAACTATCACGCTGACA
ACTCCGGATCCCTGCACACCTTGAGAGTCGGATATTCTTGCATGCATTTAGTGTTCTGTT
TGCTGCAATACGGCTGCACTTTTGTTAATTTGTTAGTGGAAAGAGGAGATGTAAATTAT
TTAGCTGCCAACACGATCACCGTCCTTTTCTTCACCCATTGTATAACGAAGTTCTGCTAT
TTTGCCCTTAGATCGAAACTCTTTTACAGGACGTTAGGAATATGGAACCAACCGAACA
GTCATCCTCTGTTTGTGGAATCCAACAACAGGTACCACGCACTGGCTTTGAAGAAAAT
GAGGACTCTGATCATTTGCGTTTTTTCGACAACTGTCTTTTCGGCCGTAGCATGGACTT
CGATCACGTTCGTCGGCGAGAGCGTACACAACATCAAGGATCCCGAAAACGAGAATAT
GACGCTGGTCGAAGAGATCCCGCGACTCCTGGTGAAATCCTGGTACCCTTGGAACGCG
ATGTCAGGCGGCGCCTATTACATCACCCTCATTTTCCAAGTCTACTACGTCGCCTTCTCC
CTGATGCACGCGAATCTCCTGGACAGCCTGTTCTGTTCGTGGCTGATTTTCGCCTGCGA
GCAGCTGCAACATCTGAAGGAGATCATGAAACCGCTGATGGAACTCTCTGCATCTTTA
GATACGTACGTACCGAAGAGTGCAGATTTGTTTAGGGCTCCAAGTGCAACATCCCAGG
ACAATTTAATCGAAAACGATTATAACAATGCTAAAAACGAAGAGCTTAACCTGAAAGG
AGTGTACAACACGAGGCAAGAAATGGGAGCGAACTTCAGATCTGGCGCACTTCAATC
ATTTGGACCAGGAGGAGGAGGTGTCGGACCCAACGGCCTTACCAAGAAACAGGAACT
CATGGTTAGATCGGCTATTAAATATTGGGTTGAGCGACACAAGCACGTCGTAAGATTGG
TAACAGCCATCGGTGACGCATATGGTGTTGCTCTTCTTCTACACATGTTAACCTCAACC
GTTATGCTTACACTCTTGGCTTATCAGGCGACAAAGATTAACGGAGTTAATGTCTACGC
AGCCACCGTTATTGGATACCTTGTTTACGCCCTGGCTCAAGTATTTCATTTCTGCATATTC
GGCAACCGACTCATCGAGGAGAGTTCATCTGTTATGGAAGCAGCTTACAGTTGCCATT
GGTATGACGGTTCAGAAGAGGCGAAAACTTTCGTTCAAATTGTATGTCAGCAATGTCA

GAAGGCCATGTCGATCTCTGGAGCTAAGTTCTTCACCATATCTCTGGATCTGTTTGCTTC
TGTACTCGGCGCCGTGGTGACCTACTTCATGGTGTTGGTGCAACTCAAATAATTTTTCT
TTTTGGAAACAATCCGCGGGGGCGTTCTCGCTCCCCATCCTGTCTGTTTTTTGATTTGA
AGTACCTGATGGTGTTTACGCATGGTCCAAACTATAATAGTAATAATAAACTGAGGTGAT
AAACTAATTATACCGCATGGATACTGGCAAACTAGGTAATATACTGGTATTTTTCGGCTG
AAATCCGTTAAATGAATTAACATTGGCAACAACGCATCAACTAATTCGTTACTAACATCT
CCCACTCTGACTATAAGACTATAAGAAACATATATCATCCTGTAACGAATATTACTGAAA
ATGCAGAAATGCCTCGTATAGAACCCTTTCTATCTCCCTTTTTATTTCCTATTAAATTCTT
AATTGAATTCGTTCCTCTATCCCAAACTCTAATGTCTATACTAACATTTTCAGCATACATT
ATAAATTGTAATTAGTAATAATCTTCTTATCCGAATACCTAACAAGTTTTTGAACTACGC
ACATGAGGTAAACCCAGAACGAAATGCTCGTTCTGTACCGTTCAGCTACTCTGTATGTT
GAGTTTGAGAAGTATTTTCTATTATCCCACTAATGGGCCGAACTACAATTCCATAAGTTT
CGAAAATTCCCTCCTTACACATCAGCGGTTCTATTACGAATGAATTTTAGATGTGGATCA
ACATCTATTTGCTAAGAAACCAATCTTAAAAACAATTCATTTCATTGCAATATTTTTGTTA
TCGTCCTAGGGTGATTACATATTTCTCGTCGGGAGCATTGTCACAATACGTGATACAACG
TTTTGTTTTCGTTGCTGTACGTAGAATAAACTAGTTTTACCAGAAACAATTTGGCAGTT
GCATACCATTGAGGCTGGGAAATCGAGTGGTTGTCGGTGCAGTCAAAATTCGCTAATTT
TTTTCGCTAAACGATATTTGTTTGCCTACTTTGTCAGTATCCAAAGGGTAAACATAATAA
TAATACTCTTTTGTGATCGCTAACAGCAATATATTAGACAAGCAAATTTTCAAACTGCAA
TAGCCGCTGATTATGATTATAGACCTAACAATTTTATATATTATTTACAAATTAGTTTTCTA
GTGTTTCATATTAATCGCGATCATACTGGGTGGTCCTTCGGTAATTATACGTGGAACTATC
ATGAATGATTTACTTTAAAATATTACTATGAAAGTTAAAATTTTACAAATTATTTTTTGCA
ATAATATCAACGTTTTTGGATATTCTGACGTGGAAATTTATAATTATGGGTTTTTGGGCGT
AGGACGTAATATTTTGGTGGTGTCTTCTTTAGAAGGTCATGTGGTGATTTGTGGTGTAA
ATGTGTAAAATTTTCTTTCTTTCAAATTAGCTTTGGTTTTGTAAAAAATGTTAAAGATGC
GTCATTTGAAAAAAAATTGTATCTATCGTATTGACACGTTTTATTAAAGATGTAATATTTT
TAATTAAAAAATTTACTATACTTACTTCCTCGTACGATTATTCGAAGACCATCATTTTCAT
TAAATCACCGAAAAATGTTTTGTTTGACGCATTTGTGAAATTTATATATGTTCCAGAACA
TTCTGGTTGATTTGATAAAAATTACCAGTTTAGAAGTAAAAAATTTTAAATGACAAGGT
TTACGGTATCGTATTTATTTTCTTCAAAGAGTTTTGCTGGTTATTAGATGTTTTAATATATT
TATATATTCGAACCTTTTTGGTTTGAATATAAAGGCAATTGCAATAAAAATTAAATATAGA
CGTGGCAAGACGTAGAAGAGGACCTAGAGGAGAGGATCAATTAGAGCAATAAAATCG
ACTTGGGAGTTAGGAAATGAACAAAGAAGATTCAGTACAGACACAACAGGCGAAGAT
TGTAGATGCAAAATATTGGGAGGGGAGAATATAAGAGTAATAAAATCTGAAGATTAAGT
TGATCTGGATATATTAAAAGTATGGATAACCTTAGAGAGGTAAAGAAGATTACCGAATG
GAAAACAAATGTGAAAAGACGTAAAAGAAGGCCTAGAGGTAGGTAGAAGGACCATGT
AAAAAGTAAATTACCAGTGGAATTAAGAAATGGACAAAGAAGGTATCGAACAAGAAA

AACTGGAGAAATATTGTAGAAGCAAAATACTGGAAGCAATGAATATAGTAAGAGTAATA
AAATCGCAAACATCGAGATGGTCTGGACATGTGAAAAGGATGGATAACCTTAGATAGG
TAAAGAAGATTATCGAATGGAAAACGAATGTGAAAAGACGTAATAGAGGACCTAGAAG
TAAATAGGAGGACTATGTAAATAGCAAATATATTTACCATTGGAATTAGGGAATCGCCGA
AGGAGGTACAGAACAGGAAAAACTGGAGAAATATTGTAGAAGCAAAATACTGGAAGC
AAAGTATATAGTAAAAGTTATAAAATCCCAAACATCGAGATGTCTGTACACGTGAAAAG
GATGGGTAACTATAGAGAGGTAATAAGAAATACCGAAGAACGACGAATGTGACAAGAC
CTAGCAGACAACTTAGAGGTAAATGGGAGGACCAAGTAAAAAACAATATAATCATGAA
AGTTAAAGAATAGACGAAGAATATAAAAGGACGAGAAAAACAAAAGAAGGATGGTAG
AAGCAAAATAGTGGGAAGAGAGTAATAAAACCCCGAAGATTGAGTTGGTCTGGACAT
GCCAAAACTATCGATAACCTTATAGAAGTAAAGAAGATTGCCGAATGGAAGACGCATG
TGACAAGCTATAGAAGAGGACCTAGAGGTAGATGAGAGGATCAATTAGAGAACAATAA
AATCGACATAAGATTTTAGAAATGGACAAAGAAGATTTAGTTATGACAACAGGGGAAA
GATTATAGAAGCAAAATATTTGAGGGGTGGGGGAGAATATAGTCATAGTAATAAAATCT
CGAAGATTAAGTTAGTCTGGATAGGTCAAAAGGATGGATAGCCTTAGAGAGGTAAAGA
AAATTACCGAATGGAAAGAGGATGTGAAAAGAAATACCTCAGAACCTAGAGGTAGAG
AGGAAGACCATTGGAATTAAGGAATGGACAAAGAAGATATAGAACAGGAAAAACTGG
AGAAATATTGTAGAAGCAAAATACTGGAAGCAAAGAATATAGTGAGAGAAATAGAATC
TCAAACATCGAGATGGCCTAGACATGTGAAAACGATTGATAATCATAGACAGGTATTAA
GGATTACAGAAAATCGACGAATGTGACAAGACCTAGCAGACAACCAAGAGGTAAAAA
ACAGAAGAAATAAGGTAGAAGCAAAATATTGGGAGGAGAGGAGAAATAAAATCCCGA
AGATTGAGTTAGTCTCGACATGCCAAAAGGATGGATTACCTTATAGAGGTAAAGAAGAT
TACTGAATGGGAGACGAATGTGGCAAGACATAGAAGATAACCTACAGGTAGGTAAAAG
GATCAATTAGAGAGCAATAAAATCGACATGGGAGTTAAGAAGAAGAAGATTCAGTACA
GACAACATGGGGGAAAGAAGCAAAATATTTGGAGAGGATTGAGTTAGTTTGGACATAC
TAAAAGTATGGTTAACCTTAGAGAGGTAAAGAAGATTACCGAATGGATGATGAATGTG
GCAGGGCCTAAAAGAGGACCTTGAGATAGGACGAACTAGAGGTAGAGAGGAGGACC
ATTGGAATTAAGGAATGGACAAAGA
>Cluster-12689.10464 MsigOR1
GCTACTTACTACAGACGAAGTAGCTGTTAAGTGGTATTCAGCAATATGGAAATATTAAC
TTTGAATATAAAAAGTTTGCAAATTTTGCGTATAATACGAAAACCCGGCGAAATGTGGG
TTAAAGGTATTTTTATTAATTTTTGTCAAATTTTCGCTACGGCATTTATCGTGTATCTAGT
GATCCTTGCAGGTGTGCATCAGATGACGGTGAACTTCGAAGATTCTATGATTTTAGCCG
AGACAATTATGCCTGTTTTAAGTGGTGGTGTTTACCTTTTTATCTTCTTCATTTTTAAGAT
TACAGCCAAAAGTATCGAGAAGACAATCGAAAAGTTTTCAACCTTTTTAAGATTTTGTG
ATAAGGAGGTTATTGTTGAAGCAGAAAAGGAAGTCCAGTTTTTTTCAAAAGTGTTCTT
CATATATTTTTGCGTGGGGAGTTCAATCTACGGAGCTGTTCCTATTTTAGACTTAAAATC

TTGCTATGAAAGTAGATTATCTGACGAATCCAGAAAACATGATATGTGTGGAATACCTTT
CAGAATTTGGGCTCCTGCTGATGTTACAAAACAAGCAAATTTTTATTTTTACATATTACT
GGTTACTTTCGTTTGGGTTGTATGCGTATCAGGTGTATTATGTATTACTTTACTCTTAGTC
GGATTCTTGATTCATATTAACGTACAATTAAAAAATTTGCAAAAACATATTCTTCGAATG
TTTGATGATGACGAACAAATCGGTTGTGACAAACGGGAAGAAAACATTCACTTCTGCA
TCAAATACCACATTGCAATTATTGAATACTCCAAAGAAGTATTTAAACATTTCGAAATGT
CGCTTATAATTCACGTGTCTTTACCTTCGTTCATTATGGGAGTAGTTTGTTATCAAATGGT
TTTCGAAGAAAGCTTATTGGAAAAATTACGGTATTTTTTACATTTAGTGGGATGGGTAG
CCGTCCTGTTTATGGTGTGCTACTACGGACAAATAATACTAGACCAGAGCAAATATGTC
GGAGACGTCATTTATGATTCTAAATGGTATAATGGTCCCCTTCATTTGAAAAAATCTGTT
TTATTGATGCTCCTTCGTTCTCAAAGACCATTGAAAGTGACAGCTGCGTCATTCAGTGT
ATTATCTTTGGAGACATTCCTTAAGATCATCAAAACGGCGTATTCGTTTTTTACTTTACTT
CTAACTATGTCTACATAAATTTTTTGAGCCAGCTATGTGTGCTTTTTTATTAACTTAATAT
TTGCAGAATTTCTCAAAGAAGCAACTTTATGTCACTCCATTTCTATAGTATATACAATGT
TTCAAAGCGATAGTAAAATATCTCACCAG

>Cluster-12689.10756   MsigOR2

GGACGTACTGAAAGAAATGCTAAACGATTTTAATCAACCGATGTTTCAGCCGAAATGC
GAAGAACATCTAAAAGTCGCTAAAGTTACGGTAACAATACAAAAAACTTTGTTTTACG
TTTGTTTAGTGTTAAGTATCCTTGTTGATTGCGCTTTTATGGCAGTGCCTATGATAGTAAA
TGAAAGAGTGATGGCTGTACAGGGATGGTTCCCTTTCGATTGGAGACTGTCACCCAAT
TATGAAATGGTTTATATCTTTCACTGCACTGTAGCACTGTGGGTCACTATGACGTGTATG
AACTTGGATATATTTACGTGTGGTTTGCTAATGCACATCGGATTACAATGCGACTTTATAT
GCATCACTTTAAGTAGTTTAGATATCTTTTGTGTTAAAAATGGCGTTTTGCAACTAAACG
ATGGCATAAAATTGACACCAAACACCAAACAATTCTCCAATACTATGGTGGAAAATCTA
GTGGTTTGTATTAAACACCACAGACATTTAAAAAGGTTACTAGCTCATGTTGAGGACAT
CTACAAAGTTACCCTTTTTATTAATTTTTTATTAGGAGGAGTAATACTTTGTTTCGGCTTA
TTTAGGTTATCCACCGTAGAAGGTGGAGCTGTCGAGAGCATGGTGATGATTTTATTCAT
GATATGCATGATAATCGAGCAGTTTATATTCTGTTGGTTCGGAAATAGACTAACCGACAA
GAGTGCTAATATATTTTTTTCTGCTTATAATACGCCGTGGACAACTTGCAATTCAAATTTC
AAGAGGATTCTTTTACTATTTATGGCCGTAACGCAAGCTCCAATGGAACTAAAAGTTGG
AGGAATCCTGGTTGTATCAAATCCTGTCTTTGTATCGGTTGTGAAATCTTCGTATTCGTA
TTTTACTTTGTTCAAAAATCTTCAACAATAATTTACATCCACACTTGAAGCAGGATTAGT
ATAAATAAAAAATTAATTAAATCACCTACACTTAAAACACCAAATCTTTACTGACACGT
CACCTAATCTACGATTAATCACGTACAATTTATTTTATAAATTATCTTCTTTTATCGCTAAA
AACAAACAACAATGAACTATCCGTTTTCTTGTTCACAACTATAAATACAAGATATACGTA
GAAGTTTGTTTTAATTGAAAGAAGGTAATAAAAAAGAGGTAGCAACACACTTGATATT
ATTATAGCAAATAAATAATTCAGCGTTTTAAAATCAAACCAGTTGCATTTGGCGTTAAGG

ATTGGAGGTTTGAGGAATACCTATATGTTTGTTAAATCTTTTATTTTCAGCAATTTTTTGT
GTACTATTAATAGTATATTATACAACGATCATGTAATGATGGTTATTAATACACAAGCATT
AACCGGGATACGAAACGAGCCG

>Cluster-12689.14368 MsigOR3

TTAGAAGCCGATATATTTTTGTAAAATGACTGACTCTTATGAAGTGGATTTAACTGACAT
AGTGAGCCGAAATATAAAAATTTATATTTTTTTGGAGTTATCCCTCAAGAAAAGGAATC
CAATATGGCACTTTTAATATATGTTGTAAGGGTATCTATATTAACAGGTTTACTTTATGGA
GGTTTAATATTGGCTCAAGTAACTCAATGGATTCTAAGTTTCACAGGAGATATTGAAGA
AATTGTGACAGCTACGTATTTAACCACAACTAATCTGTTTTCATTCATAAAAGTTTGCTT
CGTGTTTAGGCACAGGAAACGTCTACTAAATTTAATAAGCACCCTCAACATAAAAGAAT
TTAGACCTAGAAATGTTCATCAATCTAAAGTTCTACTTGAAAATATAACAATGGCAAAA
ATGGTGACGAACGGTTTATTAGGCATCAGTTACTTGACATGCACTTTTTGGGCTATATAT
CCGTTTACGATTCAGTACGGTCCATGTATGCCTTTAGTTGCCTACATCCCTTATAGCATCG
ATAAATTATCGGTTTTTGCCATAACCTACGTTGGAGAAGTAATTGGCATTGTTGTAAGTG
CAAATTGCTGTCTTGGTATTGATTCTTTAATAACAGGTTTAATTATAGTTATCTCTGCACA
ACTTACTATATTAAATGATTCATTAGTTAATCTTCGCAAATATTCAGAAATGGAAATCCAT
AAAGCCACCGATAATAATAAAGGCACTCGTTCAAATTTATTGGATAAAGTGATGGTTAG
AAACATAATAAGATGCGTTGAACAACACCGGCGTATTTTACAGTTTTCAAAAGATTTTC
AAGATATTTTCACCGTAGCAACTTTTGGACAATTTACCGTAAGCGTATTGATCCTATGTA
CAACTTTGTTCAAATTATCACTGATAACGAACTTGGATTTAGACTTTTTTTCGACTGTAC
TTTATCAAATGTGTATGTTAATGGAAATATTTGTACTGTGTTATTTTGGAAATGAGGTGAT
CGTTAAACATCACTTATTGACTGAATCTGCTTACCAGTCTGACTGGCTGGACACCAGTC
CAACGTTTAAAAAGAATTTGATATTTTTTATGACAGTATCACAAAGAAACTTCCGGCTT
CTTGCAGGTGGTTATGTTACATTATCCATGGATACATTTGCGAAGATTCTGAAGTCTTCG
ATGTCCTACTACACAGTCTTGAACCAAATAAATGACGAACAATGAAAAATTCTTCAAAC
TTTATGGAAATCAAAATCAGTCAAATATTAATAATTTAATTGAGCACATTTTATTCAATTA
TACATGTCTGTATCAATAAATATTAGCAGCATTACAA

>Cluster-12689.16833 MsigOR4

GGTAAATCCGGTTTGTCGGGGAATTCGGTTATCAATTAGTTAAAAGCTTCGGAAATTCG
GATTCAAACGTTACAATTTTCTTAACGGAGAAAATGGAGAAAGCGGCTGACGAATCTC
GCTTCGTGGCTAAATGGAGGGAAACCAATGGAGGATATGGGATGAATTTTAAAAGAAT
TGTCGGGGCAAACGTTGCCGCTTTAGAGTTATGCGCTTTCATGGTGCCAGATTTTAATG
GAATAATGGATAGGATGTGGTATGGAGTAAGATTTTTACTTTATGTAGTGGGCACATATG
GTAGTCAAATAACTAGCGAAACAATAAATTTGTACTACTCAACAGGAGCCATTTCAGAA
ATAGTATCGGCCAGTTTTCTTTTTCTGACGCACGCCGTGCAAATTATAAAAGTCGTGTAC
TTATACACGTACATGGATAGAGTGAAAAACCTTATTAAATCGATAAATCGCCCAGAGTTT
CAACCTCAGTCTGAATATCAACGAGACACCTTAAACTTTTATATCAAAGTATCGAAGAT

GATCACCTACAGTTTCTGGGGAGCTTGCGTGGCAACTTGTGTGTTTTGGGCATCTTACC
CATTTACAGAAGACGAACTTGGCCTGCCATTAGCCGGATGGTTCCCATTTAATACAACA
AAAAGCCCAAATTTTGAATATGCTTTTACTTATCAATTCATCGCTGCCACTTTGAATGGA
CTCTCGAATATCAGCATTGATACAATAATGTCAGGTCTGATAATGGTGATATGCGCCCAA
CTGCACATCCTAAACGATTCCCTGATAAATATCCGCCATTTTGCGGAATCGGAACTGGA
GCACGAGTTCAGAGACGATAATGGTCGAGATGAAATATCGCCGCGATTACAGGATACA
ATGAACAGAAAATTAGTTGAATGTATTGTTCATCACAGATGCATTCTCGAGTACACAAA
GGAATTCCAAACTTTGTTCTCCAACAGCATTCTGGGTCAGTTCATTGTTAGCGTCATTAT
AATATGCATCACCATGTTCGAAATGACGCTGACACCCGTTGGTAGCCTTCAATTTTTTTC
AATGGTACTCTATCAATACTGCATGCTTCTGGAAATATTTTGTGGTGCTACTTTGGAAA
TGAAGTGATTATAAAGAGCAACGAGTTGACAAAATCTGCTTACCTGTGTTCATGGATAT
TTTGTTCTGAAGAATTTAGAAAAAATCTAAGATTCTTTATGACCAGAACCCAGTTTGAA
ACCAACATTTATGCAGGAAGATTCTTCACTCTGTCCTTAGGTACCTTTGTGACGATAGTA
AAATCATCTTGGTCGTACTTTGCAGTACTCATGAATATTAATAAATAACGGACCTAACCA
ATAACTACAGTTTAATTATGATGCAATTAAAATGCGATAAAGTTAGCAAATAGCGTGTAT
TAGAATATTTAATGAAAATAAAGTTGGTAGTTAAGCA

>Cluster-12689.17431 MsigOR5

TTAACAGCAAAGTCTATAAAATATATAAAATTAATTCTAGCTAAGTTCCGTCATGGAAGA
CATGGGTATTCTTAGTTTAAATATTAAGAGTTTAAAATATCTGTTTCTTTGGCCTAGACCT
AATGACAAATGGTTAAACCGATTTGGTAGTTTCACATTATTTGTATTGATTGTGATGATG
AGCGCATCACAAACTATTGCTGCAGTTGTCCACCAGTTTTTCGTTAATTTCGAAGACAA
CAAAGTAGTGTTCGAAGGTATTATTGCTTTAGCTGATTTTTTTGGATACGGTTTCATGTA
TGCAAATTTTAAATTGAATTGCCACAAAATTAAATCAACGATCGACAAAATTACTGTGT
TTTTAAAATTTTGCCCCGAAGAAATTTTGTATGAAGCTGAAGAAAAAGTTCGATTAATT
TCAAAAGCGTTTTTATTATATATTTGTGGGGGAGTAGCCGTTAATTCCGCTCTACCAATG
TTGGCATTTCAAAGTTGCGAGGAAACACGTCTTAATGAATATTACAAGATCCACGATCC
TTGCGGAATGCCAGTTAGAACTTGGTATCCGTTTAATGCGAAACAACCATTGCACTACT
ACTTTTTGGTAACACTGCATGCATACACTTGCTTAGCGATTGCGTCAACCGTTTTAAGTA
TTACTATGACACTCGTAGGATTACTTATTCATATTATTACACAAATAAAGCATTTACGATA
CCTTATGCTCAACGTTTTTGACCATGATGTCGAGGATCGTGTTAAATGCGAAAGAAAGT
TACATTTTTGTATAAATTATCACGTAACTATTATCAACTATGCCACTGAAGTATTTCAGGA
TTTTAATTTGCTACTCATAGTCCACGTCTCACTAACGTCCATTGTTATGGGCAGCTTGTG
TTTTCAAATTGTTTATGCGGAGAATGCCACGGACAAATTGAGATTCTTGTTGCATTTGG
GTGGTTGGGTTACTATGCTTTTTATTACTTGTTATTATGGTCAGCAAATAATTAATGAGAG
TTTAAGTGTGGCCGATGTTGTTTTCGATTCAAAATGGTACAATGCACATCTTGAAGCAA
AACGCTGTATAATATTGATGATGATGCGTTCACAAAAACCGTTAAAGTTAACAGCTGCA
TCTATAAGTGTTCTCTCACTCCAAACATTTCTATCGATCACCAAAACGGCATACTCATAC

TTTACATTGTTATTAAGTATGGAGCAATAACCACGTAACTAATCCTAAAATCTCGGACCT
GAGAATGAACAGGGCAATGTTTTGTTTTATGACACGATAAATGTTAAATTTAGTGGTCA
CATTAGATATTGGTTATGTAGTGTTAGTAGTTCACCAGAACAGTTCAGTGAGTGGAGTAT
TTCGAAAGTAAAAAATGGATATTGACCCTTTTAATATTTAAATTCAGATTTATTCTTAATT
ATAACTGCTGCTCTAAAATGAATATGTTCATAAATGTAGATTAATATAGAATTTAAAGCA

>Cluster-12689.18574 MsigOR6

GTCAACATGGACAAGATATTGCCTCCGACCAAGTTTATGAAACTTATGATGTACGTTTG
TGCATTGACAGGTATTTGGCCGTTGACGTGCGAGAATAAGCCACGACTTGTGCGATTGT
ATCATTATTTCTTCATATTGACTTATAGCAATGTATGTATCGCAACGGTGTCCATGCTATC
ACAACTGTATATTATCCTTGACAGAGAGAATTTTTTATCAGACGAAGCCGTTGGCATCAT
GTCGTTTGTCCTTGTGTGGACCGCTATTCTAATTAAAGTAGTTCAAATCAAGCAACCTC
AGACTAAAGATCTCATTAGGAAGATACTGCACTTCGAAAACAGTGTTAACGCCAGCGC
CGACGAAAAGCTTATCGCCATTTTTAATCAGTACAGTTCAAAAAATAATCTACTGTGCA
GTGTGTATATTACAGGAGTGTTTTTTAATTTTATCGTTGTGACGCTCGTGCCGATATTGC
AGCCAAAGATTATGGAGACAACAACAAGAACGTTCCCTTTAAACTGCTGGTTTCCGTT
TGATCAACAAAAGTTCTACGCTGAAGTTTATGTTATATTAACATTTTACGTGGTGCTTAT
ATCTATTTACTGTCTTGGTGCCGATATGTTCAGTTTCAGCATGATATTATTTCCTATAGCA
CACTTGAAAATAATTCAGCACATTCTCAGCGATTTCGACAATTATGTAGAAAAGACGGC
GAAGCAATTAAATTGCAGTCCCTCCGAAGCGCGCTTTATCACAATTCGAGAATGTATTA
TTTTACATCAAGACGTTATTAAGTACATGAATGAATATAATAAACTTTTTGGGGGAGCTT
CTTTATTTGATGCAATACAGAGTACAATACAACTATCTTCGCTAATTTTGGCAGTCGTGT
TGGGTGAAATAACGTTACCACAACTGATTAAAGCGGTTTTTCTGATTTGTAACACCTTA
TTTCGATTGTTTATTTTTTACTGGTACGCTGACGATATTCCTATTGAGAGCGTTAAAATCA
CAAAAGTTCTTATGGAGAGTAACTGGCATGAACAACCAACAAGAATTCAAAAAATGAT
GTTATTTATCATGATGAGATCCAACCGTCAGCTTGCTATGAATTTGGGATCGTTTACTAC
AATTTCATTAAAGGTTTTTTTCGCGATCATTAGAGGGAGCTACTCGTACTTTACTTTAAT
TTACCAGACCAGCTAAGTTAAATGTATAGAATATACTAAGCAATATAAGCTTAAAAGTAA
TTTTTTTAAATTTCACATGGAAATAAATAAAATTTAAACTTCATCGATTTCAGTTACGAA
GCATCTATGAATGAAACCAAATTAAACAGTAAAAGCATTTAGAAAAAGTTATTTACAAC
ACGTCGAGTGCAATAACCACTTTTTACATGAGTTGCATACAATATTTTTTATACGTTCAT
GCAAAAAACCACTAAGTGTAGGAAAATAAAAAGCATAATTTGACAGCAAAATATTTGA
TAATTAAGTAGCACTATCGACTATCGAAAAAGTTAGGAACTAATAGGAACAATTTAACT
CAAACTTTGTTAATGTTAAAAGTAAATGTACA

>Cluster-12689.18959 MsigOR7

GTTACATTTGAACTTAAAAGAAATCATCGATATTAGTTGGAAATGTTTGTATTTTTTTGG
GGTCATGTATCCCAAATTTGAAAATTTGAAGATAACTGTTAAATACATCGTAAGAGTTTC
TTTCTTTCTTGGAATTTTCAATGGCGGTTTACTACTAACTGGACTCGCTCAATTCTTCTT

AGTAATTGGAAATTTAGAAGAAATGATGAAAGTTACATTTCTTACTTTTACAAATATAAT
CGCATTCGGAAAATTTTATGTAATTTGCAAACACCAACCAGATTTATTATGTTTAGCCGA
GAAGATGAACAGAAAAGAATTTCAACCGAAATCCGAAAAACAAATTCAAGTGCTTAA
AAATTACATTAGATTCTCGAAAATGATTTCTTTCTGTTTATATGTTGTATGCGCAATGACT
GTAGGTTTTTGGAGTGTTTATCCATATACTCAAGAAAATGGACCGTTCTTACCCACGGC
AGGGTATATACCTTTTGATACTAATAATCCTATAGTCTTTGGATTGATCTATGCTTATGAG
GTCGCAGCTTTAGTCGTTAGTGGATATGTGGATATGAGTGCTGATTTTTTTATTGCAAGT
TTAATTATGGTAATTGTTGCACAACTTAAAATACTCAATGACTCCTTGGCCAACATAAGC
GAGATAGCTGAAGCAGAAATAAAACAAGAGCGATACACAAGTTTGGAATGTTTTCAGA
ACAAACAGAAAATAATAAACCGAATACTTATCGAATGTGTTGAACACCATAAAGCCGTT
ATTGAGTTCGCCGAAGAAGTTACTAGATTGTTTGCCGTAATAATCTTTGTTCAATTTATA
GTAAGCGTCTTCGTTATGTGCGCAACTTTCTTCGAAATTATTCTAGTACCAGTAATGAGT
ATGCGATTCTTTTCAATGGCGTGGTATCAATTATGCCTGCTTTTGGAAATATTTCCAATCT
GTTATTTTGGCAATGAAGTGCTCATTGAGAGTGATAAACTGACAAATTCAGCTTACCAC
AGCGACTGGATTAATTATTCCGTGGAGATAAGGAAAAATTTATTATTTTTTATGACCAGA
AGTCAACGAAACTTAAAATTAACCGTTGGAGGATTTTTTACTTTATCTCTGGATACATTC
ATAATGATACTAAAGTCATCTTGGTCATATGTAGCAGTTCTAATTCAAATTCAGAATAAG
CAATAAGTCACTATCAAAGAAGACCTATTTATAGCATTCGCACATAATAAGTTGTTTATT
ATCACAAGAACATAAATAAATTGCACTTATA
>Cluster-12689.19570 MsigOR8
GGACAACATGGAAAACGACGCATACGCTAAAGATTTCTTTATTGTGAACCGATGGATAC
TCAGGTTCGCTGGTTTGTGGAGACCGGAAAGTCAAAATGAGATTATTCAGTCTTTATAT
ACCCTTTATGTAGTTGGTATTTTTCTTTTTGTGAATTTGTTTTTCACTTTTACGGAGTTTT
TAAGCATTCTATATGTGTACGAGAATGAATATGATTTGATAAAAAACATAAGTTTTGCAT
TGACACATTTTATGGGAGCTGTGAAGGTAGTTTTCTTTTACTTTCAAGGGCACAACCTT
AAAAGAATAATGACCACTCTAGAAAGTTCCGAGCTTCATTACGAGGATTGCGAAATGA
AAAAATTTTATCCCGCAGTAACATCGAAGTTATACAAGAAAACTGGTATCAAATACACA
ATTATATTCTTCATGATGGCTCATGCTACTTTAACATCTTCGTACCTACCTCCGTTTCTGG
CGGCTTTGAAATCTGAAGTGAATAATACGGAGAGGATGTTACCGGATAGACTACCATAT
TATAGTTGGATGCCTTTCCGGTTTGATACCGCTGGTACTTATCTAATAGCTCTCGGATATC
AGGCGATACCTATGTTCTCCTATGCATACAGCATTGTTGGTATGGATACACTATTTATGAA
TATAATGAACTGCGTTGGAATGAATTTGGAAATAATACAAGGAGCCTTTGTGTCAATTT
ATCCTAGAGCTGTCGAAAAAACTGATGGGCCGTTACTTACTCCAGATGAGCTGCATAAT
ACAGAGACGTTAACCGTCATTTTGAGAGCGGAAATGAAAAAAATAAGCAAACATTTAC
AAATTGTTTACAAGGTATGCGACGACTTAGAAGATATTCATAAATATTTAACACTCGCTC
AAGCTACTGCCACATTATTTATTCTGTGTTCTTGTTTGTACTTAGTGTCAACGACACCCA
TTGGAAGTAAACAATTTCTAGCAGAAATAGTATATATGATAGCTATGGGTTTTCAACTGA

CATTATACTGCTGGTTTGGAAATGAAGTAACACTGAAGGCGGACAAGATGCCGTTTTAT
ATTTGGCATTGTGACTGGTTAACTGCTGATAACGATTTTAAAAAATCTATGATTCTTAGC
ATGGCAAGAGCAAACCGACCACTTTACCTTACCGCTGGAAAATTTGCACCACTGACTT
TACCTACTTATGTAGCGATTTTAAAAGCGTCGTATTCTTTCTTTGCAGTTATTAAAAATAC
CAGTGACTAATTAACACCGCAGACGTTAAAAAGATGAGCACAAGAGACATTGCAAAA
ATGTAATTATATAATAA
>Cluster-12689.19693 MsigOR9
TTGTAACTTAATTGTAAAGAAATAATATAATATGAATTTGTATAAAATATATATATAATTAT
AATGAGGTGTAATTAACAATTTAAGTGTTTATAAAAGTCATGCATTATTCCTATAGAAG
TTTTATAAATTGTTATATGATAAGATCGATGTATCGATGTATCGAAACATCATCAAAAAAA
TATGTTTCTGTTATGATCTAAAGTTACTTTATTCACCGTTGACGATTTTGGCATAATGATA
ACTTATTATACAAATTATCGTTTACTTCTAAACAACGTCTACACAAGAAAATTGAGGAA
CAGAGGCGAATATACCTATTTCGATCCAGTTATGTAAAATCGACAAATATTAAAAGCTGA
TTTAATAATGCGTGTGCATTCCTGATATTGAACTTACTTAAGTTGTGTTTCAAAAGTGCT
TTAAAAAAATCAAATACTTTTCAAAAGACTTTTTTTTAATCTTTAGCTATCATTTGGGC
CACCAACTTGTGGTAATTTAGTCGTGATGATAATAAATTAGTCATAGTTTAAGTTAGGAC
TACAATGTTAAAACTTTTTAAAACCTACAATATAGACTGATCTCTAAGTTTAGGTTTTCC
TTATTAATATTTTCACTAAAATTAATAAATTCAGTGTTTATCATTTAAAATGTTGTACAATG
TTTTATTTTCTATAGCTATAAATTCATGGGTATCCACCGCTTCTATTACTTACCCTTCATAT
TGGATAAAAAGGTATAAAGGGAAAAAGTAAATTTTAAAACTTTGAGATAGGTATCCATA
TTAACATTATATAGACCATAAGCACTTAACTGTGGAAAATTTTGAGAACGTTGTAACATA
AACATTATATCCTTAACCAATTCTTCATCTCCAATGGTGTGCCAGTTGGTTTGAAAAATA
AATATCGGTAAAATAGTTGCATGATGTAGCAATTCGTTTCCTATGGCACAGTACACTAAA
AGTTGATTTAGCAAAACCGCTAACGTTACCAGATAAAATACTACTTTGAAGGAAGTTAG
GTTCTCCGCATGTGTCAGCATGAATAGAAGAAGGCATACATCTGTTATTGTATATGAAAA
CTCAATCATTTCAATTGAACTAAACATTTTATTCATTTTAGTGGTTATCCTGAGTAACATT
AAATGATGTTTTACGCATCTAACAAAAAATTCCTTGTGAACAGTTTCATACTTCCTTCCA
AATGCCTCGCCAGTCTCGGACCTTAGTTTTTCATTTATGGTGCACATGACCGGAGTATTA
AAAACTAAAAGACTGTGCTGCAAAACTTTGTACTGCGTTATCACGCAGGAACAGATCG
CAGTAAACAAATAATCAGTTCCTATTACAATAGAGTAAAATAGGCAAGAGTCCGTTATG
AATTGGTGAAGATATAATAGTTGAAAATATGGTGTGGTTTTTATATTGATTTTCGGATAAT
CAACGGAGAAAGGCAGTTCTCCATAAATGAACGGAGAAGACATTATCAAAACACTAA
ACGTCAAAGATAAAATCAACATAATTACTAAAATATGTGTTATAATACTGTAAAATGTCT
TTATTTCGTTTTCTGAGTCATTGCTTACAAGATCGTAAGGCCAAAAATTATACAGTATAT
CATCCAAAATTGTTTTTAGTTCATTCTTGTTAACAAAAAGAGCAACCAGTTTTGCCAAA
GCCTGACTCATTGAGATGATGAAAATTATTTGTGTACAAAATGATTCAATGTCATCGGAT
ACATATATACATCGAAAAATAAGACCAATGATAAGGAATGCAAACAAAAGTGCTGATAT

TGCAAAAAGAATATGTCTTTGATAATAATTTGTTTTCTGTCGAGGCCAGAAGTAAATAG
CTCGTAACAATAGCTTTGGTAACCATAGAAATAGTTTACAATAGTCTTTTGTATTCATAA
CACCAAAGGCAGAACTTTCCAATATCATAGTGATGCTTTTCG

>Cluster-12689.20868 MsigOR10

ATCGCGTCAAGTTTTCTAGTTGGTCTATTCCTGATGGAAACAGTAATTGACTACCGATCC
TTCTTTTCGTTCCATGTTTTCGTCTTCAAAATATTTGGATTCTGGAAACCGGACAATACA
ATGAAGCACAAGGTACTTTATAACTTCTACACTGCATTTTGCACAATAGTATGGATATTG
TTTCTACTTTCTCAATTCATTTACATGTACAAAAATCTCGATGACGTCGAAGAACTGAC
AGCGGTCTCGTACTTAGCAGGTCCGTTTATAGTCGATCTGATTAAAATGTTGGTTATATA
CAGAAAAATGGACACGATAAAAGTATTGATGGTGAATTTGAATCAGCCGATGTTTCAG
CCCAAATGCGAAGAACATTTAAAAGTGGCGAACGCATTGAAAAAATTCCATAGAAACT
TTTTTATTTGTGTTTGTATTTGGGTGTACAAACCTACTTATTCTTCTTGATAATGCCTTTT
CTAAGGGACGAAAAGCTGCCGCCGACGCAAGGGTGGTTCCCCTTCAATTGGAGACCG
TCACCCAACTTCGAAATAATTTATATTTTTCAAAACTCCGTTGTACTGTGGAATACAATA
ATTTGTTTGAATTTGGATACATTCTCGTCTGGGTTGTTGATGCAGATCGGCTTGCAGTGC
GACTATTTATGCATCACTTTGAACAACTTGGATAAGTTTTGCGTTAAAGATGGCGTTTTG
GTGTTAAACGGTGAAGTTCCATTAAACAATTCCACATTTCCCGACACTATGATGGAAAA
TTTAGTTGTATGTATTCGACACCATAAGTATATTAAAAGACTAGCAGCTAATATTGAAGA
CATTCACAAGACCAGTATTTTCATTCTTTTTTTGGGAGGAGGAATAATAATTTGTTCTGG
CTTATGGCAACTATCCACTGCGAAAATTGGAAGTATTGAAAGTTTTATGCTAGTTTCATA
TACCATTTGTATGCTGACCGAACAATTTATGTATTGTTGGTTTGGAAATGAAGTAATCCA
AAAGAGTGGTCATATATTTAATTGCGCTTACAATACGCCGTGGAGAAATTGTGATTTGA
AATTTAAAAAGATCCTTCTGCAATTCATGACGCTAACTAAAATTCCAATAAGGATCAAA
GTCGGAGGAATGCTGGTGATATCAAATGCTGTGTTTGTCTCGGTTGTGAAATCTTCGTA
TTCCATATTTACCTTACTACAAAAGATTCAAGAATAAGTTTCATCCATTGTCTTAAAGGA
AAAAGATCACAATAAAGCTGTTTGCGCAATATGCTTATAATACAATTAAAAAATGTACTT
ATAGTTACGCATATCCAATAGATTTTAG

>Cluster-12689.21641 MsigOR11

GTCGAATCTTATAATGGAAGTTAATAAATTTTTGTTTTTTAAAATATTATTGTTAATACTAC
CATAGTTTAGCGATCCTAAGTTTAAAAAAGTTTTTCAATTACCTTATTTTTATCATTGATC
GCTTTGTGAAAAGATTTATTTATTATTGTGAATAAAACTAAGAGCTAATACACAGTAGAA
ACTATTTTTAACACTAAAACAATCTAAAAAAATCTAAAATCTAAAAAAATCTTTGTGAT
CCTATAAATAGTTGTTTATTTTGGTTTTCATGCTTCTTGTAGTTCCTCCATAATATAGTAAT
TGCTTGGATACAAAACCATTTCTGTAGTTTACCTTAAATTTACCGCAGCGGTAGCTAGA
GAATATATTAATCGTATGCCACCTGAAATTGTTTTAAAATTTAGAGCATATAAATTATAAG
CTGTTATTTCCACATCTTGAAGGCAATTGGACATAATAATGATTAATGCTTTTCTGTTTTT
GCTGTTCCAGTCTGTCCATTTACAATTGTATAATACGTTGAGTAATCGTGAAGAGCTGTC

TGTTATGGCTTGACCGTTGAAACAAACATCTCCGTAACCAATAATTAGCGTGAAAATTG
CAAAATACATCCTGAAGTTGTGAGGTGGATCTATACCCAAAACTATAAAATAAATTGCT
CCGACAGCCATAATTATGCAAGCTACAGAAGTAATGAGCAAGGTGAAACTTTGAACCC
TCAAAACTATTTGGAAACCACTCTTTATTTTTATGTGATATTTTACAATGTTGCATATATTT
TCGTAAACCATCTCTTGATAGATGTCATCATTAGAACATCTTGGATGTTGTTTAATTTCGT
TGAAGCTATTTTCAATATATTTAAGTATCCAAGCAAAATGCAGATTAACGTGGAACGTAA
AATATACTAAATAAGAAAATGGAAAAACTATTGCCAAACCACCTAGAACAGTAAAGAG
ACAGTAAATAAAAATGCTAACGTATCGCATGTGTTGTTCCATGTAGTAGTTTATTAACCA
GTATCCAGAATTATAGTCTATGTCGTCAAACCAGATTGGTAAGAATGATATGTGTGCAAC
GATTATTGTCACGAGAATAAACATTGTTTAAAAATGGTTTGTTTTGCAATTATGGAGAT
TTTTTCTTCGAAGATTTGATCTTGCATTCTCAGTTGACGTGAAATAATTTCCATTTCCTCT
AAACCTTCAATTACTGAATCTCGGTAATATAATGAAGCTATATAAAAACAAGAACATGC
CGGTTGATATAAGCACAACAATGTCACATGTGGTATAACAGCCCTAATCCCCCTATGATA
GATGATCAGAATTATACTAGTTGCCAAAAATAATTCGAGAGTCACAAATATCGCTTGGC
CCAAAAAACGGATAATCCACATTTTACTTTTATCCACTCCATACGTCATTAGTTTCAGTA
AAATATTGACTTTATCTTCTCTGTTTATTTGCGATTCCATTGTCACTGCTTCAAATACAGT
TTAATATGAGCCACTAATTCAGATAATTTCTGAAACGAAATAAAACTTTAAAGACACGT
TCGTTCACTTTATTGGTCGTTAGTAAAATGAATTAACTTATGTTCACTGAATGTGTGAGT
AAAACTTATTAACGACTATTTATTCTAGGAAAAATTAGCCGTTGCAACGAGTGG

>Cluster-12689.22548 MsigOR12

CAGCAGATTATTTAAAAATTGTTTACAAGATGGAGGTCTCCAAGGGCGCTCATCTGAAG
GTGGTCATCAATTTCGCAATAGCAAGTGGAATTTGGCCGATTGTCTACACCAAGAATAG
ATTTTACAAGATATGTTACTATGCGTATTCTTGGTTTCTATTTATGATTGCAATTGTGTTTA
CCATGCAACACTTAATCAATTTCTTTGTTTGTATAACAATAGATATGGACTTAAAAAGAT
TAACCAATATTACGACTTCATTTACCGTGTATACCATAGGTTTGTTGAGACTCAAGTATAT
TTGGACAAAGAACTCTATCAAAATGATAATGGAAATTATTGCAAGGGAAAAAGAAATA
CAAACCTCTGAAGATAACATGATTAAAAATATTTATAAAGAAAATGTCAGAAAAACCCA
TCGTTTGAATTATTGGTACCTTTACGTGTTATGCATTGATTCTGTCGTTTATTTAACTATAT
GTCAGTTTTTAACACCGAAAGAAACTTATGTTAATCCTGTTACAAATGTAACTTACTTTC
AAAGGCCTCTCCCACTTTATCCATGGTTACCATTCGATGAGCACGATTTCCATACAGTG
GCTTTTCTTTGGGCAGAGGTGGATGCACGTCGTGTTTGATAATTTGCTACGGAACAGA
TGCTATAGTGCACAGTTATATGGCGTATATATTAGGACAATTTCATATTTTTAAGTACATTT
TGAATAACTTTGATGCATACAAAAACAACATTAAGGGACAGTTGAAATGTGACGAAAA
TAAAGCCGATTTTGTAACAATGCAGCTGTGTGTTATCGATCATCAAAGATTATTGAGGTT
TTTAGATACGTTTAATGATACAATGAGAATTGTGATGCTTTACGATTTTCTTCAAAGTTC
AATGCAGTTGGCAATGTTGAGTATTTTCTTGATGCATGAAACAAAGCTGATGGTTTTTG
TTGTATCTGTCGGGTATATTTTGACAATGATTGGTAGACTTTTTATTTGTTATTGGTATGC

CAGCGAAATAAGCGCCGAGAACTACGCACTTGTAGAAGATCTATTCGCAGTAAAGTGG
TATGAAAAACCGCAGAACGTAAAGAAAATGCTGGCAATCCTAATCACGAGATGTACGA
AACAAATAGGTTTACAAATTGGAGGATTAGCAACAATGAATTGGAGTATTTTCATTGGG
ATAGTAAAAGGTGCTTATTCGTTTATTACATTTATGCTCCGCTGAAGGAAACCATGCTTT
AAACGTTCAGATGCTTTCACTTAGTAATTTAGTAAATGCTGTTTCTTAGGTAATAAAGTA
CACCTACGCAAAAA

>Cluster-12689.22720 MsigOR13

AAATTTTAATCTTTTTTATTTTACATTACGTCTAATACAAATGTGTGTGTTGGTTTAAAAA
TAAAATATGATTTTAATACATCATAAGTTTTAAGGCATGTGATCCTTACATTTAATTAACT
CTTCCTAATCGCTTGCTGCTCTTACACTTCAGAGAAAAACGTGTAGAGGGAAAACGAA
AACTTGACTACCCTTATATAAGTGTCGATATTTATATTGTACATGTTATAAGCGGTTAACT
GTGGAATATCTTGAGAACGCTGAATCATAAACATAAAATCTTTTTTAAATTGATTATCCT
CCAAATTGTGCCAATTTACTTGGAAAATAAACTCCGGTAGGAGACTTGCCTGGTAAAA
CATCTCATTTCCGATTGCGCAATGTAAAAAAAGTTGATTTAAAAAGAAAACTAAAAAA
GTTAATAGGAATGCTCTTTGAGATATAGTTGGATCTTTCAAAGATGTCAAGAAAAAGAG
AATAACGGATATATACATGATGACACATGTTATTTCAACCATTTCTATTAAACTGAACATG
ACATTCATTTTATCGGTAATCCCCAATAACATTTGATGGTGCCTTACACACCTAACAAAA
AATTCTTTACGCACGTTATTGTACTTTTCCCCCAGTTCATCGTTCTCGACCTGTCTAAGT
GTTTCATTAACTTGATCCATTTCCGGCGTATTAAACACTAAAAGGCTGTGATGCAAAAG
TTTGTATTGACTAATGACACTCGAGCAAATGGCCATAAACAGCAAATCGCACCCGATTC
CCACAAAATAAAACAGAAGAAACGATATGAGTTGTACGATATACACCACTAGATAAAA
AGGTTGGACGTTGTAAGCGATATACGGGTAGACCACTGGCACCGGAAGTCGGCCTGAT
AACAGGGACGGTATCAAAACTGAAGCGTCATAGACGAAAGCCAGCGTTAAGAGAATA
ACCATAATTGTTGTTATTATATTGTACCATCTTCTTAGTTCAGTTTTTAAACCGGTGTTTA
ATAAATCGTACGGCCAAAATTTAACAAATATGTCATCCAAAATTCCATTTAATTCCGAGG
TATTGATATAGAGGACAATGCATTTGGTCACCGTTTGAAACAATGATATGAGATTAATTA
ATTGGTAACAAATAGCTTCTGTATTATCTAATACGAGTCGACAATGTAAAACCAATCCTA
AACCATAAATACAGCTAACGATTAGTTGACCGATTAGAGAAAGAGTTTTTTATAGTAAT
TTTGCGTTGGCCAGAAAATAAAAATTTGTAACAACGATTGCGGTATCCACAAGAATAAT
TTGCAATAGTCAGTCGTTCCCAGAACACGAAGCCCAATATCTTTAAACATTTTGTTGTT
TTACCGTTAGTTACTCGATTTTTCTTACTAAAAGTGTTCGAGACAAACTAATTTCATTAT
TTTGTAGGGTATTAG

>Cluster-12689.23347 MsigOR14

TCTATGTATAATTTTTTGCCAAATGATTTATTGCTTTTGTGTAATCCAACATGTACTACCC
AATTAACAAACAACAACCCTTTTATTCTACACTTTTGTCACTCAGTTTATTAGGGATATA
TCCTTTATCAGCTAAACATAGAAATTTTACTATTTCGCGTATAACTACGGCCGGAAACGT
GATTTTTGCTTTATTAATATGTAATATTGGAGCTATAGGTCACCTTATAGTTTCGTTAAAA

GGAAATAAAGGAGCTGAGATCAGCGAAGATATGGCAGTAGCTTTTGGAGGTTTGGGAT
TCCTTATGTGTGCAGTGCTTTTCAAAATGCGCTGGACAAGATGGAGCAAGTTTTGGATA
GAATTAACAAATTTTAAGGAGTTTGGAATACCTGAAAATTTAAGCGAATTAACAACTAG
ATGTAATATTATGTCGATGATTTACAGTGTTTATATATCTGGAGGAATGTGTGCTTACGCT
TTTATGTCCATATTGGAAATGGATTGCGATGACTCACAAGAAAATAATAATTTGTGCGGA
ACTTTAACACAAATATGGTTTCCAGTTCGCCATGTATCAAATTCGATAATTAAAGTTATT
TTTTTCTTTCAATTATTGGTATGCATATGGGCGTGTGTTGCAGCAGGAAATTTATTTTTG
TCTCTTTCGAATCTTGTGAGTTTATTGTTTGCCATACACATCATCTTAAAAAGAAGTTAC
TTGAACTTTTTGAGGTGGATAATGAAATTACAAGAAAAATAAATCTAGAAGTTTGCATC
AGATATCATAATTTTATAATCAAGATGGGAAATCAATTAAATTATTTAACTAAAACCACTC
TTGGTCATATGTCATTAACTGCAGCTGTTGTTATGGGTATGATATCAAATCAAATTGTAC
AGAAGTACAAACCACTAGGGGCTGGAATTTATCTTGGAGGTTACGTCATTGCTATATTT
TGGTTGTCGCATGCTGGCCAGAGAATTACAGATGAGAGCTTTGCTGTAGCTGATGCTGT
TTTTGAGGCACCTTGGTACAAGGCAAGCAGTGAAATGAGAAAAGATCTTGCTTTAATA
ATACAAAGATCACAGGAACCACTTATTTTAAATGCTTTACCTCTTGGAACTTTTAATTAC
GCCTTGTTTATAACGATGCTTAAAGCTGCATATTCTTATTTGACTTTGCTACAACAATCT
GTTTCGACTGAAAATGATGGACAAGTGATAAAGTAATAATAAAAATCTTACAAACTACT
TGATACTTTTATAATGTTATCGACGACACTGCTATCATGTAATCAAATGTACCTACATAGA
ATGCACCTCTTTAACCAATTAGGATTTTATTTTGGTGGATACAGATTAAAACACGTGAAC
CAAGGAGATCAAAAGAAACTTACATTTTGTTTTAAAATAACTTTTGTTTGTGTTGAATA
ATAAAGTTTAGTCTGCAAATCATAGAGCTACGATTAAAAAAAACAATTAATTTCATGTC
CGAATGAAATTCTTACCTTACGTTTTAGATTTTGTTTATTCCAGTTAAAGTACTTAAACA
AAATATTATTGCAAGAAACAAACTTCACA
>Cluster-12689.23944 MsigOR15
TTGATTGATCTTTTAAATCGTACTCAAAATCGTTTCTTAATTTAATTAAGCATGTAGTAAC
GGAACGTTTATGAATACCAATTTGAGTGTAGGAACAGATTTGAGATGAAATTGAGGGTT
ACTTAAGATTTAGGACTGTTTATGAATTCCACCGTTAGTCGAGAACGAATACCTTAATTG
CCTAAATTGCCAAATAATTAGATCCACTTGTCTCAACTCCCAGACTTTCTTGGAATTTTC
TATATAGAAGTGGGAATGTCGAAGTAACTGCTAGACTAGATGGTCACTAAAAGCTTGTT
ACTATTTGTATAAAAGCGACCACGTTTATCTTAGTTACAGTAGTTGTTTATATTTAAATTC
TATCATCGGAAGAATCTATAACGACTAAGTTACTTATTTTAGATATAATAAGAGTAATGTA
AGATGTGCACAATTCACAATAATATTAATTTTCTTTTTGTTTTTTATTAGAATGCAAAGTT
TTACATTTGTAGCGTCTAGTTGTTATATTACCTATAAATATCCATTAGCTTACAATTTACTT
TCATTTTCTATTGACAATTAATCAAGTTTAAAAGGTAAACCAACAAATTTTTTTCGTAAA
ATTAGCATACGCTTACTTTTCATTTTTTTCCTTCACGTGAGACAGAAACATGTAAATGGA
AAATCCATACTGAACCACTTGTATATAGAAATCCATATTGATATGGTACAAATTGTAGCA
AGATAGTTGAGGAATTTTTTCAGCACGATGTAACATAAACATAAAATCTTTCGTAGGTA

CTTGATTCTCGATCTCATGCCAATTGGTTTGAAATACATAGTCGGGTAATAGACTTGCCT
GATGGTATAATTCGTTGCCAACTGCACAATATAGAAAAGTTCATTAAGGCAAAAAAAT
ATTATCGATCCAATATATAATATATCCATTATATTTGAGTTTTCCTCAGCTGTCGAAATGAA
AAGTCCAAAACATACTCCTGTGACGGTGAAAGCTATCTGCACTAATTCCACGAAATTGT
ATATATCGTTTACGTATTGTGTGATCCTTAGCAATAACTGGTGATGTTTAACACACCTGA
CAAAAAACTCTTTGTGGATGCTCTTATATTTTTCTTGTAGATTCTCTGGGTCAATCATCC
TTAATTTATCATTTATGGTCAACATTTCTGGCGTATTAAATGTTAATAGAGTATTTTGTAA
GAGACTATACTGAGATATCATAGAGGAACAGATAGCCAATATGAATATATCAGTGCCCA
AAGCCATGCAAGCACCGAACGGATAAATTGTTGCTATTTGTCCAAAATAAATAATTTCA
TAAAACGGAGTAACGTTTGTTTTAATTAGTGGGAAATAAACTGCGACTGGAAGTCCTC
CATGTATTAAGGGTATCCAAAAACTGAAGCTTGTAGTAAGAAGTATGGATAAGACAAG
AGACAACATGAACCATAATATGTTGGTGTAAAATTTTTTAATTTCCTCTTTTACAGTTGC
ATTCAATAAATCATATGGCCAGAATTTAGTAAGAATTTCATCCAACATGTTCTTTAACGT
TTTTCTATTGAAGAAGAAAACAGCGAATTTAATTATAAGCGTGGTCAATGCTACAACAT
ACATCAGATCCAAAAAAGAATAAATATTTTGATAGACAAATATACTTTGCAAAATTATAC
CCATACTAAAGATAATGAACAATATTTCTGCCGCAATGAAGAAAATAAGTTGCTTATAG
AAATTGTTTTTTTTCTTTGGCCAGTAATTGATCACTTCAAATAGTAATTTTGGGATCCAG
AAAAACAATTTGCAATAATCCTTCGTTTCCATTACACGAAAAGCGATCGCAT
>Cluster-12689.25514 MsigOR16
AACTACTTCCCTACTCAAATTTGAAACTTTATAAGGTAATTTAGTTAATTCCCAAAACCA
ATTTTCTACATAAACATTCGTCGTCGCCTTCTTTTAAAAAAACAAGTTATATTACTAGCT
TTGTTCCAAATAGATAAAAAATGGGTTTCATTACATATACGAGAATCTTGTTTATTATAAGT
GGTATTTGGCTACCAGATATATCTCATCCTGTGTTATTTCGGTGTATTCAATATTACGCCA
CGTTTGCACGAACTCATTTTTTACTTGTTTGCATAATATTTTTTACTGGTCTCATTTTTGG
AGACGAACAAGTACTGGAAGATAATTTGCAATATTTTCCACCGTTCGTTGTCATGGTAA
TCAAGATGTTTTATTTAAGAAGAAAGGAGCTCTTGGATTTAATAGCATTGTCTCGAAAT
ATTGAAAAAAAATTATTAAACGATGGAAATCCAGAAATTTCGCAAATTGTTCGTGAAG
AGAACAAATATAGCAACAATTTATTCATTGCTTTGATGTGTCTTTTTTTTCTTACACTTTG
CCAATTGCTACTAGTAGTACTGTATCTTATGACTTCTTTCAGAACAGTGGTTAACACAGC
AATCTGGTATCCTTTTGATTATCGGAAGCATGTCTATTTTACTACTTTTCATCAATTGTATT
ATTTCTCATATGTTATTATTTTATATGCAGCGTATGATACGTTTTTAGGATCGTTGATTATTT
TTGCTACGACAAGAATAAAAGTGTTGTCATATAAATTCAAAAATCTTAGCAAGCTGAGT
AAGAGAACTAATATACCAGTAGAACGAGTTATTGATACTCTAATTCAAGAACATAACGA
ACTAATAAGGTATGTTGACAACATTAATAAATGTTTAAAGTGGTGTTTCTTCGTTGATTT
CACAATGAAATCATATAATTTCTCTCAATTCCTTTTTTCTTTAATAAACAGCCTAGAAAAT
TCATACGTTGAAATGATGTATTCAATCTTCAAACTTATTTCTGTAATAGTAGAGAATTGG
TATATAAATTACCATGGAAACGATTTAATATTGGCCAGCAAAGACCTGGCATCAAGCATA

TTTTCAAGCAGTTGGTATGAATTTGATATTAAATCGCAAAAATGTTGTCAAATGATAATG
TTACGAGCGCAACGACCACTGGAAATACAAATTGGAAATATGTACCATCTTAGCAACG
ATTTATTTATTGCTATACTCAAAGGAGGCTACACCTTTATATTATTTTATAATGTTTAGTTA
TAGTAATGTTATATTGCAACCAAAACCAAAATAAATTGCACTATGCAAATAGCAGACGA
CACTAATTGTTATTTCTGGGTAGTTTCTATCAAATGGATAATATAGAGTACAAAATGTGT
AAATGTTTGGTCATATTT
>Cluster-12689.25974 MsigOR17
GCTACTTGTAGAGTGTTGTCGTAGCATTTTGATAGATATAGAGAATATCACATTACTTAG
TAACAGCCAAACTGCAAATAATTGAATAAAGCAAGTTTAAACGAATAAGTACGCTGGA
GTGCATCTTATATCTTAAAAATGAAAAACGATAATAACAACAACACTTGGAGTTATCCA
GAAGAATTTTTTCGAACAAACGAAATTATTACTAAGATAACAGGAATGTACATTCCCAC
AAAGGATGATACCGTCTGGATGAAAGTATTTTATACTATGTATCTAATATGTTTTTATACA
ACTGGAATAATTTTCATTGTGTGCGAAGTTTTGATCTTCAACGAGACCATAACGAATTT
TAATAAACTAATAAGTCATATCGGCATGCTTTTCACGCACCTGTGCGGAATTTTGAAGTG
CATAATTTTGATTTTTGGACGTAAAAAGTTGCAACGGATGATGAATACATTACAAGATG
TTAATTATTATTACAGCCCACTGGACAATTTTTCTCCCGGTTTAATGTTATCTAAGGAAA
AGAAAGCATCCTCTTTGATTTCAGTTGTAGTTTTTATTATGTATAGTTTCGTTGGAATATC
AGCTCATATTTCTTCAGAAATGATTTTAAATGAAGAAATTAAAAGTGAAGCGTTTAAGG
GAACAAACAAGACGTGCCAAGATTTTATGCCCTATTTTTTTTACGTTCCATTTGATACAG
AAATGAAATCGCAATGTAAACGAGCTTTCCTGATCATGGATGTAGGACTGGTAATTTTT
GCTTGGGTAATTTCCTGTTACGATGGAATCTTTGTGTCGCTACTAAATTGTTTAAAATCA
CAATTGCAAATAGTTTGTCACGTTTTCCGTTCTTTGAGAAGCAGAAGTTTAAAAAAATT
GCAATTAAAAATGAACTGCAACGACAACTATAACGAGAATTCCGCATTAGAAAATGAA
ATGTATAAGGAACTAACACATTCAACTGAACACTTGAAAATACTTTTGAGAGTTCGGG
ACGACGTTGAAGCTACATTTACTTTTGTGACTCTCACTCAAACTGTAGCATCTTTACTTA
TTTTTGCCTCATGCTTATACATTGCTTCTAATGTACCTATTAATTCACCAGAATTTTTTGC
TCAAATGGAGTACTTCATGTGTGTGCTGGTGCAATTGTCACTGATTTGTTGGTTCGGCA
ATGAAATAACAACAGCTAGTGAATTAATACCATTATCCTTATATGAAGGCGACTGGCTTA
GCAGTAGCCCTCGCTTTAAGACTTCGATGATGTTGACTATGACCAGAATGCAACGACCC
GTATATTTGTCTATAGGAAAATTCTCTCCCCTAACTTTAGCCACTTTGGTCGCTGTATGC
AGAGGATCTTTTTCATACCTTGCACTCTTCAAAAATGTTCAGTAATCAAGAACCTGGCA
AAACGTTCTAGCTTCAATGATACTACTGAATTAGTATATATAGTTTAATGAATCAACTGC
ACTTGTTTTAATAGGGAGAGACCTTTTCCATTTTCAGTATAATGACGGGTTAACGAATAA
CCTCATTCCATATCACTCGATTTGCTTATAATTTTATATTTATGGAAGAGGTTTGTGAAAT
GATAATGTTGGAAAATTACCGAATTTAAAATGGTTCTTGATCTTCTCCTTGTGTGTTTGC
AGCGTCCCAGAATCGTTAAGAAGGTGGTTCTAACATTGGATGTCATCTTCGCTTTTAAA
ACGTTGGAAATAATTTGATCTCGTTTCTATGTAAACAATATATAGCCTCGTATATTCGGAA

AACTTAACGTTAGTTTAATGTCAGACGCTGTGGATACTTAGGTATTACTCCTTGCTTAGG
AAGACTAGCAAAAAAAAATCCAACATATTATTGACTCGGATGACTTAAATAATAATAAT
GATTTAATTAATGTAGAATATAATTAATTTAAAAAAGCTTTTTGGAGAATGTTTTTAGGTT
TGCAGAGGATTTTTTTCATATGTTGCCCTATTTAATAGCATACAATAAAAATAAAACTGT
ACACATTTTTCACTTCATAGAAAAAATGCGAAGAAAGTTACGCTTAAGAAAATAGTTC
GAATATATGAAGAATTTAGATCAACGAGCCTAAAAATACACGACATGTAATGGAGTAGA
CGTAGATATATTATTTTTAATAATAAATTACACTGGATTTTATAAGTAGATGTAGAATAATG
TTAATAAATGAAGCATCAC
>Cluster-12689.26397 MsigOR18
AAACCATTCAAGAAGTGATTTATTTGACGACTAATTATTAATTTTACAATAACCGCTTGT
AAGTTTGTGAGTAGTGCAAAAATCTTGTTGTGTAAATAAATATTACCATTAATGTTTTTT
CTATTAGGCAGATCCATTTAAGAGTGTGAAGTAGGAATATGAAGTTTTTAAAAGCAACA
CAAACAAAGTGTAGTTTATTACACCGAGTGGTAGAGCATCCAAATGAAGTGGTTTTTG
ACATCTAAATAATATAAAAACAATATCTTTTTTAGTTTCGTTATCTAAAGTGTGCCAATCA
GAAGTATACACTGTATCATGAAGAGTTGACATTAACTGATTAACAGTTTCACCACTATG
GCAAATCATAAACGTTGCTACGTTCCATCCGATCAGAAACAAAATAGCTGCCGACCGTT
TTGTATCTCGTATTATCATAATTTCAACACTTGCAATTACAAAAGCGGCAATCAAAGTAA
CATAGGAAACTGTTTTCTTTGATAAATTGTTTAATTCATCGGTCATTCTTAATATATGATT
GTGATATTTTACCCAATAACTAAAACATTCACGTCTTGTTTGAAGATTCCTGTCGTTAAC
AATATTGGTAAAATGTTTGCCTAATTGCACAAAATGTCCTTGCAAAATTTCTGTTGCTTC
AAATGTGAGAAATACAAGCATGGCCACAGGGCATATATCATATTGTGTAAGCGTAAACT
GTATTACGAAAATTAAATACTGGATATATCCAGGAATTTTAATAGGATACCACATTGACA
AAAATGTGTCACATTGTAAATTTATGTTCCTTTCTTCCATAACTCGATGACAATGCGGTT
GTTCTCTATATGTGACGTAGGCATACACAAAGCCTCCTCCCACCAAATAACTTGCAAAA
CACTGTGCAAATAGATTATTTCTATAAATGATTTCATCCATAGTTTCTGGCTTTCCAAATT
TAGAATTATCAACAAGTTCTATTAGTAGTTTTTTCCAACGCTTATGCAAATATGTCGCCAT
TCCCAGAATAAACATTGTACCAAAACCCGTAGAAAGGGCAAGAATATCTTCACTAGTAT
CAATTTCGTCTATGTGGTCTAATTTAATTTTAATAATATGCGATATAACTCCGACAAGAAC
ACCAAAGGTGAGGATCCTCAATATGATAGTTTTCACGAAAAATGCTCTTAAATTTCCAT
TTGCTGTGTTGGGCAGAATTAAACACATTTGCAGAAATTTAATTGACACGTTAAATGGT
ATTTTGCTGACTGAGAACATTTTTTTT
>Cluster-12689.26484 MsigOR19
GTTTGCGATCCGTCATCGGACGTAATATAAATGTTGAGTTGTCGTAAAACATGGCTAAAT
CCGTCGAAAACACTGAAAAACATCTGTATGAAAAAGAGTTCCGCAACGTTTTGAGATT
GTGGAGATTTTGCGGAATGCACCCATTGAAGGGTTATTCTAAGCCTTTAATCGTATTTAA
CGCAGCGTTGACTTTTTATATTACGGTCCTTATAGCGCTTAAATTACTTTTAGGGCATGA
ATTGGTTACTGTCGAGTCTGCTGGAGTTTTCACACAGATATGGGTAAAATTCGTGATGC

TGACTACAAAAAAAGACAAAATCCTCCAACTATTCACTGACATAGAAGGCTTTTGGAA
GACGGACCCTCCGGGTAGCGAAAACGCGAACATGTTAAAGAGCCTGAGAAAAATGGA
ACGCGCCTTCTTAATCTACATCTCGATCAGCACTTGCATGTTTCTCTTCAAACCGCTTTT
AGTAAAAGGCACTACCATCTACTATTACTACCAAATTCCCCAGATCCCTTTCCTTATTTC
TTATGTGATAGAGTTTTACGTTACTTTAGTTACCATGTCGATGGTGATAGGGGTCAATTT
GTTTATCAGTATTACTATCAAAATTGGCGCTGGCCAGTTTAGCAACTTAAACGCTAAAAT
AAAGCAACTGGACTTAAGTAAGACCCAAAACGATGAAAGAGGTTATCAAGCTTGTAG
AAGGGAAATTAAGGAAAACATCGAATATCATGAACATTTGATTAGCTACGTTCGGCACC
TCGATGGAATATTTTCTTGGCTATTTACACTTTTAATTTCAATCATCACATCCCTCCTCTG
TATGAACATGTATGTGTTATCTCAACCGAATAATACAATTGTTGATATAATTCGATGCGGC
ACAATGGTACTTGCATTTACTTCAGAGTTTTTGTTGCTTTATGGTGTACCAGCCCAACA
GCTAATAGACGAGGCAGAAGAGGTTGCTAACTCAGTATTTTACCATTGCAAGTGGTACT
TACCAGGCATCGTTGAATTGAGAAAAGCTTTAAGTTTCATGATATTTAGAAGTCAGAAG
ATGGTATGTCTGTCTGCTCTCGGTTTTATTGATGTTAATAGACAAACAATCGTTGCGATG
ATAAAGACAGCCTACTCATTTTTCACATTTCTACAGACTGTGGAAACCTAAAATAATAAT
AAATGTTTGTTTCTGGTGACCGTTATTCATGGGTAAAAAATGTTGTGTCACAGTAGTTT
GAAAAACTGTAGTATTTTACTGCTATTATAATATCTATATTGTATATGTATGTTTTAGCTCC
TTAGCACTTAGCAAGAAAAAATAAAGAATAAAATTTGCATGTTGAAAATAAACACATTTT
TATTATAAGGAACATTCTATAATTGTTCAATGAACGGGGTCAATCAAAATAACACTATCA
>Cluster-12689.27122 MsigOR20
TTTTAATTGTGAAGGCTTTATTTATTTTTTGTGATATTTGTATCTAGGCGTCTAAATGAAG
CTGCTAACAAGAACGATCTATCTTCATTGTACTTTATTTTTATTTTGCATTTGTAGTAGAA
CTGCGACATATGACCAAGATGATTTCAATATCTTTATAAATGTATCCAAAGACAAAGTGA
AAAATCCTGCGGCGGTTAACTTTAAGTTACGTTGACTTCTGGTCATAAAGAATATTAAA
TTCTTTCGTACCGGCACCGAATAATTTATCCAGTCACTGTGATAAGCTGAATTTGTCAGT
CTATTACTCTCGATAAGCACTTCGTTTCCAAAATAACAGACAGGAAATATTTCTAAAAG
CATACAATACTGGTATAATGCCATAGAAAAGAATCTTACGCTGCCTATCGGCACCGAAG
TCATTTCGAAGAAAGTTGCACACAGGACAAACACGCTTACTACAAATTGACTAAATATT
ATTGTGGTAAAAAGTCTTGTTACTTCTTCAGCAAACTCAATAATAGCTTTGTAGTGCTTC
ACACATTCCATAAGTTTTCGGTTTATTGTTTTCTGTATATAATATTCGAAATTATAGTACTG
ACTTTGTTTTAACTCTGCTTCAGCCATTTCGCTTATATTAGCAAAGGAGTCATTCAGTAT
GTTAAGTTGTGCAGCAACTACCATAATTAAACTTGCAATAAGCAAATCAGCACTCAAAT
CCGTATATCCACCGATTACTGTAGCTATAATCTCATATGCATAGACCAACCCAAATACAAT
CTGATTACTAGTATCAATTGGTATATAAGCTGCTATCGGTAGAAATGGTCCATTTTCTTCG
GTATAGGGATAAATACACCAAAAGCCACAAGTCATTGTGCATCCTGCGTATAAACTGAA
AGAAATAATTTTCGACAGTCTAATGTAACTTTTCAGCGATTGAATTTGTTTTTCCGATTT
CGGTTGGAACTCCTTTCTGTTGATGTTCTCGGCTAAACTTAGTACATCCGGTTGGTGTTT

GCAAATTACGTAAAATTTTCCTATTGAAACCAGATTTGTTAGAGTAAGAAATAGAGCGT
TCATCAATTCGCTCAAGTTGCCGATACTTAGGTACAAATTCGCTATTTCAGCTAATACTA
TGCCACCAAAAAAAAATCCAAGAAAAAGAGAAACCCTAGCGATGTACTTTATGATCAC
TTTAAAATCATCAAACTTGGGATACATAATCCCAAAAAAATACAAAAATTTGGTACTGA
CACTAATGATCTCTTTAAAATCCAAATGAAACTTGTTCATCG
>Cluster-12689.27337 MsigOR21
TTTTTTTTGAATGCTTTCTTCTTCTTTAATACGACTAATTTATATCACATACAAAGCAGTT
TAAGTTTATACTAATATCTAATTATTCTGGTAACCCTTATGAGTGTGCGATTTTTTAGGGT
TGACCGACTTAATATCCAATTGCAACTTTTATAATATACTAAGAAACGTTTTCTTCAGTG
AAACATTTTTTTTTTATTTTTTCTTAATGCCTCGTGCAGTATTGGTGCATTTTTAAATTTTA
CTGAATACTTTTAAATAGGGCAAAATACGAAAAAGATCCTCTGCATACTGCAACAAACG
TTGCTAATTCGAGAACTGAAAATTTCCCAATTGTCAGATTGACTGGTCGTTGCATTCTT
ATCATAATCAAAATTAAAGATTTTTTAAATCTTCTACTGGAACCTTGCCAATTGGATTGA
TAAAGTGACTGCCGAATTTGTTCACCTGCAGCTGTAATTTCATTTCCAAACCAACATAT
GAGAAACAATTCGACTAGTATACAAAAGAAATATCCCATTTGAACAAAGGACTCCATC
GATCCAACTGGTAAGATAGATACTGTAAATACACATGAAGCATAAATGAATAGAGATGC
CAGTGTTTGTGTTAAAGTTAGAAATGAAAAAAGATTTTCAATATCATCTCGAACGTTAA
ACAGTATTTGTAAATGTTTAATACAGTGGCAAAGTTGCTTGTACATTTCATCTTCAAGAG
CAGGATTATCAGTGTCGTAGTGGATTTCATAATTTTTGGGTAAGTTTAAAGCTTTTAGAC
TTCTTTCCCTTATGGATCGAAAAGCATGACAGAGTATCAAAAATTGACATTTTAGGAAA
TTTAGTAGTGCCACAAAAACCGCATCATTGCAAGCAATAATCCAAGCAAAAACTTCCA
AACTAATGTCCATAAAACCAATAGCTAATTCACACTGCCACTTCTCATCCGTACTAAAA
GGAATGTAAAAATAGTATTGCATAAAATCTTTACAAGTTTTGTTTATCGCTTTGATGTCA
TTTTCCTTAAGTTCTTCATTCATTACTAACCGTGAAGAAATATGAGCAGAAATTCCGAC
ACAACTGTACATCATTATCAGCCAAAGTGAAATTTTTGAACTTTTAGTTTTTTCTTGAGA
TAGATATGAACCTGGAGAAAAGTCATCTGTTGAGTCGTACAAATATTCAGCATGCTGTA
GAGTGTCCATAATTTTCTTAATTTTATGTCTGCCGAAAACCAAAATGCAAAATTTGAATA
TACCAACTGCGTGAGTCAAAAACATTCCCAGGTGATTGAAAAATTTGTTTAAATCATTT
ATAGTTTCGTGAAGAAGCATAAACGTACAAATGAAAAAACAAATTCCGATAGTATAAA
ATGATATTACATATAACAGATAGGCGATCCTAAAAAATATGTTGTAATCCCTTTTCGGAAT
CCATATTCCGGCTATTTTGTAAAGAAATTTGTTAATGTCAAATAAATCGATCGGATAGCC
TAATGCCTGTCGAAAATTGGAACCTTGATTCATA
>Cluster-12689.27738 MsigOR22
TTTTTATTGCGTGTATTTTTAATAAATTTGCATAAATAAAAATGTAATTATTACTACATTGC
AAATGTGCATCGTGCTTCCTTAGAATGCGTTTTATTTCCATTTATGGATATGTTGCTTATT
TCTCTGATAATGTAGAAAGTAGAGTATATGTAGAAAACGTCAAATTTAAAACCTTTGTA
AAGGAATCCATGTTTAGATCATAAACACCATAAGCGCTGAGTTGAGGGGTTCTTTGCGA

GTGTAGTAGCACGAACATCACATCTTTTTGAAACGTATGGTCTAAGTTTCTCCAGTTGG
ATTTAAAAATACAATAAGGTAATAAACTTGCGTGATAATTTAGTTCAGTGCCAATAAAAC
AGTAGAGAAACAACAGCGAGAAAATACCAATACCCACAAAGATTCGATCAAAATTAAT
ACTGTCCTCCATAACGCAAGCAGTTAAAATCATTCCTAGGTTAATAATGCTGAACAAAA
GATGAACGATTATGTTTACTTGGAAGATGTCGTTTATTTTCTTTGTAAACCTTGTAAGTA
GTTTATGATGTTTACAACATTTTACAAAATATCCTTTTGTTTCATCGTATTCCGGATTTATA
TTGTTCTCCAACTGTTTTAACTTGTTATTGATTATTTCCATTTCTGGTGTATTCAAAGAAG
CAAAGCATTTCTGTAAAATTCGATATTGCACCGTTGCACAAGCTCCAAAACTTATCAAC
AAAAAGTCAATTACGAAGGAAAATGGAATCATATATACGAAAAATCCACATACTTGCGA
AAAATACACCAATTCGTACCATGGGGATGCTTGCCAATCAAAGGAATACTTTACCAGAA
AAGGTGTTTCTCTATCTTGTGATAATAGTGGTCGCAAGAAAAATAAAAGCATTGCACTG
TAAGGTATAATACATATGGAAAGCATAAACAGCATAACAAACAAATAAAAATGCTTTAG
TTCCTTTTCTAGTTCTCCTCTTATTAAATTATAAGGCCAAAACCTATTAAGTACCTCGTCT
AGAAGTTCTTTTACCTGTGTTACCTTGTAGCATAACACTACAAATTTAAAAACTGCCTG
TAAACCATATGCAATAAATAGTACATTAAATAAATGTTTACCAATACTGCCTCCAGCGAT
ATCAAGAGTTTGAATCAACCCTCCGCATACAAAAGTGATTATCAATATTCCAATAAGAC
AATTAATAGATGACCGTAAACGAAAATTTGCATTATAAGGCCACAACTGACAAATATAC
AATGTAACCATTGGAATCCATAGAAACAGTTTTGCATAATCATTAGTTCCGAGCAATTTA
CGAGCCAAATTGTCG

>Cluster-12689.27993 MsigOR23

ATCGCAATCGTTTACGGCTAATAAACAGTTGGGGTTTACTAGTGAATTCAACAAGATCG
TTCACGTTTTTTCTGTGTTAATGGTATTATTCAATTAATCCTAGCTTCACCATATTTGTGG
TTTTATTAATTCAGTGATAATTAAGTTCAATCCACTCCAATTATCAGATTCAAAATGGAC
AAACTAGACGGTTTCGGTTTTAAGGTAATGGGGACCAGAGATTATTGCAAGCTATTTCT
GTGGATTCCAAAACTAATTTTGTATGCTATTTGCTTATGGCCGGAAGCGGAAGCCACTT
ACTGTCCAAGATACATTTTTTTAGCTGCTGCAGAAATATTATTTTTGTTACTAAGCGCTG
GTATAGCCATGCAATGTATATACGTATCCCGAGAAATTAGTGACATTTTTATGGAACTGA
TTTGTGTTGTGGGGTTGGTATCGGCTATGATCAAATTTGTAGTACTTTTCGTAAATAACC
GAAAACTACAAATAATCATTGAAGACATTATGAATAAATTCTGGCCAAGCGATTTAGTT
GACGATGGTGAAGTAAAAAAAGAGATGAAAACATTTCACTCTACCATAACATGGATCA
TGGTATATGTGATAGTATCATTTTTATTCGGGGCCATTCTGGAATTTTTCGTCGTTCCATT
AATATACGGGGGGCTCCCTGTCCCTGTTTATGTGCCACTGATTAACTATAAAGTTGGCCC
GTTCTTTGCAATTGTTTATTTTGGAGAGATCATAACTGTTGGATTTTTTTGCGATTTCGTA
CTTATGGGTTTTGACTTATGTTTCATGGCTATCTGTTCCTGCCTTATATCCCAATTTAAAC
TTTTCTGCAACGTCATTACTACATTTAACACGCCAGAAATGGTTGAGATCAATACAAGA
CTGAGAGAGGTCGACGAAAAAAATAAAAAAAGTTTGAAAAATAAATATAAAACCGTA
CACGAGGAGTTTTTTGTCAGATGCGTTCAACATCACCAGCTATTATTAAGGACGATATA

CCACATAAATAATATGTGTGGTTTTGTGGAATTAGTGCAAATAACTTTCACCATAACAGG
AATTTGTTCAGGACTTTTCGTTTTAACAACGCTGGATAATCTACATTTTCTAAAAGTATT
TCTCACATCTTCATTCATCGTTTTTTCTCTAAACGAACTGTTTCTATATTGCGCAGTAGG
AAATGAACTTCACTATCAGGCGAGTCTGTTACCACACTTCATCTTCAAGGCCAATTGGC
ATCAGATGGAAGATACGAAATTTACCAAGAATTTTATGTTTATGCTACTTCGTTCACAAG
ATATTCCTAAATTAACCTCGTACAATTTGTATGACTTGAATTTGAACTTCTATGTGCAAG
TTCTTAGGTTTGCCTTTTCTATTTTCATGTTTTTAACAACTATACGAACGAAAACTCAAC
AGTGAAGCAGCCGATACCTTATAAGATTTGATTAATTGATAAACGATAAGAAATACAAA
AAGCAAACAAGGGTAGTCTAGATTAACATCCATACATTTTGCGTTCAAAAAATCTTCAA
AGCAACATTGCTACTTTTCCATGTCTACTTTGTAAACTGCAGACTTTTATAATGTTCTAA
TGCTTTATTTGATGATGAAAAAGTCTCACTAATTAAACCCTAAATTTTTGAAAACAAAA
AGATACACAAGGTATCAGATCGGGGGGATATGGTTTAAAATTGTAAAAATAAGTGGTTC
GTCGGTCTTTGTTAGTGAACCCAAAATCTTTATAATTCTCCTGGTCTATACATATCCTAAC
TCTTTTTTTTAGCATATAATGTTCTACGATCAAATATTTTTAACTATTTGTACTCAATTATC
CACACAATTTTTTTCCTTAAATACCTTTCTCATCGTTTAACCTTTTTCTTTTCCAGCTCTA
GCATTTTAGCCACGGTTTTTGACATATTATCAAAGGAATTATTTAGACTACACTTTAATA
AACGACGATCTCTTTGTCTGAAACGTTTTTCTCCTTTCAGCTGCTTCTGGCTACATATGG
TCCATTTTATAACTTAATATCCCTATAATCTGAAAAGATTTCATTGATTGATGGATGAATT
GGACATCATTCTTTTACTTCGTCGTCTTTTCTGTTTTCTGAAGGGATCAACTCCTCTGCC
ATCGTTTACTACTCCTGCTACTGCAAAACTGACATTTATAATGAGAACAAAATTTTTAAA
ATATTCTTATATGATGATTTGAGGAATTTGCGTCGATATGATATAAAACAGATATTGATAA
TGTAAATAATGGATGGACAAAAATTTTCGGTTCATGCAACAATGGAAATATATAAAATTT
TTCTTTTGGAGAACATTGGTCAAAACTGCAAACGATATCTACATAATTGATAATAATATA
TAATAATATATAAGGTACATAATTTCGAACTTCAATGCTTTTTTATTTATTGTCTTGCTTAG
TTGTGCTTTAATAATAGATACGAATAAAAATTAATACAAAGTATTTAATTTCATTCTCACC
TTCAAAATACTGACTCTTTTGTGTCTACTGACGTACTTACCTGCGCTATCTAATACAAGT
GATACAAGATAGATATATAAACATTTGGAATTGTTAGGTTAGGTATATTTTCTAACATTAA
AACTATAATTTGTTGAACTAGTTCCGCCGCAGATCTAAACATAAGAAAAACCGTCCGAT
GCTGTTCAACCAAAGTACCCACCATAAAGAACATATTTCAACCAGCATTTAAAGTAATG
GGTACGTAATTAATACTACGAAAGTCACCTTTCTACAGTTGGTATTACTGTCGGCACAA
AGTAACTAACCCGCACTGGTTTTGTAACGATTTTCTAGGTGAGGAGTAACAATTACGTA
ACCTTGACGTTATAGTTACTTATCCAATTAACACATCGTTACTAAACAAAAACGTAAGG
ACATAGTAACTATACGTTGTACAAAGTTTAATTTACGTTGAAGATACGTTGATGCTCGTT
GTTTTTCGGAATTTTTCTGGTTTTCACTTAAGATAAAGATATAATAATATTTTTCTATTAG
AGGTAAATTCAAATACAGTGGCGAGTGGTTCCAAGAAATGAATAATATCATCTAAGCCG
TAACACAATAGCAGTGGCGTTTCAAACATATTTAAAT

>Cluster-12689.28205   MsigOR24

AAGCTGCAACACGATGTTCTTAGATGGTGTTGCTTATAAAATACTGGGAACAAGAGATT
ATGCGAAGTTGTTTTTGTGGGTTCCAAAAGCGATATTTCAAATAGTCCAATTGTGGCCA
ACTGCAAACCATTTAACAACAAAGTCATTATTTATTTGTTTCTACCATGTAACAATGAAT
TTATTTCTTTCAATAGGGGTTTTTCGATATGTAAAGGATAATATTTTCGACGAAAACAGC
ATGATTCTTCTTGTCGTACTTATGGGTAGTTTCGAAGCAATCGTTAAAAATATATTGCTAT
TTTGCAAAGGACCTGAAATAAAACAATTTTGTGATGATATCCTTATAAAATTCTGGCCTC
ATGATTTAGTTGGAGAAAAACTAAAAGCTAAACTACGGTTCTTTTATCTTGCGGGATCG
GTGTGCCAGTTAGCATTTTTATTAACTAATATATGTGCTGGATTAGTATTCGTGTTACCAT
CGTTTCTAGGGGAAAGAAGATTAATTTTTGCAGTGGACTATCCCTTTGACTGGACATCA
TTTCCTGTATTTGAAGTAATACATCTTTTACAAATAATTACAAATCTGACGTCAATAATAC
CTACTGTCATTGGATTTGACAGCCTGTTTATGGGAATAGGAGGTTGCATGGTAACTCAG
ATTCGAATTTTACAACACTGTTTTTTGATTTTTAATACTTCTGCAATGAAAAATACAAAT
ATTAGGTTAAAACATTTTTACGAGGAGGAAAATTTGCGTTATAAAGAAAAACGAGAAT
ATTTTGTGAAGTGTTTGAAGCACCATCAACTTCTTATGAGGTTTGCAGAAAAAAATTGGT
AATACGTTCAGTTTTGTTATTCTGATACAATTATTGTGCAGTACTATTCTCATTGCGTGCT
CCATAATGTCGGTAATTTTAAAGAACCAAATTACGATATTTCATATTTGCTTATTCATACA
TGGTTTTATTCAATTGCTCATGTATTGTATCATTGGAAATGAACTACAATATCAGGCCGA
GTTGTTACCGGAATATCTTTTTAAGTCAAATTGGAGGGAACTGAACATCAGTTTTCAGA
AAGATGTGATGTTTGCAATACAACACTCGCAAAAATTTCCCCAGCTTAGCGCTTACGGT
CTTTATGATCTGAACATGACTTCTTTTTTAAAGGTTGTCAAGACGGGATTTTCTATGTTT
ACTGTTCTGTCGAATTTGGCACAAAAATAAATGTTTGGTTTGCAGAATTAAACTTAGAT
TTTACATCACAGCACGACCACGTTCTATAAGTTATGTTAGATTTCTATAAAATATTACTGA
TAATGCGGCGATTTATTAACATTCAAAAAAACACTATTTGCATAGCTTAAACATTGTAAA
TGCAGTACGTACTTATAAATGGCACTATAGTTTAAAAATTGTACATTGCCAATGCAGTAG
TTTTAACCTTAGCATATTTTTATATTAATCCACACTTATATCACTGATCTGTGTTCCAATTA
GAGAAATTAATTTTCTTAAATTGGCCACAATGTTGACATTTTACACATCACGCCTAATTC
ACCCCATTCTGCCATTCAACGCCACAGGGTGAAGTAAACTATAATATGTTTGCCACAAG
GTGTGCTTTTATTTCATTTTAAATCTTTTATTATCACCGGCATTATGCCATAGTATTAAGTA
CTGTAGTCATTAATGTGCCAGGGTTAAGTGGTGTATTTCATTATGCGTAATTGACAAAAT
ATACGTTTCTATATAACAAACAAATATTTCATTGGTGAGCAATTAACTACCGAACATAAA
ACGATAATTATATAGATATTTGTCACTTATGTCCACAAGTCAATAGAACATGATGTTATTT
GGATTGTATCAAAGAGAATGTTACATCTTTTCAGCGCCAAAGTCCTCTTAAGATGTCGC
ATCTCTACACAGTCCCTTCATTATGAAGTAGTCCATAAAATTTGAAGTTAAAATTAAGAA
AATCTTATATTTTGAGGTATTAAAAAACTCCAATAGAAATTCAACAACTGCCTCTTCAAC
ACGAATTTTATTAAAACTTTTTCATTTAAGGGCTCTAATTAAAAAATCACATTGTACTGG
CTACTAGGAGGATTTATGCTTATTTAAACAATTTACTAGTTTATGCTGATAAGAGACAGA
TTTTGCTCTTTTCTTGTTAAATAAACATTTTTCGTTTAGTTAGAATATAATTTGAATTTTG

TAATCTACATTTCGGTTGAAAATTAATTTGTGTTGAAGCTATGTAATGGTTTTTATCGAGT
TTAATAAGAAAAATGTATTTTTATTGTTAATTTCACAATGTTTTAGTTATTTAAAATTAAA
ATAATATTTGATATTGATTTTTCCGACTTTTTAAAGAAAATGGATTTTTAAAATGAATAAT
ATTGGGAAGTTATTTTCCGTAATCAGTTTAAAAACTAGTCTC

>Cluster-12689.28663 MsigOR25

TTAATATCCGAGAATGAAGACGATATTTTTAAAATCCTTTGTAGTGGAACTGATTCTGGT
CAAGATTTGCGGCATATGGGATTACGTATATAACAACAAGAGATGGGAGCATTTATATCG
GTTGTATTTTTTAATGTTGAACGTCCTTCTGGCCATGTACAACGTCATGAAATTTAACGA
TACGCTCCAAGAAACGACATTGGTGGGAGCCGTTGCCGCTGGTTTCGTATTGCCGATC
GCTCTCATGGGTAACGTTCGATCTTTTTGCTTTTTCGCGTACAGAAAGGAGTTCTTTGA
GTTATTGAGCACGTTAGACGATGAGATTTTTCAACCGCGCGATGCCGAAGAGGTGAAG
ATGGCGCAGGGAATGTTGAAGTATTATCATAATTTTAAAATTGCGATGTATGTGGGATCC
TTTTTGCCGTCCTTCGGCTGTCCCATCGGACGAATATTATTTGGTGAACGAGGTCAATC
GTATTGCGAAGCTGTCATTACAAGTAAACGAGGAGAGGCTATCTATATGTTTCAAGCCA
TATCTCTCGGAATGATCTCCGTTATAAACGTCGTAACGAATTATTTCATGGTTGGGTTTT
CTCTGTTTATCGGATTGCAGTGTGATCGCTTGTGTTATCGGTTACAAAATATGGAAATAA
CAAAAGATGGTACACAATCCATCAAACAATACGCTGAACATCACAGGCGCATATTGAG
ATACGCATCTTGTACGGAAAAACTCTTCAGCATCATATATTTCACATTTATGATAATGTGT
CTGTTAGCATTCTGTATGACTCTGTTGATTATCTCTATAATTCCGGATCGCTATTCATTCC
AATGTTTACATCTAGTTGTGTATCAGATGTCAATTTTTGTAATGTTATTAATACCTTGTTG
GTTTTCCACGCAAGTGAATACAAAGAGTGAAAACATTCCGGTAGCTGCATATTTTTGCC
AATGGCCAAATCAATCGAAAGGTTTTAAAAACGAATTGATCTTTTTTATAAAGAAGTCG
CAGACACCGATTCAGTTCAAAGCTATGGGTATCGTTGATTTATCCATGGAGACATTTATA
TCGGTAGTGAAAACATCATTTTCATATTATACTGTGCTTAACGATATACTATTTGAACGTG
AAGTTTAACCATAATTATTTTGCAATGTCGGTTATAGAATGCAATCAAGCTTTGAAAAGT
GAATATTATTCAATTAATGATGAATAAAATTTAATTTAGGAATTAAAA

>Cluster-12689.29193 MsigOR26

AAGCCGTAAACTTATAATCAACTTTATCGACTTTGCCATATAAGAGCGTGACTTCGATCA
GCGTCAATAATATAACATATAAGTAACACTTACTCAAAGTCGGTTAAATTACTCGAATAT
TAAGAGTGGGACATATATTTCGTATACTGGAAAATGTTTTTACGGATACCAAAGCTGTTA
CTACAACCATTATACTTCTGGTCTCACCAAAATCATAATTCTCATCAAAGAAACTTCATG
ACGGTCATCTCGGTAATTTGCTTGGCTATAATTTTTATTGGAACGTGTTACGATGGTTCG
TTGGTATCTAGTAACCATTTAGATTTATTTTTACAACTAAATGTCTCTGCAGGAATTTTAC
AGGGAACTGCAAAATTATGTACATTTTGGGCGAATTATAATACGTTTAAGATAATTATTG
ATGACATCAATACTAAATTTTGGCCCTACGATTTATTGAATATTGTCAAAGTAAAAAGTG
AAATAAATCTATTTTACAAACTGATCACGTCTATTATGGTGTGTCTGCCAGTTTTTACTT
GCATTTCTGTTCTATTGATGGTCTTCAAATTTGGAATACATCCTTTTCCGGTGGCTTATCC

TTATTTTAATGCCAAAAGTTTTCCATACTTAGAAATAATATGTTTAATGGAATGTGTTGTA
ATTTTTGCCGTTTATTGTGTTATGATGGGTAGCGATTTATCTTTTATGGGAATTTGCGCTA
GTCTTACAGTTCAATTCAAATTGCTGATGAATATGCTTGTAATACATAAGATGCAAGATA
TGGATGAAGTAAACGCTAAGTTACATACAATTGATAACGAAAATTTAAAGAAAAAATAT
AAGGATGTACGAAAACAATTTTTCGTAAGGTGTGTGAAACATCATCAACTGTTATTAAG
AACAATCAAGAATGTGAACAAGATGTACAGCTTTATCCAAGTAAGCCAACTAATTTTTA
CAGTCACAGGAATTTGTGGAGGACTCTTTGTTTTGAATTATGTGGAAAACCTAAGTCTT
CTATCAACGCTATTTGTGTGGTCAGCCATGGTTTGTTGTCTGAATGAACTTTTACTGTAT
TGTATAATAGGGAACGAGTTACATTGTCAGGCTACTATTTTGCCGGAATACATATTCCAA
ATAAATTGGGATGTAACTGGTGATAAAGTATTTACCAAAGACGTTATGTTTATGTTGCAT
CGTTCTCAGGATATTCCCCAGATATCTGCTTACACGTTGTACGATATAAATATGGAATTCT
TTATACAGGTTGTTAAGTTCTCCTTTTCTATGTACGTATTTCTCTCGACAATGCAAGAAA
TCAGCAACGCAAATTAATATCACAAGGAAGGAGCTAAGCTCTTCAAGATGAAAATTAA
CTGAAAATATTTGCTGTCTGAAAAACACGAACTTAGATTATATAGATATAAGTAACTAGT
GTGAACTGGGTAATAGCTCAACTCAATATAGAATGGAATAGAATTCGAAAAATTAAAAG
TGTACACTTAATATTAATAAATAAAACTACAAAGATTGAAAAAAAAGATCGG
>Cluster-12689.29396 MsigOR27
TTTGCATCTTCATTCTATTTTATTTTCTACATAAAACTCATTAACATTTGATCCAGAGACA
TGGCGGGTAACTTCATTGTTTAATATATATTTAATAATATAATTGACTGCCTTTATTCGCGT
GAAGCATTTATAAGTGTGTAAAATGAATATGATGTTTGAACTATCTTTGAAAAAAATGTT
AAATTAAGAATAAACATTTTTCCCGCAGTAATACAAAATGGTTTCTGTAGTCGTTTTAAT
ACAAACAAAACCAATTTCTTCAATTTTATTGGATAATTAATCCACTCGGTTTTATAAACT
GTTACAGCGATTTCTTGAATCTCTAGAGTTAAAGTTTGACAAATATACGAGAAAATAAA
TATTTCTCCAAGTGATGCAATCAAGAAGCTTATACAACAAAATGCTTCTGCCATGTTGG
TAACCATAGGAAGAATGTAACTCAGACTACATAAAAAGAATGTAGCACATCCATATTGA
ACCAACATTTGTATTGTACAAAGTTCTTCCAATATTTCGAATCCCCTGTATATTTCTTGTA
ATTCATTAACGCATTTCACTAATCTAATTTCTTTGATTAAAAAGATATTTTTCGATGTTTG
GTCTTTACATATATTTGTATCGATGTACTTAACTGCTTCACCTACAATCTGAGCCTGACCT
ATCAAGTGAATTATACAAGCCAAGAAAACTGTGTCAACAGGTAGGAATGCGTGTAGAA
GTGCAAATCGTCCATAACATTGGAAAAAATAAGCAAACCAAATATTTTTTCCACTATCC
AACGACGTATCGAAAGGAAAATAGATACGCAGTGAAAATTTTCTGTATGAAGATGTTTT
ATTTAAATACGGATTAAACTTTTCATATATTTCAGCATTAAAATCATTGTAAAACCAAATT
CCACTCGTAACAATAGATGTTAATAATATACTTAAAAAAATGGTGCAGCATATTAGTCTG
GTATAAAACATTGTTTTTATGCGATACGTTTCCAAGGTGTTACTCGTTTTAGAGAACTGT
GGAGTAGTTTTTTTCCAAACATCTTGGAATTGTTCATAACTTCCGAGTAGGTGACATTTT
ATATTTCTTCCTATAGTCGAAGCTTCAATATAATCAATAGCAGTAACAGCAAAACAGTGA
AAATTAAATGATGGTCTTCTAATAATAGTTTTAAGATCTTTTAATCGATTTCTAGTCAACC

AATACACCGATATTCTAAATACTAAAATTGTATCGAAAATGGTCAAACTTACAAAAAAC
ATTATCATTTTGAAATCATCATCAGAACTATTAAAGATATTCAGCCATTCGCAACAGCTA
AATAACCAAGAACAAGCAAGAAGATTCGGAACAGTCACTAAGTAGTAAAGATATTTAA
AGAACGGTTTTGCGGGCGGTTGAAGTCCTGTAATTGTAAAGAAAAAATCCAACCAAGT
GAAAAAATCGCTTGGTACATATTTTTCCATAAACTTGTTAATTTTAAGAAAAACCATTTC
TCG
>Cluster-12689.29608 MsigOR28
TTTGAATATTAGTGAATTTATTGTGTAAAAAAAACTTATGACGGTACAATCATTAGTGAG
AAAATTCTTTGGTTTTTTTGCTGATGTTGCTTGTATTTAATAAAATCGCAATCATTAAATG
TACGCTAAATCTCATGCGCCATATCTTCCATGGTTCTTAGAAACATATAATACGAAACTG
TCATCTTATACATCTTTAAGCCGCTTTCAAAGTTTAGATCTATAAAATTTGCTGCAGTTAT
GTATACTGGTTTACGACTCATCAATGCTATCATTGTAATTATTGGTTTCTTTAATTCTACA
GAATTATCATGCCAGTTACAAGCAAATGCGGCGCTTTCTGTATTTTCCGCCTCAGATGTT
AACGATTGACCGGGTATAATATAAAATGACAGAAATTCATTTTGTGCTCCTATTAATAAC
AGTATTCGTAAAAATTGTCCTGATCCAGGTGGGCATCTGGCGATGACAAATATGCCTAA
ACAATTAGATATGACGAAGTAACCCATGAAAGCCAAGTTGGAAAAATATGTAACATTAT
TGATCCTATCGACGTACTCTTTTAGAAAATGGTGATGATCGATACATTTCTTAATTCCCCT
TTGGAATAAAATTCTATCTTCCTCATTGTATATCTTCGATTCTAGAACAACTGCAAGTTCT
TTATTAAGTAACTTCCATTGGGCAACAGTTATTAGTATTAACGTTCCAACTAATACATCA
AAAAATACAATTCCCATATAAATAACCATAAAAGAAAAATCCTGTACGAGATATAATACA
GCGTAGGGAGGATTCTTTGGTACATACGTTGACATTGGTAAAGTTCTGACCTGGAGGAT
AAGCGGGCCGAAAAGAATATACGTAGTAGTTATTACTCCGAATCCCGGGTAAGCCCATA
GAAGGATCTTCAGGTTTCGTAAAATGCGTTGGCTACTTTTTCTCTCCTTCTCGAATGGA
AACTCATCGATGTTCCAGAAGAGTTTTGTGTCATTTAACAACCTGCCCATGTCCTCATG
TTTCACGAACAAAACTGTTATTTTGACTATAGCGTGAATTAACAGGAGAATATTGCCAA
TACGGGGTAATAAAAGTAATATATCTGAAGTATCCTCGTAAACGAATTGCAAAGAGCAA
ATGTATATCGACCACGTTGAGATAAAAACACTAAAAAAGTAGAGTACTTTCTGATAATT
CTTTTCACGAATCAGCGGATTTACCCCTACAGTACTTAACATCATAAGACATATTCTTAT
ATTAAAAAACATATCGCCGACGAGGATTCCAAGAAATGAAAATCCTATTTAAGTATTA
>Cluster-12689.29934 MsigOR29
GTTTTAAATGCTGTTTATATTAACTATATAAATAAATAAATAGATAAATAAATAAATTGGTA
TCTAATTATATATTTATCGTGTGCACAGTTCGACATAGTAATTCATTCGGCAAATAACTTT
ATCCATACATAGTGTTCAACACGGCCATGTAAGAATAAGAAGATCTCAAAATCCCGGTG
AAGGTGGCTAATGTCAGGTCGAAGATTCCAGCATGCAAAACAATTGGTTTTACGCTCC
TCGTCATCAAATTGAGTAACATTTTTTGTATTTTCACATCGCATTCGTGCCAATTCGATA
AAAAGCAAGCATCTGTGACTTCTGAACTCTTAAGCAGAAATTTTTGACCAAACCAAGT
GTAACAAAAGATCTGAAAAATCATTGAAGCTAAAAAAAATCCTTGAGTAACAAATCTG

CCGGAAGGAAATTCAGTAGTAACGGCCAACTGAAATCCCAGGAAACAAATTACTATAA
CACTTCCAATACATTGAACGAATATAATATTGGCGTAGAGAGCTTGTACGTTATTTATGT
AACTTATAATTTCTTGATGCATTATTACATACTTTCGTACGTCCTTTTCGATTTGTAATGA
AGTCCGAGATGTGTCCAAATGCATCAATTTGTACATCAAAATATCGAATTCGCAACAAG
CCACATCCAACAAAACGTAATAAGTGATATCCATGCTGGTATTGGGCAATCCGCTCATG
ATAAAACAAATTGTTTGGAAACAAAGCGTAGGATAATAAAATTTCGGATCTTGGAGATC
GAATGGGGACCAACTAATCAGGATTACGGGTCTGAATTTATGTAGTATCAAGCAAACGA
CCACTTGAAATGCCAATCCGCCGAGTACCATGAATCTTTGCGGAAAATACAAAATATTT
CTAGTTCTGTCCTCCTTGATAACGTGATCGAATATTTGCATCGGAATGCCAGACACATCG
ATAGATTTCAAATGGTTTTCGATCTTCCTCATGATTTTGTCCCCGAAAACGAACAAAGG
TAATTTGAATAAAAACGTGATGCTGATTATGATGAAATGAGAAGCCTCCAAAAAGTTGT
CTATATCTTTGACCACCAGCAGCATCAACATCAGACCGAGCTGAGCGCAATTGATCAAG
AAACAGAAAGTTAACCGGGCATATCGGAACTTAACTTTCGGATTGCGTATCGGGTACAC
TCCGCACCATTCCAGAATAGATTTTTCTACGTTGAACGCCTTCAGAAAATCCTTTAATG
AGCATATTAATACTACTGTTAACACTATTCCTTGTACAATTCAAGGATTCATAATCCTTAA
TTGTAGAAACTTTCATATTCTACCTGTTCTTATTTTATTGTATTATAGTTTAAATCGACCCA
ACTATGAGTTCAATATTTATTTTCTGTAGAGAAAAATTCGACTGAAATTCCTATGTTGTT
TAAGCTTTAATTAACAGGAGCCTATTTTCCAAGGGGATGAAGTATTTTAGATTTACTCAA
TAAGTTTGAGTTTACGTAATGAAAGAATCTATTCCTTAATTATCTAATTTTTTTCATCTG
ATTCCATTTATTGCCGGTTTTCTAACTTTTTGTCTTATCTATGCCGTACTGATGACAACAC
AATAAGTTTGAAACATACATGTGTCTACGACAGCTACACATTTTCATTTTTTTAAATGCA
TATATTACTACACTACAGATACTAGTGATCGCCGTGATCCAAGCTTTACTTTCCTCTTTCC
TATACTTTCTCTTCCTAATCTTTTACCACAAATTCTTATAATTCTCTTTGAGGTCGGCCTT
TTTAGTCTATCAGTCCTTACTCCTTTGTCACTACCTTAGTCATCCATATTTTTGATGGCAT
TTTGACTCTATGTCCTAACCATCTGATTAGATAATAGTGTAGATCGCAGATGTTTACGAA
TTTTTTTAAGAAGCATTAAAAACACTAAAACGTCGATAGGAAAATGGAGACCCTGTTG
AGATATAGAACTGGTATATTAGGTATAACGCAAGGAGATCAGCCCTTTTAAAACTGTGA
TTTTTTTTCAAATTTTAAATCTTCTTTCCTTCAAACGAAGTAGTTTCATAGTTATAGTTTT
CTATCAATTCATGCAATTATCGACAAATTTTTTTCTTTTTTATTTTACTCTTTAGTTAAAGT
TAGAGAAAAATACTTACTGGTTTAACTATTTTAGTCATTTTCTACAGCGTATAAGCGTCA
CACAGTTTTTTAGTATATCTCTCTATAAAAGAATCTATTGTACTTGGAAACCTTGAA
>Cluster-12689.29945 MsigOR30
TTCATGCTTCAATCTACTTTATTGACTAAAAATCCATATATACTAAATTACTACGAGAAAG
AATTTCAAGTTCAATATATAGTTTGCTTCTTAGCGGAGCACGAAAGTTACAAATGAATA
AGCACTCTTTATTATCCCAATGAAAATACTCCAATTCATTATTGCTAATCCTCCAATTTGC
AAACCTGTTTGTTGATTACATCTGAAGATTAAGATATTCACCATTTTCTTTACGCTTTCTT
GTTTGTCGTACCATTTTATTTCGTAGAGGTCTTCGGCAAGTGCAAAACTCTCAGCCATT

ATTTCGCTGGCAAAATAATAGTATACGGCAAGTCTTCCGACCATACACACAATATACAC
GACAGCTACTGAAACGAATATCATGCTTTTTTCATTCATTAAGAAAACAGCCAGCATTG
CCAATTGCAATGAACTTTGAAGAAAATCGAAAAGCATAACATCTCTCAATGAATCATTA
AACATATCTATGAATCTCATTAGTTTTTGATGATCGCTCACACACAGCTGCATTGTCACG
AAATTGGCTTTATCTTCATCGCAATTTAGTTGTTCCTTAATTTTATTTTTGTACGATTCGA
AATTCCTCAGAATATGTTTAGTAATATCGAATTGTCCCAATATGTAAGCTATAAAACTGTA
CATTGCCACGTCCGTTCCGTAGTTAATTATCAAACAAGAAGTGGATCCCACCTTTTCCC
AAAGGAGGGCTAGTAAGCGATGTTCGTGCGTATTCCAAGGATACCATGGATAAAGCGT
GAGAGGCATTTGTACGTAAGTTTTATTTGAAACAGGATCGACGTAGTGTTCTTTCGGTA
CAATAAACTGACACAGATAGAGGAAAATTGCAGAGTCCACGCATTGCAAGTATAGATA
CCAATAGTTCAGTTTGTGATTTTTTCTAACATTTTCTCGATACATGGCTTTAACCACAGT
ATCATCAGAAGTAACAATTTGTTTTTCCCTTTCCTCAAGACTCAATATCAATGCGGTGGT
ACTTTTTCTCCAAAGGTACGCTTGCCTGACCAAACCTATTATAAATACTGTTGCCGATGT
AACGACATTGGTCATTATTGCTATATCTTTGTCTGTTTTCACTTGAATAACGAATGTGGC
CATCTGTGCTAGTGTGAATAAAATTGCCAGGATGTATACAAGTAACGAATATGCGTAGT
ACCCATATTTATAAAATTTATTTTTGGCATAGATGAGGGGCCAAATTCCACCCGCTAATG
TGAAATAGATCACCACCTTCATATGTTCAATTTTGGGCACCTCCATCTTGCAAACAATTT
TTTAAACAATCTGGCGATCTGATCGGTGCAGTGTTTGTGATACTAGTTTTATTAATGTTG
TGTTTATTAATCTATCGAGTCAGTTGTAGCGTTCTATCGTTTATGTTTTGCGCATTTAAAA
AAAAAGGAACAATAAAATTTAAAAGCAATGCCTGTCATAGATAACTGC
>Cluster-12689.30745 MsigOR31
AAATGAACGAGTTTCATCTGGATTTTAAAGAAATTATCGGTATCAGTTGTAAAGCTTTG
TATTTTTTTGGGGTTATGTATCCCAAATTTGATGATTATAAGTTATTTTTAAAATATGCCCT
GAGAGTTTCTGTTGTTTTTGGATTTTTATACTTTGGTTTAATGCTAACTGCACTCGCCCA
ATTCTTGCGTGTGATTGGAAATTTACGAGAAATGATGAAAGGTACATATCTTACCTTTAC
AAATATTTTAGCAATGGGAAAATATTACGTGATTTTTAAACATCAACCTAATATATTAAAT
TTAATTAAGAAAATTAATAGAAAGGAGTTTCAATCGAAATCTGAAACACAAACTCAATC
GCTGAAAAGTTACATTTTACTGTCGAAAATCTATTCTGCCATTTTATACACCGGATGCAC
AGTTACTGCTTGTTTTGGGACTATTTATCCCTTTACTGAGGAAAATGGACCGTTTTTACC
CTTACCAACGTACATACCGTTTGATACTAGTAATCCGATTGTCTTTGGGTTGGTCTATGC
ATATGAGATAGTAGCTGGAACGGTTGCTGGATATATGGTTTTAAGTGCTGATTGCTTCAT
AGCAAGTTTAATTATGGTGGTCGTCGCACAACTTAATATACTTAATGACTCCTTTGTTAA
TATAAGCAGGATGGCTGAAGCAGAGTTAAAACAAAAGCGATCAAGAAGTGTCGGATAT
TTTCAGGAAATAATAAACAGGAAAGTTTTCGAATGTGTTGAACACCACAAAGCCATTG
TGGAATTTTCCGAAGAAATATCTTCAATATTTACCCTAATTTTATTTATTCAATTCATTATA
AGCGTTTTTGTTATCTGCGCTACTTTCTTCGAAATGATGCTGGTGCCCATAATCAGCATG
AGATTTTTTTCAATGGCGTTATATCAAATCTGCATGATCTTAGAAATATTTCCTATCTGTT

ATTTTGGCAATGAAGTACTAATTGAGAGCGATAAACTCACAAATTCAGCGTATCATAGC
GACTGGATAGATTATTCTGTGCAGATAAGAAGGAATTTAATATTTTTCATGGCCAGAAGT
CAACGGAATTTAAAATTAAACGCAGGTGGATTTTTTACTTTGTCTTTGGATACATTCATC
AAGATATTAAAAACCTCTTGGTCATATGTAGTGGTTCTAATTCACATGCAGAATAAACA
ATAAATCACTCTCAAAGAAGATCTTCTTTATTTATATTTGCACTTAACATACTGTAAACGT
ATCACAAGAAAATGAATAAATTGCATTTGTAAAAGCAAA

>Cluster-12689.33814 MsigOR32

TTTTAGTATAATATATGCTTTATTCCACATTTTCTCTTAACGTTAGCCGTGCTCCTAATAAT
AAGCAACAAACTATTATTGCAAGTGCATTGAAAAAAGACATCTTAAGTAGTAGGTTTTT
TAAAGTTCAATGTGAATCAAGTATTGTTCTATTTAGCATATTTTTTTCTGATATATACGCT
TTATTTTTATGCAAGGTGGCTTAAAACCTGACACATATAATTTGAGAAGTGAAGTAAAG
TTAAAAAATATTGATAGTATTTCAGATAATTTTTTCATTGATTCTTGTCGTTATTAGACTAT
AAGCACGAATATTACAAATTATAGTTTTTGTGTCTCTATTAATTTATTTTTCTAATTTTTTC
AACTAAGCTGGTATAAAAAATATCTATCTAAATGTACAAATAATTAGATAAGAAACAAC
AAAAAAAAAAGAGTAAACTATTTAATACCAATAAAAAATTTAATAACACCTACTTTAAA
ATAATCATATTTATAGAAGAAAAATAATATACGAGAACATCAATGTACTATACTAATAATG
TTGCGCCTTTATATTTTATTATTTCGTTGCAGACTAGTAGTAGAAGCGTACGACTGAATG
ACTAACGTTGATAGAGTTAACTTCAATGAAATGACAAAAGAAACTAACTTTAATATGAA
ATCAAAACTTGCCAGAAAAGAAAACTGTCAGAATGACAGAAACCACAGAAAAAGTC
AGTAGCTTTGAAGAGACATTTAAGGATAAAATGCATTACGATCTACTTCTGGCTACTTCT
GTGCCAGCAATCAGGACAGTTGTTACTTTCGATAAATCTCGGAAGTCTGAAGTTAGTTG
CACATACAGCTTTGAGATATAAAGTTCCGGTCATAGCATCGAATAAAAAGAAAAATATG
TGCGCTGAATATTGGACATGCACTTAAATTACTAGAAGTATACAAAGTTTAATCTGTGTC
CATATTGACATCTGAAACTAAATCAGTTACGCCTGATTTAGTAATCTACCACAACATGAT
AGAAAAAGTGAAACTATTACAGACGTTACTCAATTCGAAGCAAATTGTCCAACTTAAC
AAAATGTATGGCGAGAAAATAATGGATAAAAACTTTCGAACTTTTAATGAAAATTCTGA
AGATTGGCCCAAAATGTAGAATTAATATTAAAAAAGTTGCTAGTAGGATCTATTTGGTAC
TCTTGTCAATTTTTAGTTGATACGCTTATGAAAGCAGAATATTTCGCACGCTTAATCAAA
TACTGACCTAACTCAAAAACTCAGCAAAATGAGTTGCCTATACGGTAATACTCAACTCG
GCGTCCTCTACATTTTTATAGAAAAAACGTAACAATCTGACACTATTTTACCTACTTATG
GTGTAGTTAGTCTCTTCTCCACTAGAAGTTTTTCTGAGCATGGTTTAAAAAGATAGTGA
CCTAAGTCAGTAATTGTCAGACGGCTTGAGAGTTAGCACGTGTTAAAAGTTTCAAATA
GTGACTCAATAGTGATTACGGTAGTTTATGAGCAAAATATCAGGTAAGTCAAGTTAAAT
TTTTTTATAAAACCAAGACCGTTTTTGTATTGGGTCAGTTTATTAAAAATAATTGCCAAT
TAAATGGGTTGTATTAAGTTTTAAGTAGATTTCTTTTTAAAATTGATGAACATATCAATAG
CTTATTAGGTATTAGGTCTATATATTACTAAAAAATTTGTGAAATTTAGTCCTAATATATTA
TTATTTATAATTGAGCCAGTTTCGTACTAAACGGTGCACTGGATTGTTAGACGTTTTGAG

AGATCAGTCTCTTTTCTACTATTTTTCTTTGTTAGAAATTCTTTCTCGTTTTTATAAAGAT
GTCATAAAACCATAAAACACGTTATACAATAATTTTTTCCTCTCCTAGCATGTCGGCAAG
ATCAAAAATGATTATGAGTATGGTACCCAATGATTCTACTAGATCATCAGAGATACATAT
AGAAAAGGCGCCGTGTGAAAATGATCAGGTAGCTATTTAATTATTTAAAAACTTGTTGA
TTTAGTTCAGAAATCATTAAACTCTATTTCTTTTAACCATAGTCTAAAATTCAATTACACA
AGTTGAGTTTATTTGTATAAATCAGTTTATAAGAAATTTTACCTATCTTTATAGATTGTGT
CAAATACTATACCGGACAACAAGCCATATTCAAACACCAGAACTAACAAACAAATTGT
TAATTATCAAACATCACCTATATACTTAAATGAAAGTTACGTGTTGGTACCCAATAATTG
TAATAGATCACCAGAGGAGGTACATATAGAAAAGGCATCAGAGGACAATGATCAGGTA
GTTATTTATTTTATTTAAGAACTTGTTGGTTTAGTTCAGAAAGCTTTAAATTAATTTTTAT
TTAACTACAGTCTAACATTCAATTAAAAAGTGGACTTTATTTGTATAATTCAGTATATAAG
AATGTTTTTCTATCTTTACAGATTGGGTCAAATACTATACCAGACAGCAAGCCATATTCG
AAAGCCCGAACTAGCGGACACATTGTGAATTATCGTACATCACCTATACACTCAAATGA
AAGTGATGTGGATTGTAGTGATGCTGATCCTACTTACGACTCCAATCAGGCCGGTTCAA
ATAAAACAACATTACGTTTGATCATTGCACCTCGAGACCGTTCGGCTTCAACATCAAGA
AGCTTTTCATCTTTTTCAAACAGCTCTTCATGTTCTTCAAGCAGCTCGTCATCTTCTTCA
AGCTGCGCTTCATGTGTTTCACCGTCGAGTACGAATGCTCCAGTTATCGAAACCAAAG
AACCAGGATCCCTAAATATTATAGAAAATGTAGTAACCGCATTGACAAATACATTAGAC
GAAAATACCCATTTGGAAAATGATAAAAGAGGCAGAAAACGTACGAGGCGCCCTGAA
AAGTGGAAACAAAAGGTGGCTAAGAGATTAAGAAATAGTGGGAAGGGGTATGTTACT
ACAAACAATAAAACTGTCAATGCTCGTAAAATGAAACCATCATGTCCAGAGCAGTGTA
GACTTAAGTGTTCTGAAAAAATTAATCACGATCAGAGGAGTAAGTTATTCAACTTGTTT
TGGAATTTAAGCAGTATTAATAACCAACGTTCTTTTATTTCGTCGTGTATGACTAACATT
ATACCAAAATACAAGTATACGAATGCTCTCCACCCAAGGCAACCAAATAAAGCTTTTAG
CTTTATAATCAACGGTACGTCAATTAGAGTATGTACATTGTTTTTTATGAATACTTTGGAT
ATTTCGAATCGTACTATACGCACCGTTAAAGAGAAAACAGATGAAAATGGCTTCATAAA
GACAGATGGGAGAGGGAAACATCAAAATCATACAACGGCCGATGAGGCACTTGTAAC
AGATATTAAAAAATTTATTGACAAGATACCACGAATAGAATCTCATTACATTAGACAAAC
ATCCAGTCGTGAATTTATAGATGGCAGCAAGACTATCACAGACATTTTTAAAGATTTTC
AAGAATCGCAGAAGCAAAAGGATAAACCGGCCGGTAAATATTGTACGTTTTACAAAAT
ATTTACAACAGAATACAACATATCCTTTTTTAAACCCAGGAAAGATCAGTGTGATGTTT
GTATGCAATACAGTAACTCTACAGCTGAACAAAAACAAGAAATCTTTCAAGCGTACAA
GATGCATCAGGAAGAAAAACAATTAAGCCGGCAAGAAAAACGTAATGACCGTTTGAA
AATAGACCAGACAAATTTAGTAAGTGTGTTTGACCTCCAAGCTCTATTACAGTCGCCAA
AAGGTAATACATCATCCTTTTATTATAGATCAAAGATGAATAGTTATAATTTTACCATAGC
TCAGTTAACTAAAAAAATCGAAGTCAAAGAAAATGAGTCATATAGCGACGTTCACTCT
TTTTTTTGGAATGAAACGGACGCTAAACGTGGTGCCGTAGAAATAGGCTCAAGCTTATT

TAAATATTTTCAAATAGTTTGTGCAGGACCCAGAAGTCTTCCCGAAACTAATTTTAAATT
AACGTTGTACTCAGATAACTGTTGTGGCCAAAACAAGAATAAGTACACTGTCTCTCTCT
ATATGTATGCAGTATCGCGTCTAGATATTGCATAATATTACCCATAAATTTCTCATCAAGG
GCCACACGCAAAATGAGGGCGACAATGTCCATAGTTTTATAGAAAAGGAGATTCAAAA
GAACTTAAAGTCCGGTCCAATATATTCGCCACATCAATATGTTGCGTTAATCAAAAATGC
TAAAAAGTCAGGCAAAAAGTTTCACGTTCATGAACTTTCGTATGACTCATTTTTGGAGT
TGAAAAAACTTCAGGAAGACTGGGGTTATAATTTTAATAGAGATAAAAATTGTGACACT
AAAATCTGGAATGACATAAAAGTCATTAAAGTAATGAAAGAAAAGCCTTTTTCATTTTT
TTTACAAAAAATCATATAAAGACGAACATTTCATTGAAGTTTGTGTTAGAAACAAACGA
ACGAAAATGTTGTCGTTGCAAGACATAAACATATCGGAAGCACATAGCAGCAAACAAG
AGCTTAGTGAAAATAAAAAGAAAGACTTAAAAGAACTTTTAAATAGAAACTTAATTCC
CAGTTTCTATGCTGATTTTTATAATTCCATGGTATAATGAATTATTGTAAAATACACCCAA
GTTTTAGGAATGTATTATAATATTTAAGTAAGAATGTTAAGTTGGTTATTATTTTTAACTG
TTTATGCAAATCTGATATTTAGTTGTGTTATTTGGCTGGATATGTATCTTTTATTTTTGACA
AAAAATAAAATTTTATTATGTATTTTATTATGTATTTTCTAACAACCGACATAACTATACA
AAGTTTAAAACCATTTTGTTTATATAAAGACTGATATCCGAAAAGGACATTCAATTATTA
CATTTATAAATTTTAAAACAGACCTAAAACAGAATGCTTGTGAGTACTTGGAAACCTAC
CACAATGTTTTTAGTGGTAAACAGGCCCAAAAATGAAACTCCGATTTACTATTTAAAAT
TACGCACTACAGTAAACTTAACTGCAACGTTGTACTGAAGATATCATATTTTCATTACTA
GACTATCATTTAGATCAAAAATTGTAATCATATTGATAAAAGTGTTTCAATTATAAACATT
TATAAACCGCCACACAGAATGTTAAACACAATTTTGCTCGCTCTGAAAATCTTGCATTT
TTGAATTAAGTAAGTTTATGATTAAGCGTGCGATTTATAAATGAATTTTTGAAAATGACA
AATGCACCTCAATTTAAAATTATTGCATATGTTGCAACCATTGAATTGCCCATCTCGAAA
ACGTTTGAGAAACTAAGTTATAATGTTAAAATGGCGAAGGTAGCAGGTGAAAAAAATT
TGAATGAATAATCATTGATTATCATATTGATAACGCAGTTGAGTAGCTATAAGAAAAAAA
CTACTTATAATTGCCAAATTTCGAGTTACATAACTCTTAGTAATAAAATTTGTGTATGCAG
TTTCATCAATTTTTTTTGACCTTCCGTACTTCTCTAAGTTTGAGATCGTTCTGGAACAAA
CTGTATAATATACGATAAGGAGTTTTACAACAACAACAAAAAATGTATTAGATGATAAC
ACAATGACAATGGACGTATCTGCTCCATAAAAAGATATATTACATAAAAATCTATTATTAT
ATTATTGACCGCTTTAAGGGAAGATGTCCTTAAGTGTGTTAGAGCTATTGAGTAGCATA
ACAAACATGCATGTTTTCTGATGAAAAAAATACTTAGCAGAAACAGAGTTTTCATTTCG
TGGTTCAAACTTAAATGCAAAAACTATCTAACCTGCAAATAAATAACGAACCAACCTCA
TTTGAATTTTGTCAGTGACGGGCAGCAAGTTTGAACCATCTAACCAGAGAGAAACTCC
GATTTAATAGATAAAATAAACTTGCACACAGTAGACAGATAGAGGAAGAAATTTGAATC
TGTTATTCATTGAATCGAAATGTTTCTTTGATTTGATAGATCAAACTTGCTGCCTGCTAC
TGAAGAGACCAAAGAAAGGTCTGTTTATGATAAAGTACAAGAATTTACTTGCTTATCAG
GTAAGTTTTTGTATCTTATTTTGAATACAGAAGGTTAAAATGTTGAAACTTTTAAAGGCC

TGTAGTTTCCAAGTGTATTCTTTTATTAGTAAATATGAGTGTTGGTTTTGCTACGCTATAG
CTTCAACATATTTCAAGAGACTTTCTCTTGAAGCTGTCAATATAATAATTATATTTATGTA
TGCTAATTTATTTACGGAACAGGCACTGCAAAGGTGTGTCATTGTTCTGTCATCTAATCA
TTTTGTTCCATAGAAGTAATTTTTTGTTTTGAAATTATAATTAATTACTTTTTGGATATTTT
AAGTGAGCTTACAATAGAATTTTACTAGCTTTTCTTGCCAATGCTATTGTCTTTTTTCGTT
CATAATAATTTAACGGTAATCCCAATTATTCAAACCCCATGTATTCGACTAATTACAATCG
AAACAATAGGAGCCAAAGTGTCAACCCTCGGCGCGACCGGTTAAAGGTTAATATAAAT
GTCGCTTGATATTTGATTTTCCGGTCTTGCTGTGTTTGGGTCCAACAGTAATAAATTTAC
CATCAACTATTTTACGAGAATTGGTCACTAGAATAATTTGTAACTATTTTCGGCTGTTGC
TTTAAAGCGTATTCTGCACGTTTTCGCCTCATATATTGCGGAACTAATTTCTACAATCGTA
GATTAGAACAAGCGGATTTAATTAAGAGAAATGGTTGGTCGTATCATCTTATTTGTCGTA
CATTGTGGTGAAGAATGTGCATGTTGTGTAGCATATTCTCATGATCTTCGTAAAAGAGTT
AAGAGACATTTCTCCTATGGCAACAGATTTAGAGACATCTTTTTTTGCGAGCGAAACA
TCAATATTAAAAGAGTCCTTTTGTCCCTTTCATTCATTTTAATCCAATTTGTTTCATTATA
AATAACGTCGGATATAGTTTCGCTCTCGTGCTGTATTTCATTTGCAAACCAAAAAAATAT
ATACATTTGTGTAACGGTCATACAAAAACCAGCGAAACATCGAGCCTTAATTTGCATAG
AGTCCTCCGTTAATATTGGTATCATAAATGCTGCGGTATCCAAAACATAACTACAATATT
GAATTAAAATTATGTGTTTTAAAGAGTTTTGAATCCAGTCCATCAATCTGATAACATATT
GATGCATCTGAACACAGTCTCTAAGTGTCAAATTATCTGAAACAGTAGTTTCATATTCTT
TATTTTCAATATTCCGAATATAATATTTTAAGAGACTGATTTGACTGAGAAGAAAAACAA
TTAAAGATAAAGGCACTGTTTTATAATAAGAAAATAGTATCATTCCACAAAATACAGTA
ACCAAGTCAAATGATGAACAAATCCATGGATAGTTCTCAATTTTGAAAGGGTACCATAT
TTGAAACACGAAACCTCTTGCTATAGGACAATCTGGATTATACACAATTTCCAATGAAT
CCCTTTTTCCAACTATATACCACATCCACGAGACAATATTAATAACGATTATGAAAAAAA
TTGTAAACTTGTTATTCATCCTACAATAAGCAAAATATATCTCTGACTCTTCTTTAGCCTT
AAAAATCTTTATGTTTTCGTACTCAATTATCCAGCTTAAAAAGTTTTTAATACTTCCTGCT
TTCAGTAGAACTGTTAAAATAATGGCAATAATGAAGTGGATGTAATAAAAGATTATTTCG
AAAACTGAGTTACTGCACCTAGGGTAAAAAAAGATTGTAAAAAGGAAATAATGGGTAC
ATACAGCAAACGCAAACAACAGTAAGTCAGAGAATTTGTTATACATCCGTTTCAGAAA
AGTAAATTCGGCGAATTTCCATTGTCCAGCAATTATCATAAGCACTTTTTGAATCTTCAA
ATGACT
>Cluster-12689.34658 MsigOR33
GTAAGACGGGCCCATTGTGTTTCAATGTTGTATCAACTGCATTAAAAAAAATGTTCTCA
GTTAGCAACATACCATATAATGTGTCAGTGAAATCTCTGCAGATGTGTTTGATTTTCCCC
AATACTACAGAGAAAAATTTAAGAAAATTTTACGTAAAAACTAGTATACTAAGGATTTT
AACCTTAGGAGGTGTTCTTGTCGGAGTTATATCGCACTTTATTAAAATTCAAATGGACC
ACATAGACGAAATTGATACAAGTGAAGATCTTCTTGCCCTTGCCGCCGGTTTTGGTACA

ATGTTCATTATGGGAATGACGACATTTTTACATAAGCGTTGGGAACAACTGGTAAAGGA
TGTTGTTGATGATTCAAAATTTGACAAGCCGGAAGCCATGGATAAAATCATTTATAGAA
ATAATCTATTTGCAACGTGTTTTGCAAGTTATTTCATAGCAGGAGGCTTTGTGTATGCAT
ACGTCACATATAGAGAACAACCTAATTGCCATCGAGTTATAGAAGAAAAGAATATAAAT
TTGCAATGTGACACATTTTTGCCAATGTGGTATCCTATTAAAATTCCTCAATATATCCAGT
ACATAATTTTCCTATTACAGTTTATACTTGTACAATTTGATTTGTTCTCTGTGGCTATGCTT
GTCTTTGTCACATTCGAATCAACAGAAATTTTAAAAGGACATTTTGAACAATTAGGCAA
AGATTTTCACAAAATTGTTAACTACAAGAATCTTAAAACAAGACGAGCAGGTATAAGTT
ACTGGGTGACATATCACAATCATGTAATAAGAATGACCGGTGAATTAAACTATCTATCAA
AGAAAACAGTTGCCTATGTCACTTTGATTGCTGCTTTAGTAATTGCATGTGCTGAAAAT
ATGTTAATAAGTAATACAAAACCGTTGGCAGCTATTTTATTTCTGATTGGATGGAACGTA
GGAACTTTTATTCTTTGCCATAGTGGTGAAAATATTAATCAGTTGATGGCAGCTCTCCAT
GATGCAGTATATACTTCTGATTGGCACAAGATAGACAATGAAACTAAAAAGGATATTGT
TTTTATATTATTTAGATGTCAGAGACCACTCACTTTGGATGCCCTACCACTCGGTGTTATA
AACTACGCTTTGTTTGTGTTGCTTATAAAAACTTCATATTCCTACTTAACGCTCTTAAATC
AATCTGCCTGAAAAGGAAAAGTTTCAACAGTATGGTAATCTTTATGTATTCAACAAGTT
CTCTTCACTACTCACAAACTCACAAATTATGTTTGTAAAATGAATAGAGCAACAAATAA
AACACCGTTTGAATTGTTAAAAA
>Cluster-12689.36417 MsigOR34
GTTTAAACAATGTTTGCAGATGTGGTTATATAAAATTCACACTGGACTCTCCTTCCTACA
ACCCCGATCTTGTACCTTGTGATTTCTTTTTGTCTAAAAAATTAGGTAAAATGCGTTGAA
GGATTTTTATCGTCAGATGAAGCGTTAGAACCTTCTAAAGTGTCATACTAATAGTGGC
ATTGTTGCTTTGAGGTTTCTACGAATGCAAAGTGTGTTGGTGTTAAAAAGGTATATTCTT
GTTTGTTTTCTGAATCCGTATCGTATATTAATGAATCACATCTTGCAATGGATCGGCTAAT
TTATTGTTGAGATTTCTCTTGCATAGTTGTTAAAAACGTGTACATGGAAAAGGCAAACT
TAATAACTCGTACATAGAAGTCCAAATTGAAGCTATATAAATTGTATGAGGTTATTTCAG
GAATATCTTGTGAACGATGTAACAGGATCGTAAAACTTTTGGCATATTTCGTATTTTCCA
TCTCATACCATTTGGCCTTGAAAATGAATTCTGGCAACAGACTCGCCTGATAATATAACT
CATTGCCTATTGCGCAGTACAAAAACAGTTGATTTAGGGAGAAAAGGATGACTCCTAAT
ACGAAAAATACTTTCGTAAAAGTTCGATCTTCCATTGTTGTTAGAATGAAAAGCCCTGA
ACAAATTCCTGTTATGGTGAATGTTGTCTGCACCAATTCCACCAAACTACACATCTCATT
TATTAGGTCTACACTCCTCAATAATAACTGGTGATGTTGAACGCATCTAGCAAAAAACT
CGTCGTGTACGGTGTTATATTTGTATTTCAAATGTTTTCCATTTTTTCTATCCATCTCTCTC
AGTTTGGCATTGACCTCAAACATTTCTGGCGTGTTGAATGTAATAATGGCGTTGCAGCA
AAGTTTAAATTGGGATACGACGCAGGAACACAAAGCCACAAAACAGAAGTCAAAACC
CATCATTACGAAATTGCACATATAGGTCATCGTAAAGAGCTCGAAAATATAAATAATTTC
AAAATACGGGAAAGCTAGGTAATTAATCACCGGCACGTAAATTGGGAGAGGAAGTCCT

CCGAATATAATTGGTTCGACGACAACATCTACAACTATATTTAACACGACTAAGAATGTA
AGGTATACTAAGATCCATATTATGGTAGAGTGGAATGTTTTCAACTCTTTTTTTACTTCGT
CGTCGTTTACTAAATCGCTCGGCCAGAAGTTAGTCATCACGTCTTCAACGATTACTTCT
AGTTTTCGTCTATGGACAAACAGTGCCAGGTATTTAAGCACACCCGATATCATGCCAAC
TCCATAAACTAATTCAAAAAAAATGTCGGTAACGTCTGTCAATATGTATATGCATTGCAT
AGCTACGCCAGCACCCAATAACGTAATGAATATTTCTGCAACGGCGATGAAAATGTATC
TTGGACAGTAAGTAGGTCCGGGCCACATACAAATAGGTTTCAAAATTGCTTTTGGAATC
CATAGAAATAGTTTAGAATAATCTCTCGTGCCCAATACCTTAAAACCAATATCATCCAGT
TTATCCATATTTTATCAGTAATTTGATGTGTTATTAATTATCACTGGGTGAATGAATCTGCC
AAAAAGGTTAGGCTAAGTTTGAATGATGAATATCACTGATACTAAGAGAATGTAAAAG
ATATTGTTGAATTCAG
>Cluster-12689.36840 MsigOR35
CACATGGCGAGATATATAGATAAAATAATGCTTTATTATTTTTAACTTTTGTTTTTAATTTA
TTCTTGACCGAATACCCATAAAATTTAGAAAATAATTATACGGTTATTAGAAAATTTATGT
GTAACTCAAACTTTTCTGATGTCACCTTCAACGACAAGGGAGACATAAAAGTTTATATA
CACAGTAGTGTGTCTTGAAAAGATTTCTATCAAATTTCATAGCTAATATACTCTTACTTG
TGCATCTTTTTTGATAACAGACATATGTTTAAAGTGTGAGAATATTAAGAAAAGATGAG
GCTATAGTGTAACGCTCATCGTATAAGTTGTTTCCGATCTCCCATTGCTGCAAAATTAGG
AGAAAATTTTTTTTTAACTTTACATACCTACAATATAGTTGCCAACTAAAAAATGCTAA
ACTGTTATTACAACACTCAACTTAACAATTAACATACGAGGGCAACGGGAACATAGTAA
CTAAACATGACATCGTATTTAACCCATTTTACCGATTTTTCTCAGAAAACATTGATTTATC
GAGTCAGCCTTACTTTCTTTAAAATTGCAATATTTACAACTTGCTCAAACACATTTTAAT
AAGGGATTGAACATGTTACAAGTAGAATCCTATGTGTAGTTTTAGAAAAAACGCATTTT
ATGTAACATAAACAATTCAGAATACCCACAGTATATAAAATGAAAGATTCAGAATAAAA
CCTATAAAAACGATAACAGCCCTTGTAAACCAAGGTAGGGCAATATTGTTCAATAACGT
TGCATAGTTCAATAAACTAATTGAATTTAATGCTTTTCGAGGTTTCATAAACTTCCCGCT
TAATCTTAAAACATACTTTAACTGGTGTGTTTATTTTTGATAGCAGTATGGATTAATTGTG
CCTTATTGAAATATTTTTCTAGCTCCTTTACATCGTTTGTTTTAGCAAGGTTACGTAAGA
GTACGATGTCTTAAGAATCGTTACTACTGTGCTGTAAGTTATGGGTCCAAATGGTCCAG
CCCTTAAAAATATTGGTTTTGCACTTCTTATTATCACCATTGTTAAGTCACGTTGAAGGT
CGGGTTCTAGATCGTACCAATTGATGCGTTGCATGATGGTACCAACTGATATGCTGGCTT
CCATTAATCTTTGTCCTCCTAGACAAACTATGAAAGTGGAAATCATCCAACCAACCATC
AACGCACTGTCATCTAAAGGAATTCTCGTAAGAAAACTGTAGCCTGCCACTCCAATAA
AAGGACCGGTCAAACTAATATGCACCATCATACATGGGCTAAAGCATTCGTTCAAAAGT
TGTCCCATGCCAATGACTGCTTGATGGTATTGCACTGCTTTATAAAATTTTTTTCTTCTAA
TTACTCTATTTTTTTCATTTAATGCGTCCACAAACGTGTCCCCCACGTGTTCAAATCGAA
GTCCCAAATGTTCCAAAGTTGCTAACATGACAAACGATATAAATCCTGCCGTTTGGTAG

ATCACGAACACGGAGTAACACTCATATAAAAACACAAGCTGTTGAACAGGAAAATAAT
TAAACCGAAAAGGCATCCAGGTTGGAGCTGGTAAACCACACACTTCATGCCAGTTATT
TTCTAAATTTTCTTGGTGACATTGAGGTATGTATAATAAAGTCGCATTAAGCATAGTAAG
TATGATGACAGTATGGTATATTATGTAAATTTTCGACAATTTGTTTATGTAGTTGTTTCTTT
TAGTAAAATTGTTGGGTTGTCCAAAAGTTGTAAAATCTGAAAATTGTTTAAATAAAATA
ATAAGTTTTTTAAGATTTAATAAAAATCTAAACACAAAGTAAGATGCTCCGTGATACGAT
ATGATATTGCCGATATCCTTATCAAGTTTGGCATAAATGCCATTTTTTATATTAAATGTCAT
ATGCAATGCAGCTCCTATAGAAAAATATATAGAACTTAAATTAACTATTAAATATTTGTAA
TAAATGTTAGCCTTAGGATCATTCATTTCGCTGGTAGAAGGAAAAAGATACGGTATACG
CAACAAACGTATCGAAAATTGCATTATTTAATTTCTTTAATGTTTTCTATATTCATTTTGT
CCAAGGAAAAAAATCTCCCTTTACAAATTTCCTGAAAGTTGCTGCTGCCTTCAATTATC
G

>Cluster-12689.39599 MsigOR36

GCGGTTCTTCCCGAAGAGCCAGTATTTTTCATTCTTCTGACCGCGGAAACCTTTCCGAA
CATTTCATATATATATATATATAGGTATATATACCCTACTTTTTAATATATGCTTACATTTAGA
TAAGATAATCTAAAAATATTGTATTAAGCTAGCCATGTTCATTGAATATTGAGTGGCTCA
AACTGGCCATTGCTGTCATGACGAAATTATGTTATACGCCAATTAAGTGTCAGAAATGT
CCTTTATAATTGACATGATAAAGTAAATCGAAAGCAGGCTATAATCATATGGCGTCAAGA
AAATCGCTATCTAGTCAAAAGTTGTAGTTTCTGCTGCTACAATTACAATGTATCTACATA
TTCTTCTAGACTAACTTATAACAATAATATCTGGTTTGAGACGTAATATTCTACCATTTTT
TGCTGTTTATGTTCATTTTTTATTCATAAAAGTATAAACAGAATATACCGATTTGAAAACA
TTAATATAGCAATTACGATCCATCTCAAATATTCCTCCTGCTCTTATGGATATGTCTTTTAG
GCACATTTGCATAATCATTATTAAACATTTCTTGGATTTAATATTTCCGTAAATTACCATAT
TAGAACTAGCAATGATTTCTGCAGAATTTCTAGACTCATCTCGCACAAGGCTTACTGGA
ATACAATAAAGCGTCAACTGGTTACACATTGATAGCATGTAACATACTGCTTTAACTTTC
AAATTAAATGCTTGCTGACGATCAAGTACAACCATCGTCTGTATGCAAATTATAAACATA
GACGTGAAATATGTTACAAAATAATGAATTTTAAAACATTTCTTGATACGCTGAATGTAC
CTTAAAAGAAAATTATGATAATCTACACATTTCTTTATATCTTCGAAAGTGGTCATTTCA
GAAAAAGATTTATTCAAAATTTTAAACTGAAAAGATAATTCTATAAATATTAGTCCCATA
ATACCATCGAGACAGAATATCGCGATAGTCGCATGACTGCAGGTCCAAATCTCCATGAA
ACTTATCAAAATAAACCAGATTTGTCCATTTGGAATCCAACAAATATATGGTAGAACAC
CGTACAGAAACGGTTTACTCGACTGAGTTATGAATGTTAGTACAAAAAACATTAGCATA
ACACGAAAATAGTTCCACATAAATTTGTATATTCTTGTTGCCTTGTTGACTAAATCAGTT
TGTTGATAGTTCCAAAATTCTTTCCGCATTTCGAAGATTTTGACAATATCATCATTGAAT
ATTATCATGGTTGTATACTCGGAAACAACCAATACGTAAGTAGACATTGGTTCGCAAAA
ATTGATGAAAAAGCCCAAGTTCAACTCGGAGGTAAATAAATTTGTTAGAATTAAAAGC
AAAAGAGATACAGAAAATGTGAAGATCGAAGTTAAAATTATTTTATCAAGCATATTTTG

GTTTTTAACCAAACCAATCCCATGTAAAAACGTTCGAAGCAATTTAATAATGTCTGATC
CTAACATATTGACCAAATAGTATAGTCACAACTATTTATTTAAAATGTCGATATCTAAAGG
AAAACCGAATATTTTAAGTAATAGGGTAGAAGACTTATACACGTGCATA
>Cluster-12689.40380 MsigOR37
AAAAGAGTGACTCAGAAATAACTGAATTGTTATAGGAGATACTCATCTTTTAAAACAAT
CCTATTTTTGTTTGACGATTGTTTAATAACGGCAAACTTGTGACAACAACCAAACTTTA
GAGTTCAACCTATAGTGCACAAAATAAAATAGAATTTGAATCTTAAAATGAGTCACCAT
CAAATGAAATTTATCAAACCCTTCATGGTACTGGCAGGAATATGGCCATTAAAAATAAC
AGGCATAAAGTTATTCTTGTATAATGCTTACTTTGTAATTTCGTTTACTTACTTTTGCTTA
AATATTGCTACAATTTATGTTACAGCCAACCAGTTAGCCGCTCATGGGAGACCTACCGA
AATAACGAATTACATTTCTAATGCCGTTTTCGCAACGGTCATGATATACAAAGTTCTGGT
ATTCAGGTCTAGCGGAATGAAAGAATTATTAGAAATGATAGACAAGTACGAGAAAAGT
ATTTTTAATGCGTTCGATGAGGAACAAGAAAAAATTTATAAAATGTACGGTAACTGCGA
TTTCTTGGGTAGAACTTATTTGTTTTCAAGTATATTTCTGTGCTTCTGTGTCCAGCTCCTA
CCGCTGATAGGAACTCTCACAAGCAAACCAAATGAAGACGGTTACAGAGAAAAATATT
ATATAGTAACCACTTGGGAGCCGTTCGACAAATACAAATACTATTTCACAACTTATTTCG
TTCAATTGGCATTTACTGTGTTCGCAGAAATGTACGTTACTTTTTGCGGCACATTATTTC
TGTACATTCTTAAAAACGTAATCGGACAGTTGTTTGTCCTGCAACATCGGTTTGGAAGC
ATATTAAAAAACGCCCGCAAAATGTGCGACGGGCATGAAATTACGTTCAAAGAAGCTT
GCAGTTATTTGATGAAGGATGCTATCCGACAACACCAAGAAATTATTAAATTGGTCGAA
CATATTGACAAAACTTTTAAAGGATATATTTTCTTTGAATATTTATGCACGTCTTTTGGTT
TATCGCTAATTGTACTCCAATTTTTACTTGAAGAAACTGTCCAAATGAAAATTGCAGCA
GCGGGATATTTCTGTGTATTTGCTGGACAGTCATTTTTAATATATTATCAAGTGGAAGCA
GTAACAATGCAGAGTAGCACTGGATTAGCCACTGCAATATTTTCAAGCGATTGGTATGA
TTTAGATGCTGATGCTCAAGAATTGTTGCCATTCATTATTTTAAGAACACAAAAGCCCCT
AACTTTATCTATAGGATCTTTTGGAAAAGTTGGTATCAAGTCAATCATTGCTCTGTTTAA
AGCGACCTATTCTTTCCTCTCTGTTATGTGGTAAAAATGAACTTGACAGGGAACCGGTT
AACGACAAAACTAAAATAAGAAGAACCTTCGCACCGCACTTGCTGCATCGCACCGCAT
TTGCCGCTTTGTAGAAGAATACACAGAAGAATAGGACTGTAGAGAGATATATTTACAGT
GTAGAAAACATGTCTGAATAAAAATTGAGCATCTAAAAA
>Cluster-12689.40624 MsigOR38
GACGCCTGAAAAAAAATAGAAACCACCAATCCCAGGAAAGTCATTGTCTATCCTAAGG
GCCTTATCCTGGGAACATCTTAGACTAGGGTATGCACAATAGATCTATGCAGGTCGCGA
TGCAAAGAAGCTCTATAGCTTATGCTACCCACGCTAATATCTAATATCTATTATTGAATCT
ATAGATGGCGTTCTTTTTACGCCGCAGGGACCACTTAAGGGTTAAAGTGCTAATCATGT
ATGCGGATTATTTTACAAATAATGTTGACAGAAGAGTGTAAAATGAGAAGGACAGCCT
GAGAACCTTAATATAAGTAGAATAATCGAGAGGGATAAGTTTTAACGCAGTAATTTGGG

TAGCAATCTGGGCGTTTCGCATCAACATAACCATATCCTTTTTAATTTTATATTATCTATC
TCGTACCAGTTACTATTAAAGATATGATCCGTTAGGTTGTCGGACTCAGCGAGTAATTCG
TTTCCAGACGCGCAATATAAATAAAGCTGAAATAAGTGTCCTGTTAGATAACAACTCAT
TGTAAATGCTTGTAATTTATTAATATTCTCTTGCGTAGATATAAATCCCGAAACGCATATT
GCAACCATGCTGCTACACAATTGTAGGGCGGCTGCTAAGTTAAACACATTTTCAACACT
CTTGGTTGTTCTAGACAAAAGCTGATGGTGTTTAATAAGTCGTACAAAGAAATCCTGAT
TTTCATCATATATTTTGAATTTTCGCTTGTTCGGTCGATTGTCCAGTATTTGTAATTTTTG
GTTAAAGTACTTTCCTTGTTCTGTACCAAATTCCCAAAAAACTCGTTTCATTATAGCAAA
CTGGGTAACTAAATTGGAGCAAAAAGCCATAAATAGGAAATCGAATCCAAGAACTGCT
AAATTGATGAGGTAACCATTTACATAAACATGAATAATATAAATTATTTCGTGAGTGGGA
CTAGCGGTATAATCAAAAGGAAATACGGATCTAAAGGGCAGGTCGTGATTCTTCAACC
AAAGCGGTAAAGTCAAACTGCTCGTAGAAAACATCAACGAAAAATACGTAAGCATTAT
CATCGCACCTAACATTATGCGATAATTTGTCCTGAATTTCTCGGTATATTCTTGTACCAAA
TCGTCGGGCCAAAATTCTACTGAAATATCTTGTAGTATTTTTCCTAACGATCTTGAATGG
TACAAGAGGATGGTCAATTTTAGCGTAGCCTGAAAAATTGTAGACATTGACGAAATGA
CGGTTAAACTTTTGTTGTAATCTTGAAAATTAATATAAAAATAACGCACCAATCCCCATT
GTACAAAAACAAACACTGCCAAGGAAAATATGTAAAACAATTTTAGGTTTCTTAAGTT
CTTTTGTGGCCACATGAAAATTAATTTTAACATCAGCTCCGGCAAAAAAACTATTCGTT
TTGAATAATCCTTGGCATTCCTGAAACTGAGGGTATCCTCTTGAGGAAT
>Cluster-12689.40977 MsigOR39
GCAATGGGTTCTGTAACTCTCGACTTCGTGGCGTACTATAAATCGAATTTAAACGCCCT
GAAGTACCTGGGCATTTGGTCCCAAGATGTTAAAGTCAAATTTAAATCCATATATTACGC
ATATTGTTCGGCAGTAAGTGTAGTATTCGTATTAACATTTAGCATGGGACAAGTTGCCAA
TATGATCGATCACAGAGATGATCTCAATACGGTTGCAAATTCGTGCTACACGTTCTCTAC
TTGCTATATGGCGATAGTCAAATCCTACTATATGTACAAAAATCGACATGCTTTTCATAA
GCTTCAAAACTTGATAAACAAACCTATTCTACAACCTATTACTGAAGCAGACGTTTCAA
TTGCGGTTGAAGCTCTACGTATTTATACTGTCTTAAAAATAACTATCGAAGTTTTGGGGA
ACGTATTAATGTTGCTGTTCATGATAAGTCCTTTGATTTATGGAAGCTATATGGAAACTTT
TCCGCTTGAAGTCTGGTATCCTTTCAGTGTGACCGAACCTTCTGTACGAGCGGTTGTCT
ATGTACACCAATGTATATCAATCTATTACATCGGTTGCATATACATGTATATTGATTTGATC
ATTTTCGGATTGTTAATTTATATTGGGCTGCAATGTGATCTTTTATGTAATAATATGATCAA
TATCAAATTTATTAGCGGTTATGAACTAAAGAAGATAATAAAACGTCATGATGAGCTGGT
GAGATTTGCAAAAACAATTCAAGATATGTTTAATGAAATATTTCTTTTTCAACTGAGTGC
AACCGTTTTAGGAATGTGTATGAGTATGTTTTTGCTAGCCACGAGGGAAAACACATCAG
TCGACACCATATCCCTTTTGCTTTACCAGATGACCATTTGGATCTTACTCTTATTACCATG
TTGGTTTTCAACAAATGTTAGAGATAAGAGTGAATTAATTCCTCTGGCTATTTATTCGAT
GCCATGGTGGGACTGTAATTCCAACAATATTAAAAAGGATTTAATTCGCTTTATACATGC

TACTCAGAAGCCGATCATTTTTACTACTGCAGGAATATTTACGATTTCCGCCGAAACATT
TCTTAAAATTCTTCGTAGCTCTTTCTCTTTCTATACATTATTAATAAGCATGAACACGAAA
GGGGAGCCCACAATTTAAACCGGTGTATTAAAATATTGCCCGAAGATATCACCACTGTT
AGTATATTTTTTTTTAGATTTTTATGTTAGTCTTAATTTTGAACGAAAAAAGGATATCAA
GAGAAGTGCAAAACAGGCAGAATATTGACGTTCTTTATCAATATTGTAATAATGTATATA
ACGTTTCTTTAATTTATTAGAATCCAACGCTTTCTATGACAAGTCACAATCACAGCTATT
ACAAAATGGTCGAGAAATACTAAATCTATATTAAATCAAATTTATAACCAATTTATGTTTT
CCCAGTCTAAATTTTATATATATTTGAATTCAAAGTTCTGATAAGACCTCATTGTTGAAAT
TTATGGAATGATTCTGGCATATCATAAAAAGAAATATACATATCTTATTTGAAAATTTTAT
CTTCTCGTAGTAAACTGAAAGTATTTCTCCTTTATATTTTCAATTTTTTATAGATCCCAAT
TTTCTATTGTATTTTAAACACTACTACACTTACATCAAATAGCTGTCGCATTTATTTTAGA
GAATAAAGGTAACAAGCAACTCAAAA
>Cluster-12689.45629 MsigOR40
ATTGCATTCAACAAGATCTCTAACGTTCTATTAGTGTAATTAATCATTTAAACTTAACCTA
AATTTTTTGAAATCTTAGTCCCACAGTGGTCATTTAGTAAAATACACATCAAAAATCTG
GTAAAACATGGACAAACTGGATGATATCGGGTTTAAGGTATTGGGGACGAGAGATTATT
GTAAACTATTTCTATGGATTCCAAAAGTGGTTTTGTATTCTATTTGTATGTGGCCCGGAG
GCACTTACTGTCCAAGATACATTTTTTTCGCTCTTGCAGAAATATTAATTTTGTTAGTCG
GTGCTGGCGTAACTATGCAATGTATATTCATATTCAGAGATGTTACTGACATTTTTAAAG
AAATAATTTATGGAGTTGGGTTTATATCGGGTATGCTTAAATTCCTTGTACTTTTCAATAA
CCGAAAACTACAAGTGATCGTTGAAGACATCATGACTAAATTCTGGCCGAGTGATTTAG
TAAACAACGAGGAAGTGAAAAAGGAGTTGAAAACATTTTACTCTACCATAACATGGAT
TTTGGTATACCTTTCACTAACATTTGTGTTTAATGTAATTCAAGATGGTTTCATTGCACCA
TTGAAATTCGGAATACACCCTATTACAGTGTATGTGCCACTGATTAATTATCAAGTCAGT
CCGTATTTTGAAATTATTTATTTAGTGGAGATCGTCACAGTTTATTTTTTTTGCGATTTCG
CAATTATCGGTTTTGACTTCTGTTTCTTGGCTATGTGCTCCTGTGTCGTATTCCAATATAA
ACTCTTCTGCAACGTCCTTCTTACATTCAACACGCCGGAAATGGTTGAAGTCAATGCTA
AACTGAGAGAGATCGACAGAAGAAATAGAAAAAATTTTAACAATAAGTACAAAACCG
TACACGACGAGTTTTTTGCAAGATGCGTTCAACATCACCAGTTATTATTAAGGACCGTA
GACCACATAAATATCATGTGTAGTGTTGTGGAATTGCTACAGATAGTATTCACTATAACA
GGAGTTTGTTCAGGGCTGTTCATTCTGACAACACTGGATAATCTGGATTTTGCCAAAGC
ATATTTCGTAGAATCAATCATTGTTTTCGTTCTAAATCAACTGTTTTTGTACTGCGCAATT
GGCAATGAGTTAAGTTATGAGGCGAGTCTGTTACCAGAGTTTATCTTCAAGGCCAACTG
GCATCAGATGGAAGATATGAAATTTGCCAAGAATTTTATGGTCATGCTACGCCGGTCCC
AAAAAATTCCTCAAATAACCTCTTATGGTTTATATCACTTGAATTTGGACTTCTATGTGC
GAGTTCTTAAGTTTACATTTTCTATATACACGTTTTTAACAACTATGCAACAGAAAGCTC
ATCATTAAAGCAGCCGATACATGCGAGATTCGGTTCACTGATATACGAAATGGATCCAA

AAACGAAACAAGAATACTTCATCAATATATTTTTTATTAGTAGAAACCGCAAAGCAACT
ATGTAACCGATCATTAGATACTTTAGAAACGCCAGACATTTCGAATGTTCGAATGCTTTA
TATAGCGAAGAAAAAGTAATCAACGCATTTTATCCAAATTGTTTTTAAACAAAAACAGA
TCACTACAGACAAGATCGGGATTGTTTGATAGAGAGTCATCGTTTAGTGGACACAAAAT
TTTTGTGAAAATTGAAAAGTTGTCCTCCATAAATATTCTGCTATATACTTTTCTAATTCAA
ATATTTTTTGACTGTTTCTAGCCAGTTATCTTATACTTATCGTCAAATAACAATCATTATTT
GTTTTACTGAAATCCCTTTCTTATCGTTTCCTTTTTCTTTCAACTCTAAAATTTTTGAAGC
TACGGTCGGTTTTTCACATTTCATCAAAGCTATTATATAGACTTTAATGAAGGTTGTTTTT
TTCGCATGGAACGACGTTTTGTTTTTCTCTTTTCAATCCCTTTTTTGTCTGCAGAAAAAG
TAATTGTAAGTTATTACTTTTTTTTGTTTCATTTTACTGTTATACAAAAAAAGTTCTTCTT
TTTCCACAATGTTTCACTGTCTCGAAGGGATAAGCTACATCAATTAATTTTTCTTCAACT
GCTATTACTACTGCCACTGATACTTCTGCACTAGAATACCACCCACATAATAAATGTCAA
GAGCTGAGTAGCTAAAGTTAATAAATTTAGCCTCAAGAATTAGGTTCTCTAATGCAGTA
ACAGGAAGAGTGATTTCTATTAAATATTCGTCTATACCGATTTGAGAAATTTGCGTAGAT
GATGCTACGGAATATATATAGAAAACTGAAATGAACTAAAACGTTCGGTTGACTCAACA
ATGGAGGAATATACAACTTACCTTTTGAAGAACTTAATCAGCAAACAACGTTTATGTGA
GCGACTTTATATCAATGCATTACACAATTACGTGCTTCAATGTTTTTTATGTACTATTGTT
TTTATTAGTTTTGCTTTAATAATAGATGCAATAAAAAGTTACAAAGAAA

>Cluster-12689.50120 MsigOR41

AGATGAACATATTTTAGTCATTAAAATCAATGTGTTCTTGTCACTAATATTTCAGTTATTT
TGCAAGAAAATATTTTTCCAGATATTGATATACCGTTTGAGGTTTCGTTACAATTTATGC
AACGGGGTTTAATTTTCCCAAAAACGAAGCAGTCTTTAAGATATATCCAAACTATTTTG
CTTAGGGTTGCCTTTTTTATGGTTGTTATAGCATGTGTAGCATATTTTATTAAGGCAAAAA
TAGGGATATATTCATCAAATCATTTTTGGCCTTCGTTGACTAAATCGGAAAGAAAAATAT
CGAAAAGTTATAAAGATTCCTGTTAGCAGAGGTTTTTTTTCCGATTGATATAAAAATAG
AACAAGACATTTATTCTATTAATAAATTCCAGAAAATGTATCAAATTCAGAGACATCAAC
CATTTTATGAAACTTTGAAAACTCTACATGTTTCTTTAGTGTATCCTACAAAAAATGACA
AAAACCATTTAAAACGATTTTTTTTAATGAGTTTTTTACTTCGATGTCCCATTTGGATTG
ACATTTTATTAGTTGTGGTGCATTATTTTCAAGTCAAAAAAGCCAAAATTTCCATGGATT
TTAGCGAAAATTTCTTTGTATTAATTGGAGGAGGAGCGCTTGTTATAGCATGTAACATTT
TGCCGTTCACTGCGACAGAATGGTCAAAACTTTTAACGAATGTTACACAATTTAAATTT
TTTGGAGAACCTAATGAGCTAAAAAAGTGCATAAAACTTTGGAACAGACTGTCAGTTA
AATTGCGTTATTATTGCATTTGTGGAGGTACATTTTATGCACTCCAAGGATATTTAGAATC
AAAAAAATGTCTAATGATTAAAGAAGAATACGACGTACACATAGTATGCCTTGCCTACT
TTCCTATTTGGTTACCTTTTGAGCTTAATACAGGAACTAATTGGATTATTTTTATTTGTCA
AATGATTACTGTTTTCTTTGTAACGTTGCCAGGAGCTTTAACCTGCTTTCTTATATGGGA
AGTTACCAACATGCTACTCTTACATTTTCGTCGACTTAAATATGTGTTTTCGACAATTATT

GTGGAACCTGATGAAAAGAAAAGGAAGCAACAAATTAGGTTATGGTTATTTTACCAGT
TGCATATTTTCAGGATGGTACGACGCTTAAACATTTTATCGAAGAAGTGTATGGGTATAT
ACTCACTCGTTGCAGCTTCAATATTTGGATGCATTTCATATCAGCTGGTCAGTGGGCATA
AACTTATTGGAGCAGCTATCATATTTTTTGGTTATTTCGTATCTCTCTGGATGTTATGTGA
TGCAGGTCAGAAAATACGAGATGAGAGCGACTCCGTTACTGAAGCAATATATAATTCAC
CATGGTACACAATCGACACCAAAACGAAAAAGGACATTCTATTTATGATGATGAGCAA
CTCAATCAATATCCACTTGGATGCTTTACCACTGGGTCACATGAATAATGCATTATTGGT
TATGATCATGAAAGGAGCATATTCATATCTAACATTATTAAATAAAAGTAGTAACAAGAC
AATGTAATATTAATTTACTGTGTAACATTAAAAGTTGTATTTCTCTTTAAAA
>Cluster-12689.50988 MsigOR42
GCCAACTTCAAAACCTTCAGGAATGATTCCATGTTTAAATCATAAACACCATAAGCGCT
GAGTTGTGGAATTCTTTGCGAGTGTTGTATCATAAATATTACATCTTTTTGAAACTTGAT
GTCATCTATTTCCTTCCAGTTTGATTTAAAACTATAATCCGGTAGCAAACTGGCTTGATA
ATTCATTTCACAACCAATGAAACAATAGATAAATAACTGACCTAGGAAACCGCATCCAA
AACAAAATTGGTCAAAAGTAATACTGCCCTCCAAAATAACTCCAGTTAATACCGCTCCT
AGTGCAACCATACTGCTTAATAGTTGAGCAATCATGGCTGGACTAAATACGTCATTTATT
TTCTTGGTAAACCTTATAAGTAGTTGATGATGTTTGCAGCATTTCACAAAATATTTTTTC
CTTTCATCATAATGCGAATGTTCTGCATACTCTGACTGTTTAAATTTGCTGATGATTAGCT
GCATTTCTGGTGTGTTTAGAGATGAAAAACATTTTTGCAAAATACGATATTGAACTGTT
ACAGAAGCTCCGGCTCCCATCAACAAAAAATCACTTCCCAAAGTGAAAGTACACACA
CACACACCAAACGCAATTATTTGTGAAGAATAGACCAGTTCGTACAATGGGGTTACCTT
CCAATCAAATGAATACTGAACAGGAAGAGGTGTTTCTCTACCTGACATTAGGAAGGGT
CGTAAAAATATAAGAAGTGTAAACATTACAGGCATTGAAAGTTCACAAATCATTAGAAG
AATAATAACTAAATAAAACATTTTCAACTGCTGTTCCAATTTTCTTCCTGTCATATTATAA
GGCCAGAACTTAAAAAGCACATCATCAAAAAGCTCTTTTATTACCGGTGCCTTGTAGCA
CAATAGAATGGATTTATGGGATGTTTGAAAAAAACCCGTAATCAGCACTATGTTGCACA
TATTTTTTCCGATACCGCCTGCAGCGATATTCACAGATTGGGCGAGCCCAAAAGTTACA
AAAGCTATTGTTGAGATTCCAAAGCAACAAAATGTAATTGATCTTAAACGAAAATGTGC
GGTATATGGCCACATTTG
>Cluster-12689.51760 MsigOR43
CGCGCCTCTATCTCTATTTATTTTCAACTTTGCTCTTGCAATCGCGGTGTTTAGAATTTAT
TCAACTTAAGTTTGCTTATATGTATACAGAGTATCGTATTGAGAAAGGAAAATATTTAAA
CATATAATGGAAGTCACCGAGGTAGTTGTAAAAATACTTCATTTTTTGGGCTTAAATATC
TTCGAAACCTAAATATTCAGTCTGCTTAAACTTTTGGTTATGTCAATTTAAATTATGCAC
ATAGAACTTGTAAGGAAATAAAAAACCATTCAAATTTAATTTGACAGTCTCGTTTAAAT
GTTTTAAAATGTGAAAGAGTAGGTATTTATAATTCTAATTTAAAAATATTTTGGATAAAAT
GGTTTATAGTTTTTCGCTTAGTAAGTTGCTAACGGTTGTAAATTTAAGCCTACAAAGTAA

TTTCGACTAAGGTTTATGCATATAATAAGCTTTTTAAATTCATTATTACTAGATTGTTTAA
CATTTATTTAAAGGAGAAATAATGTAATTTATAATTATTTAAAAAAGTTTTAAATAAATAT
TTTTTTTAAATAATAATAAAATTATCCGCGTTAAAATGTATATGATTGACATCATTTTAATT
GAACATTTTGGATAGGTAGGGTGTTGTTTCGAAGGTATAATGGTTTCTAATAAATAGAG
AATTTTTAAACAACTTTAGTGACATCGACAAGTTATTTAAATATTTATATTTATTTTCCAA
CGAAAGATACTCTATAATACAAACAAATTGCAATGTGCGTTTTTTTGCTATTTAATGAAA
ATGAAATAACCAACTATAATTTTGTAGTGCTTTACAAATACGAATAAATTTTTTACTCTA
GATTTTTTAACTACTTTGCAGATATTTTTGACAATACTGTAAACATGGAAAACGCCATTT
TCAAAACCTTTGCATATGAGTCCATATTTATGTCATAAAGACCATAAGCGCTAAGTTGGT
TAATTCTTTGAGAATGCTGTATTATAAACATTACGTCTTTTTGCAACTGGTGATCTAAAG
TCCTCCAATTTGATGCATATATGCAATTAGGTAATTGACTGGCCTGATAATTTAATTCGTT
GCCAATAATACAGTAAAAGAACAATTGTTCCAAAAAAGCACATGAAAAAAACAGTTG
ATCAGGTGTAAAGCTACCCAATAAAATTATGTAAGTTCCAAGAGTTGATAGCGTAATTAT
GCTGCAAAATAGTTGAAGAAGGATTACTGAATTAAAAATATCATTAATTTGCTTTGTAA
ACCTTATAAGTAGTAAGTGGTGTTTCCAACATTGTAAAAGATATACTTGTGTATTATTATA
CTTCACTGATTCAACTTTATTCAAATGTTTTAACTTAATATTGATAGCTTGCATTTCAGGG
GTATTAAATACAGAAAAACACTTTTGCAAAATTCGATATTGAGTCACTGCACAAGCTAC
AATACCAAGAAAAACAGAATCTGTTCCCACAACATTAGGAATCATTACTATAAAGAACT
CAATTACTTGTAAAAGATAAACCAGGACGTACAGGCCAAAAGAATCGTCATCAAACGC
ATATTTTACAGGAAGAGGAAATTTTACAGTTTTTACTAATAGTGGTCGTAAAAATAATGT
GGCTCCAAAAACTATACCGATAATTACGAAAAGAAGTAATGGAAATAACAGAGCGTTAT
AGAAAGACTTTATATTATGTTTTAATTTGCCTTCTGTCAAATTATATGGCCAAAACTTTTC
AAAAATATCATTTAATAGCTCTTGAAGCGCAGATCTCTTGTTATATAGTATTAAAGCTTTA
CAGGCTGTCTGAAAAGTACTTATCAGAAAGACGATGTTATACATACTATTTTCTATACCT
AAAGTAAAATCCATAAATCCAACAAGTCCGACCGATAAAAAAACTGATAGTGAAATTG
CAAGGATACAATAAACTAATGACTTATAGCGATGATGTTTGCTGCTTGGCCAAATTTGA
CAAATTTGTAGTAGGATTTTTGGAATCCATAGAAACAGCTTTGCATAATCTCGAGTACCT
AGTACTTTATAACCGATATACATTGCTTATTAAAGTCTATTATACTCTTTTTTCACAGGTTT
CATCCAATATTATATTATACTTGTAGTATCTCGTACAGGTGGGGTCTTCGTACATCTTGC
>Cluster-12689.5356 MsigOR44
TGTGTATATCTTCTTTTCAGTCATATGTGTAAGACAAATCTAGTGAGTGGAAAATCACAA
AATGTTAAACGGTATACCGACTCGTATTTTAGGAACAACCGACTATTGCAAATTATTTTT
GTGGATTCCAAAATTCTTGTTGCAAACAATTCTTTCTTGGCCACGGCAAAAAGACACT
TTCTCTTCAAAAATATTTTTCATTTGGTATTTATTAGTGGGTTGTATTTCCTTTATTGGAAT
GACTTGCTATGGTGTTGTTGTAGCTGAAAATACTGAGGCACGTTGCAAGCAAATCCTTT
TTACTGTAGCTGTTGCTCAAATGGTGTTAAAATTGGTAGTCCTCCACCTCAACAATGCA
CGATTAAAAGAAATTTTGAACGACATATTTCTTAAATTTTGGCCGTACGATCTTTTAAAT

ACAGACGTACAAATTGAAATAAAAAAACTGTACACCTTTATTACGACGATTATGATTCT
ACTCTTCATAGTGGGTACTTCATACGACGGTTCTGTGGTATGGCCGATTATGATAAATGG
TCAGATTTCAGGACCAATGTTTTTCCCGTACATTGACCTGAATACGGAACCTTACTATCA
AATTGCGTACGTCGCACAAACTTCATCGATTATTTTGTATTATTTCGTGGTGTTCGGCTG
TGACTTCCTATTTTTAACCGTTTGCTCGTGCATGATTACCCAATACAAACTCTTGCAACA
CAGCCTCTTAGTGTTTAATACACCGGACATGGTGGAAGTTAACAAAAAGTTAAGGTTA
ATCGAAAATGATCGTTTAAGCGACAAGTATAATGACGTACAAAAAGAATTTTTTGTGAG
GTGTGTACATCACCATCAAATGCTATTAAGGATTACCAAACAAATGAACGTTGTGTTTA
GCTTCATAGAAATAGTGGAATTAACTTCCACCGTGATGTATATAAGCGCAGTTCTCTTCT
ATACGACATCTTTGATAGATCCAACTATTTCTGACGCAGTATTAATGATTATTTTAATGGT
TTATTATTTAAATCAGCTGTACTTGTATTGTGCTATAGGAAATGAGTTATATTATCAGGCG
GGGCTCCTACCCAAGTTTATCTTCGGAGCAAATTGGTATAATTTAAAAGAAAACGCAAT
GAAAAAGGATTTTATGTTTATGCTGAAACGCTCACAACGTACTCCACGGCTAAATGCTT
ATAATTTATATAATATCGATATCGAGTGTTACATTAGGGTATTGCTTTTCGTGTGGTGGGA
ACGCATGACTATTGCAAATTATTCTTATGGATACCAAAATTGTTGTTACAAACCGTTTTT
CTTTGGCCTCTGCAAAAAGATACTTTTCATAAGAAAACCATTTCATTAGTTGGCTCAGG
ATTAGTAGGTTGTATCTATTGTGTAGGATTAGTCTCTTATGGTGTATTCGTAAATCATGAT
ACTGAGGTTATTTGTAACCAATTAATGTATATTGTAGCCTTCGTCCAAATCTTTTCTAAAT
TCGTTGTCCTATACCTCAATAACACGCAATTAAAAAAAGTTTTGGACGATATATTTTTAA
AATTTTGGCCCTACGACCTTCTAAATGTCGATGTAGCAACTCGATTAAAAGAAATGTAC
ACGTTTATCATAAAAGTTAACTTCGGACTGTTTGCGTTGGGTTTGGTATACGACGGTTC
AGTTGTGTGGCCGCCTTTATTAACTGGACAGCTTTCAATGCCTGTTTTTTATCCGTACGT
CGATTATAACATTACACCGTATTATCAAATAGCGTACATCGCACAATTAACAACGCCACT
TCTGTATTACACTGCCGCAATGGGCTGCGACTTTTTATTTTTGGCAATGTGTTCGTGCGT
TATTACACAATACAAACTTTTACAACACAGCCTCTTGGAGTTTAATACACTGAACATGT
TGGAGGTTAATGGAAAATTAAGGATAATCGAAAATGATGGATTAACCAAAAAGTACAAT
GACGTTCCTAAAGAGTTTTTTGTGAGATGTGTACAACATCATCAAATGCTATTGAGAAT
CACTAAAAAAATTGACAGTTTGTTCAGTTCCATGGAAATAGTGGAAATTATAGCTGTCA
TAACGTATATATGTATAATTCTCTTTTTTATGACGTCATTGGCATATCCAACTACCATTCAA
ATTGTGCTGTTAGCTTCTGTATTGGCATATTTTTTAAATCAGCTCTTCGTATATTGCGCTAT
AGGAAATGAGTTATATTACCAGGCAAGTCTCTTACCCCAGTTTATTTTCGAGGCGAATT
GGCATAATTTGAAAGATGAAGGATTTAAAAAAGATTTTTTATTTATGCTGCAGCGTTCA
CAAGATGTTCCACAGTTAAGTGCCTATAATTTGTATAATATCAATATCGATTGTTACATAA
GGGTGGTCAAATTTTCATTTTCCCTGTACACGTTCCTCTCGAAAATGAAAGAAATGTAA
GTTATCTTAGAAGAGTAACTATGATATGTGAATTATGAAGCCCAAGTTTTTAAGCTATAG
CGAAT
>Cluster-12689.5679 MsigOR45

CGAGTTTTGACATTTTTAATAATGTTTAAAGACAACAATATACCATTTAACGTGTCACTA
AAAATCTTAAGGATGTTTTTAATTTTCCCTCCTAAGATACCAAATTTGAAGAAATTTTAC
ATAAAAAGTGGTATTATAAGAATTTTAGCGATTTTCGGTATTATCGTTGGATGTGTTGCTC
ATTTTGTTAAATATACATTAGACCACGAGAATGAGGTAGATAAGAGTGAAGATATACTTT
TAATTTCCTCTTGTTTTGGCACTATATGTGCTATGGGAATTACTACATATATGTATAAGGAT
TGGGAACAATTCTTGAATAATCTTGTAGATTTTTCGAAATTTGGTAAACCCGAAAACAT
GGACAACACAATAAAAAGAAATAATAAGGTGGCAAGAAATTTTACAATTTATTGTGTAT
CATCTCTATTAACTTATGGTTACATTACATACAGAGAACGACCAAAATGCCATAAAATTA
TGGAAGAGAAAAATATTAGTTTAATGTGCGATACATTTATACCTGTGTGGTATCCCATCA
AGGTTCCACAGTATGTTCAATATATAATGTTTGGGTTTCAGATGGTACTTGCAGACATTG
TTTACTTTCCTGCATTCATGCTTGTTGTTCTGTTTTATGAGTTAGTCCAAATTTTGAATGT
ACATTTCGAGCAATTAAAAAAAGATTTTCAAAAAATTGTTCACGAGAAAAATCTTAAA
ATCAGACGTGAAAGGTTTGCTTATTGGGCGAAATATCACATCAACATAATAAGAATAAC
AGAGGATTTTACAAGTATTGTAAAAAAGTCATTTTCTATCTTTGTGTGGATCACTGCATT
TGTGTTTGCTTGTGCCGAAAATATGTTAGTACGAGAAACGAAACAATTGGCAGCGTTG
ACATTTTTGGTGGGATGGAACGTAGCATCGGTTTTGGTATGTCATTGTGGTCAAGAAAT
TCATGAGTTTATGTTATCTCTTCATGATGCAATATATGCTTCTGAGTGGTACCTTACTGAT
AATCAAACAAGGAAAGATATTGTCTTTATATTACTAAGATGTCAAAAACCGCTTAATTTG
TCCGCTTGGCCGCATATCGTAATCGACTACGCTCTTTGGGTTTTGGTTTTAAAGACGTC
GTACTCATATCTAACGATTTTAGCTGCCTGAAAATAGAAATTTGGCTCCAACTCAAAAA
ACATTAGAGAACTCACTGCCTTAAATTACACCTATATTGTTCATAAATAAATAAATTTGA
C
>Cluster-12689.45922 MhieIR8a
GAGCAGAATGTGACCATATTGCTCTTTTATGGGATAATCCCATACTCGGTATCTGCCGTC
GTCCGTTGAAGCATTTAAGGTCAATTCTTTCCACATTTTATAAAGTATGTCTTCGGCATA
TTTCATGTTAATAAAGTACTTATGTGTTTGAGATGCCTTTACAACGGTATAGTTAATTCTT
GACTGCCTGGCTAGTTGCTCTAAGGACTGAACAGGAGCCTGCATTCTCTCGACAGTCA
AAAACGCAGCCAAATTTGCAGTAAAAGTTGCCAACATAAGTACTACAAACAACCAATA
TGCTGCCACCAAAGTCCTTCCAGACAATGCTTTGGGAGCTTCTCCACCTCCTTGGGGC
GTAAACGATGTTAGCGCAAACCAAAAACTTTCCTTAAGTGTAAATTCCCTGCAGGCGT
ATGGATATGCTTTCTTGTTATTTCTACCACTGTAAGGAGAATATTTATCTAAGAACCAAAT
CATAAGTCCAGTAACGATCAATGCGGCCACAATACTAAACCATACTTCTAGTTTTAACA
CTGTCATAAATTTAAATAGGGAGGTTTTTCTTACAGGTTTGCGCATTACAATAGTTATAC
CAGTTTGATCAAAGTAAGGAGCAACAAAATCAATTACTTCCTCTTTGTCAGCTGTCATA
ATTAGTGCTGTGATTGCCAGATCTGTCTTCCCGGAAACTAAATCGCCAACCACACCGTC
CCATATACCATTAATTTTTTTGCCGAAAGTCCCTTCTTTGGGTTCGATGAATTCAAATTC
AAAATTTAACATTTCCGCTATTTTTCTACCAAAATCAGCACAGTAGCCGGTCCATATCCA

TTGGCCGCTTTTTTCGTCCAATATTTTGTAGGCCCATGGCATTGCATATGTTATACCGATG
GTGTAAAATGCTTTAATAGGTTTAATCGTGGCATCTTGAGCTATACTGACAACTCCATTC
TCAACTTTTGCCACTGTATTATCATTGTCGCCGACTTGTATAAATCCATTCGTTTTAATTC
TTATTTTAGATGAATTAAACGATAGCATATTTTCATTCTCTATAGCATTGGTAAGACACTC
CTCTATAACTTTACTAGTTTGATGGGTAGCTTCACTTTCGTTACAAACAAAGCCTGGAA
ATTCTATTCCTTCTATTGTCATATCATCAATGCAACTCTTAATTAGACCAAATGCTTTACT
GAAAAACGATTTTCTCAGATCGAAATCGCTCGGGCAATCACAGTCGTTGTTTGAATTTA
AAAAATAACAGCATACTTCAGGGTTCATGCTGATCAAGTTCAACGGTTGATTGGCAATT
AAGGATTTGTCGAATTTTCTAGTCTGAAAATCTAAGAAAAGTAGATTCCATCGATCCGG
TAATTTCACTAAATTTTCTTGAAACGCCAATTTCAGTAGCTTGTTCATCTTGTCTGTAGA
TGCCAACAAAGCAAAGTTGTTTGGCATTGGTCGAATATCTCTCAACTTTCTAACCGTAC
CAGCGTTTAGTGTTTCTGTCATTAACATCCTCATGGTGGCGGAATTTAACCAGTAATAAA
GGGTTTGGTCAATATATGATGGATCTTCAAAGATGAATGTTACATCGGTTGCATTTCGTA
AATCCAAATATCTATCAAGAAGATCCAAAAATGGCGCAATCGACATATCGATTTTGATGT
AAGGAATTCCAATATCGGAAAAAAACGATTTGGCCTCTTCATTTTCACACCAACTGATG
TCCAAAACTATGTTTCCTCCTTGAGCTATAGCCTCACATACTTTGCCATTATCTTGCGAG
TCCTCAACATTTAAGGTAATGCTCTCTAGTTTGACTGGTAGAATAAGTTGATTGTACCAT
TCCAAAAATGACTCTTGGCCGCTTTTTTCCTTAATAAGAATTAATTTGGGATTGTCTTGA
GTTGCCCCAATAACAAAAAACGTCGTTACTAATAACCACCACATATTTC
>Cluster-12689.38096 MhieIR25a
TTTTAATTTTAAAACAATGTGAATCGCAAACCACACAAAATATTAATGTTCTCTTCGTAA
ATGAGGAAGGAAATGAAGTGGCCGATAAAGCTCTGGACGTTGCATTGACGTACTTAAA
AAAGAACAGTAAACTTGGCATCAGTGTAGATTTAAGAAGGGTCGTTGGAAACAGAAC
AGATTCGAATGCTTTCTTAGATTCACTATGCAGTACCTACAACACCATGTTGGAGAGCA
AAACGTATCCGCATTTAGTTTTGGACACAACCATGACCGGCTTAGGATCAGAAACCGC
CAAGTCCTTTACGGCAGCTTTGGCCTTACCGACAATAAGCGCTTCTTTTGGCCAGGAA
GGTGATCTGAGACAATGGAGAAACATAGACGACACGGAAAAGAGTTATTTGGTGCAG
ATAAGTCCACCGGCTGATCTGATACCCGAAATAGTGAGGACTATCGTCTTGAATCAGAA
TATTACGAACGCTGCTATTCTTTTTGATAACTCGTTCGTTATGGATCACAAATACAAATC
CCTTCTCCAAAACGTGGCTACCAGACATATTATTACTCCAATAAAAGACGGTAATGAAG
TTATAGAACAAATAAGTCAACTTCGAAAATTGGATTTGTTAAACTACTTTATTTTGGGAA
GCTTAACCAACATTAAGAGAGTTTTGGATGCAGCAGACACACTTAACTTTTTCAACAG
AAAATTTGCTTGGCATGCCATAACTCAAGACGAAGGTGAAGTGAAATGTGTATGTCGA
AATGCCACCATTTTGTTCGTAAAACCGCTACCAAATGCCACGTTTCAGGATAGATTGGG
AACGATGCAGAGGACGTACCAGTTGAATACCGAACCTATAATTGCTTCTGCATTTTACT
TTGACCTAGCTCTTCATTCTTTTATTGCTATAAAGGAACTAATCGCAGACGGTGTTTGGA
AAAATGCTGTTTCGAACTATATAACGTGCGACGAATATAACGGCAACAATGCTCCGAAG

AGAGTGGGACTGGATCTCAAAAAATACTTTAATAAGGATAATTCCGAGCCGTTCACTTA
CGGTCCCATATCTGTCGTTTCCAACGGTTTGAGTTATATGGATTTCCAATTGGTACTCTC
CTCCGTGGGCGTTCGAGAGGGAGCTTCCGACAAATCTCTCACACTTGGGACGTGGAA
CGCTGGCTTCGATAACAATTTGACATTAGTGGATCCCAGAGCGATGATCAATCTAACGG
CTGACGTTGTTTATAGGATAGTAGTTGTTGCGCAACAACCTTTCATCTTCAAAGACGAA
AAGGCACCAAAAAAATATAATGGCTACTGCATCGATCTCATTAACAAAATAGCAGAAAT
ACTAAAATTCGATTACGAATTAGTAGAAGTCGACAAATTCGGTAACATGGACGAGAATG
GAAAATGGGATGGTATGGTCAAAGAGCTTATTGAGAAAAGAGCGGATGTAGCTTTAGG
TTCGATGTCTGTTATGGCGGAAAGGGAAAACGTCATCGATTTTACGGTGCCATACTACG
ATTTGGTGGGGATAACCATTTTAATGAAATTACCGGAAACTCCTACCAGTTTGTTTAAAT
TTTTGACTGTACTGGAAAATGACGTTTGGCTATGTATTTTGGCTGCTTACTTCTTTACTA
GTTTCTTAATGTGGGTCTTCGACCGTTGGTCACCTTACAGCTACCAAAACAATCGAGAA
AAATATAAGGACGACGAAGAAAAAAGAGAATTCAATCTCAAAGAGTGCCTTTGG
TTCTGTATGACTTCTTTGACACCCCAAGGAGGAGGAGAGGCGCCCAAAAATTTGTCCG
GTCGTCTTGTGGCAGCAACTTGGTGGTTATTTGGATTCATCATAATCGCCTCGTACACCG
CCAATCTGGCTGCTTTCTTGACGGTCTCCAGGTTGGATACCCCTATCGAATCTCTGGAC
GATCTATCGAAACAATACAAGATCCAATACGCCCCACTGAATGGATCTTCCGTTCAGAC
GTACTTTGAGAGAATGGCCAATATAGAGGCGAGATTTTACGAAATATGGAAGGACATGA
GCCTCAACGACAGTCTGTCAGAAGTTGAAAGATCAAAGTTAGCCGTATGGGACTATCC
AGTCAGCGACAAGTATACGAAAATGTGGCAAGCTATGAAAGAAGCAGGTCTGCCGAA
CAGTTTGGACGAAGCCGTGCAACGAGTTCGCACTTCAAAGTCATCCAGCGAAGGATTC
GCGTATCTAGGCGACGCCACTGATATCAGATATTTGGAAAATACGAATTGTGATCTTACT
ACTGTCGGCGAAGAATTTTCCAGGAAGCCTTACGCCATCGGCGTCCAACAAGGATCAC
CTTTGAAGGATCAGTTTAATACTGCAATTTTGCAACTGCTGAACAGGCGCGAATTGGA
GCGTCTAAAAGAAAAATGGTGGAACAAGAATCC

>Cluster-12689.12561 MhieIR40a

CAAATCTATGGCATCCTAGATTCAGAGTCAGATCGTATCAAAGATTATTTTTGATTGTA
CGTGGTTTACGATAACCATTTTATTAAAACAGACTGGTCGTGAACCCAAATCTAACAAT
AAAACAAGATTCTTTATTATTCTTTTATCATTTTCCGTAACTTACGTAATAAGCGACATGT
ACTCAGCAAACCTAACGTCATTATTAGCCAGACCAGGGAGAGAAAAAGCAATTCACAA
TCTGTACCAGCTGGAAAACGCTATGGTGTCAAGAGGTTATAGACTATATATTGAAAAAC
ATAGTTCTTCACACGCACTTTTAGAGAATGGCACTGGAATATATTCGAAATTGTATGATT
TAATGATTCTACGACAAGGATATAACAACGTATTGGTCGATTCAGTTGAAGCAGGAGTT
AAGTTGGTGCGAGAAGAAAGGAAAGTTGCGGTAATGGCTGGTAGAGAAACTTTGTTT
TTTGACATTCAACGATTTGGTCCTAGTAATTTTCATCTAAGTGAAAAATTAAATACGGCC
TATTCTGCTATAGCTCTACAACTGGGTTGTCCCTACATAGAAGAAATTAACAAAATCTTA
ATGGCCATTTTTGAAGCTGGAATAATTACAAAGATGACTGAAAACGAATACGAAAAAC

TTGGAAAAGAAAAAGCACTTTCTTCATCAGAAATAGCTGAAAATGTGGCGAAAGAAA
CCAATAAAGATTTAAAACGGCAGATTAAAGTAGAAGAGGAAAATGACAAATTAAAACC
GATTAGCCTGAAAATGTTACAAGGAGCTTTTTATCTATTATCCTTTGGAAATGTATTCTCT
GGATTTATTTTAATTGGAGAATTGATGTTTTATAAGCATAAACTCCGAAATAGAGCAAAA
AGACGAAACAGAAGATTTATTTCTAAAAGATGGGAAAAAGTTGTTAAATTGAAAATGA
ACAGAGTTCGTCTGATGATGAGACGATTTTACAGGAATGCGATGCACGAGGCCTTTGTT
TCTACGTTAGAATATATAGAATAGATTTAGTTTTTAATTAAAATTCAA
>Cluster-12689.22510 MsigGluR
ACACAGGATGCGTAAATGTTTTGTGTTGCTTCAGGAATGATTTTCTCCAGAAAAAGTTT
ACTTTTTATCAGGATATTAATATTTTGTGCTAATTATGGTGTTTCCAGTGCAGAAGAATAT
AAAATAGGTGCCATTTTCGATGAACCGCACACCAAACAAGAAGCGGCGCTCCAAGAA
GCTGTTATTCAAATCAATGAAAATGACGATGACATCGATCTGGAACTGATAGTAGAACA
TATTCCCCGGGATAATCCCTATTACGCAGCAAAAGCTACATGCAGTCTCTTGGAACAGG
GCGTCGTCGGGATACTAGGTCCATTATCAGAGGACAACTCCAATACCGTGCAATCAATC
TGTGATTTAAAAGAAATACCTCACATTGAGGTAAGATGGGACGATTATCCTACAAATGG
TACCTTAATTAACCTTCATCCCTACCCCGATTCACTCACCAAGACTTATTACGATCTCATT
GTGGCATGGGGATGGAAAGACTTCGTCATTTTGTATGAAAACAATGAAAGTCTACAAA
GAGTAGGAGAGTTGTTAAAACTTTTCGATCCTTCTTCTCACAGAATTGTTGTAAGGCAG
TTGAACGCAGCTCTATCGGGAGGAAATTTTAGACCCATTCTGAAGGAAGTGTGGCGAT
CTGGGGCTACGCATTTCGTTCTAGATTGCAGCATCGAGATATTGGAAGATGTGTTAAGG
CAGGCGCAGCAAGTAGGTCTTATGACCAACAAGCACAACTTCATTATTACGAATCTCG
ATTTGCACACAATCGAACTAATGCCGTATCAGTACAGTGAAACTAACATTACCGGGATG
CGGTTTGTGGATCCTGATGCCGAAGATCTTGCTGAGATTGCCAACAAAATTTATAAACA
AAATTTACAAATTTTCACGGGTTATAATTTAAAATTGGAGGAAGCACTGATATTTGACGC
CGTCAAAATGTTCGCTGAAGGTCTTAAGGCCTCAGAACCTTTGGTTCAATCAATGAGC
ATCAGCTGTTATAATGATAAGGAAAAACTTAGATCCGGACTTACCGTTGTGAATATGATG
AAAAGTTTAGAATATGTAGGACTAACAGGTCCCATAAAATTCGACGTCAGAGGTTTCA
GGAGTACTTTTGCACTGGATGTTTTTGAATTAATGGAAGGGGGGCAAACCGTGGTCGG
CCACTGGAATGCGTCTCATTCTCCACCACTAAATATTTCCCGCTCGTTTCCCCCTGTGCC
TAGTGATGAAGAAGATATTAGAAATAAAACGTTCAGAGTTATGATCACCTTGACTGAAC
CCTACGGCATGAGGGTAGAATCTTCACTACCGTTGTATGGAAATGATCAATACGAAGGA
TTTAGTGTGGATTTAATTAAAGAGTTAGCCGCCATGAGAGGTTTCAACTACACGTTTCT
TGTAAGAATGGATGCAAAAAAATGGAAATTATGACAATAAAACGGGTAAATGGAACGGC
CTAATAGGAGACTTAATAGATGAGGTATCTGATCTTGCGATATGTGACTTGACCATTACT
AAGGAAAGAGCAGAAGCTGTTGATTTTACAGGACCCTTTATGATGTTGGGTGTCAGCA
TATTATATAAAAAACCAACCAAAGCTCCACCGAGTTTTTTCTCGTTCGCTGACCCATTC
GCTTTTGAAGTTTGGAAATTACTTATCGTGTCATGGTTGGGAGTATCTTTAATTCTATTC

GCTCTCGGAAGAATATCTCCAACTGAATGGGAAAATCCATATCCTTGCATAGAAGAACC
GGAGTTTTTGGTAAATCAGCTGGACCTGAGGAATTGTGCTTGGTTCATAACTGGATCTA
TTATGCAGCAAGGTTCTGAAATTGAGTTAAAGTCAATTTCTACAAGAATGGTTGCCGGT
ATGTGGTGGTTTTTCACCCTTTTAATGGTGTCTTCCTACACTGCCAACTTGGCAGCTTTC
CTCACAACCGAAAAACCCGATCCCCATTTTAACAATTTACATGAATTGGTTGAAAGAGC
CCCAATAAAAGAAATTAAAATTGGTGCAAAATCTGGTGGAGCTACGGAAGTATTTTTAA
GAGATAAATGGTTAGCCGACAAAACCAGCGATTTTGGAAAGGCTTATACATTATATATG
AAAGATAAAGATAACCCAAAAATATTGGACAACAAAGATGGAGTGGCTGAAGCGCAA
AAGGGTTATTATGCATTTTTCATGGAAGATCAATCGATCTCTTATGAAACACAAAGAAA
ATGCGATCTGAATAAAGTTGGTGATAAATTGGATGACAAAGGATATGGAATCGCGATGA
GAAAAAATTCAACATACAGAAGCACTTTATCGACAGCAATTCTTTCGTTACAAAATTCT
GGAAAAATTGATGAAATAAAGAAGAAATGGTGGGAGGAAAGGAAAGGTGGCGGTCA
ATGTTCGTCGGACAGCGGAAGTACCGATGCTACGCCCCTAAATCTGAAAGGCGTTGAA
GGGGTATTTTGGGTGACCATCGGAGGTACCATACTAGCTGTCTTTTTGGCGATGCTCGA
AACCGTTTTATATTGTCTTAAAAAAGCTTCCCGGACTAGAACGACGTTTTGGGCGAACC
TAAAGGAAGAAATCAAATTTTACTTCAGATTTAGCAAAATGGAGAAACACGTAGGAAA
TGAAGGTGAAAATGGAGAAGAACCATCTGAAGAGAGAGATATAAACTATGAATTAGTG
AAAAAGCAGAGTTCCGAAACGTTGCAAAGTAATAATAGCAGTAGGAGTAGAAGTAGG
CGCAGTAAAAGTAAAAACAAAAATAGATTAAGACCACCCAATTTGAGTGTTGCAGCAG
GGACGATGGGTTGGATGGTTACTGAAAATGAACCGTAGTTTGTAAAATAAACTAAAAA
ACTAATTTATATTTTGTAATATATTAAAGTAGCTTAACAAGCCTTTGAGACCCTTTTTGAC
ATTCCTGCTCCGATTATTAAATCTTGACATCACATTCAAAAAAGCATATAAACAAGATAA
CCAATATGTAAATTATAAGTTTTTCTTCTTTCGATATTGCGTTTATTAATTTTTAGTTTGAC
AATTTTAAGCATATCATTTGACGTTGGAATTTGATAAGGTTAATTGTAAAGTCTTTTAAT
GCTAATTTCAGAAAAATATAGAATAGAAAATTAATAAACATCTAGTGAATGTTTTAGCTC
ACTTGGTGACCAATACACACGTATTTTTTATTTTGTATGGAGTAATTTAAATAATATTGTG
AAATTAACCGAGTTTGTAATAAAATTTGATTAAAAGGAAGTATCCGACATGTGATCTGC
TTTGATGGTTAAATACTTGTGTAGCTACAGAGGGAGCTTATTTAGAATATGTGTGCTGGT
TATTGTGGCAAGTAAAAAAAAAGTTGAAATGTTAGCAATTATCCACATTAAAATCTTTTCT
TTGTTACAAAGTTGATTAAAAAAATCCTTTCACCGTTTATTTCATTTTTCAATAGCATTC
ACTTCCCCGTGTCATTGAGATTTCCAAGTTTTAAAATTAAACTTTTTTATGAGGTCCTGA
ATTATAACCTCAAAATAATCCGGTATCTCCTTGATGCTAAATTTAACCATTAAACTATCTA
CACATTAGAGTAGTAAACTGAAATTGGGATTTCCCGACATACCTATCCTGCGCAACTAC
TTTAAATTTCTAAATTAAAATAATATTATTCGCAAGATCGCTAGGAGATTAATTGTAATCC
AATGTTGTAAAATCAAATGCTATACACATACTGTAAAACCAAGTATACAGTATTTCAATT
ATTATCTTTTTTTGACCGAAAGTATCCCCTCGATGTTAAATTCAACCGCAGTAATAACTA
AACTAGTTCTACAGAGGTTGTTTTGGTAATGGAGTGATTAGAATTAATCAACTGATGAA

GAACTGCCGAATGTGAAGGATATTTTATTTTTTTTTTTTTT

>Cluster-12689.27206 MsigIR64a

TTTTAAGCTGTGTAATTTAAACTTACTTCGATTACACATTTTGGCGTAATTAAATTAACA
CTAGCCTAGATGCCTTTTATTACAATCCATCAATATATTATAACAAAATGTTTTAAATCTT
GTGTTACAAATGCAGTGCAAATTTGTGTTGGTGTTTTAAATGCAAAAGGTTTGTGTGAA
TGGGTGTGTGAACATTATTACAAGCTTATATTAGGTACAGTTTCTAATCAATAAAAAACA
TTCATTTCATCCTCCTCTTTCTTGTAGGTTCGTATTTCGTACAGCAGGATCAACAAACTA
ATCAGGATTCCAATGAAGAAAACAGTATAAAGAGGATAAAAGTAATCAATACCGAGAC
TGATAAGGTCTACAGTGGTTTTAATGCACTCGGGTTTCGTCGGATGCCAGTACTTCATTT
GTTTAGGCAAAATTCCACCTTCCGCTACCCGCTGCAAGCAAGTATCGAACATATCCCTA
AACGGTGATCTCTTCGGAACTAGGAGATTACCTTGTTGCAGTGACATTGTTATTTCTCTT
AGTTCGCAAATCGCTTGATCGGAATAAGTAGTGGCGATCAGTGAATAAACGTTCCCCAT
GGCAAAATGAAACGCGAAGCCTCCTTCTTGTACTAGTTCAACACCTTTTTTCGTCGTGT
AAAAGTTTGATGAATTTCCTTTTCCAAGAATTTTCTTATGGTAGAGTTCTTGTACCATTT
TATCCGTGGTTTTTACGTAAAAATCTCTATTAAGTGGCGATTCTTCGGATCCAGCTTTCA
GCGGAGAGTTGAGAAGATCTTCGACGGTTTTGATCATGGTTTTGGGTTCGATTAGAAG
ACTGGAAACGATGACGGCCGAGTAGAATTGGTAAGTTATCGAGGCACACATCAACATA
ACAAAAAGCGTCATTCGTCCCGAACTGAAGATGGGAATGTGACTCAGATCTTGTTGAC
AAAAGATAGCCATCGTATTCATAATCATAATGGTCCAGCTAGTTTCTGTTGTTTGAGATA
GATGTCGTTTCTCTGATACGTGAGTGTATTTTAAAAATACAACGCAAAATAAACACATG
AAAATTATACAAACCCACACTGTATAAGTGAAAGGCATAACGAAAGCTTCCAAAGAAT
TAAGTCTCCTGGGATTTCTAAAAATGTATCCACTCCTTGTCACCCATGTCTGTCTACCAT
ATTCTGCAAATGGGAGTCGTTCCTCTTTCACAAGCAATACAGTACTGCTATAATCAATG
GCTCTTGTCCTTAATTCCTCTACCACACCATCAACTGTACCGTCAGCAGCTACGTAGCC
CCATGAATCTGTTAGGGTTATATTTGGCGTAAAGTTGTAGTATTCCACACAAGAATTTAA
TATGTCGTATTGATTTCTGCTAACTCCATTTAAATCAATTTGGTCTTTTGTTTGTAAGTAG
TCCAACAGGTTTCCTTTAAAAGGTGCCTTACACACTACGCTCGCTTTCAATGTAACTCC
TGTCATGTTCCTTCTGGACATATATTTATGGACGTTATCCATCGCAACGAATCCCAGAGT
CTCGTTGTATACATCAAAAATTCTAGTTTTTAATTCTCCCCCTTTCCAGGTAGCCGGATT
GTAAACATCTTCTATAACTAGATCGGTACGATTTTCGATAACTTGGGGACGAACTAGAG
TGATATCGGCATCTATGTATAATTCGACGTCTTTAAAAATATTTGAAAAGTTCGGATTATT
GGACAGTAGCAACCAGTGCACCTTGCGGTAAAAATGTTGTTCCATTCCGCACTTAATCA
GAAACAATTTTAATTCCTCATATTCACAATCTCCGTCTACTATAATTCCAATATGGCGAA
AGTTGTTCTCGAAAAATTTCATTTTCCCCATAAACGAGTCAACATTTACGAAAGCCAGA
TGTTGATTTCCAAAACTAAGAGATTTAGCCAATTGCATCCTCTCCCTATTTGTGAAACAT
CCGACGACAGTGGCGTAATTAATGGTTTTTTGTTTAAAATATCCCTGAATTAATTTAACA
TCTAAAAATCCGTCCACCAAGAAAACAGAAATTTGAAGTATAAAAACAATTCTTACAA

ATCGATTAACTTTCATTCTGTGTATCGTCGTTTCGAACTGAG

>Cluster-12689.12534 MsigIR21a

GGGAAACATGTTGTATTTGTACTTATTGTTAATAACAAAATTGACCGTCGTCTCTTCTAT
TCAAACGGTGGATAAAAGAGCGCTACAGAAATCGCACGAAAAATCCCAACAAGAAAA
ATGGGCGGATGCGTTTTTAAACAAAATCCAGGTCGAAAAACAACTGGATTTGGTCATG
CTTTTAAAGTTAATTACTTTACAGTATTTATCTGACTGTACGCCGATCATCTTATTCGATT
CATTCACCGAAAAAAAGGATAACTTACTGTTAGAAAAATTATTAACCAACTTCCCTATA
GCCTACATTCACGGACAAATAACCGAGGATTACAAAGTATCTTTGAAAATATCAAAGGA
AGAGATGCAGCCCACTTGCATTAGTTACATGTTGTTTATGAAAGATGTGATGAAATCGA
AAGATGTTATCGGAGAACAGAATTACGAAAGAGTTGTTGTTGTGGCCAGGTCTTCGCA
GTGGAGAGTGTTTGAATTTTTGTCGCACGAAGAATCGAGGTATTTTGTTAATTTGTTGG
TTGTTGTTCAATCTGAAAGAATTATGGCTGCACACGAGGAAGCTCCGTATATCTTATATA
CTCATAAGTTATATATAGATGCTTTGGGTTCTAGTAAGCCAATTGTTCTGTCAAGTTTTCA
AGATGGAAATTTAACCAGAAATGTGGACTTATTTCCCAAAAAGATTGTTGATGGTTTTT
CGGGGCACAGATTCATTGTCGCCATCTCTCATCAACCCCCATACGTTATTTCCAAAGGG
CTGAGGACAACTGACGGGGAAAAAGTATTTGAAGGAATCGAAATTCGTTTGGTTAATT
TGCTATCATTACTCTACAACTTTACGACTGACTACAGGGAAGCAACGGAGGATAGTGA
AGTAGGTTCTTCAGAAGCAGTTACCAGAACCATAGTTAAGAAAAAAGCCAATATTGGA
ATCGGTGGTATATATGTTACTCCAGATAAGTTGTTCAGAATGGGTTTAACAGGATGGCAT
TCTAGAGATTGTGCATCGTTTATTTCTCTTGCTTCGACGGCTTTGCCCAGATATCGTGCC
ATTTTGGGGCCCTTCAACTGGACAGTGTGGTTAGCTCTGATAGTCATTTACATGGGAGG
TATATTCCCTTTAGCTTTTTCGGATAAATTAACACTTAGACATCTACTTAAAAACCCCGA
AGAAATGGAAAATATGTTTTGGTATGTGTTTGGCACCTTCACTAATTGCTTCACGTTTAG
TGGAAAAGGATCATGGAGCAAAGCCGATAAAGTCACGACCAAATTGCTTATAGGTTTT
TACTGGCTTTTTACGATCATCATCACGGCCTGTTACACTGGTTCCATTATCGCGTTTGTA
ACATTACCTGTATTTCCTTCGGTTGTAGATACAGCTCGTCAACTGCTTAGCGGATGGTAT
CAAATAGGCGTCTTAGATAAAGGGGAATGGCAATATTTATTTCTCAACTCTAGTGACGA
GGTGGCTGCCAAACTTTTAAAAAATTTGGATTTGGTTAGTACCATCGAAGATGGATTAA
AAAACACTACTAGATATTCGCTGTGGAGATATGCTTTTCTTGGATCCCGAGCCCAATTG
GATTACATCGTTAGAACAAATATGACTTCTAAAGGTAAACGTTCTGTATTACACATTAGC
AAAGAATGTTTTGTGCCATTCAGCGTGTCGCTGGCCTATCCTTTAAAGGCCGTCTATGG
AGATGTCATTACTAAAGGAATAGAATCGATTAAAGAAGCTGGAATTATGAACAAAATAA
AGGGGGACGTCGAGTGGGAAATGATGCGTAGCGCGACTGGACAACTGCTTGCAGCTA
GTACTGGAGCTAGTTTAAAAGCGTTAAGCTACGAGGACAAAGCGTTGAGTTTAGAAGA
CACGCAAGGGATGTTTCTCTTGTTGGCGATTGGTTTTGTAATAGGAGGTGGGGCTTTGG
TCTTCGAATGGTTCGGAGGCTGTTACAAAATTTGCCAAAGAACTAGAAGGGGAAGTAA
TGAGTCCATTGAAAGTAATCCTCGAACTCACGAAAGGCAAACGAAAAAACAAAAGAA

GTTCAATTTTCAAGACAACTACGCCATGTTGAAGAAATCGTTTGAAAACAAATCTTTCG
AACATACACCTTTCGAAAACAAGTCTTTTGAAAATAGCGCTTTGGAAAATCAATCGCTC
GAAAATAATATTACTTATGATGAAAGCGAAAATTTTAAAAATAATGTTCAGAATGAAAA
TGTCCCACCAGGTGCTTGTTGTGAAAACGCTCCCAGAAATGAAGAACACATTGAGAA
AATGATTGACACAATGTTTGATGACGTTTTAGGAGAAGATAATGCTGAAGAACAAAAG
AATCGAAGATTATCAAACTGACGAAGAAGAAGAAAGTCAGTATTTCTAGTGAACTTTA
ATTCCAGTAATTACAATTAATCAATTTGATGTAGTAGATGGATACAAATAAAAGAATTAA
G
>Cluster-12689.34844 MsigIR64a.1
AACGGCTTTTGGTATGTTGCAGAAAAGTGAAATGCAAAAAATGTTTTCGTACTTGTTG
ATGTGTTTAATGGTGATTAATCCTATAGTATTGGGTTATTTAGATTGTGATTTGATAACGG
AATTTAGTAAGACAAAATTTGTCTCGAGAGGAACTATTTTCAGCTGTATGAAACCAAAA
GATACCATATCTCTGTCAAAATGTTTAATGAGGAACTATTTACAATTTTATATAAATAATA
ATGATGATATTTCTTCTGAAAGATTTGACAAAACGTTAACTATCACTCAGTCCCATTTAG
GAGTCATTTTAGATGGCGACTGTGAAGATTGGCCGTTAGTTATAAATGAGAATACCGAC
AGAAAATACTTTTTTGAAACCTATCACTGGTTAGTGCTCAGCCGCGATAAGAACGTAAC
TAACGATTTAAGCAGCCTCAAACTAAATATCAATGCGGAGATGCATTTGGTTCCCTTGG
ACTCGTACAACGAAGAAAATGACACGTACATTATATATGACGTGTACAATCCGGCGTCA
GCGCATGGAGGAGAATTTAAAATTATGGAAATCGGTTATTTTGAAAAAAGTACGGGTTA
TGTTATCACAAATTATGAAAATAAATATTGGAGAAGAAGAAATATGACTGGCACTCATT
TTAAATCTGCGATAGTGGTCCCACAATTAGACCAACCTTTACGAGATTACCTCGAAAGT
GATGAGAATCGACAAATAGATTCGATGCACAGGTTTCAAGCTGTTACTGTTAGGAATTG
TAGGGACATGTACAATTTCAGTGTAGAAATGCGAAGAACCGATTCTTGGGGTTATATTC
AAGACGATGGTAAGTTTGACGGCTTAGTAAGATTGTTGGAAAAGAGAATAGTTGATTT
TGGAAGTTCCCCACTTTTATTTAAACTAGATCGAATGCCGTTTGTAGACTACGGATATGG
AAATTGGATACTAAAGTCTTTCTTCATTTATCGAAAACCCAAAGTTACAACGAGTTCTT
ATGAAATATTTTTAAGACCCTTGGACGAAACAGTTTGGTTATTGACTTTATTGACCATCT
GTATTATATCGATACTTATGAAAATTGTCTTTGTAAATGAAGTAAGTCTTATACCAGAAG
AAAAGGTACGCGTGGAGGATACCACTTGGAGTTTCTTGGTACTTTTCACCCTCGGAGC
ATTCTGCCAACAAAGCGCCGCCTGTACACCTCAATTTCTCAGCAGTCGCATACTAGCTT
TCTTCATTTTTCTCTTCTCTATTCTAATCTACCAGTTTTACTCGGCCAGTATAGTCTCCTAT
CTACTGTTGGATCCTCCTAGGACTATTTTTAACTTGAAAGATTTGCGGCTGAGTTCGTTA
GAAGTGGGAGTGGAGGACATTTTGATCGACAGAAATTATTTTGTGCAAACCACTGATC
CCGACGCTATCGACTTATTTGAAATGAAAATCAAATACAGCAACAACAATTCCGGTTTC
TATACTCCAGCGGTCGGTTTGGAATTGGTCAGGAGAGGAGGATTCGCTTTTCACGTCG
AGACGAGCACGGCTTACCCGATCATCGAAGAGACTTTTTCCAATCAACAAATTTGCGA
AATCGAAGAAGTGCAGATGTACAGAACTCAGCCGATGCATACGAACTTACCGAAGAAC

TCTCCGTTTAAGGAAATGATGAACTATTGCATGTTCAAGCAAGCTGAGAACGGTAATAT
TGACCGACTAAGAAAACACTGGGATGCAAGAGAACCGACCTGCATTGAAGGGGCAAA
AAAGCAGGAATTTAACGTCAGCTTCAAAGAATTTTCCTGCGGCCCTGTCGCTGTTGCTT
TGGGAATATTGTTCGGAATGATCTTTGCAATTTTTGAAATTTTGATATTTAATAAACATAA
GATATTTGATTGTCTAAAATCCCGAATGAAAGCTAAGAAGTCTGATAAAACTTATCCTTT
TATCAATTAGACATCAAGACGATAGTAACATAAAGAGGTCATGCTGAAATTTCACAAGA
ATCTGAAAATTAAATACTTGTTTCTGTAGTTTGATTGTTAAGATAATAAATAATAATAAAC
AAATGCACA
>Cluster-12689.28734 MsigIR75s
GAAAGTCGTAGTAATAATGAAACTGGCAATATTTTTCTTTATATTCAGTTTTGTTAGTATT
CGTGCCATCAAATTTGATAGTATAATAGCCTCACTAAACCTTTCTGAAGCAAATGTCGTT
ACTCAATTTTCATGCGATAAAAAAGTTTAATTTTTAGAATCATTTCATCTGCTGAAACAG
CTAGCTAAACAAAATCTCGTTCGAATTTGGAATATGAATCTCGATAACTTGGGTTATGTT
ATTCTGGCAAATAAACATACATTTGTTATGAATTTGATGTGTGAACATTCCATAGCACTT
CTAAAGCAAGCTAATAAAGTTAAATATTTTGCATTTCCTTATAAATGGATACTGTTCCATA
CTACACCTTTGAACGTAGTCATAGAACAATATTTTAGAGATTTAGATATTTTAACAGATA
GTGACATTACTCTCTACTATGAGCGTGAAAATGATACTACATTAATAAATAAAATTTACA
AGGATTGTGCAAGATGTTTAATAAAAATAGAAAAAGTTGGTTATTGGAGTGAAGGTGA
AGGTTATCATCATTTAGGTATGGAAAAAAGCATGGCCAGAAGAAGAAACGACTTACAA
AGAATTATCCTAAATTCTTGCCTTGTTATTACTAACAACGATACTTTAAATCACTTAACTG
ACAAAAGAGACAAACATATTGACAGTATAACAAAAGTTAGTTATATCTTAATGCAGCAT
TTAGCTGATATTGTTAATGCCACATTTACCTATACGGTTGAAAGCACTTGGGGTTACAAA
ATTAATAATTCTGAATGGAATGGAATGATTGGGCAATTATTAAAGAAGAAAGCAGATCT
TGGAGGATCTCCATTATTTCTAACATTGGATAGAGTGGATTTAATAGATTATGTTACAATG
CCTACTCCTACGGTATCGCATTTTATATTTAGAGAACCAAAATTATCCTATGTTACCAACG
TGTTTACACTACCATTCGATAGACAAGTTTGGGTATCCAGTGTTGCTTTGGTTATAATATT
AGGATTTTTCATATACATTATTTTGAAATGGGAGTATCTAAAAAAAGATGATACCACCAA
ACAGGTCACTAAGGATAATAGCAACGTTAACATGGCAGAGGTTGCACTTATTTCTTTTG
GAGCAGTTTGTCAACAAGGATCCCCTACAATACCAACTAGCCTATCAGGACGAATCAC
CACTATAATATTATTTGTGTCCTTAATGTTCTTGTATACGTCATATTCTGCAAATATTGTTG
CTCTACTGCAGTCTTCTTCATCTAGTATTCGAACTTTGGAAGATTTACTACATTCAAGAA
TGAAAGTTGGTGTACAGGATACTGTCTTCAACAGATTTTACTTTCCAAACGCCAAAGA
ACCAATTCGCAAAGCGCTATATGAACAAAAAGTGGCTCCTCCTGGCCGTCCACCGCAG
TATATGTCCTTACTTGAAGGAGTTCTTCACATAAGAGAAGGTTTATTTGCATTTCATATG
GAAACCGGCGTAGGATATAAATTAATTGGGGAAATATTCAAAGAACATGAAAAGTGTG
GACTAAAAGGGATTCCATTTTTAGAGATTATCAAGGATCCGTGGTTAGCAATTCAAAAG
AACTCTTCCTATAAAGAGTTGATAAAAATAGGCCTAAGAAAAATTCAAGAAAGTGGAA

TACAAGAAAGAGAAAATAATTTAATTTACACGAAGAGACCAAAGTGCTCATCGAGTGG
ATCTACTTTTATTAATGTCGGTCTAGTGGATTGTTACGCAGCTGTTGTCGTTTTAGCATTT
GGTATTGTACTTTCGTTACTAATATGGACCCTTGAAGTCATGTCATATAAAAGGTTGAAG
AAACTTAATAAATTCAGGATAAGAAAAGCACAATGGAATTTGTAAATTCAATAAAAACT
GTATAAACTGCTTAAATCACGGTTCGTGAAAACACATGTTGCTTAATAGTTGTATTGCAT
AAAAATTAAAGAAAATAAGTTCAAAA
>Cluster-12689.30630 MsigGluR2
GAAAACAGCAATTTGCATTTCGGAAATGTAATCAAACGGTTTTGGAGAAATTGTAGTG
AATTTTTAATATCGATTTAAAATGGCCAATAACCTGCTATGGCGTGTTTTTTGGGTGTTTT
GTTTATTTATTTTAGGATATGTCGCTTGTGCTAAAACTGTTGTGACTATAGGCGCACTTTT
AGAAGATTTAACCTCGCAAATAAATATTCCATTAAATAGTACAATTTATAAGAAAAATAT
GTTTGATCAGAGAGCTTACTTTTCCACAAAAATGATGGAAGTATCTGCGACAGACAGT
TTCGAAGCCAGCCAAAGCTTATGCAGAATGTTGGAAGGTGATATGGGCGTGATCGCCG
TATATTCCGATGCCGTTAAAACTATTCCGATTTTGGAGTCGACTTGTACTAATTTTGAAA
TTCCTTTTTTCACAACGTCCTGGAGAAACCCGTCATCGCCTAAAAGGGATACTGAGTC
GGAAGCTAGAGCACTTCTCAGCTTCTTCCCAGAAGCTGAGCTTTATTCTCGAGGTTTG
GCAGAGATCGTGAAAAGCTTGCAATGGAATAGTTTTATAATAATTTACGAAACAGAGGA
AGGTTTGGTAAGAATGCAAGAAATTTTAAAATTGCAGGAACTCAGTCCCGATTCTAAG
AAAAATAACATCCTTGTGATGGAACTTGGCTCAGGTCCTGATTACAGGCCACTTTTGAA
GAAAATTAAAAATACGACAGAAGACAACGTCATTTTGGATTGTCACAACGATAAAATAT
TGACAGTCCTGACTCAAGCTAACAGTTTGGGAATGCTAACTTTACACAATAGATATTTC
ATAACGTCGTTGGATGCTCATACGTTGGACTTTTCAATTTTGAATACTACTGCCAATATT
ACAACGGTCAGATTACACGATCCCAAAAGTGATGACTTTTTAAACACGATTCATCGTTG
GGAACTCACAGAATTTGAAAATCATAACAGAAGAATTCCTCTAGATCCGATGGCCATAA
AGACTGAAACTGTTTTGTTCCATGACGCAATACTTCTTGTAACAGATACGATAAATTCTA
TGTCAATTAAACCTGGAATCAGCAACAAACCGGTATTCTGTAACGGTACCGATGTGTCT
GAGGATGGATTTACTTTAAGAAAATACATTCAAATCAATACACCAACGCTTACATTAAC
TGGGCCGATAAAATTCAATGATGAAGGGGAACGTATAGATTTTACCATTCACGTTGTAG
ACATAATAGACGAAACTTTGATAGCAACGTGGTATGCAGGCAATGAAAGCCTTGTTTTA
GCTAGAGACTTTAATCAGACGAATAATGCAGTTGTATCTAATTTACAAAAAATAACTGT
AATAGTATCTTCCAGAATTGGAAAACCATATCTGACGTGGAGAAGTCCGACGTACGAG
GGCGAAATATTAACTGGAAACAACCGGTTTGAGGGGTATTCCTTGGATCTAATTGCTGG
TATTGCAAAAATTATTGGATTTAATTTTCAGTTTGAAATTACTGATAAATATGGAAATTGG
GATCCAGCTGAAAAAAGGTGGAATGGTTTGATTGGAGAAGTTCTGGAAAAAAGAGCT
CACCTTGCAATATGCGATTTAACCATTACACCTGAACGTAGAGAAGTTGTAGACTTCAG
CATGCCATTTATGACGTTAGGTATATCCATCTTACATGTACAACCTAGTAATGAAGATGT
GAATATGTTTGGTTTTCTAGATCCTTTTTCACCTAGCGTTTGGATATACACAGCCACACT

CTACCTCGTAATCTCTGTGGTGTTATTTTTCGTTTCGAGGATGACACCCGGTGATTGGGA
GAATCCTCATCCTTGTGAAGAGAACCCAGAAGAACTGGAAAACATATGGGATATCAAA
AATTCTCTTTGGCTTACTCTCGGTTCTATAATGACCCAGGGTTGTGATATTTTACCAAAA
GGAATTTCGTCAAGAATGGCAGTTGGGATGTGGTGGTTCTTTTCCCTAATTATGACTAG
TTCTTATACAGCTAACCTGGCGGCCTTTTTAACCAAAGCTAACATGGAACCCGCAATAG
ACGGTGCGGAAGCTTTGGCCAAACAAACGAAAATCAAGTATGGTTGTATGTCCAATGG
AGCCACGATGTCGTTTTTTAAAAATTCAAATTTTTCCACATTCCAACGAATGTGGATGA
ATATGCAGCAGGCGAAACCGAGTGTATTTGAGAATACTAATGACGATGGTGTCCAAAG
AGTGAAAAATACAAATAAGGGATTGTACGCTTTCTTGATGGAATCAACCCAGATCGAAT
ACGAAGTTGAAACTAAGTGTTATTTGAAACAAATCGGAGAATGGTTGGATTCTAAAAG
TTTCGGAATAGCTATGCCTATGAATTCTCCATATAGAAGCGCCATTAATCATGCCGTTTTA
AAAATGCAGGAATCTGGAGAGCTTACGGAATTAAAGAATAAGTGGTGGAAAGATAAAC
AAAACGCTACTCATTGTAGTGAAGTTGAAGGAGAAGATGGTGCTGATGGTAAATTAGG
TCTAATAAATGTCGGTGGTGTATTTTTGGTACTGGGAATCGGAATAGTAATGTCATGTGT
ATGTGCTCTTGTGGAGTTTCTTTGGAATTGTAGAAATATATCTGTAGAAGAAAACATTAC
TTACGCTCAGGCTCTAAAAGCGGAAGTGAAATTCGCCTGTAACATTTTTATAACGAAGA
AAAGGGTAAAACCACTTTTATCGGAAGCTGGTTCATCAGAGGGAAGTGATAAAGTAGA
TGAGAAACATGATAACAAGAGTATGATCCAAAGCATACTGCAAAGTATCGGTTCCAATT
TAAATATAGATCGTTCTTCATGAATAACTACAATTAATTATTTAATACAAATTAATAAAGT
ATGTAATATAATATTCTATTATGTCAAAAAAAA
>Cluster-12689.33625 MsigIR75q
TTTTAAAAAGATTAAACAATTTTATTTGAAATATTTTAACTTCGCTCAAAAACTAATAGC
AAATAATTACTTCCACTTACATCAAAAAAATTTCTATTTGTATTTATTTCAGTTCTAGGTG
CCTCTAAATACAATAACTAAATATCTGCAAGCAGTTGTGTAGGAACTTAAGCATAATTAT
GAAATAATCCATTTCGTGACATGTTTTCATCGTAAAAGTTAGCAGCCACATCAACAAAC
TTTTTTCGATTATCTTTTTATTTGACAGCAGAACTGTGGTCAGTGATCTCAACAACAAAT
TGTTTTCTAACTTTCTTGTCATAACATATTTCAATGCCGAGTAGTAAAAGACTCAAAGCC
AATCCCGCTCCAAAGATGAGAAAAGCCGCATAACAATCCAAAATACCAGCACTACCGA
AGCTATTTCCTTTTCCGTGACAAACCGGCCTCTTCGTATACAGGCGGTTGACGTGTCTT
TTTTGGATTCCTGACTCCAATATTTTTCGCAAACCAACAGTAACGACTTCCTTGTAGGC
AGAATTCTTTTTGATGGACAAATAAGTTTTTGACAAACTGAGAAAATCGATTTCTTTTA
AACTGCATTTCTCGTCCTCTTGAAAAGTTTCACCGATCATTTTGTACCCGTTGGAAACG
TCCACGTTGAAAGCGAAAAATTCGTTTTGAACTCTGGTGATTCCTTCTTGCAAGGTCAT
GAAGTTGGGTTTTTGACCTTTCGGTGCGATCTTTTGCTGATATATGGCTTTTCTCACTGG
ATCCGTTGCCGCCTTAAAGAAATAACGCTGATAGGCGATGTCTTCAACTCCCAAGGTAA
TCCTGGAGTTCAGCAAATCTTTAGTACTTTTAATACTTTCAGTGGTACTTTGCAAAATGG
CCACGATATTGGCGGAGTAGGCTGTATACAAAAACATGAAAGCGATAAATGAAAAAAC

AGTGACTATTCTGCCAGAGTTACTCAACGGTTCACTGTCAGTTCCTTGTTGCGTAATAG
CACCCAGTTCCATGACTAATACGTCGAAAAATCTCGGTCTTAACGGCGTTATGGCATTC
GCGTGTGTTTCTGCAGCTTTGTGTTTGAAAATCGGGTCTTTCCATTCCCAAATAACGAT
TAAATACACTACAGCAAATATTAATGGAACCAATGCAAAACAACAGTACCAAACGTAA
ATATCAAATGGTAATGTGAAAACGTTGGATACATAAGAAATTGGTGGAGCTCTGAAGAT
AAATTTTAAAAGAGTGGGAGCGTCGGGTGCTACGAATTCTATTACTGCTATTCGATCTAT
TGTGACGAATAAACCAGTACCTCCAATTTCAGCAATTCCAGATTGCAAATCACCGATCA
TGCCTAAAAATTTAGTTGTATCATTGAAGCGATATCCCCAAGTTGGTTGAAAAATTGGT
GTTTGGGTAGCGTTTAATATGTCTAATAAACATTTCATTACAAGCCAATCCAACTTTGAT
ATTGGTTCAATATGAGAATTTCTGTAGTCTTCTAAATGGTTGTATGTGTCAGAATCCGTA
ACCACGTAAGAAATATTTAAATTTGTTCCTAATAAATTGGTTCTATTTCTCGAAACAGAA
AATGGGTTATATTCTATAAAACCTTTAATCGGACTCCATTTTGCTAATTCGTTATTTAAAA
ATTCGGGTGATTGCTCGAAGTTTTTGTATAACGATTTAATGCTATAATTATAATAGTTATC
AATACGATTCTCCTCTTCCTGTATTTTCTGTACAATGAATATTTGACTATCCACTCTGAAA
TATATATCTTGGAACATTGAATAATCGATGTGACCCCAAATTATCCATCTGTATGGTCTAT
AGAACAAATTTAATTGACTAGCCTTTTTTAGTATTTTCTGAAAACCGTTGCAAGTTGAA
TCTAGCAAAAAAAGCTGATGTTCCTTAGGAGACAAGTATCTCGCACTATCACTAAATTT
TGCAATACGACATGCAAAGCTTTTATTTGACATTTCCTTCCAAAGTAATAGTTGATCATC
TTTGGACCAACAAACATAGGCGGATATCTTAATGGGGCCATTTAATTTAACAACAAAAT
CCTTTATCAACCTAATAATTTCATGATTTTCAGATTGGACGGACAATTTCAGTATGACCA
TTAGAAGAAACGAAACCG
>Cluster-12689.3217 MsigGluR1
TATTTATATTATTTTAACAGGTTTTTATATCTAAAACAGCTAGATTGTCACTATAAATGTGC
AAATGTGCTTAAATCATATGTTATTACAAGAAACATCTTTCTTCTTTACCTTGAAGATAC
ATGATCAGCACAAAAACGAATTACTGTTCGAATAAATCCCTCCCGGATCTCTGCGAGTT
ATTAATTTGTGGAGACACCCTATTTCTTGGTGATGCTCTAACTGGAGGTAGAGGTGGGT
GTGGAGGAATATCTAACATGGCGGGAACGTACGAAACTCCTGACATACATTTTGAACAT
TTTCTACGAAGAGCGGGACGTTGACGTGAACCGCTACATTTTAGAGCGAAACAAAATT
CGCTCTTCATTTCTGAGCAAAGTGACTGATGAGGCACGCTGGCAGTTCTCTTTTCATTT
AATGCATTTTTCTTCGAGTTATAACAGAATTCGAATATGGCAATAATGACCGCAATTGCC
AAACCGCAAAGCAAAACGACGAAAACACCTCCGATATTATCCACTCCCAAAGAATTGG
CTTTGAATTCTTTTCCCTTATCATTTCTCTGGCAGGTGTCCCCGCTATTTTTCCACCATTT
ATCATAAAACATCTGAATCTCTCCTTTTTCTTGAAGCTCTAAAATAGCCAGGGAAATTTT
GTCTCTCCAAGGACTACCTTTCGGTGTAGCAATTCCATATCCTTTGGAATCAAGGGGGC
CCCCGATCTGTGTTAAATTACAATCACGTTGTACTATGTAATCTAACATAGTAGATTCCAT
CAGGAAGGCGTAATTCCCATCAAGCACTTTTTGAATTCCTTCTTCGTACGTAGGGACGA
ATACTGACGGTTTTCGATTTTCCATAAAACGCCACATTTTTTTATATGTCTCGATCATTGA

ATCCCTAAAAAAAGTCATCGTAGATCCACTTTCTAATGTACCGTATGGGATTTCTGTTTG
ACCTGCTAAATCTTCAGCATTTTCAATTGGAGTAATCATTCTTTCTACTGTTAAAAATGC
TGCTAAATTGGCGGTGTATGAGGATATAATTATTAGGGTGAAAAACCACCAAATTCCTC
CTACTATTCTAGTTGAAGTAGCCTTTGGATTTAAGTCCGAACCTTGCTGCATTAATGTTC
CAATTGTAAACCAAAATGAGTTTGCCAAAGAAAACTGATTTTTCACCAATTCGTTCTCG
ATTTCGCATGGATGTGGGTTTTGCCACTCGCAAGGAGAAAATCTGGCAACTACAAACA
TCGTTATTGAAACCAAGATGTAGGCAGATAAGACATACAGCCAGATATGCGTGGCCAA
AGGGTTCATGAACGAGAACAACTTAGCTTGTTGACTTTTCGGTACCTTGAAAAGAATG
CTGATGCCAAGATTCATGAAGGGTTTCGTGAAGTCTATGACGCTCTCCCGGGCATAGTT
GATTGTCATCGATCCCACAGCCAAATCTGCTTTGTGCTGCATTAACTGCAAGACCATTC
CGTTCCACAGACCCGTACTCGGATCTCGTGCCCCGTACTTTCTATCCGGCACTAAATCG
AGTAGGTAATCGAAACCGACTTCCTGCGAGATACGGTCCAAGATGTCGATGCAGAAGC
CATAAAATCGACTATTTCCAGTAAAATTATTTGCGGTGTGCATCATCACATACGGTGTTT
CCAGAATTGTAGTAACCACGAGCGTAAAGTTCATAGTTCCCGGATCGAAAAATGCAGC
TCGGTCTGTAATATTCACGCCGCTTCCCGGATGCCATTCCCCAACTTTAACCAAAGCCG
GTTGTTTTAATTTGAGCAAATCTAATTTAAATTGTATACGACGACCTTCCTTAAATTCTAT
GGGGCCGCTTAATCCTTTTCTTTCCACCGAATTAATGTAGTTGATGAGACTGAGTCCAC
CGTCCCACGGCTGTTCCTTGTCGCAGGATAAGTTCGACAGTTTCAACGTGTGCGACTG
TTCGAGAGTTTGCAATCCGACGGCGAAAACGAAAACAGAATCGTATATCAAAGCTGGC
TCTGCCTGAATAAAACTAGAATTCAAAAGACGAAATTCCCTATTATACTGACTCTTGAA
CATACTTCTGATGATCTCCTTGACCGAAAGATCGTCCGTATCGACTATTCTAAAGGCAGT
CATATTTACAAAGTTGTATTTAAAATCCTCCAAGTCGAAAGATTCCATATCGAAAGTAGT
GAAGAGATAATGGTATTTGTAGTCGTTCATTTGAAGTTGTAAAATCCCTTTTAGAAAATG
TTGGAGATTTAACGGTTTAGTATCTATAACGATATTATGTATCTCCATATTTTTAATTTCCT
TTAGAACGTCCTGATATGACTCTGGATTGGCCTGCCGTAGGTGAATTTCCAGACCGCTA
TTCTGAGGGGATCTTACAAGTTCGCGGAGTTTCAATAGACCATAGTCCTTTTCATAAATA
ATAGCGATTCTAGTCCAATTAAGAAAGGCCATAACATCCTGAAAAGCAGCATTGAGCA
AATGTTGTGTGGGATGTAAATTAATAGAAAATTCTCTGATATCGCTCTCAATGTCGAGAC
GAGCTTCTAAGTGAGGAATATCTAGAGCATCACATATTGAGTGAATATGAGCTCCTAAC
AGTGGATCAGACGGACCAAAAATCGCATAGACACCGTACTGCACCAGCTGACAAGCTT
TTTTGCTTGCATGAAAACTGTCATCGCGAGGCACGTACTGAATGTCGTACACGAGTGAT
GTATAAGGGAGCAACGTTTTGTCTTTGTTGATTTTGTTGACGGCATATTTGAAAGCCAA
CTCGTTGGGGCCATTTTTCTCATCTTCCGTAAAAATTGCACCTATCTTGACTTGGGGCG
GCAGTGTGGAGGCGGCAGAAATGGAATCGAGTTTACTGGCCACGATTCCCAGAAACA
AGACGGCTGCTGCCCCCAAATGCCCCCATCTACGGCTCATGGCGACGAGCAGCCGCTT
CAACGGGACCACCTCTCATTTCACCATAGGCGAGTCATTGAAGCGGGTTAAAATCGAA
TTATAATAAATCTTAACTTGCCAAACTCCATGCAACTCACCTCTGTTTTTCGTTCACTAG

AAGTTCGGGAAGAGAAGAGACCGCACAAAATAACAGAAACGAAGTTTCAAGATCG

>Cluster-12689.37037 MsigIR93a

TTTTGTGAACTGGTGGATCGATTTTATTTAACACATTACGTATATAAATAATTTATGATGC
TTCCAGTATACTTACCTAAGGTTCACCGAAACTTGCAAATGCACTAAGATATATAAATAA
TCCTCTCTAATATTTTAATAAATTCAATTAAGACACAAAAGGTTGTATCATTTTTTGCCGT
TGTTTGTTGAAATTTCTATGCCATATTTTTTCGAAAAGTAGTAGCATCGTAGCCAACGCA
AAACCCATGAGTAGAACAAAGAAACTTCCTTGCATATCGTCCAAGTTAACTGTATGGTT
GTTTACTTCAATTAAATGTCTATTTTTCCAACATTTATCTTTTTTTGGTAAATAATCTTGA
AGCCATTTTTGGATAAGTCCAACTTGGTGTAGTTTTTTTATCTCTTCATTAATTTTAGATA
AATATGGTGATTCTGGAGCTACTATCAACGATAATTTTTCATCAAAAAATTCTTCTAAAC
CTAGAACGAAATCGCATCGACCGCTTTTTAAGAATTGTTTCTTCATAATATACTGCAATT
TCATTTTCCAATCAATGTAAATGTGTTTTCCGTCCTCGATACTTTGGATTATAGCGTCATC
ATCAGTGGAAACATGTTGAGCTCTTCCATTAAATATTGACATGTAACGTGGCTCATTTGA
AGCCTTCAGTTCTCTCTCCAAGTAACTTCCTTCACGAAAACTCCAAGTGACTGTATCTC
GATGAGCTAATAATTCGTCTAAAGTTGTTATTGGTATATCAATCTTGGGGAAAGTTAAGA
ACGCTACCAAGTTGCCGCAGTAAGTTGTGGCCATTATTAATACAACCAGCCACCAAGCT
CCCACTAATATTCTTGCACTATCTGCGTATGGAAGATGCATGCCGCCTTGTTGTAGTAGA
GCTCCATACATATACCAAATGCAGTTTTGTATAGATGCTAGACCTCCTTTCATTGGATAA
CCTTTATATTCGTAGACAGGACTGTGACGATGAATAAAATACAAAAGCGGGCCCATAGA
AACAATAGCTGCAGCTAGACCCAACCACGTACCCAGCGTAAATGGCGAGATGAACAAC
AGCGCGCGACTAAGTTCTCTGGGTCTTGCCGCCAAAAATGTGTAAGTCAGTATTGTTAT
TGGTCTCGTAAAATTCACCACTTGCTTTAATTCATCAGTTACGGTCACTGCGCACGCTC
CTAATGCGACCGTTTTATTTTTGATTAAATCCAAAAGCACTTTCGGAACTTCGTTCGTTA
AATCATTGTCGGTATCGTAAAATGTAGCATTCGATTTACCTGTGTTAGTTTTGTTAATGAT
TTCCAGCTTAAATGTAAAATTTAAATTTTTTGATAGTTCAGTAACAACATCGAACATAAG
CCCACCATACTCGACTACGTCTCCAGATTCGTTAAATTTCAGAATTTGCCAAGGTGGAT
TATGAAATGTTATCATAGGTAAATTTTTGCCTCTAAATCCATGAGCAACATGTAAAAACA
ATTCGTCTGTCATTTCGGCACCATCGCTAGGTCTCCAGGATCCCACCTGCAACAGTTTC
GGTTGTGCTCTTTGATCAGTTGTTTGGTATTCAACACCCCACGTTTCACCTGTTTTCATT
TGCCAGCAGGTACAATTGTCGCAGACGCCATTTTTCGATAGGTAATTCTTCATATTTTTT
AATAAATAATCCCTTCGTTCACTTTTAGTTGGTCTAATTGCTTCCCATTCCTCTTCTGACA
CTTGACTAGCCGTATCGAATTCTTCTTGTATAGCTTGATCTAACGCTCTAGTAAACGCTT
TGAATAATTCCTCCATATGACATTTACGTCCACCCACACACTTATTTGTTGTAAAACTGG
AGTTATATAGAAAAGCGACGTTATCCCCCTCCTTTAGCAAATTTTCAAAAATTTGTACGT
CCTGAGTATTACGGTTAGTGTCCGAAATGACATACATCCACTGATTCGTCGTGCTAGCC
AGATCCAAAACTTTGGCGTATTCCATGACGACTCCAACTAGGCGGTAACCAGCGATAA
CCAAATAATTATTTCCGAGGACTTTGGGATTGATCGTGGACATAACTGCTCTTAAATCCG

TTCTGGACATGTTTGATTCTAATTTCATTAAAGAAATGCCCGTGGCTTTGGCGTTGCTTG
TTCGACGTTGTGTGAGCGATTTAATTACTCTGGTTATCATATCTCTATTGACAGTGCCATC
ATATATTATTACTAAAGTTTTCCAAGCGTAAATTCCTGAACTACGAAGGTCTAAAAGGAT
CTGAGGTAATTCTTCACCACTCTGTATTAGTGGTATAGTAATAGCATTTTCGGGAGGCAG
TCTTGGACAATCGGAATCTGTAATAACCATGTGAACTAAATCTTCGGCTTCGGTTAGAC
GGAAAAGCCTCCAAGTATCATAACAAGAGGCAATGCTAAAAATTGCAGTTAAATCTTTT
CTTACGTTGATAGCTGTCCAAGAATAAGGATATACGTTTACTCCTCCATGTTTTAATATTT
CCCTTTTGGCATAAACAAGATAATCATCAATTTCTTGTTTCACTTCATCAAATTCTTCAA
CAAGAAACTCCCTATCAATAACAATAGCTATTGAAGCGTTAGTCGTCAATAAAGAAGGA
AACGTTTCGGATATTCCATTCTTAAAAATAGACAAAAAGAAAAGGCATGCAAACACTG
TGTAATACATCTTGCCGACAACATTTCCGACACACTTCTAACACACATTTTACGTTAACT
TAATAAATCTCATGCTGTTACTTTTTTTTTTCTAAATATTAATATCTACATTTGTGCACGTG
CGGTGTTATATTGTAAGTTGTAG
>Cluster-12689.12762 MsigIR75q.1
ATATTGTGGAACAAATTTATTCATAATCACTTATTAAATACACAACAAATTCAAAGGAGT
AATTCTGGTATTTCCATCTAAAATTTTATTGTAGTAGTAGAAATACGGATTTCATTATTGA
ATTCTTTCTTACATTTCTTTTCAAAGCAAATTTCGATAGTCAAAAGGAGAAGACTTAAA
ATTATGCCCGCTCCAAAAATTGCAAAAGCTCCATAACAATCCAGGATACCAACGCTGCC
GAAGTTACTGCCTGAGCCCTGACAAACCGGTTTTTCGTGTACAGACGGTTTATATGTC
TTCTTTGGATTCCTGACTCCAAAAGTTTTCGTAAGCCAACTTTAATCACCTCCTTGTAG
GCAGACCTTTTCTTGATGGACAAATAAGGTTCTACTAGATTTAAAAAACGGATTTCCTT
CAAACTGCATTTTTCAGCTTCTTGAAAAGTATCGCCGATCACTTTGTATCCGTTAGATGC
TTCCACGTGGAAAGCGAAAAATTCGTTTTTTACTCTGCTTATTCCTTCCGGTATGGTCAT
GAAGTTGGCTTTCTGGCCTTTTGGTGCAATTTTTTGTTGGTAGATGGCCTTTCGTACTG
GCTCTTCTGCAGTCTTGAAGTAATAATGCCCATAAACGATATCCTCGACCCCTAACGAA
ATTCTGGTATTCAACAAATCTTCCAAAGTTTTAATACTTTCCGTGGTACTTTGTAAGAGT
ACTACAATGTTGGCGGAATAAGCTGTATACAGGAACATAAAAGCGATAAATGTAAAAA
CAGTGGCTATTCGACCTGAGTTACTTAACGGTTCAGTGTCGGTACCCTGTTGCGTTATA
GCACCCAGTTCCATGACTAATACGTCAAAAAATCCCGGTCTTAACGGCACTATGGCATT
CGCGTGGCTTTTTGCTGCTTTTTGTTTAAAAAATGGATCTTTCCATTCCCAATTAACTAT
TAAATACACTATAATAAATATCAAGAGTACCAATCCAAAACAAGAGTACCAAACGTATG
TGTCGAATGACAAAGTGAATACGTTCGAAACGTAAGAAAGTGGCGGAGCCCTGAAGA
TGAATGTAATGTAAGTAGGAACGTTGGGTGCCACGTACTCTATGACATCTACTCTGTCT
ATCGTGAAGAATAAAGCGGTACCTCCAATTTCAGCAATGTCGGACTGCAAATCACCGA
TCATGCCAGAATACCGAGTCGAATTAGGGATGGGGTATCCCCAACTTGGTTGAAAAATT
GGTCTTTCAGTGGCATTTAAAATTTCCAACAGATGTTTCATTAAAAACCAATTTAACTTT
GATATAGCATCGACACGCGATTCTCTAAAATCTTCTAGATGATTGTACGTACTGGGATTT

GTAACGACATAAGAGATATTGAAACGTCTTCCCATGAGATCAGTTCTATTTCGGTAAAA
GGATAGAACTTTGTAGTCTAAAAATCCTTTCGTCGAACACCATTTCGCCAGTTTATTTTC
TAAAAATTCCGACGAACCCTCAAAAAGCTTGTATACCGATTTAATAAGATAATTATTTGG
ATTTTTATGCAGCTTTTCTATGACGAATATTTGACTGTCGAGTCTGAAATAAAAGTCCAT
GAAAACGGTGGAATTCGTTTGGCCCCAAATTATCCATCTATACGGTTGATTGAACATATT
TAATTGATTTCCCTTCTCTAGAATTTTTTCAGAACCATTGCAGTTTACATCTAACAGAAA
AAGTTGATGTTCTTTTGGAAAAGAAGATTTTAAATCGTCGTTTATCTTGCAAATTTGAC
AAGCAAAGTTTTTATCGGACATTTTCTTCCTAAATAATAATTGGTCATCTTTTGACCAAC
AAACATAAGCAGAAATCTTAGTAGGAGTGTTTAATGCAAGGACGAAATCGCCGATCAA
CTTAATGATATCATGATTTTCAGATTGCAGGGAGGTTTTCAGTAAGACCGTTAGAATAAG
CAAACCCTTTTT

>Cluster-12689.14179 MsigIR75c

TTTCTCTGTTCATAGCTTCAAATGTGTATTTTATTGCAAAGTAACTTAATGTGAATACATA
ATTATTCAATCTAATTATCTCTAAAATTTAACATCCATAGTGTGCGGAGAACTGCCTGTG
CAAAAATATTTCCACGAAAAATATTACTAGCGAAATGACAATACCCCCAAATATTATTAA
AAATGTCAAAAATAACTCTCCCAAACCCACCGCCTTGACTCTGGAGCTCAGGAGACAT
TCGGGTTTTTTCGACAACCAATAGTCTTTAGCTCGTTTGTAGAGGCCGCTGGATTGCAT
TTTTCTTAGCGTGATTTGAAAGATTTTCGTGTATTGCGAACATTTTTGGGCTACCATTCC
GATGTCACCGTCAGGTATGAATTTAATTTCCGCCAAATCGCATATCAGATCTTGATCGGA
GTCTTTCAGGCAAGAGTAGGCGGTGGCGAGCTCCGTGTGGTAGGCATAATTTCTTTGCT
TCACCTTCGAAATTCCCTCCGCCGCATTCATAAAGTGGGGAACGTCGTTCTCGTATATTT
TGGAACTGTTCAGCAAACTTATGTAGTGGTCGTCTTTTTGTTGAATTATGTACGTAAGTG
TGTACGGTAGGGGTTCTACTGCTACCTTTAAATTGCTCTCATAGAGCTCCCTAATCGTAT
GAATTGCTTCTGGAATACTACTCAGTAGCGAAGAAACCAAGCTCGAGGTGTAGTAATT
GTACACCATCATCGACATCATCAATGATGTCAAGAAAATTATTCTTCCACTGGTTCGGA
ATGGTTCTATGGCTGAACCTTGTTGGGCGATCACCGATATGGTTATCAAGAAAGAGGTG
CAAAAGCTGTATTTCGATTTACTTTCTACGAATCGATATTCCACCCAGTAACCTAAGTAT
ATCGTAATACCAACGGTCAATGCGATTACCATTAGAGCGCACCAAGTTTCCATGGTAAA
CGGTTTTAACACTTCCGCTCCTGCTTTAACTTTTCCCGGATTTACAAATATAAATGCCGC
CCTAAATTTATAAACTGGTACCAAAATGTCGTAGTAATCCGTTCTAGCAACCCGCATAA
ACCCTCCTGAAGTACTTATATCAACTACTTTTTCGTGGACCAACTGCGCAAGACCTCCA
TCCGTTCCTGATGTGTTATTGCCAAACCATGCGTTGCGAAGTGTTGTATTATGACTAGAA
CACAGAAATGTTATTGATGGTCTTTTTGTTAGTATGGCTGTCTTCTTATCCGTTCCTATTA
CTTACTGGAAATTATGTACTTCTTTTAGGGTAAAGAATAACTGAATATTAAACTTCTGGA
ATTCCGTATCGTCGGACCTCAATGCGTTTGTATAATCTTCTTCTGTTACGAAAGAGTAAT
TAAACCTGTTTCCCGTCCGTATTAACACGCCGTTCATATCTTTTCTACTTTCATAAAAAG
AACTGTTAGTTTTGAAAGGAGCTGTATTATTTTTATGAAACAATCTCTCGAATATTACCC

GTACACCGACGTTAATACCAGGATTGTACACTGAAAATATGCGGAAAGTTTCGTCTTGA
TCACCATCGAGGGTTGCAAATGTTATTCTGGAATCGAATCTGACGTTGGTTGTGGAGAA
AATACGTTGAATCTCGTCCTTGAGTATTGCCCGATTTGATATGATAAGCCAGTTGTATAA
ATATCTGAATTTATATTCGGCGTCCTTTTGCAGAATTTTCGTGCCAGACGGACAATCAAA
ATCCAAAATAAATGCCATTTCATCATACGGTGTAAGATTTAAATCCCACACATTTCCTGT
TACATCAAAGAATAACATTCTTGTAGTGCTAGTTGCAAGTAGATGTTTTTGGAGTTTTAC
TTTATCTGAATCTGACCAACACATCATGAATAAACTAAAACGCGGGTTGTTGTCATAAA
TAAACTGCGAAATTAAATTAAATTCGGTTTTTAAACATTCTACTGTAGTAACAAATAAAA
AGAACAGCTTGATAATCA
>Cluster-12689.33994 MsigGluR3
ATAGAAACTAGAAGTGAAAAATGTCGACGGGGGTGATAACATTGTGTCTGCTTGTTTTT
CAGTGTGCAGGTTTTGGAACTGGTCTACTCGACGGTGATCTCAAAATTGCTGGTATTTT
CGAAATTATACAGCTTCACCAGGCAGCATTTACATATTCTAATAATATCTACACATATGGA
AAACATCCCACAGATATAGCAGTTTCCCATATAGTTGACTCTAGCATCGTCAAAGATGA
ACCTTTTACAGCTCTTAGAGGAACTTGTTTGTTTTTAAAGCAGGGGATTGTCGGGTTAT
TTGGTCCTCAGTCATCTCTAAACTTGGAAATAGTTCAATCCATAACGGAAAGAAAGAA
CATACCGCATTTTTTGACTAGATGGGTCAATCCTTCTCAGATGGGTAGAAAAACCATCA
ACCTCTTTCCACATCCTTCGATTTTGGCCGATGCGTTTCTGGAAATTTTGATGGCGATGG
AGTGGGAAAGTTTCACGATTCTTTATACGAATTCTGAACATTTACTTAAAATTTCTGATT
TTATTGCAGAAGCGAAAGAGTGGGGGATATTGGTGTATGTGGAAAATGTGGATCCCTCT
TCAACTGGTAACTATAGAACAGTTCTTCGTAATGCCGGAAGATCGGGTCAAAAAAATTT
TATTTTGGATTGTCCTATAGAAGATTTGAAAACACTATTGACACAAATTCAAGAAGTTG
GATTACTGACTGATGGTTATCACTATTTTCTTACAAACTTGGATGCTCATACTGAGTATTT
AGATAAATATATGTTTAGTGACGCCGTAGTTACAGGGGTTCATTTTCCTAGACCACCTCC
CGACGTGGAAGTGCGAACTTCGAAGGAACTTTGCATTATATACAACGCCACATTTAAA
CAAGGTTGCGGTACCCCCGAGTTAGATATTGAAACCGGTTTAATTTTGGATGCTGTAAG
GATATTTTTAGACACTATGAAAGAAGAAGCGATCGTCCAAGGCCAGATTTTAAGTTGTG
ACAGCGATGATAGCTGGAATTATGGAGCAGACATAATCAATACGATAAAGTTGGGCACG
TATGAAGGTTTGACTGGCCAAATCAGGTTCGGAGACGATGGCTTTAGAAATGCTTTCG
AATTAACTATTTATCAGATCAGAAGGGGAGTCGTATTGGAAAGAGGTAGTTGGAACAC
CACTTATGGTATTGGCGAACACGTTGTTTTAAATTTTGATGAGGAGGAAGTAAATAAAG
ACGAGGAAGATGAAGAACTTCGAAACAGAAATTTGAATGTTTTAATAACTTTGACTGA
ACCTTATGTTCGATTACGAGAATCAACAAAACGTCTAACAGGTAACAATAGATTCGAAG
GCTTTGCTATCGACCTCATAGAAGAAATTGCAAGAATAGTAGGATTTCAATACACTTTTC
ATCTCAGAACCGATAACCAGCACGGCCAATTCGACTCATTATCTGGAAAATGGACGGG
AATGATCGGAGATATTATTGAAGGGAAAGCAGATTTAGCAGTATCCGATTTGACCATAA
ATAAGGATAGAGTCGATCCTGTTGAATTTACACAACCGTTTATGGGCTTAGGTGTCAGC

ATTCTTTTCCGCAAACCATCAGTAGTTGCTCCGTCCTTCTTTTACTTCGCCCAGCCTTTC
TCCATAAGCTTTTGGCAATACTTGGGAATTTCGTACCTTATAATAGTCTGCTCTCTGTTTT
TGATCGGCAGATTATCTCCAAACGAGTGGCAAAGACCAGAAACTTGTAAACAGAGTAA
AAAGTATTTGGAAAATGATTTGACCTTATTGAACTGTTTATGGTTTGTTTCTGCAGCAGT
CTTTAGGCAAACAACTAATGTTAAAATGTATTCGATATCGGCAAGAGTAATTTCTGCAA
CATGGTGGTTGTTTTGCTTCGTTTTATTGGCGATGTATATTTCCTTCTCGCTTTCGAGGA
ACGCAATCGAAGAAAAGGAAAAACTCTTTGAGAACGTTGCGGAATTGCTCGAATACG
CGGAGGCTAATGATATTAAATTTGGTGCCAAAAAGGGAGGTGCCACTGAAGCATTTTTT
AAGAATTCGAAAAACGTATATTATCAACAAGTGGCTGAATACATGAACATTCACCCTGA
GGATATGCCAGCAACAACTGGCGAAGGAGTACAAAGAGCCATGCAAGGAGATTATGCT
TTCTTTATGGAATCAGCTACTATAGAATATACGATCAGAAGGCAGTGTAATTTGACCAGT
TACGGAGGCCTGCTCGATCAGAAAGGTTTTGGAATAGCTGTTAAAAAAGGTTCTCCGC
TTCTGACACATCTGAATAAAGCTATTATTAAATTACAATCTTCAGGAGATTTGAATATGC
TCAAAAGAAAATGGTGGGATGAAAAATATGCTGGAGATGCTTGTGACGACGACACCG
GTTCCGACATTGCACCGAAAACGGTGGATCACGTCAACGGATTGATCGCCATCACATT
CGCTGGCATAGCTATAGCTTTGGTAATGGCCCTTTTGGAATTTATGGTACACGTTTATCA
GTTGAGCAAAAAAGTAAAACAACCATACGGTAAAGCATTTTCCGAAGAATTGAAGAA
AAGTTTTAAAAGAAAGCACACCGTGCAAAACGTCGAAGCCATAGCTCTTACTAAAACG
GAAAATGGAAATAATTCCCAGAATAACAAAGAAGAGGCCTAATGTCGGTTATTAGAATA
CAGTTGGTTAAAGAAGTATCACTTCCGGATTCGTACTGTAATATTTTGAATTGTGAAGA
AATGTGATATCACTTCTTTAATTCAGTTGACCCGAGTATAAACTCAAATAAGTATTAGAT
TTTTGCCATCACTAGCTGGTAATGGTTTTCATTAAAAATAATTAATAAGTAGCTAATTAA
AACATAAAACATGTATAAAGACAACAAAAAAGTTTAATTCGTAGATTTTCTGGAACACA
TACTTATTCAGGATTTTTGTTGGTTAACAAATCCTAATTTATACTTTACACTGTATTTGAT
AAATACTAGTTTTGTTTACATATCGATGGTGTAGCGCACAAATGTAGTAAGTGCAAAAA
TGTTAGTTTTTCAGGAGTACAAATTTTAAGTTCCGTGTGGAGCAGTAATATATCGCAATA
TTTAATTATAATTTTATACAGGTTATGATTTTTAATTCATTTGTATC
>Cluster-12689.2279   MsigGR1
GAGAAACTCAATTTACAGTAGCTTAGTTGTAAGGACAACACCGTCATGGCTCTTTTAAT
TTCCTCTTCCACTTCAAACGTTAGCAGCTCGCAAATTAAACTTGACGTTAGTTTATGTTC
GTTTATGCAAATCTGACATGGTTCTACAATAATCAAAAGTCGTCCAATATGTCCCAAAA
GCCACACTGTTTGAAGTAATATGTAAAATATATCTGCGTCGCCGAATAAACCAGACAAT
AAAAAATATGGAGTTACGATTAAGTGAAGGAGACAGCTCAACATTATAACGTGTATACA
TAATTCCATGGAGTTATTTATAGTTGCTGTGGCTTCAAAGATTTTATCTTGAAATATCGAT
AAAGATATAAGCCATTTACCTGTTTCCATGGATTCATCACTTTTAACATAAGACGCAGGT
GTGTTAGCAGCGCTTCCAGTGAAAGTTTGATTAGTGTGATGTCTACTGTTAGTTGCGGC
GATCTTATCAAAATACATCTTGCCGACAATTTTTTCCAAAATTTTATTCTTGTTTAAAATA

GCCTTGTCTCTGATTTTCGTCAGATCCTTGTTTAACAACGAAATCCTTATTTTTATAAGG
AAAATTATGTGCCAATAAAATACCTCGATCATAATTACGATGCAGTAGAGGATGTAAAA
AGCGAAATAGCGTTTTACGTAAGCTATCATATTTTTTCTGCTGGCTTTTAAGAAAATATC
TGAAATGAGAATTAGGGCTATTGTCGATATCACAATGGCTATGAAGAGGATCGACGATT
TTCGTATTCTTTTCGAATTTTTAAATGGAATAATGTTGTCGGCCTGGTTTAAAATGTTCAT
CAGTTTCCAGAATTTCCTAATTTTCATGTGCAGAGTTATCGCTGCGAATACCACTATGAT
AATTATAATGCTAATATCACCGCAAGTAACAAATCTAGTTTTATCATCCGTCATTCGTAGA
GAATACTGTGGATCCGTTTTAATATCCGCTATCATTCCGTAAACCGCCAATAAAGATAGT
CCAACAGATAAAAAATAACTGTATGCTCCGTAAAAAATCGACCAGTTCAATTTGTAGTG
GTTACCAATCTTTTTATAGCTGATCGGAAACAATCCAAACATTCTCGATGTAATAATCAA
GGGTTGTATAATCTCAAATGTTGGTATCGAAATATATAAATCATCTACTATCATCGTTGCC
TTAGTGCTTCGTCATTTTCAAAATCTTGAGAGAGATCTCCTATAATATCCGCAATTTTGA
TATCGACTTGAAAGATTGCCAAGTACCTATGCCTTACTTGTTCCCTTTAATAATTGAACG
TCACATCCAGCAGTCGGTGGA
>Cluster-12689.31379 MsigGR2
TTTAAATTTTGTCTATATAACTTTTAGTTACAACAATATCTTAAAATAACGGATATTTTGA
AATAAAAACGTTCATAACATGTTTAATTTTTTATTTTTGCAAAGGTAACGATAAAGCAAC
AATTCTACATTTGATTGCTTAAACAATTTTACTATTTATTTTGTAATATGAAAACTGGCAT
TCTCATGGTGCAAAATGTTGGTTTTTTGCAATTTTTGAAGTCATTGGTCATAAACAAATG
AATGTGCAATACACTACAATAATAGCGCTACGTTCTTTAGGTGAAGTAGTAGCTAAACA
AGCTAAAATTGTTTTAAAAATACTACAACAGCGATTGATTTTGGTAGATTTACTTTAATC
TTAAAATATCAAAGCAATTGACTTCTGTTTTATTGCAAATAATAATAATAGATTCAATTTT
CATCACTACCAATAGTGTCGTGTATCAAATTTGAAAGCCCTGAGTCGAATCGTCTAACT
GATTCTTTACATCAGTGCACGTGGGTCTTTTTCTGAGCTACAGTTAATTTATTATCGAGT
TTTGTGCCATCAAGAATCGATATGTCTTTCAATCAAAGACAATTTTAGTTTTTTGTATCC
ACTTATTTTTACTTTTTTTTTTAATCCATTTTTGCTTGATTTCCAATAATTCTAAAAAATCT
GCTGTAGCGAAACTTGCAATTTCTTATTTTTTAAAAAGCCATAGAACAACTTCAATTGT
GATTACTCGCTTTATAGAATATTTCCATTTAGAAATAGTGTTATCATTTAAATAAGGAGAG
TTGGGAATAAGGAAGTAATTGTTTCAATGTCAAGTACTGACAAACAAAAAAATACAAA
TTTTGTATTGTCTTTTGGCTTACTTCAAAGTAGATCAAAGTAGACGTATTGCAATATTATA
CAAGTAAACTAGAATCTAAAATAAAAATACAAAATTTTGTCTATTCTTGTTCAGATTGAT
GATCGCTCATGTGTTGTTTTGAATTTTTGTATTCATTTTTAATCTTATTAAAAAAATTTAA
ATTATTCCAAAATAATAATATTGAAGTTACATTGACGGGTTAGTTAATAACTGAACAGCG
ACAATAACATACGATGTTATGCTTCCAATAATGAAACCCATCATGGTAAAGTTTACCGAA
AAAAATCCTGACGCCGAAAGACAAGGCATCCTATGTACAGCTTGTTGAATTAAAAACT
TTAAGTCTTCTTTGACAGCTTCAATATTAGAATGAGAATTCTTGTCCAAAGAATGAATG
ATTCCATAGCAGATGATAATTGTTTTGTTAGCTTCTCTTGATAGGTGCTCGCCTGCTATAG

CCAAAACTGATGTTTTTATAAGAATTTCTATTATCCAGATGATGCAAGAATTTTTTATATT
AAGTTTTAACGATGAATCAATGAATGAGAACATTGAAGTAGCTATTATTAATAAATTACT
CAACAATCCAACAATAAAAACAATAATGTACAAAACATAAAATAACAAAGTTAATCCA
AACATATCGTTGATTATGGAAACGATGCCACATAAATCATTATATACTCGTTCCATTATTT
TTAGCTCCCTAGAAACTTTCTTTATATTAAACACTGCCATTTTTTCGCAAGTTGTCATGG
AGGAAACATCGGCATAATTATTAATTAGTCGAACTTCCCTCTCAATTCTTTTATTTAACTC
CTTCAACCTCAGTTCGATTTCTTTAACTAACCAACAGCAAATACCTACAGCTATGTTGA
CTTGTATGTATTGAATATTTCGTAACACTATACTTTGATATTCTTGTGTAAATTCAACTTTT
ATCCAACTATAAGTTTCAAATATCGAAATTGCTATAATCTCCAAATGATTTATACAAAAC
AGAATACCATACTCTACGGAAAAAGTGGACATGTAGTTTCTTCTTAGTAAAGTATCGAC
GACGTCAAAGCACTTTAATATTTTTTCCAGTTTTGGCCGGAGAAATATCGTATTGATAAT
ATTAATAATGAACGTTAACGATAGAAACAAAGTAGGAGCCTGTTGAATTAGTTTGTAGA
AAAAATTAAACTGCTTCATAAATTCCTTGTTGTTCGCCTTTCTGTAAGAAAGATACGAA
GGTGCTAATATAAAAGTTATTAATAAAATAAATATGTAGCATTTAATGTATCTCAAATACG
AACTTTTCCATGTTACTCCCCAAAGAGAGGAATAAATAAATATTGCATTAATAAATTTAG
AACCACTGTGCGCGTTTGAGTTCATCGTGTACATACAATCAG
>Cluster-12689.11554 MsigGR3
ACAAATTAGAAGCGACCTTATTATGTTTCCCAAAAATAAGGTTTTATCTAAAGATTCGA
AAAACAAGTTATGTAACTTGAAGTTTATTTTACAATTATGTTCTATCATTGGAATAGCATC
AAAAACTCCTCATAATAAAATATACAAAACCTATGTTATAATCCTGGCACTATTTTCCGT
TGTTGGATCATTATATTCTGCTTATGGAAAAATGGCGTTTCGGGAAATATCTATTAGCATG
TATATTACTTTGGCTGATCAAATATCAAGTGTTTTTTTATGTTTGGCTACTGTTATTTTAA
GCATTACAGCGGTTTTTGTTTATCCGGAAAAATTTTTGGAAACGTTGAAAAGCTTGAGG
AACTTTGATCGATTACTAAATTTATACAGACCACGTTCAATAAAACAGTTCACAATCTAT
TTAATTTGTATTCATATAATGGTATTGCTACCCATTTTTCTTGACACTTGGTACTGGACAT
TTCTTTTTGGATTATCGGTCTATAAAAACTATTTAGTGAGAAATGTTCAATATTATCAATT
ATCAATAATGATGTTTCTTTGGTTTTGGCTGGTAATGGAAACTAAAAACAGATTTTCGA
GGCTGAATTGCATTCTGCAGGATATGGTTTCCACCCCTTACATTTTACTCAGAACCAAAT
TCAATATAGTTGTCTTTAAGACAAATTTTTCCAAAACGATTGAGACAATCAAACAAATA
TCTGGACTGCACAACGGGCTTTGTGATGTAGTAGAATTGATAAATGAAGTGTTTGGAA
AAGGGCTGTTGTGTTTTATCTTGTTTACAATTTCTTATATTTTGTGCTACACTATAGTGCT
TATTGAATTCGGAAATTATTATTCAGGAGGTGAAATTGGAAGATATTTACGAATTATGAG
TAGCGTGTGGATTATAGAAAATTTTATAAAAATACTAGCCTTAGCTTCGGCTGGTGAAC
ATTTGACAAAGGAGGCAAATAAAACTATCGCAATTTGTTACGGCATTAAAAAATGTTTA
GACCAAAATTATATGCACAACGTAGAGGCTCTTAAAGAAGAACTGGATTCCTTGATTCA
ACAAGTTGCACATAGAAAACCAATTCTATCAGCATCCGGATTTTTTGCCGCCAACTCTA
GCATGCTCGGATTTATCATTGGAAATAGAAATACTAGCCTTAGCTTTAGCGGGTGAACA

CTTGACGAACGAGGCAACTAATACTATCGCAGTTTGTTATGGCATAAAAAAATGTTTAG
ACTCAGATAATATGGACAATTCAGAAGCAATCAAAGAACTCGACTCTTTGATTCAACA
AGCGGCACTAAGAAAACCAATTCTTTCAGCATCTGGATTTTTTGCTGCCAACTCTAGCA
TGATGGGTTTTATAATTGGAAGTATTACGTCCTACGTTA
>Cluster-12689.19841 MsigGR4
TTGATATCGTGATAGTTTAACTTTAGTTCAATAAACATTCAATAAATACGATTTACGCAAT
AGGGTAACATGGATTTTCAAGTTAGGAAGAAAGGATTATTGAACGATATTATGGTGATA
AAACTCTTTTACCAAATTGGTTCTGTGTTAAGTATTTTCCCCAGAAATAGTAAAAGTGT
ACAAATCTTAATCCAGTTCATAGTGACTGGTTATACAATTATTGCCGGAGTTTTTGTTATA
TTCATGTACATAATAGGAGAAAATACCGTTTTTGGAAAAATACTGGAAGTATTAATTGTA
GGAACGTTTTGGGTTTTCTCTATAATGAATATGTATCAATTATTTTATAAGAAATACAAGT
TGAATCTGTTTTGCAAGTGGATATTGTATTGTGATAAAATCATGGGATCTGTCTACGTCA
ACAAAAACATAATATTTACCAGATTTATAATTTATACTGCATTACTGGTATCGGCAATTTT
TCCATTATTATTTTATTGCGCAACGTCTGCGTATTTAGTGAAGTGCTTATTTTATTGCTTC
GTATTTTGTCAGTCTGTTTATATAACAACTTTTATATTTGAAACTTCAATAGTCGTAACAA
AGAGGCAGGAAATGTATTACGAACGGCTGAAAAGTTTATGGAGATGTCGAAATACTAC
TAGGACCAAGTATATAAAAAATCTAAAAACCTTGAAAATTATATATAAGCAAATCTACTT
TATCGTTACGCAGCTAAACGCAGCCTTTAATGTGCACGTGTTACTGATTGGAACTGTGA
CGTTTTTGGAAATTCTATACAGATTGGAGAAACATTTACTCGGAATTGGTCCTATTCATT
TTTTGAAAAATATTTGTAATTTATTCATAACCTGTGCTCTATTAGGTCAAGTGATTATGTT
AGCAATAGCAGGAGACAAAATTGAAACGACTGGTCTTAACATCACTAACTTTTTTTATT
CCTTACACATGGATCTTAACGATCCTTTATTAAATGATTTATTAAGTCAAGATACAGTGA
GGGACTTTTTAATTTTCTTGGAAAAGCTTAGACCAACTTTAACAGTAGGAGAGTGTATT
ACGCTCAATAGAAAATGTATTCTAATTTTACTTTCCAGTTTAATCCCGTATGTAATACTTA
CAATGCAAATATTTTCACCAGTTTAAATAGTTCTGTGTCAGCTCTACTATTGGAAATTAA
TTCGTATTTCACAGTTCTTTTTAATACTAACTCATAATAGAAAAATTAGACAG
>Cluster-12689.28995 MsigGR5
AAACTTTCTAAATATTTAATAAAATAATAAATCATGAAAAGCAAATTATTTTGTTCCATTT
ATTTTTACCGTCACCTCTGCTTAAATAATTTATAGGTTGCTTTATTTTTGCATTTTGTTGTT
TAAATTGTAAAATATGCGTCGCGCCACCTTTAGGGTAGTTCATGACCACACGATGAAGC
AACTGAACTAACTACTCGATGTTTTTAAAACTGATTTTTTGGCTTCTTTAAACGTCTGCC
GGATTTCGATGACGCACAGTGTTGCTGTTTCGTAGCTCATAACAAAGTCAAGTTACTCT
ACAAGCCTTTTAATACCTAATTACACACTATTTCTACTAGACACATTTATTGTACTACTGA
GCAGTCCGTCAGTAATGGTGGAAAAAATTTAAGTGCCATTGTTTTTGAATTGCCTGGTA
CCGTGAGATTCACACGAAAATAAGTGCTTTGAACATTATGACGTAGATGCCACGAGATT
TTTCACCACCTCTACATAGATTCGGCATCAGAACATGTTTATTTTATTATTGTTACAAAA
AGGTTGAACTGCAATGAGCAGGCACAGCATTCTTGAAATGTTATGTAGTATTCTAGTTC

GTTTTGCAGACATATTTCCATTGTCTTCTTTTTTATTTGGAGACATTTACTTTTTTTATAAT
GAACTATTTATTTTTAAGATTTATATAGATCGTCGCGAAATAGATTTTAGCCGCTGTGTTA
AGAGGCATAATCAAGTCACATTTATTAAAATCTCTAATGAAAAAGAAATAAATGCATTA
CGGTGATATGTTAAACATGGTCAACTGTAGTTTTTATTTGTATCCATACTATAATATATGT
GGTAATATTAGAAAGTATAATGGGAAAAAATTTTCTATTAATTACGGTGACCCCTTCAAG
CATTAAAGATGGTTTCAGATTCTCTAAATATGTTGAGAATTCTTCCATTTTAGCCTTCAC
TAACAAATCTTCTTGCAGTAGAGACTTATTCTTACGGGAAACATTAAATAATTTCTTTAT
TCTTAGATCTGACGTTTCAATTCTGTCTCCAGCTGTAGCTAACATAACCAAAAGTATCA
GAAGCACAGGAATATAGAAAAACGGCTCTGTGTTACTTAATATGTTTGAAGAAACGTG
ATCTAATTCATATAAAAACTCGATAACAATTAAAGTGATGGTAAAAAACATGGTTGTACC
TAAGATTTTATTTAACTGCATCGCCAAGAAATATAAACTTTTGTAAATATTTTGAAGACT
CTTTAGCTCCTTAAGATACTCAATTTTATTTTGATGTTGAATACTTAAAAAACGCTTAAG
TCTACCATAAAGTATCTTTTGTCTATTTGCTATTAAAGTTGAACTCTCAAAAATTAATAA
AACTATGAGTACCATATGAAAATAGTTCACCGTATAATAAGCCGCAAACCAAATTGATCT
CTCTATGGTACCTATCCCAAATGGTAATAGCACGGCTACTAGAAGCAACGGTAAAGAAA
CAATCCTTCCAACAACTAATTTTTTTGAAATGCAAATAATGTTCAAATTTTTATCTACCA
AAGAAAATACGCAAAACAGTTTTTTCCAAGAATGTCGATAGCGTATTAATCGATAAAAC
TGTGTAATGGCAAAACTCATGAAAAATCCTAGATCGATATCGTTAATAATATTCGAGAAT
AAATCTTTTTCAATGAAAATTGATAAAAAAGTGGTGTAAAATCCATCGGCCAATATAAG
TATCAGTAAAATATATTGAAATAAAATCTCTCCATTTTTATAATTCCAAGGGAAAAGTCC
CATTACGGCTCCACATTTGTAGAATAATAATAACGGCAGCGTGTCCTTGGCCCCGCTCTT
ACGTAAATTGTTGAAGTACAT
>Cluster-12689.8671 MsigGR6
GTTCTGGCTGTTGGTTCAACCCAATCTATTTAATTGTTACATCTATGAATGAAGCTAATG
CTATTTTATAAATCGTCACTAGTGCCAAAACTTAAAATCCTTTGTAGGTAGGCTAAATTA
AGAAGTGAGTACCATATTAAATTGTATGATAATCACAAGGTAAGTGACAATTGCTGAGA
AAATCGATGACAAACAAGACTGGTTTATTTCATAAAACCCAGCCGCAGAAAACACTGG
GCGCCATTGTTGAGCGTACGTCTCCACAAGAAGCAACTGTTCTTTGTTAGCTTCGTCGT
CGAGGGATTCGTAAAGAAGATTACACGTTCTTATTACTTGATTTCCGCTTTTTTCTACTG
CATCACAAGCCATTATCAAAATAACCAACAACACCACAAACGGAACGGGGTAGATGTA
TTGCGATAATTTTAGTCCTAGATCACTCGGCGTCTGATCAATCGCCAGCACGGTCAATTC
TAGTACGATAAAAATAAATAAACAATTGTTTAAAAATATCGCAGTACCAAATAATTGATT
GTTCAAGTCTAGGACATTCTTGCCGAGTTCAAGATATTTGATAATTTCATCGCATCCATC
CTCAAATTTTAGGTTTTGTTTTTTACACAAGTCGATAATCATTAAATTAAAATTGTCGTAT
TTGTTTGTTAGCCAGTTTGCAATAGCAATATAATATGTAAAAATCAAAAAGCTGCAAAT
GACGGCGACAATCTGCGTGATTAAAACTATGCAAGCATTTATATTATTGTTTATTATATGG
ACATAATATGTTAGGGAATATAAAAGTCCAAATAACGTTAAAACTACTGCTAGTCGAAG

ATGTAAATAGTGGCTATTTTCTTTTTGACATTTCCTTAAGGTAATTTCATTTTCTAGCTCG
TAAATAGAATTAATTAAATTTAGTTTGCATTTTTTTGCAAAAAAAGGAAAATGTATGAAA
CAGTATAAGACATACGCTATTTCAAATACTCCCTCTGCAATTAGCATTTTGCCCAAAACA
ACACTAAATCCATCTATAAAAAAATATCTAATTATTAAGGAGTAACAGCTTACTGGTAGA
AATAGACAAATTAAAATAAAGGCGACAAATTTAAGGTTTCCTGCCAAAGGTCCCAAGG
CAAAATATTTTATAGCTTGTAATGAGGTGACAACGTATTTAAAATCCAATTTTAAAGATG
TTTTATTAACTAAATCGTCGTTTCCAACAATATTCATGTTTGTAGAAACTACGAGTTAAC
TTTAGGAGTCATAGAGTGATT

>Cluster-12689.7908 MsigGR7

GATAACATGTTTTGCCATTATTGTATGTTTATATCATGTATTTATAAACGGTTATGTGTTTT
CTGATGTAGCAATATATGTATTTTACATCGGCTCGTTAGTTTCAATGATATTATTTTTGCA
ATTGTCAAAAAGATGGTCCCTTTTGATCAATACTTGGTGTCAAATGGATAAAATTATGA
ACACAAGGTACGGATATCCTCCGACATTAGATACACGATTGAGGATATGTTCAGGAATT
TGTATAAGCCTTGGGTTAATCGACTACGTCCTTAGCATTTACAATCGTTGTATAAAAATG
ATGGTTTTATATGGTGACCACAACGAATACAAGTATTATTTCAAGGATCTTTTTCCTCAG
CTTTATATTGTACTACCGGTGAATATGGTAACGGCGGTATACTGTTTGTTTCTAGCTACAC
ATGCAACGCTTATAGAAGTTGCCAATGATTTATTCATAATATTAATGAGTATAACATTGGC
ATTAAGATTTAAACAGATTACTAATAAACTGGAAGAAAATTTACTTAAGATGAAGAGTG
AAGACTTTTGGATTGAAATTCGTGAAGACTATGATCGACTGAGTATACTGTGCAAGGAA
TTGGATGATAATATTTCTTACATAATTTTGCTATCATACACTCAAAATCTATTTTTTCTGCT
AGTACAATTTTATCAGAGTTTAGAAGGCGTATATGGCCTAGTCGGAAAAGTTTACTTCG
TATTTTCATTCGTTTATATAATATTCAAGATAGTTTGTGTTTCGCTATACGCCGCTTGGATT
AACGATGAAAGCGTTGAACCTGCCAATATCCTTAATTCTGTTTCATCGTCGTCCTACAAT
GTTGAGGTTAGAAGATTGTTGATGCAAATTAGCTTTGATAATGTTGCACTAACTGGTTG
TAGAATGTTTAAAATAACCAGAGGAATAATATTGAGTATTGCAGGTGCTGTCGTAACTT
ATGAATTAGTGCTTATACAGTTTAATTCTGCGACGCAAGTGTCGACTCATAGCGGATTAT
AAGTTAAAATGTTCAAATGATT

>Cluster-12689.51891 MsigGR8

GACGACTTTACATTTTTAATTGTATAAGAATTATAGCATATGTTGTAATAGTAGTTAGAAG
TGCTGGTACTATATTTCTATTTAAATCAAAAAATTCTAAAGCTGTTAGCACAGGTCGAAG
TTGTTCTGTAAAAATCGATAGTTGTCTTAAATGATCCCGAATTTCAATGTTCTCTGCTTC
GCTTTGTAGCTTGTAACACAGTTTTGAAATCTTGATTCCCATTTGCTCGATTTTGTCTCC
AAAAACTGCTATTGAGACAGATATAACGCAATATAAAAACAAAACCAAAATGGAAGCC
AACAGAAATCGGTTTGCAAATTCTGTATCACGTGGAGAGAGGTCCAAAAAACTATTAA
TATCATTAAGTATCTCCGTAAAGCAAAGTATCAATAACAACAAAATGAATATACCAAATA
TATCATTTACTTCGTCAACTATAGAATTTATATTTATATAAATATTTTTAATATTTTTCAATT
TTCCTAAAATTTCACATTTACCAGTATAACGAAACTTGCAAAAATTTATAAGCTGCAAA

CAAAATTTCTCTTGCCTGGTAGCTACTGCCAATAAAATTTCTACTAAAAATACGGTAATA
ATCACCATTTGCAGACCTACTATATACATTACGAAATCGAAAAACCCGGATTCTTCACCC
AATAGGGGAATCATAGAGAAAAAAATGCTTAAATAACTTAAAGACCAAAATATTATCTT
GCCAAAAATTGTGGTTTTTTTAACATGATTTTTTCCTAAAATGTGGTCAATAGATAAAAG
GTACTGAAATATTTTTCTAAACTGTTTTCCATTTGTAAATAATCCACGAAAAAGGAAAAT
ATTAAATAGAACGTAGATAGGTAAGTGTATCATTTCCAGACATACAGATGTAGTGGAATA
ATTTTTCTGATAGTTCTGTATGTATGTTAATGCAACAACAAGAATATTTATAATTACTGCA
ATTACTACTTGTAATCCACAGCTACCTTTCTTTAAATTCCACGGAAACATTCCTAAAATG
GCTCCAAGTCTATAACTCCACCGTAACAATACTGTATCATATGGTAACCGCTCTTCAAAG
>Cluster-8951.1 MsigGR9
GTTTTAATGTGTTGGCGGTATGTTAAAATGAGTTCTTTAAACAATATAGAATTTGAGACG
ACAGATACTTTTCTATTAAAACTCACTTTCAAATTTGGCAAAATAGTAGGAATCTTTCCA
ACAAATTCTAATAAAATTGCACACTACGTTCAAAACTTCTGTGTGACCACATTAGCTGT
GTTGGGAGTAATCATAGTTATGTTATTTTCATTTTATTATGACGACAAACATTCTGTTATT
ACAACCGTTCTCTTAATAATGGATGGGACAGTATTAATGATTATGAATTCGGTAACAACT
TACCAATTTTATAAACATAAACATATTTGGGATTTACTTTTTAAAATGATTTCGACACTGG
AGAAAAACGTAGGAACGATTTATGTGTCAAAAATTTTAATTATTCTTAAATTACTATTTT
GGTGGTTAGTGTTATCAGTTAGTGTCTATTCATTTTATGCTTATTTTGCAGTTTGGCAAAT
TTTCACGAATATATTTACAACGTATGCTGTAATCGTAAACTTTCATACCACCATTATCACT
GTTTTTATTTATGAGGTATCAGATTTTCTTACAACCAGACAAGACGTTTACTTGAAAAA
GCTTGAAAGCTTTTACTTGAACCAATCAATTAAAGGTGTCGAATTATTTAGGAGTTTGA
AATATTTAAACCAAACATATACACATTTTCACATCACTGCTAAATGTTTGAATAATTTATT
TGGTACTAAAATTTTATTAATATTTATTCAACTTTTTCTTGATGTCTTAATTGCATTGGATG
GAGAGATGAATGATGCACAGGACAAATTTAATTCTGGAAACGCCACGGTACAGACCAT
GGACAAATCTAATGCTGGAAAACCCATGGTCTGTGACATTCTTTACCTAATAGTGTTATG
TATTTTGGCCATCACTTTGGTAATCGCTGGAGACAAACTTGAGTTGTCCGGACTGAAGA
TAACATTTTTATTCTACACATTACGGAATAAACTAACCCCTCATTTACATGAGGTTTCGTT
AATTAAAGATCAAATAAAGGATTTCCTGAAATTTTTGGAAAAATTAAGACCTGAATTAT
CAGTCGGAGGCTACATTATCCTTAATAGAACAGTGATGCCATTTTTATTATTTAATATTAC
TTCTTACATCATTGTGTTAATTCAACTCAAAACACCAGATTTTAAATGATTTATTAATTAA
TATTTTTTCTCGATGTTCTCCACAACGTTATTAGTCTTATTTCTGTCTAGGTTTCCTTATT
GTTTTCAAGAGCCACTGTAGAAACCGTACTGTTAAATAGAATTCAACACTAAATGGTCG
GGCTCTCCAAATTAATTTATTTCTTTATTTGCCGTAACTGCATCACATATTAGTATTTTAC
AGCTCCCTGTTAAGTTTTTAGGTCTCAGCAATGAAGACTTTCTTAAGTGGAATGTGCAT
ATAAGTATCTTAAGGCTTACTTTTCTTTGTTTGAAACACACGTCATGGGTTACCTTTTGA
GGCTTTTCCAGTAAAAGATGGTCCACAAAATAGACCTAAATATCTGAAATATATTTTAAG
ACAATATGGAGTTTAAAACCAAAGTAAAAGATAGTGTTGAGTGGTAAGAGCCGAGAAC

TGGATAGAGGTGGGTAATAGTTTAAA

>Cluster-13832.0 MsigGR10

AGCAAATCTACGGTGTTTACAACATCTTTTAAATTATTGTAGTATTTGTGGTTTAACAAA
TGGTTTTCCCAACTCCATATTGTTTAAAATATATGTAATATGCCTCCTATGTGTTTCCACG
GTGGGATATGGCATATCCGCCTATATGAAGGAGACGCCTTTGTTTGCAGAATTAAATCTG
TTTTTACGACTTGCAGATCACGTTTGCAGTCTGTTTTTGTTCTTGGCAGCTGCTACGAC
CACAATAAATATGGTATTCATACGTCCTGGCAAACTTAGAATAATTTTCGAAGAAATAAA
CAGATTCAATAATGTTTTAGGAGGAAATCTTGGAGTATTAAATTTGGGTTGTTTTCTATC
TATATACCATATCAACGCAGCGATACTTTTAACCTTTGATGCTTATTCGTGGATTATTTCA
GTAACTTTTGAGACGTTTAAGTACTATATAGTTAGAGATGTACAGTATCACCAAATGAGC
ATATTGATGTTTTTATGGTTTTGGTTAGCTAAGCGGATAGGAAAATGCTTTGAGAGGTTA
AACGAGATACTGAATGATATTAAAATAAAACGTTTATGCAGAAGCAGCAAAATCGATTT
GTATAATAAACATACAGATATTTCATTATTTCCGGATGTCGAGGAGCGGTTGAAAACCTT
GGCAAGAATTTACAACGATATGTGTGACGTAGTGGATTTAATAAATGAGTCGTTCGGTA
TTTCCTTGTTGTTTTATGTAATGTACACAATATCGTTTGTTGTTTGTTATACTATGGTTTTG
ATAATCCACTCATTTGTTGTGGAGAATCCCGATTTTGATATCAGCAAATATTTAAGAATT
AACAGTTTGTTGTGGATTTGGGATAATTTTATTAAAATATTGGCTTTGGCATTAGCAGGC
GAGAAGTTATCAAGTGAAGCGAATAAAACAATAAACATTGGTTATGGGATCCTAAATTG
TTTGGACAAAGATCCTAATAGAGTCGACCCAATCAAAGAGGAATTGACCTTTTTAATTA
ACCAAGCTGTGCACAGGAAACCTTGCCTTTCAGCATTCGGATTCTTTATGGCAAATTCG
ACCATGATGGGTTTCATTATTGGAAGCATTACTTCATACGTTATTGTGGCTGTTCAGTTT
TTAAAGTAAACATTAGCACAAACATGTAAATTTGTTTAGGAATTTTTGAAATGTATAATT
TAAGAAATCGTTCTTTGAACTAGCCTAGCAGATTTAGACAGTTATTTTGTTTTAACTTAA
GTGATCATGACTTACCGCAGAAATATAGGTACATTTATCAAATGAGAGGTGTCCAAAAA
AATTTTTGAATTATTTCTAAACTTAATACTACATAA

>Cluster-12689.7473 MsigGR11

AAGTATTGCTCTGTCCTAGAATAGTCAGTATAACTATAATAAAAATTTAGGTTTTCTATTA
AAGACATTGGTGCCTCAATAAATTATTTTTAGTTGGAAATAACTAAAAAACTCTTCCATG
ATATCTACATTAAAAGATAAAAACCACAAGATGCACACCACAAAAAAACAAAATGATC
AAGTTGAAGAAAAATACGATTCTTATTCTCTTCACCATGTTTTAAGATGGTTATTTTTTAT
AATGCAAATAGTAGGATTTATGCCCGTCCAAGGCGTATTTAAAGATCGTTCGTCGGACA
TTGGTTTTTCTTGGAAGTCTCTAAGAACCCTGCATTCTTATTTAACCGCAATTGGATTAT
TCTTTATGACAGCAATTCAGATTTCCAGATTTTTCCTATTTAAAGTTCAAATGGTTGACG
TGCATCGACTTTGGTACTTTCTCAAAGCTTTTTCTATAAGCATATTGTTTATATCTTTATCT
AAAGACTGGAAGGAATTTTTAAAAACTTGGTATAAAATAGATACAGCTATGCTGGTATT
TGGTAAACCAATATCCGTTAAAAGAAGAGTTCGATTGTTGTTAATCATTTTTTGTCTCCT
ATTTGCAGGAGATTATGCTTTAATACAACATCAAAGAGTCGCAGCTGATATTAAGAAAG

GGCACCTGGTTTTGACTTTTAAATCCTGGAATCATATTATCCGAGATTTTCGATTTGCAT
ACGTATTTCAAATAGTTCCTTATAACTTTGTAGGTGGAATTTGTCTATTAATATCACATCT
TCAAGTACTCTTTGCAGGAACCTTTTTAGATATATTTCTAATGATTATAAGTCTCTCTTTA
GCTGCAAGAATGCAAGTAGTGACAAAACGCATACAACAAGTATCTAGATGTACCGACG
TTCCACAGCACGTTTGGATTCATGTCCGTGAAGGTTACAATCGAATGGAATTACTCTGC
AAGTATGTCAACTCCAAAATTGGATACGCGATTCTAATAAGTTTTATAGGAAATTTAGGA
ATACTGCTTATCCAACTATATAACAGCTTACATACGAAAGATACTCTTATGCAACAAGTA
TACCTATATTATGCGTTCGGATTTTTGATGTGCAAAATGATTTCCGTTTGTATTTTGGGAT
CAAAAATTAATGACGAGAGCAAAAAGCCTTTAAATTACTTGTATGCTACAAAAGATTCA
GCCTATAACATAGAAATTGATAGACTAATTCGTCAAATATCTAGAGACAACGTAGCCCT
GTCAGGAATGAATTTCTTTCAACTAAAACGTAATATTGTACTAAAGATTGCTGGTTCCAT
CGTTACCTACGAGTTGGTTCTCGTCCAGTTTGCCGGTGACTTCTTAAAAGAGCACGAA
ATCGTAAATAGAACTACTGTCCTGTAAGATATAGATTTATTTTAATGTTTACAACTAATAC
ACAATATGTTCAAGAAACAATTAATAAAAAATAATTTTAATCAAAATATGTTTAAGATTT
ACGTTTCAACATTAATAAACTATAAATATATTAAAA
>Cluster-12689.45327 MsigGR12
GTAATTTAGTTAAGTAGAAAAAAGTTTGAAAAATCTAAAATGCTCTCGGGAAAGCAGA
GCGTCAGCCCATTTCCAAGAAAACCATTTGTAACCAAAAGCTTTTTGTTCACTTTATCT
AATAACGACTATAAACTGTTAAATATATATTTAAAGGTTGGGAAATATTTTGGGGTCACA
TACTTTTTTCATGGCACAGATATATCGGTTAGCGGTGGTTCCTATTTCAAGATAATTGGA
ATCATATTTTCTGTAATTTTCGTATATTTTTGCTGTTCCTCTTATAAAGCTCAAACCACTTT
AAATCGGGGTTTGCATCCCACATTAAAGGGCATGGCCACTGTTTTGGTAGCAGCAGAA
ACCGCTTTTCTTTTGTATTACGTCGTTTCTTTGTTATTGAAAAGAAAGAAAACGTGGCA
CAAGCTGTTCGAAAGAATCCACACTCTGGAAAATATTTTAAATTCTTGCGAATATTTCG
GCAAAGAGAAAATATGGAAAATCGCTTTTAAAATATGTTTCGTTTGTATTGCTGTACCG
CATTCCCTCTACAGATACGCAGACCGTATGAAATTTTGTGAAGCACCCTAAAACAGAAC
TTTCCTGTGTTCCCGAAAGAACCCAACTAGCGCCTATTTTTTCTAATTTATTTTTGTTAAT
GTTTCACTGTTTCACTTTTGCCTGTTGATTTAATAACACAGTTGAGAATATCGATGGGTG
TTTTAAATTTTAAATTTGATAAATTATGTAATTGGTCATGTCTTAATCTTCTTATACTGAAT
CACAATAACATATTTTTTTTTACGTTTGTGAAGTCATCAACGGATTCAAGAACAGTTTTT
TCTCTCAGAGCTCTAACTAATCTCTATATGATATTGATAACAACGTATTATTTGGTTTTAA
CAAACTGGCTATTCAACAGATATGAGTTCATGAACAAATTTTTAAGAACAACGTTCAAC
AATAAACGACATTTTTTAATAGTACGAAAAGTTATTGTATCTTATAAACTGGCGAATACC
GTAGTCCAGATCACTAATGATTTGTTTGGTAGTATTTTGTTTGCCAATTTAACGCTGTGC
GTATTAAACATTTTATATTATTTCGTACTGGCCTTGGATTTGAACAATATTCCTATAGAAC
AAAAAATAATAAATTACACAGCACCTTTATTGTATGCAATATTCTTGGGGACTGCGACA
ATGTCCTGTAATTCTGTGGAAATAAGTGGACATTCTATAATAAAAACCTGTTATCTTCTC

CACGAAGGTTTAGAAAATGATGTCGACAAGGATCATTTACTACTTTTGATTAAATATGC
AGAACTCTGGCGTCCAATTTTTACAGCTGCTGGTTTCTACGACGTCAACCAGTCTAGTT
TATCATCTATTTTTTCTGCACTTATCACTTACTTAGTTATTGTGATACAGTTCAATATGGTT
CTTTCTTAGTTGAAAATGTATTAGTACAATCTGTAATTTTTTAGATAAGATCAAATAATTT
TGATTCGATTTGTCAATCCTCTAAATAACTTTTACTATGTAACTTTCAGAGTTCCACAAA
TACACAAAATAACTCTTAGTCTTTACACGTTCCTTCTTTATGTATTATTATTACCAAGGTT
CGGAATTTTGCTGCTGTCAACAGTATCGGATATTGAATAGATGACTTTATAAAAACCCAT
ATGTTGGGAAACTTCTGAGACTAACCCAGCGGTTCTCAAACGTTAGCTGTTCGCGCCC
CCTATTGTAATTCAAAAAATGTTTGCTTCCTCCATTCGTAGGAATTCAAATTGAAATTGC
ACTTTTATTTTTTACGTTTCACGAATGTCAAGTTCTTAGAAATGACAACCAATATTCAGT
TGGGTATAGAAAGTAGGAAAGTAAATTCCTCAAAATTGCTTTGACAAAATAACGCAATT
ATATTTGTTTTTGAGAGTTTGTTAGTTTTTGTTCAAATAAAAATATTGAATCCTTTTTTTA
TGATTTTATTTATTGATAATAAACATAAAATAATATCACATCAATTAGATTTTTAAATAAAA
AAATATAATATTTACAATTTT

>Cluster-12689.19188   MsigGR13

CGCTCATTTTGAAGTTGGCATGTCCGAGAGAATTTGTAACAAATATTTAATAAGAGACA
CTTTGCTGCTAAGGGTATCGTTCAAAGTCGGTGCTGTCTTTGGACTTTTCCCATGGAGT
TATTCTAAGGGTGGAGTGAAATGTCAATCTATAATTATGATACTAATAACTGGAAGTATAT
TGATGAATATATATATACATGTATCTTCGGGACGTGATGCAGTGAAAAGTGCGTTAAATA
TAATATATTTTACAGTGCACTTATCCTTCACAATGCATAATTTTTACAGGTTAATACATTG
TAGAAATGCATGGAAATATACATTTGACCTGATTGATTCTCTAGAAAAAGATTTAGGAG
TTACTTATACCTCGAAACCATATTTTTACTTTAAGAGCGTCTTGTATCTTTCGATTCTAGT
ACAATTCTTATATCCTGACCATCCCGTCAGTACGATATGGATGGTTCCTAATGCAATTAA
CTTCATATTTGTACATTATCACATGATTATTATTACTGTGTTCATTTTTGAATTTTCTTCAG
TATTGGTACGTAAGCAACAAATATTTCAGAACCATTTAAAGATCCTTTGGAAGAACAAA
TACGATACGAGAGTGCAATTTATTAAAAATCTTAGAACTTTAGAAAAGACTTATCGCAA
CTTCTATTTTCTTGCGGACCACATTAATGCGGATTTTAATGTATCAATCTTACTAATTGTT
GTTAAAGTTTTTTTTCAATTCCTGTTTGCTCTGCATGATAGTTTTAAGAATGTTTTCAAG
GAGCGTACCGTTGAATACGGTTCCATGGTCGAGAATGTTCTTTACATAACAACATTATTT
ATTGTATTAGTTACTTTAATAATAGCTGGAGATCGTATCGAGTCCAAAGGATCAGATATA
ATCGAATTATGTCAAACACGTGATGAAGGGAGTCTCACAGAAAATTCTTCCTGCGAAG
AGCCAGTTGAGGCTTTTATTGATTTTCTAGATAAGCTTCGACCTGGTTTGACAGCAGGA
GGATATATGGACCTCAACAGACAATTAATATCAGTTTTCGTCTTTACTCTAACAACCTAT
TGCATTATATTAATACAATTATTAACTTAATGAAATTCGTAACTTGTTAAGGATTGGACGG
GTAAAGCGACCATGTTGACAATAGCTGGAGAAAGAATTGATACGGCTAGTAGTAAGAT
AACCAATTTTTTTTATTCGTTACACATGGACCTTGACGATCCTTTATTAAATGATTTAATA
AGTCAGGATATAGTGGGAGATTTTTTAATTTTCTTGGAAAAGCTTAGACCAACTTTAAC

AGTAGGAGAGTGTATTACGCTCAACAGCAAATGTATACTGATTTTACTTTATAGTTCAAT
CCCGTATCTAATCCTTCTAATGCAAGTATTGTCAGTAGTTTAAAATAGTTTAGTGCAAGT
TTTACGCTTGGATATTAAATTGTTTTTCACAGTTTTAACTACAAACTGATACTAGAAAAA
TCAGTTAGCAAATAATGATTTCTTTCACAATTGACCTCAATTTGCATCTTTAAGCTTTGT
AACAAAAATGTTAATCAACTTTTTAGAAAAATAAAGACCCG

>Cluster-12689.15094 MsigGR14

CTACATTTGGTTTCATTGCTGTAAACATGAATATACAAAATACAAACAAAAATGCGAGA
AAAGATACTGTAGTTTTAAAAATTATTTTTAAGACCGGCGCCACTTTTGGTCTGTTTCCT
TGGAATATTGATGGTGAAATCATATGTCAATCAATATTTATGGCGTTTACGTTAATTTGCG
GCACCTTCATTTTGTTTCAGACTTTAATGAAAAATGAAAAACCACTTGAAAAAATATTA
CGTGTGTTATGTATTGCGCCTTATTTTATTTTCGCCTTAATAAACTCTCATCGATTACTGT
ACCACAGAAATGAATGGAAACTCTTTTTGAAATTGTTATCATCTTTCGATAAAAAGATG
AGCACCACCTACATTTCAAATAGAACACTTTTTATGAAACTCTTTTTCCACATTCTACTA
CTAGCTATGATGGTATGTTCGTTGGCCGTGTTTTACGATGACTGGTCCTACGTATTCGAT
TTTTTTTTCATTACTGAAAACTGCCAATCTGTTATCTTATCTGTGTTTATTTGGGAAATTT
CCTCAGTTATAACAAATAGACAAAAAGTGTTTTATAACCGACTTAAAATGTTTTGGAAA
AATCCATGGATTAACAAGGTAGTATTTTCCAAAAATCTCAGAGTGCTGGAAAGCAACTA
CAGAAAACTTTACTTTTCTATTAGCTTTGTACAGAATATATTTGGCGCTCACATATTATTA
TCCCTGGCTCTTGTTTTCTTCGAATTTTTTTTTGCCGTCAATCGGAATCTCAATTTAATTC
TCAATGGATTGCCGATTAATGTACCAGAAACTATGTCTGACTTGTCTCATAAATGTGTTA
ACTTAGTGAGTATACCTAAATGATAATGCACATTTTTTTAGTTGCCAACAGATTCTCAAC
CATTTCATACATAATCTTCAGTTGCAAGAAGGTATTTAGGGACTGAAAATTCAGTCCGG
TGAACCATTTGCATATGCGTCAGCAGATATACGTCAATTGTCAGTGCGAAAACGTGAAA
ACGTGAGGTTTTTGCCTAAAAACGATGGGCGTGAAAACGTAACACTTTTGCATCACGT
GTGTAACGTTTGTGACGTCGTGGTTTTATGTAACAGCGTCACGCACATGCGTAATGCAT
ATGAGTCACGCACTTCATACAAACATGTCTGTTTTCTTGTCGCACCAGATATCGGATGC
ATGCAGAACTTTTTCCGGGTGTATATCCAGTTGTGTATTTAGGGGCGACTTCATATTTAA
AACATCATTGATGTCAAGTTGAGTAGCATTAATGGCTTTATCTATAATTGAGAAATTTTC
GTTGTTTGCTTAATCGAGGGGTGATTCCCAAGTGATCACAATGGCGAACGGCGAAATG
GCGTCGTGTTTTATCCACGTAAAAAGTATTACAGCTTCTATATTAAAACTCGTAACCGAC
GTTCGATTTCATGTCACTTGACAATGAATCATTGACATGGAATCGGGAATTTATTCTTTC
TAAAGTTGTAAACACAATAAAAAATTGACCGTAGAAAAAGCTTGTTTAATAACACGTC
TAAGAACTCGACGGATATGTAAACAATGTTGACCAGAAAAAGAAAGTTTTACATAAAT
TAGTAATTTAGTCTCTTGATGCGAAGGAGTTGTATATTTTCGATTGAGAAATTTTCCAAT
GCAATTGTCAAGAACTCTTCTGGTAAACCATGCTTTTTAAAAACTTCTTTGTTTCTTTCA
AGTTCTTCATGTAAAAGTTTATAGCTTGAACAAAGTGTGTAGGCCCTATCGACCAGGAT
ATTTATA

>Cluster-12689.18120 MsigGR15

ATTAAGTTCCTAACTAGAGCCAACATGATGGTCACAAATTTGAAAAAATCTTCGAACAG
TGACACAATGTTTTTAAAATGGTATTATAAGTGCGGAGTTATTTTTGGAGTTTTTCCGTC
AATTTCTAAAAAAGGAATATTTTATTGCCAAATTCCTCTACTGATAATAATGTGTGCACT
TATTTGTTTAACCAATTTTCTATATGAGCGATTGAGATGGACAAATCATATTGATCTTTCG
TTTGTTCTAAGAATAACTAATATGGTGATATTATTAGGTATGTTTTGCTCAAGTATTAGGA
GCCTACTTTTGCATAGAAGAACATGGAAACTATTTTTTTCTTGTCTGGTAAACATTGATA
AAGTGTTAAATGACCAATATTATAATACGAACAAAATTATTTTCGGAAAAATATTGTTAT
GGGTATTTGCATGGATAATCAATGTCTGTTGTTACGCTCCTTATTTTCTTCATCGCTCATT
AATTATTTATTTGGCTTTGGTATATCATGTGGTATTTACATTTGAATTGTATATTATATGCGC
GTTTTTATTAGAGACTTCAATACTTCTAACGAGAAGAGAAGAACTATTTTATACAAAAG
TTGAGAATTTTTGTAATAAGAATGATAGCAAAACAGAAAATATCAAGACATTTATTGGT
GTATGGAAAAATATTTATTTAATTACAAAACAAGCAAACCACGCTTTTGGTTTTTCCATG
GTTATGATTGTGATGAGTTACTTTACAAATGTTTTTATCAACATTGAAGATACTTTAGAG
ATTTCATTTAAACATGATAAAATAAAGTTAAATATTAATGAAACGATTTTCCATGTCATAT
TTGATATAATTAAACTTTCTACTATTGCCGTTGTTATTGCCACTGCTGGTGATAAGTTAGA
AAAAACTGGTTTAAAAATAAGTAAATTATGCCATCTTCTTGAAATAGATGTCGAAGATA
AAGCAATCAAAGGAGAAATGAAGGATCTCTGCAATTTAGTAGATCAACTACAACCAGT
GCTGACGGTTTCAGGATTTTTTACCATTAACAGAAACATGATACCTATGTTAGTAACTAG
TCTTACGTCGTATGCTATAATACTTATACAATTAAAATCCTAAAAATAATAATAATAACAA
AATAGTAATAAATATATTAATGTTGTAACTAGGAGTAAGCAGTTCATCGATTTAAAT

>Cluster-12689.8145 MsigGR16

GTGAATCTGTTTTATTTGCTGAAGATGTTGGTTTATCTAAAGTCAAGAAGAATAAGTTA
GTTTGCCTGAAGTTTGTTTTAAAGTTATGTTCGTTTATTGGAGTGGCGTCAAACAATCC
CCACAACAAAGTTTACAAAACCTATGTGATTATCCTTACACTAAGTTCAGTTATCGGATC
AATATATTCTTCTTACAGAAAAACAATGACGCACTTTCGAGCTGAAGTAATATACATCTC
TGTGGTTGATCAAATATCAAGTATTTTTCTATGCTTAGCAACTGTAACTGTAAGCGTCAC
AGCTGTTTTTATTTATCCAGGAGAATTTTTAAAAACGTTGGAAAGTTTGTGGAACTTTG
ATCAAATGACAAATGTTTGCAGACCCTGTTTAATTTTTGTAATTAATTTAATTTTTGTGC
ACGTAATGATACTGATACCTATTTTTGTTGATACTTGGTTCTGCATATTTAATTTTGGTTTA
TCTCCTTATGCCAATTATTTCGTAAGGAATTTTCAATACTATCAACTATCAATAATGATGT
TTCTTTGGTTTTGCTTAGTAATGGAGATCAGAGACAGATTTTTGAGAATCAATTCCAGC
CTCGAGAAAATGGTTTCAACACCTTATATTTTACTCAGAACAAAAATGAATACAAATAA
TTTTAAGGCAACATCTTTCCCCAAAATGACTGAGATGATCAAACAAATATCTGGACTGC
ACAACGGACTTTGCGATGTCGTGGAATGGGTAAACGATGTGTTTGGAAAAGGATTAAT
GTTTTTTACTTTGTTTACAATTTCCTATATTTTATTATTCACTATTTCGCTTATTGTATACGG
AAAATTTTATTCAGGAGGCGACGTTGGCAGGCAATCACAATATGTTAGTGGAATTTGGA

TATTGGAGAATTTTATAAAATTGCTAGCCCTAGCCACAGCAGGTGAACATTTAACACGA
GCAGCAAATAAAACTGTCACTATTTGTTATGGAATTAAAAAAAGTTTGGATCAAAATTG
TTCTGATGACACAAGAGTCGTAAAAGAAGAACTAGATTCGTTCATTCAACAAGCTGCA
ATTAGAAAACCAATTCTTTCAGCTGCCGGATTTTTTGTTGCCAATTCGAACATGTTGGG
TTTTATCATTGGAAGTATTACGTCTTATATTATTGTTGCAATACAATTTATAGATAGTTCAC
ACTAAAAAAAAGTTATGTTTTAACAGTAACTTGCATTTTTCCTTAATATACGTGAAAGA
AAAAGAATAAC
>Cluster-7456.0 MsigGR17
GACGATTAGGTATGTTAAAGTAGCTGAAAACAACGAAGATAGAACTTGTTGATTAATAA
CAGCGAATCCACCAGCTGTGTATTGGGGTAATATGTGCTTATTTAATTCGGCAAATTTGA
ATAAATCGTCTTCTAAAGCAGATCCCTTTAATGTTTCTTGATTTGTGTAACATGTTGATA
GAATACTCAATGCGCTTTTTTCCACACTATCGCATGATTTTACAATCATTACCGTTGAAA
TAATATAAGTCGCTCCATAGGCTATATCAACAGCTATCTCAGACCAAAGCCAATCTTGAG
TCATTAAGTTAATCACAGTTAAGCTTTCAAGGACAGTACAAGCGAGCATGAAAAACAT
TTGCCAACCAAAAATCTCATTAAGGTTACTAACCAATTTAAACAACAATTTAGAGGTAT
AGAAAATCTGATCCAGAGTTACTGTCTTATTGCCAAGTTCAACGTCTACAGTTTTAGGT
GCCACATTTTTTTCTCTAGTAATGTATGCCTCGATCATCTGCTTTAGGAAGTCATATCTTA
ATTGAATAACTCCATTTATCTTAGATAACAAATTGGTTACAAATAATTGATGGTACATTCC
AATCAAACTCGGAAAATACAAAATCAGAAACAGCATCGAGTTTTGTCTACTGGCTCGA
TAATAAAAATCGTAAAAATGTATTACAAAATACAAAACATGTAGAAGAATCAGCCTAAC
GTACGACCACATAACGGCTCGTCTTCTCACTTTAAAATCCACTGCACTAAGAGATCGTT
CCATTAAAACGGTTGTTTCATATATTACAGAAAACCTTTTGTAGTTGGTCAGCGGTGCTA
ATCTGCAAGTTATTATAAACATAATCGTCGTTACAACTACAAGGCAATCGACAACAACA
TGTACTAGCTTCATTTTGGAGTAGAGTCTTTGTCTGTCGTAAATGGAGAAGATAAACAT
AATTAGAATTAAAACAGTTATCACTATTAGATATGTAACAGATAGACATTTTTTCAGTAA
ACTGGTAGTTGAGGAGTAAAGAGGTACGATACCAATGTATCTACCGATTTTAAAAAATA
TTTTCACTATGTTCATGTCGGCGTTAATTTCTACCGGTTTATCCATTATTTTTT
>Cluster-12689.46436 MsigGR18
TAAGGGAGTATGGTTTAATCATGGATATTGTGGTAATAAAATTCTTTTACCAAATTGGTT
CTGTGTTAAGCCTTTTTCCCAGAAAAAGGAAATTTGGACAAATGATAATCCAGTTAATA
GTAGTTACTTGTACAATTATTGCGGGAGTTTTTGTTTTGATGCGGTACATAACAAAACTC
AATAACATTTGGGTAAAATTACTGGGTCTGTTAATCGCAGGAACGTATTGTGTTTTCTGC
ATAATGAATATGTATCAATTATTTTATAGCAAATATAAGTTGGATCTATTTTGCAAATGGAT
ATCGTATTGTGATAAATTTATGGGAGCTGTCTACGTCAGTAAAATTGCAATATTCACTAG
ATTTATAATTTATATAATATTACTAGCATCGATAATTTTGCCAGTAACATTTATTTACGTAA
AACATGGGAAATTATTTAGGTACTTTTTTTATAGCTCCGTATATTGTCAGTCCATTTTGAT
AGCAATTTTTATATTTGAATCTTCAATAGCCGTATCAAAGAGGCAGGAAATGTATTACGA

ACAGCTTAAAAGATTATGGAGATGTCGAAATACCACTAGGACCAAATATATCAAAAACC
TAAAATTGTTGAAAATGATTTATAAACGAATCTACTTTATTGTTGCGCAGCTAAACGCAG
CCTTTAATGCATACGTGTTAATAATTGGAATTGTGACGTTTTTGGAAATTCTATACAGAC
TAAAGATGTATTTTTTCTCGACTAGGACTTTTACTTTCATGGATAATATTTCTAATGGATC
CTTAACTTGTTTTGTGTTCGGTAAAGCGATCATATTGACAATAGCTGGAGATAGAATTGA
TACGGCTGGTCGTAAGATAACCAACTTTTTTTATTCCTTACACATGGATCTTAACGATCC
TTTATTAAATGATTTATTAAGTCAAGATATAGTAAGAGACTTTTTAATTTTCTTGGAAAA
GCTTAGACCAACTTTAACAGTAGGAGAGTGTATACCGCTTAACAGAAAATGTATACTAA
TTTTACTTTCTAGTTCAATCCCGTATGTAATACTTATAATGCAGATATTTTCAGTAGTTTA
AACAGTTCTATGTAAGCTGTACGTTTGGATATTAATTCGTCTTTCACAGTTTTTTGAATA
CAAACTGATAATAAAAAAATTAGGCAGCAAG
>Cluster-12689.2807 MsigGR19
CTAGTTTAGATCTGCATTTAAATCTACATACAACTTGAATTATTTGATACTAGTTACTAAG
AAGAGACAACATGACTCCAGTAGGCGAAAAACGATATTTGCACAGTGACATAATATTTT
TAAAATGGTGTTATAAGTGCGGTATGGTTTTTGGAGTATTTCCATCAATTTCTAGAACTA
AAATATTCTATATACAAATTCTTCTAATGTTGATATTGTGCATAATTTCTTTTTTTGCTTTT
TATCATTATGGAAGGATGATGTCGACAAATCGAGTGATGGGTCTTTCACTAGCTTTACG
ACTATTGCATACGTTGTCACTAGTTGGCATGTTTTGTTCTAATTTTAGGAGTTTAATGTTG
AATAGAATAACTTGGAAAATAATTTTTGCTTGCTTAGCTCACATTGATAAAGCATTAGAT
CATTCTTATATTACCAAGAAGAGAATTTTCGGAAAAATATTATTTTGGATATGCCTATGGA
TAATTGATCTTTGTTGTGTTGCTCCTTTTTTCCGACATACATCATTAACCGTTTATTTGTT
ATTTGTGTTTTATACGGCCATTAATTTGGAACTATATGTAATATGTGCATTTTTATTAGAAA
CTTCAATACTTATATCCAGAAGAGAAGAACTATTTTACATAAAGGTTATGATTTTCTGTA
ACAAGCAATACGTTGATAACATAACAAAAAATATTAAGATATTAGATGATACGTGGAAA
AATATTTATTTAATAATGGGAGAAATAAACCGCGTTCTTGGTTTATCCATTTTAATGATTG
TACTAAGTTTTTTTACAAATATGTTGACTACCATCGATTATATGTTACAGATTACACAAAC
TCAGCTGCGTTTTCAGTTAACTAGTTTTGAAATGATTTTTCATGCCTGTTTTGATTTAATC
AAACTTTCTACTATTGCCGTTGTTATTGCCACTGCTGGTGATAAGTTAGAAAAAACTGG
TTTAAAAATAAGTAAATTATGCCATCTTCTTGAAATAGATGTCGAAGATAAAGCAATCA
AAGGAGAAATGAAGGATCTCTGCAATTTAGTAGATCAACTACAACCAGTGCTGACGGT
TTCAGGATTTTTTACCATTAACAGAAACATGATACCTATGTTAGTAACTAGTCTTACGTC
GTATGCTATAATACTTATACAATTAAAATCCTAAAAATAATAATAATAACAAAATAGTAAT
AAATATATTAATGTTGTAACTAGGAGTAAGCAGTTCATCGATTTAAATTCTA
>Cluster-12689.9127 MsigGR20
CAGCATGTCAATTGAACGGTATATTATAACGCATTGCATAATATATGGTGTATAATTCAGA
TATTTTCGAAAATGTCGGACAGAAGAGTAATTATCCTAAGCAACCCTTTATATCAAACG
ATACGACCGATTCTTTTGATCGCTAAATTATTCTTCTTGTTACCGTTTCCGATCCAAAAG

AAGGGAAACGACTGGTGGATCACATGGTCTCACTGGAGCTTCGTAGAGGTCGTATTAA
TTTTTGGTCTAATAGTGTCATTTGGTTTATATGGAAAATTCCAGATTTATCAATTGGAAA
GTGTCCAGCCGATAAGATTTCAACGATTATCAAGTACATTAATAACTATTAGTGAGGATA
CCACACTAATTTCAACTTTTCTAATATGCAACGTATACTCGTTGATGAAATTCAATTATCT
CAGAAAATATTTCATTTACATAAATCGGGTGGACACAATTTTAAATTTTAACCCATCCAC
TACCAAAGAACCACTAAAAATTTTAGCTACAGCTACTGTGTCTATGGTATATCTGTTGTC
AGTTCTTTCTGCTGATATATGGATTTGGCTAAGTTTAACTCAAAATGAAATAGAGCCGTT
GACATACATTAAATACCAAATCCTTTTTTATGTTTTGTTCGCTTTTTTAGTGATACCTGCA
TTACAATATTTTATGCTGGTGAGATGTATTTTAATCAGATTGGAGTGGATAAATTATGGTT
TGAGGATTCATTTTACTAACTCCCCCAACAGGATATTCTCAGATGTTGCTCTAGTAAAAA
TTGGCCCATTATCGCGTCATTTTGTAACAGATCAAGTATTGTCCAAAACAGCTAAAGGA
CGCAAACATAAAATAAACTATGCTGATGGATACGTATTACTAGTAGAATCGAACATAGCT
TTAAACAATTATTTTGGTTTTATACTATTGGTGCTGGTATTTGGAAATTTCCTTTTTCTCT
TGATAACACCTTACGGTCTATTCATGGGAATCTTGCATAAAGCTTATTATGCAATTTTACC
GGTTTCCATGTGGATAGTTGGTCACTTTTTACGTTTTTTGTTACTTGTCGAGCCATGTAA
CGCAATACACGTACAGGTCAAGAAAATGTCTCTCATCGTTTGCAAATTATTAAACCTAA
ATATTGATGACGAATTTCGTAACCAGCTAAATACGTTGTTTTCTCAACTGAATCATTTTC
CTATTCACTTTACCCCTTGTGGACTGTTTACTATTGACAGGGGTTTATTGATGACGAGTT
CAGGAGTGAATATTAACCAGAAGAATGATATGGGAATAGTCGCACGATTAAAACTTAAT
CTAATGCTATACTTTTCAAGGATAGCGATGCAAGAAAATATAATTTTAACAGACTCCTTG
TATCAAATACTTCGACCAATTCTGGTGATTTGTAAAATATTTTGCCTACTACCGTTTACG
GTTCAGGAAAATGGTAGCGAATGGTGGATTCGCAGATCCTGGCGGAGCATATTTCAAG
GCTTTTTTATTATTACAGCAA

>Cluster-12689.23413 MsigGR21

TGTTAAACCAGTACAAATACAAAATGAACTTCTTCAAATTCTTCAAAATGTCGGAAGA
ACCAATAGTATTAAAAAATCGTATTAACTCCAGTACTTTTCAAGAAGCTATGAAGTTTCC
CTTGAAATTGGCACAAACATTTAGCTATTTCCCCATCTACCTTGGAAAGCGGGAAGAGC
AGTTAGAATTTAAGTGGTTCTACTGGAGAGTTGGTTACTCTTGGGTAACTTTTTCCTTTT
TTGTGATCGAATTTTTCTTTGTGATTCTAGATGCTTTTCGGTACCAACTTAATTTATTGGA
TATAAAAGTGATAGTATTCCACTTGGGAGCAGTCGTTCAATATATATTATTTTTCAAATTA
ACTTACTTTTGGCCAAGTTTCGTCAAAGAATGGGCAAAAGTGGAATTCCATATGAAAC
ATTTTGAAATAATGGGAAACCTTAAATTAAAATTGGGTTATTTAGCAGGAGGAATCATG
TTTTTAGCTGCAGTCGAACATGGACTGGTTAACGCATATAAATTAAAACAGCAGTTTGA
CAATGAGCCTAGTTTTATGGAAGGTTTTAAAACATTTTTCACTCGTACATATGAGCACAT
TTTTCGTGTCATGGAATATTCATTCTGGCTGGGACTACTTATCCAGTTTTTGAACTTGCA
ACGTACGTTTTACTGGAATTACACTGATGTGTTTATAATGTTAATCGGATCTGCATTAAC
ATACAGACTACGGCAGTTATCAAAAAAAATTAAAAATGCAGCAAAAGTTAAGGTAAAT

GACTTAGTTGTTTGGAAAACTCTAAGAAAAGACTACACACGACTATCTGAGCTTATTTA
CATCGTCAACGAGAGGATATCAGGTGTCATAATAGTATGCTTTCTGATGGACTTGTATTT
CGTTCTTCTTCAACTATACAGTAGCCTTAGACCCATCGAGAGTGTAGTGGAAAAAATAT
ATTTTTATTTGTCGTTCGGACTACTGCTGTTGAGAATATTTTGTATTTGTATTTTCGGCGG
TGCAGTCTATGAGGAATGGAAAAATATAAGATTTTATTTGAATACCGTCGCAGGATCTG
CATATAACGCAGAGGTTGATAGATTAGTGAATCATGTAGCAACATGGGAATTATCTTTGT
CTGGAAAAAATTTCTTCAATATTTCAAGAGGATTAATTCTTCAGATGGCAGGTGCA
>Cluster-12689.49537 MsigGR22
GGGAGACCCCTGAACATATAAAAAAAAATCCGTTCCGTAAAACGTTGCATTACGAATC
TACTTTTTTGGCTCTGGCGCTTGGATTAATATTTGTTTTTAAATGTCGCACATAGAACTA
ACTGCATTCAGCATAGGCCTTGGTATTTCTACTTTTGTGCAAATAACTGTATCAATAGCA
CTAAATAAGGAATGACACTGGAAACTAGAATTACAGCGTAATTTCTGTTAAGAGTAATG
TACCCTATAACTGTCAGTTTTGGTCTAAGTTTATACAAAAACATCGAAAAATTTTGTTTG
TATGTTTCTAGATATACATCTTCCAACAAAGGGTCTTCTAAATCCAAATCTAACAAGTAA
TATAGCTTCGTTATTTTTGATCCGGCCTTGTCGATTTTATCTCCAGCGACGGCCAACATA
ATAATTATTACAAACATAGCAAGACAACGGTAGAGGGTGGCAAGAGTAGCTGTGCCAT
TAAAAATTGAATTCATGTTTTGCTGAAGTTGCAACAGACATTTAAAGAACGACATAAGC
GTAATCCACAAAATGGAAACACCAAAAGAACTGTTCAAGTGTCTAATCGCAAAATGAA
AATTCTTATAAATAAGACGCAGTCGCTTTATTTTCTTGACAAACTCAATTTTACTCTGAT
TCTGATTTTGCCATAAAATCTTAAGTTCATTGTAAAATATTTCCAATCTCTTTGTAAGTGA
TATCGAACATTCCAGGACAAAACCAACCACAATTATAGCTTGAACGGTTGTATATACAT
AAAACATAGATTTTGTTAAATGCAATGCCCTAATTGACTGCAAATAGAAGTTGAATTCT
AACGTTACAAAAAGAATATAGAAAATACTCTTAAAAAATATTTTAGTTCTTGAAACAAA
GCTAATACCTATGGTTGTGTCAAAGAATGCAAACAATTTAAAAAATCTGCTCCAGTTAT
TTCTGTTGTAAATTAAGAAATAAAAGTTTGTTGTAGTAAAAATCAAAAAGGATCCCATC
GTCATTATTCGTAACATTTTAACAAACATATCTTCCATATTACCCAAAGTTATAATTATTG
TTGATAATCCTAAAATTGGGAAAACTGCCGAAAGAATGGACTGAATTACGATAATTCCA
CTTCTGTTACAAGGAAAAAGTGTTACTATTACACCAACTTTGTGAAAAATATTTAATAA
CAACATGTCTTTTATAAGAACCTTTTGCATAGTCTCAAGAGCTGTGTTTTTAATTGATTA
CTGAATGAAAGGTGATTTTACTTCACATTAA
>Cluster-12689.48074 MsigGR23
TAAGAATTTAATTTTTAAAAAATTACCATTTCCAATTATATTAAAATATTAGAAAAAGAA
AAGTAGATAATCGATATGAGATGATCAAAAGACGTGTAAAAACAAACTCATTTCTACTA
CTTTGTACCTCTGTTTACAAACAATTACGAAGATTAATGTATTACTCTATTATTAATAAAG
ATATTGTAATGCAACTATTATATAAAACATAAAAAAAAATGAGTTTTATATAACTAACTAAT
CGATGGTCTTTTTAAAGCATTGAGGTTATGTAAATAAACGGTGATTGTGACCGATATTTT
CTACTAGTGTATACACCTAAATAAAATTGTCTGAATATCAATATCCGAAATGGATTTATCG

CCACAGCTTCATTGCGTATACAAAAGCATATTTTTCTTGACAAGACTCACCGGCATTTC
AATGTTTTCGTTAAAAGAAGGTGCGATCAAAACATCCAACTCCGGCATATTCCACTCAT
TCGTTTTAATGGTAATCTACTGCCTAATATCAGGCATATGTCTGAACGAAAATTTGAAAA
ACACTTCGGTGGCAGACATGAAAAAAACGGTGAACGTCATAATGTCCGCAGTAAATAT
CGTTTTCATCAGCGTCGTAAGCTTCACGGCGACTACATCTCGAGAAAAACATTTAAAAC
TAATTTTCCATTTAAGCGAAACCGAAACACTCCACAAACGGCTACAAGGGACAGTCGA
CTACTTTAATATTAAAAAATGTATCAATATATTCTATTGGAAGACGTTCTTCATGTTGAGT
ATCAAGTTGTTGATATTGGTTGTGGATTGCTCGATCAAAGGCGTAGGGGACGGGGGTTA
CGGATGCTGTTTCATATCTTATTCGTATCCGCAGTTGATATGTGGATTATTCTGCATTCAA
TTGTTTTCGTACATTTATTTCGTGAACATCATTTTTCGATTTTTGAACGACGAAATCGAT
CAACTCTCGAAGAACTCCTTGGATGTCGTATTTTCTATCAGAAGAAAACCTTCGAACA
GTACGATCGCGCTGAAAGCATGTTCCGAGTTATACAATAAACTCACCGGGATTATAAGG
CAGATAAACGATACGTTCGGACTGGCTTGCTTGGCCATGTTCTTCATGGTGTTCACCCA
ATTGGTTATAGGGATATACTATATGAATAATGTGACTGAAGATTTTTGGGATCGTCCTTTG
TCGCATTTGCTGCGAGCTGTCGATTATACCATTAACGCCGTTTTTATTTGTTGTTTGTGTA
ATACGACTATTGAAAAGGGTGACATGACAGGACTTCTTCTTTATAAAATAGACACATAT
GATGATGATTTAAATAAAGAAATAGATATATTCACTTTGCAAACTATAAATGAGAGAGCT
ATATTCCATGCAAGTGGATTTTTTGCTATAAATAACAGATTGTTGTTTACTGTAAGTTAAT
AGATTTATGACTTGAGGGGGTCAGTGGAAAAGTGGTACAGGTGACATTCATAAGATTC
TGAGTAATTGAAGGGTGAAGTTTATGGATATTAATAAGTTTGGAGTATAGGTAAACCCA
AGACTGGCTACTAATTAAAAGATGGTGCTTTTCTTTTAAATAGATATACACTAACTATAT
GGTGATGCACTTATCTTCCACTATGACAAAAGCAATTATTTTTACAACTAAACATAAA
>Cluster-12689.32150 MsigSNMP1a
CAATTACCCCTACCTATCATTTAGTAGTGTCCGCAGTCCATCGCGGTTTCTAATTTTATTC
AGCGATAGAAAAATTGTTGATGAACTTGGTGCAGTTAGAAAAACTTTTATTGTCGCTGG
TAGTTAAGTTCTCATCAAACAGAAAAATGAAAATGCGCCTGCCGATCAAGTTGGCCAT
CGGGTCGTTTTGCGCGCTTTTCTTTATAGTGCTGGTCGGATTTATTCTTTTTCCAAAAAT
GATCACCGGGAAAGTGAAAAAGATGGTGAACTTAGCTCCAGGTACGGAAATTAGAGG
AATGTTTACCAAAGTACCTTTTGGATTATCATTTAAAGTATATGTATTTAATGTAACTAAC
CCGATGGAAATTCAAAATGGAGCCATGCCAATAGTAAACGAAGTTGGTCCTTTCTGTTT
TCTGGAATGGAAAGAAAAAGTTTCAATAACAGATGAGGAAGACGGTGACATAATGACT
TATTTATCAAAGGATACCTTTATAAGAACTACAGGCCCTGGATGTGTAGATGGTCAAAA
AGTAGTAACGATTCCTCACCCTTTGATTCTTGGTCTTGTAAATGCAGTTAATAGAACAA
AACCTGGAGCCCTTTCGTTAATTAATAAAGCCATTAAGTCCATTTACCAAAATCCAACC
TCCATCTTTTTGACAGCTAAGGTCGACGACATTTTATTTGATGGTGTTGTTATGAAGTGT
GGAGTTACCGATTTTGCTGGAAAGGCGATTTGTACACAACTAAGAAATTCTGGAAACT
TAAGAATTATAGACGAAAACGATTTGGCATTCTCTCTTATGGGACCGAAAAACGGTACT

GAACAAAAACGACTTAAAGTTCTTCGTGGAACAAAAGATTATCATGACGTCGGAAGA
ATAGTTGAATACGATGGACAAACCGAAATGACAACATGGCCAACTCAGGAATGTAACG
TGATCTCTGGAACTGATGGTACAGTATTTCCCCCACTGCTGACGAAAGAAGACGGACT
TGTCTCCTTTGCTCCTGATTTGTGCAGGGCGTTGAAAGCGTTTTGGGTAAGGAAGACC
AAATACGACGGTATACCAGTCAGCGAATATACAGCTACATTAGGGGACAGTTCCAAAG
ATGGACCCGAAAAGTGTTACTGTTTCACCCCAGAGACTTGCACGAAACAGGGGTTGAT
GGACTTATACAAATGCGTCGGCGTTCCCATTTACGCTTCTATGCCTCATTTTTACGATTCT
GATGAATCCTACGTCAAGGGTGTCAAGGGTTTAAATCCGAATAAGAAGGAACACGAAA
TTACAATTTTATTCGATCAGTTGACCGGAGGACCAGTATCAGCCAAGAAGAGATTGCAA
TTCAGTATGCCGCTGGAAGCTAATCCAAAAGTTGACTTGTTTAAAAACTTCTCAAGCAC
TGTCATTCCAATGTTTTGGGTAGAAGAGGGTGTCGATCTCAACAGTACTTTTACCGGGC
CACTGAAGATGCTGTACACAATGAAGAAAGTGGTCAACATATCAAAATATGTAATTTTG
GTTGCATCCATAGCCGGTCTGATAGCGTCTGCGTACCTCTTCTTCAAAGATAACGAGAA
AATCACCATAACAAAGGTAACGGACGCCAAGAAGCCGCCAGAAAGTGGTATTTCAAC
GGTGAACGGTCATGTAAATAAAGCAATGTCTGATAATGAAATTGACAAATACTAGAACA
GTTTTTAGTTTAGTAGACATATTATTATGAAACATCAACATTTCGTTGCAAAATATTAAAA
TTGCCATATCATCGTTATGGTGCTTTGTTTTAGATAATAAAAAGTAATTTTTTCTCGGAGA
TTTTGGGATCGGGAATTTACAAAATTTATATTCTGACGTTGGTTATTTCGATTTGTTTAGT
CGGTTGTGCAAACTACTGAACGACACGTCAAATTTAGTTCTATTTTAAATGAAATATAC
ACAGATCTGATCAATTTCTCCTCATCAGATTAATCACTCCTGCGGGGTCAATCTGATTAG
AGGTGGGAACATACCATATAGGAAAAATGATCCTCAGTGTTCAATAATTAATCAGTCCT
GCCCGGTCGATCCAAATCGAGATGTGAAAATGTTATGATTGAATTGAGAAAACATAATT
TGTTTGGAGTAGTAAAAAGCTGTGAAATTAACAAGTTCGATATCACGGATTTGTAATTT
TTACATTAATAATAATTTGTTTATTATCTTTGCAGTAGAAAGTGGTTTAGTTCCTTTTGAA
TGATAGATTTAATAAATAATTGTATTTAAAAAGGACGTTTTATATTACCTTTGTTAAAATT
TGTTAATTAAAACTATATTAAATAATTATTTATTTGTAACGTATGTCGAAGGAGACTTCTT
ATAAAATTTATTTAAAATTATAAA
>Cluster-12689.39408 MsigSNMP1b
TAGTAGTATTCGAATTATTTTATATTGCAAAAGATATAGGTGTTTTAAAAACTGTAAATAT
CACGTTTCACGTCCACCAGTAGCATATTCGACAAAAAATTATACAGAATATCTCACGGC
AAGTACGACCACAGGAATAGGTTAGGAATACTCATTAAGTAACATTGCATTTACTTTAAT
TTCTTTGCCCCTATCTTACGGCATCAATTCTGTTTGTCGATGGGAACAATACCGTTCCAG
TAAATTATAAATCGTTTAGTTCTACCACATTGAAAAACTAATTATAACTTTTTATTTAATT
TAATTTATACTTCAACAATTCTAGAAAAAATCATTTAAAATTGATATCTAAGGTGGTTTAT
TGTCTGCAACTGAAATTATTATGTTCGCCTCCATCGCTCGGACTAGGCGTCCAACTTCTT
TTGATCTATCAAATGACAAATAGAATTTTGTGTCCATTTGCCTTGAGACATACTGTACAA
ATGTTTTCTCTAAATGCTTGTTTATTTCGGAATCTAATGATCCCTTGAAAGTGGAAACAA

TTTATATTAATGTAATAAATATATGTACACGTTCAAAAATTGTAATATTAAAAATATCACC
GTGTTGGTTTCGTTTAATTATTTTGCTTATTCGTATCTTTCAAATTCATGACCGCTCATAA
TATTATTAGTATACCCGTTCTTCCCTTCGTTCCGTAATTGAGCTATCAGTTCGTTTGTGCT
TCTGTTAAAAGCGTTTTCGTCTTTCGCTTGCCGTTGATGTACCGGGGTGATTTTGACGG
TTTTGCTGGTCTTGAAGTGCAGATAACCTCCATAGCCCATAATTCCGAAAGAGGCAACG
AGGAACACATATTTCATGATAAGCGCCATTTTTAACAGAACAAAGATGTTGCTGATTTT
AATGAATAGAGGGCCTTCCAGCGTTAAACCATCTTCCAGCCAAAATATCGGATGTAGAG
AGGTCGACACATTGTTCATGAAGGATATCTTTTGATTGGGCGCTACGTCTAAGTTCATTT
GAATCCTAATCCTTACACTGATCGGAGCGCTGGTCATCGGATCAATATTAACGTTCATCA
CATGATCTTCTCGAACAGGGTTTAGACCTTTAACTTGAGTCAAATAACTCTCATCTGCAT
CCAAAAAATGTGGTAGAGATGCTACTATCGGGGCGCCTACACATTTAGTTAACTCAAAT
AGTCCTTTTTTAAGACACGTTCTAGGGGGAGGCGGGCAGTAACATTTGTCTTCTTCGTT
GCCTGTTTGATCGCCTAGATTACCTTCATATCTTCGAACGTGTATACCTTTGAGCACATC
ATCTCTGACATAAAATAGAGCTATATTCCTGCATAAATGAGTGGTGTAGCATTTGATACC
GTCTTCCGCTTTTAATAGAGGAGGTATAATCCAGCCATCAGTTCCTTTAAAACGATTACA
TTCTTCAGTTCCCCACAAACCTATTTCTTTTTTACCGTTAACTTCTAATACCCTTCCCAA
ATCCTTGTAATTTTTAATACCTCTCATGACTTTTAAAGTATCTGCAATTGTTGCGTTTCTC
GGTCCTATTAAGGAGAACAATAAAATATTCTTTTCAGTTGGAGATTCCTTTATGCCCTTC
ATCAGAGACTTCATTTGCGTGCAAACAGCTTTAGAAGCAAAATCAGTTCCTGTACAATT
AATTGCCATGCCATCAAAGAGGATATTTTTTACTTTGTCTGTTAAATAAATCGACTTGGG
ATTATTAAAGAGATGAACGAGGGCTTGGTTCACAATAGATAATAATGCTGGCGATGTTT
GTGATACGGTATTTATCATTCCCACAAGAAGAGGATGTATAATCGTTACCACATCTTCGT
CTGACAATTTTCCTGATAGGGTTTTGTTGAATCTGTAGGTATCAAAAGCATCATATTGCA
AGGAATCTTCCGGGTCATTGTCGATAACATTAATCTTTTCCTTATATTCATCATAATAATA
AGGTCCAACTTCTTTTAAGATAGGTATGGCACCATTCATGACTTCGTTCGGATTCGTTAC
ATTGAAGAAATTGATCTTGAAGTTGAGAGGGAATGGTATTTTTAAATAAATACCTCTTAC
TTCATTTCGTTTCCTCAGAGCTGTTTGATCTTTGACCACGAATTCCACTAAAGTCTGAA
AACTCAAAAATCCGAACACTATTGTCCCGACCACCAAAACGGCACCGCCGATAATCAA
TTTAACGGATAATTCCATATTTTCTGTTAAAATAATTTTTTTTAATTTTAATTTTTAACAAG
GAAAACCGCACGTCATCGCCGATTTCATTCGACTTCAGAAAGACAAAAAC
>Cluster-12689.34083   MsigSNMP2
TTGGTGCACAGGCACTTATTTGTCATAATATTGTAGGCAAAATTTTGATATTACAAGTGT
GTGTATTTTAGAGATTATTAATCTTTTACGTGTTTCAATTACTACCGGAAATTACTGGAGT
CCACAATTTTTTGGAAAATTTTCGTTAAATCGTGTTCATGAAAAATACAGTGGATAATTC
GAGCACGAAGAAGATTTAATAGCAAAACTCTACACGAAATGTACATTAAATCAAAAAC
GAAAAATAACTAACAAGAGTAACGTATTAATAAGCTACCTATTATTCTATTGATTCATAA
ATTAAATTTAGTGCGTACAGTGAAGTGTTAAATAAGTTTATTGAAAACATCACAGATACT

AATATAATCAGTTTAACGTTGTTGGACATAGCAACTCATGATGTTCTAGTGAGGTGGAA
AAACACGAAAAACGGGGAAAAAATGAAGAAAGTTTTAAAATCTAATTGTTGTTCCGTT
AAAGTTCTGACGATTATTACATTGGCCTTATTGGTCGTGTTGATTGGAGTGTTGGTGTTA
AGTTTTTACGGTATTCCCCGTATAATAGATACACAAATCGATGCGTCAGTTCGGTTAAAA
AGTAACACAGAACAATGGGATCGGTTCCAAGAGTTACCAATACCAATAATTACAAAAA
TATTTATGTATAATGTGACCAATGCAGATGATGTACTAAACGGTGCAACTCCTAAACTAG
TTGAAACAGGACCATATGTTTTTAAGCAAAAATATAGTAAGAATATATTAAATACGAACG
AAAACGAAGATAGTGTAACATATGAGCAGCATATAGAGGCAACATTTGACCCAGATTTA
TCCGGAAATTTAACATTAAATGATACAGTAGTTATGGTCAATGCTCCACTATTGGTGTTG
ACTCAAATGAGTTCATCGATAGAGCAATTGGTAACGCTAAATTGTCTAGATAAAATATTC
CCAGCAGAGTACAGTACTCTCTTCATAACGGCCCAAATACACACTTTAATGTTTGAGGG
TTACCTTTTTGCACAAAACACTGCAGATCTCGGATATGCTTGTAGTATAGTCAGAAGTC
TAGTTATAGAAAAATCGAGAAGTATAAGAAATATTCAACGCATATATAACGAGGACAAG
CCTGATGTAGTTGAAGCACTTAAGTTTAGCTTTTTGGGATTCAAAATTCAAAAACCCGA
TGGAGTATACACAGTAAATAGAGGTATTGCCGACGTAACTAAATTGGGAACCATAATGC
AATGGAATTATTCCACTCATCTTCCATTCTGGGGAACAGCTCAATCGATAAACAATGATA
CTTGTCAATTAGTTCGAGGATCAGACTCCACCTTATATCCACCGAAAATACATCAGAAA
AAAAATTTTGAAATATTTTCGACAGACATTTGTCGGTCCGTAAACATTTTTTATAAAGGC
GAAGGGTCATATAAAGGTATTACAGGAAGGCGTTATGAGCCAGATACAAATACTTTCAG
ATCGAACACTGCAAACGATATCAATGATTGTTTTTGTACGGAAAAAACTCTAAATAAGT
ATGGTAAAACATCTTGTTTTTTGGACGGCGTTTTGGATGTATATGAATGCTTCGGAGTGC
CACTCTTATTGTCAATGCCTCATTTTCTTTATGCTGATGAATCATACATAAATGGAATTGA
AGGTGTATCACCCCCGGATCCTGAGATACATGCAATTTATTTATTGGTGGAACCGAATAC
TGGTACTCCATTGCAAGGCAAAAAGAGAGTACAAATTAATTTAGTACTTAGACCGATCC
AAAACATGCCATTTACAAGAGGTCTTAATGGAACAGTATTGCCAGTTCTTTGGCTTGAA
GAGGGTGCAGATTTAACCGACGATCTGATAGATATGCTCAACAGCAAATTTTTCAATTT
AGTCAAAATAGCAAATGGGGTGAAATATGGATTAATAGCGGTATCCGCGGCTGGTGTAC
TAGTTTCAGGCGTCATACTTCTTAGAAAAATGGTTTTGAAGTGGTAATTTATTATATTTTA
TAAATATACTCTTTACA
