# Supplementary material for: Identification of candidate chemosensory genes in the antennal transcriptome of Monolepta signata
Source: PLoS One. 2024 Jun 7;19(6):e0301177. doi: 10.1371/journal.pone.0301177 (PMC11161048; doi:10.1371/journal.pone.0301177)
Supplement: S2 File — (PDF) [file pone.0301177.s006.pdf]

File S2. The amino acid sequences of olfactory-related genes in *M. signata.*

>MsigOBP1

MKFYLVFLCFCCTSVLSQDSGTINQIIRANQNCSRRTGVSPAMAAGLMSGQFPDNAALRK
HVRCMLEEVGVQDSQTGQLRIEEVERMANALAPSNVPRANLAPVIRQCAVQRATPDDTA
YEMVRCFYNAGRAALNG

>MsigOBP2

MKSIVIFALFLVSANAIDSQVVDAFVAKVAKFSQKCVEETKATPEDLAKLMAREEPDSHEG
KCIVSCVYKAFKVQNEDGTMNFEETKKFMEKVKESDEEIYNKLMEVYNKCKGVAEQVD
DDPCITALNVAKCAIKEGKAAGLSKEVLGM

>MsigOBP3

MKFILLGLCLVLFVQYGLCAMSEKQMNATKKLVRNTCTNKAKVAPEVVDAMHQGDFSQ
GQCYLLCIMNTYKLLLPDGTFDWEGGIKAIEANAPPSIAGPAAASVKNCKDAMKDKSNK
CNGAAEIVKCIYDDNPSNFFLP

>MsigOBP4

MKVIFVFLCAIAAVLASSLPNLPSSEAKHLRDVHNSCQSNPRTFCDEDKLRNLVSNVDDRQ
VGVHMLCMAVKAGLMRDDGEFNLDTMKNKISLVTHDKSKVGGFVSQCSRKHENPGKTA
NKMWICFFENGIQYYHKL

>MsigOBP5

MKNKIMKSIVIFALFLVSVNAIDSKLVQEFVAQATKFGEKCIEETKASSDDIATLMAHDIPD
SHEGKCMISCVYKAFKIQNADGSMNSEETKKLMEKVKESDPDMYEKLVQVFNKCQGIAE
HIVDDHCITAVNVAKCATVEGRALGLKAEMFGM

>MsigOBP6

MRTICSIILVLTCVLGYMEALKCDFQQADGEKIKNALSHCVKNNETEKLWHLLQGDDSDE
SDSSEENDSQPTSINSTSTSSKTKRAVDADTNSRIVTPTDNSTEHTNTSDDTEETSVEDVTQ
DCIIHCVLDNMQMLDSNGLPDHSKLLEAILRTASARELHNFLQDSVDQCYQEVNEGKA

>MsigOBP7

MHLLYIASIFLFVTVVTTVTADIAADENEFIKAREKCQADPVSRLDDDTLKKLEKHERVDK
QKLRKNSECLNVQLGLVKQNGDVDPDKLRWVAKYFTNDKVKENTIVEKCASISSGSPEET
IVALHSCAAKIARGSN

>MsigOBP8

MKIFYIFALVLFVTLVTAAPTAEQEAEEVAIVKKAKEECQADPETKLDEDAVKDKSKEERM
NDPKVRKHALCMNVKTGLQQENGEVNKDKLKSLLERRMDDQDKINKIVEECSVKGTGT
PVDLAIDFDKCMMKHLHKHHHDHGHHRHHEHKHD

>MsigOBP9

MKTLLLLFFTTIICSVFCDLNGKGLGDRLKKKAHKMHSDCLLYTGVTDELVEGVLLGQFP
DNEPSIQRYNFCIWNVAEVFDENYEISESVARENLGDSMTDEDVLTHVKCNKKGRDLGGN
SPVKLAWIMKKCLYKNIPKERYATLRLN

>MsigOBP10

MFCGLLILSLISFVAVIGNEQNETNYTNRCEIPPSAPKRVEEVINQCQDEIKLAILSEALQSLN
LNENAHSRAKRAAFSDDERRIAGCLLQCVYRKMKAVNETGFPTIDGLVALYTQGINQKEY
IRATIESVHVCLRSAEKKFAVVPKTLAEEHGKTCDIAYDVFDCVSEEIGKYCGQSP

>MsigOBP11

MKSIVIFALFLVSANAIDRQIVDAFVAKLTKFALRCVEETKATPKDLAKLIAHEEPESHEGK
CIISCVYKAFKVQNKDGTMNLVEAKKLIETVKKSDEKIYNKLIEVYDKCEGVAEQVDDDP
CITALNLERCIIKEGHAVGLNEEILRM

>MsigOBP12

MKCLAVLTFIVCMAVYTTGLTEKQVKAAVKVVTNVCLPKSKATIEDVEKMHKGDWDIDR
TAKCYMYCAFNMYKLINKDNTFNYESAMSQIAQLPDFYRTEAKECIDKCRYAGDKATDK
CEAAYEMSKCVYFCMPDVSARTPDLR

>MsigOBP13

MKSIAIFALFLVSVYAGDDAIKEFAAKASKIGEKCVEETKATSEDVAILMSHNIPDSHEGQC
MISCVYKAFKMQNADGTMNAEEIKNLIEKVKDVDMDMYNKLNQVFDKCHAVGEQAEA
DHCVGAVKVAKCAMTEAKAVGLNGQMIGMQSTRLFLVGLYTCH

>MsigOBP14

MKNMKFFCIAALALFVAVATAAPTEAERDAIKKAHDACQADPATRIDEATIEKLKNHEKVD
RTVLNKHALCMNVKNGLQQENGDINKEKLRSIVERHVDDKDKVDKIVEECGNRGSATAE
DAAVALDKCLEDAAGRPRHHGPPGGG

>MsigOBP15

MLRFIILGILAGGALAFTEEQIQLMNDLHAECVSQTGVPEDLIDKAKSGDFVQDPKIKCYM
KCVFDEIGVIDDDGKIDIEGALAILPDELKDIATPVIKSCGTQAGADVCEAIFNTLKCYYDT
DKRAFFLPQTAMSTNN

>MsigOBP16

MTKIILSAILVTLFYMTVSLGQEETIKKCMKETGVTKEDAETKPPKNTPAIRCFLKCLLTTK
GQLLASGKLDLDKLTEVFKKIDASDSDKEEVKKCLSTIKIETCDDIQQISECWGDLAQTPG
AII

>MsigOBP17

MNYSSFFITFVGVVCCILNDYSAEAQNNTVREHCIKEVGISKEKVAKLEDDTLKDIDEQCN
CYLRCVFLDIGAIDAKGKVNVEKTLKEFKDVFDADCLKKVPKIIECNDMAALDDCDKTR

>MsigOBP18

MKFLTSLLVIIGACYVSANITQEQHEKVMGYHKDCAKKSGVNLELVKQAKEEGKFVDDD
KFKIQLFCVSQKLGLQNEDGEIQLDVLKAKLSAILEDAALAEQLISKCAIPRGSGPETAYQT
MKCYKESNPKRVLVV

>MsigOBP19

MKVLYIFALVLFVALATAAPTSEEESEEEDIFKKAHDACQVDPKTKLDEDAVKGKSKEEIM

NNPQVRKHSLCMNVKTGLQQENGEVNKDKLKALLEKRIDDQDKVNKIVEECSVKGTGT
PEDLAMEFDECMMKHRHKHHRGHEHHKHGEKKDD

>MsigOBP20

MFLSIFVCAVFGIAFCKIDFSDSYEEEMLKVINECQEDPETRAADDWIETEDLETADQEGPH
ILCRLTKIGSLTQDGDIDKEAMKTDLEKGIDDATVVETIMEKCGTRPPGLNATESAVFNWR
CVLSTFKELTDIKE

>MsigOBP21

MKTLVLGVILTILICLANCYLERKDFSDVLNSKVDDLHANCKCYSGASEKLINQVRLGQFP
ENNSCIKRYNYCIWKDSGVLDENNTIIEKVLMSHLSDCNYDDAHIYIECNNKVDCGGTCPI
NPMWEMQKCIAEKVKPENYIYF

>MsigCSP1

MNNLTLLILVVSIGVVLCEKYTTKYDNIDLDQILTSERLLSNYFNCVMEKGKCTPDGKELK
AHIGDALENDCEKCSEKQKDGAVKVIKFLAKNKKQMFDDLAAKYDANKTYRNKHREEF
QKEGISI

>MsigCSP2

MKLSIVFCIVLVIVAVSAKPGEEKYTTKYDNLNIDDIINSDRLLRNYIDCLMGTKKCTGEGE
ELKKVLSDAFASNCEKCSDVQKAGAKKVLLHLLKNKKDWFNEIEGKYDAGHHFLTTNA
DEIKAAGISV

>MsigCSP3

MVPLLYVVIVSLCGLVVPAPAPEEKEKYYTTKYDHVDIEMILNNRRLVNYYMACMLNKG
PCPPDGAEFKRILPDALRTNCKKCTEKQKVTTLRAVKRLMKEYPKVWAQLQAEWDPDDV
YVKRFINTYNNGEYNPPEPQKPIIVESQKPITDEPPNPIDVFNRFGDEDDDSQPPVKKPQQT
LTDNKATESVTKSTVVKYNGESSSSTLGIFLPPIPTLPPVPNEFNPIANTIGQG

>MsigCSP4

MQKLESYVVYVVVLLTLVVVAASASRDRSSISDDALEAALKDKRYLLRQLKCATGEGPC
DSVGRRLKSLAPLVLQGNCHQCTPQEKRQVRKVLSYMQVHYPKEWNKLVKHYSG

>MsigCSP5

MQRVGLLVFLIAVGYAMGDLTPAKYKHIKVDEVLSNTRLLKRYVDCCLGEVTVCNNESGF
LRDTLPKAINTNCAECSQNEKEGALKVIAFLQKNHSDWWVKLDKLYHASESQFCIENAA
KI

>MsigCSP6

MKTFIVALFLAIVALAAAEKYTTKYDNVDLDTIIKSDRLLLNYVNCLLEKGKCTPDGLELK
KVLPDALLTDCSKCSETQKKGSKKIIRHLIDNKPEWYKELEAKYDKDGTYKKKYDAEIKT
KK

>MsigOrco

MMKFKVSGLVADLMPNIRLIQASGHFMFNYHADNSGSLHTLRVGYSCMHLVFCLLQYGC
TFVNLLVERGDVNYLAANTITVLFFTHCITKFCYFALRSKLFYRTLGIWNQPNSHPLFVES

NNRYHALALKKMRTLIICVFSTTVFSAVAWTSITFVGESVHNIKDPENENMTLVEEIPRLLV
KSWYPWNAMSGGAYYITLIFQVYYVAFSLMHANLLDSLFCSWLIFACEQLQHLKEIMKPL
MELSASLDTYVPKSADLFRAPSATSQDNLIENDYNNAKNEELNLKGVYNTRQEMGANFR
SGALQSFGPGGGGVGPNGLTKKQELMVRSAIKYWVERHKHVVRLVTAIGDAYGVALLLH
MLTSTVMLTLLAYQATKINGVNVYAATVIGYLVYALAQVFHFCIFGNRLIEESSSVMEAAY
SCHWYDGSEEAKTFVQIVCQQCQKAMSISGAKFFTISLDLFASVLGAVVTYFMVLVQLK

>MsigOR1

MEILTLNIKSLQILRIIRKPGEMWVKGIFINFCQIFATAFIVYLVILAGVHQMTVNFEDSMILA
ETIMPVLSGGVYLFIFFIFKITAKSIEKTIEKFSTFLRFCDKEVIVEAEKEVQFFSKVFFIYFCV
GSSIYGAVPILDLKSCYESRLSDESRKHDMCGIPFRIWAPADVTKQANFYFYILLVTFVWV
VCVSGVLCITLLLVGFLIHINVQLKNLQKHILRMFDDDEQIGCDKREENIHFCIKYHIAIIEY
SKEVFKHFEMSLIIHVSLPSFIMGVVCYQMVFEESLLEKLRYFLHLVGWVAVLFMVCYYG
QIILDQSKYVGDVIYDSKWYNGPLHLKKSVLLMLLRSQRPLKVTAASFSVLSLETFLKIIKT
AYSFFTLLLTMST

>MsigOR2

MLNDFNQPMFQPKCEEHLKVAKVTVTIQKTLFYVCLVLSILVDCAFMAVPMIVNERVMAV
QGWFPFDWRLSPNYEMVYIFHCTVALWVTMTCMNLDIFTCGLLMHIGLQCDFICITLSSL
DIFCVKNGVLQLNDGIKLTPNTKQFSNTMVENLVVCIKHHRHLKRLLAHVEDIYKVTLFIN
FLLGGVILCFGLFRLSTVEGGAVESMVMILFMICMIIEQFIFCWFGNRLTDKSANIFFSAYNT
PWTTCNSNFKRILLLFMAVTQAPMELKVGGILVVSNPVFVSVVKSSYSYFTLFKNLQQ

>MsigOR3

MTDSYEVDLTDIVSRNIKILYFFGVIPQEKESNMALLIYVVRVSILTGLLYGGLILAQVTQWI
LSFTGDIEEIVTATYLTTTNLFSFIKVCFVFRHRKRLLNLISTLNIKEFRPRNVHQSKVLLENI
TMAKMVTNGLLGISYLTCTFWAIYPFTIQYGPCMPLVAYIPYSIDKLSVFAITYVGEVIGIVV
SANCCLGIDSLITGLIIVISAQLTILNDSLVNLRKYSEMEIHKATDNNKGTRSNLLDKVMVR
NIIRCVEQHRRILQFSKDFQDIFTVATFGQFTVSVLILCTTLFKLSLITNLDLDFFSTVLYQMC
MLMEIFVLCYFGNEVIVKHHLLTESAYQSDWLDTSPTFKKNLIFFMTVSQRNFRLLAGGY
VTLSMDTFAKILKSSMSYYTVLNQINDEQ

>MsigOR4

MEKAADESRFVAKWRETNGGYGMNFKRIVGANVAALELCAFMVPDFNGIMDRMWYGV
RFLLYVVGTYGSQITSETINLYYSTGAISEIVSASFLFLTHAVQIIKVVYLYTYMDRVKNLIK
SINRPEFQPQSEYQRDTLNFYIKVSKMITYSFWGACVATCVFWASYPFTEDELGLPLAGWF
PFNTTKSPNFEYAFTYQFIAATLNGLSNISIDTIMSGLIMVICAQLHILNDSLINIRHFAESELE
HEFRDDNGRDEISPRLQDTMNRKLVECIVHHRCILEYTKEFQTLFSNSILGQFIVSVIIICITM
FEMTLTPVGSLQFFSMVLYQYCMLLEIFLWCYFGNEVIIKSNELTKSAYLCSWIFCSEEFRK
NLRFFMTRTQFETNIYAGRFFTLSLGTFVTIVKSSWSYFAVLMNINK

>MsigOR5

MEDMGILSLNIKSLKYLFLWPRPNDKWLNRFGSFTLFVLIVMMSASQTIAAVVHQFFVNF
EDNKVVFEGIIALADFFGYGFMYANFKLNCHKIKSTIDKITVFLKFCPEEILYEAEEKVRLIS
KAFLLYICGGVAVNSALPMLAFQSCEETRLNEYYKIHDPCGMPVRTWYPFNAKQPLHYYF
LVTLHAYTCLAIASTVLSITMTLVGLLIHIITQIKHLRYLMLNVFDHDVEDRVKCERKLHFC
INYHVTIINYATEVFQDFNLLLIVHVSLTSIVMGSLCFQIVYAENATDKLRFLLHLGGWVTM
LFITCYYGQQIINESLSVADVVFDSKWYNAHLEAKRCIILMMMRSQKPLKLTAASISVLSL
QTFLSITKTAYSYFTLLLSMEQ
>MsigOR6
MDKILPPTKFMKLMMYVCALTGIWPLTCENKPRLVRLYHYFFILTYSNVCIATVSMLSQLY
IILDRENFLSDEAVGIMSFVLVWTAILIKVVQIKQPQTKDLIRKILHFENSVNASADEKLIAIF
NQYSSKNNLLCSVYITGVFFNFIVVTLVPILQPKIMETTTRTFPLNCWFPFDQQKFYAEVYV
ILTFYVVLISIYCLGADMFSFSMILFPIAHLKIIQHILSDFDNYVEKTAKQLNCSPSEARFITIR
ECIILHQDVIKYMNEYNKLFGGASLFDAIQSTIQLSSLILAVVLGEITLPQLIKAVFLICNTLF
RLFIFYWYADDIPIESVKITKVLMESNWHEQPTRIQKMMLFIMMRSNRQLAMNLGSFTTIS
LKVFFAIIRGSYSYFTLIYQTS
>MsigOR7
MYPKFENLKITVKYIVRVSFFLGIFNGGLLLTGLAQFFLVIGNLEEMMKVTFLTFTNIIAFGK
FYVICKHQPDLLCLAEKMNRKEFQPKSEKQIQVLKNYIRFSKMISFCLYVVCAMTVGFWS
VYPYTQENGPFLPTAGYIPFDTNNPIVFGLIYAYEVAALVVSGYVDMSADFFIASLIMVIVA
QLKILNDSLANISEIAEAEIKQERYTSLECFQNKQKIINRILIECVEHHKAVIEFAEEVTRLFA
VIIFVQFIVSVFVMCATFFEIILVPVMSMRFFSMAWYQLCLLLEIFPICYFGNEVLIESDKLTN
SAYHSDWINYSVEIRKNLLFFMTRSQRNLKLTVGGFFTLSLDTFIMILKSSWSYVAVLIQIQ
NKQ
>MsigOR8
MENDAYAKDFFIVNRWILRFAGLWRPESQNEIIQSLYTLYVVGIFLFVNLFFTFTEFLSILYV
YENEYDLIKNISFALTHFMGAVKVVFFYFQGHNLKRIMTTLESSELHYEDCEMKKFYPAVT
SKLYKKTGIKYTIIFFMMAHATLTSSYLPPFLAALKSEVNNTERMLPDRLPYYSWMPFRFD
TAGTYLIALGYQAIPMFSYAYSIVGMDTLFMNIMNCVGMNLEIIQGAFVSIYPRAVEKTDG
PLLTPDELHNTETLTVILRAEMKKISKHLQIVYKVCDDLEDIHKYLTLAQATATLFILCSCLY
LVSTTPIGSKQFLAEIVYMIAMGFQLTLYCWFGNEVTLKADKMPFYIWHCDWLTADNDFK
KSMILSMARANRPLYLTAGKFAPLTLPTYVAILKASYSFFAVIKNTSD
>MsigOR9
MILESSAFGVMNTKDYCKLFLWLPKLLLRAIYFWPRQKTNYYQRHILFAISALLFAFLIIGL
IFRCIYVSDDIESFCTQIIFIISMSQALAKLVALFVNKNELKTILDDILYNFWPYDLVSNDSEN
EIKTFYSIITHILVIMLILSLTFSVLIMSSPFIYGELPFSVDYPKINIKTTPYFQLLYLHQFITDSC
LFYSIVIGTDYLFTAIСSCVITQYKVLQHSLLVFNTPVMCTINEKLRSETGEAFGRKYETVH
KEFFVRCVKHHLMLLRITTKMNKMFSSIEMIEFSYTITDVCLLLFMLTHAENLTSFKVVFY

LVTLAVLLNQLLVYCAIGNELLHHATILPIFIFQTNWHTIGDEELVKDIMFMLQRSQNFPQL
SAYGLYNVNMDTYLKVLKFTFSLYTFLSNMKGK
>MsigOR10
METVIDYRSFFSFHVFVFKIFGFWKPDNTMKHKVLYNFYTAFCTIVWILFLLSQFIYMYKN
LDDVEELTAVSYLAGPFIVDLIKMLVIYRKMDTIKVLMVNLNQPMFQPKCEEHLKVANAL
KKFHRNFFYLCLYLGVQTYLFFLIMPFLRDEKLPPTQGWFPFNWRPSPNFEIIYIFQNSVVL
WNTIICLNLDTFSSGLLMQIGLQCDYLCITLNNLDKFCVKDGVLVLNGEVPLNNSTFPDTM
MENLVVCIRHHKYIKRLAANIEDIHKTSIFILFLGGGIIICSGLWQLSTAKIGSIESFMLVSYTI
CMLTEQFMYCWFGNEVIQKSGHIFNCAYNTPWRNCDLKFKKILLQFMTLTKIPIRIKVGG
MLVISNAVFVSVVKSSYSIFTLLQKIQE
>MsigOR11
MESQINREDKVNILLKLMTYGVDKSKMWIIRFLGQAIFVTLELFLATSIILIIYHRGIRAVIPH
VTLLCLYQPACSCFYIASLYYRDSVIEGLEEMEIISRQLRMQDQIFEEKISIIAKQTIFKTMFIL
VTIIVAHISFLPIWFDDIDYNSGYWLINYYMEQHMRYVSIFIYCLFTVLGGLAIVFPFSYLVY
FTFHVNLHFAWILKYIENSFNEIKQHPRCSNDDIYQEMVYENICNIVKYHIKIKSGFQIVLRV
QSFTLLITSVACIIMAVGAIYFIVLGIDPPHNFRMYFAIFTLIIGYGDVCFNGQAITDSSSRLL
NVLYNCKWTDWNSKNRKALIIIMSNCLQDVEITAYNLYALNFKTISGGIRLIYSLATAAVNL
R
>MsigOR12
MEVSKGAHLKVVINFAIASGIWPIVYTKNRFYKICYYAYSWFLFMIAIVFTMQHLINFFVCI
TIDMDLKRLTNITTSFTVYTIGLLRLKYIWTKNSIKMIMEIIAREKEIQTSEDNMIKNIYKEN
VRKTHRLNYWYLYVLCIDSVVYLTICQFLTPKETYVNPVTNVTYFQRPLPLYPWLPFDEH
DFHTVAFLWAEVGCTSCLIICYGTDAIVHSYMAYILGQFHIFKYILNNFDAYKNNIKGQLK
CDENKADFVTMQLCVIDHQRLLRFLDTFNDTMRIVMLYDFLQSSMQLAMLSIFLMHETK
LMVFVVSVGYILTMIGRLFICYWYASEISAENYALVEDLFAVKWYEKPQNVKKMLAILITR
CTKQIGLQIGGLATMNWSIFIGIVKGAYSFITFMLR
>MsigOR13
MFKDIGLRVLGTTDYCKLFLWIPQSLLQIFIFWPTQNYYKKTLSLIGQLIVSCIYGLGLVLH
CRLVLDNTEAICYQLINLISLFQTVTKCIVLYINTSELNGILDDIFVKFWPYDLLNTGLKTEL
RRWYNIITTIMVILLTLAFVYDASVLIPSLLSGRLPVPVVYPYIAYNVQPFYLVVYIVQLISF
LLFYFVGIGCDLLFMAICSSVISQYKLLHHSLLVFNTPEMDQVNETLRQVENDELGEKYN
NVRKEFFVRCVRHHQMLLGITDKMNVMFSLIEMVEITCVIMYISVILFFLTSLKDPTISQRA
FLLTFLVFFLNQLFLHCAIGNEMFYQASLLPEFIFQVNWHNLEDNQFKKDFMFMIQRSQDI
PQLTAYNMYNINIDTYIRVVKFSFSLYTFFSEV
>MsigOR14
MYYPINKQQPFYSTLLSLSLLGIYPLSAKHRNFTISRITTAGNVIFALLICNIGAIGHLIVSLK
GNKGAEISEDMAVAFGGLGFLMCAVLFKMRWTRWSKFWIELTNFKEFGIPENLSELTTRC

NIMSMIYSVYISGGMCAYAFMSILEMDCDDSQENNNLCGTLTQIWFPVRHVSNSIIKVIFFF
QLLVCIWACVAAGNLFFVSFESCEFIVCHTHHLKKKLLELFEVDNEITRKINLEVCIRYHNFI
IKMGNQLNYLTKTTLGHMSLTAAVVMGMISNQIVQKYKPLGAGIYLGGYVIAIFWLSHAG
QRITDESFAVADAVFEAPWYKASSEMRKDLALIIQRSQEPLILNALPLGTFNYALFITMLKA
AYSYLTLLQQSVSTENDGQVIK
>MsigOR15
METKDYCKLFFWIPKLLFEVINYWPKKKNNFYKQLIFFIAAEILFIIFSMGIILQSIFVYQNIY
SFLDLMYVVALTTLIIKFAVFFFNRKTLKNMLDEILTKFWPYDLLNATVKEEIKKFYTNILW
FMLSLVLSILLTTSFSFWIPLIHGGLPVAVYFPLIKTNVTPFYEIIYFGQIATIYPFGACMALGT
DIFILAICSSMISQYSLLQNTLLTFNTPEMLTINDKLRMIDPENLQEKYKSIHKEFFVRCVKH
HQLLLRITQYVNDIYNFVELVQIAFTVTGVCFGLFISTAEENSNIMDILYIGSIIFFCLNELFLY
CAVGNELYHQASLLPDYVFQTNWHEIENQVPTKDFMFMLHRAEKIPQLSCYNLYHINMD
FYIQVVQYGFSIYMFLSHVKEKNEK
>MsigOR16
MGFITYTRILFIISGIWLPDISHPVLFRCIQYYATFARTHFLLVCIIFFTGLIFGDEQVLEDNLQ
YFPPFVVMVIKMFYLRRKELLDLIALSRNIEKKLLNDGNPEISQIVREENKYSNNLFIALMC
LFFLTLCQLLLVVLYLMTSFRTVVNTAIWYPFDYRKHVYFTTFHQLYYFSYVIILYAAYDTF
LGSLIIFATTRIKVLSYKFKNLSKLSKRTNIPVERVIDTLIQEHNELIRYVDNINKCLKWCFFV
DFTMKSYNFSQFLFSLINSLENSYVEMMYSIFKLISVIVENWYINYHGNDLILASKDLASSI
FSSSWYEFDIKSQKCCQMIMLRAQRPLEIQIGNMYHLSNDLFIAILKGGYTFILFYNV
>MsigOR17
MKNDNNNNTWSYPEEFFRTNEIITKITGMYIPTKDDTVWMKVFYTMYLICFYTTGIIFIVC
EVLIFNETITNFNKLISHIGMLFTHLCGILKCIILIFGRKKLQRMMNTLQDVNYYYSPLDNFS
PGLMLSKEKKASSLISVVVFIMYSFVGISAHISSEMILNEEIKSEAFKGTNKTCQDFMPYFF
YVPFDTEMKSQCKRAFLIMDVGLVIFAWVISCYDGIFVSLLNCLKSQLQIVCHVFRSLRSRS
LKKLQLKMNCNDNYNENSALENEMYKELTHSTEHLKILLRVRDDVEATFTFVTLTQTVAS
LLIFASCLYIASNVPINSPEFFAQMEYFMCVLVQLSLICWFGNEITTASELIPLSLYEGDWLSS
SPRFKTSMMLTMTRMQRPVYLSIGKFSPLTLATLVAVCRGSFSYLALFKNVQ
>MsigOR18
MFSVSKIPFNVSIKFLQMCLILPNTANGNLRAFFVKTIILRILTFGVLVGVISHIIKIKLDHIDE
IDTSEDILALSTGFGTMFILGMATYLHKRWKKLLIELVDNSKFGKPETMDEIIYRNNLFAQC
FASYLVGGGFVYAYVTYREQPHCHRVMEERNINLQCDTFLSMWYPIKIPGYIQYLIFVIQFT
LTQYDICPVAMLVFLTFEATEILQGHFVQLGKHFTNIVNDRNLQTRRECFSYWVKYHNHIL
RMTDELNNLSKKTVSYVTLIAAFVIASVEIMIIRDTKRSAAILFLIGWNVATFMICHSGETV
NQLMSTLHDTVYTSDWHTLDNETKKDIVFILFRCQKPLHLDALPLGVINYTLFVLLLKTS
YSYFTLLNGSA
>MsigOR19

MAKSVENTEKHLYEKEFRNVLRLWRFCGMHPLKGYSKPLIVFNAALTFYITVLIALKLLL
GHELVTVESAGVFTQIWVKFVMLTTKKDKILQLFTDIEGFWKTDPPGSENANMLKSLRK
MERAFLIYISISTCMFLFKPLLVKGTTIYYYYQIPQIPFLISYVIEFYVTLVTMSMVIGVNLFIS
ITIKIGAGQFSNLNAKIKQLDLSKTQNDERGYQACRREIKENIEYHEHLISYVRHLDGIFSW
LFTLLISIITSLLCMNMYVLSQPNNTIVDIIRCGTMVLAFTSEFLLLYGVPAQQLIDEAEEVA
NSVFYHCKWYLPGIVELRKALSFMIFRSQKMVCLSALGFIDVNRQTIVAMIKTAYSFFTFL
QTVET
>MsigOR20
MNKFHLDFKEIISVSTKFLYFFGIMYPKFDDFKVIIKYIARVSLFLGFFFGGIVLAEIANLYLS
IGNLSELMNALFLTLTNLVSIGKFYVICKHQPDVLSLAENINRKEFQPKSEKQIQSLKSYIRL
SKIISFSLYAGCTMTCGFWCIYPYTEENGPFLPIAAYIPIDTSNQIVFGLVYAYEIIATVIGGYT
DLSADLLIASLIMVVAAQLNILNDSFANISEMAEAELKQSQYYNFEYYIQKTINRKLMECV
KHYKAIIEFAEEVTRLFTTIIFSQFVVSVFVLCATFFEMTSVPIGSVRFFSMALYQYCMLLEI
FPVCYFGNEVLIESNRLTNSAYHSDWINYSVPVRKNLIFFMTRSQRNLKLTAAGFFTLSLDT
FIKILKSSWSYVAVLLQMQNKNKVQ
>MsigOR21
MNQGSNFRQALGYPIDLFDINKFLYKIAGIWIPKRDYNIFFRIAYLLYVISFYTIGICFFICTF
MLLHETINDLNKFFNHLGMFLTHAVGIFKFCILVFGRHKIKKIMDTLQHAEYLYDSTDDFS
PGSYLSQEKTKSSKISLWLIMMYSCVGISAHISSRLVMNEELKENDIKAINKTCKDFMQYY
FYIPFSTDEKWQCELAIGFMDISLEVFAWIIACNDAVFVALLNFLKCQFLILCHAFRSIRERS
LKALNLPKNYEIHYDTDNPALEDEMYKQLCHCIKHLQILFNVRDDIENLFSFLTLTQTLAS
LFIYASCVFTVSILPVGSMESFVQMGYFFCILVELFLICWFGNEITAAGEQIRQSLYQSNWQ
GSSRRFKKSLILIMIRMQRPVNLTIGKFSVLELATFVAVCRGSFSYFALFKSIQ
>MsigOR22
MVTLYICQLWPYNANFRLRSSINCLIGILIITFVCGGLIQTLDIAGGSIGKHLFNVLFIAYGLQ
AVFKFVVLCYKVTQVKELLDEVLNRFWPYNLIRGELEKELKHFYLFVMLFMLSICIIPYSA
MLLFFLRPLLSQDRETPFLVKYSFDWQASPWYELVYFSQVCGFFVYMIPFSFVIDFLLISFG
ACATVQYRILQKCFASLNTPEMEIINNKLKQLENNINPEYDETKGYFVKCCKHHKLLTRFT
KKINDIFQVNIIVHLLFSIINLGMILTACVMEDSINFDRIFVGIGIFSLLFLYCFIGTELNYHAS
LLPYCIFKSNWRNLDHTFQKDVMFVLLHSQRTPQLSAYGVYDLNMDSFTKVLNLTFSTYT
LLSTLSEK
>MsigOR23
MDKLDGFGFKVMGTRDYCKLFLWIPKLILYAICLWPEAEATYCPRYIFLAAAEILFLLLSAG
IAMQCIYVSREISDIFMELICVVGLVSAMIKFVVLFVNNRKLQIIIEDIMNKFWPSDLVDDG
EVKKEMKTFHSTITWIMVYVIVSFLFGAILEFFVVPLIYGGLPVPVYVPLINYKVGPFFAIV
YFGEIITVGFFCDFVLMGFDLCFMAICSCLISQFKLFCNVITTFNTPEMVEINTRLREVDEK
NKKSLKNKYKTVHEEFFVRCVQHHQLLLRTIYHINNMCGFVELVQITFTITGICSGLFVLTT

LDNLHFLKVFLTSSFIVFSLNELFLYCAVGNELHYQASLLPHFIFKANWHQMEDTKFTKNF
MFMLLRSQDIPKLTSYNLYDLNLNFYVQVLRFAFSIFMFLTTIRTKTQQ
>MsigOR24
MFLDGVAYKILGTRDYAKLFLWVPKAIFQIVQLWPTANHLTTKSLFICFYHVTMNLFLSIG
VFRYVKDNIFDENSMILLVVLMGSFEAIVKNILLFCKGPEIKQFCDDILIKFWPHDLVGEKL
KAKLRFFYLAGSVCQLAFLLTNICAGLVFVLPSFLGERRLIFAVDYPFDWTSFPVFEVIHLL
QIITNLTSIIPTVIGFDSLFMGIGGCMVTQIRILQHCFLIFNTSAMKNTNIRLKHFYEEENLRY
KEKREYFVKCLKHHQLLMRFAEKIGNTFSFVILIQLLCSTILIACSIMSVILKNQITIFHICLFI
HGFIQLLMYCIIGNELQYQAELLPEYLFKSNWRELNISFQKDVMFAIQHSQKFPQLSAYGL
YDLNMTSFLKVVKTGFSMFTVLSNLAQK
>MsigOR25
MKTIFLKSFVVELILVKICGIWDYVYNNKRWEHLYRLYFLMLNVLLAMYNVMKFNDTLQ
ETTLVGAVAAGFVLPIALMGNVRSFCFFAYRKEFFELLSTLDDEIFQPRDAEEVKMAQGML
KYYHNFKIAMYVGSFLPSFGCPIGRILFGERGQSYCEAVITSKRGEAIYMFQAISLGMISVIN
VVTNYFMVGFSLFIGLQCDRLCYRLQNMEITKDGTQSIKQYAEHHRRILRYASCTEKLFSII
YFTFMIMCLLAFCMTLLIISIIPDRYSFQCLHLVVYQMSIFVMLLIPCWFSTQVNTKSENIPV
AAYFCQWPNQSKGFKNELIFFIKKSQTPIQFKAMGIVDLSMETFISVVKTSFSYYTVLNDIL
FEREV
>MsigOR26
MFLRIPKLLLQPLYFWSHQNHNSHQRNFMTVISVICLAIIFIGTCYDGSLVSSNHLDLFLQL
NVSAGILQGTAKLCTFWANYNTFKIIIDDINTKFWPYDLLNIVKVKSEINLFYKLITSIMVCL
PVFTCISVLLMVFKFGIHPFPVAYPYFNAKSFPYLEIICLMECVVIFAVYCVMMGSDLSFMG
ICASLTVQFKLLMNMLVIHKMQDMDEVNAKLHTIDNENLKKKYKDVRKQFFVRCVKHH
QLLLRTIKNVNKMYSFIQVSQLIFTVTGICGGLFVLNYVENLSLLSTLFVWSAMVCCLNEL
LLYCIIGNELHCQATILPEYIFQINWDVTGDKVFTKDVMFMLHRSQDIPQISAYTLYDINME
FFIQVVKFSFSMYVFLSTMQEISNAN
>MsigOR27
MVFLKINKFMEKYVPSDFFTWLDFFFTITGLQPPAKPFFKYLYYLVTVPNLLACSWLFSCC
EWLNIFNSSDDDFKMIMFFVSLTIFDTILVFRISVYWLTRNRLKDLKTIIRRPSFNFHCFAVT
AIDYIEASTIGRNIKCHLLGSYEQFQDVWKKTTPQFSKTSNTLETYRIKTMFYTRLICCTIFL
SILLTSIVTSGIWFYNDFNAEIYEKFNPYLNKTSSYRKFSLRIYFPFDTSLDSGKNIWFAYFF
QCYGRFALLHAFLPVDTVFLACIIHLIGQAQIVGEAVKYIDTNICKDQTSKNIFLIKEIRLVK
CVNELQEIYRGFEILEELCTIQMLVQYGCATFFLCSLSYILPMVTNMAEAFCCISFLIASLGE
IFIFSYICQTLTLEIQEIAVTVYKTEWINYPIKLKKLVLFVLKRLQKPFCITAGKMFILNLTFFS
KIVQTSYSFYTLINASRE
>MsigOR28
MFFNIRICLMMLSTVGVNPLIREKNYQKVLYFFSVFISTWSIYICSLQFVYEDTSDILLLLPR

IGNILLLIHAIVKITVLFVKHEDMGRLLNDTKLFWNIDEFPFEKERKSSQRILRNLKILLWAY
PGFGVITTTYILFGPLILQVRTLPMSTYVPKNPPYAVLYLVQDFSFMVIYMGIVFFDVLVGTL
ILITVAQWKLLNKELAVVLESKIYNEEDRILFQRGIKKCIDHHHFLKEYVDRINNVTYFSNL
AFMGYFVISNCLGIFVIARCPPGSGQFLRILLLIGAQNEFLSFYIIPGQSLTSEAENTESAAFA
CNWHDNSVELKKPIITMIALMSRKPVYITAANFIDLNFESGLKMYKMTVSYYMFLRTMED
MAHEI
>MsigOR29
MLMLLVVKDIDNFLEASHFIIISITFLFKLPLFVFGDKIMRKIENHLKSIDVSGIPMQIFDHVI
KEDRTRNILYFPQRFMVLGGLAFQVVVCLILHKFRPVILISWSPFDLQDPKFYYPTLCFQTI
CFIMSGLPNTSMDITYYVLLDVACCEFDILMYKLMHLDTSRTSLQIEKDVRKYVIMHQEIIS
YINNVQALYANIIFVQCIGSVIVICFLGFQLAVTTEFPSGRFVTQGFFLASMIFQIFCYTWFG
QKFLLKSSEVTDACFLSNWHECDVKIQKMLLNLMTRSVKPIVLHAGIFDLTLATFTGILRS
SYSYMAVLNTMYG
>MsigOR30
MEVPKIEHMKVVIYFTLAGGIWPLIYAKNKFYKYGYYAYSLLVYILAILFTLAQMATFVIQ
VKTDKDIAIMTNVVTSATVFIIGLVRQAYLWRKSTTALILSLEEREKQIVTSDDTVVKAMY
RENVRKNHKLNYWYLYLQCVDSAIFLYLCQFIVPKEHYVDPVSNKTYVQMPLTLYPWYP
WNTHEHRLLALLWEKVGSTSCLIINYGTDVAMYSFIAYILGQFDITKHILRNFESYKNKIKE
QLNCDEDKANFVTMQLCVSDHQKLMRFIDMFNDSLRDVMLFDFLQSSLQLAMLAVFLM
NEKSMIFVSVAVVYIVCMVGRLAVYYYFASEIMAESFALAEDLYEIKWYDKQESVKKMV
NILIFRCNQQTGLQIGGLAIMNWSIFIGIIKSAYSFVTFVLR
>MsigOR31
MNEFHLDFKEIIGISCKALYFFGVMYPKFDDYKLFLKYALRVSVVFGFLYFGLMLTALAQF
LRVIGNLREMMKGTYLTFTNILAMGKYYVIFKHQPNILNLIKKINRKEFQSKSETQTQSLK
SYILLSKIYSAILYTGCTVTACFGTIYPFTEENGPFLPLPTYIPFDTSNPIVFGLVYAYEIVAGT
VAGYMVLSADCFIASLIMVVVAQLNILNDSFVNISRMAEAELKQKRSRSVGYFQEIINRKV
FECVEHHKAIVEFSEEISSIFTLILFIQFIISVFVICATFFEMMLVPIISMRFFSMALYQICMILEI
FPICYFGNEVLIESDKLTNSAYHSDWIDYSVQIRRNLIFFMARSQRNLKLNAGGFFTLSLDT
FIKILKTSWSYVVVLIHMQNKQ
>MsigOR32
MIIAGQWKFAEFTFLKRMYNKFSDLLLFAFAVCTHYFLFTIFFYPRCSNSVFEIIFYYIHFIIAI
ILTVLLKAGSIKNFLSWIIEYENIKIFKAKEESEIYFAYCRMNNKFTIFFIIVINIVSWMWYIV
GKRDSLEIVYNPDCPIARGFVFQIWYPFKIENYPWICSSFDLVTVFCGMILFSYYKTVPLSLI
VFLLSQISLLKYYIRNIENKEYETTVSDNLTLRDCVQMHQYVIRLMDWIQNSLKHIILIQYC
SYVLDTAAFMIPILTEDSMQIKARCFAGFCMTVTQMYIFFWFANEIQHESETISDVIYNETN
WIKMNERDKRTLLILMFRSQKKMSLKSVAIGEMSLNSFTKIMRICYTTCTFFTTMYDK
>MsigOR33

MFSVSNIPYNVSVKSLQMCLIFPNTTEKNLRKFYVKTSILRILTLGGVLVGVISHFIKIQMD
HIDEIDTSEDLLALAAGFGTMFIMGMTTFLHKRWEQLVKDVVDDSKFDKPEAMDKIIYRN
NLFATCFASYFIAGGFVYAYVTYREQPNCHRVIEEKNINLQCDTFLPMWYPIKIPQYIQYIIF
LLQFILVQFDLFSVAMLVFVTFESTEILKGHFEQLGKDFHKIVNYKNLKTRRAGISYWVTY
HNHVIRMTGELNYLSKKTVAYVTLIAALVIACAENMLISNTKPLAAILFLIGWNVGTFILCH
SGENINQLMAALHDAVYTSDWHKIDNETKKDIVFILFRCQRPLTLDALPLGVINYALFVLLI
KTSYSYLTLLNQSA

>MsigOR34

MDKLDDIGFKVLGTRDYSKLFLWIPKAILKPICMWPGPTYCPRYIFIAVAEIFITLLGAGVA
MQCIYILTDVTDIFFELVYGVGMISGVLKYLALFVHRRKLEVIVEDVMTNFWPSDLVNDD
EVKKELKTFHSTIIWILVYLTFLVVLNIVVDVVVEPIIFGGLPLPIYVPVINYLAFPYFEIIYIF
ELFTMTYMCNFVMMGFDFCFVALCSCVVSQFKLCCNAIITFNTPEMFEVNAKLREMDRK
NGKHLKYKYNTVHDEFFARCVQHHQLLLRSVDLINEMCSLVELVQTTFTITGICSGLFILTT
MEDRTFTKVFFVLGVILFSLNQLFLYCAIGNELYYQASLLPEFIFKAKWYEMENTKYAKSF
TILLHRSQDIPEITSYNLYSFNLDFYVRVIKFAFSMYTFLTTMQEKSQQ

>MsigOR35

MNIENIKEIKIMQFSIRLLRIPYLFPSTSEMNDPKANIYYKYLIVNLSSIYFSIGAALHMTFNI
KNGIYAKLDKDIGNIISYHGASYFVFRFLLNLKKLIILFKQFSDFTTFGQPNNFTKRNNYIN
KLSKIYIIYHTVIILTMLNATLLYIPQCHQENLENNWHEVCGLPAPTWMPFRFNYFPVQQLV
FLYECYSVFVIYQTAGFISFVMLATLEHLGLRFEHVGDTFVDALNEKNRVIRRKKFYKAVQ
YHQAVIGMGQLLNECFSPCMMVHISLTGPFIGVAGYSFLTRIPLDDSALMVGWMISTFIVC
LGGQRLMEASISVGTIMQRINWYDLEPDLQRDLTMVIIRSAKPIFLRAGPFGPITYSTVVTIL
KTSYSYVTLLKQTM

>MsigOR36

MLGSDIIKLLRTFLHGIGLVKNQNMLDKIILTSIFTFSVSLLLLILTNLFTSELNLGFFINFCEP
MSTYVLVVSEYTTMIIFNDDIVKIFEMRKEFWNYQQTDLVNKATRIYKFMWNYFRVMLM
FFVLTFITQSSKPFLYGVLPYICWIPNGQIWFILISFMEIWTCSHATIAIFCLDGIMGLIFIELSF
QFKILNKSFSEMTTFEDIKKCVDYHNFLLRYIQRIKKCFKIHYFVTYFTSMFIICIQTMVVLD
RQQAFNLKVKAVCYMLSMCNQLTLYCIPVSLVRDESRNSAEIIASSNMVIYGNIKSKKCLI
MIMQMCLKDISIRAGGIFEMDRNCYINVFKSVYSVYTFMNKK

>MsigOR37

MSHHQMKFIKPFMVLAGIWPLKITGIKLFLYNAYFVISFTYFCLNIATIYVTANQLAAHGRP
TEITNYISNAVFATVMIYKVLVFRSSGMKELLEMIDKYEKSIFNAFDEEQEKIYKMYGNCD
FLGRTYLFSSIFLCFCVQLLPLIGTLTSKPNEDGYREKYYIVTTWEPFDKYKYYFTTYFVQL
AFTVFAEMYVTFCGTLFLYILKNVIGQLFVLQHRFGSILKNARKMCDGHEITFKEACSYLM
KDAIRQHQEIIKLVEHIDKTFKGYIFFEYLCTSFGLSLIVLQFLLEETVQMKIAAAGYFCVFA
GQSFLIYYQVEAVTMQSSTGLATAIFSSDWYDLDADAQELLPFIILRTQKPLTLSIGSFGKV

GIKSIIALFKATYSFLSVMW

>MsigOR38

MLKLIFMWPQKNLRNLKLFYIFSLAVFVFVQWGLVRYFYINFQDYNKSLTVISSMSTIFQA
TLKLTILLYHSRSLGKILQDISVEFWPDDLVQEYTEKFRTNYRIMLGAMIMLTYFSLMFSTS
SLTLPLWLKNHDLPFRSVFPFDYTASPTHEIIYIIHVYVNGYLINLAVLGFDFLFMAFCSNLV
TQFAIMKRVFWEFGTEQGKYFNQKLQILDNRPNKRKFKIYDENQDFFVRLIKHHQLLSRT
TKSVENVFNLAAALQLCSSMVAICVSGFISTQENINKLQAFTMSCYLTGHLFQLYLYCASG
NELLAESDNLTDHIFNSNWYEIDNIKLKKDMVMLMRNAQIATQITALKLIPLDYSTYIKVL
RLSFSFYTLLSTLFVK

>MsigOR39

MGSVTLDFVAYYKSNLNALKYLGIWSQDVKVKFKSIYYAYCSAVSVVFVLTFSMGQVAN
MIDHRDDLNTVANSCYTFSTCYMAIVKSYYMYKNRHAFHKLQNLINKPILQPITEADVSIA
VEALRIYTVLKITIEVLGNVLMLLFMISPLIYGSYMETFPLEVWYPFSVTEPSVRAVVYVH
QCISIYYIGCIYMYIDLIIFGLLIYIGLQCDLLCNNMINIKFISGYELKKIIKRHDELVRFAKTI
QDMFNEIFLFQLSATVLGMCMSMFLLATRENTSVDTISLLLYQMTIWILLLLPCWFSTNVR
DKSELIPLAIYSMPWWDCNSNNIKKDLIRFIHATQKPIIFTTAGIFTISAETFLKILRSSFSFYT
LLISMNTKGEPTI

>MsigOR40

MDKLDDIGFKVLGTRDYCKLFLWIPKVVLYSICMWPGGTYCPRYIFFALAEILILLVGAGV
TMQCIFIFRDVTDIFKEIIYGVGFISGMLKFLVLFNNRKLQVIVEDIMTKFWPSDLVNNEEV
KKELKTFYSTITWILVYLSLTFVFNVIQDGFIAPLKFGIHPITVYYVPLINYQVSPYFEIIYLVEI
VTVYFFCDFAIIGFDFCFLAMCSCVVFQYKLFCNVLLTFNTPEMVEVNAKLREIDRRNRKN
FNNKYKTVHDEFFARCVQHHQLLLRTVDHINIMCSVVELLQIVFTITGVCSGLFILTTLDNL
DFAKAYFVESIIVFVLNQLFLYCAIGNELSYEASLLPEFIFKANWHQMEDMKFAKNFMVM
LRRSQKIPQITSYGLYHLNLDFYVRVLKFTFSIYTFLTTMQQKAHH

>MsigOR41

MYQIQRHQPFYETLKTLHVSLVYPTKNDKNHLKRFFLMSFLLRCPIWIDILLVVVHYFQVK
KAKISMDFSENFFVLIGGGALVIACNILPFTATEWSKLLTNVTQFKFFGEPNELKKCIKLWN
RLSVKLRYYCICGGTFYALQGYLESKKCLMIKEEYDVHIVCLAYFPIWLPFELNTGTNWIIF
ICQMITVFFVTLPGALTCFLIWEVTNMLLLHFRRLKYVFSTIIVEPDEKKRKQQIRLWLFYQ
LHIFRMVRRLNILSKKCMGIYSLVAASIFGCISYQLVSGHKLIGAAIIFFGYFVSLWMLCDA
GQKIRDESDSVTEAIYNSPWYTIDTKTKKDILFMMMSNSINIHLDALPLGHMNNALLVMI
MKGAYSYLTLLNKSSNKTM

>MsigOR42

MWPYTAHFRLRSITFCCFGISTIAFVTFGLAQSVNIAAGGIGKNMCNIVLITGFFQTSHKSIL
LCYKAPVIKELFDDVLFKFWPYNMTGRKLEQQLKMFYLVIILLMICELSMPVMFTLLIFLR
PFLMSGRETPLPVQYSFDWKVTPLYELVYSSQIIAFGVCVCTFTLGSDFLLMGAGASVTVQ

YRILQKCFSSLNTPEMQLIISKFKQSEYAEHSHYDERKKYFVKCCKHHQLLIRFTKKINDVF
SPAMIAQLLSSMVALGAVLTGVILEGSITFDQFCFGCGFLGQLFIYCFIGCEMNYQASLLPD
YSFKSNWKEIDDIKFQKDVIFMIQHSQRIPQLSAYGVYDLNMESFLKVLKL
>MsigOR43
MYIGYKVLGTRDYAKLFLWIPKILLQICQIWPSSKHHRYKSLVYCILAISLSVFLSVGLVGF
MDFTLGIENSMYNIVFLISTFQTACKALILYNKRSALQELLNDIFEKFWPYNLTEGKLKHNI
KSFYNALLFPLLLFVIIGIVFGATLFLRPLLVKTVKFPLPVKYAFDDDSFGLYVLVYLLQVIE
FFIVMIPNVVGTDSVFLGIVACAVTQYRILQKCFSVFNTPEMQAINIKLKHLNKVESVKYN
NTQVYLLQCWKHHLLLIRFTKQINDIFNSVILLQLFCSIITLSTLGTYIILLGSFTPDQLFFSC
AFLEQLFFYCIIGNELNYQASQLPNCIYASNWRTLDHQLQKDVMFIIQHSQRINQLSAYGLY
DINMDSYAKVLKMAFSMFTVLSKISAK
>MsigOR44
MLNGIPTRILGTTDYCKLFLWIPKFLLQTILSWPRQKDTFSSKIFFIWYLLVGCISFIGMTCY
GVVVAENTEARCKQILFTVAVAQMVLKLVVLHLNNARLKEILNDIFLKFWPYDLLNTDVQ
IEIKKLYTFITTIMILLFIVGTSYDGSVVWPIMINGQISGPMFFPYIDLNTEPYYQIAYVAQTS
SIILYYFVVFGCDFLFLTVCSCMITQYKLLQHSLLVFNTPDMVEVNKKLRLIENDRLSDKY
NDVQKEFFVRCVHHHQMLLRITKQMNVVFSFIEIVELTSTVMYISAVLFYTTSLIDPTISDA
VLMIILMVYYLNQLYLYCAIGNELYYQAGLLPKFIFGANWYNLKENAMKKDFMFMLKRS
QRTPRLNAYNLYNIDIECYIRVLLFVWWERMTIANYSYGYQNCCYKPFFFGLCKKILFIRK
PFH
>MsigOR45
MFKDNNIPFNVSLKILRMFLIFPPKIPNLKKFYIKSGIIRILAIFGIIVGCVAHFVKYTLDHENE
VDKSEDILLISSCFGTICAMGITTYMYKDWEQFLNNLVDFSKFGKPENMDNTIKRNNKVA
RNFTIYCVSSLLTYGYITYRERPKCHKIMEEKNISLMCDTFIPVWYPIKVPQYVQYIMFGFQ
MVLADIVYFPAFMLVVLFYELVQILNVHFEQLKKDFQKIVHEKNLKIRRERFAYWAKYHI
NIIRITEDFTSIVKKSFSIFVWITAFVFACAENMLVRETKQLAALTFLVGWNVASVLVCHCG
QEIHEFMLSLHDAIYASEWYLTDNQTRKDIVFILLRCQKPLNLSAWPHIVIDYALWVLVLK
TSYSYLTILAA
>MsigIR8a
MWWLLVTTFFVIGATQDNPKLILIKEKSGQESFLEWYNQLILPVKLESITLNVEDSQDNGK
VCEAIAQGGNIVLDISWCENEEAKSFFSDIGIPYIKIDMSIAPFLDLLDRYLDLRNATDVTFI
FEDPSYIDQTLYYWLNSATMRMLMTETLNAGTVRKLRDIRPMPNNFALLASTDKMNKLL
KLAFQENLVKLPDRWNLLFLDFQTRKFDKSLIANQPLNLISMNPEVCCYFLNSNNDCDCP
SDFDLRKSFFSKAFGLIKSCIDDMTIEGIEFPGFVCNESEATHQTSKVIEECLTNAIENENML
SFNSSKIRIKTNGFIQVGDNDNTVAKVENGVVSIAQDATIKPIKAFYTIGITYAMPWAYKILD
EKSGQWIWTGYCADFGRKIAEMLNFEFEFIEPKEGTFGKKINGIWDGVVGDLVSGKTDLA
ITALIMTADKEEVIDFVAPYFDQTGITIVMRKPVRKTSLFKFMTVLKLEVWFSIVAALIVTG

LMIWFLDKYSPYSGRNNKKAYPYACREFTLKESFWFALTSFTPQGGGEAPKALSGRTLVA
AYWLFVVLMLATFTANLAAFLTVERMQAPVQSLEQLARQSRINYTVVKASQTHKYFINM
KYAEDILYKMWKELTLNASTDDGRYRVWDYPIKEQYGHILL
>MsigIR25a
MLESKTYPHLVLDTTMTGLGSETAKSFTAALALPTISASFGQEGDLRQWRNIDDTEKSYLV
QISPPADLIPEIVRTIVLNQNITNAAILFDNSFVMDHKYKSLLQNVATRHIITPIKDGNEVIEQI
SQLRKLDLLNYFILGSLTNIKRVLDAADTLNFFNRKFAWHAITQDEGEVKCVCRNATILFV
KPLPNATFQDRLGTMQRTYQLNTEPIIASAFYFDLALHSFIAIKELIADGVWKNAVSNYITC
DEYNGNNAPKRVGLDLKKYFNKDNSEPFTYGPISVVSNGLSYMDFQLVLSSVGVREGAS
DKSLTLGTWNAGFDNNLTLVDPRAMINLTADVVYRIVVVAQQPFIFKDEKAPKKYNGYCI
DLINKIAEILKFDYELVEVDKFGNMDENGKWDGMVKELIEKRADVALGSMSVMAERENV
IDFTVPYYDLVGITILMKLPETPTSLFKFLTVLENDVWLCILAAYFFTSFLMWVFDRWSPYS
YQNNREKYKDDEEKREFNLKECLWFCMTSLTPQGGGEAPKNLSGRLVAATWWLFGFIIIA
SYTANLAAFLTVSRLDTPIESLDDLSKQYKIQYAPLNGSSVQTYFERMANIEARFYEIWKD
MSLNDSLSEVERSKLAVWDYPVSDKYTKMWQAMKEAGLPNSLDEAVQRVRTSKSSSEGF
AYLGDATDIRYLENTNCDLTTVGEEFSRKPYAIGVQQGSPLKDQFNTAILQLLNRRELERL
KEKWWNKN
>MsigIR40a
MYSANLTSLLARPGREKAIHNLYQLENAMVSRGYRLYIEKHSSSHALLENGTGIYSKLYDL
MILRQGYNNVLVDSVEAGVKLVREERKVAVMAGRETLFFDIQRFGPSNFHLSEKLNTAYS
AIALQLGCPYIEEINKILMAIFEAGIITKMTENEYEKLGKEKALSSSEIAENVAKETNKDLKR
QIKVEEENDKLKPISLKMLQGAFYLLSFGNVFSGFILIGELMFYKHKLRNRAKRRNRRFIS
KRWEKVVKLKMNRVRLMMRRFYRNAMHEAFVSTLEYIE
>MsigGluR
MFCVASGMIFSRKSLLFIRILIFCANYGVSSAEEYKIGAIFDEPHTKQEAALQEAVIQINEND
DDIDLELIVEHIPRDNPYYAAKATCSLLEQGVVGILGPLSEDNSNTVQSICDLKEIPHIEVRW
DDYPTNGTLINLHPYPDSLTKTYYDLIVAWGWKDFVILYENNESLQRVGELLKLFDPSSHR
IVVRQLNAALSGGNFRPILKEVWRSGATHFVLDCSIEILEDVLRQAQQVGLMTNKHNFIIT
NLDLHTIELMPYQYSETNITGMRFVDPDAEDLAEIANKIYKQNLQIFTGYNLKLEEALIFD
AVKMFAEGLKASEPLVQSMSISCYNDKEKLRSGLTVVNMMKSLEYVGLTGPIKFDVRGFR
STFALDVFELMEGGQTVVGHWNASHSPPLNISRSFPPVPSDEEDIRNKTFRVMITLTEPYG
MRVESSLPLYGNDQYEGFSVDLIKELAAMRGFNYTFLVRMDAKNGNYDNKTGKWNGLI
GDLIDEVSDLAICDLTITKERAEAVDFTGPFMMLGVSILYKKPTKAPPSFFSFADPFAFEVW
KLLIVSWLGVSLILFALGRISPTEWENPYPCIEEPEFLVNQLDLRNCAWFITGSIMQQGSEIE
LKSISTRMVAGMWWFFTLLMVSSYTANLAAFLTTEKPDPHFNNLHELVERAPIKEIKIGAK
SGGATEVFLRDKWLADKTSDFGKAYTLYMKDKDNPKILDNKDGVAEAQKGYYAFFMED
QSISYETQRKCDLNKVGDKLDDKGYGIAMRKNSTYRSTLSTAILSLQNSGKIDEIKKKWW

EERKGGGQCSSDSGSTDATPLNLKGVEGVFWVTIGGTILAVFLAMLETVLYCLKKASRTR
TTFWANLKEEIKFYFRFSKMEKHVGNEGENGEEPSEERDINYELVKKQSSETLQSNNSSRS
RSRRSKSKNKNRLRPPNLSVAAGTMGWMVTENEP
>MsigIR64a
MKVNRFVRIVFILQISVFLVDGFLDVKLIQGYFKQKTINYATVVGCFTNRERMQLAKSLSF
GNQHLAFVNVDSFMGKMKFFENNFRHIGIIVDGDCEYEELKLFLIKCGMEQHFYRKVHW
LLLSNNPNFSNIFKDVELYIDADITLVRPQVIENRTDLVIEDVYNPATWKGGELKTRIFDVY
NETLGFVAMDNVHKYMSRRNMTGVTLKASVVCKAPFKGNLLDYLQTKDQIDLNGVSRN
QYDILNSCVEYYNFTPNITLTDSWGYVAADGTVDGVVEELRTRAIDYSSTVLLVKEERLPF
AEYGRQTWVTRSGYIFRNPRRLNSLEAFVMPFTYTVWVCIIFMCLFCVVFLKYTHVSEKR
HLSQTTETSWTIMIMNTMAIFCQQDLSHIPIFSSGRMTLFVMLMCASITYQFYSAVIVSSLLI
EPKTMIKTVEDLLNSPLKAGSEESPLNRDFYVKTTDKMVQELYHKKILGKGNSSNFYTTK
KGVELVQEGGFAFHFAMGNVYSLIATTYSDQAICELREITMSLQQGNLLVPKRSPFRDMFD
TCLQRVAEGGILPKQMKYWHPTKPECIKTTVDLISLGIDYFYPLYTVFFIGILISLLILLYEIRT
YKKEEDEMNVFY
>MsigIR21a
MLYLYLLLITKLTVVSSIQTVDKRALQKSHEKSQQEKWADAFLNKIQVEKQLDLVMLLKL
ITLQYLSDCTPIILFDSFTEKKDNLLLEKLLTNFPIAYIHGQITEDYKVSLKISKEEMQPTCIS
YMLFMKDVMKSKDVIGEQNYERVVVVARSSQWRVFEFLSHEESRYFVNLLVVVQSERIM
AAHEEAPYILYTHKLYIDALGSSKPIVLSSFQDGNLTRNVDLFPKKIVDGFSGHRFIVAISHQ
PPYVISKGLRTTDGEKVFEGIEIRLVNLLSLLYNFTTDYREATEDSEVGSSEAVTRTIVKKKA
NIGIGGIYVTPDKLFRMGLTGWHSRDCASFISLASTALPRYRAILGPFNWTVWLALIVIYM
GGIFPLAFSDKLTLRHLLKNPEEMENMFWYVFGTFTNCFTFSGKGSWSKADKVTTKLLIG
FYWLFTIIITACYTGSIIAFVTLPVFPSVVDTARQLLSGWYQIGVLDKGEWQYLFLNSSDEV
AAKLLKNLDLVSTIEDGLKNTTRYSLWRYAFLGSRAQLDYIVRTNMTSKGKRSVLHISKEC
FVPFSVSLAYPLKAVYGDVITKGIESIKEAGIMNKIKGDVEWEMMRSATGQLLAASTGASL
KALSYEDKALSLEDTQGMFLLLAIGFVIGGGALVFEWFGGCYKICQRTRRGSNESIESNPR
THERQTKKQKKFNFQDNYAMLKKSFENKSFEHTPFENKSFENSALENQSLENNITYDESE
NFKNNVQNENVPPGACCENAPRNEEHIEKMIDTMFDDVLGEDNAEEQKNRRLSN
>MsigIR64a.1
MLQKSEMQKMFSYLLMCLMVINPIVLGYLDCDLITEFSKTKFVSRGTIFSCMKPKDTISLS
KCLMRNYLQFYINNNDDISSERFDKTLTITQSHLGVILDGDCEDWPLVINENTDRKYFFET
YHWLVLSRDKNVTNDLSSLKLNINAEMHLVPLDSYNEENDTYIIYDVYNPASAHGGEFKI
MEIGYFEKSTGYVITNYENKYWRRRNMTGTHFKSAIVVPQLDQPLRDYLESDENRQIDS
MHRFQAVTVRNCRDMYNFSVEMRRTDSWGYIQDDGKFDGLVRLLEKRIVDFGSSPLLFK
LDRMPFVDYGYGNWILKSFFIYRKPKVTTSSYEIFLRPLDETVWLLTLLTICIISILMKIVFV
NEVSLIPEEKVRVEDTTWSFLVLFTLGAFCQQSAACTPQFLSSRILAFFIFLFSILIYQFYSASI

VSYLLLDPPRTIFNLKDLRLSSLEVGVEDILIDRNYFVQTTDPDAIDLFEMKIKYSNNNSGF
YTPAVGLELVRRGGFAFHVETSTAYPIIEETFSNQQICEIEEVQMYRTQPMHTNLPKNSPFKE
MMNYCMFKQAENGNIDRLRKHWDAREPTCIEGAKKQEFNVSFKEFSCGPVAVALGILFG
MIFAIFEILIFNKHKIFDCLKSRMKAKKSDKTYPFIN
>MsigIR75s
MNLDNLGYVILANKHTFVMNLMCEHSIALLKQANKVKYFAFPYKWILFHTTPLNVVIEQ
YFRDLDILTDSDITLYYERENDTTLINKIYKDCARCLIKIEKVGYWSEGEGYHHLGMEKSM
ARRRNDLQRIILNSCLVITNNDTLNHLTDKRDKHIDSITKVSYILMQHLADIVNATFTYTVE
STWGYKINNSEWNGMIGQLLKKKADLGGSPLFLTLDRVDLIDYVTMPTPTVSHFIFREPKL
SYVTNVFTLPFDRQVWVSSVALVIILGFFIYIILKWEYLKKDDTTKQVTKDNSNVNMAEVA
LISFGAVCQQGSPTIPTSLSGRITTIILFVSLMFLYTSYSANIVALLQSSSSSIRTLEDLLHSRM
KVGVQDTVFNRFYFPNAKEPIRKALYEQKVAPPGRPPQYMSLLEGVLHIREGLFAFHMET
GVGYKLIGEIFKEHEKCGLKGIPFLEIIKDPWLAIQKNSSYKELIKIGLRKIQESGIQERENNL
IYTKRPKCSSSGSTFINVGLVDCYAAVVVLAFGIVLSLLIWTLEVMSYKRLKKLNKFRIRK
AQWNL
>MsigIRGluR2
MANNLLWRVFWVFCLFILGYVACAKTVVTIGALLEDLTSQINIPLNSTIYKKNMFDQRAYF
STKMMEVSATDSFEASQSLCRMLEGDMGVIAVYSDAVKTIPILESTCTNFEIPFFTTSWRNP
SSPKRDTESEARALLSFFPEAELYSRGLAEIVKSLQWNSFIIIYETEEGLVRMQEILKLQELS
PDSKKNNILVMELGSGPDYRPLLKKIKNTTEDNVILDCHNDKILTVLTQANSLGMLTLHNR
YFITSLDAHTLDFSILNTTANITTVRLHDPKSDDFLNTIHRWELTEFENHNRRIPLDPMAIKT
ETVLFHDAILLVTDTINSMSIKPGISNKPVFCNGTDVSEDGFTLRKYIQINTPTLTLTGPIKFN
DEGERIDFTIHVVDIIDETLIATWYAGNESLVLARDFNQTNNAVVSNLQKITVIVSSRIGKPY
LTWRSPTYEGEILTGNNRFEGYSLDLIAGIAKIIGFNFQFEITDKYGNWDPAEKRWNGLIGE
VLEKRAHLAICDLTITPERREVVDFSMPFMTLGISILHVQPSNEDVNMFGFLDPFSPSVWIY
TATLYLVISVVLFFVSRMTPGDWENPHPCEENPEELENIWDIKNSLWLTLGSIMTQGCDILP
KGISSRMAVGMWWFFSLIMTSSYTANLAAFLTKANMEPAIDGAEALAKQTKIKYGCMSN
GATMSFFKNSNFSTFQRMWMNMQQAKPSVFENTNDDGVQRVKNTNKGLYAFLMESTQI
EYEVETKCYLKQIGEWLDSKSFGIAMPMNSPYRSAINHAVLKMQESGELTELKNKWWKD
KQNATHCSEVEGEDGADGKLGLINVGGVFLVLGIGIVMSCVCALVEFLWNCRNISVEENIT
YAQALKAEVKFACNIFITKKRVKPLLSEAGSSEGSDKVDEKHDNKSMIQSILQSIGSNLNID
RSS
>MsigIR75q
MVILKLSVQSENHEIIRLIKDFVVKLNGPIKISAYVCWSKDDQLLLWKEMSNKSFACRIAK
FSDSARYLSPKEHQLFLLDSTCNGFQKILKKASQLNLFYRPYRWIIWGHIDYSMFQDIYFR
VDSQIFIVQKIQEEENRIDNYYNYSIKSLYKNFEQSPEFLNNELAKWSPIKGFIEYNPFSVSR
NRTNLLGTNLNISYVVTDSDTYNHLEDYRNSHIEPISKLDWLVMKCLLDILNATQTPIFQPT

WGYRFNDTTKFLGMIGDLQSGIAEIGGTGLFVTIDRIAVIEFVAPDAPTLLKFIFRAPPISYV
SNVFTLPFDIYVWYCCFALVPLIFAVVYLIVIWEWKDPIFKHKAAETHANAITPLRPRFFDV
LVMELGAITQQGTDSEPLSNSGRIVTVFSFIAFMFLYTAYSANIVAILQSTTESIKSTKDLLNS
RITLGVEDIAYQRYFFKAATDPVRKAIYQQKIAPKGQKPNFMTLQEGITRVQNEFFAFNVD
VSNGYKMIGETFQEDEKCSLKEIDFLSLSKTYLSIKKNSAYKEVVTVGLRKILESGIQKRH
VNRLYTKRPVCHGKGNSFGSAGILDCYAAFLIFGAGLALSLLLLGIEICYDKKVRKQFVVE
ITDHSSAVK
>MsigGluR1
MSRRWGHLGAAAVLFLGIVASKLDSISAASTLPPQVKIGAIFTEDEKNGPNELAFKYAVNKI
NKDKTLLPYTSLVYDIQYVPRDDSFHASKKACQLVQYGVYAIFGPSDPLLGAHIHSICDAL
DIPHLEARLDIESDIREFSINLHPTQHLLNAAFQDVMAFLNWTRIAIIYEKDYGLLKLRELV
RSPQNSGLEIHLRQANPESYQDVLKEIKNMEIHNIVIDTKPLNLQHFLKGILQLQMNDYKY
HYLFTTFDMESFDLEDFKYNFVNMTAFRIVDTDDLSVKEIIRSMFKSQYNREFRLLNSSFIQ
AEPALIYDSVFVFAVGLQTLEQSHTLKLSNLSCDKEQPWDGGLSLINYINSVERKGLSGPIE
FKEGRRIQFKLDLLKLKQPALVKVGEWHPGSGVNITDRAAFFDPGTMNFTLVVTTILETPY
VMMHTANNFTGNSRFYGFCIDILDRISQEVGFDYLLDLVPDRKYGARDPSTGLWNGMVL
QLMQHKADLAVGSMTINYARESVIDFTKPFMNLGISILFKVPKSQQAKLFSFMNPLATHIW
LYVLSAYILVSITMFVVARFSPCEWQNPHPCEIENELVKNQFSLANSFWFTIGTLMQQGSDL
NPKATSTRIVGGIWWFFTLIIISSYTANLAAFLTVERMITPIENAEDLAGQTEIPYGTLESGST
MTFFRDSMIETYKKMWRFMENRKPSVFVPTYEEGIQKVLDGNYAFLMESTMLDYIVQRD
CNLTQIGGPLDSKGYGIATPKGSPWRDKISLAILELQEKGEIQMFYDKWWKNSGDTCQRN
DKGKEFKANSLGVDNIGGVFVVLLCGLAIAVIIAIFEFCYNSKKNALNEKRTASVPHQSLC
SEMKSEFCFALKCSGSRQRPALRRKCSKCMSGVSYVPAMLDIPPHPPLPPVRASPRNRVSP
QINNSQRSGRDLFEQ
>MsigIR93a
MYYTVFACLFFLSIFKNGISETFPSLLTTNASIAIVIDREFLVEEFDEVKQEIDDYLVYAKREI
LKHGGVNVYPYSWTAINVRKDLTAIFSIASCYDTWRLFRLTEAEDLVHMVITDSDCPRLPP
ENAITIPLIQSGEELPQILLDLRSSGIYAWKTLVIIYDGTVNRDMITRVIKSLTQRRTSNAKAT
GISLMKLESNMSRTDLRAVMSTINPKVLGNNYLVIAGYRLVGVVMEYAKVLDLASTTNQ
WMYVISDTNRNTQDVQIFENLLKEGDNVAFLYNSSFTTNKCVGGRKCHMEELFKAFTRA
LDQAIQEEFDTASQVSEEEWEAIRPTKSERRDYLLKNMKNYLSKNGVCDNCTCWQMKTG
ETWGVEYQTTDQRAQPKLLQVGSWRPSDGAEMTDELFLHVAHGFRGKNLPMITFHNPP
WQILKFNESGDVVEYGGLMFDVVTELSKNLNFTFKLEIINKTNTGKSNATFYDTDNDLTN
EVPKVLLDLIKNKTVALGACAVTVTDELKQVVNFTRPITILTYTFLAARPRELSRALLFISPF
TLGTWLGLAAAIVSMGPLLYFIHRHSPVYEYKGYPMKGGLASIQNCIWYMYGALLQQGG
MHLPYADSARILVGAWWLVVLIMATTYCGNLVAFLTFPKIDIPITTLDELLAHRDTVTWSF
REGSYLERELKASNEPRYMSIFNGRAQHVSTDDDAIIQSIEDGKHIYIDWKMKLQYIMKK

QFLKSGRCDFVLGLEEFFDEKLSLIVAPESPYLSKINEEIKKLHQVGLIQKWLQDYLPKKD
KCWKNRHLIEVNNHTVNLDDMQGSFFVLLMGFALATMLLLFEKIWHRNFNKQRQKMIQ
PFVS
>MsigIR75q.1
MSDKNFACQICKINDDLKSSFPKEHQLFLLDVNCNGSEKILEKGNQLNMFNQPYRWIIWG
QTNSTVFMDFYFRLDSQIFVIEKLHKNPNNYLIKSVYKLFEGSSEFLENKLAKWCSTKGFL
DYKVLSFYRNRTDLMGRRFNISYVVTNPSTYNHLEDFRESRVDAISKLNWFLMKHLLEIL
NATERPIFQPSWGYPIPNSTRYSGMIGDLQSDIAEIGGTALFFTIDRVDVIEYVAPNVPTYITF
IFRAPPLSYVSNVFTLSFDTYVWYSCFGLVLLIFIIVYLIVNWEWKDPFFKQKAAKSHANAI
VPLRPGFFDVLVMELGAITQQGTDTEPLSNSGRIATVFTFIAFMFLYTAYSANIVVLLQSTTE
SIKTLEDLLNTRISLGVEDIVYGHYYFKTAEEPVRKAIYQQKIAPKGQKANFMTIPEGISRV
KNEFFAFHVEASNGYKVIGDTFQEAEKCSLKEIRFLNLVEPYLSIKKRSAYKEVIKVGLRKL
LESGIQRRHINRLYTKKPVCQGSGSNFGSVGILDCYGAFAIFGAGIILSLLLLTIEICFEKKCK
KEFNNEIRISTTTIKF
>MsigIR75c
MRVARTDYYDILVPVYKFRAAFIFVNPGKVKAGAEVLKPFTMETWCALMVIALTVGITIY
LGYWVEYRFVESKSKYSFCTSFLITISVIAQQGSAIEPFRTSGRIIFLTSLMMSMMVYNYYT
SSLVSSLLSSIPEAIHTIRELYESNLKVAVEPLPYTLTYIIQQKDDHYISLLNSSKIYENDVPHF
MNAAEGISKVKQRNYAYHTELATAYSCLKDSDQDLICDLAEIKFIPDGDIGMVAQKCSQYT
KIFQITLRKMQSSGLYKRAKDYWLSKKPECLLSSRVKAVGLGELFLTFLIIFGGIVISLVIFFV
EIFLHRQFSAHYGC
>MsigIRGluR3
MSTGVITLCLLVFQCAGFGTGLLDGDLKIAGIFEIIQLHQAAFTYSNNIYTYGKHPTDIAVS
HIVDSSIVKDEPFTALRGTCLFLKQGIVGLFGPQSSLNLEIVQSITERKNIPHFLTRWVNPSQ
MGRKTINLFPHPSILADAFLEILMAMEWESFTILYTNSEHLLKISDFIAEAKEWGILVYVEN
VDPSSTGNYRTVLRNAGRSGQKNFILDCPIEDLKTLLTQIQEVGLLTDGYHYFLTNLDAHT
EYLDKYMFSDAVVTGVHFPRPPPDVEVRTSKELCIIYNATFKQGCGTPELDIETGLILDAVR
IFLDTMKEEAIVQGQILSCDSDDSWNYGADIINTIKLGTYEGLTGQIRFGDDGFRNAFELTI
YQIRRGVVLERGSWNTTYGIGEHVVLNFDEEEVNKDEEDEELRNRNLNVLITLTEPYVRL
RESTKRLTGNNRFEGFAIDLIEEIARIVGFQYTFHLRTDNQHGQFDSLSGKWTGMIGDIIEG
KADLAVSDLTINKDRVDPVEFTQPFMGLGVSILFRKPSVVAPSFFYFAQPFSISFWQYLGISY
LIIVCSLFLIGRLSPNEWQRPETCKQSKKYLENDLTLLNCLWFVSAAVFRQTTNVKMYSIS
ARVISATWWLFCFVLLAMYISFSLSRNAIEEKEKLFENVAELLEYAEANDIKFGAKKGGAT
EAFFKNSKNVYYQQVAEYMNIHPEDMPATTGEGVQRAMQGDYAFFMESATIEYTIRRQC
NLTSYGGLLDQKGFGIAVKKGSPLLTHLNKAIIKLQSSGDLNMLKRKWWDEKYAGDACD
DDTGSDIAPKTVDHVNGLIAITFAGIAIALVMALLEFMVHVYQLSKKVKQPYGKAFSEEL
KKSFKRKHTVQNVEAIALTKTENGNNSQNNKEEA

>MsigGR1

MIVDDLYISIPTFEIIQPLIITSRMFGLFPISYKKIGNHYKLNWSIFYGAYSYFLSVGLSLLAVY

GMIADIKTDPQYSLRMTDDKTRFVTCGDISIIIIIVVFAAITLHMKIRKFWKLMNILNQADNI

IPFKNSKRIRKSSILFIAIVISTIALILISDIFLKASRKNMIAYVKRYFAFYILYCIVIMIEVFYW

HIIFLIKIRISLLNKDLTKIRDKAILNKNKILEKIVGKMYFDKIAATNSRHHTNQTFTGSAAN

TPASYVKSDESMETGKWLISLSIFQDKIFEATATINNSMELCIHVIMLSCLLHLIVTPYFLLSG

LFGDADIFYILLQTVWLLGHIGRLLIIVEPCQICINEHKLTSSLICELLTFEVEEEIKRAMTVL

SLQLSYCKLSF

>MsigGR2

MYTMNSNAHSGSKFINAIFIYSSLWGVTWKSSYLRYIKCYIFILLITFILAPSYLSYRKANNK

EFMKQFNFFYKLIQQAPTLFLSLTFIINIINTIFLRPKLEKILKCFDVVDTLLRRNYMSTFSVE

YGILFCINHLEIIAISIFETYSWIKVEFTQEYQSIVLRNIQYIQVNIAVGICCWLVKEIELRLKE

LNKRIEREVRLINNYADVSSMTTCEKMAVFNIKKVSRELKIMERVYNDLCGIVSIINDMFG

LTLLFYVLYIIVFIVGLLSNLLIIATSMFSFIDSSLKLNIKNSCIIWIIEILIKTSVLAIAGEHLSRE

ANKTIIICYGIIHSLDKNSHSNIEAVKEDLKFLIQQAVHRMPCLSASGFFSVNFTMMGFIIGSI

TSYVIVAVQLLTNPSM

>MsigGR3

MFPKNKVLSKDSKNKLCNLKFILQLCSIIGIASKTPHNKIYKTYVIILALFSVVGSLYSAYGK

MAFREISISMYITLADQISSVFLCLATVILSITAVFVYPEKFLETLKSLRNFDRLLNLYRPRSI

KQFTIYLICIHIMVLLPIFLDTWYWTFLFGLSVYKNYLVRNVQYYQLSIMMFLWFWLVME

TKNRFSRLNCILQDMVSTPYILLRTKFNIVVFKTNFSKTIETIKQISGLHNGLCDVVELINEV

FGKGLLCFILFTISYILCYTIVLIEFGNYYSGGEIGRYLRIMSSVWIIENFIKILALASAGEHLT

KEANKTIAICYGIKKCLDQNYMHNVEALKEELDSLIQQVAHRKPILSASGFFAANSSMLGF

IIGNRNTSLSFSG

>MsigGR4

MDFQVRKKGLLNDIMVIKLFYQIGSVLSIFPRNSKSVQILIQFIVTGYTIIAGVFVIFMYIIGE

NTVFGKILEVLIVGTFWVFSIMNMYQLFYKKYKLNLFCKWILYCDKIMGSVYVNKNIIFT

RFIIYTALLVSAIFPLLFYCATSAYLVKCLFYCFVFCQSVYITTFIFETSIVVTKRQEMYYERL

KSLWRCRNTTRTKYIKNLKTLKIIYKQIYFIVTQLNAAFNVHVLLIGTVTFLEILYRLEKHL

LGIGPIHFLKNICNLFITCALLGQVIMLAIAGDKIETTGLNITNFFYSLHMDLNDPLLNDLLS

QDTVRDFLIFLEKLRPTLTVGECITLNRKCILILLSSLIPYVILTMQIFSPV

>MsigGR5

MYFNNLRKSGAKDTLPLLLFYKCGAVMGLFPWNYKNGEILFQYILLILILADGFYTTFLSIF

IEKDLFSNIINDIDLGFFMSFAITQFYRLIRYRHSWKKLFCVFSLVDKNLNIICISKKLVVGRI

VSLPLLLVAVLLPFGIGTIERSIWFAAYYTVNYFHMVLIVLLIFESSTLIANRQKILYGRLKRF

LSIQHQNKIEYLKELKSLQNIYKSLYFLAMQLNKILGTTMFFTITLIVIEFLYELDHVSSNILS

NTEPFFYIPVLLILLVMLATAGDRIETSDLRIKKLFNVSRKNKSLLQEDLLVKAKMEEFSTY

LENLKPSLMLEGVTVINRKFFPIILSNITTYIIVWIQIKTTVDHV
>MsigGR6
MNIVGNDDLVNKTSLKLDFKYVVTSLQAIKYFALGPLAGNLKFVAFILICLFLPVSCYSLII
RYFFIDGFSVVLGKMLIAEGVFEIAYVLYCFIHFPFFAKKCKLNLINSIYELENEITLRKCQK
ENSHYLHLRLAVVLTLFGLLYSLTYYVHIINNNINACIVLITQIVAVICSFLIFTYYIAIANWLT
NKYDNFNLMIIDLCKKQNLKFEDGCDEIIKYLELGKNVLDLNNQLFGTAIFLNNCLFIFIVL
ELTVLAIDQTPSDLGLKLSQYIYPVPFVVLLVILIMACDAVEKSGNQVIRTCNLLYESLDDE
ANKEQLLLVETYAQQWRPVFSAAGFYEINQSCLSSIFSAIVTYLVIIIQFNMVLTS
>MsigGR7
MILFLQLSKRWSLLINTWCQMDKIMNTRYGYPPTLDTRLRICSGICISLGLIDYVLSIYNRCI
KMMVLYGDHNEYKYYFKDLFPQLYIVLPVNMVTAVYCLFLATHATLIEVANDLFIILMSIT
LALRFKQITNKLEENLLKMKSEDFWIEIREDYDRLSILCKELDDNISYIILLSYTQNLFFLLV
QFYQSLEGVYGLVGKVYFVFSFVYIIFKIVCVSLYAAWINDESVEPANILNSVSSSSYNVEV
RRLLMQISFDNVALTGCRMFKITRGIILSIAGAVVTYELVLIQFNSATQVSTHSGL
>MsigGR8
MFPWNLKKGSCGLQVVIAVIINILVVALTYIQNYQKNYSTTSVCLEMIHLPIYVLFNIFLFRG
LFTNGKQFRKIFQYLLSIDHILGKNHVKKTTIFGKIIFWSLSYLSIFFSMIPLLGEESGFFDFV
MYIVGLQMVIITVFLVEILLAVATRQEKFCLQLINFCKFRYTGKCEILGKLKNIKNIYININSI
VDEVNDIFGIFILLLLILCFTEILNDINSFLDLSPRDTEFANRFLLASILVLFLYCVISVSIAVFG
DKIEQMGIKISKLCYKLQSEAENIEIRDHLRQLSIFTEQLRPVLTALEFFDLNRNIVPALLTTI
TTYAIILIQLKM
>MsigGR9
MCWRYVKMSSLNNIEFETTDTFLLKLTFKFGKIVGIFPTNSNKIAHYVQNFCVTTLAVLGV
IIVMLFSFYYDDKHSVITTVLLIMDGTVLMIMNSVTTYQFYKHKHIWDLLFKMISTLEKNV
GTIYVSKILIILKLLFWWLVLSVSVYSFYAYFAVWQIFTNIFTTYAVIVNFHTTIITVFIYEVSD
FLTTRQDVYLKKLESFYLNQSIKGVELFRSLKYLNQTYTHFHITAKCLNNLFGTKILLIFIQL
FLDVLIALDGEMNDAQDKFNSGNATVQTMDKSNAGKPMVCDILYLIVLCILAITLVIAGD
KLELSGLKITFLFYTLRNKLTPHLHEVSLIKDQIKDFLKFLEKLRPELSVGGYIILNRTVMPF
LLFNITSYIIVLIQLKTPDFK
>MsigGR10
MKETPLFAELNLFLRLADHVCSLFLFLAAATTTINMVFIRPGKLRIIFEEINRFNNVLGGNL
GVLNLGCFLSIYHINAAILLTFDAYSWIISVTFETFKYYIVRDVQYHQMSILMFLWFWLAK
RIGKCFERLNEILNDIKIKRLCRSSKIDLYNKHTDISLFPDVEERLKTLARIYNDMCDVVDLI
NESFGISLLFYVMYTISFVVCYTMVLIIHSFVVENPDFDISKYLRINSLLWIWDNFIKILALA
LAGEKLSSEANKTINIGYGILNCLDKDPNRVDPIKEELTFLINQAVHRKPCLSAFGFFMANS
TMMGFIIGSITSYVIVAVQFLK
>MsigGR11

MISTLKDKNHKMHTTKKQNDQVEEKYDSYSLHHVLRWLFFIMQIVGFMPVQGVFKDRSS
DIGFSWKSLRTLHSYLTAIGLFFMTAIQISRFFLFKVQMVDVHRLWYFLKAFSISILFISLSK
DWKEFLKTWYKIDTAMLVFGKPISVKRRVRLLLIIFCLLFAGDYALIQHQRVAADIKKGHL
VLTFKSWNHIIRDFRFAYVFQIVPYNFVGGICLLISHLQVLFAGTFLDIFLMIISLSLAARMQ
VVTKRIQQVSRCTDVPQHVWIHVREGYNRMELLCKYVNSKIGYAILISFIGNLGILLIQLYN
SLHTKDTLMQQVYLYYAFGFLMCKMISVCILGSKINDESKKPLNYLYATKDSAYNIEIDRLI
RQISRDNVALSGMNFFQLKRNIVLKIAGSIVTYELVLVQFAGDFLKEHEIVNRTTVL
>MsigGR12
MGVLNFKFDKLCNWSCLNLLILNHNNIFFFTFVKSSTDSRTVFSLRALTNLYMILITTYYLV
LTNWLFNRYEFMNKFLRTTFNNKRHFLIVRKVIVSYKLANTVVQITNDLFGSILFANLTLC
VLNILYYFVLALDLNNIPIEQKIINYTAPLLYAIFLGTATMSCNSVEISGHSIIKTCYLLHEGLE
NDVDKDHLLLLIKYAELWRPIFTAAGFYDVNQSSLSSIFSALITYLVIVIQFNMVLS
>MsigGR13
MSERICNKYLIRDTLLLRVSFKVGAVFGLFPWSYSKGGVKCQSIIMILITGSILMNIYIHVSS
GRDAVKSALNIIYFTVHLSFTMHNFYRLIHCRNAWKYTFDLIDSLEKDLGVTYTSKPYFYF
KSVLYLSILVQFLYPDHPVSTIWMVPNAINFIFVHYHMIIITVFIFEFSSVLVRKQQIFQNHLK
ILWKNKYDTRVQFIKNLRTLEKTYRNFYFLADHINADFNVSILLIVVKVFFQFLFALHDSFK
NVFKERTVEYGSMVENVLYITTLFIVLVTLIIAGDRIESKGSDIIELCQTRDEGSLTENSSCEE
PVEAFIDFLDKLRPGLTAGGYMDLNRQLISVFVFTLTTYCIILIQLLT
>MsigGR14
MNIQNTNKNARKDTVVLKIIFKTGATFGLFPWNIDGEIICQSIFMAFTLICGTFILFQTLMKN
EKPLEKILRVLCIAPYFIFALINSHRLLYHRNEWKLFLKLLSSFDKKMSTTYISNRTLFMKLF
FHILLLAMMVCSLAVFYDDWSYVFDFFFITENCQSVILSVFIWEISSVITNRQKVFYNRLK
MFWKNPWINKVVFSKNLRVLESNYRKLYFSISFVQNIFGAHILLSLALVFFEFFFAVNRNLN
LILNGLPINVPETMSDLSHKCVNLVSIPK
>MsigGR15
MMVTNLKKSSNSDTMFLKWYYKCGVIFGVFPSISKKGIFYCQIPLLIIMCALICLTNFLYER
LRWTNHIDLSFVLRITNMVILLGMFCSSIRSLLLHRRTWKLFFSCLVNIDKVLNDQYYNTN
KIIFGKILLWVFAWIINVCCYAPYFLHRSLIIYLALVYHVVFTFELYIICAFLLETSILLTRREE
LFYTKVENFCNKNDSKTENIKTFIGVWKNIYLITKQANHAFGFSMVMIVMSYFTNVFINIE
DTLEISFKHDKIKLNINETIFHVIFDIIKLSTIAVVIATAGDKLEKTGLKISKLCHLLEIDVEDK
AIKGEMKDLCNLVDQLQPVLTVSGFFTINRNMIPMLVTSLTSYAIILIQLKS
>MsigGR16
MTHFRAEVIYISVVDQISSIFLCLATVTVSVTAVFIYPGEFLKTLESLWNFDQMTNVCRPCLI
FVINLIFVHVMILIPIFVDTWFCIFNFGLSPYANYFVRNFQYYQLSIMMFLWFCLVMEIRDR
FLRINSSLEKMVSTPYILLRTKMNTNNFKATSFPKMTEMIKQISGLHNGLCDVVEWVNDV
FGKGLMFFTLFTISYILLFTISLIVYGKFYSGGDVGRQSQYVSGIWILENFIKLLALATAGEH

LTRAANKTVTICYGIKKSLDQNCSDDTRVVKEELDSFIQQAAIRKPILSAAGFFVANSNML
GFIIGSITSYIIVAIQFIDSSH
>MsigGR17
MDKPVEINADMNIVKIFFKIGRYIGIVPLYSSTTSLLKKCLSVTYLIVITVLILIMFIFSIYDRQ
RLYSKMKLVHVVVDCLVVVTTIMFIITCRLAPLTNYKRFSVIYETTVLMERSLSAVDFKVR
RRAVMWSYVRLILLHVLYFVIHFYDFYYRASRQNSMLFLILYFPSLIGMYHQLFVTNLLSK
INGVIQLRYDFLKQMIEAYITREKNVAPKTVDVELGNKTVTLDQIFYTSKLLFKLVSNLNEI
FGWQMFFMLACTVLESLTVINLMTQDWLWSEIAVDIAYGATYIISTVMIVKSCDSVEKSAL
SILSTCYTNQETLKGSALEDDLFKFAELNKHILPQYTAGGFAVINQQVLSSLFSATLTYLIV
>MsigGR18
MDIVVIKFFYQIGSVLSLFPRKRKFGQMIIQLIVVTCTIIAGVFVLMRYITKLNNIWVKLLGL
LIAGTYCVFCIMNMYQLFYSKYKLDLFCKWISYCDKFMGAVYVSKIAIFTRFIIYIILLASII
LPVTFIYVKHGKLFRYFFYSSVYCQSILIAIFIFESSIAVSKRQEMYYEQLKRLWRCRNTTRT
KYIKNLKLLKMIYKRIYFIVAQLNAAFNAYVLIIGIVTFLEILYRLKMYFFSTRTFTFMDNIS
NGSLTCFVFGKAIILTIAGDRIDTAGRKITNFFYSLHMDLNDPLLNDLLSQDIVRDFLIFLEK
LRPTLTVGECIPLNRKCILILLSSSIPYVILIMQIFSVV
>MsigGR19
MTPVGEKRYLHSDIIFLKWCYKCGMVFGVFPSISRTKIFYIQILLMLILCIISFFAFYHYGRM
MSTNRVMGLSLALRLLHTLSLVGMFCSNFRSLMLNRITWKIIFACLAHIDKALDHSYITKK
RIFGKILFWICLWIIDLCCVAPFFRHTSLTVYLLFVFYTAINLELYVICAFLLETSILISRREELF
YIKVMIFCNKQYVDNITKNIKILDDTWKNIYLIMGEINRVLGLSILMIVLSFFTNMLTTIDY
MLQITQTQLRFQLTSFEMIFHACFDLIKLSTIAVVIATAGDKLEKTGLKISKLCHLLEIDVED
KAIKGEMKDLCNLVDQLQPVLTVSGFFTINRNMIPMLVTSLTSYAIILIQLKS
>MsigGR20
MSDRRVIILSNPLYQTIRPILLIAKLFFLLPFPIQKKGNDWWITWSHWSFVEVVLIFGLIVSF
GLYGKFQIYQLESVQPIRFQRLSSTLITISEDTTLISTFLICNVYSLMKFNYLRKYFIYINRVD
TILNFNPSTTKEPLKILATATVSMVYLLSVLSADIWIWLSLTQNEIEPLTYIKYQILFYVLFAF
LVIPALQYFMLVRCILIRLEWINYGLRIHFTNSPNRIFSDVALVKIGPLSRHFVTDQVLSKTA
KGRKHKINYADGYVLLVESNIALNNYFGFILLVLVFGNFLFLLITPYGLFMGILHKAYYAIL
PVSMWIVGHFLRFLLLVEPCNAIHVQVKKMSLIVCKLLNLNIDDEFRNQLNTLFSQLNHFP
IHFTPCGLFTIDRGLLMTSSGVNINQKNDMGIVARLKLNLMLYFSRIAMQENIILTDSLYQIL
RPILVICKIFCLLPFTVQENGSEWWIRRSWRSIFQGFFIITA
>MsigGR21
MNFFKFFKMSEEPIVLKNRINSSTFQEAMKFPLKLAQTFSYFPIYLGKREEQLEFKWFYWR
VGYSWVTFSFFVIEFFFVILDAFRYQLNLLDIKVIVFHLGAVVQYILFFKLTYFWPSFVKEW
AKVEFHMKHFEIMGNLKLKLGYLAGGIMFLAAVEHGLVNAYKLKQQFDNEPSFMEGFKT
FFTRTYEHIFRVMEYSFWLGLLIQFLNLQRTFYWNYTDVFIMLIGSALTYRLRQLSKKIKN

AAKVKVNDLVVWKTLRKDYTRLSELIYIVNERISGVIIVCFLMDLYFVLLQLYSSLRPIESV
VEKIYFYLSFGLLLLRIFCICIFGGAVYEEWKNIRFYLNTVAGSAYNAEVDRLVNHVATWEL
SLSGKNFFNISRGLILQMAGA

>MsigGR22
MQKVLIKDMLLLNIFHKVGVIVTLFPCNRSGIIVIQSILSAVFPILGLSTIIITLGNMEDMFVK
MLRIMTMGSFLIFTTTNFYFLIYNRNNWSRFFKLFAFFDTTIGISFVSRTKIFFKSIFYILFVTL
EFNFYLQSIRALHLTKSMFYVYTTVQAIIVVGFVLECSISLTKRLEIFYNELKILWQNQNQS
KIEFVKKIKRLRLIYKNFHFAIRHLNSSFGVSILWITLMSFFKCLLQLQQNMNSIFNGTATLA
TLYRCLAMFVIIIMLAVAGDKIDKAGSKITKLYYLLDLDLEDPLLEDVYLETYKQNFSMFL
YKLRPKLTVIGYITLNRNYAVILVSSVIPYLVLLIQLFAQK

>MsigGR23
MDLSPQLHCVYKSIFFLTRLTGISMFSLKEGAIKTSNSGIFHSFVLMVIYCLISGICLNENLK
NTSVADMKKTVNVIMSAVNIVFISVVSFTATTSREKHLKLIFHLSETETLHKRLQGTVDYFN
IKKCINIFYWKTFFMLSIKLLILVVDCSIKGVGDGGYGCCFISYSYPQLICGLFCIQLFSYIYF
VNIIFRFLNDEIDQLSKNSLDVVFSIRRKPSNSTIALKACSELYNKLTGIIRQINDTFGLACLA
MFFMVFTQLVIGIYYMNNVTEDFWDRPLSHLLRAVDYTINAVFICCLCNTTIEKGDMTGLL
LYKIDTYDDDLNKEIDIFTLQTINERAIFHASGFFAINNRLLFTVS

>MsigSNMP1a
MNLVQLEKLLLSLVVKFSSNRKMKMRLPIKLAIGSFCALFFIVLVGFILFPKMITGKVKKM
VNLAPGTEIRGMFTKVPFGLSFKVYVFNVTNPMEIQNGAMPIVNEVGPFCFLEWKEKVSI
TDEEDGDIMTYLSKDTFIRTTGPGCVDGQKVVTIPHPLILGLVNAVNRTKPGALSLINKAIK
SIYQNPTSIFLTAKVDDILFDGVVMKCGVTDFAGKAICTQLRNSGNLRIIDENDLAFSLMGP
KNGTEQKRLKVLRGTKDYHDVGRIVEYDGQTEMTTWPTQECNVISGTDGTVFPPLLTKE
DGLVSFAPDLCRALKAFWVRKTKYDGIPVSEYTATLGDSSKDGPEKCYCFTPETCTKQGL
MDLYKCVGVPIYASMPHFYDSDESYVKGVKGLNPNKKEHEITILFDQLTGGPVSAKKRLQ
FSMPLEANPKVDLFKNFSSTVIPMFWVEEGVDLNSTFTGPLKMLYTMKKVVNISKYVILV
ASIAGLIASAYLFFKDNEKITITKVTDAKKPPESGISTVNGHVNKAMSDNEIDKY

>MsigSNMP1b
MELSVKLIIGGAVLVVGTIVFGFLSFQTLVEFVVKDQTALRKRNEVRGIYLKIPFPLNFKINF
FNVTNPNEVMNGAIPILKEVGPYYYDEYKEKINVIDNDPEDSLQYDAFDTYRFNKTLSGK
LSDEDVVTIIHPLLVGMINTVSQTSPALLSIVNQALVHLFNNPKSIYLTDKVKNILFDGMAIN
CTGTDFASKAVCTQMKSLMKGIKESPTEKNILLFSLIGPRNATIADTLKVMRGIKNYKDLG
RVLEVNGKKEIGLWGTEECNRFKGTDGWIIPPLLKAEDGIKCYTTHLCRNIALFYVRDDV
LKGIHVRRYEGNLGDQTGNEEDKCYCPPPPRTCLKKGLFELTKCVGAPIVASLPHFLDADE
SYLTQVKGLNPVREDHVMNVNIDPMTSAPISVRIRIQMNLDVAPNQKISFMNNVSTSLHPI
FWLEDGLTLEGPLFIKISNIFVLLKMALIMKYVFLVASFGIMGYGGYLHFKTSKTVKITPVH
QRQAKDENAFNRSTNELIAQLRNEGKNGYTNNIMSGHEFERYE

>MsigSNMP2

MKKVLKSNCCSVKVLTIITLALLVVLIGVLVLSFYGIPRIIDTQIDASVRLKSNTEQWDRFQ

ELPIPIITKIFMYNVTNADDVLNGATPKLVETGPYVFKQKYSKNILNTNENEDSVTYEQHIE

ATFDPDLSGNLTLNDTVVMVNAPLLVLTQMSSSIEQLVTLNCLDKIFPAEYSTLFITAQIHTL

MFEGYLFAQNTADLGYACSIVRSLVIEKSRSIRNIQRIYNEDKPDVVEALKFSFLGFKIQKP

DGVYTVNRGIADVTKLGTIMQWNYSTHLPFWGTAQSINNDTCQLVRGSDSTLYPPKIHQK

KNFEIFSTDICRSVNIFYKGEGSYKGITGRRYEPDTNTFRSNTANDINDCFCTEKTLNKYGK

TSCFLDGVLDVYECFGVPLLLSMPHFLYADESYINGIEGVSPPDPEIHAIYLLVEPNTGTPLQ

GKKRVQINLVLRPIQNMPFTRGLNGTVLPVLWLEEGADLTDDLIDMLNSKFFNLVKIANG

VKYGLIAVSAAGVLVSGVILLRKMVLKW
