# Supplementary material for: Identification of candidate chemosensory genes in the antennal transcriptome of Monolepta signata
Source: PLoS One. 2024 Jun 7;19(6):e0301177. doi: 10.1371/journal.pone.0301177 (PMC11161048; doi:10.1371/journal.pone.0301177)
Supplement: S3 File — (PDF) [file pone.0301177.s007.pdf]

File S3. The amino acid sequences of Coleopetra insects were used in phylogenetic analyses

OBPs

>PyasOBP2

MMVKIIGLLLLVIVIGGNAKLTLPPELQEYVDDLHKICISKSGITEDDHQAYDILNKDQKM
MCYMKCLMLESKWMKPSGEIDYEFIESQPYPFKDILMAALNKCRTIENGADQCEKSYNF
NHCMHKADPENWFFV

>PyasOBP5

MGRGSNGKSVLVYFLISLLSLTAADQRQKIVDFHANCLDEHGVEEVALHEALDGRPPEEIE
FYNHMFCVAKKSNVMSEDGVVNLDNFEVDMKDVIDEDNMENLVNILKKCLIQKEDIMST
VRDAVACFTKEDHRL

>SzeaOBP1

MVFLKNGILVLVIAGCVMKVVLAGTNDPQRQKIIDFHAECLDAHGLDEEAMIEALDGNPS
EDDSFYHHLFCAAKKANVMTENGEVTLDTFHIDMAGVIDSHNMENIHNILRKCLVQKAD
IMTTLRDAVQCFIKEDHNL

>SzeaOBP28

MSQFTKYFVFLLCASSTLAMFDESTLSDDMKKMFKILHDICVNQSGASEALINKLKIAEFP
EGDKNIKCYVKCLLVQTGAMDLQGNIDVEAAMDAIPDALKDGIRVGAQHCVAKFDHMT
DHCQKAYLLVQCLYEVDAEAYMMV

>AmalOBP3

MRKFLFLVSCAFMLNKIEATPMNEAQLQNAAKLIKNVCQPKLKISDKVIENMHNGDFAEN
EKLMCYLECVFRMGQMMKNGKFDEKAALAQIPTLPPERQQPTKDSIKKCADKGQGDDK
CVAAFETAKCIYFDNPQNYFLP

>AmalOBP8

MKSLIILCIFGLFVSTKARLTEEQRNQLKTIYATCEEKTKVDKEQIKLSHEGKKTDDPKLNQ
YYYCVLTKLGVVDGESKLKFDVVDMDLSKEQKQNLQQIVDKCKNERGKDKYETAKLLF
ECYWKTTSIRTSVK

>HoblOBP1

FPAMRSNIKMFKNSALFILGIILPSVLCMSEEMEALAKQLHDDCVAQTGVDEAHISTVKDQ
KGFPDDEKFKCYLKCLMTEMAIVGDDGVVDVEAAVGVLPDEYKDKAEPIMRKCGVIPG
ANPCDNVYQTHKCYYDTDAKSYMIV

>HoblOBP2

LTLHSITGFKMKYFVVFAALCAYVLGDTLQEHSQKVMEEIITVGAECAEKLGATPEDIDKL
MKEQIPDSRAGLCVVSCANKKFGLQNADGTVNRGSTLVNIEKVKDIDNDMYKKMSSVW
STCSENTPNDSDECNTGVHLVKCMKEEGAKLGLNKETMGF

>HoblOBP9

MKISLFCFALCVAIVNASIEKYIDQLTTEIATAKTECAKQVGASVDDVVEVSQGKTPTSKEG

KCIISCVLKLFGGQDSNGKINKHAVIGKIEELKPIDTDVYEKFSSVWKSCSEKTSDDNDGC
DSAAKLMICLAQEVDKHGISKKLIGL
>HoblOBP13
MKSIIVLFALIVISAGLTAKEEEKIKEFAKECRTSSGIEREKLEKLRNGDIEDDPKVKDFISCL
FKKLSLQKSDGNIDQDIFKSKLSSKLTEAEKETIINDCINLKGSNDSETAFLLYKCYRSKTPS
HNADLLV
>HoblOBP24
MNMKYNIVLLLLCVICTYNEALLSQTDQIEAAKLCANQTGIAPKSIAKLTRKLSVPKKDPA
VKFFLVCVYKYVGLQDQNGDMNFQKIREQLKNDFFKYDTDTIVDNCSSVKGNTHEDNA
YLVTQCLLKQIRKLSKSNKNKD
>HoblOBP5
MDQDFLTKATARMSQYVEECAKDAGATKDDLAELMEIRIPSRKEGKCLLACYNKKYGIQ
DKDGKLDKEASIEAMKDLKLADTELYDKAVKLFDTCIDQVPNQDCECNTAAIFMYCFNIN
GGLMGLQPGMVPM
>HoblOBP8
MMKLQLVLVLLLGLCLQVSSAINEAQMKAARKLIRNTCRTKTKISDEQIEDMHKGIWND
DDDVTKCYTHCCMGIMKMQNKHGGFDRELAAKQYPQIPESMRESLVSSLEGCADSGEGL
TKKCDISYAFFKCVYFSNPEEYILP
>HoblOBP3
MMKVPLVLVLLAGLCLRISCTITDAQMKAARKLIGNTCRTKTKISDEQIESMHKGIWPDD
DEVMKCYTHCCLATMKLQSKDGGFDKELAEKQYPKFPESIKESLIESLEKCADSGEGLTK
KCDLSYAFFKCVYFYNPEGYIFP
>HoblOBP4
MTMFLYFLIVNIFLVETTQGAMTNAQIEATQRMIRRTCKSKMKITSDDELDGMLNGKWDN
LSPATLCYLHCCVKMIKMVTGDGHVDYDSNVKQINNLPEPKRHPLTDSLNNCKDAGKSL
TDKCEIAHEICKCFYFSNPGAYIIP
>HparOBP15a
MTSTLIGSLVLISFLCIGASDPDKEKLREAAKSIMYDCKGKVGASDADVQSLLDKTIPTTK
EGACLLECIFTTSKVMKDGTLDKDATLKTLEVVLKKDKDKEAKVTQILDACQKQIGKGT
DDKCQTAKMIADCLQKQGKLAGLSPSS
>HparOBP1
MFKNTALLILGTFLPTVLCLSEEMEALAKQLHDDCVAQTSVDGAHITTVKDQKGFPDDEK
FKCYLKCLMTETAIVGDDGVVDVEAAVGVIPDEFKDIAEPIMRKCGVIPGANPCDNVYQT
HKCYYEMDPKSYMIV
>HparOBP2
MKYFVLFAALCTYVLGATLQEHHQVVMEQIINAGAECAEKLGATPEDVDKLMKEQIPDN
KAGLCVVACVNKKFGLQNDDGTVNKGSTLVNIEKVKDIDEDMYKKMSSVWSTCNENTG

NDSDECNTGINLLKCMKEEGGKLGLTKESMGF

>MaltOBP1

MSSSIFLVVVFFLLAVSKVEPTMKRSEFPPKVLELADALHSTCLPRSGTDEESINKVIDGEF
TDEPKIKAYMQCLMDESELVDENGELIMDLIIPLTPPKIFDEALKNTKFCDGERKEVKERTD
KAFVFFKCIYGKNPDTFIFF

>MaltOBP6

MKTVFVFLCVIVGILAQDKKLVAEELMLEHIHDECQANPATYANHDLLHNLAANIDNPQV
GAHMLCESTKVGLQKESGELDIETIKSKIGLSVTDPERVEFLVKECAIKKNTPEKTAINLFM
CLDKNGVTYFHEF

>MaltOBP5

MNKLTVLFYITFFAAVHGLTEEEKMAIHEDCFSQSGVSEEMASKVMDGVFVDDPKLKLYI
LCFAKKVGIMNDSGDIQVDVFRAKLGTKVPDEVKLNEIIAKCAIKKGTPEETIFALSRCTH
GNKS

>MaltOBP4

MKFSVFLLCLCLVVPYVYSALSEKQMNATKKLMRNTCQNKAKPTSEQIDAMHKGDFSG
DRNAQCYLLCLMNTYKLLTKENTFDWEGGIKALEANAPASVAGPGTISLKNCKDAVKTQ
SDRCVASMEIAKCIYDDNPSNYFFP

>MaltOBP3

MRATTEIFLIVGVCAALVSGMSDEMKDLLDSLHAQCLSDTGANEDLISKAQKGEFTEDEK
LKCYMKCIFDETGLFGDDGQIDIDGMIAMLPDEIREPFSPTVRKCGGLLSGSPCQQSFMLY
KCCFDEAPQYYFLP

>MaltOBP2

MKAVLVLCVVVAGALAQLPDSERIHLKQVHDSCQADRATYADESRLKQLNKYIDDAVVG
NHMLCMSKKAGLQKGNGDLDIGVIKQKIALVTADKSKVDGLVKKCAVVNGNPQKTANL
LWLCFVQNNIDYLHRL

>MaltOBP13

MDSVALLLYFFLYVIPSKGISLTQEEVKQAKLEAHASCLETTGVSEDLVMDIKREGKFSND
ENLKCYVKCVHDYLGLMADDGTMDYETIIAHIPEEYQVKYASRIRACGTIYGSDLCETAW
LTIKCYGEGISQLPHP

>CbowOBP1

ELTEKQMKATKKLIRNTCQNKAKATTEELDAMVKGNFNQGKNAQCYQLCILNTYKLLKS
DNTFDWQAGVNALKANAPERIAGPGSASIKNCKDALKTKDDKCKGATEIAQCIYEDNPE
NYFLP

>CbowOBP2

LNEKQMKAAVKMVRNVCQPKFKATDVDIDKMHKGDWNIDHTAMCYMHCAMNMYKL
MNTDNSFNYQSALAQLNQLPDSYKKATEICMEQCKDSAVTLSDKCISAYELAKCMYFCN
PEKYFLP

>CbowOBP3

MRAAKGDYQDDMKLKKQILCFNKKVGLQDENGDIVLDVAKSKLFDIVKDEKKTMDILK
KCAVKKDTPENTAFESAKCLHKLAPEEKLVI

>CbowOBP4

MKHREEIGLECLRQVNIQRDTIENAKATLNFPEDRKYKDFLACSYKKQGFQSQDGVILYN
SIKDFLSRYYKRNDLKVMDNCKENIREDHGEMALNALRCIMDNLKNMEEKSRR

>CbowOBP5

KPLSEEGREKAQKINEECAKESGIEEDNLEKILADEFPEDDKMKEHSFCFLTTLGVMDKDG
KIDKDVMTDTLKLFAPEGKEVEIMEKCAVETDDAKETAFAIGKCVHEQVKSV

>CbowOBP6

GQLPEDEKERLRQVHLSCQADSKTYCDEDLLRKLGDNVNNPQVGIHMLCMSVKAGLQE
RNGDLNRSFIKSRIALVTEQAKVDGYVQKCAVKKETPEKTAAMLWLCFVQNGINYYHKL

>CbowOBP7

LSEEMQELADMLHATCVHETGARQDDIENARKGIFAEDEKFKCYIKCIMAQMACIDEDGII
DEDATIAVLPEEYRNQAEPVIKKCGTKKGSNPCENAWLTHKCYQNEAPEDYFLV

>CbowOBP8

VGKFPDGRPYPDGFEDCLKSSNAKLEEVLNKPKANISEEVYCFFKCLSERVGFIDQQGNV
HIDKMDVTQIFQGAVEEVPDELKSCLGGVNKVESCQDMSKICECFLKMAP

>CbowOBP9

VRNTAGKITQNESAKKTLGNCKTETGATMADIESLKEKKIPKTKTGRCFMECLFSKAKIM
DNGRFNKKGMVVAFTPALKGDLTKMGKLRELSEVCEKEIGLNKLENCEGGKKIVECVAK
HGKSYGMSFSTTK

>CbowOBP10

EKKLPIEAEECLKITNTDLKDMMAHPKEMSESHYCFFKCIFEKRGIINKVDGTVVPDVLDD
IKDVAVLQVASEEKLAELKKCMADVEKIEKCTDMENFRVCFDKLMS

>CbowOBP11

YITEEGWGEPLIALANSLHNKCVPITGVTQASIDQVKEGNFIEDEKMKRYVLCLWLVSEVI
SEKFELNTEIFKLLPKKLQDGHNIIGCTKKINGTDVSELYEKTYSVTKCIQKANPDEFIMF

>CbowOBP12

LECGIAKANRNEIRQALSMCVKNNDTLEDILEMSSLSSSTTSSPTEDSDDEDSQEDTIKSTS
STTKSPRRIKSSRIKRARSFSNTKQYASKATERNREESNNSNNTNNKIDNLKDAQKDDSED
DNEVSQETPKKRQDMSENCIVHCVLEHLNLTDETGLPDHSKLSEELLKTASGRELRNFLQ
ESTDECFQEVNEENDLDSCSYTTKLITCLADKGKSNCADWPAGALPF

>CbowOBP13

GVTEEQKKKIESYHKECSKQTGIDEDLVNKARNGQYTDTQILKDYLFCTSKLAGFINDNN
ELQKDVILQKTSVSTKDSAAAQKMFEACAVPQKNGPETSYHVLKCYYEKSGLSLV

>CbowOBP14

APSDSSNSTIKDYCIKEVEISEEKVNKFEKNPDDTPDEDIMCYTHCILVTLGVVDDDGKIIIE
KFTKIFDKYDMECVKKIPKILECTDLINLNKCAATDE
>CbowOBP15
EETEKEKMKRIHEECQSDPATKVEESVLKAAEEGDVDVTKIGPHTLCMNVKVGLQKENG
DIVKDELRAGLRRVPGVDESKIESIVEECGQREGGTAEEAAIKLFQCLQKRSKITHHHHHH
E
>CbowOBP16
YLSEEDYGPKLSAVANKVHNACIKKHAVNEDTIMQVRKGNFVEDELIKKYISCIWLLSTVL
DESGNLNIKIINDLCPPKGKDTLPKIYHDCHAENAGVSQLDEKVYNIMKCWYEKDPELFF
VL
>CbowOBP17
IELPSELQEYVEDLHKICVTKSGISEDDHAAYDVKTNPHDPKLQCYMKCLMLEAKWMNP
QGDIQYDFIIDTSHPQIKDLLVAAINKCRAIDNGANLCEKASNFNFCMYDADPVNWFLI
>CbowOBP18
GKNQTNNCDIPASAPKRIEETINSCQEEIKLAILTEALEALNVNEHIHSRAKRSAFSKDEKRI
AGCLLQCVYRKMNAVNERGFPTAEGLISLYTEGIRHKDYVLATMQAVNHCLYHVQKNHL
TTPQSIDEHGKTCDIAYDVFDCVSEEIGKYCGQTP
>CbowOBP19
TDREDKMKIHNVCVAETGVSQEFIDKMIAGEFCDDTNFKNYLVCFLKNDGVFLDNGELK
ADGANRQIREFADDEDTVSGFMANCAVQMATVEESAFHYSKCMYNTLYG
>CbowOBP21
WMSDDKFGDKLEKMYRLWHDDCMKKTGAPENTVELIRSGIFPDDPRMKAYNRCLYTDV
MDKNARLLPEKLDYYIYPAFGKTGLKMYLDCEEKVKDEANYDDRVYKMQQCIYEANPD
VSFNFF
>CbowOBP22
QMVSPSQLNTILQYHTECREKTKLPNSLVTGLIAGQFPNDPVLKSHLLCVHQKLGVQDAD
GNLRKEFISETLGAVLPASVDSKELLNKCAVQKSSPEDTALDLDRCLYQTVQPMRG
>CbowOBP23
LSEENMKEIEEFQKSCVAEVHTSPDVLSQIMSGDVSDDPKIKAHLLCFAKKAGVMTESGE
TIMDKLKQKLNQYLGSKADGFFEKCNIEITTPEDTAFNVYKCLSEMLQEGK
>CbowOBP24
MNVHAGLQKPNGDIDKDDLRRALSEGIRDVITVDDIVDDCGQRVGSTAEEASVNLFRCIF
GHSNAYVHEWKPSMLRQTSGAEGFFTSSLVLSVILATLSVRLI
>CbowOBP25
YNFEDTEFNQILANDLEDVYSFTYSHPRSRRDDKAVEEDKCHPPRRGRPLCCAEETMRKL
HDDKKEIKRACFKEITGKEKPERPDRHHGPPFDPFSCEKIEQHRRDMMCIQQCVGEKLDY
LDADGKPKPEQFEKYVEGIFEKEDYLLPLKDKIVSVCLDEAKNATEKVSSDPCKSTGLVLE

HCIFINTQLNCPEDQIKDKKMCSKFQDRLRQGFDKRSSPSPEAEDE

>CbowOBP26

KVDPKVIEEIIEEFTETVAKCSDEINPNADDIAALTEMKHIPDSHEGKCMIYCIYRSFDAVEE
DGHVKFEGGMAFLSKIKESDPDMFDKMSAIYKKCTETDYFDKDPCISSANFVSCNIKAGK
EANISSDISSW

>TcasOBP01

EDDDRQETIRQYRDDCIAETKVDPALIDRADNGDFTDDAKLQCFSKCFYQKAGFVSETGD
LLFDVIKDKIPKEANREKALAIIDKCKELKGADSCETVYLVHKCYFLHSYGTDKKTE

>TcasOBP02

LTDEQKEKIKNYHKECSAVSGVSQDVITKARKGEFIEDPKFKEHLFCFSKKAGFQNEAGDF
QEEVIRKKLNAELNDLDATNKLIAKCAVKKDSPQQTAFETIKCYYENTPTHVSLA

>TcasOBP03

LTDEQKSKLEEYSKECLKESKVDESVLKEAEKGVYLDDPKLMNHVYCLVKKINSQKDKG
ELEVTQIKEKLMMQINDEKEVDKLIQLCLVQEKSARYSLGKCEVSS

>TcasOBP04

FNNPEDELRRSAACLEQSKVSSESIKNLQIGNFDDDERLKEYLFCVSKNAGYQDPAGHLQ
HEMIRLRFKGGRYSDDTINEVLQQCGHQKDTPQETAFQFMKCAYQNAFPRNYK

>TcasOBP05

LTKEQIDKLEPISKECRELNGISEDTILKVRRGEAVNEPKLKNHVLCVSKKTGLASETGETN
VEVLRTKLRKVSENDDEVNSIIQKCVVKKSTPEETAFEIFVCLRKVKPNFSPAN

>TcasOBP06

LSEQQTEKLNQLSKECRALTGVSQETITNARNGNFEEDPKLKLQVLCIGKKVGIMNESSQI
DENVLKAKLRKVSDNDEEVNKIYNKCAVKKPAPEETAFETIKCVMKNKPKFSPVE

>TcasOBP07

LTKEQKEKLDKISKECKNQSGVSQELIDKARTGELINDPKLKAQIYCVSKKAGLATEAGEI
NMDNLKTKLKKVAANDDEVNKIIQKCVVKKPTPEETAFEVYKCLHANKPNFSVVD

>TcasOBP08

KQDFHKKCLASSGANADTIAKVRNGKFSNDPQTQKYFGCMLRSVGVVNQAGQLQVAAL
RKQVPKDMKRDEAMKIYMSCKDKKGANNDETAYLLYKCFWEASPRHVKIDGQ

>TcasOBP09

FSLSNREQAIFLSTYSTCLETSKVDSERALRTASGIIDDEPKLKEFLFCINKQNGVQDDAGN
FVKDAVRKRIEHPLLTDKTMEIIVNKCTRKRETGEETAYQFLKCSYFTIMNEKHQ

>TcasOBP10

QHLTEEQKNNWRKWSNECKVLIGVSQEAINKIRNNEFDSVDDKIKKHGLCFAKKASLAD
SSGNIIINQIKIKLKRVIEDDEEVDRIVTKCTIRKNTPEETTFETFRCLRENSSKFVPV

>TcasOBP11

LENEGQNPDTANCVALGGQRIKDSEIAKMAHCILTKTNLMTDKGTFNSNLLKERLRQSVH

SDELVDKVVMMCTVEKETPLKSAFSGYKCLRYLVPWFPLD

>TcasOBP12

KKCFLAEDTDKLEVMINECKTKTGVPDDILQKARNGEKIDDPKLREHALCMMKKSEMM

NDAGEMQMDKIRARIKHAVSNEAEGTRIMNECAVKKDTPLATAYEMICCLIRNKNSVDE

>TcasOBP13

ETAKEKLRKYSDECKSVSGVSEELLNKVRNHEDVHDPKLDEHGFCILKKAGFMNEAGDI

LADTIKTKLKENSEHPDTVDALVEKCNEKKDTPQHTASHLFTCLVDKKVHSH

>TcasOBP14

RKWFDKDPQDVAKWQKECFEASGVSMESMNKLPNITLSEDPKLGENAFCLLKKLGFISE

DGTLLIEKLRTSLKNQWGDEIANKLVNECARQKSTPQETAHEMFLCIPAKLK

>TcasOBP15

KQQKQDTLDEEKEKMKKWTQECIQESGVTSEILQQLRNQKRVEDPKLKEYTFCTFKKNG

FMNEDGKLQYDVIKSTLMKVSGSEEEANKVVKDCVVEKSTPQDTAFETVDCWYRYKKN

>TcasOBP16

EEMQELVNQLHSTCVAETGVSEDLINKVNSDKVMIDDEKLKCYIKCLLTETGCISDDGVV

DVEATIALLPEDMKAKTTPVIRSCGAKMGANPCESAWLTHKCYLETSPADYVLI

>TcasOBP17

EEMQELANTLHATCVDETGVSEDAIESARKGNFAPDDKLKCYMKCIMEQMACIDDEGIID

VEATIAVLPEEYQAKAEPIVRKCGTKIGANACDNAFLTNKCWYEEDPEVSLQLN

>TcasOBP18

IEMDDDMKELINNLHNTCTGETGATDDQIENARKGNFAEDDSFKCYFKCVFDQMGCMTD

DGKVDSEAVIAVMPPELADKIASTVRGCTEVGANPCETAWLANKCYQKSNPDVSKVSSN

VRSD

>TcasOBP19

EIVVPDDLKDYINELHDHCLKEMGLTEGDHKNYNIHVKDPKMMCYMKCLMTTSKWMN

MDESIQYDFILSSVHPAVKNILLPALDKCRDIPKGTMECEKAYNFNMCLFNADPENWFFI

>TcasOBP20

EIDGYYDICYKQIGLTKDDLKAYKIGDRDPKIMCFMKCVFVEAKWMDENENLQYDYIKN

TIHHSIRHITLPELENCGKKAEGDKCEKSFSFFNCMNKAEPEDWVLIQ

>TcasOBP21

EIDEYFEQCFEPNGVTMDDIKAYKMGDKDPKIMCFMRCLFVSGKWMDENENMQYDYIK

ETIHHAIRHITIPELENCGKEAQTGDKCEKSFNFFMCMNRAEPEV

>TcasOBP22

EEDNVGKIESVEKKCQEKTGVSEESLQKIMRLEEVDDPLVKENALCTLKAYGVMDDDGN

IFPDKFEEKLKPEIGADEAKRVAEKCAVKKDSPEETAHQTLWCATEENALTDTSQEQ

>TcasOBP23

DEDNLNTENVQSIEEDCQKETGVSDESLQELSETGDSDDPLVKKNALCILKAYGVIDDQG

EISEDKLEEKLEPDRGKEEAEKVAKSCAVKKDSPEETAHEALLCMQQKSQK

>TcasOBP24

TPSLDDFKKVQKDCQKKTGVSDESINKVNNLEPVCDDLLLQENALCILKTYEVMDEEGKI

CPDKLMEVLEPKFGKEKAEKLIEKCTLEKDTPQLLAHATLFCLSVQKYVV

>TcasOBP25

QFLQKIKKVSEDCIAETQATKNDIKTLLEHKIPDSHEGKCMIFCFHKHFQIQNEDGSLNKVA

AISLLEPIKDHSQDIYDKVVKIFNTCFDSAERDDDSCIYASNLAECAIRESKSVSVQ

>TcasOBP26

VDQEFVEKFLQKMEKIGEECAEETHATSDDIADLIEQRDPKTHEGKCLIFCYHKKFNTMK

EDGSLDKVGSVLALEEVRDADFELYKNILTIFVTCGDKAKIYDDPCETATALTMCGRDEAK

AVSWAYFA

>TcasOBP27

FLEKMQEFGAQCAEETDATSDDIAELIARKLPPSTHEGKCMIFCMQKKFNMMKENGGIDR

AGAIAALKPLQKADPELHQKVLKIFVTCGMRVKPSPDPCDTATELALCGKKEAEAVFCRL

GWKTLS

>TcasOBP28

QDFIDKFVAKVKSIGETCVPETNASKDDISSLLAHKMPDSHEGKCLIFCFHKQFQIQNDDG

SINREGAIKALEPLKADDAELYEKVISIFKKCESTPVDGDSCLYAASLAECAVKEGRAVSFW

KNTNFKLI

>TcasOBP29

MDESFLQQTRDRVKAIVKECVTEEKATDSDFDDIMALKIPTSHEGKCVFFCSHKKFNMQH

PDGSINKEGALDTFEVVKDVDAEFHDKVITVYNHCLSTPVDPDPCVYSVNLFQCFMKEA

KAVRK

>TcasOBP30

IDKEFVQELRQKLRSHVEACAKEVNAGPDDVSAIFAHKLPATHEGKCIFFCMHKLYNAQN

EDGSLNMAGALANLELIKDMDPDVYTKVSTSFKNCESAPFDSDPCLYAANLVTCIVKEGR

AVSNNFSGFFF

>TcasOBP31

GVSIILDPKFLEKLTQEVQAVGTSCGEKEHATADDMIEIMEEKFPPTSHEAKCVVACFYKH

YKMMKEDGTFDKDAAVKAFDEIKAQDAEIHAKILKVIDACDAKKQMSDDHCVSAASMA

GCVKTEAIAV

>TcasOBP32

LSLSATVFGQSLSEDEMRENARKLMTSCKDKVGASDADVEALKMHQMPESREGFCMLE

CVFDSAKIMQDGKFSKSGMIEGFKPLIGDDKAKLESLEKLSATCESELGDGEDKCETAKRL

VECVIKNGKTHGFEVPPPRE

>TcasOBP33

EENDINEIRSVEENCQKQTGVSVEKVNNFELVDDPLVKENALCILKAYGIMDEDGNIYEDK

LKEQITSELGEKNAEQVAKKCTIKKESPQETAHESLWCVGEQKPIPGASPDEKN

>TcasOBP34

DESVYLSNHEACVKLSGVDETLLETIYEGDVFEDMKFKTYIHCFFKKSGFQDENGVMHF

DAIKSSFHKDFSQTENIDKTITECEEKKLNGESALETAFLHFKCFMGEL

>TcasOBP35

QMKAALKLVRNVCQPKTKATNEQIEAMHTGNWDLDKNGKCYMWCILNMYKLIGKDNS

FDWEAGIATLKAQAPESVRDPAIASVNNCKDAVKTTSDKCEAAYEIAHCMYLDNPEKYFL

P

>TcasOBP36

AMSEAQLKAAVKLVRNMCQPKSKATNEDIEKMHHGDWNIDRTAMCYMHCALNSNKLIT

KENVFNRDYAITLAEKNLPTALKTASIEAANLCKDSAKTLDDKCVAAYEISKCLYESNPEK

YFLP

>TcasOBP37

AILEDSELMKVVENCVKKTNANESEFSSPNFLETTPSQPALCTAKCLLESLEIVNSEGNINM

ETLKEYAQPFESPAREAVATCGEEIKSVTTCDDMEKYRKCVEPLIKNS

>TcasOBP38

EVREECLSENSMTMDELHEGWKMENLPESHLCFLKCLLEKREVIDENGVPQKEKIDEILT

VKQLSDEKREEISTCITNVEKIENCETMSEIMRCFPKKRRD

>TcasOBP39

LDLMADKNFVELRNKCLDKLGLKEEDLRDLKFDGDVSEDLMCFGKCIQEEDGLLDSEGN

LNEEKLEKKIETMPFLSRVSDDTKNNIMECLKEIGKIETCQDFGKQRDCIHKYV

>TcasOBP40

EKESEEAQIFTELDGPAAELRDQCLEKNSMKVTDLKTYNTSNDIPEKELCFYKCFYEGVEF

IDANGNLNVNNMKEIPAISELGDEVLNEITACVEKIGKIRCCGDLRKIEQCYQNITM

>TcasOBP41

QGKYWTTISECLTEHSMGVEDMKKFDLPAEKMSEEMLCFNKCFYDKLLITDENGEINTDN

LMSIPLVNAIDASKHDDLVTCLKKVGKIEECDGVKKIEQCFVEFI

>TcasOBP42

LCMNETGVSEETARNYKPAEDPASEEILCMVKCIFEKIGCLKDDGSFCVDTMKKKNYIMD

VINEENEEKIYECLRGVGKITNCRDMAAVEECFVKNDSK

>TcasOBP43

RSFSHDELDTDLSFIKTCNRTSPISMSKFGLFLTEFNLTEPGTMNECFLHCLFMKYGWMDS

DGGFLLHDIKQTLEESDVEIASLEFILYKCTATESNNRCERAFVFTQCFWDKMAEQQPSED

QFFYNIEDKK

>TcasOBP44

QPEDRHQIALQCIDIVGIDQKVVEDAINIEIPKNNPKYKEFLACSYKKQGYQNENGEILME

NIKKFLQKFYHPSDLQELNSCSGHNGTNHAENAYQALQCIYNRLSNMTVVGN

>TcasOBP45
AIRPWRTCGTWPPCPPNGKMLQNFRIKRASVRLTNTETNETTPEPKAVSSEAQATENCIIQC
IFDNLQMTDSTGYPVHTKILDGLLKNTTNRELRDFLQDTTDECFQVMDKEDTMDPCSYS
NKLVTCLAEKGRSNCADWPVGELPFKP
>TcasOBP46
MLWRRGRFLYGENLDMFDPAGLQACMKKLSVGETELAKALEDKSKDPPEKIMCLFKCAL
EDSGFLQDGVVDKSKWPMPECVQDVVKITNCNDMVALKHCFD
>TcasOBP47
YEFNDPLFMELESSAYPHHRSRRDEDAVTEKCRPFRKKKLCCAEETFDELHDKDRDFKRE
CFKQVVGSKDGPREFDPFRCDKVDKHRRDMTCVSQCVGQKKDVLDKDGNVKEAEFGEF
VKETMAKESWFVSIQDKVVSTCLAEARNATANRDTSDTESCNPAGVKLMHCMFREIQLG
CPTEQIKDQKACARARDKIKRHNEFLPPPPQFLNDE
>TcasOBP48
EDAKEKKCDIPPTAPKKIEDVINQCQDEIKLAILTEALEALNINEHTKSRAKRDTFSDDEKRI
AGCLLQCVYRKMKAVNEKGFPTVEGLVALYSEGVTQKEYIIATLQAVNVCLNKAQKKHLT
KPQSLEAEHGKTCDIAYDVFDCVSERIGEYCGQTP

CSPs

>OcomCSP12
MKFAVFLCVISFVVIVTADKQKDEKYTTKYDNIEVDKILNNDRLLRNYVDCLVHGTHCTK
DGEVLKKVLSEALKTKCEKCSEKQKKEAVKVITFLLKNKRDWWNEIEAAYDPNHVYREV
YKKEIKDAGIQF
>OcomCSP11
MQTFQPGLILLIVFVSVIVVKAAITEKSSSTERPSISDEALEASLKDKRYLLRQLKCAIGEAA
CDSVGRRLKSLAPLVLQGSCSQCTPQEQRQIRKVLGYMQVNFPKEWNKILKQYSG
>OcomCSP10
MKTFVVCIFAAGFFLIHAEKYTTKYDNVDVDSIIKSDRLLLNYVNCLLDRGKCTPDGLEL
KKVLPDALLTDCSKCSDVQKKGSKKIIRYLIKNKPDWYGELEEKYDKDQTYKKKFEKEIS
T
>OcomCSP9
MGFARLIFLLAVVSYSFAQTYNTRYDNIDIDRILASKRLLDSYIHCLLDDNFKKCSPEGREF
KKYIPEAIHTNCGKCSDSQKRIVKKAAKHIIQNRPQDWDKIRKKYDPERKYQQSFNTFLN
SP
>OcomCSP8
MNKSVLFVFLLAVGYSAAAYSHKYDYINVMDVLHNARMLKRYVECLLDVPDTCTKDGD
YLKQVLPDAIRTNCGECDNKQKDTALRVIGYLMKHHSDWWQKIDARYSASGSQFLAVNS
AKIEEYKNTL
>OcomCSP7

MKPIFLVLLIVWYLYGIVICDKYTTKFDNINLDDVLKSERLLKNYFDCLMDRGKCTPDGK
EVRDNISDALETDCGKCSEKQRTGSMKVIKFLVQNKRPMFDELSAKYDPNGNYRTKHKE
EFAKEGIVL
>OcomCSP5
MKVTIAICVVVLMIVVASAAPADDKYTSKYDNVNLDEIIKNERLFRGYIDCLLGTKSCTKD
GEVLKKLLPDALKTKCERCTEAQKKGAKKMIRYLLQNKRDWWNELEAVYDPEHVYVKE
YENELKEEGIVL
>OcomCSP4
MVSFLLVLCLAGVSLATVTEKSKYTTKYDNINLEEIVHNERLLKNYVDCLLEKGKCTPDG
LELRKNMPDAIATDCSKCSDKQKEGSEYMMRFLIDNKPDYWNPLQEKYDPSGAYKQRYL
ESKKQEVKVEPITKA
>OcomCSP6
MEMCFILFILFTAISVRGDEEKYTSKYDNIDYRSILASERLLNNYFRCMMDEGPCTPDAQD
LKKVIPDILQTECSKCTDKQKAAGREIMKYLVDKKLDMWKKIVEKYDPQGIYKEKYRDE
WIKEGFPEL
>OcomCSP3
MNSFYLSFVVGILVVVAVFGAPKTFEENLEILKKVDVDAVIKNDRIMRNYIDCVLDKKTCT
PEGTAFKESWKEGLERGCGDCDEETNRIVKKLVKHVYVNKRNWYDELVEALDKDKKYS
KKYETYINDILKDTSI
>OcomCSP2
MNRCGLSVLLLGLLLVAAVLGAPKKTLDENLKVLRKIDINTVLNNERIIKSYVDCVTGKK
RCTSEGTALKESWKEGLDRGCDTCEEEEKKKIKKIAKHIYTHYRPLYDELVESLDKDGKY
REKYKSYIDELLNDSSI
>OcomCSP1
MVPLICVIVAGLSGLVVAAPVPEDNEPYYTTKYDHVDVEMILNNRRLVNYYTACILNKGP
CPPEGIEFKRILPDALKTNCRKCTEKQKVTTLHAIKRLMKEYKKIWKQLKAEWDPDDIYV
TKFLETYSKPTDANIFSNRFDGEESSESESTKASTNSTTESSNFSSSTPTTLGIYLPPVPTLSP
MVKPIANTIGQRLKSTVSFGGNIVGQVIKDIQRIGNTVVLTGAEIAGNIGNRVIQRGTQIAG
ALRAIANPAFRFNRT
>DarmCSP6
MKTFIFLVVLVSLYALSSCKPQEKYTTKYDNIDLDAIIRNDRLLRNYIDCVLGKRKCTKDG
QELKVHLPDALQSDCSKCSEAQKNGSRKIINHLLKNKRGWFNELQAKYDPAGTYLSKYS
DELRREGVIV
>DarmCSP9
MNSHCFQLPLIALLVLVALVSFVRNETTERPSISDEALEKTLSDKRYLQRQLKCAVGEAPCD
PVGRRLKSLAPLVLRGSCPQCTEQEKKQIKKVLAYVQVNFPKEWNKMLQTYAG
>DarmCSP8

MKICILVCCAFIGLVLADTPKYTTKYDNVDLDEIIKSDRLMKNYVNCLLEKGKCTPDGAE
LKKVLPDALHTECSKCSESQKKGSRKIMRHLIDNKPEWWTELENKYDKEGAYKKQYREE
LKKDGIKL
>DarmCSP7
MGSLTSFLVLMVTLTLVMMGAKNGANSRNVKRSAQSYTTKYDNIDIDQILASNRLLKNY
VNCLLDKGACTQEGKELKKYLPDAIATECSKCSQAQKKITGRVLQELLLNHRDDWELLTN
KYDPDGNFQRKYMQEDEDYSELEEA
>DarmCSP5
MQLIVSVLIGMALDLAGAKPTGKYYASKYDHIDVGTILNNRRMVNYYSACLLSQGACPP
EGVELKRILPEALQTNCARCSEKQAATALMAIKRLKKEYPKIWQELSGKWDPSATFVERF
ESTFEALHGPGRLTTPAGDKLDSPDSNTIDANITQTSPQDSDRGNQPATSTQIITTNPPFSSST
NPFASTKRPSPIPGLVPFNTFFTNPPIPIRPINIGATVKAIKQVEKMVADIALEKIRSILRPWRR
VKKAQNV
>DarmCSP4
MHCACVFLVLSALLVLIPAQYTSKYDNVDVDKILKNDRVLTNYIKCLMEEGPCTPEGREL
KKTLPDALASACSKCNDKQKSTTEKVIRHLQTRRAKDWDRLSKKYDPEGVYKQKYEAE
LKTDKTA
>DarmCSP3
MWKLILLGSLLASINPSLAEVTGKTQYTTKYDNVDINEVVHNERLLKNYVNCLLETGSCS
PDGLELKKNMPDAVETDCSKCSAKQREGSEVMMRFLIDNKPEYWNPLEEKYDPTGSYKK
RYLDAEKEEVAIQPAEKTP
>DarmCSP1
MKVVLLLVVVVGVAFGDEYTSKFDNVDLDQILSSDRLLRNYMNCLLDKGKCTPDGXEL
KKNLPDALENECSKCTPKQKEGAKKVIHYLIENKRDYWDQVAAKYDPEGKYYKKYEEQ
AKKENIKL
>DarmCSP2
MKFCVVLVLVLQISICLCQTYSSRFDNINIDEILSNKRVLNNYVRCVLDEGPCTAEGRELRT
HIPEALRTNCAKCTPSQQKFVRKGASFLIKNDPDQWKRITKKFDPEGKFASQFHQFLNA
>MaltCSP12
MRVLCQMVLTAMALLVLGSIYERSVSAAMFRTKREEKYTTKYDNFDVAGVLASQRLVRV
YLNCLLETGPCTPEGKELKKYIPDAIATECKKCSPVQKKQAGIVLYHILLNYREDFHKLTD
KYDPEGKARKIYGIDQDDDDDDNLDQ
>MaltCSP11
MNQYLLVLFVALIGVVASQKYTTKYDNVDLDQIIKSDRLLKNYIDCVLDRGNCTPDGQEL
KKNLPDALLTDCSKCSETQKNGSTKILRHLVKNKRPWFDELAAKFDPDSSYRKRHEEEFA
KEGIV
>MaltCSP10

MLYLSSLICLVCISSVIGQKYSNKYDHIDIDRILSSKRVLNNYIKCILDQGPCTPDGREFRDHI
PEAITTNCVKCTDAQVKIIRKTSAHIMNNRPEDWEKIKNKFDPEEKYKDSFMKFIDG

>MaltCSP9

MKVVLVFAVLCAFAYAEYTTKYDNIDLDEILRNDRLLKSYFQCLEGTKSCTKDGQELKNIL
PDALKTKCSQCNETQKQGAEKVIRYLIDNKPEWWKKLEAIYDPSG

>MaltCSP7

MNELVYLAIFLSAAVVGEEMYSSKFDNIDYEEIIKSDRLLKNYANCLLDKGGCTAEATELK
RILPDALKTDCAKCSERQKKGAKKIIQFLVNNKPDLWEQLMAKYDPDGEFKEKYEGDWL
NED

>MaltCSP6

MVCLLPVLVTTGTLLGLIDGANAQYYASKYDHIDVDAILNNRRIVNYYAACLLTKGPCPP
EGIEFKRILPEALRTNCQRCTEKQKTVTLRAIRRLKKEYPKVWAQLEKQWDPDGSYISKFE
STFGAKSTESIPESSVQIVNRYASPEDNKTIENALSQIFTTPLPTSTSTSTSTTSTTTATTSTTS
TTAKTTTTSKATTVSTKAAPVTKTTTHRVSTINIIKNINTISSSTKPTKRPRPSIGQSIQATVSI
GTNIVGDLVRGLEAIGNRVVETGAEIAGVVLKNIARPL

>MaltCSP5

MKLVVFLFFVALCGITYGRPDDKYTTKYDNIDLDEILKNDRLLRAYVDCLLGTKKCTNDG
EELKKVLPEAIDNECAKCSEKQKDGAKKVVHYLIDNKSDWWKELEVVYDPTGKYRKKY
EEQAKKEGLKI

>MaltCSP4

MTVRYAVVTLVLSLVIAVEVQGQRGLTGNNYVERQLLCALDKAPCDNLGRQIKDALPEIIG
NNCKSCDQKQMANAKRIARFVQNKYPDVWNALITKYGAE

>MaltCSP3

MRAYVLAVFVVLVGVVASQKYTTKYDNIDLDEIIKSDRLLKNYLDCVMERGNCTPDGQE
LKKNIPDALVTDCSKCSDRQRDGTRKILRHLVMNKREWFDEVAGKYDPEGVYRKKNREE
FAKEGVEI

>MaltCSP2

MLLIAVTMKVSLLFVVVLGLVVSIRADEKYTTKYDNVDLDEIIKSDRLLKNYVNCLLDKG
NCSPDGTELKKVLPDALLTDCSKCSETQKNGSKKIIRHLIDNKADWYKELEAKYDKEGVY
KKKYEEELGLKKEKREEAKEEKKEKEEKKEKEEKKEKEVKDEKKD

>MaltCSP8

MLKMIIYFLVAIGLITYATSTVTERTKYTTKYDNIDLDEIIHNERLLKNYVDCLLEKGRCTP
DGLELKKNMPDAIETDCSKCSEKQKEGSEIIIRYLIDNKPEYWNPLQDKYDPTGSYKKRYL
DAKKTEVNVEPIVKS

>MaltCSP1

MHFKCQIALLLCIAVMTIVTEAFPQADTERSEVSDEALESTLKDKRYLMRQLKCALGEAP
CDPVGRRLKSLAPLVLQGSCSQCSPKELNQIRKVLSYMQINFPKEWNRVLKQYSR

>AmalCSP8
MRFLVQIALFCSFIIFALSIPQNRPSVSEEALEKTLNDRRYLMRQLKCALGEAPCDSVGRRL
KSLAPLVLRGTCPQCSPSEMRQIQKVLAHVQKNYPKEWGRILQQYSSG
>AmalCSP5
MGNRNWIFLSIFLTVLVTTLAQRHIIGRLQSVNLEQVLNNDRLFNNFYKCLADEGKCSND
AKSIKNLLPEVVKTKCGQCTADERSQIKKSLNLVRKNKPEQFNSLAAKYDPEGVWKELVR
>AmalCSP4
MSRIATVCLLAAFVVIVSSVPQAKYTIKYDNVDLDQILSNQRLLENYYNCLMDKGKCTPD
GQELKKNLPDALTTKCSKCSEKQKEGTQRVVEYLIKNKNDWWKNLERKYDPSGNYRRD
YGPELAQRGITV
>AmalCSP1
MKASIIFLFTVFLSCIAYAMSASKYTTKYDNIDLDEILKSDRLLGNYVKCLMEEGKCTPDG
AELKKVLPDALKHKCEGCSDKQKQGSKKVVNFLIKNKQDWWKKLEQKYDPNGQYVKD
YKDELEKEGIKL
>HparCSP17
MCKVFILVIIALVVAVTSEKTDDDKEQYTSQYDGIDIPALLANRRLVLGYCKCLLGKGACS
PDGAELKRVLPEALETNCVKCSEKHRNGARLVLNHLIDHEAKCWKELEEKFDPEGTYVK
KYKEEYKLKD
>BhorCSP2
MKAYLSFVALLVAVACARADDDKYTTKYDNVDLDEIVKSDRLLKNYVNCLLEKGNCTPD
GTELKKVLPDALLTDCTKCSDTQKKGSKKIIRHLIDNKADWYKELEAKYDKDGVYKKKY
EEELELKKE
>LoryCSP10
MQIVVLFTFLSGILFSFCLAKPAKSYTNKYDNVNIDEILNSDRLLKNYMNCLLDKGRCTPD
GAELKKNLPEALEDDCSKCSEYQRNRGAKALKYLIEHKRSYYDQLERKYDPERKYRKRY
EASFKREGINL
>LoryCSP9
MNSATLTCILVILILNICYGADEKYTNKYDNINIDEILQSDRLLNNYMNCLLDKGRCTPDG
AELKKNLPDALEDECSKCSEYQKDRGRKALKFLIEKRRGYFDQLEAKYDPDGKYRKRYE
ENMKKEGITL
>LoryCSP8
MKFRLLFFVAFIGIVVAANSKYTTKYDNVDLDEIIKSDRLMKNYVNCLLEKGKCTPDGAE
LKKVLPDALHTECSKCSDTQKRGSRKIMRHLIDNKPEWWKDLEDKYDKEGAYKKQYRE
ELKKEGIKL
>LoryCSP6
MKYLVLVLIATAVVVANARPDQYTSKYDNVDIDEILKSDRLLRNYVNCLLGTGKCTPDGA
ELKKNLPDALETGCTKCTEKQREGSKKVIHYLIEKKRPWWDELAVKYDPTGKYTKKYEE

EAKQQNN
>LoryCSP3
MFWIKAIFYIVVVFEIFYDKRSFCALLSRSKRAEMYTTKYDNIDVDSILASTRLLKNYVNC
LLDQGPCSPEGKELKKYLPDAIATECTKCSEAQKKIAGKVFSHMLLKHRDDFEKITAKYD
PERKIYNKYLLDDKDYADLEEA
>PyasCSP4
MHKFFLLFLAVQIFVCFAQQYTSKYDNLDINKILSNDRVLTNYVKCILDAGPCTSEGRELK
THIPEALSTSCAKCTESQKKFVRMGANYLIKNKPNEWGKIAKKYDPEGEHSAQFNEFLKG
>CforCSP1
MRLCFVLFVACLGIALAKPQEAKYTTKYDNVDLDEIIKSDRLMKNYVNCLLEKGNCTPD
GAELKKHLPDALHTDCSKCSETQKNGSKKIMRHLIDNKPDWWKQLEDKYDKEGTYKKK
YREELSKEGIQLQ
>CforCSP5
MNSVIVLFATVCLALVCGQKYTEKYDNVDLDAIIRNDRLLQNYVNCLLDKGKCTTDGAE
LKRILPDALQTACSKCSEKQKNGSKKIVNHLIDNKPTVWSELEKKYDPQGVYLKKYKAE
AKTQGVHL
>CforCSP6
MISVVPLMIFLSLSKFSAGEVTEKSKYTTKYDNIDINEIIHNERLLKRYVYCLLETGSCTPD
GLELKKNMPDAIATNCSKCSEKQKEGSETIIRYLIDNKPEYWSQLQDKYDPSGSYKKRYLE
SKKAEVIVEPIG
>TcasCSP14
MKTFVILFFGVFFIIFSDFVNGKTLHRSTRDDKYTTRYDNVDVDRILHSKRLLLNYINCLLE
KGPCSPEGRELKKILPDALVTNCSKCSEVQKKQAGKILTFVLLNYRNEWNQLVAKYDPDG
IYRKQYEIDDDYDYSELDSAKK
>TcasCSP13
MIPLIAIAGILAVSAAPAEFYESRYDHLDVESILNNRRMVNYYAACLLSKGPCPPQGVDLK
RVLPEALQTNCAKCTEKQRTAAYRSIKRLKKEYPKIWEQLRAVWDPDDVFIRKFETSFESG
KPSGVISTNTSPPSPILSNRFGENEEADAASNVISSTPLPPTTSTTTRTTLTTKFTTKPSTKPT
NKPVVVTKPPQAPPFATVGANLQATVTFMAALKNIVIQLCQHKLEVINSILSGR
>TcasCSP8
MIFKIHFLVFGALLTYVSSVEYLILREIDTILKNDQMTRNYLDCVLDKGKCTKEAEKLKKG
ITETMKNGCVKCEQKQKEDVHKVFQHLMIHRPNWWHELETKFNPHHEIKLQHLHQSKF
NPHEEVKLQHLHQFPHHDFLEREGFIR
>TcasCSP19
MKFFIAFLMLLGAVWCEQYTTKYDNINVDEILASERLLKNYFNCIMDRGACTPDADELKR
VLPDALKSDCAKCSEKQKEMTKKVIHFLSHNKQQMWKELTAKYDPDGIYFEKYKDKFDS
>TcasCSP18

MRFFVIFFVACVSVALARPEDQYTIKYDNVNLKEILQSDRLTENYVNCLLEKKPCTPDGEE
LKRVLPDALKTSCAKCTDKQKQGAKTVIQHLYKNKQDWWKQLEAKYDPEHTYVKAHE
DELKAL
>TcasCSP16
MPLVKSLVVVVLLIGVVYQVQGQLGLAGNNYIEKQLLCALDKAPCDALGNQIKGALPEII
GKNCERCDSRQVANARRIARYVQTKHPDVWNALVKKYSV
>TcasCSP12
MKLISAVILCAFLVAVSAAENKYTNKYDNVDVDKILNNDRVLTNYIKCLMDEGPCTSEGR
ELKKTLPDALSSGCTKCNQKQKETAEKVIRHLTQKRARDWERLSKKYDPQGQYKKRYEE
HVATSRAA
>TcasCSP11
MYSYLIPLYLFLFVHYGWSEDTTHKYTTKYDNIDLENVVKNERLLKSYVDCLLEKGRCSP
DGLELKKNMPDAIETDCSKCSEKQKEGSDFIMRYLIDNKPDYWKALEAKYDPDGTYKKR
YFESQKDEVSKVEA
>TcasCSP10
MKLFVINFILMSLVYMSFGASVPYETVDIDKLLADDKMVTEYMACLRGEGPCNPAEKDL
EEHIPLVLGNYCADCNDKQKNFVIKLATFVIKNRFDEWRQVQKRFDPDLSHADDFNKFIL
GS
>TcasCSP7
MKTFVLVAFAAVLGLALARPQEKYTTKYDNIDLEEILKSDRLLKNYFNCLMERGTCSPDG
EELKKALPDALHSGCSKCTEKQKEGSRKIIHYLIDNKRDWWNELEAKYDKDGVYRQKY
KDVIEKEGIKL
>TcasCSP1
MKTLVPLLFFVIAIASSLAENSKYTTKYDNVDLDEIIKSDRLLKNYVNCLLEKGKCTPDGA
ELKRHLPDALHTECSKCSETQKNGSKKIMRHLIDHKRDWWNELEEKYDKEGEYRKKYE
AEIKGKKD
>TcasCSP9
MKTLVLVLFVAVLSVVFAADKYTTKYDNIDLNQILKSDRLLKNYVNCLLDRGKCSPDGQE
LKNNLADALQTSCSKCSQRQKDGSRTIIRYLIKNKRDWWNELEAKYDPTGIYKNKYADEL
KAEGIVL
>TcasCSP6
MFLAIVLVVCACTNVLSEEYTNQYNDELDAALKSERLMKSYFECLLGTGKCTPSGEELKK
DIPDALKNECAKCNDKHKEGIRKVIHYLVKQKPEWWEQLQKKFDPQGIYKKRYQNYLD
KEGLKA
>TcasCSP2
MFATSALFAFICIQGLVSAEEYLVPQNIDLDEILKNDRLTRNYIDCILGKGKCTPEGEELKRD
IPEALQNECAKCNEKHKEGVRKVLHHLIKNKPNWWQELEAKFDPKGEYKQKYNKLLEK

EGLQA

>TcasCSP5

MTAIVFLLALACLKTYVSSQEYLVPQNIDVDEILKNDRLTRNYLDCVLGKGKCTPEGEEL
KKDIPEALQNGCAKCNEKHKEGVRKVIHHLIENKPNWWQELESKFDPQGEYKKKYDELL
KKEGLAN

>TcasCSP4

MFKVLFVVFACVQAYVYAEEYTVPQNIDIDEILKNDRLTKNYLDCILEKGKCTPEGEELKK
DIPDALQNECAKCNEKHKEGVRKVIRHLIKNKPSWWQELQEKYDPKGEYKSRYNHFLEE
EGLN

>TcasCSP3

MLFTVFLVLTCAHVVFLEEYVIPDNIDIDDILSNERLLKNYVNCLLDKGRCTPEGKKLKSTI
PEALSTDCAKCNEKVKANVRKVLHHLIDNKPDMWKQLEAKYDPSGEYRSKYKDELEKN
GIHV

>TcasCSP20

MKIIILAVLIATAVAATYDVYPTKYDNVDIDAILHNKRLFDNYLQCLLKKGKCNEEAAILRD
VIPDALITGCRKCNDHQKVSVEKVIRFLIKERNSDWQQLISVYDPKGEYQTQYAHYLEKI

ORs

>CbowOR1

MIGFLKRQTLVDKLKIHVTFFFILILDVLILVKISTTHNKTLEDIMTSYEAVGSYLQMTTKIL
TLIIYNGDLKQILAMTNQFWKYDKFGPVISNKQQKYPRMMPSFITAYFFFCICTLTVLMLK
PVLFHELPRSCCIPEGEVWFYVVSAIQNETLFYCTFTVFAFDAMFALLYTEAAMQFCLLNE
AFSRMKNHGDLKECVDYHVFLYNFVKKLNDVYWMFLLVQSFDCLSETCFQLLTMVHTQ
ENLTLRVKAVLYAIALYMQLSFFCFPVGFLQDESQASSTAISACPWYLKDAKFKRSVFIVMI
RAQKKISVRAGGFFEMDRQAFIYLCKSSFSVYTLLKSIN

>CbowOR3

MATITNSLFVILEIGICIIKFLPFKNDPKKIRKTLFALNQDMFNRATESQRRFIEETEAACRNI
FAIFMTFCLLSLFSWPIKVLFYEQRRFPIDVWLPFDPFENVSIYLGVFAYLFIATGNAPIGNA
AIDTLIAGLIIHAACQFRILKDNLRCLSQRADEKLNGLPQELKEMKRNEIVYRNIRECILHY
DAIYDFVKEVEKTFSVVIFSQFAVSILVICISCFQLSIAEPLTITFFAMVIYVVSLLLEIFLYCYY
GTVLYEESNTLIAAIFDSEWYDLDEKSKKALFILMERAKRPMMLTTGKLLSVSLETWTMII
RRSYSLLAVLKNHQ

>CbowOR4

MLFYVCGFAVVICEYMMFKESIKDIGKFVSHIGMVLTHLAGIVKFCLLTIGHGKILKLMHV
LQNKDYQYCSLEDSKPGEVLRKGQTVNNVIAYSTFVMYTLVGITGHISSVRNLNEQIKGD
NFEGTNKTCYDFLPYMFYIPIPSETKWQCEMVFNLMDIGFALHAFVIAAHDGIFAGLLICL
KSQLLIVCDVYKTIRQRSLKNMHLPENYTITNDMENPALENEMYRLLVHSMEHLKILLWV
RDELEYIFTMVVLTQTVASLFILASNFYVASTILTASLEFFAKLEYTFCIFFQLSLICWFGDDI

TRASDLIKLSLYESDWLSSSPRFKHAMVLTMIRMQRPVFLSIGKFTPITLSTLVAVCRGSFSY
FALFKSIQK
>CbowOR5
MIKMIQIRFHTLGFLACMKIFMLSFGQLLSKTLPLKCYSAKWIPFHVMWFYQSFLTSVCII
MPIIAMDLLLMTFISLTHIQFKMLNLEIDRVFRRSERAKKSEIARLVDHHNFLIDFSNRINNT
FSTMLLAYIFVFVISMCVEMYKSSANPSFSVFMNAVTYLSAAVFGLIFLFCIPGQNLTDEAN
NIPNAVYFTDWYRDSKQSTSVLMMISNGQRDISIKAGEVIKINLATSLSTIKTLLSYFMFLR
TVVLDE
>CbowOR6
MYTIKKSQPFYSSLRTLRFFLVYREFVQKSTFMLLSFFSSFMSFSAFLVFVCGCILHAVMSIR
ENIGGDISEDLSVSIGGLAMMVNVAMFKYHQDKWSNFFKDVTNFEKFGKPTDFDATKDR
ANLLSTLYMIYCITGTIVYSCVGVIESSCDELSEETKQKVICGTLAPIWLPFEDVSLTVRNTI
LLVQYVLANYIITPSAVICFLPFETTELLICHINFLKDKLLKVFGNEDGMIRNDKLRFCVAYH
THILGMADQLKYVVKFSVGHMSLVCALVFGCIGNQIFRAKPVGAVIFLLGYMVSLFLLCY
AGQRIMNESLSIVDVIYNSKWYKGNTQIKKNVRFMMARCQIPVTLDAWPFGIFSFPLFMM
IVKTSYSYLTLLRQST
>CbowOR7
MISPTSYLIKLIIFKSKSVHVLEMLSFLELTEFNNYPKGLSGIVERTVKFSRYLGYAYQFMCC
LVITLYSTIPLFTKADLPIRFSHDVGKLKPAVYIFQVIGLSSAASNNSCLDVLAMSLMGICSA
QIDILNKKLITLGKNEDEDETDGSNNSYLRLKKCAKHHVEIIRFQKALERVFSSIFLAQFATS
VMVICNIGFQLVHVQPASVQFALMLFYFIAMNTQLVMYCWYGNEIIVKVYHCLSILSSAIR
DACYKFEWFDSNMETKKLLLIIMEHSKKHLYLTAGKISVLSLESFTSVMRTSYSYFALLQT
LYRNNQD
>CbowOR9
MLTFSLIHITSLVQVSETLSFNLTQLAYLCKLLNFQIHSKRLLELEDFLRKTTLTNVTVEEEA
IIRNTMKGSRRLATVYRSLCVIIVFLYALFPLIDENSGDEKKLPLPMWFPFDTNNHFGKVWF
FEIFSIAIGAWTNSNLDVICVTMITLTTCQFNIMNSRLSNLRKSTDDVEEEDTVQKALKECV
IHYNDIISFQILVETTFSLSIFGQFVFSVLVICMTGFQMLVISFKSVQFVLLLSYLLGQTCQIV
MYCWYGQSILDSSEAINDACYSSEWFNCSKETQKMFLIIMERSKRPVKMRAGKFFFLNLD
TLMSILKSSYSYFAVLRHIYSSKFT
>CbowOR10
MMVKQTKNEAKRFFRYIGMLFTPTQALGCYVLLRIKQHGIEKVREELLDEQFHYKSCGSF
RPGKIFNDAKSFCDKFVVITIILYSLVVASAHISAYVTLNLAFEGEYFPANITCYDFMPNYFV
IPFPTPTKSSCKNALTYMDVSLNVYATLLASYDTTFCSVLICFKTKLQILSGAMRSMRERVT
TEMNLPLNSSLELDDPEVEAKLYEEIKQCARHLESLLSVCKQIEDIFKYGTLMQIVNALVII
SSCMFVLSITPQSDPDFFVMIHYIIALFVQLFTVCYFGNEITEVADELNNSLYQSNWLSCSK
RHKQCMIIMMSRMQKKIHVMIGKFSPLTMNMFVAVVKGALSYCAVFRAVDNAEI

>CbowOR11

MGAVKVLFFYFRGDKLIKIMATLESTDLHYEKCQKRKFFPGSISTNYKKVGIKYTLLFFML

AHATLISSYIPPTIAAIQSELDNPGKSLPDRLPYYSWMPFKFDTSTTYLIALGYQAIPMFSYA

YSIVGMDTLFMNIMNCIGMNLEIIQGAFLSLRERAADKIAGPLMTQDGLHNSHELKTALN

REMKKVCRHLQIIYRLCEDLENVHTFLTLAQTVATLFILCSCLYLVSTTPASSKQFLSEIVYM

VAMGFQLILYCWFGNEVTLKADMIPFFIWQSDWISADREFKHAMIFTMIRAKRQLHLTAG

KFAPLTLTTFIAIIKASYSFYAVIKNTST

>CbowOR13

MDYGYPKNFFEANDVVKRISGIMLLQGKEDNIFFKWYQIIYIIFVYSSTVVFTVGQYIMTK

NSVNKISNLVSSLGVLLTTHVGHFKFWLLLSKKKELENLKNDIEGENYQYATIGNSNPGLL

LTNEKKFCTVCTYVYLAGCYLIGIFGNITTVVRLNGALTGNNTFESINMTCNDFASFTFYIP

FFTANKWQCIISSTVMYSGMAFESGIHAACDLLFFAMIHCLKIQLRIIADVFRTIRRRSLLKL

NVPEDYTVLHDEENSALEEELYRQLSHSTEHLNILLRVTQEIEHLFTYVLLAQTLSSLLIVA

SFLYLTSTISINDADFFLQMQFLLVILIQLALLCWSGNEITEGFQLIKTALYESDWLSCSHRF

KRSMILTMIRLQRPVLLTLGGFSPLTLATLVGVCQGSFSYFTLFKSFQ

>CbowOR14

MATEFQKAFETEKWILSLFGFYPQWNPEPLWKHARRVFCIAVTLTYIISMCTGPFLENSVMI

LCTVMGLSKMVQLLTSKRQFREIEHYISNMKPSIIRRSSLIGAFRVSVVTLVGFLGIMPLSM

KNQRMLPYKSWLPYSVEGASPYYSTFIFEVISIVMAAFTNSTIDMMYYCLVDICCAELDVL

KLNIIEIDMSDHVDIVEDELKKIVIHHHKIIRLVGIIQEIFSSVVFVQCMASVLVICFLGFQLIY

VDKLPSVKALIELSFIACMLIQIFCYCWFGHNITMKSSEVGDICYHTKWFESDLMIRKIILII

MERCKKPVELRAKIFTLNLQTLLAILRSSYSYMAILRTLYTDE

>CbowOR15

MKEVHFKNTVLNFYEFYNTDFKLLKFFGIWIPDSSNSKFHKIYFIVINFVFCAIFNLAQVSN

LLHEINNLKNLAACGYVVAIACMANVRSYYFLKNREEFLYLIRSLNDSHFQPESEDQICSA

KKSLRFYSKVKMIVSILCTITVFISMSTPVFYKKNELNLPFASWYPFDVSSYPIYQIAYVHQ

CISVIYVTSINTYVDIIMAGFNTFIGIQCDLLCSRLYNISKDHSSEENETTLLDCIRHHKLIVR

FANNTEILFNRIYLGQFIACTSALCMALFLLTLHQESRFESSFLVFYLTAIFSLLFIPCWFSSE

MQGKSENIPEAAYSCNWVTASKLFKKDLIFFILRAQKPLKFYAVGFFQISVETFVLIVRSSFS

YYTVLNNMIMKEG

>CbowOR17

MNIEHIKEIEVLENSLKFLRIFFLFPRKSEINDPKKNVYWKFILLSLSTAYFSIGAAIHLVVNV

RSGAFVNVDKDVGTIISYYGALYFISRYLGNIKYIIILYKQFSDFKTYGLPNNFEKTNKLLN

KFSKIYFVYHMFIVTGMTTSTLLTIGTCEEENLENNINDICGLVGPTWLPFEFDYFPLKQIV

YGYQVYCSFVIFQLAGHLSYTLMESVEHLIIRFEHVGHTFVEALNEKNSYTRREKFYVAIQ

YHNDVIQMGKLLNSCFAPSLIVHISLTGPVLGVAGYRFLTEIPLDSTCLFFGWMFSTFIVCR

GGQRLSEASLAVGDVIYRVNWYNLETDLQRDLKMVMLRSRKPVYLRAGPFGPMTYSTIV

TILKTCYSYITLLKQTM

>CbowOR19

MFRVGDAIAFQSTINYMTFFKVFTVQTDTRYVGTILRFWSFCLICLFNTFHLVYVKIENIDV

DTSEDLVVILGGMGILLICIFSASSSRRWTSFLHNLIDFEKYGKPDGIEHAIERGNYWAHFF

GLYYIIGTVIYGIVTYMEAPSCHRLNNEKNLHLICDTFVAIWLPFDIPLKSIRLSVFFIQFVLI

TCNVNPAAMACFLTWECTEILRCHLRHLKKHFHKMVKEGDVRKRPDGIGYWIRYHNHIL

SLSYELKSLFKISVGHTSLISGLVIGCTENQILKSKPLGASLFFLGWMAAMMLLCHAGEIL

MEETLSVADTLRDSQWYLADLETRRDMVFIMLRSQKPVHLEAMPLGVMNYALFVMILRT

SYSFMTLLNQSS

>CbowOR20

MRDVEEAEKSLMVTGWFPFDTREYFAVAYLFQLQIAIIGGLFLVALDSLIISLIMVAPLRLKV

LANYFRHFGDKKSMNSLLSLKNLISEHQGIIRYVEDLNASLKWLFLADFVVKSYNISIVLS

NAVSIYEFRNIVIIIYYVLQTRGRNKSELAFSALFLCFLLSQLYCFYFHANEILLESTNLAENI

FKSKWYEQNSQIKRSLIIVMIRSQKPLQITIGDLHAIENILFVKIVKAAYTFLLFQYLGL

>CbowOR21

MKTQFQKAFQKEKWILTSLGSYPQWEPEALWQHCRRFFCMTVSITYITLMCTGPLLENSI

MILCTVMGVVKEMQLLFSKQQFREIEEYIGSMKPCKIPISRLLGSFRNSVVTVIIFLGLMPY

TKRSLRMLPYKSWLPYDVSRAPVYYYVTFVAEVLTIIMAAFTNTTIDVLYYCLIDICCAELD

LLKMELMEIDMSESYEVVQNKLKNVVIYHQRIIRLVEVIQDVFSSVVFIQCMTSVLVICFLG

FQIVYVDEIPSGKATIEVSFIGCMLLQIFSYCWFGQSIMMKSLEVADVCYNSNWYDADLRI

RKMIFIIMERCKQPLELRAKIITINLQTLLAILRSSYSYMAILRTLYSD

>CbowOR22

MSSFSESPENEEQPFSATTKMMRLLCVYPLGFEKWQMVRFYVNVVVVKLFSFFFCCVLCL

LHLVMTKIDGAHKADLSEDVSMIMAGTGMLATNLLFAYKVKKWNSLMGKVADSPEMRN

IQNFEAIKKRCNRLARLFTMYCVIGAGIYLLSGYYESLVCIRKNEENGSNEICRTLMPVWL

PFRLSSAAELTLFALQAFAGINLSLPGANMPFLVWEITEMISLRISHLKKISESIVVEKNIKSR

QERLKHWVMSHQQIIECISLLNEQVRLCFGHISTIAALVLGCLANQAINSVHLGAMAELGG

WMVGLFLLCSSGQKITDITESVAEAIYAMEWYSTDVQTMRDIRFILMRSQKPLVLQAGPL

GALNYPLYMMMVKASYTYLTLLANTI

>CbowOR24

MMTEKDEKMPKKLEIVCMPTSIRIFRFYCAFPPSDKLLNPGKMFYIRFALIALFSSVVLVGS

TMHLIKNVKDRTYNHIELDFTYIVSNLAGYGLLCSYFTKVNAAVQLYLILSDFEEFGKPINF

DNTNKKFNKYAKYQYCYLESITVCILLGSNMFRGAQCRKDNAELDQHEVCGLFAYTWLP

FDIDFFPVKQIYLACQLFGIHYVYMMAGLASWMVLESVEHIATRLRHVSHFFNEALKEAE

QRKRREKFNFAVRYHVAVLDLESKLNQTFSVFMFTHMVMSGIIMGYGVYSYMKGKNVST

ILIATGWLIGLLMDCYSGQRIQDESTLVGTALYDADWSDADDELKRDIRFVMMRCQKPMI

VQATSFGIMDHPLFLAVLKATYSYVTLLSQSDL

>CbowOR26

MKHKKLYNFYTIFCTSVWVTFILSQLVYMFSSFSNMDEMTSIIYVAGTVTIDLVKMLAIYS

NMDRIKPLLNDLNNPLFQPKCKEHVELALAVKKFHSRLFYFCLYFGVQTYICFSAIPFILEE

NVTLTQGWFPIDWTYSPNYEIVYAFQNIVILWNTLIFLNLDTFTSGLLMQVGLQCDFLSTTF

NKIDAFHVSSGVLIENGEQMALSLKDNHEFFNRVMTENLIVCVKHYRKIRRLATEIEDIHHI

SVFILFLGGAIIICADLFQLSIVQTGGVEFVLFVSFLMCMLMEQFMYCWFGNEIIFKSDNIFA

ASYNTPWLDCDLKFRKILLNFMTQSIDPIGLKAGGLFTMSIKAFVSVLKSAYSYFTLLQRIQ

EKECSELN

>CbowOR27

MIPSEDNSLSSMWLTKLILKSIFMWPDDYSDTKRKTFYKISMTICLFIQSGLVLNLTQNYHD

WEKNLAVVSSMSTIFQTVFKMTALYQNSDHIKFVLMCMCRKFWPHNLDNENSEYIFKQS

HSRRMRLMVFLLASGFLFSLGSVISPMFTRDTPFKSDYPFNWRRSPFYELIYLIQVAANGY

LINMTVIGFDFLFMDICAALTNQYVLLGSCFERLGTENMQDFYARIRERGCQKWPPKVGG

ARRFLGICVQHHQLLTQITKVVGHIFNVVAFLQLCSSVVAICVSGFIATKDDVTTSQIATMG

SYLIGHLIQLYIYCSVGNELLFQSSTLTNHIFGSNWYNLDSTTTKKDIIFIMKKAQIPAKLNA

FKVFPLNFATFIAVVRLSFSSYTLLTSITNK

>CbowOR28

MYASNLDWCLKLNFLLGVHPAKQKSFTQTLQYLFIIFGSCAIMILTVLLLYYKEDAVSMKD

ITDVSTNFTMFPHGMIKLTTLYMKRAEILDLLRRTKEHFWQIKDDREDVKRSYKLAKLLK

NLFFNSVVLFIISAIVKPIIIGGNTLTYKCHKPELIPRWLFLIFQDAMCVAILFTLSCTDVLILT

LLILTQIQFRMLNEKIQTIHDNEVDFHDRLKECVDHQNFLMDFVDRFSKVFSKTILLFIGNII

LSLCMCMYIITTESANINVQMEALFHLIAGLNEICLCYSIPAQTLMNEADEVGKNAYFSKW

YEHPKDAKLILQIMIRDQKRMVITAGDFVRIDMEMFLTACKTIVSYCMFLRTMSMVDQ

>CbowOR29

MTCNEFASFTFYIPFFTATKWQCIISSTAMYSGMLFEAGIHAVHDGIFFGLIYCSKIQLQIIGD

VFRTIRQRSLSKLNIPEDYSVLHDEENPALEEELYRQLSHSTDHLKILLRVQDEIKKLFTYVL

LAQIISMLFTLASFLYLVSTISINDPGLFLQFFWFMTILLQLTLFCWSGNGITEGFDSINTALY

ESEWLSCSRRFKKSMILTMTRLQRPVLLSLGGFSPLTLATLVGVCQGSFSYFTLFQSYQ

>CbowOR31

MMAFGYPKNFFHMNEATVRFLGVWLPSKKHHILIRLLHPFYFIFVYSTLIYFVIGQYMKVE

MKNVTTIISSLAVLLTTHAGHVKGSLVVFGGRRIQEIKDILQDVNYQYYPVGEVNPGSTFQ

KEKTFYTMLSYALLVGFMMPGASGTVTTASRLMIEMKGNNTFESIDKNCNDFITYTFYVPI

FIETKWECIISSSLMYSGMTMYEGIAHAAHDGLLAGLLICLKTQLLILGDIFRTLRQRVLSR

LNIPEDYSVIHDEENPALEEEMYRQLCLCTEHLKILLTARDKIEKTFTYMLLMQTIASFPVF

ASSLYAASQTPLSSTDFYTNIDFFGCVLVQLAMFCWFGNGITEAGEAIRSALYEGDWYSCS

PRFKKSMILTMTRMQRPVYLSIGRFSPLTITTLVSVCQGSFSYFTLFKSL

>CbowOR32

MEEDQKIYHFKTLKYILILMGQWKFRNRSRFFIKLYELIISRLVVAYLILSAHMFLINVAFAW
DCKARVMEMLTAYMQNLNVIIVTLIMRSQRMRNVLRYVQHYENIKLKDENIAVRDIYMQ
YVSINHKICRLLIGVVVFTALFYFATGMRNSFLISSTEECPMMKGIMFQLWYPMDTKKYFY
LVVLNDNLIIINIVITIIHAKGLIIANMIFAISQMKILQYELTLVGQIEQETDEDVELRVKKCIM
IHQEIARMMDLFVSASKDIILMQYFITSSELALYLLQMMLADSFAVFGRLLMNFIYLFVEVF
LLFWCANNVLVESMAISDVIYNESNWITLSNGAKKDMLMMLSRAQVPMAFKATFVGNIS
LETFTKLLKLCYSVVAFLSNVVRE
>CbowOR34
MTFFFKIKGLSPPKTTAVKIIYLSLALPHILMCTFVLILSEWMAFALSQQTFKERMFNMSVA
TLDTILVFRTTVWAFNKAKLDEIRAIITRKSFNFRCFDLLKVGCEQVLTVGRTKEVEKERGL
SCKEIKQLWQKAKFVTEKKCNEMDVFRKELMLNTRLLCCFIHLAITIISVLTYTLSFVNDF
TNDTYEAYNPILNRTSLYRKFQYPLYLPFDTSFDGYYWLAYFYNCYAHLGNIITFLPIETTLT
CSLIHLISQTAVLKEAFKYVDENNFGYQSFGEASIIKEIRIVKCINEIQEIYRAVELLENLCNV
QLMVQYGFATFLLCSICYVIPLVENTMEGICCLIFFAASLGQIFTFSYCCHTLALELQAIGVS
VYNLDWTNYPSKLKRTLNISILRTQKPANLTAGKIIVIDLLFFIQVVQKSYSFYTLITKTN
>CbowOR35
MSHNEIVCMTTSIKILRTYGVFPSQSRELHPGMVFYIRFLVLAVVTSLTLVGSTLHLIKTIQN
NEYNYTEMDLVYIVSFVTAYALIGSFVMKVKASGEMFVFLSNFEEFGKPINFDKNNKLFN
RYSKYHYVVYLESLILIILFSSNIFKSKTCRLENELYNLKEVCGLFTYTWMPFNIDYTPVREIY
LTIQLLGNHHIYMLAGLVAWQVFETIQHIIIRIRHVKHLFVEALQEGDVKVRRKKFNFAVRY
HNAVLSAFFSLEDKLNAAFGIFMVTHMVLTAAVIGTGIYCLFRRRSLSSFLVCMGWFWGLF
MDCFSGQRLQDESLELAVALYDSPWYEMDKEFIKDIMFVLSRCQIPMKLRAYAFGVIDRA
MFLAVMKGTYSYITLLRSQ
>CbowOR36
MYFFYVSFLYGTGVIFFVCEFMIFNETIGKISKFVSHIGMLFTHVVGILKMSILIFGRYRILKI
MNVLQNEKYHYAPLEDSQPGLLVVKEKFVSSGISILVFVLYTFVGVSAHISSLITINEEIKGD
SFEGTNKTCHDYMPYFFYIPFPTETKGQCGIAFAFMDVGLGIFAWVIACHDGVFVGLLNCL
KTQLLIVCNIFTTIRARSLKAVNLPKNYKILHDEYNPALEKELYRQLSHCTEHLKLLLVVRD
DLEIMFTFVTLSQTLASLLIFASCLYVASTVPMTSPEFFAQMEYFLCVLVQLSLICWFGNEIT
RASELIRLSLYESDWLSCSRRFKSSMILTMIRMQRPVYLSIGKFSPLTLATLVAVCRGSFSYF
ALFKSVQ
>CbowOR37
MYFNTIQKVLPILYIIGADPREGFTKSQFFLYFYNIFSAIGMVYLLVLKFANAENKVTVKDIT
DAVICLFLFCHGMVKSTTMFVKKNSVQTLLAQMEKHFWPMNNYKYSYIHNGILNICKTIR
NTTNFIWFMHFCNAMGFLVGPLITKDPVLPFECYRPEWMGYYTLLLFEDVTSIITILCPVL
AMDVFFVTIIKLTQIQWKMLNSEIQSMFDLSPSGKISREDEENIMVMKIKKCVVHHNFLLN
YQQLLNDTFSIPLFFFLIVIVLCMCVEMYVISTVSDWESLRTAIVYTATGCLEFMLCYCYPC

QDLSDEADNISYSIYFSNWYRNPEYFRDTQLIMQKGQKLVAIRPGGFMIMDLKTGLSVGIF
PSP
>CbowOR38
MFAGCIFTCTFWAICPFTEDVASLPIAAWIPFKTDSSPSFELAFAYEIIATVIGGITDLNADCF
MAGFIMVVCAQMKILNDSLLNLRHFAVEELNAETGGNNDDDGIAEELQKIMNRKLVECV
HHHRYILEFAEEANSLFTTSILGQFAVSVIIICTTLFEMTLVPFASIKFISLILYQYCMLMEIFIV
CYFGNEVILESSKLTKYAYHSDWRDCSQEFKRNLLFFMTRSQRALKLYAGGFFTLSLDTY
VKILKSSWSYFVVLIQVNKDSG
>CbowOR40
MYEKEFRNVFWLLNFVGMHPLKKYVTPFIVFNAILTFYITVLIILKLLWDKELVAVESLCVF
SQIWLKIFVLTTKKRKIKQVIDDTQQFWENDPENSENKQLLKNLAKLERIFLTYISCSTCMF
LFKPLLVKGTSIYYYYKIPQIPFYVSYPIEFYVTIVTMALAIAVNLFISIVIVIGAGQFSNLNA
KMKQLDLSIAEDCQDGLRTCTLEMNKNIEYHDFLIKYVSHLDEIFSMLFVVLTGIITSLLC
MNMYVLSQPTTTAVDMIRCGTMVCAFTSEFLFLYGVPAQRLMDEAEEVANSAFYHCQWY
LPNIIPLRKSLSFMIHRSQKSVCLSAMGFIDINRQTIVAMIKTAYSFFTFLQTIETTGGA
>OtauOrco
MMNFKVTGLVADLMPNIRLMQASGHFMLNYYADNNGALHTLRLGYCFMHLFLVLLQYG
FTFGNLVQESDDVNDLAANTITVLFFTHCLTKFVYFALRSKLFYRTLGIWNQANSHPLFAE
SNNRYHALALTKMRRVLAIVVIGTLASWIAWTTITFFGDSTHTRKDPNNENETITEEIPRLLI
KSFYPWNAMSGMKYYISLSYQVYYVLFSMLHSNLLDVLFCCWLIFACEQLQHLKEIMKP
LMELSATLDTYVPKSADLFRAPSVNSQDNLIDNDALDYNNMKNDELNLKGIYSTHQEMG
INYRGGNLQQFDSGGGGIGPNGLTKKQELLVRSAIKYWVERHKHVVRLVTAIGDAYGIAL
LLHMLTATITLTLLAYQATKIDSVSKYALTVLGYLFYALAQVFLFCIFGNRLIEESSSVMEAA
YSCHWYDGSEEAKTFVQIVCQQCQKAMSISGAKFFTISLDLFASVLGATVTYFMVLVQLK
>HoblOR1
MAPDGQFNNKISVRNFFKPYLIVLKLFTHFPFETKSTLYNIYSFSMIMLIFIIFDILESLYIIISL
HSIEDMVDSFMLYCTDLSYLMKLHCLVTKNGQIRKILKEISGDVFQPKSEYQEQLIITSLLTI
RRCTKLLIYIALGSCVFWVVLPLVSRSGEKLLPGQLWFPFNTSNHPVYDILYIYETIAVILHP
LTHICMDTIPLALMAFICGQFDVLDDTLINLKIFAAERLESNTKRTKDDLHEEMNKLLIECII
RHQETKRLANEVNDVYTSHFFAQLVNCVSVLSLIMYKLTTIPIASVGFFTLALYLTCLLGLL
LLYCWYGNEIVSKSSAVYLAAYSSDWTGCPISFQKNLLFMMLHLQRPVTMYAGRFFPLSL
DVYTAILRFTWSYFTLLLLNIDE
>HoblOR7
MNQFEIIHREKRMLGIFGLYSRRDDENGVFFKIRRALIAVFSTVMLICLFIKMYENRNNLLD
IFETCYYFMVQAAFVIKLYIYFYHLPDLHALEEMLSGKIFNSLSKEQEMYISDAMKSHKIFA
GCYQFCCVCCAIIYSIFPAMDGQALAVPIYSPFNVEKYRMFLYCFEVCCFFMTACNNSSFD
GMTVGLTTIMSAQLDVLKDNLKHAADRDMGGKFLEQEKKIKDRLRNCVRHHNAILEFTA

KTQDIFSTGVFFQILASMLGICLTGLQSLMVPWGSVQFASVGLFLVTEIIQIGMFCWFGENII
IKSSEIELSCYMSEWRSCSTSNKRIFLIIMENAKTPIKFTAKGLFILSLGTFVMVLRSGYSYF
AVLRQVSQK
>HoblOR23
MEDMKTFFSSNTFILKLSGMWWFLKQNNTHYKHVFYGIFCIIISQFYYLPSEWYRLKDIYS
DFDNLINQLGMLLTHQLGMFKLLNIFLRRNRILNMIQTFQNSYDFESCGKFKPQQIIADHK
KVARNAITLYFMLACCVPVTATISTAIQLSKMSSDEFNGGSEVCDSILPYYIVIPFDISTKISC
CFALIFQILPFVVFAWQITAHDLLFAVFLIALKCHFIIVRGAFETLRSRCLKMLNLQPHYNIL
HDRDNIKLDNAMKTEMRKCTQYYQIIIKLAQEVENSFTHIILVQVLIVIFIMVSCLYMMSSL
VITSGKFFAEANFVIAILIQITFYCWFGNEMTLASADICSGIYDNNWLSASESFKRSMLIQMI
RSSRITYITVGKFSPVTLLTLASILRGSYSYFALLSNLKE
>HoblOR2
MSAGNTTKPVIRKLSLEFSRDVYKDGVKMCILPGKVLLQGVCCWPDDEGLRMKIVGWFL
FWNLFVIEIFHASYVFLNFNDIGDAVDAGATVTTTMEGLVRLHIMLTKKGVINSTLVKIWK
QFWSLDVIEPVKRKKIKRQAQTAVMLTSIFLGSSIISNSQITGTPFIRNRGLVLKSVFPFDWR
QYYFYEIIYIWQYYSDWFVLFMINAFDFFFIALVIICSVQYVIMQEIFKYILTDESKRHRKIIF
GERGETMTDREMLFECLEQHKLLIGICNELESSAWFNIPILIQFFTSTCGICAATLIMKVDYS
QFSKMFTLVGAHISQLFCYCYVGQHLAFESDYLAYAIYDCGWHIDYDRSFRKALLLMMQ
RSQRTQRLTAAGITELDYASFLGILRLSFSFYTLLNNLFMKNVGQ
>ItypOR49
MTMTIYPKTENMRLTAIYSSTLGIFPWKFLFQDRKFYQKLYRYYSIFILTWYIGFVVTAYIEL
FVLLSGETIKMEEVCTNMCLTLVFTCAGLRACVMRYGNRLDDTIQSIIDTERNTNLLDDEN
VREYENKYISVMRTFTHCYAASVIIPDAQRSVFVAITSPQITEVGNVTVSYKPHIMSSWFPF
DKQEHYWPAYWFQVFDGSMGASFVAFVDIFMFNLISYPVGQLTKLQHLIRNLKTYQDKA
WQAGVQQADLSVLNDLVRRHQKIISYVDVYNNYMGTFAIFEFIQSSVQIASVLAQTSPDN
LTLEEASFIVCFFISMTIRMFLYYFSANQVIVESRKVAISVWESNWYEQRPEIQKSLLLMMI
RAQKPLCYRIGGFGIMSVESIIAIMKGTYTYITIIYRGY
>ItypOR46
MNAFPDSDSLTALKFTSVLGLFPWKLAFQQNKFYQTLYYWYSLFVLLWDIGFVFTSYVEL
VILLRGEVLHIDEICTNIRITTIYTCIIIRLVMIRTSSGLLKLIQEIIDSDKQVTIVDDEETTKLV
KTETSINNLKFIWYVGICCSIGLQFFIRPLVAEPEIIQIGNITEVGPRDLIILTWFPFDEQKYYW
VAYFLQVIDGIIGTLFVALSDVFIVNLILYPTTQLKKLQHIFRNFEHYQQSYKRLNSCETEES
AGIKVITHLVQRHQRIIKFVDTFNGWMGPLMVFDFLQSSIQIASILISDLRRDITFAMVCFIV
TFFVGMVLRLYLHYYSANELILESEKLADAIWYSNWYEQSPKIKYLMLIVIIRAQKSLKYN
IGAFGIMSLESGIAILKATYTYITVFTSNN
>ItypOrco
MMNKFKVAGLVADLMPNIRLIQASGHFMFNYYADDSGSLHILRLAYSCMHLFLVLLQYGC

IFGNLVREKDNVNYLAANTITILFFTHCLTKFIYFAVQSKLFYRTLGIWNQSNSHPIFLESNN
RYHALALKKMRNLLYIIIVGTIISACAWTAITFVEDSVHEIPDPDNENSTIVEAIPRLLIKSWY
PFNAMSGMTYYIALVYQIYYVFFSMFHSNLLDSLFCSWLIFACEQLQHLKEILKPLMELSA
SLDTYVPKSADLFKSPGSATSQDNLVDNDFNAKSDDLKGVYSTRQELGNLHFRSGALQTF
GQGGGGVGPNGLTKKQELLVRSAIKYWVERHKHVVRLVTAIGDAYGVALLLHMLTATIM
LTLLAYQATKINGINPYAATTLGYLIYSLAQVFHFCIFGNRLIEESSSVMEAAYSCHWYDGS
EEAKTFVQIVCQQCQKALSISGAKFFTISLDLFASVLGATVTYFMVLVQLK
>HabiOR4
MTFITGADNLQIESKNVDHVNIKYPSKVFTATENLEMFVGQKKFSDKTLMAIKIIYSFYNY
SLFSAAVIFIVSELVAFRKALGDTSALVSQIGMMFTHLVGMGKLWVLVHNREKIEDVKKQL
QDKQYHYMPLGDFQPGRRMRREKLISMFIATFVFLLYTFVGVSAHISAALMVQRNTIGDT
FIGNTSCDSFTPYYYYYPFDVSTATSCYYSLAYMDISLDIFAWYIATLDMVFVSLLHILRTQ
LDILGEALTTIRKRCLNKLKMNPTFSILYDTDHPELEEEMYSEITRCTKHLYSLLGIRNDIEG
IFNFITLAQTLASLLIFASCLFVAAQEPLTSPNFFSQMEYFCAVLAQFSVYCWFGNQMTIAG
EALPTAIYNSDWFSASPRFKNSMLQTMTRMQRPLFVSIGKFTPLALPTLLAVIKGSFSYFTV
FQSAGNVE
>ItypOR6
MLRKNPRYATDFFSVNQWMLRRAGLWRPSNKNKTVQFCYTLYAIFVFIFVNLWFTSTEFIS
LFYTYKDKYALIKNVNFFLTHFMGAIKVVFWYFCGQYLMEIMQDLENPNNHYEGYKDY
QPGIISQKYKKQGSKYSLMFLMLAHATLTSSYVFSTITTIQHMKGNSTVALPDRLPYYSWM
PFSYDTGPKYLLAMAYQAGPMFSYAYSIVGMDSLFMNIMNCIAANVTIIQGAFKTIRERAL
PGQPNNVLHESKADMDVMRVELRKIVNHLQTIFKACDKLENVHRMVTLCQVTATLFILC
TCLYLVSIAPPLSKQFLVEFVYMLAMSFQLYLYCWFGNEVTIKFQELPRYIWASSWLATDT
QFKKALLFTIMRTKRPVFLTAGKFSRLILPTFMSILKTSYSIFALIRNTSK
>HabiOR3
MFNRTYARDFFVVNRWMLMCAGLWRPETKNKYIQFFYTVYAVTVFVFVNLWFTFTEFVS
LFYTYKDKYELIKNVNFFLTHLMGALKVIFWYFYGNFLVEIMKTLEDPQLQYESYKHYQP
GEISHKYKKTGMRNSLLFLSLAHATLISSYIPPTVSVLQYIRHGENMDNSTMSLPSRLPYYS
WMPFSYNTGYTYLLAVAYQAGPMFSYAYSIVGMDTLFMNLMNCVAGNVTIIQGAFLTLRE
RCISRSDSISLSHNTLYENELLMTSMKIEIKKIIKHLQITFKACEKLESIHRYVTLSQVTATLFI
LCTCLYLVSMAPPLGKQFLTECVYMAAMFFQLFLYCWFGNEVTLKFQELPTYIWQSDWIA
TDSFFKKIMMFTMMRARRPVFFTAGKFSPLTLPTFMAIIKTSYSIFALIKNTTS
>ItypOR5
MLNHDYPKNVLSSVDTILLICGLGKISKKIPWVIRLAYSIYNYLIMSIALIFLMFELIAFQMA
LDDLPTFLSQIAMVLTHSAGFVKLWMLLYMMNPMEKIRNKLQDGRFKYVPVGNFQPGLK
MRRAKVLMSRVTVLIFTMYSFVGVSAHISAAVDVIKNTRNGKFVKGITCHDILPFNFYIPF
DISTPTMCHYALMYMNISLDAQAFYIATFDLIFVCFLLFLSAQLDILSDAFTTIRKRCLKKL

EMDSEKQCFYDDQCRELEEEMYREVTHCNQHLNLLIEVRNDIEHVFSLITLIQTSASLLIC
ASCLFVAAQVQGNSPTFFSQLEYTAAILSQISLYCWFGDKITISSSEIPMALYKSDWLSCSQR
FKKSMLMAMTRMRKPLYVSIGKFTPLALNTLLAVLKGSFSYFTLFQRAG
>MaltOrco
MFNYHADNSGALHTLRLLYSCMHLVFCLFQFGCIFGNLVVEKDDVNYLAANTITVLFFTH
CITKFVYFALRSKLFYRTLGIWNQSNSHPLFVESNNRYHALSLKKMRTLLICVSATTVLSA
AAWTGITFVEESVHNIKDPNNENETITEEIPRLLIKSWYPWDAMSGMAYYGSLVFQIYYVL
FSLTHANLMDSLFCSWLIFACEQLQHLKEIMKPLMELSASLDTYVPKSADLFRAPSAKSQD
NYIESDYNTKNEELNLKGIYNTRQELGGNFRSGALQTFGQGGVGPNGLTKKQELMVRSAI
KYWVERHKHVVRLVTAIGDAYGVALLLHMLTSTVMLTLLAYQATKINGVNTYAATTIGYL
VYSLAQVFHFCIFGNRLIEESSSVMEAAYSCHWYDGSEEAKTFVQIVCQQCQKAMQISGA
KFFTISLDLFASVLGAVVTYFMVLVQLK
>AchiOrco
MMKFKVSGLVADLMPNIRLIQASGHFMFNYHADNSGALHALRLGYSCAHLLFCLFQYGC
IFGNLVVEKDDVNYLAANTITVLFFTHCITKFVYFALRSKLFYRTLGIWNQSNSHPLFVESN
NRYHALALKKMRTLLICVTATTVLSAAAWTGITFVEESVHNIKDPDNENETITEEIPRLLVK
SWYPWDAMSGMAYYGSLIFQIYYVLFSLAHANLMDSLFCSWLIFACEQLQHLKEIMKPL
MELSASLDTYVPKSADLFRAPSAKSQDNYIENDYNAKNEELNLKGIYNTRQELGGNFRTG
ALQTFGQGGVGPNGLTKKQELMVRSAIKYWVERHKHVVRLVTAIGDAYGVALLLHMLTS
TVMLTLLAYQATKINGVNTYAATTIGYLVYSLAQVFHFCIFGNRLIEESSSVMEAAYSCHW
YDGSEEAKTFVQIVCQQCQKAMQISGAKFFTISLDLFASVLGAVVTYFMVLVQLK
>RvulOrco
MNTFKVAGLVADLMPNIRLIQASGHFMLNYHADNSGALHGLRLGYCCMHLLFVLLQFGC
IFGNLVKEKDNVNDLAANTITILFFTHCLTKFVYFAVRSKLFYRTLGIWNQANSHPIFIESNN
RYHVLALKKMRNLLYIIMIGTIFSASAWTGITFMGDSVHYIKDPNNENETISEEIPRLLIKSW
YPFDAMSGMPYYIALVFQVYYVLFSLLHANLLDSLFCSWLIFACEQLQHLKEIMKPLMEL
SASLDTYVPKSADLFKAPNSASSQDNLIENEYNSKNDELNLKGVYSTRQELGNLTFRSGA
LQTFGQGGGGVGPNGLTKKQELMVRSAIKYWVERHKHVVRLVTAIGNAYGVALLLHML
TATIMLTLLAYEATKIDGVNVYAATTIGYLLYSLAQVFHFCIFGNRLIEESSSVMEAAYSCH
WYDGSEEAKTFVQIVCQQCQKALSISGAKFFTISLDLFASVLGAVVTYFMVLVQLK
>RferOrco
MNTFKVAGLVADLMPNIRLIQASGHFMLNYHADNSGALHGLRLGYCCMHLLFVLLQFGC
IFGNLVKEKDNVNDLAANTITILFFTHCLTKFVYFAVRSKLFYRTLGIWNQANSHPIFIESNN
RYHALALKKMRNLLYIIMIGTIFSASAWTGITFMGDSVHYIKDPNNENETISEEIPRLLIKSW
YPFDAMSGMPYYIALVFQVYYVLFSLLHANLLDSLFCSWLIFACEQLQHLKEIMKPLMEL
SASLDTYVPKSADLFKAPNSASSQDNLIENEYNSKNDELNLKGVYSTRQELGNLTFRSGA
LQTFGQGGGGVGPNGLTKKQELMVRSAIKYWVERHKHVVRLVTAIGDAYGVALLLHML

TATIMLTLLAYEATKIDGVNVYAATTIGYLLYSLAQVFHFCIFGNRLIEESSSVMEAAYSCH
WYDGSEEAKTFVQIVCQQCQKALSISGAKFFTISLDLFASVLGAVVTYFMVLVQLK
>TmolOrco
MMKFKVSGLVADLMPNIRLIQASGHFMLNYHADNSGAVHTLRLGYCIMHLIFMLLQYGC
NFVNLIFERGDVNDLAANTITVLFFTHCITKFVYFAARSKLFYRTLGIWNQPNSHPLFVESN
NRYHALALKKMRRLLYIIIIWTSFSAIAWTSITFVGDSVHNIKDPDNENMTITEEIPRLLVKA
WYPWNAMSGMPYYITLVFQVYYVFFALSHANLLDSLFCSWLIFACEQLQHLKEIMKPLM
ELSASLDTYVPKSADLFRAPSATSQDNLIENDYNTKNEDLKGVYSTRQELGGHFRGGALQ
NFGGVGGGVGPNGLTKKQELMVRSAIKYWVERHKHVVRLVTAIGDAYGVALLLHMLTST
IMLTLLAYQATKITGVDKYAATVIGYLLFALAQVFHFCIFGNRLIEESSSVMEAAYSCHWY
DGSEEAKTFVQIVCQQCQKAMSISGAKFFTISLDLFASVLGAVVTYFMVLVQLK
>CchiOrco
MMKFKVQGLVADLMPNIRLIQMSGHFMFNYHADNSGALHTLRLAYSCMHLVLCLAQFG
CVFGNLVAEKDDVNNLSANTITILFFTHCITKLIYFAVRSKLFYRCLGIWNQANSHPLFVES
NNRFHALSLKKMRTLLYCVCATTVVSAAAWTGVTFVGDSVHNIPDPDNENETITEEIPRLL
IRSWYPWDAMSGMPYYVSLVFQLYYVFFSMAHSNLLDSLFCSWLIFACEQLQHLKEIMKP
LMELSASLDTYVPKSADLFRAPSATSQDNTIESDYNAKNEELNLKGVYNARQELGSHFRS
GALQTFGQGGGGVGPNGLSKKQELMVRSAIKYWVERHKHVVRLVTAIGDAYGVALLLH
MLTATVMLTLLAYQATKIDGVNKYAASVIGYLVYSLAQVFHFCIFGNRLIEEVRVIDTLFM
HNILQLKYIGLYRVDPLYPTWWKT
>CchiOR8
MNLCGVHPAKVGSKVQMVVFLFLIINFSITTYWMAMFFVVKEQKDFNMFVMSLANVLC
TVHAILYLSCLYVQRPSIMQLLTEMREKFWEIDIYTDKALIENYQSSLDKVQMKYALFCFI
MFYTALTYLYWRILTDQRPIGDNLLFESYVPEGMSFWFLLAWQHIPAGALISIEMAMDFLI
CSILSLTAQQYRLLRYEMERVFGNLERKEDTEENISKRIRRCNDQLTFLLSFRNTLNEAFSY
LMLAYIGVVVLILCFEVYLLFQMDSIEHIIKILAYSGFIFFEFFVCYCYPAQDLTVDAEQLAN
SIYFSEWYKYPNHCKEILMLVGKSQIRVVFTAGGLLELDLPTGMAAIKTMFSYSMFLRTM
TMIE
>CchiOR10
MGKLLLLVPHVAMLLNTCVGLFEFATLIYNQPKMAKLKKLLQSESFNYEECSNTTKIVEDS
RAFYNRITIASYLIYWMVAVGGHLSALKNLNAESRAGRYGVNTTCYDLIPHLFVIPFQTDT
TRRCKNALMVMDFGLLIIAGYIATHDTVLYSFLSCIKAKFEVVSEATITIRERTALKMHMD
KHFEMLNDEEFPEFEELMYCEIKRCNANIVILIRACKDLESIFCYTLLGRSVSSLLVIAACLY
IGSFLSPRDPEMYHLAEYLISGFFQMFMICYFGVFITERGAAYKTCLYESNWYSCSSRFKQ
AMLIMMNQMQRPLYLTIGKFFPLSLQTFVQVTKAAFSYATVLKTV
>CchiOR16
MTTKRSKNLFEYQDVIFVLNGVPLHSVSYFKRVIITLWGVVLLAFCTCFYYLEAMMMKN

NVKELSVFFQQIGILISALVDILKLLAIAMHWKRLFSIRLRLESDEFQYEPYGSFQPDQLLSN
SNKFCKRLTAFMLCYYMTAFSSHYSALSRLNLEQSDYYFQDNSSCYDYLPYYYHIPFDTN
TIRRCEYALIGMDIGMCIYIGYTLVYDNLFFALIIYLQTYLQILAEATKYIRERTLESLKLPAD
YDMFRDDENPALEKATYYEIRKCCRNLLFLLRIQEDIETVSTYITMSQTISTLLTLATNLYTL
SMTSPSDADFLGLLVYILLGILQMTMICHFGYSVTETVSMSSIALHETFVIKRCSWISDSNN
FGLYNANCKTFRITVSFLVPLSNNFTYSK
>DvirOrco
MMKFKVSGLVADLMPNIRLIQASGHFMFNYYADNSGSLHTLRVGYCCVHLVLCLLQYGC
TFGNLVVEREDVNDLAANTITVLFFTHCITKFVYFALRSKLFYRTLGIWNQPNSHPLFIESN
NRYHALALKKMRTLLICVVATTVLSAAAWTGITFVGESVHNVKDPNNANETIVEEIPRLLV
KSWYPFNAMSGGAYFLSLGFQIYYVLFSLMHANLLDSLFCSWLIFACEQLQHLKEIMKPL
MELSASLDTYVPKSADLFRASSATSQDNLIENDYNTKNEEINLKGVYNTRQEMGANFRSG
ALQTFGPGGGGVGPNGLTKKQELMVRSAIKYWVERHKHVVRLVTAIGDAYGVALLLHM
LTSTVMLTLLAYQATKINGVNPYAATVIGYLVYALAQVFHFCIFGNRLIEESSSVMEAAYSC
HWYDGSEEAKTFVQIVCQQCQKAMSISGAKFFTISLDLFASVLGAVVTYFMVLVQLK
>AquaOrco
MMKFKVSGLVADLMPNIRLIQASGHFMFNYHADNSGALHALRLGYSCLHLVLCLVQFGC
TFGNLVIERNDVNDLAANTITVLFFTHCITKFVYFAVRSKLFYRTLGIWNKANSHPLFLDSN
NRYHALSLKKMRTLLICVMTTTILSASAWTAITFVGDSVHNVKDPDNDNETITEEIPRLLIK
SWYPWNAMSGTAYYVSVSFQIYYVFFSLAHSNLMDSLFCSWLIFACEQLQHLKEIMKPLM
ELSASLDTYVPKSADLFRAPSANSQDNLIENEYNEKNEGLNLKGVYNTRQEMGANFRSG
ALQTFGQGGGGVGPNGLSKKQELMVRSAIKYWVERHKHVVRLVTAIGDAYGVALLLHM
LTATVMLTLLAYQATKIDGVNKYAATVIGYLVYSLAQVFHFCIFGNRLIEESSSVMEAAYSC
HWYDGSEEAKTFAQIICQQCQKALSISGAKFFTISLDLFASVLGAVVTYFMVLVQLK
>CbowOrco
MMKFKVSGLVADLMPNIRLIQASGHFMFNYHADNSGALHALRLGYSCMHLVFCLFQFGC
TFGNLVVERDNVNDLAANTITVLFFTHCITKFVYFAVRSKLFYRTLGIWNQANSHPLFVES
NNRYHALALKKMRTLLVCVMATTVLSASAWTGITFVGDSIHHIKDPDNENETIIEEIPRLLV
KSWYPWDAMSGTAYYASLIFQIYYVFFSLAHANLMDSLFCSWLIFACEQLQHLKEIMKPL
MELSASLDTYVPKSADLFRAPSANSQDNLIENDYNAKNEEINLKGIYNTRQELGINFRSGA
LQTFGQGGGGVGPNGLSKKQELMVRSAIKYWVERHKHVVRLVTAIGDAYGVALLLHML
TSTVMLTLLAYQATQIGGVNKYAATVIGYLVYSLAQVFHFCIFGNRLIEESSSVMEAAYSC
HWYDGSEEAKTFVQIVCQQCQKAMSISGAKFFTISLDLFASVLGAVVTYFMVLVQLK
>AglaOrco
MMKFKVSGLVADLMPNIRLIQASGHFMFNYHADNSGALHALRLGYSCAHLLFCLFQYGC
IFGNLVVEKDDVNYLAANTITVLFFTHCITKFVYFALRSKLFYRTLGIWNQSNSHPLFVESN
NRYHALALKKMRTLLICVTATTVLSAAAWTGITFVEESVHNIKDPDNENETITEEIPRLLIK

SWYPWDAMSGMAYYGSLIFQIYYVLFSLAHANLMDSLFCSWLIFACEQLQHLKEIMKPL
MELSASLDTYVPKSADLFRAPSAKSQDNYIENDYNAKNEELNLKGIYNTRQELGGNFRSG
ALQTFGQGGVGPNGLTKKQELMVRSAIKYWVERHKHVVRLVTAIGDAYGVALLLHMLTS
TVMLTLLAYQATKINGVNTYAATTIGYLVYSLAQVFHFCIFGNRLIEESSSVMEAAYSCHW
YDGSEEAKTFVQIVCQQCQKAMQISGAKFFTISLDLFASVLGAVVTYFMVLVQLK
>GdauOrco
MVRSAIKYWVERHKHVVRLVTAIGDAYGVALLLHMLTSTVMLTLLAYQATKINGINPYAA
SVIGYLVYALAQVFHFCIFGNRLIEESSSVMEAAYSCHWYDGSEEAKTFVQIVCQQCQKA
MSISGAKFFTISLDLFASVLGAVVTYFMVLVQLK
>SvelOrco
MMNNFKVTGLVADLMPNIRLIQASGHFMLNYHTDSSGPVHLLRVAYCCMQLFFILIQFGAI
FGNLVAEKDNVNDLAANTITILFFTHCVTKFIYFALRSKLFYRTLGIWNQANSHPIFIESNNR
YHALALKKMRTLLYIVLVGTLFSIAAWTGTTFVGESIHYIKDPNNENETIAEEIPRLLIKSW
YPFDAMSGMAHMIALIFQIYYVFFSLFHSNLLDSLFCSWLIFACEQLQHLKEIMKPLMELS
ASLDTYVPKSADLFKAPGSASSQDNLVDHDFNSKSEESLKGVFNAATQELGNLHFRTGAL
QTFGQGGGGVGPNGLTKKQELMVRSAIKYWVERHKHVVRLVTAIGDAYGVALLLHMLTS
TIMLTLLAYEATKIDGINTYAASTLGYLLYTLAQVFHFCIFGNRLIEESSSVMEAAYSCHWY
DGSEEAKTFVQIVCQQCQKALSISGAKFFTISLDLFASVLGATVTYFMVLVQLK
>PbreOrco
MMQFKPQGLVADLMPNIKLIKFSGHFMLNYYADNSGAVHTLRLGFCFAHLFLLLLQYGFT
FGNLVKESDDVNDLAANTITVLFFTHCITKFIYFAVRQKLFYRTLGIWNQSNSHPLFLESNN
RYHQLALTKMRRLLIVVMVGTMTSWIAWTTITFFGDSVHTRKDPNNENETITEEVPRLLV
RSWYPWDAMSGVPYYISLVYQIYYVGFSMLHSNLLDSLFCSWLIFACEQLQHLKEIMKPL
MELSATLDTYVPKSADLFRAPSAASRDNLIDNDYNQRNEEATMKGFYTTSQEMGVTYRS
GNIQDFSGGIGPNGLTKKQELMVRSAIKYWVERHKHVVRLVTAIGDAYGIALLLHMLTSTI
TLTLLAYQATKIDGVNKYALTVLGYLIYALAQVFHFCIFGNRLIEESSSVMEAAYSCHWYD
GSEEAKTFVQIVCQQCQKAMSISGAKFFTISLDLFASVLGATVTYFMVLVQLK
>OcomOrco
MMKFKVTGLVADLLPNIRLIQASGHFMFNYHADNSGSLHTLRVAYSCMHLIFCLFQYGCT
FGNLVKEKDDVNYLAANTITVLFFSHCITKFVYFALRSKLFYRTLGIWNQPNSHPLFLESN
NRYHALTLKKMRTLIVCVVAATVLSAAAWTGITFVGESVHNIKDPDNENDTIVEEIPRLLIK
SWYPWNAMSGMTYYITLVFQIYYVLFSLMHANLLDSLFCSWLIFACEQLQHLKEIMKPL
MELSASLDTYVPKSADLFRAPSANSQDNLIENDYNAKNDELNLKGIYNTRQEMGINFRSG
ALQTFGQGGGGVGPNGLTKKQELMVRSAIKYWVERHKHVVRLVTAIGDAYGVALLLHM
LTSTVMLTLLAYQATKINGVNPYAASVIGYLVYALAQVFHFCIFGNRLIEESSSVMEAAYSC
HWYDGSEEAKTFVQIVCQQCQKAMSISGAKFFTISLDLFASVLGAVVTYFMVLVQLK
>PverOrco

MMKFKVSGLVADLMPNIRLIQASGHFMFNYYADNSGAVHTLRLGYSCMHLIFCLLQYGA
TFGNLVLERDDVNYLAANTITVLFFTHCITKFVYFALRSKLFYRTLGIWNQSNSHPLFVES
NNRYHALSLKKMRTLLICVMTTTVLSAVAWTSITFMGDSVHRVKDPDNKNETIIEEIPRLLI
KSWYPWNAMSGMTYYISLIFQIYYVLFSLTHANLLDSLFCSWLIFACEQLQHLKEIMKPL
MELSATLDTYVPKSADLFRAPSANFQDNLIENDYNTKNEELNLKGVYSTRQELGANFRSG
ALQTFGQGGGGVGPNGLSKKQELMVRSAIKYWVERHKHVVRLVTAIGDAYGVALLLHM
LTSTVLLTLLAYQATQINGVNTYAASVIGYLVYSLAQVFHFCIFGNRLIEESSSVMEAAYSC
HWYDGSEEAKTFVQIVCQQCQKAMSISGAKFFTISLDLFASVLGAVVTYFMVLVQLK
>TcasOR1
MMKFKVTGLVADLMPNIRLIQASGHFMLNYHADNSGALHTLRLGYCCMHLVFVLVQTFS
CNFVNLVLERGDVNDLAANTITVLFFTHCVTKFVYFAVRSKLFYRTLGIWNQPNSHPLFVE
SNNRYHGIALKKMRRLLYIIIIWTSFSAIAWTGITFVGDSVHNIKDPENENLTITEPIPRLLVK
AWYPWDAMSGMPYYITLVFQVYYVFFSLAHANLLDSLFCSWLIFACEQLQHLKEIMKPL
MELSATLDTYVPKSADLFRAPSATSQDQLIENGTNPAKKNEDLKGVYSTRQELGGHFRGG
ALQNFGSGGVGPNGLTKKQELMVRSAIKYWVERHKHVVRLVTAIGDAYGVALLLHMLTS
TIMLTLLAYQATKITGVDKYAATVLGYLLFALAQVFHFCIFGNRLIEESSSVMEAAYSCHW
YDGSEEAKTFVQIVCQQCQKAMSISGAKFFTISLDLFASVLGAVVTYFMVLVQLK
>TcasOR3
MKLSSVTTCLFSSDFHTRMNFDWKDTIKLNFLMMKIVGLWPKEKYKINFYTLYTLISVNL
FICGHVIFHTVAVFVVGRDLKHLIGALYMSLTETLLLVKICYFIKNSRLVKSLLTSLDGDIFQ
PKNEKQLELTNPSLIFWKKVHKSFAILVANTVFLFVSLPILSKSTKLYRLPLEAWYPYNTQK
SPNYEITYLYQFISTLFRGMASVSMDTFIAALNMYIGVQCDILCDNLRNLNETNFMENLSL
CIKHHKAIVSFARECNKFYNGIVLGQFFSTSIALGLAMFLLSLVTPLSTESNTLLFYLGATTS
EIFLYCWFGNEVDVKSSKIPYSAFESDWTGAPIEAKKNLLIFILRTQKPIKMSAINLFSLSLE
TFTTILRTSWSYFAVLRQVNGQA
>TcasOR7
MNKLQKFDWKATIRPNIAFLHYLGIWPEGEEYYKLNFYTLKTILYIIILVISTIVFQVINIFFT
LDDLTSLTANIYVLLTEILYFIKLCFLVKNMPALKLLMKTLDHKLFQPKANQIVIIQPLLNFW
KLIFLAFVITCSFTVLFWAIFPILDSSEEEKRLPLLAWYPYDTKISPNYELTYLHQVASYIYIC
YSHLNIDTFITALNTYIQCQFDILCDNLKNIKSDTKNVDTKLAKCIKHHLLILMFANTSNEF
FSWIIFFQFTSSAAITGMTLFQLTVVKPFTTEFYNFMAYVTAEVVQIFMYCWFGNEVQVKS
SNIPYAAFGSDWTEFSPNKQKSLLFLITRSQKSVKMSAFNVFDLTTDSFILKSAWSYFALLN
QVNS
>TcasOR17
MDDFNWISTVKTNLLLLHIGGIWPRGDGTHKLNLYTIYAIFITFTFTTYHCFSQIINFFFVDD
LQALTESIFISLIQSMALVKAFYILKNMRILKNILKNLETNKMLQPRNLKQIKMVQPSLTQW
RLLSQMFWISAVFAMCLFGAFPIVESTYKEFRLPYLAWYPFDTKSSPFYEIMYLHQFVSSY

TIAIVDIGADTLIAALNVFVATQCEILCDNIRNINGSVEEMDSKWKECFTHHKEILKVARHC
QKFFNWIVLMQFCASVICIGLTMFQLTLVVSFSSEFFSSLFYFGAITVQIFMYCWFGNEVEL
KSSKILYATFEANWVEAPHQVKKNILIFAIRCQNPIKMSSLNVFYLTLETFMAIFRTSWSYFA
VLRQIQNRISEE
>TcasOR20
MNSFNWQESIKTNLKALRLVGLWPKSDFYKFDLYTFCTSLTVGVIVCGHNLSQIVYILQVY
SDLKALTATIFVASINFLGAVKMYFFIKHIKTVKILFKMLKTYQFKPKNIHQTQLIKPFLNLW
KILYVGYSINVYLIVAMWSLLPVLNGWTWQKKLPFPARYPFDVTKSPYYELAYVYQFICI
WYITVANLNLDTINIALMMYTSCQCDLLCDDLKNLTETRFFHKKLIECIKHHKAILVFAEK
SNGLFNMIVLSQIATSTVVLALTMFQLSMVSPLSSEGLNHLFYIGGIIMQILLYCWFGNEVE
AKSSNILYAIYESTWFEASKNSKKNLLIFSIRCQRPIKATAVKLFALSLRTFITIVRSGWSYFA
VLYNVGSK
>TcasOR24
MEEDFDFLSSLQTTLFCLRCVGTWPSNTYKLDAYTLYATASITICLFGHNFFQTVNIFFIFND
LNTLTGVIFVALTCLVAILKSLLFIFNMRRLKKLLLVDIRQKLFKPRNRQQVVMVQSRVNF
WKKIYFMFTGMGVATMFFWALFPIMDGTVKEHRLPFLAWYPFSVNKSPFYEITYIYQIVSV
FFIVIVNMNSDMLLVALMNILGVQCDLLCDNLKNIQFRERINEEFLRCVNHHMQILSYASD
CNKFFNTIVLAQFFTTVVSLGLTMYQLTIVTPFTSEFYSFIVYGGAVLMEIFLYCWFGNEVE
FKSLNIPFASFGFDWTIGSVGLQKNLIIFIAKSQRPIRMSALNLFHLSLETFVKILRTAYSYFA
LLNNVNSLN
>TcasOR46
MSKSEKIHTLATYFDSNIAFLKLTAFWIYDDETTRRKKYLQHAYNIFWIFYLFVAYQPAELL
YVYYSFNDLSVFLRALRDIGNHVSLAYKAFNYFIMRRDILKLMETLQHGNYHYEDCGDF
QPKLIVDEEKKEALKWTKYFLNFCNAICLSMFANGVFTFIFLSDKQYVERNGQRVYHQEQ
PVNTVSPFGSGTKLRFFVTFIYTMIALTFYAWTIVALDSLFITIMSCISSHLKILQGAFKTVRA
RFIKLCASLSKLLISVSGKLESIYSTQTFVQTFISLGEMCFSLYLLSETADQNIGNEITYLIATG
FELLMYCWFGNRITEASLKISYALYESDWFPTSLSFKKQIIFTMTRMQKPINVTIGKITPLAF
STFLTIARGAYSFFTFLKQRHGINH
>TcasOR58
MPFTIKDYDLRNAFETERTLLTLSGFYPRRTKKYNFFYNTSALINLFIAYGQLFSMVVQMVI
DRNELSKLSETLLFFMTHFTFLCKLTNFVYYKKKMFEIEDNLSRKIFYGFELWQIKPKIDSC
KFIAKIFRILCILVVLFYTLVPYLDDKEDLSLPLPGWLPYNTKKYYYPTVIFQVMSVSVSAY
NNSSIDVLTCMLITVASAEFNLLKGALKTIDFHPKGHNTKQLIEAKFENCVNHHKEIVKFA
YQIETIFSKGIFLQFFASIIVICFTGFQMIVVPIPSMQFIFLIIYFSCMMCQVAMYCWYGHDIIT
TSDSIGQAFYMSNWYESDVKIRKNICIFLERTKKPVILTAGKFVTLSLTTFTTILRSSYSYFAV
LQHLYKEDS
>TcasOR59

MDEEFLIGTFETEKKFLRYGSFYPCGKRIKFIFLGLFMFVYSWTEFLSMITVLFVERDNLTK
LSETLLFCMTQAAFLFKLVNFLYHNKTMLRIESILKNPILNCLDQFEKNIIEKYMIRVKYLA
RLFRILCILTVSFYGLFPFIDEDPDHMLPLPGWFPFDVKTHQIELVIAQTCGIAIGAFLNSTLD
ILPTILITLGSAQFDILKIRLENITSVDTSKSWLVKKAIKKCVIYHTILLNYITQIEILFHKGIFV
QFTASVVVICLTGFQMLVISVRSIQFILLMIYFSTMTCQIALYCWYGNELMYRSMGLSDAC
YMSEWNKCDTSVCKSLAIIMERGKRPVVLKAGNIFSLKLTTLMTVLKSSYSYFAVLQRLY
ATSE
>TcasOR60
MSEDYTFRNVFAREKKILTISGFYPLREYEKNYFHFFSGTIQWIISLGMLFSMIIQSVIKRND
LMVLSETLYFLTTHLTFVCKLANLEYHKKLLLDIEDMLKTTRFQKTLSLDLIEKTGMNEKI
RKFNLVAKTFRIVCVWCVVLYVLVPYFDPGKSKTLPTPGWFPFNWTDKYYYGTYFFEVA
GISITAHMDSSIDILSWLLVTIASFQCDILKENLKNIYYNYDKEHDIRETFKDCIRHHEEIIKF
TTKVEQSFSQGILLQFLCSALVICFTGFLMLVVPVLTFQFANTIMYFCCMMIQLGMYCWYG
HEIMTTSDEIGQYFYLANWYDSSLTLRKDFAIFLERAKRPITLTAGGFVVLSLNTFTRILRSS
YSYFAVLKHLYNKS
>TcasOR61
MGDYDFRAAFAFEKAIFSLSGYYQRQAGFSSLIICAIASLITIAQFLSMVMQIIVAGNDLTVL
SETLLFFMTHFTYMCKLVNLLFYKSKLLHIEDLLSRPRFYGFSQNELTIIKDGIEATNTVANL
FRIFCVLACIAYGLVPYLDHTKAMALPLPGWLPYDTTKYYYPTYFFQMVAVSITASVNSTI
DILTWKLITIASVQFDILKRKLKDLDYKLETTSLQIQFKTCVKHHKEIVNYVKNVEKTFSK
GIFIQFFASVIVICFAGFLIIITPVLSMQFLYLTLYFMCMISQVAIYCWYGHYVMTTSDEIGQD
FYMSNWYESDVAFRKDIIIFMERVKKPVTFTAGNFITLSLVTLTRILRSSYSYVAVLQHLYNE
V
>TcasOR63
MGFMIQDYDLRNAFSLERKLMLVVGFYPKRDNKHEILYWLSAFFNLLISYGQLTTMIIQM
VFDRSDLSKLTESLLYFFTHFTFLCKLLNFQYYSKDLIEIENFLTDPIFYGYSFEQLDIIKAKI
RSCAFISNAFRICCTFTCSFYCLVPFIDESRKKILPLPGWFPYDTTNYYYSTFFVQSLSLFISA
YCNTAIDILTWKLITLASAQFEILKENLTKIDYEGGFNETKGALVRCITHHAKIVNYTERVE
AIFSKGIFLQLFGSVIVICTTGFQLIVVPIPSVQFAVLGTYLCGMTTQVATYCYYGHEVMTTS
DAIGMSLYLSNWYASHVKIRKIVMIFLEKTKKPTIVKAGNFITLSLATLTQILRSAYSYFAVL
QRLYKDS
>TcasOR64
MMSDEYVKDVFIANRWMLRCAGLWTPSTRSKLVQIPYKIYAIVVFLFVNVYFTSTEFLSLF
YTHKNLYNFIKNVNFFLTHFMGAVKVIFWFFKGHVLRDLMRTLESPEFHYEPCEGFQPGLI
WRKYRRIGFKYSLGFLALAHMTLSSSYIPPLLTKLPYFSWMPFSYSTPRSYLLALGYQAGP
MFSYAYSIVGMDTLFMNIMNFIAAHLVILQGAFASSKMRVLDPGQMNNEMKRNCRHLQTI
LRVSEDLERVHRYLTLGQLTATLFILCTSLYLISTTPASSKQFYAELVYMVAMGFQLYLYCW

FGNEVTLMASEIPVNVWKADWYDCDQSFKKSMIFTMTRMQKPIYMTVGKFAPLTLQTFV
YILRTSYSIFAVIKNTSI
>TcasOR65
MTATKSLKEIPPIYLRVHLTVLQILGIDILPVESVPQNLFYTYTALIISTMCLFTIAEFLDMVL
NYEDIYRLTFGLCYCVTHVLGTVKMFLMLYLRKKLWGNLTTLEEGIFKPNPTRGGPEELQI
VNDAITMCNRQGYVFYTLVFLIIGARLLYASLANWPYDKHNYFDGNVTVIVNTKEMPYTT
WMPFDYNDSPLYETIFAFQIFSTTVYGFYIGAADAVICGFMMLIKAQFLIVKRELETLIERA
QKAAIAENPDNEDNFGREIERIELLDKRTQDYVAKYANECVYHHQELIALCDHAEEDFCY
LMLLQFISSLLIVCFQLFQVSTLSPDSVEFFSMVCYLLLMLFQLLCYCWHGNEVQIVSGEL
SRYAFGINWIIMRESPKKTLLLLMMRAQRPCYFTAGKFSLLSLQTFMTIVRGAGSYFMFLR
QMNI
>TcasOR66
MSKNLKEIPPVYLKVHLTVLQILGIDILPNERIPQTLFYTYSVLLIATMVVFTTAECLDLVLN
YEDIYKLTFGLCCCVTHVLGAAKMFLMLYLRKKLWGYFTTLENGIFKPNPCRGGAEEFEI
VTSAINMCKRQGYVFYVLTVGVTGGQGLYAALANLPYDKHNYFDGNVTVVVNTKQMP
YATWTPFDYNDSPLYEIMFAFQIFSTTLYGFYIGAADAVICGFLMLIKAQFLIVKRELETLVE
RAQRAGNPDRGDFGGGINRIEMLDDGTQVFVEKCANECVYHHQELIALCEHAEEDFCYL
MLLQFISSLLIVCFQLFQLSTLSPGTFEFFSMACFLLFILFQLLCYCWHGNEVQFVSGELSR
YAFSINWIIMRESPKKTLLLLMMRAQRPCYFTAGKFSLLSLQTFMTVVRGAGSYFMFLKQ
MNT
>TcasOR67
MDFTIRDFDLRNSFSLERKLLLVLGFYPIRDKEKHRILHQLSAFLNLLLYYGQLLTIIIQMVI
DRNDLSKLTDSTLYFLTLFTFLCKLFNFQYYGKDLIEVEKSLTDPIFYGYSFHKLQIIKAKVR
SCTLVCLAFRISCTCSCFIYSVVPFIDRSGQKTLSIPGWFPYDTAKHFYITFFLQSLSLFISAH
CNSATDTLPCKLISLATAQFELLKDNLRTIDYENSFEETKHALVKCITHHRKIVNYTKRVETI
FSKGIFLQLFASVLVICTTGFQLVIVPFGSLKFAIHGIYLCAMTAQIAIYCYYGHDVMITSDEI
GTSLYMSNWYASHIKIRKIMVIFLEKTKKPTIVLAGNFITLSLVTLTQILRSAYSYFAVLRRL
YADD
>TcasOR73FIX
MTRKHIFLNFTVTILKLSFLWPSNDNYDQWRLVKDASLIVSLMPCALPILAHFVLQITGDV
YNMVTITENLIALICIIGMIYMTICFVKNRKLVKTLVKNLPAFTKYSKTTDIILTDKKANLYT
KIFVFYGVIGNVVYMIMPYLNIEKCQQRQNNDVPCGLVTRCWFPFKFDYSPVFEIVFVHQ
FYTCLMVSVIILDLTMLICGFLMHITNQLKHLRGFIKRFDCSSQKIAEDVIYCVKFHTAIITY
SEKTNEAFGTMMMLHITLTSLVISALGFEILIVDNFNDSLRFTLHLLGWLVLLLLICYYGQL
LIDESIAVAEDIYYVPWHLAPVDVQKDIYMILMRSQKPLTLNAANIGVMSFPTFLRVISSAY
SYFTLLLNIKS
>TcasOR76

MMESTVTRLKRMYLWPTASVTSRKPAFFLITFSCFLLYGSVMHLIVNDISMEEVHVIETTA
GQFGVLYYLTLFTIYRKGILEIYADLSNFTKFGKPYNFDKRNKQLNQWSRWFSVVLYFFVI
SVFAWPGIFTQSCEDLNVALNKTEVCGVVSPVWLPFRFDYKPMKQFVYFWQSFCCLYSN
GGAGTISFAMSETIEHLILRVEDLKILFPKIVAERSPEVRRKMLAKWVDYHLWLLSIGKLM
NDTYRYSFSVIVLCAGTLFGCIGYTVMKNASTNFNSSFIFFGWMESVFVICVCGQRLMDA
FHSVGTTVYNSEWCDTDVDFQKGVILITIRAQKPVRIYAGPFSYVSHLLILTVFQTSYSYIN
LLNASS
>TcasOR77
MKYILMKKTIAFLSVTGFWPKTKESTKTRAFCILFSSSFLLFGSLGYLIVYRKFGSDDIDSIE
TATSHFGVLYFMFFWILKRDGLVHIVNLLSDFSKFGEPRFFNDRNRQLDYLLQYCIFVLSVA
TGGVFLCPIIFVKNCEMVKQEKNLTKVCGLVSNVWAPFDYSEYPMKRVVSLWESYCCFIN
FGCGGIMSFTMIKTMEHLHIRVEQLKDMFPDVVNEKNLAVRKQKLEKWVKYHLHLYDIG
ELMNNTYRYCLSVIVLCVGILFGCIGISTMQPGSSHNSLFLFMGWFQSICILCMVGQRLLD
VFLSVGVMAYDSAWYEKDVDFQKAVLMIMIRARRPVLIYAGPFTNLSHLLILGVLQTSYS
YINLLNAK
>TcasOR78
MGHAIMTEILTYLTLMGFWPRSPKSSKASAFLIILSTSFLFFGILFYLIVNRQFGSSEIDSIETI
TSQFGVLYYLILFTWKRNDIVEIVELLSDFSKFGKPPFFDQRSTRLNYRLSCIVLILIVANIVV
AALPVIYIDSCHKANEQLNLTKTCGLIAPVWLPFDYNEYPRKHLVFAWEVYCCVMNYVG
SGIGALTMVGTMEHVIIRIEQLKYIFPKILDQPNPRIREQMLKNWVRYHLALFEIGRLMND
AYKWSLSVIVLCVGALFACIGISMLQSTASQINSICLFFGWFPSIAFLCMWGQRLLDSSLSV
GTAVYSSRWYDMDVAFQKSVLMILIRSQKPIRISVGPFTHLSMLLLLGVFQSAYSYINLLNA
TS
>TcasOR79
MGHVIMNEILTYVTLLGLWPRSRKSTKTISYLIILSSSFLFFGSLLYLVVHRKFGSNEIDSIET
VTSQFAVLYYMTFFTLKREGTVRIIDQMSDFSKFGKPPLFDQHNKRLNYLLSYFVICLFVAI
VGVVALPAIYTGSCHKANEQLNLTKTCGLVAPVWLPFDYNGYPLKFLVFAWEGYCCIITYA
CSGISSLVLVGTMEHLIIRIEQLKLMFPEILNEANRHIREQKLKNWVQYHLALFGIGKLMTA
TYTYCLSVIVLCVGILFGCIGVSTMQSASSNNSVFLFLGWFQSLIVLSVCGQRLIDTCLSVG
IAVYNSRWYDMDVSFQKSVHMILIRSQKPILIYTGPFSYLSHLLILSVLQTAYSYINLLSARG
>TcasOR80
MGHVIMNEILTYLTFLGLWPRSRKSTKTVAYLIISSTSFLFFGSLFYLIAHRKFGSNEIDSIET
VTSQFGILYYWVLFTLKREGTVEIVERLSDFSKFGKPRFFDQRNRRLNYLLSYFVLVLMVA
IGGVVALPVVYIDSCHKANERLNLTKTCGLIAPVWLPFDYNEYPRKNFVFAWEVYCCIMT
YACCGIAALVLVGTMEHLIIRFEQLKLMFPEILDEPDRHTRQQKLKNWIEYHLTLFDIGKL
MTSNYTYCLSVIVLCVGILFGCIGVSTMQSASSHNSVFLFFGWFQSIGVLCIWGQRLLDTC
LSVGIAVYSSRWYDMDVSFQKSVLMILIRSQKPILIYAGPFSYLSHLLILSVFQTAYSYINLL

GAKG

>TcasOR84

MTEEKELRLCLWSCYYLKLSLMWPLKREEFKSSKGLYLRLLVFVIISGSTFTAMIFMHLYK
SLKVGSYDVSEDLAILASNIGYVLMMTMYVSRQKDLELLLLDLSDFKTYGKPPNFDKVR
KRMDLYAHLIFFYSMFGSFVYNMDKIILIDKCKEARRINEVCGSAIPFWTPFETEDLFTLTL
VITYVLINIFVVVKVAMTVSVQVLEISSHINLRIEQLKIFIAGCFDRDFKASRERLDFCIRYH
NVIIDFSERFSRCFSYVMFIHLAITGIIIGCLENQIVQEHQPEAMLHMGGWSTATFIACYGGQ
LLMDASTSIADEFYNCPWYEADVKMRKDLILIILRAQKALFVSTGPFNVLSFALFVSIMKL
SYSIFTVLS

>TcasOR86

MALNQEDAICSKSCFYLRYSFLWPEEAPTRSFYAKFILVLILSFLTAFLPLFIHFLILVERGLD
PSEDLFVIISYTGFALIMIIYVIHVKKTSYLIVQLSDFEKFGKPRGFDYWDKKFRLISSGVYY
YVLIASSGLNLGRWVGMAECRKERDFQVCGIVIPYWLPWKVDSWLFFILLDLYVLKMTL
VVNCALFLIIIQILEITTHLKLRIDHLKEMLVKCFDSDSQTNRKQLVNCIRYHTYIINCSKLF
KKCFTHAMFSLIVTMALSCGCLESQVVKFDLWALPPISAWIFILFIACMAGQILMNASLSIG
DAGYHSKWYQTDANFRKYLILVLMRSHKALVLSAGPFNILCFELFVAIMKFSYSVFMLLN
QN

>TcasOR87

MKHVIMDELLIFLTFLGLWPRTPTSPKIISYLMIYSTSFLFFGSSIYLILHRKFGSDEIDTIEIIT
SQFGVLYYLTLLVVKRDGITKIVNLLSDFSKFGKPPLFDQRSRRLNLLLRLFVTVLLAATVA
IVSVPVVFINSCNKQNLQLNATKICGLAAPVWLPFDYTQNPRKYFVSAMEIYCATMNYAG
SGSGAFLVIGTMEHLVIRIEHLKNMFPEILNEPDKQIREKRLKKWIEYHLSIFEIGELMNETY
KWPLSVIVLCVGILFGCIGVSTMQSVSFQNSSVFLFFGWFQSIFVLCFWGQRLLDSCLSIRK
AVYNSKWHEMDVSFQKSVLMILIRSERPVLIHAGPFSYLSNLLVLGVLQTAYSYINLLNAR
S

>TcasOR88

MTEEKQLRICLSSCFFLKWSFMWPTKSEEFRTSKGLYFRLLAFVIISGLTFTAMIVMHLLKS
VEAGDYDISEDIAILATNTGYILMMLLYIIRQKDLESLLVDLSSFKKYQKPPKFDEVNRKLE
WCTRMVFGYCVFGSVFYNLVKILAIPSCKKSRRINEVCGVAIPYWVWFDTENWSIKLPLIL
HTFLVIIIVDKVTLLVSLQVLEIACNIKLRLDQLNCMLVSCFDGDVEASRRRLNECIKYHKE
IISYSEIFSKCFSIEMFTHLTTTGIICGCLENQVVQEHRPEAILHIGGWITAIFVSSFGGQILIDS
SLSVAEAAYSSAWYEADVSLRKDLILVILRAQKALFVSTGPFNVLSFALFVSIMKMSYSILTI
LQ

>TcasOR89

MKEAVLQQSKKEMHLLNLWPKGHVKHFRFRYVITLIIVSPFTLGTLTHFINVLKENLDVDL
SGDISVIAVVTGLHFMLITFVWGHKKIAYLWENLGPHEYFGKPDNFEKRCKQLNFYSRLYA
YYCYLGLTVYIIMKNRGGIECRRLNVERNLTEICGLVTTFWAPFDIDFFPFRQILFVDQVFAT

YFIVKGGAAISFTTLEVGEYIILKIKHLKRLVKEVFDDPREEVQRKKLVFCIKYHQYIISIQE
LYDGRYKHCNGCYILMVGIIIASLSNEIMKNHNIEALLHLVGWVFSFYICCFSGQSLLSESL
TIPDAAFESKWYEAPVYMQKDLLLMMLRSQKPLMLHATPIGVMSLSLFITLVKTSYSYFT
LLNQST
>TcasOR90
MAKDTSPVLRESIEVMKYLQLWPQNERTNLRRRYFIVIFLCSPLHLGLATHLVVCLKDNLD
VDLSANIAVLSAVTGLTYMLIVFVWSQDKLVHLLAKLDTHEIFGTPDNLTKRSRRLNFYAK
LYSYYCYFGIVIYSLVQIIEMPQCRKMNEEKGLSEICGMIVPFWAPFDIDWFPLKQIFWLNQ
LLGIYIIIKGGAAVSITTFEVAQYICLKIKHLNRLLREAFDDPCDVVVEQKLLHCIRYQQHIIR
TNELFNVCFKHCNGCYVVMVGIIIASLLNQILKEKSVGALVHFAGWICSFFICCHAGQAVIS
ESLTIPEAALDSHWYEAPVKYKKVLLLLLVRSQKAFNLQATPIGIMSFDLFIALLKTSYSYF
TLLHKST
>TcasOR92
MKNQEIKICRATLTVLKYSLIWPSEADEMNPGKWYYIRVVTFILFTCPWVLSVFMHLIVSIR
NNADIHLSEDVALMVAFTGVYYMTIIYVKKQPKVAFLLRDLSYFQFGKPPGFDETERILGF
LSKLTFCYSVMAVVIYNYIKYRQKPECERMNKLKGLKENCGMLTPTWWPFEINYSPAFQL
IFLYIFTSTQVMMKLSLMISFNVLEMAHHIILRINHLKTMILESLDEQDYEASKRKIKTCILY
HLEILGFAERMDDCFSNGMFAHLTITAAICGCLEKQFVDGDNQLGSLLHIFGWILALFLAC
LGGQHLINASETISDAIWSSKWYDADLRLRKDLIFMMARSQVGLYLNVGGFGILSYALFLS
VIKMSYSILAMLTS
>TcasOR93
MTNLEIKICRATLKILKYSLIWPNEADEMNPGKWYYIRVATFLLITSLWVLSVFMHIVMSII
HDADVHLSEEVAFCVAFCGLYYMTMIYVKNQPKVALLLRDLSKFQFGKPPGFEEKERILG
FLSQFFFYYCVMAVMVYNLVKLLQKPDCEKMNEIKGLKENCGLLTPTWLPFDINYFPAFH
LTFLYVFISTQILMKLALIISFNALEMAYHVILRIDHLKIMITECLDQRNYEVSRRKLKTCILY
HLEILSLSNRLNDCFSNIMFAHLTITAAICGCLEKQFVDGDNRLGALLHVCGWISALFVACI
GGQHLLNASLSIPDAIWSSKWYEADVRIRKDLLFMMAKSQVGLHLNVGSFGVLSFSVFFS
VLKMSYSILAMLTS
>TcasOR94
MAIKICKFTRKNMQISLIWPREFEEINPGKWYYIRIVIFLITYGVFPFCTFLHAVVVIHNNLDI
RISEDIGAVVSNIGISYMAIIYVQQQNQIAYLLKDLSDFKDFGKPPFFEEENKRLNFWSICTF
IYPTCGASLYNLSKILEKSECNKINEENGLPATCGFIFPIWVPFNINYFPLFHIMLISTWFCTT
MFVRLHLSISYNAFEIAHHIILRIKHLNGMIITCFDCQDYKISRQKFTTCVLYYKQILDLSNR
LNQSFSSIMFVHFTMTSAVCGCLEKQFVDGEYVGGFIHLVGWIISLFIASVGGQDLVNASQ
SISEAIWSSKWYLADIRLKKDVLFMLMRSQKDLHMSVGSFGVLSYAFFVSVLKMSYSILA
MLTS
>TcasOR95FIX

MVVKESEIKVSRVTRKILQYSLIWPKEGDEINPGKWYYIRIFTFLSFTSLWCIAICMHFIIVL
KDKIDWDVTEEIAIIIAIYGTYYMVLAYVKNQKKAARILRDLSNFERFGVPPGFEEEEKRL
KVYIIGIFIYAFLTITFYNFFKLSQKGACERFNEEHHLDENCGLLSPVWIPFKVDRFPQFELV
FLYLFTCCHLLMKLPLVVSYNALEMVHHIILRINHLKIMITECFDEPEYEISRRKLTQCILYH
IEILEFATRVDDCFSNCMFAHLTLTGAICACLEKQIVAGISRFGAILHFIGWILALFIGCLGGQ
HFINASDTIPESIWASKWYNANLRLRKDLLLMMMRSQRDLHITAGPFGVVSYALFLSVLK
MSYSILCVLTS
>TcasOR97
MNNQKIQISNMTRKVLRYSLLWPKTNEELNPGIEYQFSVLGFFLVTGVLVLCITIRFFITIKA
VHEVDAEVLAILIASYGSYYMICAHLKNQHKVALLMRDLSVFNNFGKPPNFDKRNNQLN
FVAKLLALYSFLATIFYNGEQLINKTECKRINKEKGLSDHYCGLLAPCWLPFEIDYFPVFHL
ILIYAFTSGYLLIKMAIHISYNAFEIVSNIVLRIEHLKAMILETFENRNKQVCHKKFLQCILYH
IEILDFAARLDDSFFNSMFGHLALTGGICACLEKQIVSGVNVVAGTLHFIGWILALFIGCVA
GQYLINASEILPSAIWTAKWYDADLELKKKVLFMLARSQKSLFIRAGPFGILCYPLFVTVL
KTSYSILCMLTS
>TcasOR98FIX
MVKKESEIKISRVTRKLLQYSLLWPTEGEELNPGKWFYFRIFAFLSFTSLWCIAICMHFIFV
MKDKPDWDPTEEIAIIIAIYGTYYIVLAYVKNQRKAAGILRDLSNFDKFGVPPGFEEEEQRL
RVYIICVFIYGFITITFYNFYKMSQKKSCERFNIEHNLHENCGLLSPVWIPFRIDKFPRYELVF
LYLLTCCHLLMKLPLIVSYNALEMVHHIILRINHLKIMITECFDDPDYEISRRKLTQCILYHT
EILEFATRVDDCFSNCMFAHLTLTGTICACLEKQIVAGFSRFGAILHFFGWILALFIACLGGQ
QFINASDTIPEALWASKWYNADLRLRGDLLLMMMRSQRDLHITAGPFGVVSYALFVSVLK
ASYSILCVLTS
>TcasOR100
MSPKDKIKICGITRKVLRYSLLWPVENDELSPGIRYKLTILAFFSITGILVFSISVYSVLEIKQG
YDIDVEDVAILIAVYGTYYMVSAYLNNQHQIALLERDLSQFYKFGKPPGFEQLNSQLNFAV
KVLIIYSFLGTFVYNGTKMLLREECKKNSQEKGLSDNHCGLIATFMFPFRVDYFPVFYIVL
VITFLLAHTLIKLCMHISFNAYEIVNHIVLRIEHLKEMILSCFNERNQTIVQKKLRVCILYHIE
ILDMAARLDKNFFNTMFGHFALTGAICACLEKQIVLGVNIVAGTLHFIGWIIALFVGCVAG
QCLLNASEIIPNALWAAKWYHADLRTQKTLLFMLARSQKELTIKAGPFGILCFPLFVSVLK
TSYSILCMLTS
>TcasOR102
MQNQSKPCQLDMMDETYLQFFVKSFTYLNMLPEKTTFCTTIQQYYVSVIITITTFPILADL
VSQFYEESISFTSVNENFVALSALFAVIYVSVCFINRKHKIRALIADLALFETFSSKAVITETD
KSVKFYTKLFIVYGIVGNLCYGLLPILGYKKCHESKSVHMTRYGIPCGLVVRFLFPFKFDY
SPLAELVALYEILVCILGTSVVIVVTTLICGVLIHITVQLQCLRKIILDLSQVNDLEILEHKMK
FCVKYHTAILDYGIRTDLAFNQMMLLHITWTGFIISVLGFEISTTDDYVEAFRFFMHLLGW

LGMLFVVCYYGQKILDESLAIADAVYTFLWYKKSVIVQRYVLLILLRSQKPLTLRACGVK
VMSLATFLGVLYSAYSYFTLLLKLKP
>TcasOR103
MKQALKLADVLGFNPLKNDNLTKLKKYSSLICMISVVVSAILEFVSNFSALETYESAPESL
VPQFQTLAKISSLLLSQKDITELIDEIKYFWKLDQFGDFHTRKLKKIYKYVTIFFYFYTLML
SGACVLFTITTVIFTPEKPLFLCYGGLHGLPSPQFEIYFVVDLAAIVIMSFGVAAYDGIFFYF
AFHVYAEFKLVKVAFKGKSTFIEAVKHHDFLLKYLRKLNEIYSPIFLCQFFSNLLGICFCLF
MLSRSGMPPELTSFSKYFISLVAFTVQTYIFCLIGDLVSELSLDISNVIFYVDWLDDEVYKSK
TARLVIMNKAQSPVKLTIGKFTGMDLRTFLLIVRNAYSFLAFVNNALD
>TcasOR105
MKPALKLANVLGLDPLRNDNYTQLKKMFCALCIVSLFVSAYLEFFSNFTTFETYETAPESL
IPHFQTMFKMYSLIFSRTEIVELIQMAEQFYKFSQCDERKKLTKLYKRVDLFFYVYASLVAA
ACVLFAIVTLIFKPGKPIFLCYGGLHGLESPEFEIYLVVDLIGIVIISVTVPAFDGLFFYFALYI
YTEFKLLKIAFKTMSGQELREAVKHHDFLLKYIKKLNSVYSPIFLYQFFCNLLAICFCLFML
SRSGIPPEMVSFSKYFLCLLAFLVQSYTFCSIGDLITELSEDVSNAIFYTDWLDDEAYENKT
ARLIIMSRAQNPVMLTIGKFANMNLRTFILIVRNAYSFLAFVNHALN
>TcasOR106
MESALKLIDIIGLHPLKSDKYSTMRKTISFLSLVVILISAQLEFLSHLSVFEVYNSGPHSTIPP
LQSLLKMATLHFYKNELIDLMEKSKSFWKLDKFGDLYKQELSKLHRLVTIIVYIYIALLTAT
CVQLAVLTLIFRRGKPIFLCYGGLYGLESPHYEIYSILDAIGIGVISIAVSGYDAMFFFFALDI
YTEFKMIKSAFKRHSDQTVSSYNKQFIEAVKHHDFLLQYINQVNDIFSPMFLFQFFSGLLGI
CFSLFMISRSGLQDINTLSIYSAGLLGFTAQSYTFCLVGEVISELSEDISNEIFYTDWLDDEV
YRNKTAILIVMNRAQESPKLTIGKFADMNLRTFIMIVRNAYSFLAFINNALD
>TcasOR107
MENPLKLLHIIGLDPRQSDKYSTIKKVISFLIVLAVLLSALIEFFLHHNESQVYDTAPQSTVP
NLQALLKMFALIIYKKELIDLFTKGNHFWKLDKFGDCHKQKLTKLHKYVDLFFYVYAVIIT
GAFLQLALLILIFEPGKPIFLCYGGLYGLESPQFEFYAVLDFLAIGVIAISVTAYDSIFFYFALY
IYTEFKMIKIAFKRENCAQFIEAVKHHDFLLQYISKVNEVFSVIFLTQFFSGLLGICFNLFMIS
TQGTRDMKSFSTYFVGLVGYTAQSFTFCLIGELISELSEDISNEIFYTDWLDDEVYRNTTAR
LIVMNRAQESPKLTIGKFADMNLRTFIIILRNAYSFLAFINEVLD
>TcasOR108
MGSILLLNSVLKKMEKALKLVNILGLDPRKNDTFSKFRSIFCFTILISASFSSHLEFFLNFKG
LETCERAAESIIPQYQTMCKMATFLLYKTEMLDLIKKSERFWKLDRFGDLQAKNLHSTYPI
FQIFFYVYVVILFLTCAMFALVNWIFDTGKPISLCYGESEGLETPWVEFYIVLQSVEVTIIFL
GITGYDMVFLYYAGSVCIQFQMLKMAFAERKMNERQFLKAVKHHEFLLQYVEQLGDIYS
MWFLLQYFSSLFGICFGLFLISKEGLPTEPERLSKYFPYIFSFTMQSFTFCMTGTMLSDWSS
EISDEIFHSDWSDDQVYKNKTARLIVMNRAQRPAKISIGKFLDLNLRSFILLMRSVFSFLAF

VNNILNRIN

>TcasOR109

MGKVKFTEPLEFLNVVGLNPENCSNFSLFRRVISLGFFLVVITLGLLELLLHFEGLETCSRA
SEAMIVQYQLFIKIAVLLKHRKNLVVLMQKTRKFWPLDKFGQDAKIERPHKLLKAFFFAY
KLIMILMALQYILRKFVSKNGKPLAIAFGESKGLSPKVDHLYFVLHSTSTFVVLHAVTGFD
RLFFFLIGHVLTELKLVKKSYRLTQNRREKFLETVQHHAFALEFVRKLNRIYSQVLLNQHL
SCLFGICFGLFLVSKDGIPPDLGHVTKYVPYVISFITQTFTFCFIGSLLITWSLQVPDAIFYND
WGKNQAYKYKTDKIIAMIRGQRAAKLTLGGFGDLDLESFNLVVKNAFSFFTFVNAMNQK

>TcasOR110

MDKVEFSDPLFFLNVIGMHPFKADKFSKFRLAFSIAVYFAVIFSGVLELIVNSQGLETYARA
SDTLIPQCQLVCKIFVLAKYKKQIARLLNGSQRFWDLGQFGARYGNSFGKTHKYLKSFFL
LYKVMLTFTCLQFLAVKIIFKIPKPIAISFGETKGLEPLYDHLYLVLHAMITLVTINLVNGFDG
LFFYFIGHVLTELKMVKVAFGDSPIETNWSEEKRFKFAVRHHRFVLDFIEQFNIVYCTMLLV
QHLTCLFGICFGVFLMTKDGVPPDLDRASKYLPYIVTFIFQTFTFCFAGNLLLSWSLEIPNEI
FYHDWAKKTTYENKLAKIISMKRGQRAARLTLGGFANLDLDSFRMVLKNALSFFTFVNA
MMNKKAVTSV

>TcasOR111

MEKVRLTEPLFLLHIVGMSPHDSGTFARIRKIFSILVYTSTVVLSMAELFFNYKDLETVIRAT
ESFFTQYGLAWKIAVFVVYKTELAQIIRLCDNLWPLDEFGTGHNFQFLHKFLRRFFLLYTG
NLALLCTQFAVTAFFDDQFKSVMVYYGEKESRSQIYDNFVFTLQVIYLYVGCFVVAGFDC
FFFYLLGHAVTELKMLTISFSCKEIGRNWGYEERFKCSVKHHIHVLELLDKINKVYSVMLL
NQHLCSLFGICFGIFLMTKDGIPPNVDHFSKWSTYIFTFILQVWTYCFAGDQIMHWSLKIPD
EIFYDNYWNKYSLKNGLNKIIAIQRGQKAAGVSLGGFAMLDIESFNVVIKNAVNFFMFMD
KMYKRE

>TcasOR112

MITRLMAQFAIKGRVGTGGYIMDKVKLAQPLAHLNIIGLDPLKNDRFSKIRTVITVAVFALC
NVFSFSELFLHYNNPHVIVRSSEVVFPFFQNDWKIAIMLVYKKNLAQLIQNTSRFWQIDAF
GKNYQYSMGIKHKYVRIFYLVYRLMLMFSCSQYILLTIGSDRPMILSFGETGGLGSGALLF
YLIFHIVYLLIIFNVINGFDGLFFFLVAHVLSELQMVKVAFSSSKVITFWNHKRRFKSAIQHH
RFVLDYINRLNSIYSILLLNQHISCLFGICFGLYLFISDGFPPDYEHISKYVPYVIYYITQVWV
FCFAGQLIIDWSVNISDEIFYHDWTLNRTYENKTDKLIIIQRAQHAARLSLAGYGNLDLQSF
NLVLKNGLSFFTFVNAVIHK

>TcasOR159

MRGKTIESTTNPYSSLKKVFIDFAYSKLVISYTKASLTFHVLSLLLEVYYLVTNFSVELICRY
GCMMCLMTYMYSKKLKLLEKPCLLDFWKVYNSSTATQRLISEKSSKTNRRLYCALTCCFF
LAIILFPIWGDLNEFFIFSQVYEKYFTSWAPAFCYFYVSTLLWCCFYCFHLPGIIMYLTLHLD
LQFKLIKDKITEIDKNCSQKEIYQILRLCISHHVALKKWMDKLADLLVTIMPFFFLFGALNSI

ATSFFVLYTLQNTTMILKIRLGTLTLCNFIIVSTFAEVGQIFSGQNNSLFEQLMDCSWYLWNI
KNRKTLLMFMLNCMKPKTFSWGGITLNYSFVLFILKTSLSYASVLFKLRGETF
>TcasOR160
MSGKTKRITTKTIHLSNPYSSFKKVFSDFAYSKIMIFYTIATLAFHMLSLFLQIYYVATNYSV
ELICRYGPMMCLAIYVVTAKVVGVFYYKTFTMLENQCLFVLWKTCNSSPTTQRLILNKSL
KMNQKLHLALMSYFLLAIVMLPTWGDLNELFIFSQVYERYFKFWAPVLYYFYISTFLWCS
YYSFHLPGCILYLTLLLDVQIKLINDKITEIDQNFSQNEISETLRLCISHHIALKRWMSTLAK
MVNSVMPVFVLLGALSTVAVSFFVLNTLQNTTMILKIRLAILTVCNFVIVSTFAELGQIFSD
QNNSLFEHLIDCPWYLWNVKNRKILLMFMANCMKPKTFSWGGITLDYSFAISILKTSFSYA
LILFKLRGETIRN
>TcasOR164
MSGKTKRTTTTRKINLANPYSSLKKVFIDFAYSKIMMFYTKATLAFHVLSLLLELYYVATN
FSVDLICRYGCMICLMTYVVTAKVVGIMFSKPFKLLEKQCLFVFWKTYNSGPTTQRLILD
DSLKMNRKLYLALMFYLLLAIVLLPVWGDLNEIFIFNQVYETYFKFWAPVLYYFYISTFLW
CCYYSFHLPGSIFYLTLHLDLQIRLINDKITEIDQNFCQNEISETLRMCISHHIALKSWMSKL
AKLVDAVMPVFVLLGALSTVAVSFFVLNTLENTSLILKIRLTTLTVCNVFIVSTFAELGQIFS
NQNNTVFEHLMNCPWYLWNITNRKTLLMFMLNCMKPKTFSWGGITLDYRFALTILKTSF
SYALVLYQLRGETN
>TcasOR165
MSDNTKKATTKSLDLTNPYSSLKKVFINFAYSKIMIVYTSATLIFHILSLMLEIYYLATNFSV
ELICRYGCMMCLITYMVTAKFFGMLFSNQFKFLEEQCLLDFWKAFNSGPTTQRLILKESSK
MNRKIHLALTFYVILAIIMLPIWEDVNDFFMFSQVYENYFANWAPVLYYFYISTFVWCSYY
SFHFAGVIMYLTLLLDLQFRLINDKITEIDQNSTQNEICGTLRLCISHHIALKRWMNKLANS
VDTAMPVFILLGALSTIAVSFFVLNTLQSTSVILKIRLATITVCNLIVVATFAELGQIFSDQNN
SLLEHLMDSPWYLWDVENRKTLLMFMANCMKPKTFSWGGITLDYSFALSIFKTSFSYALV
LYQLRGNTF
>TcasOR167
MAKTGDIFPVRDPVKRCLFIPKLLLESTNFWPEKRNFLTKFANWVMLIICVLIESGQIAFVV
VNIKDITKIASAMSTVSTTFQAITKLTVLYIYNDKLRLILKSVWYEFWPSYTAGREINTKLE
TYNKIVIVSFLTILISGICFAFGFLSSPLISGERILPFETVYPFDWTKSPYYEIIYVTEWMTNIA
FILIGICGHDFLFMGLCSNVVGQFTLLRELFGYLGTKNVAQIIKKLGHDTNIEPNRQLLRICI
IHHVRVTEICKEIAEIFSFSCFIQLLSSVTALCVGALIMTFADIDAALFTVSSAYIVGHLLQLF
LYATLGNEVIYYASRLPNAIFHSHWYNIDLEVKKDILFVLQRAQKEVKISAMGVSVLDYQT
FIQVLRLSFSFYTMLSKVTDH
>TcasOR171
MVKLFLLLKHLTMKAQDSDNPYIVLRRVFVDFAFTSHMIIYTKITFVFHFLTLLLETYYMIT
NFNVELFSRYGCMMCLMTYSNVQIVLAKLLEILFARHIKFLEEERLSHFWKLEESSEETQK

VVNAESSKIRKKTFFVLSWFVALGFVLFPIFGDLNDLFMFGRVYRNYFGSWAIIPFCIYVST
FPSIAYNSICLPAVVSYFIFHLNLQISLINDKLGKISEKSRQSEIYQKLCSCVAHHVRLRRWT
NIFQNELESALPFYLFLGAINSIAVSFFILYNLQNMTLIFEIRLVVISVCNVLILWIFAEAGQEF
SDNSDSIFDAVVACPWYSWNAQNRKIMLIFMLNCLKPMTFSWGGVKLDYQFTVTIVKMS
YSYALVLYNWRYEK
>TcasOR172
MSFQALKHLLKMCAEKTPDLDNPYLTLRRVFIDFPYSKSMKIHTCITLLFHFLSLILEIHYL
VTNFSFELSSRYGCMMCLMTYVISVKIFVIMFAKPLKILEEQRELHFWKIGDSSHAMQQSV
ATEALQVKKQTYFALSCFVLLAVILYPVWGHVNDLFMFSQVYEKYFGDWSVIPYYFYVFT
FMSSSFNSFQLPGVILYFTLHLNLQISLINEKITKISGENYCQDEVFKQLRDCISYHVALERW
MARLIDLTKTAMPVFILLGALSSIAVSFFVLYSLENTRFILKIRLTVVAICNVLIVATFAKAGQ
RFSDKTGLIFDAIATCPWYSWNVPNRKIVLIFMANCLKPKTFSWAGITLNYQFAIKIVRTSC
SYALVLYKLRNGNY
>TcasOR187
MSTKREVVKNFPYYYLFKICIDFGYSNVVKRLNICCITMIVMFHLTQIHYMQENFSKELIL
KYGSGIALGIYTILSMSVQMLIEHEIKDLIAEALFSMWAVDSCGPQVEKLILRRAKVMNIIY
CSIFAWFALMATVMLPMWGDHSEWLLYDPILVEDVKTRLKIIYYLSTFIIFPMIAFSAIRLPG
ILLYGILQIHMQIMLINHKLVQVSEDLDDLNNVKKIDQDDYQERIYKELCLCVEHHIKIKL
WLNKLMKIVQLLMPPYFLLGSINAIYLLFFVVYNDTSNILKVRLCILLIVGGQILCMFAEAG
QALGEETGRIFDTLVNCPWYLWNKKNKQALTIFLSNSFQPYTIAFAGFTLNYSLALALLRS
SVSYALVLYNMRN
>TcasOR188
MFVKRQVLEGFPYYYLLQLCLDVGYSKMMKIANIFCIIINLLNVLAQIGYIKQNFGKELLL
RYACGIQLTIYTIVTMLFEFLVEQNVKKLMDEALSEMWPIDFCGLEIKKLILKRSTVMNSIF
YFMFAWFAILAIVMLPMWGDQSEWLLYDRICKEFFATWWKIPYYFYFTTFPVVAFSGIRLP
GLLLYTILQTHMQIILINQKLVQISGGLDGINDVRMIDQKNYQKRIYKGLRLCVAHHVAIKR
WLQKPVKIVQSLMPIYIIMGSTIFISLLFATVYSFRDSSNILKVRMSVVLMICCLILCMGAEA
GQALSNETSRVFDTLVNCPWHLWDQKNKKALTIFLPNTLQPVTITLAGITLNYSFAVGLLK
SSASYALVLYNMRN
>TcasOR189
MEKMFPQIRTEDMKKFPYYYLLKICIVFGYSKIVKLLNVVCIIITSSTIVLQVYYLKQNFSK
ELILKYGCGISLTIYTIASMLVEFLIEQKTKKLLNEAGTILWPVNFCGVKVEKLILKRVTVM
NIIYYFMSAWFALMGIIMLPIWGDHSEWLLCDVISNEYFETRWKILYFACSCFSFPVIAFSSI
RLPVILLCTILQTHMQIILINQKLNQISEQMGNLNNIKLVDDKCYQKRIFEDLRLCVSHHGK
IKKWLNKVLKLVQSIMPLYIILGCLNFISLLFFASDGLQNASNILKARLCVVLIVCCLVLSMF
AEAGQALSDETSGVFDTLLTCPWYLWDKNNKKVLSIFLSNSFQPDSISVAGITLNYDFAVA
LLKTSSSYALVLYNMKN

>TcasOR190
MSTKKQDLLKHFPYYYLWKVFINFGYSKLTKLVTISCIIIHSSSLFVEIYYIYCNYNKEIIFKY
GCMMSLLGYITISMVVELLLEKDTNNLVCEARSLFWTIDSCGVQAQQIIHKRAVVMNATF
GFILMWVATLGVIMFPIWGDQSEWVLCVKIFENYFENWSQMANFVFFSTFPMVAYSTIRLP
AMLLYGILQTHMQIFLINQKITEISRSKDQEKIYKELCLCVSHHVEIKRWLQRFLKMVQLT
MLMLIPLGVLSCVCVLFFVIYSFLDTSNILKMRLTVVVACTVLIVYIFAEAGQDFSDEISCIF
DTLVTCPWYFWDQKNKKALVLFLANSLKPYTLSIAKITLNYDFAVALVRTSVSYALVLYN
MKN
>TcasOR191
MRLEIEALKNFPYYYLLKICIDFGYSKIVKCINVVCIIINSSTLFIQVYYVQQHFNKELIFKY
GCGMALTIYTIASISVEFLIEKNAKNLVNDATAFVWPVDFCGEKVKKLILKRATVMNKICY
FMSAWFALMGIIMLPVWGDHSEWLLCDLLSKEYFETRWKILYFACSCFSFPVVAFSSIRIPG
ILLCTILQTHMQIILINQKLNQISEQMGNLNNIKLVDDKCYQKRIFEDLRLCVSHHGKIKKW
LNKFLKLVQSIMPLYIILGCLNFISLLFFASDGLQNASNILKARLCVVLIVCCLVLSMFAEAG
QALSDETSGVFDTLLTCPWYLWDKNNKKVLSIFLSNSFQPDSISVAGITLNYDFAVALLKTS
SSYALVLYNMKN
>TcasOR192
MVSEQTLLKNFPYYYLLRIFIDFGYLKITKVLSVACIIIHSLSTLLEIFYICQNFSKELVFQYG
CITSLATYVITSMTTGFIIENDAKNLIRETVTAFWPIDFCGPQVEQLIFKRVARINTFNFFLLA
WFAIFGIIMFPVWGDESEWMLCVIAFKKYFPKWWRVPYYVFFATYPMVAYSAIRIPAMLL
YGILQINMQFFLISQKIIQISQKPQNKTHQPGFYQKTVYKKLCQCISQHAEIKRWLQRFLK
MVKSVMPVFIFVGGLCFMSILFFVVYTFQSTSNILKVRLGVILMICNLILVTFAQAGQTVID
ESSGIFDTLMTCPWYLWDEKNKKTLVIFFSNSLKPITFSIASITLNYAFAVALLKTSASYAIFL
YNIKN
>TcasOR193
MSELEKQLPYYFLMQFCINFFYSKTVKVVTSSCIIIQSLSLLLQVYFIITNFSKELILKYGCE
MSLATYLLTSLLVDVVVENTTKQLISEGHTSFWSIDSCGHDVKNHIIANSARLSVVIYFILA
WFAVLGISVLPVWGDQSEWILFVQIFNTWKKILCYVYLSTLAVMVFLSIRLPAMLLYGILQI
HVQIILINQRIIQIGRENTNDIRMMNQMSYQNRIYKELGFCVSQHARIKRWLKKLLGIVQS
AMPIFTVLGGLIFISVLLFVLYSFENASCFLKIRLGMVVISCSLVLCMFAVAGQAFSDETSRV
FDTLMTCPWYLWDQKNKTILLIFLSNSLQPINFSIANITLNYSFAVALLKTSTSYALILYNMK
N
>TcasOR194FIX
MAMKQYPFLYKIFLDFAYAKIGKMVTYSCIIIQSLALQLQVYFIVTHFSKELIVKYGPGVLV
VTYLVTSLVVELMIENKTRKIIDFARLTFWPTDFCGLEAKNRLIKNSSKVSIVIYLILMWFA
AQGIVMFPVWGDTSEWRLHVEIFDQWKLFYYIYVSTFTIIVFSAVRLPGILLYSIFQTHMQI
VLINQKITQISQNDPNDIRMMNQTGYQKRIYKEMCLCVSQHIAIKRFIKKLLEIVRPVQPIF

MVLGLLGVISIFFFALYNLENTSNILKIRLVMVVISCILILCLFAEAGQAVSDETSRVFDTLLT
CPWYLWDQRNKKALAIFLSNSLQPISFSMAGFTLNYGFGISMLRNSASYALILYKMKN
>TcasOR195
MFRERVYDDRFIVLKTIFLEFAYCKEMKIYNMFCLVFHLFSFSLQVHFIVLNFSVELITRYG
CMLTVFLYLIAAKSFSIIIEKQVRMLEMEATSFFWPIDCCGPQVKKNIYDRAARQNIQNYFT
LAWFALFGIIMLPVWGDQSEWFLCIQVFQQYFGCWKLFYYFYFSTFPMIAFTAFRLPALML
YGILHEHLQLILVNQKIVQLSVRRSLKENIVDNANYQKTVLKKLKLCISHHVKLRDSLGKL
IGVIQLAMPVFLFIGALGSIAVLYFVLYIFLSSSNILKIRLVVITICNGLIVYTFSAAGQALADE
TGRVFDTLMTCPWNTWNIKNRKVLLIVMSNTIQPLTFTLAGITLDYKFGLTMLRISCSYALI
LYNLH
>TcasOR197
MFKKRKFDDRFIVFKKIFFEFAYSKEMKIYNMICLVFHSFSFVLQVYFIVQNFSVELITRYG
CILAVFLYLIAAMSFAIFIEKQVKMLEVETTSFFWPIDCCGPQVKKLIYDRSARINILNYFTL
AWFTLFGIIMLPVWGDQSEWFLCIQVFQQYFGSCWKLFYYFYFSTCPMIAFTAFRLPGLM
LYGILHIDLQLVLIYQKIAQLSARRIFSENIVDNAHYQKTVFRKLKLCISHHVKLKTCLRKLI
ELIQMAMPVFIFVGAVCSIAVLFFLLYVFSSSSHILKIRLAISVVSNVLIVYTFSAAGQAIADE
TSHVFDTLMTCPWNAWNNKNRKVLLIIMSNTLRPLTFTLAGITLNYKFGLTMIRISYTYALI
LYNLN
>TcasOR198
MPNVTNKRQKRLFSKTRTKSEDPFVMIKDVFVDGGYHPVTKMLNYICLVIHSCSLLLELN
YFVHNYHFDLMMKYCCAMSLMGYIIATMLFAIFQEHSAIDLTKDILSLFWPIDYCGPRVKE
EIVKKATKINRIHYIVLLFAGALGITMFPIWGDQKEWFLCVQVYQHYFGKWSKIPYYVYFF
TYPMLAFSSVRLPFMTMYAIVQIRMQVYLLHQHISEISGEYVYDMKNLQILCDQNYQNEI
YDKMRLIISHHIMLKRWMRKLVHTVQISMPVFVLLGTMTSISVLFYAIYSFHNINFILKVRL
ISVSVCTVLVVYMFSEAGQALSTETTGVFDLLMTCPWYVWNIKNRRILLIFMANSLEPMT
FSLAGVTLDYRFALGMLRTSCSYSLILYKLKTGI
>TcasOR204FIX
MTNFFSNFCSPLKNHWAKTKHLFSKFSLSSDQPFIMIKLVCVDIGYHPVAKTINYICLAIHIS
SFLLEMNYLRLNFSTDLLIKYGCGISAVVYDISTLIVAPMIERPTIGLSEGITTSFWPIDFCGP
KVKQLILEDTKKTSKIYYRTLVTIFGFAAVIMLPIWGDQKEWFLCVQVYEHYFGKWAQIPY
HIYFLSFMWFAFTSVRLPLMMSYAIKNIRVQVFLVNQKIAKMSKEYEEAKIEDVNYQNRV
YKNLRLCISHHVLLKWWLRKLQKIVRFCLPVFVVIGILTESSVVFYLIYNFKKVNLLLKIRF
LLLACTTGVIIYFFSEAGQSLYIETSQVFDSLISCPWYSWNVKNRKVLLIFLTNSLQPMFFSL
VGFTIDYRFALTMIRTSFSYAIILYNLSSGSQIASI
>TcasOR205
MTNIFSNFSLYFKNTWTKTKQRFSKTLPSSNVPFMMIKLVFVDIGYHPVSKIINYICLAIYM
SSFLLEMNFLRLRFSTHLLIKYGCGSSLSVYFISSMTVAAMTELLAVDLSEGILSSFWPIDFC

GPQVKQLILKQSRADKRMHYVVLLVFSITGLAMLPIWGDQKEWFLCVQVYEYNFGEWS
KIPYYIYFFTFPWVAFSSLRLPFMMNYAILNLRMQVFLINQKIAKMSNAYDQTTIEDVNSQ
KRIFKNLRLCISHHILIKWWLRKFVNHVKFCIPIFVIVGIATSISIVFYLIYSFQQVNLVLKIRF
LSIACCCWFVIYLFSEAGQSLYEYTEIFHSLISCRWYIWNVKNRRILLVFLANSLEPMTFSLA
GITLNYRFALNMMKTSCSYALILYKLNCDSQIMD
>TcasOR213
MAKFNDPFKFVRTIIFVDMNSYKVIKTCNVLLNIIYSLIHCLLIYYLCKNLEINLLIRYAPAIL
LFILVIFGAVFSIYMDEDILEVRSVFRENRWSLSVLKENSQTKLGRKCQFINIFILLVLLLIVS
TLAINAPCFGNQRELLICIQVFEEYFGEWSFIPYYFFFLGFPLLYYNFFRLWMTFVYGLLEG
QLQFFILEEYLCGIYETEDSKSWKYLQDSRYQQEIEKSLRLCISHHIGLKKFLKMVENQTL
KVMPFYLVFGVLILICYFSFIINFADTVTTIGKIRMFMTAICMMGVAILLSWIGQQLIDVTSD
IYFTLGGAPWYYWSQKNAKLLLMFLTNCTKNESVTLAGISLDFTLFVSIVHTTLSYALVLY
NLRESSLVSSSQK
>TcasOR229
MSARPLHLRNFPYYFLKVLVFDFEQYSAGKVLSYFCAIVHSISIFLQMHYLVKNFTKETMF
QYGCVLTVLTYCVVALFFAIASGNFVEKLESEISSFVWPLDICGEDVKAAILKRAFYTSLVA
YITIIAFPIFSVIMFPVLGDQSDMFLCVRVFNEYFTKWSQIPISLYFYSFPVIAFSGIRLPGMLL
YAILITHIQMFLLNRRIEQISELSNQRRVFETLCSCIELQAKLKRLIRNVFQLVYIAMPIFILLG
AVSSVFVLFFVVNSLETASYFLVLRMGCFFGANVLVVFIFSQSGQSFSDETGRIFDTLVMCS
WYNWDKRNKKVLLMFLANSLEPMSITIAGITLDYKFALAMLRTSCSYALVLYQMKN
>TcasOR230
MREKPLHLSHFPYYLLKIMLCDTEQYRLGRFLSYSCAVIHSISLLLQMYYLIDNFNKETVS
RYGCVVIVTTYCVVALIYEILYAQPSVSMMSQQISTLWPMDACGEKVKQMILKRAFFTSV
VTYSILFSFPIFGIIMFPLWGDQSDMFLCVRVFNEYFTKWSKVPIYLYFCSFPVLTFSGIRLPG
MLLYAILITNIQIILLNQKIAHISDLGDQRLVFGTLCSCVSLQIKLRQMLNKVLQFVYLVMP
VFLLLGALTAISVLFFLFYSLENPSDYLMIRLACFLGGNILVVFTFCESGQALSNDTGRIFDI
LLTCPWYKWDKKNKNILLMFLVNSLKPMSITIAGITLDYKLAVTLIRTCCSYALVLYQMKN
>TcasOR234
MQQAALRNFPWHYIKRIFIDFGYHRTMKIFTIVYFILYSGSLLLDLYYLFNNFSIAAMVRYG
CMIMLISYVIAGMLFCFIFEKQLLNLLSEAETIFWPPEMITSELPKFIHRTNVLNYFIIAWFGL
LGVILFPVWGDQSEWFLNVWAYKAYFGSWWYIPYNLFYYSQPMAAWTCVRLPFIMMYF
SLQIKLQIFLLNQQILEIPKGHNTNSETAPDDLSYQEAVSQKMCLCISHNVKIKRWTKSFLR
KVIQAMPVFVLLGILGSIFVTFSVLYSFESTSTILKIRLVVVVGCTILSVYMFVEGSQRLCDE
SSQMFEMLAYSPWYLYNKNNRRILLTFMTNTLEPITITWGGIILNYNFGLTMLRMSFSYAL
FLYNIH
>TcasOR264
MVYLKDPFITLRVMFLNFNKYKIVKCCDFSFIIFYSLVFCLQIYYLISYFSANPLIRYATTILL

VLWGIVGAILSVTLEKQILEATAFLDEMCWPLNMVRKEAQTKLERSCRIINIYITCSLLLILI
TVVFNMLCFSSQRDFFINIQIFEEYFGELSHVFNGLYFTGFPYLCYHGARLCYVFVYAILQI
QLQFSLIEEYLLQVYEIDCLKSWRYLRDTRYQQEMGKSLRLCITHHNALKKFVKMINDMS
LICMPFCLVLGVLILISCLAFVINFGDTLTIFVKLRILIFVVSCLCVLSVFCWSGQQLTDVSSY
IFLTLARAPWYYWRLENIKILLTFSTNCTKNDSIVLAGIRLEYMLFVSMLRISCSYALVLFN
LRK
>TcasOR276
MTMQFIVKRATRGIFHDLRVLKFISSDIFDIKIMKLCLFITFLIHLTACAITIHAFMFNNFSRR
EFISCAPVLFGCFYGLLGLGTILFKPSMTRTLMLELKAWDITAADDAVSSRIKFEINVITVFC
LVNYLLALVASFFYYMSFYGDEEIFYLIRFLEDHCPNHKRVLIKLYKISFVLLGYVMVVHA
CQVLYATQHVRFQLILCAHFMANVTKQAKNIKDEHLPDDNNYQNMIRERLKFCIIRHQEIR
RFYFDKLEEMGNLIGGFALLGCFLGISFAMHMLTSEFLRYHFARTVSSIIAGVTTFATVIAA
GQSVETEVDISTRVVKEVKWYTFNESNKRSYMLMLLNSMQTYKIKFSENYSINYELGLSI
VRGVFSIVSVVVQLDY
>TcasOR277
MDQVLEKFPENDWLRGVKFISSDIFQRKLVKAVLFMVLLVHLTASVITIRAILIKDITAKEFT
FYGPVFFGCFYGMLAIYIILFEKNFIANLSGELKMWSFRSAGAEITRQIRFESRVVTIYAIINF
VMVVIASCLHITPLESDYETFYMIRFFEDKIPDYANVCKTSYRSTFLVMGYVMMVHVYQII
YATQHGKFQIMLYLEYVKRVTQFNEKIGEKCLFYNESFQKMVARKLKNCVIRHNEFLKYH
RKNTREMSHWIVAFSLCGCLLGISVFFYILSGVIYREQYFRVAVLLTTAASTFVAFIVAGQSL
ESRVDNGYSVVSRIEWYNFSETNKKTYFLLLVMLMQPWKIKFSDKYSINYELGLSIVRGIY
SIISVMVNIRFDS
>TcasOR278
MNQPQESFLKNDYLKVLKLISSDVFEPRLVRAILFVVFAVQLTASIITVRALLIKELTAKEFV
LYGPVFFGCFYGMLAIYIIIFQSSFITNMSQELEMWSYSSGGEEINRRVKFQSRVITIYALVNF
LLAIVASYLYFSPLDSDNETFYMVRFIEEKIPDYAKICKIAYRTTFLAMGYVMIVHSYQVIY
ASQHVRFQIIFFTEYVKKVVEFDEKISEECLFYNERFQTIVGKRLQNCVIRHIQFLKFDRIKI
KEMSNLIAAFSLCGCLLGISISFYVLSGIFYREHFLRVALISVTAVSTFFALILAGQSMESKAN
SAHIIMNNIKWYNFNQSNKKAYLLLLMMSMKQYKIKFSENYSINYELGLTIVRGIYSIISV
MANMHFDN
>TcasOR281
MDYSEKSLIQGDCLKLLKVISSDIFQPKLVKLILLIVFGVHLVVDLLTLRALLVNELDFKEFI
FYGPVFFGSFYGMMALLTLVLKDDFISNLKQEFRLWPLDCAGDEIYSQIKFENKIIKIFVVF
NCIVTFIGSYLYFLPLDSDNETFYAVRFIEENYPDHRNLLHGLYRSTFLIFGYAMTVHVYQV
IYNSQHLRYQIIIFTEYVASIGNPDKRKENELFYDKGFQKVVYERLKFCIMRHQEFLVISNK
KVGDMRVFIVGYSLCGCLLGISLTFYIFSGKFYREHFPRVSVACVGAVTTFWAVITAGQAIE
SEYDSLLSTLLGKIEWYYFNDSNKKNYLIMLINLMQPWKIKFSEEYAVNYELGLAIVRAIY

SIVSVIASMHFEA

>TcasOR282

MHDYCFEPLTKNDYLKTVRFLCCDVFEAKIVKLGLWITFGTHLIVSVVTVRALLYDLTINE
FVHYAPVFVGSFYGLLALWTILFRIEMVRDVRKQFKFWTIDCAGQEAHSRIKSEIRITTVLS
VLNFIITLYASYWYVYPIEGDKEIYYALKFFEEYCPRHKMVLSVVYRATFPLLSYAMIVQA
YQVIYTTQHIRFQAILFIEFVLNIGHQTKNLSEEKLFYDTDYQKIVGERFKFCIMRHHEFIAF
RRLKLNEMSNLIVGFSILGCLLLFSFGLFVLTGKLHREHFWRFGLGSLAAVCTFGSVIWAG
QSIEIESENVVNSLNSVKWYTFDENNKRNYIIMLVNTMQPYKLKFSENFSINYSLGVSIVR
AFFSILSVAAKLYFNHV

>TcasOR300

MIGLTNGDYSPRPSMEGDCLKILKFFAVDIFNPKIVRFFLWIMLLYHVVFTLVTAYFMLYVL
SNSEIIGYTPAFLGNFYPMLCVWSVLFISRLIYVKEDMPLWAIDTAGAKVQASIKRKIFLYT
AFGIFNLVLSLSAGSFYLKNVSEDVNVFLALRIFRDYFPNYYQVLDLIYRLIYFCFSYLMVA
PSYLLIYYILHVRIQAIIFAAYVAHIDGHSDYGTDIDLFDNEEFQSEVERRFKFCIKRQIEFLL
MESKKLSQISNLIAAFSLAGCLFGISIIFHLFTGQLIQEYYFRIGLTSLAAIATFSAFIYTGQST
EVQIELVDNAIDNLCWYNFNRSNKLLYLIAKADLARVRKIKFSGQWAVNYDLGFAIVKGIY
SIISVVVSMW

>TcasOR309

MPFEWTIRKNKIKPILQNDVLLNLMLVPNTIISNKFLVILNYFYFGFIILQSVFVAVIIITKDE
WKLLNGQYAGYTSGCAIVWSSYITMYTYVDKFLNLYKEIFPHLWSLDVVGQDHFNKFSK
MAKVLKLGKNILLVVGFLSATVGLPWYRDEYEIIITVRVYKDYVDKWTTLLYFVLFSSLY
HIALTVIFCVLCLVYMVLHLHNQCVMLNKRLEALDDEQLFLDNDNYQDFVTKELKFCIQ
QHQFLLKFAKRLNDILYYPTFYYVLSGVVTGVSLLLFPKNDIKNLLRCVLIIVLGGGFAISF
CFLGQILENASEELLFSAYSARWYLWNIKNRKLLSVFLLKTQDNIVLSSSGIITINFRLLISLY
QSIYSCLTFLLNIK

>TcasOR311

MHHKNIQPMTDDYLKFIKFVSSDIFQLLPVKIFLAVVFLTHAVLDLLTIYFVLFVIEPHDFIT
YISVFLGEFYAPLFAIVMLLFRGKITDSLKHKLAMWTITSTDEKTQSDIKRQIVFFNGFVVL
NSVIISIASWFYAARLSDDVNAFFALRLIHEYFPKSIFEVIYRVTNFVLGQMMCVHVHQTLY
YTQHINIQVQMFKKIIRDLENESKIEQQLKFCIERHAEFIKIITLTTKELRGAFVGFAFGGLLL
GVAVAFYIFSGLLTPEYYLRVGAIGLASVVNFAVTIWFGQSTESHLDELMLAVGEVQWYNF
SQRNKKVYLILLMNVMKGRKWRVSEEYSVNYRLGLAIVRGVYSIISVTSSYKKS

>TcasOR313

MEQLPKNDPLLVLRVLPELLLLHKIVRHFVVFIVCYLTATTIFCLYVLATVRGLWDLFWSQ
YSLLTFGSVIGFSCYFVAFWKGFKFLELRRRVFADYWALTSLGEESFQKIKKLSKSANIFTV
GTILASIATSSTCMPWVGDEYDIMFPVRVYTDYFGERAVPLLVPFYLAMYCTGFVMIATGF
IFVHFALHLKFQFFLLNRRLDGLQTEPLVNDFSYQNRVKEELTCCIEYHQKLLKVAKEMNE

IVYYPIFIVVSSGIICSVCLIFYMKTFENSIVRGTAMAISGGLITFGFGFTGQLMENESGRLFD
TSVMLPWHLWCLSNRKLYHIFLTKSQYHVSFSSSGIINLNHTLFISLYRKVTSIFSFLMNVSN
KNST
>TcasOR314
MEQLPKNDPLLVLRALPEILMQHKIIKYVVLFIICYMTVTMILCSYVLATVRGLWDLFWSQ
YSLLAFGSSIGFSCYFVAFWKGSEFIKLRRRVFANYWPLTSLGEESFQKIKKLSIFANVFMV
ATILASLATSTAGLPWVGDEYDIMFPVRVYTDYFGERAVPLLVPFYLAMYCTGFVMISTGF
IFVHFALHLKFQFFLLNKRLDGLRTEPLVNDFLYQNHVKEELTCCIEYHQKLLKVAKEMND
IVYYPIFIVVSCGIMFSVCLVFYMKNFKNSFVRGTTMAMTGTLTTFGFGFTGQLMENESGR
LFDTSVMLPWHLWCLSNRKLYHIFLTKCQYHVSFSSSGIINLNHTLFISLYTKITSILSFLLN
VSKKNHTK
>TcasOR315
MTLVRKLQAAATNAFEIRIKDDILAELFNWPFLVLDSKWSTKFAVFLTVYCVFETLACALV
YSTLDVNMMGTYAIVIARFATTFCSFFSFFTKRKQYFEIINENFPHFWPLQSLGKSTFNRIK
MRASSVKFYSFLNVVVMLIGAVILISFTQDESEVYLSVKIYKDYVNKWTTGFVMFFYVSFI
YIGLVVAAISFVLTYTAFHLIFQCFLLNQKLKQINDSIVENEQKQAKFDEKYQSFIYKELISC
VKLHQRLILFGKRINHLVYAPLLVYIFGGIVVGVALIYYLKSSVQHIFTSLILLLIALINSTTF
VINGQMLENEAENIYISLTNLPWYSLNVQNRRVVYVMLMQSQKIIHMSASGLVSLNYQLTI
VFFRCIYTGMTFLVNVGL
>TcasOR316
MTLMRKLQTAIRNLFEIQIKDDILAELLDWPTLVLFSKWPKNFAIFSTIYCVFDTLVCTLVY
STLDVEMLGKYAIFIAKSTIALCSFFSFFAKRKQYHKIINENFPHFWQLQSMGESTFDQMK
KIATTVKFYSCLSVVAMLIGAVILILFTEDESEIYLSVKIYKDYVNKWTTGYIMFFYASFLYI
GIVTAAVVFGLTYIVFHLIFQCFLLNQKLKLINSYIVKNGQKLVKLEERNQNFIYKELISCVK
LHQRLIYFSNQINDLLYAPIFMYTFSGIVVGVALIYFLKTSIQYILTSLVLSIVSLIITTTFVING
QLLEDETENIIISLTNLPWYSLNVQNRRVVYVMLMQSQKIIHMSASGIVSLNYQLTIVLFRC
IYTAMTFLVNMGL
>TcasOR322
MTFHWITTPLEPILKDDPLFVLMALPNKLIGSKLQALVNYFFFVYMVILPVSCFLVIVATNQ
WQIFYSPYSGYASGVFIVWSCYVSFFIFGSKYRRVYRDVFPHLWSLDVAGEEHHNRLKKIG
KQLRTFKLVLITLAFIGATSGLPWFGDDYDFYIPIKLIVDYCDQWKLFFSIFFYLSFYHIGVT
VLSCFFSLMFLVLHLQNQFYLLKTRLQTFATDSGTSDVFLSMKVKDEEYNRSVTQEIVFCI
RHHQSVLMYCDRLNDLLYLPIFYFTLSFIVTGVSVILFPKYDLQALIRSLFVIVLGMCMTLL
FCSLGQLIENESENVLYSLIEAPWYLWNTTNRRLYYLFLLKAQDTVNLSSSGLITINFQLILT
LYRGIYSALTFFLNFS
>TcasOR328
MSYNIKLTKDDRLKLLKIMASDVFQSKTVKIILIVVFLVHAIANSLTIYFALHVSDTKQFISY

ASVFFSEFYPMLAILTIIFKGEVVQHLTDDINIWTIDGASKKLQSEIKLKIKILTAFVIINSFSV
VIGGFCFVQQLSDDVNLFFAIRLIRDYFPNHSTILEFFYRMTYPICAYLMAVHAYQCLYYTQ
HINFQLQMFTEIITELTDLKTISLPENRLFYNKKYQTVIEQRLKFCIKRSQEFIKVCVTKNKE
IGSLIPGFAICGLFLGIGITFFLSTGKFTTEYYLRMGVTSICGLTTFSALIWSAQTTETMINDL
VMVINKVSWYNFNQSNKKLYLTFLLNTMKERKIKFTEKYSVNYQLGLAIVRGIYSVISVV
ASKRHH
>TcasOR329
MNCENQFAKDDYLKTLKIMASEVFQSKAVKVILIFVFLVHAIANLLTIYFVLYVSDTKLFV
NYASVFFSEFYPMLAILTVIFKGQIVQHLTDEFKIWAIDSASKKLQSEIKLKIKIITAFVITNSL
IAVWGGFLYVQPLSEDENLYFALSFIHQYFPNQSSTLEFFYRMTYPILGYLMTVHAYQCLY
YTQHINFQLRMFTEVVAEFAPVKRFLLFEHHLFYNKKYQTEIEQRLKFCIKRSQEFVQICVI
KNSEIGSFIPEFAICGLLFGIGVTFFLSTGKFTSEYYLRMGVTSFGGVMTFSALIWSGQTTET
MTSELVKALNEVRWYNFNQSNKKLYLTLVMNIMKERKIKFTENYSMNYRLGLAIVRNIYS
VISVVVSKRRH
>TcasOR330
MNYKKQFAKDDRLKTLKLMASDVFQSKTVKIILTVVFLVHFIANSLTIYFVLYVFETKLFIN
YASVFFSEFYPMLAILTIIFKGDVVQNLTDEITFWTIDSASKNLQHEIKLKIKFLTAFVIINSFT
VVMGSFSYVQQLSDDVNLFLAIRLIRDYFPNYSTILEFFYRMTYPICGYLMAVHAYQCLYY
TQHINFQLQMFTEVITELNNSKTSSLLENHLFYNRTYQTNTEQRLKFCIKRSQEFIKICVTK
NKEIGSLIPGFAICGLFLGIGITFFLSTGTFTTEYYLRMGVTSICGATTFSALIWSAQTTETMT
SDLVMVINEVNWYNFNQTNKKLYLTFLMNTMKERKIKFTENYSVNYQLGLAIVRGIYSVI
SVVASKRQH
>TcasOR331
MNFFQKKLAKGDFFKTLKFIASDVFQSKAVKMVLILLFLIHAIIYLLTIYFLLYVLEPKQFV
NYATVFFAEFYPMLAILTVILKGKIIENLTDEIKIWAIENASKNLQSEINLKIKIITTFVIVNTLI
AVSGGFLYMHPLPEDVNLFFALRLIRDYFPNHYTSLEFFYRMSFPIFAYLMTTHANQFLYYT
QHINFQIKMFREVCLEVKAWKTVSPFENHLFYNKKYQTEIEQRLKFCIKRSQEFVKISVYK
NKEIASFIPGFAICGLLLGVGLVFFLSNGKITWEYYLRMGFTSLGGVTTFLALVWTGQTTE
NITSDIERAINEIRWYNFNQSNKKMYLILVMNTMRERKIKFTEKYSVNYRLGLAIVRGIYS
VISVVLSKYQH
>TcasOR332
MEFGNYKLMTDDYLKTIKFMSSDIFQPIPVKILLGFIFALHSAVNLVTAYYMLTTFDAKLFI
NYSSVFFGDFYPLLATFALISKNNTVRNLKDELEIWTIDSAGEKLRSEIKLKIKFLNIFVVCN
SLLVLVTGLTFIQPLPKDSDIFFAYRLIHEHFPKHGQALEFLYRTTYVLISYIVAVQPFQIFYY
CQHINFQLQISIETLKKISDWKTLSEDGENLIDNVKYQTEIKRRLKFCIQRSQNFICLHTEKI
KEVSTFIAGFAVCACLLGIGVIFYLISGNFTPEYYVRMGFTSVVGIIIFAATIWAGQSTESAID
EMVTSLNEVEWYNFDQSNKKLYLIFLINSMRERTIKFTENYSFNYQLGLAIVRGIYSVISIV

L

>TcasOR333

MEFEVKTFMTRDYLKVVKFLASDIFLAKPMKILLLLIFIVQASVQAMTGYFMATAFNAKF

FNNYAPIFFGTFFPLLAISILLLKNKIFHNLKNELKIWSLDNAGEKIHSGITTEIKVVTYFVIV

NSVFVLLANSTLAYPLSQDVNVFFGCYLIHKYILTYGRTFEFFYKATYLVIGHTNTGHVYQ

LLYYTQHINYQLQLYIEFIKFLDEGKTISKNEDDLFNNPTYQTLINQRLTFLIKRGQEIVKFHI

KKTNEIRTLIPAFSVCTCTMGIGVVFFIISDNFIREYYFRMGMVSLVTVSTFAAGIWSGQSM

ETNLNEITTALNEVKWYNFNKSNRKLYLIFLTTSMRERKIKITENYSLNYQLGLTIVRGIYS

VISVIINMK

>TcasOR334

MDHPDIKPMTDDSLKLIRFIASDILQPLPVKIFLGVIFLFFTVGSNLLMIYFVLYVYDIREFM

DYAPVLFASFYSGVAILSAIFKGKIIHTLPDDISLWALDSGGEKIHSEIRFKARMVTIFVICNT

LLIIGGIILNLIPLSDDLHVYFALRFIHEYFPNHKTCLIILLKASIFPVIPHMLVVHAYQILYYT

QHSNFQIQLFNKVIAEVDFWETPLRETELFYSKPYQKGIEKKLKFCIQRLQVLINAYIVKTK

EIGTLIALFAICGVLMGIGFSLYLFSGKFTPEYYLRLTFMTLVAVTTFSSIIWGGQSTETIITEM

ITALCQVRWYNFSQTNKKLYLILLTNMMKDRKIKFTENYSINYQLGLAIVRGIYSIMSVVV

KMRS

>TcasOR335

MDHPDIKPMTDDPLKLIKFMASDILQPLPVKIILLVTLLALPVGSNVLMIYFVLYVIDIREFI

DYAPVLFGGFYPSLAILIAVFKGKLIHNLQDEIKLWAIDSAGEKIHSKIKLKVRMVTIFAICN

TLLLIVATVHNLIPLPRDLHIYFVLRLIYDYFPNHKTYLLILMKLMSPVTTYMLLVHAYQIL

YYTQHINIQIQLYNKFVADVDFWETPLCEPELFYNELYQKRVEKRLKFCIQRSQHFVYVHV

AKIKEIGILIALFAVCGVLMGIGISFYLFSGNLTPEYYIRIFIIALVGATTFSSIIWGGQSTETIV

TEMIATISQVRWYNFSQTNKKLYLILLTNMMKERKIKFTENYSINYQLGLAIVRGIYSVMS

VLVKMYSINT

IRs

>DponIR25a

MKNNNIVAGGFFSIFLLNVADICGQTTQNINVIFANEEGNFVADKAVTVALNYIKKTSKLGL

SVDLRRVVGNKTDSQNVLDSLCAAYQQMLDDNNPPHLVLDATRAGLASETVKSFTAALG

IPTVSASYGQQGDLRQWRNLQPNEEEYLVQISPPGDIIPEMVRTLVLNQNITNAAILFDDSF

VMDHKYKALLQNVATRHLIDEINEDVNKIPDHLESLVKLDLKNFFVLGSLQTIKNVLEAA

EKKSLFNRMFAWHVLTKDPDDLKASIKNATIIFAKPIVNNLYQDRLRNIQTTYQLSSVTPEI

EAAFYFDVALKGFLAVKEMLLDGSWKKNNVTNYVTCDDYEPKYSPKRFNLNLRSYLQK

ESSEPPTYGPFAIESNGMSFMEFSMALSAVYVRSGASDKSLPLGTWHGGFNNNMTLLTPK

DMKNYTADVVYKVVTVVQKPFIYRDDTAPKGFKGYCIDLIDEIAKILHFDYEIDAVADGM

FGNMDENGKWNGIIKDLIEKRADIGLGSLSVMAERENVIDFTVPYYDLVGITILMKMPETP

TSLFKFLTVLENEVWLCILAAYFFTSFLMWVFDRWSPYSYQNNREKYKDDEEKREFNLKE

CLWFCMTSLTPQGGGEAPKNLSGRLVAATWWLFGFIIIASYTANLAAFLTVSRLDTPIESLD
DLSKQYKIQYAPVNGSSTMTYFQRMADIEAQFYEIWKDMSLNDSLSDVERAKLAVWDYP
VSDKYTKMWQAMKEAGLPPDLDTAIERVKKSKSSSEGFAYLGDATDIKYLEITNCDMAIV
GEEFSRKPYAIAVQQGSPLKDQFNTAILQLLNRRELERLKERWWNKNPEKKQCEKADDQ
ADGISIQNIGGVFIVIFVGIGLACITLAFEYWWYKYRKNTRVTNVAEAPNSRHHKVGGVQ
KGFPRQFEGESDMKITKLYPKTKF
>DponIR75q
MNTLYFGFFMWFALICGGHCGVHADDVLLLIEDLLDFYRFSKKVYTHVCWDKELQLHLP
RFALHAKYHEFQSTKSQVVILMDMSCKGADFFLENMQKRNLLTPRVILLLINPAALTPHYF
PVNSNALFLKPSGEGFAISKIYSGVSNVTIGTWTKTHRYIERPIAKKKLRATNLKVCYLVGD
KHEGLESSEYQPVAEATTKLNRVLLEDGIRMINSTKTDAFQIGSGEPELVNDLIAGKCDIGG
TPLALTAEKIGRLDVLAKTLREDKTFVFRAAPHSYISNVFTLPFDSYVWCSCFGLMGVIFLI
VHLVVCWEWKDPVFKLNLQPNISLRPNPVDILLMEVGSAAQQGFEAEPRSNSGRIVFMST
LISFMFLYTSFSASIVALLQSTTDSLNTFDNLFQSRINVGFKQNISLDFFNDLKHQPNRADY
HGKLKEPQFFTLEEGVKRLQNDFFAFYAETSEVYRYINRWFQESEKCSLREIPFKNTHINH
WLFMGKNSQYRDALRIGMNSIQERGIRSREYKRLFPLKPFCDSIGGNFESVGLVDSYGAFL
IMVYGVALSSLLFLLEHMSLQYNLSTKAGRIRNRIFNSIHRGDE
>DponIR76b
MGLMEVVLTTLATLCFNSTCVDQDLINASKQRLAHLKEELKHETLTVTTLKNGPLSGYEI
VNNTVIGTGVAFEILNIVQREYGFKYNVIVPDHDSFEPVNGGEGGVRNMLLNETIDVAVAF
LPQQYTDVVSYSRSLDTAQWVVLMKRPKESASGSGLLAPFTATVWSLIIISLLGVGPILWL
TILLRARMCKEDHDIVFSLPSCMWFVYGALLKQGSTLNPRTDSSRILFSTWWIFITILTAFY
TANLTAFLTLSKFTLPISEPKDISRKHNKWITNRGNGIVEQLYLSKKYANGDGNSLFEEIGM
PQWEPDVDEDTMLSTYVIKQNMMYIREKTVLESIMYEDYKVKTKADVEESKRCTYVITK
FAVCVFPRAFAFRPGFKYKELFDFTIQHLSESGITDFQQRKSLPDTTICPLDLGSKERRLRNS
DLAMTYMIVGGGLIISTIIFAVELIIYYAKMHCFNKKSHVNNNNTLVTQSNNGLFVKNHQH
QGNFRASKQFVSPPPSYHTLFHPPNLTNGEYKNKTINGRQYWVFNDKQGMTSLIPQRTPS
ALLFQFTN
>DponIR93a.1
TKWTIEAGETWGREYQMLDEATNAELLAVGTWRPSDGPNMIDALFPHVAHGFRRKLLPL
VTFHNPPWQILKTNSTGDVVEYGGIVFNIIKELSKNLNFTFNVATVKPQSLLNASTLQSPKG
DTDSSANFNGNSYITTYRVPHSILEMVHNKSAALGACAFTVTEENQRVINFTDPISIQAYTF
LAARPRELSRALLFISPFRGDTWLCLSATIISMGPVLFYIHKLSPVYEYKGVRCKGGLATIQ
NCIWYMYGALLQQGGMHLPYADSARIIVGSWWLVVLVIGTTYCGNLVAYLTFPKIEVPMT
TIDDVLAHKEMVSWSYAKNTLFEARLHNSVDKSFNIIFKDAKNIWDRKAMMGEIKSGKH
VYIDWKIKLQYMIKEHFIDSGECSFALGVEEFCEEQIALIVAPDTPYLHKINEEIKKLHQVG
LIQKWLSDYLPKKDKCWKKKRTIEVNNHTVNLDDMQGSFFVLFIGFLIAVIVISLEMLWSR

KVTNNRKRKVVHQFVT

>DponIR41a

MGSEMKILETFSQYVNASISPVINQADYWGEIWNNWSGSGLMGNLVEDKADIGAAALYT
WEFAYEYLDLSKPTVRTGITCLVPAPKLSAGWLTPFRVYSLEAWMALIGTLALSFLALYAL
NKLQISVKPQLKSKHHINQLKGKLLSKTLMSVSKPFVMQSITNKEMAQGNLAKYLMGLV
FLSTLVLSTTFDSGLATIMTVPRYDNPINTIEELAESGLPWGGTQDAWILSINNSLEPNLMK
LVARFVAHSEANLRKYSLGDQFAFGVERLPNDNYAIG

>OtauIR21a

MNVHVVLAFLAATVECKTRKRALQKSHEKPQIDKWTDKILGKDFFNQDSTLANLISTVIN
NHLKDCVPIIIYDNHSRNKSSLLDQILKKSEISILHGQISDDFTLKQRRLMDPQPNACKSYV
LLLQDVIKTKDILKLKTSEKIVIVTSASQWRVNEFLYSELSRSFVNLLIISPTLDSLVNNEAY
VLYTHKLYIDGLGSSEPTILTSWINNSFTRPEVQLFPQKLEKGFSGHRFITSLSNQPPFVIKTG
TDDNEDIVWDGSEVRLLKLLSEKFNFSIDFKESITDVEKSPADTVIDSVIENKAIVGISGIYIT
TERLRDLDVTYPHTQDCAAFISLTSTALPRYRAILGPFHWSVWAALTTAYLLGIFPLVFADK
HTLVHLIKNPEEIENMFWYVFGTFTNCFTFGKESWTKSDKNTPRILMGFYWIFTTIVTACY
TGSIIAFITLPIYPITIDTVRQLLSGRFQIGTLNKGGWDYWFKNSTDPYTEKLFKNVEYLPNI
ESGLKNTTKAFFWPYAFLGSRAQLDYIIRTKFTSTNKRQLLHISSECFVPFGVSIIFEKNSEY
KDFINKGIGNMIETGFMIKFQNDIEWNMMRSSTGKLLQANTGNNLRSIIVDDRSLSLDDTQ
GIFLLLGAGFLMGLAALISEMLGGCFNCCKRRRLSGISLKSNPRVFAVATPREKIDSNLIKN
DDLETKFFGDHFDAFEEAEEVPSGE

>OtauIR25a

MDYIKRNTKLGVKVDLRKVTGNRTDSKGLLDLLCKTYQTMLDEKSIPHFVLDTTMTGIA
SETVKSFSKALQIPTISGSYGQEGDSRLWRSLDEKQEEYLIQIMPPTDMIPEIIRTIVINQNIS
NAAILFDNSFVMDHKYKSLLQNVPTRHIIAHIVNYFVLARLVNIKKVLDAADAINYFNRKF
AWHAITQDKGDLKCCFNIIEAQTTQNLNVIYVNEEDNDVADKSIEVAMDYIKRNTKLGVK
VDLRKVTGNRTDSKGLLDLLCKTYQTMLDEKSIPHFVLDTTMTGIASETVKSFSKALQIPT
ISGSYGQEGDSRLWRSLDEKQEEYLIQIMPPTDMIPEIIRTIVINQNISNAAILFDNSFVMDH
KYKSLLQNVPTRHIIARIQDVSTLKDQLQNLRKLDIVNYFVLARLVNIKKVLDAADAINYF
NRKFAWHAITQDKGDLKCNCRNATIIFAKPTLDPKYQDRLGIIKTSYQLTSEPEITAFFYFDL
ALQSFLAIKNMINSGSWPSDLSFITCDDFDGKNSPERPDMNIKSFFYKDSTESPTYGLITLS
QNGHSFMEFSMQLSSVGVRSGSSDKSVLLGSWKAGFNNNLSLIDAQVMQNYTADIVYRI
STVIQAPFIIEDPDSPKGYSGYCIDLINDIATILKFDYEIYVAPDGKFGNMDIKGNWNGVVK
ELVEKRADIGLGSMSVMAERENVIDFTVPYYDLVGITILMKLPTTPTSLFKFLTVLENDVW
LCILAAYFFTSFLMWVFDRWSPYSYQNNREKYKDDEEKREFNLKECLWFCMTSLTPQGG
GEAPKNLSGRLVAATWWLFGFIIIASYTANLAAFLTVSRLDTPVESLDDLSKQYKIQYAPLN
GTSSMTYFDRMANIEAKFYDIWKDMSLNDSLSDVERAQLAVWDYPVSDKYTKMWQAM
KEAGLPNTLDEAVERVRESRSSSEGFAFLGDATDIRYLELTNCDLQMVGEEFSRKPYAIAVQ

QGSPLKDQFNTAILQLLNRRQLERLKEKWWTNNPKAMKCDKLDDQSDGISIQNIGGVFIV
IFVGIGLACITLAFEYWWYKYRKGAKVITVQEVAPQNVTKMVKKSKNENLSRKSSKLYPR
SRF
>OtauIR93a-X2
MLSLTFLMILTFPSTFGNNFPSLLTTNATLAVILDKEYLGDDYANIKDVVETYVGFIKREKL
KNGGLNVIFYSWTSIKIKKDFSAILSVASCSDTWRLFDVARNEHVLFMAISESDCPRLPQDL
AFTIPLIKQGQELPQIILDLRSNKAYNWNSVTVLYDLTLSRDMITRVLTSLTLDNVDIAATAS
TIALMELTQNYSSIYNIHYVREVLTSLKWRNIGSNYLVMISIELVPLVMDISKNLKLVNTES
QWLYVISNADLKNETNLDVIKHLKEGDNVAFVYNVTSSEENCKQGIQCHIEEVLDAFSRA
LESAILDEIDLAGQVSDEEWEAIRPTKSERRNFLLKSMKSYLSQNGECDNCTIWQMKAGD
TWGREFLRNSSVLSVELLQVGYWKPREGPLMTDVLFPHVKHGFRGRRLSLAVVHNPPWQ
VIEVNNSSVTFRGMVADIVNELSKNLNFSYAVIPIGNTSSSKLNSDYDFNDISTYEIPDVIINL
VRTRKVLMAASAFTISEKTKTLVNFTLPISTQTYTILAARPKELSRALLFMSPFTQSTWLSL
LTAILLMGPILYLMNKFTPVYEYNGISVKGLSSIYNCIWYVYGALLQQGGMYLPYADSARI
IIGSWWLVVLVLSTTYCGNLVAFLTFPNSDKPISTIEDVLNHPSLTWTIAQNSFFEEEAKTSN
DPKYQELFKKSIKASINKQNMVQNIENGHHVYIDWKMRLQYMMKKQFLLKNTCGLALG
KEEFCEERIGLILAKDNPYTEKINKEIKRLHQVGLIQKWLRDYLPKRDRCYKSRSTSAANN
HTVNLDDMQGSFFVLFFGFLIGLFIIFVEKLLYKYQKKRSKSKSVQPFIT
>PverIR3
MPLLQFIFLCICVCLVIWFKCKENRKLKMHSPFGLLGVQILTSNSSSMWTVIDHGKVLANA
QIQRMTQLAKELKGKTLIITTLQNGDLSGYEEKNGTLVGTGLAFDILHMFQQKYGFTYKII
LPEDNVFLQEPSHRGAKNLLQDEAADIAVAFLPIVNSFRHDVTYSTYFDVAEWSVLLNRPK
DSATGSGLLAPFTTEVWIVIIFSVLIVGPIMYLIVLIRARFSETHEESRIYPLPDCMWFLYGAL
LKQGSVLNPVTDSSRIIFSTWWLFILIITAFYTANLTAFLTLSKFTLPIKGPSDIGAKGYKWVS
NKGNGIRDFIYSETHDNWSIRNKLVDRIGRGQLYSDRKDMDILEEYVASKKMMFIREQSIA
RHLLYDDYKAKTKRGMRENERCTFVIAKFPILEMPRAFAYRRDFKYKKLFDNSIQYLVES
GIVPFKLRENLPDAEICPLDLGSTERKLRNSDLSLTYFIVAGGLAFAICVFLIEILWRLGSQRS
EKFRSVRKQHANVRTWMEKNENLMKRTQGSKFSIATPPPSYQTLFRPPFFFSYSDGHKKNI
NGRDYWVIQKNDGFREIIPLRTPSALLFQFSH
>PverIR4
MNGFRMNIIFILLLFFVGIYGQNLSRLKLVVLKEDGQDEVIQWYNDIINSMQNTEKIDSVL
VDLVDDDTVNKEKICEALSDGGTILLDLTWFGNDIGRTHAYDIGVPYIKIDVTISPLLDILD
KYLDYRNSSDVVIIFENPSYIDQALYHWINTARMRMLISETLDAETAKKLKGVRPTPSNFA
LVANTKNMQRFFEVAIRENLVELPERWNLIFLDFNYKSFDQSLMKNQPINYLSLDTNICCKI
QSLPNSCECSDTFMMQKEFLRAALKVVSTSAQEMMRNGLLDSSLACNNNDSSVKDDIDT
QFQNILKRELSGQNVMYLEKSIMRMITSGFIEIGSDSNTNTVAKYESGVIRPEKNATIKPIKA
FYRVGITHALPWSFKTIDPDSGALVWTGYCVDFTKKMAEMMNFDFEFVEPKSGTFGEKIN

GVWNGVVGDLAYGQTDLAMTAIIMTADKEEVIDFVAPYFEQSGITIVMRKPVRKTSLFKF
MTVLKLEVWLSIVAALIVTGFMVWFLDKYSPYSSRNNKNAYPYPCREFTLKESFWFALTS
FTPQGGGEAPKSLSGRTLVAAYWLFVVLMLATFTANLAAFLTVERMQAPVQSLEQLARQS
RINYTVVKESDTHQYFINMKNAEDTLYRMWKELTLNASTDDTRYRVWDYPIREQYGHIL
LAINDSNPVADAEEGFRNVEDHLDADYAFIHDSSEVKYEISRNCNLTEVGEVFAEKPYAVA
VQQGSHLQDDISKTILTLQKDRFFEGLHAKYWNHSSKGNCPNIDDNEGITLESLGGVFIAT
LFGLALAMITLAGEVIYYRQKRKTIDFKKKQAKGQLPEKYFKQNRMITIGTSFQPTQFNQ
KAVQDQKELKLSHISLYPRARNRITQVE
>PverIR5
MKMGLNKIWLVFLSFLLENCQGETTQNINVLFVNEENNGVAEKALDVAMTYLKKNNKL
GIAVDLKKVVGNRTDSNKFLEALCSTYNSMLSTQTFPHLVLDMTMTGLGSETVKSFTQAL
ALPTISASFGQEGDLRQWRNINESETDFLIQISPPADVIPEIIRTIVLNQNITNAAILYDSSFVM
DHKYKALLQNVATRHIITPIKEVSQLAEQLTQLRKLDLVNYFVLGNLKSIKNVLDAADGLN
YFNRKFAWHAITQDDGDIRCTCRNATILFAKPLPNALYQDRLGAMRRTYQLNAEPIVASAF
YFDLILHSLMAVNEMISDGSWKSGGGQSSEDPTYGPFSVVSNGWSHMEFQMQLTAIGVR
DGASDKSVNIGAWWAGFDNNLTLLDAQAMGNLTADVVYRVVTVEQKPFVFRDESSRSGF
NGYCVDLIDKIADILKFDYEIVAVDNFGVMDENGKWNGVVKELVEKRADIGLGSMSVMA
ERENVIDFTVPYYDLVGITILMKLPESPTSLFKFLTVLENEVWLCILAAYFFTSFLMWIFDR
WSPYSYQNNREKYKDDEEKREFNLKECLWFCMTSLTPQGGGEAPKNLSGRLVAATWWL
FGFIIIASYTANLAAFLTVSRLDTPIESLDDLSKQYKIQYAPLNSSSTQTYFERMANIESRFYE
IWKDMSLNDSLSEVERAKLAVWDYPVSDKYTKMWQAMKEAGLPANMAEAVERVRASK
SSSEGFAFLGDATDIRYLELTNCDLTRVGEEFSRKPYAIAVQQGSPLKDQFNTAILQLLNRRE
LERLKEKWWNKNPEKQNCDKIEDQSDGISIQNIGGVFIVIFVGIGLACVTLAFEYWWYKY
R
>PverIR6
MEIIRTVFLLTSLSVFLSTGQYVEVIGDLIETKDKVMITEYSCNTKESIKLLKLLVKHRHSTR
VLPVNYSNLQYPLPLNRQLSVVNLDCVASSVTLQNANKLHLFASPLQWIFFSETLDTTEIM
KKYFSQIDILIDSDVTLLHPNKNGSISVKKIYKRHRRSSIVVESIVEWDEMDGFGEYRNETP
VWRVRNDMKMTQLNACIVITNNNSLNHLTDKRDKHMDSIAKVNYVLVEHLADIVNISLR
YSVQSTWGYKNNKSEWSGMIGELTRNEADIGGTALFFTSDRIDIIDYIAMTTPTRSKFVFRE
PKLSYVTNVYTLPFDDYVWASVISLVILIGLVLFAISKWERTKRNNPMNLKPNASDLHDSV
SDVALYSFGALCQQGAPSIPSSISGRITTILLFISCMFLYTSYSANIVALLQSSSTSIQTLEDLL
KSRLQVGVDDTVFNRFYFPNSTEPIRRAIYLKKVKSGKKDKFMTMDEGVRKMREGLFAF
HMETGPGYKLVGEIFEETEKCGLKEIQYLQVSDPWLAIQKNSSYKKFLKIGLRKIQESGLQ
HREVNLIYTKKPICTSRGFSFISVGLVDCYPAVVVLAVGLILASLVWILEIFTYYRREISFQIK
TMIPSLIERNRNITHNELQYLD
>CbowIR64a

MDPPRTIFTLKDLAESQLRIGIEDILIDRNYFVQTTDPDAITLYEKKIKGQSNSSGFYSPSEGI
ALVRNGGFAFHVETSTAYPIIEEIFTNQEICELDEIQMYRTQPMHTNLQKNSPFREMMNFC
MLKLVENGNMDRLRKHWDARRPNCIESAKKQEIHVSLSEFCCSPIALTLGVCFSLIFLLVE
CSINYKERLKKVWTFKNHSKSQYPFME
>CbowIR6
MNLRTKMGLYKNILLFQLLLGYCEGQTTQNINVLFVNEEGNEVAEKALDVALTYLKKNN
KLGISVDIRKVVGNRTDSNAFLESLCSTYSSMLDAQAYPHLVLDTTMTGLGSETVKTFTQ
ALALPTISASFGQEGDLRQWRNIDDNEKDFLIQISPPADIIPEIVRTLVLNQNITNAAILFDKS
FVMDHKYKSLLQNVATRHIITAIKDGNQVVDQLSQLRKLDLVNFFVLASLKNIKRVLDAA
DSVGFFNRKFAWHVITQDDGEIKCVCRNATIMFVKPLPNAAYQDRLGTMKRTYQLNVEPI
ISSAFYFDLTLHSFLAIKEMISDGVWKSVTNYITCDDYNTENVPKRNGLNLKKYFNKESTE
SPTYGPITVLSNGLSYMEFQMQLTSVGVRDGASDKSTILGTWSAGFYNNLTIVEQQVMVN
LTADVVYRVVTVEQKPFMFRDESSPRGYSGYCIDLIEKIADILKFDYEIATVDCFGTMDEN
GKWNGVVKELMEKRADIGLGSMSVMAERENVIDFTVPYYDLVGITVLMKLPETQSSLFK
FLTVLENEVWLCILAAYFFTSFLMWVFDRWSPYSYQNNREKYKDDEEKREFNLKECLWF
CMTSLTPQGGGEAPKNLSGRLVAATWWLFGFIIIASYTANLAAFLTVSRLDTPIESLDDLSK
QYKIQYAPLNGSSTQTYFERMANIEERFYQIWKDMSLNDSLSEVERAKLAVWDYPVSDK
YTKMWQAMKEAGLPNTMDEAVAKVRASKSSTEGFAFLGDATDIKYMELTNCDLTVVGE
EFSRKPYAIAVQQGSPLKDQFNTAILQLLNRRELERLKEKWWNKNPEKKDCEKADDQSD
GISIQNIGGVFIVIFVGIGLACVTLAIEYWWYKYRKGSKIIDVREVAHNPTKPPTFPKQKFSE
HNPDNTKPKLPKRSKF
>CbowIR75q
MLNSLTLFMILFLNFSSAMKNYTEIINIVDELLTKQNIPSEVTAYLCWSKVLKANLFKRLSA
SNILTKIIATDDIVDLFPSEYQIYLVDLDCEGSNEILKKAQRKKLFSRPFRWVFCGNIEQPLF
NDLYFGVDSRIFFIDNAGSEYHIKMPYKREKNSKKFTVNDLAEWNSLQGFTRFDEFAAAR
NRTDLFGMNINISCVYTDSDTLNHLEDYRNIHIDPLTKLSWILVHHLMSILNASATVIFRNT
WGYRDSNTSLFSGMIGDLQTGEAELGGTASFFTIDRIDVVEFYASSAPTYMKFIFRAPPLSY
VTNVFTLPFHTYVWYCSFLLLVLIFFAIYVIVKWEWKDVVFREKLERMHDGSISPLRPTFF
SVLLMEIGAITQQGTDSEPKSNAGRIATIFTFIALMFMYTSYSANIVALLQSTTESIRTLEDLL
NYRISLGVQDIVYAHHYFEVRIQLSIDSYPNQTPGLKWLNTQDLGKLWR
>CbowIR8a
MRNVKIILLENEGQDTILTWYRSIVQAFKSPIKFEEFLISVDGEEFDRERICQAFSNGAMMIL
DLTWTGNDLARTVSMEMDVPYLRIDVSLSPFFDLLHEYLNFRNSTDVALIFDDPSRIDQAI
YYWIDNVQIAMSISESLDAMAAKKLRDFRPTPNSFAIFAETKNMEKMFKIALEENLVTLPE
RWNLVFLDFHHKSFDRGLLKKMPVNLLTLDAGLCCQLLNLNSYCECPSRFNTSKMFLKIA
LNMLVTAIEELFKDDFKFHDNIDCDSNFTKDNEESVRKTFEEVLNKAVGNDNLIRLDNSSN
LRLKTTGSIEIGTDVGTEVFAKYENEAITALRNKIVKPIKAFYRVGITHALPWSYQIKDPVT

KKLVWTGYCVDFTAKLAEKMNFDYELVEPKKGTFGKKHNGVWDGVVGDLASGQTDLA
ITALIMTADKEEVIDFVAPYFEQTGITIVMRKPVRKTSLFKFMTVLKLEVWLSIVAALIVTG
FMVWFLDKYSPYSARNNKKAYPYPCRKFTLKESFWFALTSFTPQGGGEAPKALSGRTLVA
AYWLFVVLMLATFTANLAAFLTVERMQAPVQSLEQLARQSRINYTVVSNSQTHKYFINM
KFAEDTLYRMWKELTLNASTDDSRYRVWDYPIREQYGHILLAINDSNPVANAEEGFKNVN
EHLDADYAFIHDSSEIKYEISRNCNLTEVGEVFAEKPYAVAVQQGSHLQDGISKMILLLQKD
RFFEGLQAKYWNNSVKGDCPNTDDNEGITLESLGGVFIATLFGLALAMITLAGEVLYYRR
KRKTKELNIKQSKVFPEKPLDVFPPKPLLLGNNQITIGNTFKPVNLKEKIRKEREAMKISHI
SLYPRARKPINPFEIK
>CbowIR41a
LFLPGDNLLENLSHIYSMQELKYIADIVIVNREYKDEDSQGLYMSDNVFSLWTHSYRGMD
ENAKRMFLDLWFSKNQSFMLDENLYPDKLVNQMGRKLEMATFQYEPYSIIGSSETESKGS
EMVTCLTFARHYNMTPVLVVNDEGYWGDIFDNWTGYGLLGNLVEDKADIGFSALYTWE
SDYYFLDLSKPLVRTGITCLVPAPSLAAGWTTPLYSFSTTMWAAVGSMFFVCIFVQFFMHY
FHAKIYDDTNQSLKLLDRSILCVLKLFVQQVVTTRETPPGRSGKYFMGLLFTFSLFLSSSYS
SGLSSIMTIPRYGRPINTVEEFAESKISWGATQDAWTMSLKGVEDPT
>CbowIR5
MGLIEFVVASLCLNATCEPEDAVVPGVSTHLLKLNELAEELKEETLTVTTFENGQLSGYISQ
NGSFLGTGVAFDIFHILQEKFGFNYTIVLPDADIFMDGFNKKGAKSLLEAKQADIAVSFLPV
IESFRNDVVYSRVFDIAEWNVLMNRPKESATGSGLLAPFTTAVWILIIFSVLVVGPIMYLMI
LIRAKMCKDDNNKIFSLPSCMWFVYGALLKQGSTLNPKSDSSRILFSTWWLFILILTAFYT
ANLTAFLTLSKFTLPITDPTDISRKNYHWVTNKANGLRDYIEYEKHDRLSNGRTLVQDIGK
DRYYADMKDLDILEEYVKKRNMMFIREKTLIKNVMYRDYQEKTKRGVDEEKRCTFVMA
DFPITMFSRGFAYTHDFKYAELFDRTFQYLIEAGIIQFKLRENLPDAEICPLNLGSIERKLRN
TDLMLTYVIVASGLGIAASVFLLEILWRMSKAKYKRTRKRKATTWLEKNNNLMKAKCLH
LHTNSSPPPPYQALFRPPFYYSDRDGGQKKTINGRDYWVIDKSDGLREIIPIRTPSALLFQIS
N
>CbowIR2
MVTEFSCDKLGSFEFLKKLITQGIPTRILLIDKDELSYLFPPNCQIFIVNLQCENSTNILKKA
NSLKLFSFPFRWIIYHHEPINETIFEESFLSLDILVDSDVTLLEENKNKSVSATKIYKRHRNHP
LVIEKMGYWTKTAGLRDDREEKIMVRRRKNLQQIPLNTCIVITHNDSLKHLTDKRDKHID
SIAKVNYVLVEHLSDIVNVTLNYSIQNTWGYKNNKSEWSGMIGELTKNEADIGGTPLFFII
DRVDIIDYIAMTTPTRSKFVFREPKLSYVTNVFTLPFDDYVWASTIALVCIISMVLFIILKWE
WKKKDLPSEKDSSNVPELKDSLTDVILFSFGAFCQQGAPSIPFSVPGRITTIILFVSLMFLYT
SYSANIVALLQSSSTSIQTLEDLLKSRLQVGVDDTVFNRFYFPNASEAVRRAIYLQKVAPPG
KKENFMSIEEGVKRMRQGLFAFHMETGPGYKLVGEMFHESEKCGLKEIQYLQVIDPWLAI
QKNSSYKELLKIGLRQIQESGLQTREVSLIYTKKPICTSRGSSFISVGLVDCYPAAVVSAGG

AILALIVWILELGLYYRPYMWISVKKVFAKSHEKIPGSIEQWPEWPYLK

>TmolIR2

MDSRTFLICLIFIEISTCKIVLPMINDFVEHYNKTQIVLGFVCRKNDALELVKHFSKKLHQV
KAVSVDTRELPYPIPPTYVTFVLDGQCSGAKHLLLRADAERLFATPFKWIVSYDYSYDVAD
LIENFTELNLLVDSDVTVAIMENRETFSLQKIYKRHVNGSMLVENIGNWTKRHGFVDNGY
EKIIYKRRRDLKKTILNSCIVITNNDSLNHLTDKRDIHIDSIAKVNYVLVEHLSDTVNAVLN
YSVRGTWGYKNNESKWSGMIGELTRNEADIGGTALFLTSDRIEIIDYIAMTTPTRSKFIFRQ
PKLSYVANVFTLPFDGLVWASTCALLLIMAMVLYVVVKWEWKKKKYVEIANESNDIEIPN
SWVEVTFTTVAVLCQQGSSSIPFSIPGRITLIFLLVSLMFLYTSYSANIVALLQSSSNSIQTLQD
ILHSRLDVGVDNTVFNHFYFPNATEPVRRALYLQKVAPPGQKPKFYSIDEGIRRMREGLFA
FHVETGPGYKFVSETFQEHEKCGLQEIQFLQVPDPWLAIQKNSSYKKMLKVGLRLLQEIGI
QGREVGLIYTKKPQCLSRGSSFISVGLVDCYPAAVVLAGGLGAAVMVLLLEIYVNHRLKY
YSRKWRRNKRIRQ

>TmolIR4

MVKNLLPYKCVVLLSDDIYNGIYSSAWYRRFGLYMTFIVVNVDEYEDLLSPYETTQAALS
TAKNEGCQMYIFLVSNGLQVARLLRFGDRYRVINTRAKFVILYDNRLFEKNLFYLWKRIIN
VIFIRRYGGQKSGDDKNMPWFEITTVPFPSQITSILVPRRLDIWTKSKFRKGADLFRDKTFD
LRNQTFKVAAFGHIPGTTKNMKVKSFRAVLGNFSGVETEILQTVATAMNFKCEVYEPVNA
DAELWGGKQTTGKYTGLIAEMVSTRADIALGDLYYTPYILERMDLSVPYNTECLTFLTRES
STDNSWKTLILPFKPTMWAAVLICLAISGSVFCALARFHETISRIKTEKSRNLDLYNKKKKII
TLSMCPELEKLDPNVKYTMMKEQYNPPRFEGQAMGLYQFSQPFNSMLYTYSMLLLVSLP
KLPTGWSLRMLTGWYWLYCLLLVVAYRASMTAILARPTPRVTIDTLQELVNSRLKCGGW
GDINREFFKASLDPTTKLIGENFEV

>TmolIR5

MGLIEIVLATLCLNSTCLLDEDLTYQDVSVNTRKMQFAQLAEELRQENLIITTLKNDRLSGI
LKDNGTLTGTGVAFDLLNILQKKFQFNYTIVLPKTNVWGSQKTGILNMLKEKRANLSAAF
LPVLTQYSTDVSYSPSMDTGEWVVLMKRPQESATGSGLLAPFTLPVWLLILLSLVVVGPVI
YFIIYLQSKLCPDDQNKVFPLPACIWFVYGALLKQGTTLNPMTDSSRLLFSTWWIFITILTA
FYTANLTAFLTLSKFTLPITEPKDIGAKKYKWVTTQGNALEDTVTTDGSLTDIGNMLGKPY
RYLDQSDRTILKQYVDRRDMMFIREKPIVEYVMYDDYKEKTRNQIEEPKRCTYVITRFSIV
SFSRAFAYSKDFKYKMLFDSTIQHLVESGIIKYKLREELPDTEICPLNLGNKERQLRNQDLM
MTYEIVGGGFIISAVIFVIELAVKRSHKPKQAKTSGKSNLLLNQKHTIHVNINNNYEKFGQP
PHNAKFITPPPSYHTLFNPPNNENLKKKNINGREYWVYDSLGGGTKMIPMRTPSALLFQY
TH

>TmolIR6

MSILEYITFAVLSVLSLSINTKGQTTQNINVLFVNEEGNLVAEKAVDVAVNYIKKNNKLGVN
TEPVKVIGNRSDASALLDSLCSSYNDMLASSMFPHLVLDTTMTGMASETVKSFTAALGLP

TISASFGQEGDLRQWRNIDENEKEYLVQILPPADIIPEIVRTIVLNQNITNAAILFDNYFVMD
HKYKSLLQNVATRHVIAPIKEPEKIGDQLRQLRKLDIVNFFILGSFENIKRVLDAADSVGYF
NRKFAWHAITQDKGDLKCNCRNATIMLAKPVIDAQYQDRLGLIKTSYQLNAEPEIAAAFY
FDLALYSFLAVKEMIADGAWKRNNATNYITCDEYDGKNSPKRIGLNLKKYFNKDTSETPT
YGPMSIISNGYSFMEFNMQLSAVGVRESSSDKSIPLGTWKAGYDSNLTLLDPQNMKNYTA
DVVYRIVTVEQKPFIIKDETAPKGYKGYCIDLIQRISEILNFDYEITPVSDQKFGNMDENGK
WNGVVRELMDKRADIGLGSMSVMAERENVIDFTVPYYDLVGITILMKLPKTPTSLFKFLT
VLENEVWLCILAAYFFTSFLMWVFDRWSPYSYQNNREKYKDDEEKREFNLKECLWFCM
TSLTPQGGGEAPKNLSGRLVAATWWLFGFIIIASYTANLAAFLTVSRLDTPIESLDDLSKQY
KIQYAPLNGSSTMTYFQRMANIEAKFYEIWKDMSLNDSLSEVERAKLAVWDYPVSDKYT
KMWQAMKEAELPNTLDEAVDRVRDSRSSSEGFAYLGDATDIRYLEITNCDLQMVGEEFSR
KPYAIAVQQGSPLKDQFNTAILQLLNRRELERLKEKWWSRNPEAKKCEKQDDQSDGISIQ
NIGGVFIVIFVGIGLACVTLAFEYWWYKYRKGSKVVDVQGAPHVKHPDMMSKINDGFN
AKVNKLYPRSRF

>TmolIR75s.2

STGGAKCLSVTLQCAWTICHVDFIHHINVYVRVLLGQYSGIIADIVKQYKNSGRPFKLQNS
FRSRRHGFQSLFLYYYYRTNQEGNLPKKGGATGQKPNFMPIEDGIKRMRQGLFAFHMETG
SGYKLVGETFEEAEKCGLQEIQFLQVVDPWLAIQKNSSYEEHLKIGLRLLHETGIQQRENN
LIYTKKTDVL

>TmolIR25a

RGNREKYKDDEEKREFNLKECLWFCMTSLTPQGGGEAPKNLSGRLVAATWWLFGFIIIAS
YTANLAAFLTVSRLDTPIESLDDLSKQYKIQYAPVNGSSTMTYFQRMADIEARFYEIWKD
MSLNDSLTDVERAKLAVWDYPVSDKYTKMWQAMKEATLPPDLETAVERVRKSKSSSEGF
AYLGDATDIKYIHMTSCDFVVVGEEVSRKPYAIAVQQGSPLKDQFNSAILQLLNRRELERL
KEQWWNRNEESKQCETSDDQQDGISIQNIGGVFIVIFVGIGLACVTLAFEYWWYKYRKNS
NITNVIVSDPKHRRVAGFPKDVGGKANEGELALRPGKLYVKPKY

>TmolIR75s

SKFVFRQPKLSYVTNVYTLPFVSKVWYSTVILTVLMALALYGLMKWEHAKNHFLLEKSL
AGRGRQNQTELRDSIKDVAFVTIGAV

>CforIR93a

MLFGVMFVICLVFNYAQADSFPSLLATNATLAVVLDREYLAEEYENVRLKIEEYLVYAKRE
ILRHGGVNVIYYSWTAINTKRDITAILSIASCYDTWRLFHNARSENVFHMAISEADCPRLPS
DEAITVPILEAGQETPQLLLDLRTSNIYKWKEIVLIYDNTINSDLLTRVIKSLTKPANKMSAS
GISLMELIDFATMDEIRLNIKYKLSTISSTRVGGNFLVIVSYKLVDLIMEYAKQLNLVDIKNQ
WLYVISDTNSKISNMKKFKRLLKEGDNVGFVFNSSVSSHMCEGGMVCHVEQILGDFILAL
DDAIVDEFEMAAQVSEEEWEAIRPTKLERSNFLLQKVKKYLVDLGACDNCTKWTLQTSE
TWGREYQTQDYQSDSIAEILPVGTWRPSDGPSMKDELFPHIAHGFRGKNLPFVSFHNPPW

QILKTNQTGDVVEYGGLVFDIIKELAKNLNFTYTVEIVKTTNINSNLSKFTNETATSAETSSI
TTFNIPRGILEKVHNKTVALGACAFTITEDNKKLINFTVPISIQLYTFLVARPKELTRALLFTS
PFKGDTWLCLAATIISMGPILFYINKMSPVYEYKGAKIKGGLSTVQNCIWYMYGALLQQG
GMHLPYADSARILVGSWWLVVLIIATTYSGNLVAFLTFPRIDTPITTLEELIRYRETVTWSIA
KNSFLDDQLKTSTDETYKLLYDEQIEIDDQKLLLDKIKSGKHVYIDWKIKLQYYMKQEFLI
SDTCLLTLGSDEFLDEKIALIVAPDTPYLSRINEEITKLHQVGLIQKWLEDYLPKRDKCWKR
KSSVEVNNHTVNMDDMQGSFFVLALGFFIACLIIASEKLWFKQVTKQRENVHEFIS
>CforIR76b
MGLMELIVSALCLNATCTDDNLEKYSIRKDRYFLLADQLKHENLRITTLMNGHLSGYEIIN
GSIVGKGVAFDIIEILQNEYKFNYTVVVPDDNVFYENSHEKGIKNMLLNGEADVAATFLPM
TYNETIRYSRSLDTAEWVVLMLRPKESATGSGLLAPFTASVWTLIIISLLIVGPILYLVVFIRV
KMCEDDRDAEFSLPACMWFVYGALLKQGSTLNPKSDSSRLLFSTWWIFITILTAFYTANLT
AFLTLSKFTLPINKPEDIVKKRYQWVTNRGNGIITFMNDQQGILQELIGPPKFQIDSTDKDIL
HSYVSKNFMYIREKTVLDYIMYDDYKEKTKEGVQETQRCTYVVTTFPITTLPRAFAFSPTF
KYFDLFDITIQYLIESGIIEFKEKEFLPDTVICPLNLGNKERRLRNSDLTMTYNIVGGGLIVAT
ITFALEFAVHSYKRNGCCCRDPKDGHQRPEKITGHFPKGNGLETKWVSPPPSYHTLFNDN
YANNYWYENNGVNKKQAFSMTPGIIGSKGSLVPYKTSIFLFQFTK
>CforIR25a
MIRLMIVWHLGVFLVFLGGSQGQTTQNINVLFVNEEGNTIADKALEVALTYTKKTTKLGL
SVNLVRVVGNRTESAGILESLCKRYGEMLESQTYPHLVLDTTKTGIAAETVKSFSSALGLP
TVTASYGQEGDLRQWRNLKENEKDYLVQISPPGDIIPELVRTLVLNQNITNAAILFDDSFV
MDHKYKSLLQNVATRHLLDQINEDDKQIGLQLRDLTKLDLKNFFVLGSLGSIKRVLEAAM
LENLFNRKFAWHVITQDKGDLKCNIKNATILFARPQIDAVYQDRLGTIKTSFQLNAEPEIEA
AFYFDLALRSFLAVKEMLTDGSWKRNNVTNFVTCDDYDEKNSPKRYGLELKKYLEKEST
EPPTYGPFKIVSNGLSYMEFQMALAAVYVRSGSSDKSLNLGVWQAGFDNNLTLNNPKVM
ANYTADVVYKVVTVEQKPFIIRDEKAPKGFTGYCIDLIDEIADILHFDYEIEAVSDGLFGNM
DENGKWNGIVKDLMDKKADIGLGSMSVMAERENVIDFTVPYYDLVGITILMKVPETPTSL
FKFLTVLETEVWLCILAAYFFTSFLMWVFDRWSPYSYQNNREKYKDDEEKREFNLKECL
WFCMTSLTPQGGGEAPKNLSGRLVAATWWLFGFIIIASYTANLAAFLTVSRLDTPIESLDDL
SKQYKIQYAPVEGGSTMTYFQRMADIEARFYEIWKDMSLNDSLSDVERAKLAVWDYPVS
DKYTKMWQAMKEAGLPKTLEEAVSRVKKSMSTSEGFAFLGDATDIKYLEMTNCDLTIVG
EEFSRKPYAIAVQQGSPLKDQFNTAILQLLNRRELERLKEKWWNKNPEKRTCEKADDQSD
GISIQNIGGVFIVILVGIGLACVTLAFEYWWYKYRKNTKITNIASEMPRHHHTHKQIRVKGS
DNVLDQGIILKTNKLYPKGRF
>CforIR8a
MFSVRNKPLLLAISLLTFVACQEIKIVVLKQHGQEDVVKVLEETLNYTLNDLRVTVSHAA
MEGNDEDYQTVCQSISGGVALILDLTWTGNDPAFHLSNNMSIPYIQSDVSIGPFLELLDAYL

DARKATDVLVIFDHDRYIDQALYYWLDSTRLRMVISNLDKKAIQRINKIRPTPNSFAIIAAT
SNVSSIFNQALKQDLLKLPERWNLVFTDFRYKNFDRDVIGDLPITVVTPDEDFCCDMLLRE
ECQCPSDFDMQSQFIYWLSELVSRVVKTMLDDNLQFPKVLKCNSSRFPEQTKQRFDEIVE
NVINESSTVIKRKGNSLTMNVKGIFEKNVNGTLEMSATYRNGEFNTVKGGALNPIKAFYRI
GITHALPWSYKEQDPQTGERYWTGYCVDFANKLSEVMGFDFEFVEPKSGTFGEKVNGSW
DGVVGDLARGDTDIAITAIIMTADREEVIDFVAPYFEQTGITIVMRKPVRKTSLFKFMTVLK
LEVWLSIVAALIVTGFMIWFLDKYSPYSARNNKKAYPYPCREFTLKESFWFALTSFTPQGG
GEAPKALSGRTLVAAYWLFVVLMLATFTANLAAFLTVERMQAPVQSLEQLARQSRINYTV
VKNSETHKYFINMKYAEDTLYRMWKELTLNASTDDTRYRVWDYPIREQYGHILLAINDSN
PVPVASEGFRLVDEHLDADFAFIHDSSEIKYEISRNCNFTEVGEVFAEKPYAVAIQQGSHLQ
DDLSKVILDLQKDRFFENLQSKYWNHSSKGNCPSTDDNEGITLESLGGVFIATLFGLALA
MITLAGEVLYYKRKGQREESKKKLQVAKVPKFDGLILPPKYTVAANKVSKIGPAGAKHAD
IEGIKIAHISLYPKARNRITQVS

>CforIR64a

MANLKNALVFNFCVFCLSSVVAFVDANLISSFFRFKHLDRNIVLTCFTKTDRADLRRKLTN
STDVMVSVLDLREFNFINSVVVKYKLGVVLDGDCRSSRSLLLKCKRYKCFDSTHYWFILR
TSNNYADLLRNVYLNIDCDVKVAYPKESNNFPRGYVIDDVYNPASDRGGELKTRAVGSFD
TSHGYHVTELGNKYFVRRNLTGVTFKTVIVLPEAFKGTLEDYLLSDRDIQINTFNRFHSRL
LHYCSDYHNFSMDIVVDKSWGYLQSDGTMDGLVGDLERRKIDFGLSPLFVKVDRAKYVF
YGRKTWNLRAAFIFRNPRSRKSYQIFIKPLTYQVWAGIIVCSILSVATQKLSYKFDRHNRCV
EYSWSFPILCAFGAFCQQGTQSNPNRFCGRIVIIFTLTLGYMVYQFYSASLVSFLLNVPTTLI
TTLEGLMEHNFKLGCDDVLYNKDYLKYSTDNLTREIHRRILLRHGNGTGFLNPEDGLALV
KRGQYAFHVDVVSGYPIIERTYDETTICELREVQMFRVKEMHANYQKHSPFKDLIDTCLH
RLAENGVLNRELIFWHPRKPQCLRSKSTIYINTGLEDFYPALFILLIGMIMSLQVLAIELLW
GHRNAVINVESVRRVFPYLD

>CforIR75q.1

MLGDLYTGRADIAGTVTFTPADRLKHFRYLVSTTKELPIKFVFRAPPLAYSTNLFALPFDKN
VWYSCGFVCALGAVFIGVIMIWENKRKLFDDCEENKPNIWDVVMMQIAIACQTHSFYQP
KSISGRIATLTILSTFTYVYVAFSARIVDLLPTTSDNIKNIRGLYESNMDMGVNDNDYNRFY
FTHRQDRTDEYWRKLIYEKKISSKNSDRQNFINATRGMKLVQTSYFAFHVELSTANNFIEK
TFTAQETCSVRMTDTIFRGDIPHLSTPKNSTYVEIFLVGFRRLFETGIHSREHRHYF

>CforGluR1

MFVVSRFSPYEWRILHISGEHREAPNPHTNHGGTMANDFTMLNSLWFSLAAFMQQGGDI
SPRSISGRIVGACWWFFTLIIISSYTANLAAFLTVERMVAPINSPEDLASQTEVEYGTLYQGA
TWDFFRRSQITLYSKMWEFMNSRKHVFVKTYDEGIKRVRQSKGKYALLIESPKNDYINER
EPCDTMKVGRNLDAKGFGVATPLGSPLRDAVNLAVLSLIEDGELTKLKNKWWYDRTECL
RDKQDSLRNELSLSNVAGVFYILIGGLFVAMAVAALEFCYKSHVEAKRAKIPLSDAMKNK

ARLTIGVGRDYDNGRYYTPANQIGSGTDQEQAHSNTHTQV

>CforGluR3

MALFEDSFFPLEAASRLPTETALIYDAVMMYSKLLRDVEESEPPLGVPLDCYAPGSWKYG

YTLMNLLKTASYKGLTGNIQFNQEGHRSSFGLQVYELREGGIIDVAYWNSSTGLNMTKNY

SPPVVEDQDSMRNKTFIIVITLTDPYGMLKETSEQLVGNDRYEGFSMDLIHELSLLEGFNYT

FTVQEDGANGKKVGERWTGMLGKVIYGEADMAITDLTITSERAKAVDFTSPFMTLGISILF

QKPSKAPPNFFSFAEPFALDTWIALAVAFFVVSLSFFLLGRICPDEWTNPYPCIEEPEFLINQF

SLTNSVWFATGAMLQQGSEIAPIAIPTRLVSGVWWFFVLIIVASYTANLASFLVTENNLELF

TDVQSLVEKAEEHKIRYGAKANGATFDFFDKSEGNELYKKIAKHMKDHPEDMPTDNKIGI

EMAESMRYAFFMESISIEYTTQRHCDLNMVGDRLDEKGYGIALRKNSPYRTRLSTAILKLQ

SSGVIDKIRKKWWEERKGGGQCTGDAEETEATPLDLQNVEGVFYVTVFGTIMGATLVFFE

YGFSILRISKKAKITFREALKQELKFFVKFSSNVKPVIGSGSEEDDEKLHEESQPKSESNKS

KTKSETPKTTITDNGHERPYGFVVPRSHGEYP

>CforIR40a

ARFCIALLSISATYVITDMYSANLTSLLAKPGREKSINNLYQLKSAMTSKGYRLYVEKHSPN

LDLLENGTGIYETLWTLMESQQKEYVVESVEAGVKMVKYCRNVAVMAGRETLFFDVQR

FGPKNFHLSEKLNTAYSAIALQIGCPFIEEINKLLMTIFEAGIITKMTENEYEKLGKQKNIVD

VEAQENDLPNTKTETKRAAKATEDNEKLKPISLKMLQGTFYLLFIGNVCAGIILASEILLFK

NRRAILRNRNKRSLALNNIKRKVIFNIRELQLKIVRAYEEFMRDEFTQILEYLE

>CforIR75c

MIRMLGTDRIDFYDYFMPYYRFRSGFYFRNPGVVRPNFYEVLKPLAARVWYAVLVTGLV

CCACMEVVWLMEQHRTDYEHSFLHSVLILIGIYAQQGVYVTPKRMAGRIILLSLLILSLLL

YNYYTSSLVSSLISTEPETLKTIRELYESKLRVGMELQPYTVTFMLDRSKYDRYLDLLNRT

KIYADGKPNFPSVDEGVRCIRDSDFAYHIESVSAYPLIAKTFDQASICDLTEITLIDSDT

>CforIR41a

MGSEMMTMHSFAMFYNMTVVPVVSNVEQWGEIYSNWTGNGVMGNLVEDRADIGAAA

LYTWENIYYYLDLSKPTVRTGITCLVPAPKPSNRWLTPLKAFAGATWFAVILVYVTGTITGY

LSELVTSLTSRSLKGFATVFTTQKVITTVIKPFINQNVTRTEMLVGTLGKYVMGMVFVATTI

LVASYSSGLATIMTVPR

>CforIR75s

MRWEIIFPVFLVFPAYGEPTFLDFLVEFIDADGGNLAVNLFECGKSDSVKMAKKFMYHGFL

TDAVFIDSVQETFPLRPGRQFFVINLSCNNSINLLNQMVLEHVFALPNRWLAYYKSEDYVL

SYVLKALKIPVMVDSDFSLIEIRSDGAARVKKIYKKHKTSDSFEVEEHGAGTNAIAVDSTF

KRRHLDVTLNACIVLTHNNSVNHLTDKREKHIDSIAKVNYVLVLALASIYNITLNFSVQPT

WGYKNNQSEWNGMMGELVRKEADIGGTSLFFTKDRVDLIDYIAMVTPTKSKFVFRQPKL

SYVSNVYTMPFDRRVWLCIAFTIILIIATMYVLAKWEWKKKKYVEFHDTSNSAELTDSLTE

IVLVAFGALCQQGASALPYSAPGRIATICSMITLMFIYVSYSANIVALLQTSSNSIRTLEDLLK

SRLQVGVDDTVFNHYYFSTADEPIRKAIYERKVAPAGKNPNFLTIEEGVKKVRDGLFAFH
METGAGYKLVGEIFMEHEKCGLQEISYLQVIDPWLAIQKNSSYKEMLKIGLRLLQETGIQE
RENILIYTKKPACTSKSSTFFSVGIVDCYFVATVLAVGLIASSVILIFEILVHVWIQSHKKYSQ
EFVN
>CforIR68a
MLKTLVPYQCVTIITDKIYDDVFQPTWYERFHGLISFFKIQFYDEDNEFLFQNGTEQSLTM
AKNDGCQMYVILVADGEQVAELLKFGDRGRLFDTKSKYVLLYNTQLFEKHLFYIWGRLIN
VVFIRRNKPKKPGNGSKKSPWYELSTVPFPFYFKDVLVPIRLDIWAQSKFRKAAELFKDKT
FDLKNQTLKVTALAHIPSTLKISGDDIGMKAANRATFRTSSPSDTNASFRGTEIEIVEAISNA
MNFRCALYEPSEMATDGDVGGNFTGLLGEMTSAVADIALGDLYYIPYVLDYMDLSVPYY
TQCLTFLTPESSTDISWKTLVLPFSGVMWATVLVCLLLISYVFHALAGFHVHINKVKEKCR
KNGKVPNNVRKTPINFALEPQIFKFGIDTKYTLMMEQYQPVKEENEPEGLYQFSEPVNSVL
YTYSMLLLVSLPKLPTGWSLRMLTGWYWLYCLLLVVAYRASMTAILSRPAPRVTIDTLDQ
LTTNKLRYGCWSEINRIFFKSSSDSVLRVIGEQFELVASSDEAVDKVSHGNFAFYENIYFLKE
ALAKRQQRYVHFYLYYANKTNI
>TcasIR40a
MRRDHGGDLVSASFDIVAGFLFEEICICFDKNTNINFLQHLLVRFVSNNIAIKLFNITTVEVQ
DKYFAFLNYQVTNHLGANTIFFSSHKFYEHVLLEINERDFIRRNLIYIFNWGRRPFSRYFVR
NIINVMKVFVITNPRNDTFRIFYNQAVPYKKHHLEMVNWWQHGVGLFNHPTLPAKYNNV
FKDFKENVFKIPVIHKPPWHFVQYGNDSIKVTGGRDDRILSLLSKKLNFRYDYFDPPERIQ
GSSASENGTFKGVLGLIWKRQAEFFIGDVALSHERANYVEFSFITLADSGAFITHAPSKLNE
ALALLRPFQWQVWPAIGVTFVVVGPVLYAIIALPNAWRPRFRVRSHARLFFDCTWFTTTV
LLKQTGKEPSSSHKARFFIIILSISSTYVINDMYSANLTSLLAKPGREKAINNLNQLEKAMAT
RGYDLYVERHSSSYSLFENGTGIYSRLWQMMNRRQTHFLLESVEEGVQLVRDSTNKAVIA
GRETLFFDIQRFGASNFHLSEKLNTAYSAIALQLGCPYIEEINKILMAIFEAGIITKMTENEY
EQLGKKKQTTSETEKELIPGVKKENRRVAKVSEDNEKLQPISIKMLQGTFYLLCIGNIFSGF
ILLAEILVYKHRKTYKHKKRRHRFVYLRKIRHSVASKFGAVVDAVRRVYRRAMHDAFVAT
LEYLE
>TcasIR76b
MGLFEIALAALCLNATCPGEEEPPEFPEVQYLAPDSNDRKTLFAQLTEQLKNENLIITTLKN
DRLSGTEKRNNTILGKGIAFDLLNILQDKFQFNYTLIEPKANVWGAEKFGVLDLLKDKKA
NLSAAFLPVLTQYSNHISYSPSLDTGEWVVLMKRPKESATGSGLLAPFNLPVWLLILLSLV
VVGPVIYFIIYLQAKLCKDDNNKVFPLPACIWFVYGALLKQGTTLNPMTDSSRLLFATWWI
FITILTAFYTANLTAFLTLSKFTLPITEPKDIGEKRYKWVTTKGNALEDTVTVNESLTELGKI
LGQPQRYLYVSDSDILRNYVHKRNWMFIREKPIVEYVMYDDYKEKTRNQIEEAKRCTYVI
TKFSVVSFSRAFAYSKDFKYKPLFDSTLVQIVKCHKCFSLLSRIQYLVESGIIKFKLREELPD
TEICPHNLGNKERQLRNSDLLMTYEIVGGGFIISAIVFIIEVIIRRQKKPKTKSLPLQNPNKH

TFEINLNNNYEKFGHFPYSSKFVTPPPPYHTLFNPPHKSDNMKKRNFNGREYWVYDSISG
ETKMIPMRTPSALLFQYTN
>TcasIR93a
MLLELVLSSAFVCVIRGDSFPSLLTTNATLAVIIDREFLSNEYEVIKHAIESYLVFAKREILKH
GGVNVQYYSWTTINIKKDVTAIFSIASCPDTWRLFRQARDANLLHMAISESDCPRLPPDEA
ITVPLITRGEELPQLLLDLRTRQTYNWNSAFILYDDTLSRDQVTRVVKSITAQYSNLRVNAA
AISFVKLETRLPMDEIRRQVKEILSSVSIKTVGGNFLAIIGYELVELLMEYAKMFGLVNTRT
QWLYIISNTHFRHKDINRFRQLLSEGDNIAFLYNNTVNNDTCTGGIQCHCEEILSGFTRALD
EAILFEWETSSQVSDEEWEAIRPSKLDRRNSLLQGIKTFLLQRGQCDNCTSWLMKTGDTW
GREYQQNGTDSGGLISVGNWRPSDGPSMSDELFPHIVHGFRKRNLPIVTFHNPPWQIIRSN
ESGAVSEYAGVIFELIKELSKNLNFTYTVELAKIGQEFSANLTKNEAQVVTNFIPDSILDMIR
NKSVAFGACAFTVTEESKRLINFTSPISTQTYTFLVSRPRELSRALLFMSPFTGDTWLCLSA
SIVSMGPILYYIHKYSPVYEYKGLSKRGLSSVQNCIWYMYGALLQQGGMHLPQADSARII
VGAWWLVVLVLATTYCGNLVAFLTFPKIDIPITTIDELLAHSGTVTWSMPKGSYLERTLKY
TTEPRFRYLFDKKVEVGNFKNMIEDIENGKHVHIDWKIKLQYIMKQQYLDSDRCDLALGL
DEFLNEQLAMVVSQDTPYLEIINDEIKKLHQVGLIQKWLTDYLPKKDRCWKNNRHIVEVN
NHTVNMDDMQGSFFVLFLGFLLSFFITIGEKLWHKYVTKKKMKIIQPFTT
>TcasIR64a.3
FQLRVLMERLFFLSVLAVIIYTTNCTDNHDIITSYIKEKSVKYATVFGCFTKKEKINLVKIISH
ICPISVFDINRLNIENRMESRHFHTGIILDGDCPSAEKFLINCGRSYLFDVKHHWLIVASSEKI
REKFNNVILNINADINVIIPEKPSNWSIIDVYNPASQHGGVLNFTRVGFYNKHDGYKIKYTG
VKYWNRKNLTGVTFKSMVVVTYSKTXKNSAYTIFQLPVPFEGTLQHYLDSDDNRDVNTF
NRFHSRLISFCRDYYNFSLDIEVSKSWGYTNEDGTFDGMVGALERKIIDFGSSPLFLREDR
ARVIDYGRNTWILRSLIKQQFRIISNWGFSAAFIFRNPKVRTSLEIFLRPLPSSVWLITGLLAI
VSIIILKLATSFERRRYVYDVETSWSISVIFTLGAFCQQGSPSTPKMACGRIATFFIFLLSVLIY
QFYSASLVSHLLNKPLTKIKNVRDLLLSPLKAGCEDILYDRDYFLHTTDKVAKELYAKKIL
GKSNSSNFHTPEAGLKLVAEGGYAFHVETATAYPIIESTFQDQAVCELREVPLFRTQPMHAN
FQKKSPFRDMFDTCFQRLAEHGLLVRERKHWHPRKPECIQSSKSIRFNVGLDDFYPALVIL
LVGIVASLLILVIEKEFRILTENPA
>TcasIR75q.1
SFLGTILTVYKQLAEKKIVLNVLTNHWKINQTKLSQHTFLVGDTLCPQFNSLLSHVSKFFC
YQNSQQTLGQIITSSXKWLVFDQNSTVNTNDLLLDSNFAVASQISNGRFHLKLCYKRAPNE
TIKFNEIGVFSNGFEYYNHFIPTRNRSDLSGVNITVSYVVTKPDYPFDVEDYRFRHLEAFSK
LSYAMVYPMLEMLNCTKKFIQRSSWGYKGANETQFVGGMFGDIQNGTAEIGGTVSFYTV
DRMSVVDYLSVTTPSDLKFILRAPPLSYVNNLFTLPFDTKVWYCLYFIVGVTVLILYVIVR
CESTYENALERRNNIDNIKPKFFDVVMLQIEAITQQGSENEPKTMSGRIAVFIVFLVLMFLY
TSYSANIVVLLQSTSANINTLQDLLNSKITLGVEDVVYSHHYFETQTEFTRKSIYEKKVAPK

NQKSNFMTTEMGIEKMKDEFFAFHVETTAGYKQIMDTFQEHEKCGLIEIDYLNVLYPSITI
RKNSPYKEIVKVNFRKIYESGIRHRQLNRIYYKKPHCVGKGGSFKSVGIVDIYFSVEIFAIG
CFMALWLLLLEVLFKKKIKFLVQ
>TcasIR75q.2
MKILIVFICLLINETTQNNFTDNLIVNTFNFIKILNVPVKISAHICWTRGKFDSLLMKLYXTV
LANTIHFIKSISDKYNTNLIKNVSPKYANPEHQLFIIDLKCNDSLSVLQQAEKFKLFKSPFK
WLLLGNSESLPNLYFGTDSQIFVTEPRSQLDDIKTIYKYSPMVPRFVQHSFDRFYTNTKRT
NLMGTTIKISYVITNLDSLNHLWDYRLQELKKKLYHFLICRNSHIDAINKLNYILVHNLMD
FLNASRQFTMQPTWGYKNSTTGLYSGMAGDLQKGLADLGGTPLFFTPDRIDIIDYIAATTP
TYMKFIFRAPPLSYVTNVFTLPFDSAVWHYCFVMVAVVVVCIYVIVVWEWKETKFEEKD
THSHIDTLRPNIFDVVMFEIGAITQQGTNAEPKSNSGRIITIFSFLTLMFLYTSYSANIVALLQ
STSDSIKNLEDLLNSRIKLGVEDIVYAHYYFENAQEPVRKAIYQQKVAPKGQKPNFMTAEE
GIRKVQQGFFAFHVELSTGYKIIGEVFQEGEKCGLKEIEYVNLIEPWLATQKKSPYKEVMK
IGMRKMHETGVQNREIRKIYTRKPQCHSGGSNFGSVGLIDCYSAFLTFGVGIAFAFLLFVM
ELIVRRYFIRREKERLK
>TcasIR75s
IVLPMINDLIEHFNKTQIILAYLCDKNGTNLLLIRNNNNTNFRRLSGSEPLFXKKLYQVNVL
SPNSRDMPYPTPPAFLTYVLDAGCSNTKQLLLLVPVITHXLIFGNNILKASEQKQFATPFKW
IVYYNNPVELSFFIDEYFTKTNILVDSDVTLATINPTSGTFDLNKIYKRKINGSIIIENIGIWGR
GLGVTDTGYEKITYKRRRNLTKTVLKSCIVITNNDSLNHLTDKRDIHIDSIAKVNYVLVQH
LSDTINASLEYSVRGTWGYKDNKSQWSGMIGELTRNEADIGGTALFLTSDRIRVIDYIAMT
TPTRSKFIFRQPKLSYVANVFTLPFDASVWASVCGLLVIIAGLLYVVVRWEWKKKDYVQV
VVFFAFWVDFPSSVFCRTNRTSRKFTILGSXVFITFGALCQQGSSSVPFSIPGRITLIFLLVSL
MFLYTSYSANIVALLQSSSSSIQTLQDILNSRLDVGVDNTVFNHFYFPNATEPIRRAIYQQK
VAPPGQKPKFYPIEEGIRKMRQGLFAFHVETGPGYKFVSEIFREDEKCGLQEIQYLQVPDP
WLAIQKNSSYKKMLKVGLRLLQENGIQEREVGLIYTKKPQCLARGSSFISVGLVDCYPAA
VVLAGGIGAALAVLILEIYVHQRFVGFLL
>TcasIR8a
MVISENLDKTTANRLKAIRPIPNNFAIVATSSNMEELLQTALDENLVTLPERWNLVFLDFQY
QQFDKKRLKNMPINLLHMDEEICCRFLQSEKCECPHDFNLQENFLSLATNTLAKILKTLTM
ENLLRADLNCDDSRYSEATRTRFYELLQQEVDSNDLVFKENFGLHVNINGVIETGDEKVAE
YNYKTGVTVLDGKKVEPITPFFRIGITHALPWSYKETDSSGNTYWTGYCVDFTEELSKLM
GFGYEFVEPKSGTFGKKRDGVWDGVVGDLATGETDLAITALIMTADREEVIDYVAPYFEQ
TGITIVMRKPVRKTSLFKFMTVLKLEVWLSIVGALIVTGFMVWFLDKYSPYSARNNKKAY
PYPTREFTLKESFWFALTSFTPQGGGEAPKALSGRTLVAAYWLFVVLMLATFTANLAAFLT
VERMQTPVQSLEQLAKQSRINYTVVKDSDTHKYFINMKHAEDTLYRMWKELTLNASTDD
TQYRVWDYPIREQYGHILLAINDSNPVANASEGFRIVNEHTDADFAFIHDSSEIKYEISKNC

NLTEVGEVFAERPYAVAVQQGSHLQDEISKTILNLQKDRFFEQLQAKYWNHSGKGSCPTT
DDNEGITLESLGGVFIATLFGLALAMITLVGEVLYYRRKSKIQNSETKKPKTVQTSENWKT
DTLMPVSLINKDKQSVTIGTEFKPVNRNRDLSEFGHITLYPRARNRITQTSNE
>TcasIR25a
MASSSAIIYRIAIYSRIATAHLNYSDFLNNVLTETHKMLKLVAFILYCTNLANGQTTQNINVL
FVNEEGNLVAEKAVDVATNYIKKNNKLGVNADPVKVVGNRTDASGLLDSLCSSYNEMIA
NSMNPHLVLDTTMTGLASETVKSFTAALGLPTISASFGQEGDLRQWRNIDENEKEYLVQIS
PPADVIPEIIRSLVLSKNVTNAAILFDDSFVMDHKYKSLLQNVATRHVIAPIKEADKIGDQL
RQLRKLDIVNFFILGSFENIKRVLDAADSVGFFNRKFSWHAITQDKGELKCNCRNATITLA
KPLIDAQYQDRLGLIKTSYQLNAEPEIAAAFYFDLALYSFLAVKEMIADGVWKRNNATNYI
TCDDFDGKNTPRRAGLNLKKYFSKEVSETPTYGPISIVSNGYSFMEFTMQISAVGVRESSS
DKSVPLGSWKAGYDNNLTLVDPQIMKNYTADVVYRVVTVEQKPFIIKDETAPKGYKGYCI
DLIQRISEILNFDYEITPVGDQKFGNMDENGKWNGVVRELMEKRADIGLGSMSVMAERE
NVIDFTVPYYDLVGITILMKLPKTPTSLFKFLTVLENEVWLCILAAYFFTSFLMWVFDRWS
PYSYQNNREKYKDDEEKREFNLKECLWFCMTSLTPQGGGEAPKNLSGRLVAATWWLFGF
IIIASYTANLAAFLTVSRLDTPIESLDDLSKQYKIQYAPLNGSSTMTYFERMANIEAKFYEIW
KDMSLNDSLSEVERAKLAVWDYPVSDKYTKMWQAMKEAGLPNTLDEAVKRVKDSRSSS
EGFAYLGDATDIRYLEITSCDLQMVGEEFSRKPYAIAVQQGSPLKDQFNTAILQLLNRRELE
RLKEKWWSKNPEAKKCDKQEDQSDGISIQNIGGVFIVIFVGIGLACITLAFEYWWYKYRK
GGKVVDVQAKHSDVATKINDGFHAKINKLYPRSRF
>TcasIR144
MQVSKILLLSSLLLNRDETSKCLDAIFKQPVVVLRGVPKNLQNFDAWKPETYLILAPNATV
LEQMLEKWSTIESFNPRAKFWLLTHWHEIKPKTLTILAKFYIVNVAIVTRTGQVFTYYPYK
YENIAQPDTKPVLLGQCDNVPSFPDKLPKFWRNTTVQVLTKCLLPYVDCSDLDQGLETQI
FDLVQEFLKFKVRRIFDKSFKFGLAKINGSYSASFRFLQEREVDMAMGSFRSVGSTQFRDF
EFSTNHMEDKLVWVVPKARPMVHWVRLVKIFEPSFWGLLVVLTVAMARVFEKMARFTD
EPMGIYRKSGFRVAVLILIGSYLKKTPKRFEMRIIFIFWIYFCMVLNIVFNSNLTNVFFGTFN
TFQVNSFDDIIKSNLEMGLTDDVMHILSQEQNWPEITSTKVISSCAFGPACLNRTIFQRNLV
CCWGERSIKFRMAKFYTTQVHYVDDHLLFFYLLFYFVKGYPIVPQISKMIVQLKSAGFVQ
FIKSKVDKLEPRQGNELTTKILTLKRLEGPFYFLLVGWVGGIMIFGYEVVTYERKRRKKVR
QEVTKILKKKKMRQNEKVKILEI
>TcasIR41a.1
TKMLFNNFCINILVNFIINNYHKNSRCLLIFTDGDFDYKGEIPTVRIKATNGSFNSYLIFNYH
GCQSVIIYTSNVTALLIKFETEIRLKMERFNERKFLIVPQNPSEDFDKFFNLKQLYFISDLLLV
LPTHNDTIFDLKTHKYVGVIDNNEPVLLDRWFSQNQSFLFGKNLYPNKLQNQLGRPLKM
ATFTYEPYSIIGNVFEQFFENDFILQGKSVGEHHGSELMSAVQFALKYNMTPVPVINEKDY
WGDIFPNWSGNGLLGNLVDDKADVGFSALYTWEFCYHFLELSKPLVRTGITCLVPAPKLS

ERWLTPLFSYSSYLWFCIILTLVIAIFVLSLVLFCYNHNKTLNLNYPLKRKTTYIHFLESAVTI
VLKPVFQQSLTLRELPIEIASKLLMGLVLLLALFLTSSYGSGLATVMTIPTYENAINTVEDFA
NSGLDWGATQDAWIMSIQNAEEQRYVKIVSKFHPISEEELFQFSKSGKFGFSIERLPFEDYA
IGDYIKEDVIDNFHLMKEDLYWEQCVIMLRKNSVLLPALDLFILKIFEAGLISHWQNEAVD
LYMNPKVQRAVKFYRQGQEHTVVKLQWSHVKGPFALLLIGLCISFIIFILELTLKKKRNQF
>TcasIR41a.2
TLGCLTMTNLNVLLQILLKTYFLNTRCIFLFTDSTIDLQVETPIVYFKVSNTLNPSLIFQHHG
CQNILIHHENASDIFVQFENLIRLNNERFNERKYIVTGHNSLKILLTKQLEYVSDLLLVVPK
QTGHYELITHVYRHQNRSKINEPVLLDVWYSQNHSFRQENDLFPNKLTNQNQRVLKIGTL
SYEPYSVIGKLTVNXSPYYLNLGKDDYSFDGTETSLVYEFVHKYNLTPSFTIMGDDLWGD
VYANWTGIGLFGSVLNDEIDIGYAAVYTWEEYYKFMDYTKTLIRSGVTCLVPAPQLAAGW
VTPLRSFSLGMWIALVIVLLSNTIVLNLLFYRNQKYHXNQLFQILLFNAFSKRFFIDSLTTAI
KLYVQQPLTLTLKRGLLKYFIVTNMIMVLFISSSYSSGLSSVMTVPRYGKSIQTVKDLASSH
LNWTGTTDAWIFSLRQVEEANYENIKNRFVVKTQNDLVTASKQYNFGFSVERLPYGHYA
VGPYIQRDVICNYRIMQEDLYWGQCTFLLRKNSVLLPLLDKLILRVFEAGLEAYWENQVK
CFGRKNMNLRDFLGCLPIHGHVCPKRHYVLYTTYXEHDTIKLTWEHVEGAFAVLVLGYA
ASIFTFVIELILDKVRS
>TcasIR68a
MIKNLLPYKCVVLISDDIYGGTFTKSWYRRFGPFITFVVIRVDEYEDLLSPFEETQACLDTA
KNEGCQMYLILLSNALQVSRLLRFGDKYRVINTRAKFVLLYDNRLFDKPLFYLWKRIINVI
FIRRYSGQKSDTKKNMPWYEITTVPFPTQITSILIPRRLDIWTKSKFRKGIDLFRDKTSDLRN
QTLKVAAFSHIPGTTKSLQEKTARTVIGNFSGTEVEILQTVSAAMNFHCELYEPVNVDVDL
WGGKQSSGKYTGLVGEMVSTNADIALGDLYYTPYILDLMDLSIPYNTECLTFLTPESLTDN
SWKTLILPFKYFRPAMWAAVLVCLLICGAVFHALARFHETISQNKSQVLEIHTKRKKIIILSI
CPEIEKLDSNLKYTKMREQYKPPRFEGQSIGLYQFSEPFNSVLYTYSMLLLVSLPKLPTGWS
LRMLTGWYWLYCLLLVVAYRASMTAILARPTPRVTIDTLQELVNSRLKCGGWGEINRQFF
KSSLDPITKLIGENFELVNDSNEAVDRVAQGVFAFYENSYYLKEALVKRQLRFQIARTTQN
QSEREMRDIAREDRNLHIMTDCVIKMPISIGLQKNSPIKPRVDKYIRRVLEAGLIKKWLQD
VMASILNAEVQSTQEEMKAIMNMKKFFGAIVALFIGYFISVVVLIVENVYFHFFVKRNPHY
NKYTRSIHHVKKAE
>BmelIR76b.1
MGLIEIVLAGLCLNATCEIETEDNAVKASVFQNLLVKANRLSLLGQDLSNDTLKITTFKNG
RLSGYSDENGNVSGTGVAFEIIQILQKKFKFNYTLVIPKSDIFLETGVDDGAKDLLENKKA
DLAAAFLPVVNAFRGTITYSTSLDTAEWLVLMNRPKESASGSGLLAPFTTPVWILIIFSILV
VGPIIHLLILIQARLCRDDANRVYPLPTCVWFVYGALLKQGSTLSPRTDSSRLLFSTWWIFI
LILTAFYTANLTAFLTLSTFTLPINSAEDIGSKKYNWVTNSANGLREIIYAESQEVMTQKKLV
DQIGDDQNFPDLTDMEILRDYVTNQKMMFIREKSVINHLMYQDYKIKIQEGIEEAKRCTY

VVTKFPVTVQNRAFAYGKNFKYKELFDREIQHLVESGIIQYKIKEELPDAEICPLNLKSTER
KLRNTDLLLTYIIVGSGLAIATSVFLLEILWRLGVNKCTRNQTRRNPEVTTDRFSMKNNNG
EIKNISPPPTYQSLFNPAMGFGYTEGVKKNINGRDYWIVDKSDGLKQLIPLRTPSALLFRYS
D

>BmelIR25a

MFEMVWKVLKIANFIYLLFVWTCSAQTTENLNVLFVNEEGNEVADKAIEVAMTYLKKNT
RLGISVDMRRVVGNRTDSNTFLETLCTTYDQMLQTQTYPHLVLDTTMTGIGSETVKSFTA
ALALPTISASFGQDGDLRQWRNIDDNEKEYLIQVCPPADIIPEIVRAIVLNQNITNAAIMFDS
SFVMDHKYKSLLQNVATRHIITPIREGTQSVADQLMQLRKLDIVNFFLLARLTNIKRILDAA
DSISFFNRKFAWHAITQDDGEIKCVCRNATIIFVKPSPNPAFQDRLGTMQRTYQLNTEPIIAS
AFYFDLALHSFIAIKEMIVDGAWKRNNVTNYITCDDYNGKNSPKRIGLNLKRYFNKESSE
VPTYGAISVVTNGQSYMDFQMQITSVGVREGASDKSLNLGTWVAGFENNLTLTDASIMSN
LTADVVYRVVTVIQKPFIFRDETAPKGFRGYCVDLIDKIAEILKFDYEITAVDQFGVMDDTG
KWSGLVKELMEKRADIGLGSMSVMAERENVIDFTVPYYDLVGITILMKLPETPTSLFKFLT
VLENEVWLCILAAYFFTSFLMWIFDRWSPYSYQNNREKYKNDEEKREFNLKECLWFCMT
SLTPQGGGEAPKNLSGRLVAATWWLFGFIIIASYTANLAAFLTVSRLDTPIESLDDLSKQYKI
QYAPLNGSGTQTYFERMANIEARFYEIWKDMSLNDSLSDVERAKLAVWDYPVSDKYTK
MWQAMKEAGLPNTIDEAVAKVRASKSSSEGFAFLGDATDIRFLELTNCDLTVVGEEFSRKP
YAIAVQQGSPLRDQFNTAILQLLNRRELERLKEKWWNKNPEKKDCAKVDDQSDGISIQNI
GGVFIVIFVGIGLACITLAFEYWWYKYRKGAKVIDVQEAPHNHHTQPKGIGLFKNKDGTG
FKPKINLYPKSRF

>BmelIR75s

MYLNSSLICILVLTFARNIRASTYVDFINAFIKDLNYPVVVTEYSCNRKDSFLLMKSLSLQG
YQINSLLADPSQVHYEENCDKKIFILRMNCLESHTILTKVNHLENFVLPYTWILYYDCSNT
AVFNIKDFEKYDMLVDSDINICHCNEDAIFIEKIYRPHKTTNFLSEFWGNWTNFDGYRDNG
LEKIPVRRRGNLKGVTLNTVLVVTNNDTLNHLTDKRFQHIDSIAKVNYVFMEHLSDYINIT
HNYSIYENWGYRINDSHWSGMVGALSTYQAEIGGTGLFMTGDRVEVIDYIAMATPTNSK
FVFREPKLSYVANVFILPFDRYVWVSTISLIVLFAIILRVILKWENNKSTKVPSTDLELEDSIE
DATFFSFGALCQQGAPAIPSSIPGRITTIMIYISLMFLYTSYSANIVALLQSSSDSIQTLGDLLK
SRLEVGVDDTVYNHFYFSQANDPIRKALYQQKVAPPGKKARFYPIEEGIKRMREGLFAFH
MEIGPGYQIVGNTFQESEKCGLKEIDFLNLIDPYLAVQKNLTYRKLLKMGMRKIFESGIQN
REVGLIYTKKPTCTSAGSSFISVGIVDCYPAAVVLAGGMVVAFLVLIVESATYYRIYLLSLL
KRKFRKNLYKESKSTTQDAWSYSN

>BmelIR93a

MSVVVLICLCLGGLVMSDNFPSLLTTNATLAIVIDREYTGDNYETIKNDIEAFLVYGKREIL
KHGGLNYFFFSWTTINVKRDFSAIISVASCTDTWRLFHIAEREHIFHMALLEANCPRLPPEK
AIAIPIVTRGEELSQVVLDLRTASIFNWKSLVIAYDETLTSDMSNRVVRSMTQRSNDDAAAT

SISIMKLRKNMTRADIKNMLSTVHRETVGYNFMAIVNYGLVGTIMEYAKSLNLVDTKSQ
WMYVISDTNKNFHDMELFQKLLKEGDNIAFVYNASTVSNTGSCAGGMKCQSDELLTGFF
KAMDAAVINEFEIASQVSDEEWEAIRPTKYERRQFLHASVKNYLSKYGVCDNCTFWQIET
GDTWGVEYQRIGNLANPQLIFVGTWRPTEGPTMRDELFIHIAHGFRGKVLPMVSFHNPPW
QVLKFNESGEVIEYKGLTFDVINELSRNLNFTYKVDVMKKLPGNINISATAREHMVSLEGY
LTNEVPPTLFEMVKNKTVALGACAVTIIDEYKNAINYTIPISTQTYSFLVARPRELSRALLFT
SPFTGNTWLCLAAAIVAMGPCLYYIHRYSPVYEYKGFPKKGGLSSVQNCIWYMYGALLQ
QGGMHLPYADSARVVVGAWWLVVLVIATTYCGNLVAFLTFPKIDIPITTIDELITHSDIISWS
FRDGNFLEYELKISNEPRFRALYERRRKYRQIKEVIAEVSSGTHVLIDWKMKLQYIMKNQF
SENGRCDFVLGLDDFINEQLAFIIAQDTPYLGIINDEIKKLHQVGLVEKWLKDYLPKRDRC
WKNRHIIEINNHTVNLDDMQGIFFVLFFGFLIATLWLGFEKIWHNYFSKRKDEVVKPFAS
>BmelIR75q
MFSMLFFIYLAATVSGVSENPYTEAVNFLTDFLLVQNKPSNVLAYICWPKGSKLHLWKKLS
EHGFLGGIKDTSDSLYYSYPSEQQVFFLDAECENSEYILNQAAEMELFRQPFRWIVLGSSD
NPTIKKLPFIIDSKVFLAERTNSSSKYHVKSIYRVTKNSSNFLENDIAKWSKPQGLSYFNELS
IPRDRADFKGSDIRISFVITHNDTYKHLEDYRNIHIDSITKLNWLLTRYLCDTINATAKPIFRT
NWGYLNRTTGIYSEGMLTDIRFSQADIGGTPAFVTWERIGVFEYFAATTPTYMRFIFRAPPL
TYVTNVFTLPFDGFVWYCCFALLVAIFIIVYLIVTWEWKDSTFKEKVSKQSLALRPEVFEV
VLMEIGAITQQGSDAAPKSNSGRIATIFTFTILMFLYTSYSANIVALLQSTSDSIRTLEDLLNS
RMSLGVLDLTYQRYFFETAEEPIRKAVYQQKIAPKGQKSNFVPMEEGIKRVQQGSFAFHSE
LSAAYKVVGDIFLESEKCGLKEVEYVNLFEPFLTLKANSPYKEIFKVVMTKMREAGIRNRE
YNKLFTKKPVCTTKGHNFGSVGLIDVYAAFLIFGSGISLSCFLFVIEILVLKYQNRKTSVEV
YQNNNASVEPEEFFLEEELFFNDEH
>BmelIR21a
MKRYILLLLLLISVYNCSKSVNRRALQKSHEKPQWLKWSEAFMDKARVDQQAYLVRLLKR
ITSDYLRDCIPIILYDRYTEIYDNLILEKLLTNFPVTYIHGQISENYTVNLKSSPEQNQISCLSY
VIFLKDVMKCRDIIGEKTTNRVVVVARSSQWRVFEFLSREESRSFVNLLVIVQSENIMGAFE
EAPYILYTHKLFIDALGTSKPEVLTSFQKNNFTRDVNLFPRKMSHGLAGHRFLISLSHQPPF
VISRGRNTDSDKVFDGIEVRIVNLLAKLYNFTTDYREATEDLVVGSTEAVSRTVEKRKSNL
GIGGIYVTQDKINRIGMTQWHSQDCAAFISLSSTALPKYRAIMGPFHWSVWLALTFVYLFG
IIPLVFSDKHTLRPLIQNPEEIENMFWYVFGTFTNCFTFSGKRSWSKANKMTTKLLIGFYWI
FTIIVTACYTGSIIAFVTLPVFPAVIDSVEQLLGGRFQIGTLDKGEWPTLFANSSDASSERLLR
YIELVPNIENGLRNVTKAFFWNYAFLGSRAQLDYIVRTNFSTKSKRSALHISTECFVPFNVA
FAFPLHAIYDEVLNNGLRMIIQSGIINKLKSDVEWEMMRSATGKLLAANSRVGGVKTLQT
DDRALTLDDTQGMFLLLAAGFLLGGVALLTEWFGGCLNLCKGVKRTVSTITLESNPRSHD
RKTPRESLDTFMFQSVKFNTSLSSNLDEEDYIMNAIDQNNVCNNVHRSSKSAHGSSNTNR
EHNRKLTKRHSMSPIKNVVIHRQAEDNETDDFDKRSDEIDAYSEMSKEIDNIFERVFGEEN

NHEDVANESIVDISNEQEQQCNE

>BmelIR8a

MNKTTMVFSQDTMLQTRFIHIVLITLSVVLEILCKNDTVVKIVLLKHLEGQNNIVEWYEN
AFTTLKTSMQYETIIVEVDDVDEDKNKICNALSQGGTVILDVSWHENDEAKDISTEMGVP
YIRIDVSIGPMLSFLDSYLDFRNSTDVAIVFEDPTIIDQALHYWIDTTRLRLNMVESLDIVSA
RKLKEIRPIPKNYALIASTQNMTRMFSLVVQEKLVFLPDRWHLVFLDFRGKYFDRAFIGKK
PINLLLPNSTFCCQLLMLEPLCICPENFNMQKQFLYIALETIVNILEEIARDELELTNNLVCN
TTTYNEQTQKRFQDLLAASLSKDNNPIARDNSTLKPKLIGSIEVGGNGSRSIVATFEEPQITL
LENKTIKPIKSFYRVGVTHALPWSYKVKDPVTDITTWAGYCIDFTAKLSQVMNFDYELVEP
KTGTFGEKTNGRWDGVVGDLASGDTDLAITALIMTADREEIIDFVAPYFEQTGITILMRKP
VRKTSLFKFMTVLKLEVWLSIVVALVVTGFMVWFLDKYSPYSARNNKKAYPYPCREFTL
KESFWFALTSFTPQGGGEAPKALSGRTLVAAYWLFVVLMLATFTANLAAFLTVERMQAPV
QSLEQLARQSRINYTVVKNSDSHLYFINMKFAEDTLYRMWKELTLNASTDDARYRVWDY
PIKEQYGHILLAINDSDPVATAEEGFRNVNEHLDADYALIHDSSEIRYEISKNCNFTEVGEVF
GEKPYAVAIQQGSHLQDDISRSILKLQKERFFESLQAKYWNHSAKGDCPSTDDNEGITLES
LGGVFIATLFGLGLAMITLAGEVLYYRRKRKIILPKTENSKLQFPNNSRVPAISKSWQTQDY
PLNFGTTFKPVNMKDKMSKEIEEMKLSHINLYPRARNRITQIQ

>BmelIR41a

MNMHVEVLVQFIVEQYFINIRCLLIFTEQPYLNYIGNSQVVRISVENDTFDSDIPFNSFGCQ
GIIINCNDPASVFKNFEQEIKLREERFNIRKYILIPKNSKNSYSVFDTNELDYVLDLILLQQN
DDSVFIWTNKYVGIDGGRENILLDKWYPGNQTFLYRNNLYPDKLSNQMGRELRLSTFTYE
PYSIIGDTIQDSKGTELSGAIIFASLYNMTPSLVVDTEGDWGVIYENWTGNGIQGNLVMDN
ADIALAALYLWENSFRYLDFSKSEVISGVTCLVPGGSTLATGWLTPIYSFSPEMWLATGVTF
VACIATLFYFNYFYSHTGYYKINRFNMQDLKQNLLFKSIFTITKLITSQALENTEFPLGIAGK
YFISLLLMYSLFLSSTYSSGLSSIMTIPRYVKSIDTVEEFGDSGLYWGGTSSAWTQSMDNIE
NPSYKKIIGRFKVLSEEKIGSLAKKQEIVIPLERLPHNNYAIVSVLKADDVKGYHLMKEDL
YSGTCIFMLRKNSILLPLFDKFVLAAFESGLFIYWQKEAIQLYMDTEVQEVINFYSKRHVT
HNVVKLKWIHVEGSFAILCFGYVVSFMIFSAEITYYYWNKNKIRYPH

>BmelIR75c

MAYRFLVYLIFLHQVNSLSNPVLDLTASVIKKSNAEASFFFVCWNNQDKIEIQKYLQDASG
VKIAFLPVCQGVVSKLNDEAFGKKIFVLDLSCESSLGILIEACQVYKLRNGYKWLVIANSS
LSDASDIWNQFHQLELRLDTDVKIAVSPTDNFTYNIYEIYKLGIRADMIVRPAGNFSEENLH
YFDENVSFYESRKNLHEVLLRIGSVAENVEVENPRTPVETQKLNKRESFSKLNCRLVTILR
EIHKFSYNSTKGSAWFGNSSAGYDGGLAKLLHERKIDISCEGGILRYFRMPYYDFWAPEY
KFRTCFFFRDPGIVKPGMAVLKPFSTATWQITVAATIAMSVVIKLAYQTEYKFLRSHNCSLF
TSVIITISVLSQQGSAIFPRYLGGRIVFVTILGFSILIYNYYTSSLVSSLLSSKPTVLSTIRELYE
SNLKAGMENQPYTTTYILQQANDIYIQQLNSNKIYKNGIPNFYLPYEGIEKVKNDRFAYHT

EITTAYPLIAKSFDQDSICDLAEINFVPTGVVGVMGPKYSEYTDLFTISLSKMRQSGIYKRE
YDTWVPKKPTCLLSSRIVSVGINQLFLVYLILIIGIFTATLILVLEMMWFKFQNN
>BmelGluR
MIFKRKCLLVVVIWTLCAFSYGFRHNFKIGALIDSKEFEQREAFDIAIEAVNKKIMFETIVEE
LNENEPHRAVELTCELLAKGVIAILGPFTEANANVVQSILDVKEIPHLQVRWNEHHENGTV
INLSPYPDTISNAYYDIIKGWNWRDFVVFYEHHDSLKRVSNLVKLFEPFEHTIVFHQLDSRR
SQGNYRQVFKEVRQSNAKNFILDCSIGTLPDVLQQAQQVGLMTHQYSYIITNLDTHTINLE
AYLYSGTNITYMRLVNPEDETLQEAAADIYKISKGRKIEAGVQLKLEEALIYDAVLMFANA
VYDMQDYLVPKLLECDDPTSTFSFGTGVINHMKSITHRGLSGNISFGITGYRTDIALDILEL
REGGEVIIGDWNSSRICQHPVCPLNVKRQNAPLPINHLGLLNKTFIVMTTLTVPYGMRKES
SDKLVGNDQYEGFGIDIIHALSEELNFNYTIVIREDKKNGDKLPNGTWTGMIGNIVNGDVD
LAITDLTVTKNREAAVDFTSSFMIVGVSILYNQAEPASPSFFSFAAPLAFEVWQLVMASWF
GIAIILFILGRISSAEWENPYPCIEEPEFYVNQLDLRNCVWFITGSIMQQGSEIELKSVPTRM
LAGIWFFFTLLMVSSYTANLAAFLTLEKPDPHFSSFEELIQVAEDRDIYFGAKAEGATETFF
KDKADHDKEGGDWKKAWKRMHDNYDKVMSSDNDAGVAMVQSDKFNYAFFAEDSLIE
YETERKCDLAQVGKRLDEKGYAIAMKKNASFRDDISKALYKMQQSNKIAELKKKWWKE
KRGGGQCDVKEESTEGKALKLGGVEGVFYVTIVGAVVAFFVAIFEMLLGTYRRSLRTKIPF
CHLLKEEFKFYIRFSETAKQVAYPSKESSLQGSRTSRSRETIYPQIWINKESSVETLRSRGSN
KSGKSRGKRNKRSKSKKCENQDSVETC
>BmelIR64a
MKVTTCVLCITAYCFIDVNGFLDVGIIKSYFTKKSTYQAAVVGCFSTNEKVQIMRALVFDK
QTITIGNIDDRDIYKVLFARNYHLWVILNADCYNVNNFLIQCGKKKYFGVKYHWLIMSTI
KNFSIVFENIDLYINADINVVYPEFNGTQKQYVIEDVYNPAYDKGGELKIKTKGYYTVRDG
YKILEDIPKFMERKNMTGVTLRTKIVLSDPFEEPLIEYILNRKNPGINPGSRCHFSIMQCCV
DYYNFTANVSTIDSWGYQTENGSFDGLVGTLYKDIVDYGSSPLMVRTDRAQFMEYGRRT
WIQRAKFIFRDPKSVSSVDIFLKPLSLTIWLLTFLSVTLGIVLLRISGYNEKDDGQHEASWSF
YIVSTIAAFCQQGASSTPHLFSGRIIFLFLLVLSTMIYQFYSASLVSYLLMTPPTKIKSVDDIV
KSDMKAGCENVLYHIDYLRYTTDGPSKELYQKKILGKNSNATFLNADDGLLLVKQGGYA
FHVDLATAYPIIRNTFSEQIVCELREIQMYRTQPMHSNVRIDSPYRDMFDTCLQRTAEYGIM
EREMTYWHPKKPACLRSSENSQVVIGLNYFYPALLVLVLGVLLGVIILIVEIYWSHKEARN
LQKTVKKPILVQFYVN
>BmelIR76b.2
MQQHDVQIPNYDIGTKEAVRRLSDPYEDIIPLLGDELARDNLRITTFKNGQLNGYTVQNGI
VLGTGIAFEIFHILQAKYKFNYTIILPEADVFFDDSNKTGSKDLLENNEADFVVAFLPILNAF
REIITYSTFLDRMEWVVLMNRPKSATSKLAILSPFSVLVWNLVIISILFAGVVMYLIAWIQSR
MFVGDEEKRNRYTLDTYLWFTYSTLVKEGYTVDPKNGSSRVLVSSWWLFSIIITSFYSANL
IVYLTLLTTILPLQTMEDIGSNHQKWVTNTANGLRVLLYTESNQTTTFGQTISKAIGDGYYS

NADDIDIIDYYVRQQNMVFIRERAQLNRVMYRYYLDQLKTTTMAESEICLYVVSKFNVIV
ENRAFAYRNDFKYQPLFDYELGRLFETGLVKHKLDANLPKTEICPLNLQNKNRQLKNRHL
SFTYVICGGCIGFSIFVLLLECLSKLCSRRHKRKKNEIKLHKNKINLETVRDKTEKRHSTAM
YHLLFDTPQVYGYSSGIKKHINGRDYWIVETDEGSKQLIPVRSPSALLFYIYEKKRL
>BmelIR60a
MITRLFILILIQVCAIFGIVTPSKGPLNYDTFNERQLFVGNHDFDCLSHIIKHHLYPVYLKQH
DMYQVVTVFVSDDLPSTAYEVQEKILQKMFGICNKTELEIISKNQEIKKDYQGIQYKTSFY
LLIIKAYDELPQIIRILKDKSSFNSRANFLVYLSEASDQIYQATRILETLNKNLILYCGVFISDD
SAGSNFYQMNHITEAPNACLSNHSVYVLDSCENGKMKNILHKYFYTGLKFDFYGCTIGVF
AMPYEPFVIDDQKGFEIEVLKDIGRSLNLSFNIVMGNFSEGWGKKTENNTWTGGFKYIYD
DMYIGIGNIDTGMEGTDAAFSFSYFYHMEPLVFVVPIAKYLPHWQVIVAIFTVEMWGIILAI
IVGFAIVFYFSSKYTKDIIDFKNNSLLSSYQIIVSHPVPRQPNHGLTRIFFLSLSIFSILIYSSYT
CSLLVYLKNPIREKQPSTFSDLEDEFGNIDYRIGGITRYKKLFNNTENTLFDLYETVNGTNN
TIIYWLRKVAEERDIWTISSSFYANYLLSENIQGITDNEGKPKIYIFKEQIISYSVSMLFRKGH
PLYNKFERIIKQFLYGGLVNRICNKYVYKIQEDQSEDIGGDTTLEELDIEHMQGAFAMLILG
YTAGFIVFIVEIVMGRRQMKKNKKFVNKRRVKFQIFKKN
>BmelIR68a
MQILLLFILYTIYVAYSQNTGEYILKDLEELDDDLGLLLQDIMLKTLQPYKCVIIITDELYTY
VFEQLWYNKFGIFIQYIMIIVEEYEDLYSPYNTTQKSLLMAKDRGCQLYIILLANGIQTTRL
LRFGDRYRTIDTLSKFILLFDNRLLDPSLFYLWRRIINVLFIKKYIGSKVSTGSIIRWYELVTV
PFPAPITNILIPRRLDIWTNSTFRKGTNLFRDKTSDLQNQTMKVVAFTHIPGVTKINSTQTTN
RAIFIKTNSENNTIYFAGTEIEILESVSKVMNYRCEIYEPENADTELWGRKSLSGYTGIIGEM
IESHADFAVGDLYYTPYLLDFMDLSIPYNTECLTFLTPEALTDISWKTLILPFKPIMWVGVLI
SLIICIISFYYLARFHIQITKLKKEYNHRKSHINSAIHGKITLHDKKKRVVTLSLYPEVEKLDP
NTKYTMMLEKYKVPKEEDDPVGLYQFSEPVNSALYTYSMLLLVSLPKLPSGWSLRVLTG
WYWLYCLLVVTAYRASMTAILSRPTPKVTIDTLQQLISSKLTYGGWGEINTEFFKHSSDQT
VTIISENFVIVNNSEDAVDKVAEATFAFYENTYFLKEAVVKRQQRYKYGTENVDTSAKNES
KIWRNIKDDRSLHIMSDCVINIPISIGLQKNSPIKPRIDNYIRRVLEAGLIKKWIDDVMQKVY
NTEVSTDDSHDAKALMSMKKFSGAIIALLIGYFLSVLLLIWEILYYNYVIKKNSNFNKYSR
NILENKIK
>BmelIR40a
MVIIPFIFALVEISFCNQHFLFKSYGIEMMRKDYGGDLANAISDILLGFPTQKVSICFDNLTG
KEFIRQLLIQLHNSSISLNLFNLSTVDVQEKYFDFLSYQTDNHFETYSFFIGRTRLYEHILLEI
DDRNYIRRNMIYIFYWGKSTINRYFLRNVKYSMKVYAVTNPRNDTYRLFYSQTSYKEHH
LDMINWWNYGKGLFHHPTLPITTSLYKDFKGRVLRVPVLHKPPWHFVKYQTNNHTNDTS
DGTIEVIGGRDDRILSLLSKKLNFRYNYFDPPERTQGTSDTESGLFNGVIGLIWRREGDLFI
GDVGLTYERSNFVEFSFITLADSGAFVTHAPSRLNEALALLRPFQWQVWPAIGITFVVVGP

VLYAIIALPNCWQPRFRVRSHLRLFFDCTWFTVTILLKQTGREPSSSHKARFFIILLSISATYV
ITDMYSANLTSLLARPGREKPIHKLTQLEEAMIKKGYKLFIEKHSSSHTLLENGTGIYAQLW
DLMEKQPAGNFLVESVEEGVIKVRDSTNYALMAGRETLYFDIQRFGPSNFHLSEKLNTAYS
AIALQLGCPYIEDINKILIAIFEAGIINKMTENEYEKLGEQKKISTEANENTVTGENQETRRH
AKAQEDNDKLKPISIKMLQGTFYLLGLGSIFSGAILVGEILVYKHHKKRKPKKKHRGLNIK
WIKIIKLKINICRLNLRRYYNNLMHDAFVSTLEYLP
>BlonIR21a
NMNKLKLFIILLPLVMSTEKRALQKSHEKSQWVKWSDAFLGQNKFDQQAYLVKLLKKIA
EQYLQGCTTVILYDVFTELHDNLILQKLLAGFPTAYIHSQITENYKTSFKASAEDHSQNTCL
SYILFIRDVMKVKDIIGDRSHNKIIIVAKSSQWRVLDFLSRQESQFFVNLLVIVKSEDVGGRF
VEAPYILYTHDLYIDALGSSKPVVLTSYQKNKFTRNVNLFPRKISRGFAGHRFIVALAQQPP
YIIMRGRNSESDTIFEGIEFRLMELLGTLYNFTLDYREATENTKIGSTEAVVKTIEKGNANL
GIGGIYMTTNKIRRVGFTQWHSQDCAAFISLSSTALPRYRAIMGPFHWTVWLAITAIYLIGI
FPLVFSEKQTLRQLLNNPEEIENMFWYVFGTFTNCFSFSGSKTWNKADKLTTKILIGFYWIF
TIIVTACYTGSIIAFVTLPIYPEVVDSVKQLLDGRYRIGTLNKGEWRYLFLNSSDPYAEKLLV
NLDLVSDIESGLKNVSKSFFWKYAFLGSRAQLDYLVRTNFTTESKRSVMHISKECFVPFNV
AITFPLHAIYGKTINDGLNLVIQSGILKKLKSDVEWQTMRSATGKLLAANSKIGSLKSLSYE
DRSLTLDDTQGMFLLLGAGFLIATFAVLSEFFGGCLNIFTRKRQDSVSTIASNPRTHERQTP
RDWQSVQYFRRCSNHTAEVHQPNNVAKVLDDSPDTNIPVAFQVEENNASKDVDKVFERV
FGQKCNHYESDDDNISSI
>BlonIR93a
MVSLLLLTYLVMHRVGVAKTDSFPSLITANATLAIVLDQDYLGENYEAVKTEVENFLIYGK
REILKHGGLNHVFFSWTAINIKREFMAIFSLASCADTWKLFRSAEIQNVLHMAISEPDCPRL
PPDKAITIPLVTKGQEVSQLLLDLRTESIFKWKSVVILYDNTLSPDMTTQVIRSLSQTTIYKE
NAIGVSLVKLPRTTTKTNIKSILSSINPKSVGKNFLAIVSYGLAAVIMEYAKKQELVDINSQ
WLYVISDTDDRYHEMGIFDKLLSIGDNIAFVYNITSNNQSCMGGRTCHIENLLTGFFGALE
GSIKEEFEIAAQVSDEEWEAIRPTKLERRSFLLLSIKKYLKNVASCDNCTYWKIQTSESWG
VEYENNDKDAQPFFIPVGIWRPVDGPFMNDELFIHVSHGFRRKTLPLVSFHNPPWQILKVN
NSGEVTEYSGLVFDIMKQLAKTLNFTIKVESIDKQKIEVNRTKLINSSIESVLTNNIPGIIIEQ
VKNRSVAFGACAVTVTSSLKQEINFTIPISTQVYTLLVARPKELSRALLFISPFTGDTWLSLA
AAIVTIGPILYCINRYSPVYEYEGIPKKGGVSSVQNCIWYIYGALLQQGGMHLPYADSARII
VGSWWLVVLVLATTYCGNLVAFLTFPKIDVPIVTIDDLIKHRETVSWSIRNGNYLESELKQS
REPQYKILYERQYKYNNRDLGVMNKISQGKHVLIDWKMSLLYIMKSHFQETGRCDYILG
HEEFCDEQLALITAHSTPYLTKINEQIKWLHQVGLIEKWLRDYLPRKDRCWKNKHIVEVN
NHTVNLDDMQGSFFVLFLGFVFALMLLVFEKIWKGHFAKKSQKIIHPFVS
>BlonGluR2
MSVAVVFVLKLLVLLRIQYVCSAKDRLSVGFLFNDLKTQSIYPLNSTMHKKIMYTMPVDY

KSVIQEISSIDSFEASKTLCNVLKTDEGVGVIFGAESSVTTPVLDSIATNFGVPYIMTSLYYP
NDQEDRYSFNFFPHADLFAKGLAEIIKHHQWTNFAILYETEEGLAKMQEVLKLQEFQKDG
KKNRIILKQLGPGPDYRPLLKRVNSTEGNIILDCKTENILPILEQAKGLKMLDLTDSYFLTSL
DAHTLDFSVLDTTANITTVRLFDYNTEQFKNAVRRWEIMEFDLNPTRSMYHIEPRSIKTET
ALYQDALTLVTDSINDISFESGIQSVPITCSGEEKSPDGRKISMRMKSRPISMSLTGPLTFDNF
GNRIDFNIYIEEAITGQSIGIWHARNESTTFIRTLNETIDAAVLNLQKNKVIVSSKIGEPYLM
HAVPEDGQVLEGNARYVGYSMDLIAGIARIIGFEFEFHLTSDNKNGNWDAHTRRWTGVIG
DLLERRAHLGICDLTITHERREVVDFSMPFMNLGISILYKKPDKKDINMFAFLDPFSITVWI
YTVTLYLVISIILFFISRMTPGDWENPHPCEEEPEELENMWGIKNCMWLTLGSIMTQGCDIL
PKGISSRLTVSMWWFFCLIMSSSYTANLAAFLTKANLEAPIDSAEALAKQTKIKYGLLKDG
ATQSFFQNSNLSLYQRMWQSMKETRPSVFVTDNKDGVNKVLTTKNSLYAFLMESTGIEYE
LQTKCELKEIGNHLDSKSYGIAMPMNAPYRSAINKAVLKMQESGELGELKKKWWKEQRS
EPSCDEQETEEEDDGGGLALANVGGVFLVLGVGIALAYGLALIEFLWNVRNVSVEEHISY
WGALKLELKFACAIWITRKRAKPLLSESSSGRSDKTDKTDNKSIEQSILKNSDSTHNS
>BlonGluR1
MNQIFTIHVFLWWVFQIHLCIGSQVIPIGVLFDKHQSQAEIPLNSTLFLGLRTYKRTQTFSA
NVHRISSIDTFEAGKTVCNAINSQDGVTVMFGATTSISTPLAESICTHFNIPYIITSWRESFYK
PSNVILNFHPDADLFAAALTKIVESLDWQGYYILYESAEGLMRLQEVLKLKRFKEKNTQQI
IHVKQLEPGNDQRTLLKNIRNCTSHIILDCKTENIVPILQQAKEINMLNDHFSYLLTSLDAH
TIDFSLLDTRANITVVRLFDFDTDTIRNTFREWELSQYEMFNRKLRVRESAVQTETALFAD
ALTYVSQALEDLATESEITTEPLDCEHDRKFNMGNSIVEKIRSINKLDTLTGPIKFDENGNRI
DFNLHLIDVRTQRKLAIWFQGNSSLTLTRSAEETSSAALSTLTGTIVRISTKLGEPFLMEVKP
KEGEVLVGNARYKGFSKDLMNGIANLLNFTYEFFLTADGQYGNYDVNKKAWNGLIGDIW
RKEAHLAVCDLTANHERQKVVDFSTSFMTLGISILHKYADKKDVKTFAFLDPFDTSVWIYT
ATLYLAVSVILFFISRMTPGDWENAHPCDENPPELENIWGFMNCHWVTLGAVMNQGCDIL
PKGISSRVAVSMWWFFALIITNSYMANLTAFLTKANLEPPIDSAEALAAQHKIKYGVYGGG
STEAFFRNSNVSLYRRMYETMKSSPTVFATSNKDGVARVLNSQKAVYAFIMESSIIEYEIET
KCELKQIGGWLDAKNYAVAMPMNAPYRSSINQAILQLQQSGELVRYKTKWWKLERNETS
CDVTQRDDTDASLTLAEVAGIFLVLGVGIGVTCIWGFTEFLWNVRNISVEEHISYWEALKV
EVKFACNIWITKKRIKPEISESSSSKSDKTDNRSIIQNFIHSASSFMNINQT
>BlonIR68a
FSPLMWTGVLICLVICILAFHYFARFHNSVTYPKTIESHKPVKSEQHTTILLIYPEIYKLDSN
MKYTLMREKYQVSKKDAEVTGLYQFLEPVNSALYTYSMLLLVSLPKLPTGWSLRVLTGW
NWLYCLLVVTSYRASMTAILARPTPKVTIDTLDELVSSKLTYGGWGEINKEFFKASFDQTI
QMISDNFILVNNSEEAVEKVAQASFAFYENTYFLKEAIVKQQNSVKFRDTNNTNTTTYKSR
TSQKENRNLHIMNDCIIIVPVSLGLQKNSPIKPRIDKIIRRVIEAGLIKKWIDDVMQNIIHTLM
QKDNKNTKALMNMKKFSGAFVALVIGYFLSIIMLLCEISYFHYFTMKNPHYNKYSRQIEIN

QNL

>BlonIR8a

MRGVCPVIAKYEPRITLDVSWLKTETNCNLFNNSSYIYLGIDASLRPFIEFLETYLKIRNVE
SVVIILEQSDELDQIIFPLLNTTRLKMYIIESMDSTSIKKIQDLKPMPTSYVLLASTTSVESFL
TLESTEHLLKLPDRWILMCTDVHGYKIDRYLLKNKLISLLTIDNKLCCVNKTFLTTCTCEE
NLNLQKSFLQLVLQNLISNLEVTFKDQLLNYNSQISGFRLDEFFNDTFLTEYSYLIGSTLKM
NISGNIEIGNNISSKVVGKYENKELIILENSTMKPIKAFYKVGITHALPWSFKINNSVTGTSI
WSGYCVDFTSKIAEMLNFNYEFVEPNNGTFGEKINGIWDGVIGDLVTGETDFAVTAITMTA
DREEVVDFVAPYFEQTGITIVMRKPVRKTSLFKFMTVLKLEVWLSIVAALVATGCMVWLL
DKYSPYSARNNKAAYHYPCRDFTLKESFWFALTSFTPQGGGEAPKALSGRTLVAAYWLFV
VLMLATFTANLAAFLTVERMQAPVQSLEQLARQSRINYTVVKDSDTHKYFINMKNAEDT
LYRMWKELTLNATTDDLRYRVWDYPIKEQYGHILLAINDSNPVANAEEGFNTVNAHLDA
DYAFIHDSSEIKYEISRNCNLTEVGEVFAEKPYAVAVQQGSRLQDDISKVILKLQKDRFFDE
LQAKYWNHSSKGYCPSTDDNEGITLESLGGVFIATLFGLALSLLTLVGEVIYYRKKRKSNT
MRQKSQKIGSGKLSLLPPPFIDKGKNPGANKNTTKQKLEKSIMNKVKEESRIKYPNLHSR
NTNKIHIMKESFY

>BlonIR76b

MGLIEVVLAGLCLNATCDVSERTQLEPSNYDTGRESLLALAEDLSHETLRIATFKNGELSG
YVNQSGQVIGSGIAFEIIDILQSKFKFNYTIVVPESGLFLAASRKNGAKDLLQKDDADMAA
AFLPVIHAFRNDIQYSFSVDITEWVVLMNRPHESANGSGLLAPFTTPVWILIILSILIVGPIIH
FIMWLYSKLCKDDHAKVYACHRSTWFVYGALLKQGSPLNPQTHSSRVLFATWWIFILILTA
FYTANLTAFLTLSTFTLPINSAEDIARKHYHWVTNKANGIREIIYEENILGTYQKKLVDVIG
NDNNFPDKNDFAILDQYVAKKGMMFIREKSIVDRVLYEDYKEKTKNGIEESKRCTFVATKF
SIVSSHRAFAYSRNFKYSVLFDRAIQQLVESGIIKYKMRENLPDAEICPLNLKSTERKLKNT
DLLLTYEIVGTGVIIATIVFLLEHLLHVTIKKCKERREAPRDHALFKKNNRNLSKFNIPSITPP
PSYNALFMPPFAFNPQQGVKKHINGRDYWVVNKNDGFSQLIPLRTPSALLFQYSQ

>BlonIR25a

MKCVIFTYVVIPSFLMCCSGQTTQNINVLFVNEEGNEVADKAVDVAMTYLKKNSRLGVSV
DLRKVVGNRTESNVFLEALCSTYNQMLETQAYPHIVLDTTMTGLGSETVKSFTGALGLPT
ISASFGQEGDLRQWRNIDDVEKQYLIQISPPADIIPEIVRTIVLNQNISNAAILFDNTFVMDH
KYKSLLQNVACRHIITPIKGGVQSLADQLTQLRKLDIVNFFVLGSLNSIKNVLNAADSVSFF
NRKFAWHAITQDDGEVKCACKNATIMFAKPSPNAAFMDRLGTMQRTYQLNTEPIIASAFY
FDLALHAFLSVKNMIANGDWKKNNVTHYISCDDYNGDNTPKRYGLMLRRGFNLESSEAP
TYGPINLISNGLSYMEFQMQISSVGVRGGASDKSLNLGTWSAGFDNNLTLIDPQVMSNFT
ADLVYRIVTVEQKPFMFRDEKAPKGFRGYCIDLIDKIAEILNFDYEIVAVDSFGIMDENGK
WNGVIRELMEKRADVGLGSMSVMAERENVIDFTVPFYDLVGITILMKLPETPTSLFKFLTV
LENEVWLCILAAYFFTSFLMWVFDHWSPYSYQNNREKYKDDDEKRVFNLKECLWFCMT

SLTPQGGGEAPKCLSGRLVAATWWLFGFIIIASYTANLAAFLTVSRLDTPIESLDDLSKQYKI
QYAPLNGSSDQTYFERMAHIEMRFYEIWKDMSLNDSLSEVERAKLAVWDYPVSDKYTK
MWQAMKEAGLPSTLEEAVDRVRASKSSSEGFAYLGDATDIKYLELTNCDLTSVGEEFSRK
PYAIAIQQGSPLKDQFNTAILQLLNRRDLERLKEKWWNKNPEKMDCEKVDDQSDGISIQNI
GGVFIVIFVGIGLACITLAFEYWWYKYRKQVKVVDIRRQSDFSSKLPFRKTNNANKDKGK
NQKLGLSTRPRF

>BlonIR41a

MSEMDIWINILSNLIIKTYFQDSNCLFIFTDKENAFQYVGDLPVVNVETKMSNLSPNIFLQH
FGCHGIIIRSDHPVSQFKSFEREIKFAKERFNSRKFLLLPGNNMKENFSDILQCPELMYVAD
LDIVELTNNDNGFIFTIWTHFYVGIKSEEKKVLDVWFSNNSTFLYENNLYPDKLSNQMGRN
IRMATFLYEPYSIVDDTVSEYKGSEMSVALTFAKKYNLTPLFVVNEEDFWGEIFPNWSGNG
LLGNLVLDIADMGFGALYTWENEYKFLDLSQSLVRTGVTCLVPAPKIAAGWLTPLYSYSL
KMWLAVGSLFLICVPTIFLLYYSHQGLNVAKNIETKPKTVTELIVVSTSIIFKLFLLQPINKN
EIPRNICGRYFVGLLFIFELFLTSTYSSGLASIMTIPRYENPINTVDEFYRSGLYWGATQDAWI
VSIQNATEHKYQEIVKHFRSLLERELRKLSVQGDFAFSIERLPGGNYAIGSYIKRDVIDNYH
LMQEDFYWQQCVFMLRKNSILLPMLDSFILRVFQYGLISYWQNEAVSLYMDPYVQRVVR
YYFVTHDKKNTVIKLKWLHVEGAFGILCLGYILSILIFCLEIFLYKISQEKGQLLYLP

>BlonIR75s

MNLRCVLLHFCIVCMNVDAAMYQDFLIDYLKTFNKPVTLTEYSCSLTDSVLLVKKLMNS
GITTRVLQAHHDEVQHENVPNSAVLLLNIRCSQAARILLTAAEMELFAHPYRWILFCENNC
TNLLDNMDISVSSDVNLCKYNGVDRITVEKIFKYNTNEDIVSEEWGYWTKTENFVLNNTE
KNLFRRRQNLKNITLNTCIVVTNNDSLKHLTDKRDKHIDSITKVNYVLVEHLSDIMNITLN
YSIQSTWGYKNNKSEWTGMIGELVVKNADIGGTALFFTIDRVDIIDYIAMTTPTRSKFVFR
EPKLSYVTNVYMLPFDDFVWASILSLLIIIITFLYFILKWEWRKQEFQEIKDESTIPELRDSLD
DIVLFTFGAMCQQGGPEIPLSVPGRITTIMMLISLMFLYTSYSANIVALLQSSSNSIQSLADL
LKSRLEVGVDDTVFNRFYFPNATEPVRRAIYLQKVAPPGKKDRFMSLEEGVRRLREGLFA
FHMETGPGYKLVGEIFEEGEKCGLKEIQFIQVVDPWLAIQKNSSFKEFLKIGLRKIQESGLQ
SREVGLIYTKKPICISRGSNFISVGIVDCYPALIVLISGMFLAFIVLLVEKLCWKRKILQKKT
KQKKIDPNKMINSSNHDFLWTYME

>BlonIR75q.2A

MEVVVQFLDQQRTSAVVIGYVCWPIGQKVTFWKLLSKHGYAATFSDEVVYHNLANHHQI
FVLDTLCPGYKNILNKAKKLKQFNYPYRWILINYKENNSFLNDFYFGIDSRVFLINEMHQE
YCIKSIYKISSKELTFKENYIGLWNNISGFTEYNDFIVSRNRSNLEGLTFNLAYVTTANDTLL
HLEDYRNRHIDATTKVNWLLLPFLFDLVNATARQYFLTTWGYKNTTTGLYNGVVGDLQS
GFAEIGGSSVFITADRLEFITYITATSDTDIKFIFRAPPLSYVSNIFTQPFNRSVWYCSYAILAL
LFGVIYLVINWEWSDPLFSETIEGIPDILRPVFFDVVMAEVGCITQQGIDKEPKSFAGRICTIL
TLAVFMFLYTSYSANIVALLQSTDENIRSLEDLLYSKISLGAEDIPYNTYYLKNAEGAVKKA

IYETKIAPKGQKPNFMSPEEGMRRVREGFFAFHTELTSGYKIISDTFHESEKCGLRAIRYLSII
LPWLPVRKNSTYKEIFKVGMYRIRESGIQRRVYLRIYYEKPICLSKGNNFVSVGILDCYGA
FMILGIGLLSSLIMGIAEILLHKFMNDKDMVIIKRSKKKSKKRNQNIDQGYNSLMRNVNIK
QVW
>BlonIR75q.2B
MDLNCDNVEDILELASRSDKFYHPYRWMFIGHFNITLFQNLRFSLDARLHVVEKLEGGDN
YAIKSFYKLAKESDIFFDNDIASWSKGSGLFRLNEFSLSKNRSNLLGRTLNVSYVVTNPSTF
NHLEDGREKEIDTITKVNWIITKHLLSTVNATSQPIIQTTWGYKNESTGLYSGLIGDLQTGR
AELGGTPSFFVIDRLEIVDFIAATTPTYMKFIFRAPPLSYVTNIFTLPFQSYVWYCTFALVAIT
FIVIYIISKWEWTDPLFKRDAATAPYTLRPEILEVALLEIGAITQQGSDFIPKSTAGRIATIITLT
ILMFMYTSYSANIVALLQSTTDSIKTLDDLLKSRISLGVEDIVYAHYYFETANEPVRKAIYQ
QKISPKGQKSNFMTLEEGIKRVQKGFFAFHVELARGYKVVSDIFQENEKCALKEIAFVNLI
EPWVPVRKKSPYKKIFKIGLHKIQESGIQKREVNRIYMKKPTCHSKGSNFGSVGLIDCSGA
FIIFGVGLALSFIIFIIELLVKRYKPNSNFKKVFPIEEAPEKWKNSNISDNEVFEYND
>BlonGluR3
MIFQWFVLVLLTFVTCDEINFASLFEQNENHLEKAFNFAVQSVNNEKLEDDPELSIIVHTDL
QPDEPFEALRHTCDILEKGTVAIFGPSSYDNIEIVQSICDAKEIPHIITRWKWPIRGGPEINFY
PHPPQLARAYFDVIKAWEWTTFTVLYEDYEGLSRINHLVQEAKDFGMLVDIKKLDSSVTG
HYRDTIKEIKLSDQKFFVLDCHIDNLDTILRQFQQIGLMNEEHNYFLTNLDAHTENLEPYQ
YSGANITGIRIINPENDLVEKISEELYADEPEISGTGLAAWKLRTEPALLIDAVHMFSQALSD
RRKQSSVPLANGSNSLNCEETKSWEHGFSVVNMVKTNVYDGLTGTIRFNTEGFRSDFGLN
IFELKEGGITDIGHWNYSSGVNLARMFPNQSIDAGDSLQNMSFTVIITLTEPYGMLKESPEN
LIGNDRFEGYGIDLIKKLAEMEGFNFTFIVREDKANGVYNPTLGKWTGMIGDLLEMRADL
AITDFTITAQREEVVDFTVPFMSLGISILFKEPKNAPPSFFSFADPFASDTWLALVGSFFFVSF
SFYIIGRMCAEEWTNPYPCVEEPEHLSNQFSLWNSIWFVTGSIMCQGSEIGPIAMSTRMLA
GVWWFFCLIIIASYTANLTAFLATENRVELFKDLQSLYENKHGVKYGAKEGGATLSFFTRA
EEGTLFRKIGDFMEEHPENVKENEEGVKRAESEEENFAFFMESTSIEYTVQRHCTLKQYG
GNLDEKGYGIAMRKNSSYRKRLSLAILKLQSNQWLDQLKRKWWEERRGGGQCQGQDES
SEADPLDIVNCEGCFWVTIYGTILSALVVILEYLIYIISVSRKSKLPFCEVFRQEMSVYIDFNS
ESKAVITKADFSKEETKSKSSEKTDKSKTHTKSRSRSRSPHSKCRSVKKRSNSKSTGNKSPL
PYGFIYSQSTERLQSTP
>TcasIR21a
MQRGLIVLKLCLTALALKSLDKRALQKSHEKSQLEKWEDKFLNRDPSFDQTASLVNLISK
VALDELSGCSATILYDKFTETSSDLLLEKLFRTFPIPYLHGQITDKYHMKVPKLQTSQDTCT
GYILFLKDVMRSKDVVGPQTNNKVVLVSRSSQWRVYEFLASEQSQSFMNLLVIAKSEKIV
SSSIARLICLALHLKFGTALAIYAPNGGKSAVYPSVIANVPKLGFRSAESVTSVITQNGANL
GIGGLYITDTRLKATDMSHIHSQDCAAFISLASTALPRYRAIMGPFHWTVWLSLTLVYLFAI

FPLAFSDKHTLRHLLDKPEEVENMFWYVFGTFTNAFSFFGKDSWSKTDKFATRLLIGFYW
IFTIIVTACYTGSIIAFVTLPVFPATVDTPEQLVRGKYTVGTLDKGGWQYWFENSTDPITQK
LLTRIDFVPDIESGLKNTTKAFFWPYAFLGSRAQLDYIVRTNFTTINKRSLLHISSECFVPFG
VSIIYNKNALYSKIIDQGVLQAVQSGIVDKIKNDVEWETMRSASGKLLAANSYGKSLKALT
VDDRALTLDDTQGMFLLLGIGFLLGGASLLSEWMGGCLHLCKGNRNQSATSIQSNYRSHE
VPTPREKLDSMQFNSFENHKIEEEIVEERNCIIHRQDDDDIEEHINRLFDFEGVFGEANPDS
RTGPEEELSFKNTTKAFFSLYAFLDSRAQLDYIVRTYFTSMNKRSLLHISSECFVPFGVSIIY
NKNALYSKIIDQGVLQAVQSGIVDKIKNDVEWETMRSASGKLLAANSYGKSLKALTVDD
RALTLDDTQGMFLLLGIGFLLGGASLLSEWMGGCLHLCKGKRNQSATSIQSNYRSHEVPT
PREKLDSMQFNSFENHKIEEEIVEERNCIIHRQDDDDIEEHINRLFDFEGVFGEANPDSRTG
PEEELSEENGKK
>Tcas|XP_971730.2
MLRAILLVLILFMSVHSYQNFQDHKDSLNVGLILPYTNFGVREYTRAINNAVSGLHRSRGQ
RLNWLKKYNFTPKNVHYVLITLTPSPTAILKSLCKEFLSVNVSAILYLMNYEKYGRSTASA
QYFLQLAGYLGIPVIAWNADNSGLERRASQSSLQLQLAPSLEHQTAAMLSILERYKWHQF
SVVTSPIAGHDDFIQAVRERVSAMQDRFKFTILNAVLVSHHRDLAALVDSEARVMLLYCTS
QEAIDILTAAKDFHLTGENYVWVVTQSVIANPLEAPGQFPVGMLGVHFDTSSSSLVNEITT
AIKVYAYGVEDFTNDLANAGRSLNTQLSCEGEGAARWNTGDRFFRVLRNVSVEGEAGKP
NLEFTQDGVLKAAELKIMNLRPGVSKQLVWEEIGVWKSWQKEGLDIKDIVWPGNSHTPP
QGVPEKFHLKITFLEEPPYISLAPPDPVTGKCSMDRGVLCRIASDADITEVDTTLAHRNGSF
YQCCSGFCIDLLQKFSEELGFTYELVRVEDGRWGTNENGKWNGLIADLVNRKTDMVLTSL
MINAEREAVVDFSVPFMETGIAIVVAKRTGIISPTAFLEPFDTASWMLVGVVAIQAATFTIFL
FEWLSPSGFNMRLSLNQSNDTSHRFSLFRTYWLVWAVLFQAAVHVDSPRGFTARFMTNV
WAMFAVVFLAIYTANLAAFMITREEFFEFSGLDDHRLSRPYSQKPLIKFGTIPWSHTDSTIA
KYFKEMHAYMRQFNKSTVHEGVDAVLSAEMDAFIYDGTVLDYLTSQDEDCRLLTVGSW
YAMTGYGLAFPRNSKYLKMFNKRLLDFRENGDLERLRRYWMTGVCKPGKQEHKSSDPL
ALEQFLSAFLLLMAGILLAALLLFLEHLYFKYVRKHLAKTDRGGCCALISLSMGKSLTFRG
AVYEAQDILRHHRCRDPICDTHLWKVKRELDISQMRCKQLEKELEAHGIKPPPPCKR
>Tcas|XP_969654.1
MFVFVIFALNWLTIRADLWSSNNPTVFNIGGVLSSNESEYYFKETIAHLNFDSQYVPKGVT
YYDTAILMDPNPIKTALNVCKYLITSRVYAVVVSHPLTGDLSPAAVSYTSGFYHIPVIGISSR
DSAFSDKNIHVSFLRTVPPYSHQADVWVEMLKHFNYKKVIFIHSSDTDGRALLGRFQTTS
QSLEDDVEIKVQVESIIEFEPGLETFKEQLSDMKNAQSRVYLMYASKTDAQVIFRDAAEFN
MTDAGYAWIVTEQALVANNIPEGILGLRLVNATNEKAHIKDSIYVLASALRDLNQTKEITE
APKDCDDSGQIWETGRDLFDFIKKQVLMNGETGKVAFDDQGDRINAEYNIVNIQRKRKQ
VTVGKFFFNRTSNKMRLAVDENNILWPGRQHVKPEGFMIPTHLKVLTIEEKPFVYVRKLV
EPQDVCTAEEIPCPHFNATQDLAGSYCCKGYCMDLLKELSKKINFTYSLALSPDGQFGNYI

IRNSSGSGKKEWTGLIGELVGERADMIVAPLTINPERAEFIEFSKPFKYQGITILEKKPSRSST
LVSFLQPFSNTLWILVMVSVHVVALVLYLLDRFSPFGRFKLANTDGTEEDALNLSSAIWFA
WGVLLNSGIGEGTPRSFSARVLGMVWAGFAMIIVASYTANLAAFLVLERPKTKLTGINDAR
LRNTMENLTCATVKGSAVDMYFRRQVELSNMYRTMEANNYNTAEDAIEDVKVGKLMAF
IWDSSRLEFEAAQDCELVTAGELFGRSGYGIGLQKGSPWADDITLAILDFHESGFMESLDN
KWILQGNVQQCEQFEKTPNTLGLKNMAGVFILVAAGIVGGIGLIVIEMAYKKHQIKKQKR
MELARHAADKWRGCVEKRKTLRASATTQRRIKSNGVNDPATISLAVDKYQRIGGPERAW
PGDSDIRQRRVEDSGGVQPVPRYLPAYTSDVSHLIV
>Tcas|XP_968786.2
MSVTNVKNIVLLTFFTISVSATGDKIPLGAIFEQGTDEVQTAFKFAMLNHNQNVTARRFEL
QAYVDVINTADAFKLSRLICNQFQRGVYSMLGAVSPDSFDTLHSYSNTFQMPFVTPWFPE
KVLAPSSGFLDYAISMRPEYHQAIIDTVRYYGWPKIIYLYDSNDGLLRLQQIYQGLVPGSES
FQVSTVRRISNVTEALQFLRGLEEQSRWEHKYVVLDCSADMAKEIVVSHVRDIALGKRTY
HYLLSGLVMDDRWESEVIEYGAINITGFRIVDSSRKHVKDFLDNWKKLDSTGSQNTGRESI
SAQAALMYDAVFVLVEAFNKLLRKKQDIFRNNMRRGQIFNNGSKGLDCNASGGWVIPW
EHGDKISRYLRKVEIEGLTGEIRFSEDGRRQNYTLHVVEMTINSAMVKVAEWSDETGFTP
VAAKYIRLKSNAQIERNRTYIVTTIVEEPYIMLRSPEPGETLSGNDRFEGYCKDLADLIAKH
LGITYELRVVKDGNYGSENHEVKGNWDGMVGELVRNEADIAIAPMTITSERERVIDFSKP
FMSLGISIMIKKPMKQKPGVFSFLNPLSKEIWVCVIFSYIGVSIVLFTVSRFSPYEWRLLHLT
GEHRDPSGQHSTHNSMANDFTMLNSLWFSLGAFMQQGCDIAPRSISGRIVGAVWWFFTLI
LISSYTANLAAFLTVERMVAPINSPEDLASQTEVEYGTLYHGATWDFFKRSQITLYSKMWE
YMNSRKHVFVKSYDEGIRRVRTSKGKYALLIESPKNDYINEREPCDTMKVGRNLDAKGFG
VATPLGSPLRDAINLAVLNLKENGELTKLMNRWWYDRTECIHDKQDAARNELSLSNVAGI
FYILIGGLMIALAVALIEFCYKSHTEAVRAKIPLSDAMKAKARLTIGVGRDIDNGRYYTPAN
QIAGANEQEQAHSNTHTQV
>Tcas|XP_966884.2
WGSWGATLGLAGLLAVAVALPPVVKIGAIFTEDQRDSATELAFKYAVYKINKDKTLLPYTS
LVYDIQYVPRDDSFHASKKACNQVQHGVHAVFGPSDPLLGAHIHSICDALDIPHLEARLDL
DTDIREFSINLHPAQHLLNTAFQDVMAFLNWTKVAIIYEEDYGLIKLRELVRSPHNGDLEIH
LRQADPESYRAVLKEIKSKEIHNIVIDTKPSNMQHFLKGILQLQMNDYKYHYLFTTFDMET
FDLEDFKYNFVNMTAFRVVDVTDLSVQEVLRDMARFQANINADSKLNSTYLQAEAALIY
DSVFVFAIGLQTLEQSHTLKLSNVSCDKEQPWLEGLSLINYINAVEFKGLSGPIEFKEGRRI
QFKLDLLKLKQHALVKVGEWRPGAGVNITDRAAFFDPGTMNVTLVVTTILEQPYVMLRT
QTNVVGNERYEGFCIDLLKEIASMVGFEYRIELVPDSKYGVIDLETGEWNGIVRQLMDKK
ADLAVGSMTINYARESVIDFTKPFMNLGISILFKVPTDKESAFFSFFSPLGFDIWIFVGGAFF
MSSFTLFTLARFTPYEWVYPQPWKRSKYLVNQLSMSNSFWFIAGTLLRQPSGVNPQVPTS
QQARLFSFMNPLAMDIWMYVFSAYVLVSITMFVVARFSPYEWHNPHPCDMENELVENQF

SLANSFWFTIGTLMQQGSDLNPKATSTRIVGGIWWFFTLIIISSYTANLAAFLTVERMITPIE
NAEDLAGQTEIPYGTLESGSTMTFFRDSMIETYKKMWRFMENRKPSVFVPTYEEGIQRVL
EGNYAFLMESTMLDYTVQRDCNLTQIGGLLDSKGYGIATPMGSPWRDKISLAILELQEKG
EIQMLYDKWWKNTGETCSRNEKGKESKANSLGVDNIGGVFVVLLCGLAFAVIIAICEFCY
NSKKNALTEKRSASAPHQSLCSEMGGELCFALRCRGSRQRPALRRQCSKCLPGATYVPAM
LDIPPHPPQPPSRPPPTNGLSAHVCPEDTSLRDRMMIPLELQHHMQPQHPLHQQLDN
>Tcas|XP_966711.2
MSHGAANPPPAISAPIFAEQITLKTTCAIFTEDQKDSSVELAFKYAVYKINKDRVLLSNTTLV
YDIQYVPRDDSFRTSKKVCRQMEFGVQAIFGPSDPILGAHIQSICEALDVPHLEARIDFEPL
SKDLSINLHPSQEHMNKAFKDLMTFLNWTKVAIIYEEDYGLFKLQELVKAPAAARTEMYI
RQAGPTSYRQVLKEVRQKEIYKLIVDTNPRNIQKFFRAILQLQMNDYRYHYMFTTFDLET
FDLEDFKYNSVNITAFRIVDVDDPQVKESLEVMEKFQPIGHAILNKSGIIQAEPALMFDSVY
VFAKGLAAMGSIKPMNLSCDVEKPWDDGSSLYNYLGDDDLRGLTGNIEFNGGKRSNFKL
DLLKLKKEEIRKVGQWTPSGGVNITDPNAFYESHAPNITLVVMTREERPYVMVKDEKNLT
GNARYEGFCIDLLKWIAGQVGFQYTIRLVPDHMYGVYDPDTKEWNGIVRELMEKRADLA
VASMTINYARESVIDFTKPFMNLGIGILFKQSSKSEPSRLFSFLNPLAMNIWLYMAGAYVLV
SITIWIVARFSPLEWKEPELHEHADGRTLEILENGFTIGNSFWFAIGSLMQQGSDLNPKATST
RIVGGIWWFFTLIIISSYTANLAAFLTVERMITPIESAQDLADQTDIAYGTLEGGSTMTFFRD
SKIGIYQKMWRFMESRKPSVFVKTYEEGVQRVLEGNYAFLMESTMLDYAVQRDCNLTQI
GGLLDSKGYGIATPKGSPWRDKISLAILELQEKGVIQILYDKWWKNTGDVCNRDDKSKES
KANALGVENIGGVFVVLLCGLALAILVAILEFCWNSKKNAQTDRQSLCSEMAEELRFAVR
CHGSRQRPALRRSCTRCSPATTYVPAALDLPHINGRRLYAGGTSTFACTSSAIRDCCGGPSN
SRPQRAATLNYRATPQCPDLAKKSATLGRNSGFCRDTCDIVQSGLQNDVPDKILWKFDCDI
GSPNNPDVVTRTLPDAIVSRTTTL
>TcasGluRK1
IWLLIFVAYNYLFCLADDKNKITVGAFFEREDVQSKAALNYAIDTTNMMQQHLKYALKTQ
ILAQNDSFYCCKLWSGLAAIFASKPIFESLSNRLEIPFILTKWRPASYSNKQTTVNFFPDSYL
FSHGLAIIVKNLQWKNFVLLYDSDKGLVKLQQILKLNNFNSGSVIVRQLGPGPDHRPLLKE
IRALNHNRIILDCDTENIIEILKQAKEVNLMESSYFNYFLTSVDAHTLDFSVLNTTANITTIRI
LDFTQXNQITRLGYKLXRFFGITLIVIXTETALIQDGIHSFITSVNTLHVTEPIVPSPMACDQ
KWSHGFRISSFMRVVIVKFWVCGPIGFDSSGRRLNFTIFVVEGNRENVVAKWRPENPEILI
YMRGENDSFDALVKNMQKSVLIISSRLGPPYLMERKPRFEGEILTGNSRYEGFSMDLIDAI
AGILGFKYEFRLAKDGKYGNYDPETKSWNGLIKDLLDRKADLAICDLTITHQRREVVDFS
MPFMRLGISILYKKAEEKDVNIFAFLEPFSPEIWIYTATLYLVVSVILYLVARMAPGDWENP
HACNPKPEKLENIWNLKNCLWLTLGSIMTQGCDILPKGISSRLATSMWWFFSLIMTSSYTA
NLAAFLTMERLEPTIDSAEALAKQTKIKYGTVEGGATQAFFRESNYSTYQKMWTTMIQAK
PGVFEKNNADGVKRVQTTKNRLYAFLMESSQIEYEIETKCDLKQVGNWLDNKEYGIAMPI

DYPYRSAINTAILKLQEEAKLTELKDKWWKKMRDEPSCPVRTLGKSSTELALDNVGGVFL
VLGVGMAVAFVLAILEFLWNVRNISVEEHMTYFEALKVELIFALNVWVTKKRTKPKINSL
NCVLXRVIKNQTRKSCLLIKNIVFINNMNKYDKK
>Tcas|XP_968606.2
MKIVYQNVIWWVFFAFYNYLSLAQDEPLTVDVVGFFDEKNGLSEIAFQTAISNLNIMKNSI
RFNPLSTVVNTSDSFENSKFLCETAEAGKVGGVFCATSAKIAPIIESVSDNLNIPAVQVAWR
PSATYTDMLVNVYPLPKLLFQGLGAIVRNLQWRSVVVFYESAENLIPLQDVLKTQDYNG
GNKYNSLMLKELGPGPDYRSALKQIQNKSEYRIILDCKTENIVTILRQAKELKLLEPHFSYF
LTSLDAHTVDFKLLNTTANITTVRIFDPASDNFQYAISNWNNYVKKMNIPGVNLDPYSVKT
ETALMHDAVHMFLKCITDLHATGKSVKPTKLSCENVDKWTPGFDIASFIKAYTHDTDGLY
STTAPISFDNLGRRTNFSIFVVEGNRDDVVAKWNPSDPEVLQFLKSEEDRNKELERKWSEG
IVTTRIGPPYLMVKEQKSETDLLEGNNRYEGFSMDLIALLAKDLNIKFRFEVLKSGQRGAY
DKTTKSWNGLIREILDRRAELAICDLTITPDRREVVDFSTPFMRLGISILYRKAEAKEADMY
AFLDPFSLKLWMYSATLYLALTVVLFFISRISPQDWENPHPCEQEPEELENIWDMKNCLWL
TLGSIMNQGCDILPKGMAPRLAASMWWFFTIIVTNSYMANLAAFLTNERSQSEINSAEDL
AKQTKIKYGTLDGGSTQGFFRESNYSLYQRMWTAMEQAKPSVFEQSNDAGVARVQNEK
NRLYAFLMESSTLEYQIQTKCDLKQVGNWLDSKGYGIAMPLDYPHRSRINEALLRLQEQG
EINRLKDKWWKEERKDPLCPKESEDQDANKLALQNVGGVFIVLGVGVALAYIVAVLEFL
WNVRSVSVDEHISYMQALKVELLFALDVRKTKKRAKPEVPESSSSSRSPSMARSFLQSAG
SFLRLDKMNQMETPGSSRHTSRPLE
>Tcas|XP_974911.2
MEWLTQLLLVLPLMKFSNTLPDVIRIGGLFHPADDKQEIAFRYAVEKINSDRMILPRSKLSA
QIEKMSPQDSFHASKKVCHLLRSGVAAIFGPQSAHTASHVQSICDTMEIPHLETRWDYRLR
RESCLVNLYPHPTTLSKAYVDLVKAWGWKSFTIIYENNEGLVRLQELLKAHGPYEFPITVR
QLGESSDYRPLLKQIKNSAESHIVLDCSTERIYDVLKQAQQIGMMSDYHSYLITSLDLHGV
DLEEFKYGGTNITAFRLVDPDGPEVRKVVREWNLSEAKNKKGEISSIIRAETALMYDAVHL
FAKALHDLDTSQQIDIKPLSCDAVDTWPHGYSLINYMKIVEMRGLTGVIKFDHQGFRSDFV
LDIIELNKEGLKKIGTWNSTEGVNFTRTYGEAYTQIVEIIQNKTFVVTTILSSPYVMRKEAS
EKLTGNAQFEGYAVDLIHEISRVLGFNYTIRLAPDGRYGSLNRETKEWDGMIRELLDQKAD
LAIADLTITYDREQAVDFTMPFMNLGISILYRKPIKQPPNLFSFLSPLSLDVWIYMATAYLGV
SVLLFILARFTPYEWQNPHPCNPNPDHLENQFTLFNCMWFAIGSLMQQGCDFLPKFSPYE
WDNPHPCNSDPDVLENQFTLLNSLWFTIGSLMQQGSDIAPKAVSTRMVAGMWWFFTLIMI
SSYTANLAAFLTVERMDSPIESADDLAKQTKIKYGALRGGSTAAFFRDSNFSTYQRMWSF
MESQRPSVFTASNVEGVERVVKGKGSYAFLMESTSIEYVIERNCELTQVGGMLDSKGYGI
AMPPNSPFRTAISGTILKLQEEGKLHILKTRWWKEKRGGGACRDDTTKTSSTANELGLAN
VGGVFVVLMGGMGVACVIAVCEFVWKSRKVAVEERSSLCAEMANELKFAMRCQGSTKPI
RKKGRPCDASTGVDDARFHPLGSYTSYGFVVNKEPIN

>Tcas|XP_974933.1

MATFSILFLILLEACASPENPKALKVAFFLNENAALDELAITSATNYINNYAATQYALNFVL
APRIYRIKKSEIYNVGNLACDALREGIAAIFGPENGEANEIIQSMALSLEIPQFQTFWNPNFA
TYAGLGTANKKEIFNFNLYPSPSVLSKAFATLVRENDWRSYTIIYENDDGLVRLQEVLKAL
SPNNPLVTYRKLGPEPDHRPVFKEIVASGALHIILDCEADHTIDILSQAKEVKLFEEYHTYL
LTSLDAYTIDFRQLGEIKTNVSIVRMLDQKVVDTVIGNWELVDSERKLKIPNKLIKVKTAFL
FDALNLFITAYSNLDQEQEMDVRPQSCDTNEISSHGYRLSAFIPLLNMTKGMLGEPISGSL
NFNSLGQRVSLKLEVLELRKDEFRVTGIWDSGTPHSIYSTITSADREKELEQQLKGRTFRVV
SRIYPPYLSRKPGIDSSVMSGNNAFEGYAMDLMKGICELYECNYVFELVPDNNYGKYDPK
TKEWNGLIRHLLDRKADLAICDLTTTYERRKAVDFSNPFMTLGISILYTKIVKEPPDLLAFT
NPLSLHVWLYMVTAYMVISMIIFLVARLNPNEWENPHPCNPNPEELENIWNIKNCFWLTLG
SIMQQGCDILPKGISTRMVAGMWWFFTLIMISCYTANLAAFLTQSRMGPTIQSAEDLAAQT
KIKYGCLKDGATASFFRDTNVTTYHKMWVAMETADPSVFETSNDDGVKRVISKKGKYAF
LMESSSIEYEVEKHCELVQVGNRLDTKGYGIAMPTNAPYRTSINQAILKMQEMGRLQRLK
EKWWKEKNKANTCKKDEDSKTDSANELSLAHVGGVFVVLVVGMSIAMVIAVCEFLWHV
RKIAVTQHVALKEVFLKELRFAMDIWCRQKPANPAANLNLSRETLRQGD

>Tcas|XP_974901.2

MPKPRHETEQKAFHLATDLINAKYKDSSIRLIPDSHLIDNYNAYTTYLTTCELLQKGVIAIF
GPSSIHSSPAIQTILDRKEIPHVETYFDRKLSRHDCLLNLHPHPSVMSQAYLEIVNKWGWRS
LVVIYDSEESLAKLGLFAASCKQRVTLSRLELDMYDTFRTSLTSIKKTGETNFILECSVDILE
AVLKQAQQVGMMTERHSYIITKLDLQTIDLAPFQYSEANITGFRIFNPENAEIMSLADQIYT
QEKYKGIPSGWLLRHQTALLIDSVDLLHQAVLDLTLSEQVVIQSQTLYCNTSNNWDSGHTI
VNYMKGQTIKGLTGVVHFDNEGFRRDFTLDILELSLGGLLRIGAWSFFSGLSLNRPPNLSK
VKIVDDANLVNKTFTVITCLTTPYGMLKETTQQLFGNDRFEGFGIDLMDELSKMLGFNYTI
IIQEDGYNGNYNQTTGEWNGLIGAILSGKADLAIADLTVTAEREAVVDFTLQFMNLGISILY
KKPKPVPPSLFMFVSPFSYTVWILLVVTYFLVSMCFFVMGRLSPSEWTNPFPCVEEPEYLIN
QFSIRNSLWFTIGSLMQQGTELAPIGISTRTGAGVWWFFTLIMVSSYTANLAAFLTVETLVT
PFSNVKELSEQTEIKYGAKRGGATANFFKNAGNDSVRSRIWHFMATHDEEMTESNDEGVE
RTEEKHYAFFMESTTIEYVIERHCSLASVGAPLDDKGYAIAMKKNSSYRNDLSAAILRLQE
TGKIAQLKEKWWKEKRGASNCGAQKSESAATPLNLQNVGGVFLVLFLGTGLGFCISFVEL
ALRVYSTTKKTDQQFRKELIEEIKFFIRFKKNVKSVKPETH

>Tcas|XP_966620.1

MRCLGLTVFLLIFPNFLGQEQDRKEIFLGGIFTEPPDVDDSVLSDEEAFNFAIDIANREYSDV
KFTSVSEESDLRTNGPFDSRLQACSLVNQQALVIFGPKNAEEIDIVQSICDNKDLAHVITRW
VYSSADFRSVINFYPHSAYLTSAYFSVLKLWNWKTLTVFYEDNESMLRLGDLLNLAKNEG
IIVTVKQLYEGLDETPIYRTTLKEAVRSGQKNFIIDCKIESLEEVLKQAQQVGLMTKDYNFF
ITNLDLQTINLEPFQYSEANITGIRILDPLNEMFHVKAGAIMRQKPNFNLTKMRTETALLID

AVSVITQVISRKLSIKEMEMTEISCNSPKSSRHGYTIANHVKTSKFDEMTGRIEFDGNGVRS
NFDLDVIELTQNGISKIGTWNMSKGLVITPHKDEDIVEDPLSLRNKTFKVITCLTDPYCMLK
ENSGQLFGNDRFEGFAIDLIHELAQMEGFNYTFIIREDKSNGDKNKVTGEWSGMIGDVMH
GVADLAITDLTITAEREEAVDFTSPFMNLGISILAKKPGNAPPSFFSFADPFALDTWIMLALA
YIAVSVSFFVLGRICPDEWTNPYPCVEEPEFLINQFSLSNSFWYAVGSLMQQGTELAPIGVP
TRMVAGMWWFFVLIMVSSYTASLAAFLANENTITLFTDVESLVQNYEEKGIRMGAKRKG
ATEGFFRGKDSETYKIIAKYMEEHPDDMVGDNKDGVKLANKETYAFFMESISIEYETQRH
CDLQQYGGLLDDKGYGIAMRKNSTYRKTLSTAILKLQSSGQLDNLKRTWWEEKRGGGQ
CLDSGDDATPALDVRNVEGVFYVTIGGTLCAIVLIFFELFLSLLKISKKYKISMREALNNEK
KAFLDFNSNVKPAPKAKSKSGSKSSGESGKSNNNTGAPTYGFIPTITKDTLDE

>Tcas|XP_966528.1

MLLLVITISLYFHKFSQAETLKIGAIFDTDDPIKERAFHHAIHQIEPIHGRTIEGLVKNVPPND
PFEAMLAACHLIESGAVAILGPTTHENAHMVQTVCDNKDIPLLDVRSVAHPQNSINFYPLQ
QILTQIYIKLLEAWNFENFVILYENDDSLIRLAELLKFYGNGHRMVVRQLDKYQNGNYRPT
LKEVWRSGATHFVLDCSTDILEEVLHQAQQVGLVTNKQFYIITNLDFHTLDLTSFQYSETN
ITGMRFIDPDSDEIQNLGLTLYRNDFTNTEFGFIEAWKVNLEMALIIDAVTMFGEVLNRLPK
DFAIPSIDCASDKAWTYGTTLTNLVKSVKYPGYTGLIQFDNFGLRSAFGLEIIELKEGGIIKI
GNWNYSDGLNINRVYPPDPPPLVEGSLVNRTFIVITCLTEPYGMRRDSEVPLYGNERYEGF
GIDLIAELSKKLGFNYTFIIREDKKNGEFDESSGEWTGMIGDVISGKADLAITDLTITSERES
AVDFSTTFMSLGISILYQKPKKALPSFFSFADPFSLTVWKLLAAAFFGASIALFILGRISPSEW
QNPYPCVEDEFLVNQLSLRNCVWFMVGSLMQQGSEIAPIAFSTRMVAGMWWFFTLIMVS
SYTANLAAFLTTESPDLPFKDVFELVQVAEKKGIKFGAKINGSTEKFFLDSKHVDEYQQIY
KYMKNHEDEVMVNDNKDGVHKAEHEDYAFFMETTSIEYETQRRCGLTSVGHSLDEKGY
GIAMRKNSSYRMALSTAILKLQEEGVLAKLKRKWWEEQRGGGLCPQGEKSTEGTPLNLK
NVEGVFCVTIIGTVLSCVLVFVEMAVHTFKKSLRVKKPFKVLLMDEMRFYFRTSAMLKPV
TAPKPEPYGFITS

>TcasIR100l

MPRKLFLWIFFLLVSCYGNLSETHLQFLKRYFVSANSVAISMLQTHHQEVKIRDLAEVISR
KLNSIGTPVVVHENHKSGSLNIIMIVWSLKILRQFLDSLVVPEEKGTYYIIILEQDCATVHSD
FAQILEQFWCEHNVLNVVVQNPCSGGTFYLFLPFEHRDNFWGSCKSWDFNEQMPNKLRN
LNQFPLKISLFLYNPTLIAKLPKGLKTNPRYHNLSASKGYGGLDGFLLRELVDYFNFDPVIV
ENLEEYGRVLPNGTAFGSLGDVVNQRVHFSINSRFLMDYGTKEIEYTFPYISDEICMLVPKS
LKVPTWKTLLKCFNTLSWVLIFVSCLCSTFAWYFVGPSKNLHKLIWQIYCFIVGIPQKIEPS
FSQFVFLLSCFFFNVTIFGIIQGSYFTEFATTSFYPDIDTLEELYESNLPVATHFWFLLDGDTS
DLMTKLKTHKIEATGDCLEQTARQRNIATLGRKSESDLIIRTKYTSRDGTPLVHIVEECHTS
LYLCGIVPKGSHFLAPFNQIITRLFEGGFTTKWYRDVFDGIISEEKPQLDETVSFNSLNMND
LQTAFHILTIGHLFSIMVLIGEVVIKGKHNKKLLT

>TcasIR100k
VTIIILIMMCLSLPKIQTCPIKINHLKEHFKQVKSARIMILQNEIIVTDWLIMELIKDNKITVT
VQKAIRNFEPFNTSNLTRFEALEFNDTIPTLQTDSTCGHLIIVKNEERLYQYLKSDPGFLILN
PRHFYAIVAMELFKTNVLREFWSLQVSNILLLDCDTSYTVLPFNGTTIRINAYTQRKLLRNF
HNYFLQVSMQPKPPTAIVKFPKPLRENPIYKDLVPFKDYAGLDGCLLKVLTQRLNMKYVI
VGNGQKYGTVLKNGTTTGTLAWIASNKVQISTNGRFLMTYGTNKLEFTVPYSSDQVCAV
VPKALKIPKIIMLAKSLTPSSWFMIFLIYVICVLIYTLMGSTGSTWTLYAIFHGFPVKIVPTSR
QSFFLTSCMLFSIIIMTIIEGSFFKTFTTTTYYKDINTLEELDESELPIAETFFSFTNDKSRIMTS
LKRKKLVINRDDILEQVARKRNIAKLERKRDIKVRLKTEFLDEEGESRLHVVEECFTTFYIG
FIVPKNSIFLPTFNNVIRRIFESGLTQKWYGDVEFSIFLEKIFKLENNIKHHSFSFDNIVSALC
VLFIGLSLALLVFFWEVTKXKQITLIYVSLIYCIISRH
>TcasIR100j
LTLVQVVICLLEVSHYDNEKFVNVYQHFTLVRYLTLTFLNDGVHRIDLNNLVVDLMSRLN
FSMMIKEKRLGKNSTTFQESDPFQGHIMVVYDVKVLLAFLEESTEVVPKARGSFAILFTSL
KCPTHYETNHALKQLWTNHGTANLIAFCDNIYVYHPFSKNDSTWGATLDYSPATETPNLF
RNFNGYLLRVSLFKRPPTALKQVPSYISNNPIYRDLKPGDFAGLDGTLLRFLSNYLNFTVVI
DESHPTHGRVLKNGTITGSLSDVVSHRVDFSANDWFLIDYQTPEIEPTVPFSYDQVCPVVS
KALKVPQWKAFFFIFDLTSWVLIFFMWLCCVFVWHVLNPFRDLSTIIWEICSVLFGNPVNV
VPLSNQHMFLGSCMVLNIIIMGIIQGSVFTDFTTTTFHKDINTLEELDEAGLKIASSAWYLD
FDTTDLIKRLKTKQIRNYIGSYKDTAFKRGMAVLGRKQDVEHMVKVEFVAEDGSPLLHVT
SECLQTFLLVSLFPKGSPFLPTFNNVITRLFEAGLTVKWYQDVTSTGTMLQQMKNFANRRP
TGLFSLNDAKLAFYALFVGYIASFVTFLTEILTKNHHNNVHNHVDVLKAQHHGQVQVDQ
>TcasIR100n
DTFWIVYQTHFLLTDYLTLHILETEDHKFELRQFTQNILKRVNKYGYFLSVRITKSSLNKRN
KSYHFPSTAYAPSQNLAKLSDDQEFYKAKRLSTDSKHGFALIVWDLTTLHLFLDQDYRTIV
PEGRGTYAIQVVSKQCDVKNEIAFTLQRLWTEYQVINVVAQTPCSCDKTHIFIYHPFVKRE
GFWGLATSHTLDQIKGDSRLISNTLSDFNGFPLRISIFPRTPTAMQTLPKLLHYNPIYRNLTW
SKGFAGLDGLVLATLAEYFNFEVVLVGSLLEDDFGKVLPNGTTVGSLADITERRAVYNAN
ERLVAYFNLDQIDFTVPYTREDICLVVPKAAKIPKWKILFQSLDPQSWCFTLFAYVSCFMF
WYNIGPSRSLPKVSWQMFSFFLGIPTKSFARKLDQVLFLIPCMIFSVVMLGVVQGSFFTKL
TLFSFYQDVNTLEEMADLELPIGAFIWNLIRDDSDVIRRLKSKSVKPPDNIFDMIAAHRNIA
TIETRARAQLLIGSKYVDDDGFPLLHIVNECLTTFLNANIVPKGSALLTVFNAVLGKLFESG
LTRKWNNDVVDSLIAEKMISVNRKRVRTKSFSLYDAQGAFFVILVGYACSVFVFLCEIVLK
XDKICYLALIINKT
>TcasIR100e
DDFWRVTKNHFLLVNSLTIQVLQTEEHQYDLNQYTVTLLKRLNSLNLLVALRMQEKFLSG
RNFPKHSVTNHTFSTTKPKFDPIGGEELTQLKRLSSDSSKGYFIVIWDVESLHNFLDEDFQV

VVPEARAXYMIHFAFTYSTEACKIVKLQVSSVLTRLWIDNNVFNIIAQTSCLCDLEVYVHR
PFVKRGGFWGLTNSYQMSEIVENPRIIANPLINFNQFPLKIGIFPRPPTVIETLPKLLTDSPIY
KNLSFSKGFAGVDGLVLGTLAECLNFDTTVITSKPNSYGYIYKNGTATGAIADVIDRRMVF
SANSRFLLIYNTDQLEFTVPYTAEKMCLAVPKALKVYKWSSMFRCFNKLTWVSIICSFGIC
TIFWYLLKWQKLVTALATIAQFLLGVPANVRPNVPQMLFLNSCMGFNIVIMGIIQGFLFQS
FTTTSFYPDINTIEEMVDSELPLRSSIFYFLRIDNSSLIHKLKSRTMAAPPNVYDLVAFHRNIA
TTDIKSHVDFMVRSRYLDEDGWPLIHTVDECFETFLIANIVPKGSAFLTVFNNVITKLLEGG
LTQKWYEDVINSLILENWINLNRNKSKTHAFSLYDLQVAFYVIIMGCAVAILVFVAEIVHKR
RNXNNCCNNHHKNIIFAA

>TcasIR100f

DDFWVIFSTHFLLATSLTFITVQTNSKQYDLRLLAQAIIQSMDKDQVMTTRHVILHNYAEN
INFNVVFKTGTKKNARDFVTDLLAKTKKLASDSREGFVIITWNVNVLQKFLAQHISEINPR
TRATYLFILISSSDSLRKIKHCLHFLWHKYDILNIVVHVLGCGTTTTLIYRPFCKTKNSWGEI
TAHQIEEIVQQPLLLTNSLQDLNQYPLQVSLFARDPTALTQLPKLLQNNPIYKNLASFYGLD
GSMLSTMAKILNFEVVIVENHDRLPFGRVWPNGTASGTLGDVVNRRVALSSNSRILADYN
TQEIEFTVAYNGDSICVAVPKSLKVPKWRVLFECFDAASWLLTLSVFIVCLCFWYCVALKN
FARILWDVYSFLMGIPTRIVPSRQYFFLSSCMVFNVIILQLLQGWLFTAFTKTVFYPDLDTL
EVLEKTNLPVATNMWFLFKDNSEVIQKLSSRGIGKTPNSLDLVAYSRNICVLDKRQDLELY
SQAKFVGPDGLSLLHIVNQCLTSVLLVNIVPKGSPFLPVFNDIMSRLFESGFTKKWYSDVV
TSRVTEKMVSLGRKERNFSFKIKDLQAAFYVMMAGCVFSLFVFVGELVTHXVFVMNKSS
QSKSHRFLLNCNYGV

>TcasIR100g

TLFKIAEVTFFMVTMHEEFLSLLFGNYYHTNLYQTVKIQEKFARTNNKTGAWYENVALDQ
KLDPPIDQNWQRVKLRTSDSFEGFIIIVWDPQTLDQFLNQNFSLVVPRARATYFLLFVFSIY
ENCKLVNHILKRFWSEFSVLNIIAQTPYCCNKVYIHRPFVKTTNSWGVTQSYTLTEVTQNL
ALITNPLLDLNQFPLRIALFEKNPTAIRKLPKALQNNPIYRNLSRSKGFAGSDGFLLSAMVE
YLNFDPLIDETLEPMNFGHVLPNGTVCGVLAEVVHKRTDYAGNCRLMTYFGTDGYEFTA
PYSSEKIAMVVPKAGKVPRWRSLFNCFNALSWSLIFSIAIVSTVFWCFLRRSQHLKRASWE
MFAHFVGIPCRVVPSRGQFMFLTACMMFNIIILGIIQGSFFTDFTTTSYYPDLNTLEQVLDSN
LPIMAFAWRLLRTNSSPILQKLEQRSIPYEDNVYELVALYRNVAALDRRLDLELEIKTKYSG
RDGVSPLHIVDESLVTFLTTSLVPKGSPFLVVFNHVIRSMFEAGLTAKWYDDVVTSLIIEHK
HKTPSFGVKYRPFTLQDVQAAFYVIAFGYSCSVFVFWCEIIVKFSGKIKHFHYYFVLI

GRs

>PstrGR2

MLKFKKMKNTVHAPVINREKEINLHTTHGCLRNLLILGKISLVFPVEGIEGKDPGKIQFKF
VSLKTFLSVIFLISTSTCFLLSIVKSLTSRNTMQRLISPLYYCHIASVAFLNLHLSRKWKRYV
MSIHAAELNFLKNYKPDKKLKRKIHATIAIVILVGLCQYSITEANFAISHDCLNEGAGFEQLI

RKQYHYIYDYINYNVFNGVFLLILSFASTLAWNFGDLVIITISIIYTSRFNQIVDKIKWYTSY
KNRNASIFSKEIEKLHDTFWRDSRRDYARMQLLCSTTSDLFGILTLITYSFNTMFILIQLFVS
LRPREYLIEILYFISSFSFVVGRAVMSSIYGGWLYEAGRAALPIINAVPTEMYTEEASTFISQI
QMSSPYLTGRKFFTITKGIILSIAASIITFELGLIQFNQSEIERYLSTSNVTICY

>PstrGR9

MLLDHSKWIGILGVFPVYKRNLYFCMYILLRIAMCFCCLVSTHVYFKRYLDNYEMEFGNF
FFLKLASIGYELINYVNLSQVVHFREPGRALYENIQQLRFAIKKSNYTREIAGFYLWIVVFII
CVLHGSIGFVYEEWSISFWAFNAALTYLQLVGTALLLYDLAVIFTENNDYLSIRIRRSFTSY
VRNRFETQIELDSIVKCVTRMGESTGLISDFFGISIFNFVFVAFLENLFNLNWVLSAMISNTR
DIKDVADVILFVLQILIIIVLNVSSLIMVLSCDKIEAKSNELLKHCYCLQPDVKDPILLKQLID
LTDYIKELKPKMWISGFFRVNKRLIPLMISTLASYLIIIIQFHKL

>PstrGR11

MIVEDLYVSTPTFEAIQPLLITSRLFGLFPISYKKNGTNYRLKWSLVYAVYSYSLCVGLALW
TMIGMSLDLKNDPEHSLRMTDEKTRFVTGGDVSIVVIIVIFATATLHLKIKKFWKLMITLNQ
ADSIIPLRNSKRYRDASILFIAVVVVTLSLILTFDISSWFVKLHARGLNPLDYLQYYFAFYLN
YSIMIMMEVFYWHVIYLIKIRISLLNGDLRKVKEKINFKEGNYFFEKIVGKVYLDRFSRSSS
RQTACLSSTGTIIKNEENSELGKRLIMLSILHDKIFEAVTIVNNSIEFCIHVVMLSCLLHLIVT
PYFLLKGIIKENSGTYYVLLQSAWLIGHIGRVLIIVEPCQYCINEYKKTSNLICELLTYEVDN
EVKKAMTILSLQLSYCKLRFSSCGFFKINRSLLTSIAGAVTTYLVILFQFSDT

>PstrGR13

MCEKSAKNTENPDGITDPHSPRKKSTVKRFISGILMGSFFFAIVYFGVPTMYMTCFGLQIK
CVDEVLKIGYNLKKIQEIPFFHALNWYLLMVANYFLFGETFIQHGRVYLRKYYLLDMLSS
YHRFISFCFYFIGLVWFVVILRKKILRQQFSLLFWTHFLLIVITAQACMIMQAMYEGIIWLI
MSIWLVSINDSCAFIFGKHFGKTPLIRVSPKKTLEGFILGGICTIILGAFLSHFLCHFKYLVCPI
KFASVDNEITYRSNCTPSYNFQPIAYAIGDFSVNYYPFMKHSFYLSIFASVIAPFGGFCASGF
KRAVGVKDFADTVPGHGGILDRGDCQYLMMTFVNVYIMTFVKDPNVEEIFRKIAKLSDE
QQLEFYSLLRSSLSDLLAGDVGNTSKLTLYGSYF

>PstrGR15

MDEPDRNSEPYNSLHHRNPHQRTNFQRQNLPVIEVETPTEDEMQITEAEILHFYDDFYHTT
KSLLVLFQVMGVMPIERELGHTTYRWTSATNVWAYFIFAIETIFVSIVFQERLKLVLLPGKR
FDEYVYAFIFLSILVPHFLLPLGAWTNGSEVAKFKNMWTKFQYKYYCVTKSKLVFKNFTVI
TYSLCVASWVIGIMVMLAQYYVQPDMLLWHTYAYYHILAMLNCLCSLWFINCMAKGQA
ARWLSQNLHNALQSSDPATRLSEYRDLWVDLSHMMQQLGKAYSGMYAMYCVLIILTTVV
ASYGCLTEILDHGLSFKEGGLFLISVYCMTLLFIICNAGHQTSNKMGPEFRERLLNVNLGA
VDERTRQEVNMFLTAIDKNPPIMNLNGYANVNRKLISSVITSMATYLVMLMQFRLSLMRN
AAIAAKRAAIANLTAHHT

>PstrGR19

MSQDDDVVKATLPVWVFCQMFGLCFSSTKAIASTKIPSRKRNVGKFVYWFATVSIALHLS
FYNFSYELDPSKNVVIQAGDILNGLSSIAATVSSIFTSFLFENRILTVMKDLCEVNDKLKKY
PKSISYKGIKKFAYAEIIYLLNSWIYFLISYVITCPSNLAECLGSWYLMYTITKIFDVNLARFI
SHMSIVYKQLCTLNSNILHLSEERYVISLNLPKERYKIDALENFKEIYSEILRIGEEINYVHSF
SLLLIIANQFISIFSSLYFCFFGYYINGNYIKPNSLSTLLVPLMSLIHPGGQLLAIAIVCQLTISE
SKQTGKIIYRIPINRNARTLMQRVSM
>PstrGR23
MDEARMSTVKADVNILTFFFKMGKFFGVVPLHNKKSSRLKTIISLSYFAILSLLLSTIFIVSV
WDRHKMYDSMKVTNIIVDVLVMITNFCFILTIRLGNLINNRYFDKIMESFRKIDACLDQSN
FTICRMRVFKIYFFIVGFHVLYLGMHAYELYYRIVNHQTSFLIWVLYIPGFLKIHHQLFVVT
LVAKMNTMLHTRYQFVIHCLGLNIDKENLNLELLELNKKSLTLDQIYYVFKKLYDLVSDM
NVLFNWQIFFVLTCTVLEILNVINFMKQDQAWSVLAVDIAYGALYTISTLAIIKSCNDVQM
LGEKVYVCCYTSQETLGGSKFENELLKFAKLIKPLLPQYTAGGFVTVNQRILSSLFSATLTY
LIVIIQFDLST
>PstrGR26
MTLYNAVVSFDEVMYRKLLKTARYMGVVPFSLKKRRISAIFAWLLIFMYVSTEIWQLKLR
VTYHRPFVNFMVVLSDIFLVINENIAMVTAFFTCLAHKKQWSTFFYRMSIITPFIRERCEIGF
RAKLAGYTQISLFGCCVVNEFFQIYYTKELAESITPYLMFFISNIYVIFACLLLTKIVNVILKF
QRNINQDISLLEEKKYLICDSLIKMFESKLILISKTIVDINTLFGWEMFCVFVNFVIIVMNFIT
TSFFLQEDWNRKSITYFVGNFFRVVVYLVNVMMIVYKFSEIGKQNEDFIVTCFYLQEDMP
NLHIRNELLHLATFAKKIIPKLTAVDYFQVNKLMMGGLFSTITTYLVLCAQFNVGST
>PstrGR27
MDMKRKARKEDIIWDRSIHNVFSWLFFAMQLFGFMPLRGIFKRDLSAIKFTWMSWISLYA
LLTSAGMFFMLATQVARFFIYNMHTQEMQRFFYFMKSWFVSWFFFVLAREWKDMLVRW
NEVDEALARFGRPKCAKRKLNFGMVFFVAFFIGDYVLIQTQRTEQDILKHGVILSWKAWE
HFQKHRIFKYIYELYPYNFFSSLCLLIIQLQVLFSGTFLDLFIMGTSFCLASRMKLITKKIKE
MSFNRIESDEVWINLRESYNKLELLCCYINSKIGVAVLICFMGNLGILLIHLYNSIKVHGVIE
RSYLYYSFLFLLSKTIAVCIFGAKINDESQKPLSYLFTIDNYAYNVEIKRLTHQIGKNTMALS
GMNFFYLRRNILLKIAGSIVTYELVLVQFAEDILKETHSANRTTTL
>PstrGR29
MDGSSILSTMDFHHRRIKDTYFVDFWYKAGIALGLFPWTLQKGFMKHQIIALAFLFLAFSI
LSVYIIIDKENCGYGMGLLIFTSLDNAAHGLFNLIILYDLVIERKRWRTIKELFHYLDKNLN
YTNYVSDTYITFYIIGSILHLLLNIYANWHFFFKNSSSVGIVSGVIWNLYQMQKFITIGYLIN
VSLLLKRRIEKCCRILENLVKNPYQQKTEVAYKLMSLKNYKYYVLIVWNLNSLFGRKLLL
FLVMAFITILDEVQFILDIDMSDIKIAIVLENLTELILIMVLTIIIAIAGDIIEKSGDKMIKLLNIL
QLSIEDDFLKQELSKFAALVTELRPSLSVAGFFDVNRKLLPMLLSSFSAYIIILIQLKQ
>PstrGR34

MSMTSNVLFTIVRLQLALTNVTSIVTLVLVKRRRLVGKTRRLGNVERTLQANFQRTIDYKP
TVLTAQLSLFIGLILCIGIFDVYVTASSLQYDAFDAAIHSLYRLQVFIVALTTAQVRCCCARI
QAAVEFANERMRDTLRTVAGEATFFLRRYDELCDAVDAVSDVYGAAMLMIVGCVVANLL
AGSNALLKLLTVVRRDDALLLTSLFGIVEYAVISVMLAVPCGNASNEARKTCLVSYKILLG
FQGSSRFGRCKNVKEELLLLAEHVRARNVRFSTGFFPVDYNVLFLILGSVATYLIIILQFQ
>PstrGR36
MAAVERQDAAGALNRLHEGTKPVVRAAQLFGVMPLRCIAGDWDGVRFEWRSLAVAFSA
LNAAGAFLSMALWLVKFCVDGIVVDKTVYMAFYFCTFLCTVHFMKVARNWPSILKEWSF
VETTMKNYGTEENLKRKFLWMGGVSFLVGTVEHILFIINGLYQSEVCDSFSRSRFRATMEV
LFSNYFTFISFNTIAGVLIKMINVLATFTWIYTDLFISIVSHALTVKFRQFVTRIKTNPDRVGH
QKFWREIREDYQKLYGLCKKVDKCISFLVLVSFMHNIFFLCIQLYNSLKQRKGLIETAYFVY
SFAFLVFRIIAVSMYGALLHDEAQKPLEYLHNVPTEYYCSEVRRLINQIYTCPVGIGSGFFV
VSRNFMLQIAGTIVTFELMLFQFAPIDSKNRIYNKTE
>TcasGR3
MYHQDQAVSILGEAIPKRRSVFLESGVNSADSFKASKVGPAPPIKFINKSSTDKFGNGAIYE
VLKPIYALMRIVGIFPIKNTEPGMFRVAPELLGYSVVVFVVVMGYIGFIEWDKVEIVRSQEG
RFEEAVIDYLFTVYLLPIIINPLVLYEARKLANVVTDWVNFERIYYKLTKKKLSVFFGNKPV
ILTVVLPLLACGVMVVTHITMAHFKIIQVVPYCYINCLIYLIGGFWFMQCDVVGKVASQLA
EDFQMALKHVGPSSQVADYRSLWMLLSKLIRDVGNASGYTVTFLCLYLFLIITLTIYGLLS
QLQAGFSTKDIGLTINAGLAIFILYFICDEAHYASNCLRVQFQKKLLLVELSWMNDEAQQEI
NMFLKATEMSPTDISLVGFFDVNRNLFKSLLATMVTYLVVLLQFQISIPEEASPTNSTTITTQ
TPN
>TcasGR2
MEISDLAQLYGNELHIKQISKWLRGSARAQEIQKRSELDSKDGHVIDEHDQFFRDHKLLLV
LFRVLGVMPIQRGEIGRITFGWTSIPMLYAYVFYVVTTVLVVLVGYERFDILLNKSKKFDEY
IYSIIFIIYLIPHFFIPFVGWGVAYEVCDYKNSWGGFQLHYYKITGKNLQFPLLSTLIIIISLGC
LILAVVFLLTLSALLEGFTLYHTTAYLHIITMINMNCALWYINCRAVGNASTALAESFQNDV
DRNCSAYIIAHYRVLWLSLSDLLQKMGNAYARTYSTYSLFMMANITVAVYGFTSEIVDHGI
RFSFKEIGLLVDSTYCLFLLFVFCDCSHQASLNIARRVQVTLLQVNLSQVDPATRKEIDIFLV
AIQMNPPKVSLKGYTVVNRELVTASVATIAIYLIVLLQFKISLLNMRG
>TcasGR1
MRNDHGSNTHLHPDDAIRRAKIVKVAASPTSANPDEEPDPELLDRYDNFYQTTKSLLVLF
QIMGVMPIERSGKGRTTFRWLSSTSIYAYFIFGAETIFVTMVFKERLYLILRPGKRFDEYIYG
IIFLSILIPHFLLPVAAWTNGTEVAKFKNMWTRFQLKYYQVTGTPIIFHNLTLITYSLCVISW
AVGIGIMLAQYYLQADMLLWHTFGYYHILAMLNCLCSLWFINCTAKGRVAVWMCNNLH
KALESRNPAKILGAYRDLWVDLSHMMQQLGKAYSGMYSMYCLLILLTTIVASYGSVTEIM
DQGISFKEAGLFMIAFYCMTLLYIICNEGHHATRKMGPEFRERLLNVNLSAVDQKTRQEV

HMFLMAIEKNPPIMNLNGYANVNRKLISSTVTSIATYLVMLMQFRLTLMRNAQLAARRAI
ANVSVSSGNTTMS
>TcasGR20
MSAMSTVIVSGEMFDLFSPVLYLSRIFCLQPLKWIKTDYSYVISKSGPYMAYSILTSLFLITA
SIYGLTQVYSMEAVYLIRLSSNTDRFVTFSDVVVVLIPCIIGVPIASHNIEKTIKYFSFLRQFD
CCLQKQPPKTWKSFFVPIITIVFTATILLFDAVMWLTLIARNQSIFILSLPYYVCYCFTMIIEI
MFWQFVNSIKIRLILLNERLEQIGGEDFTIVRIDRKKINITNEQVRDLIIAYQKLTDAANLVN
DSFGLLILIVISGCLVHLLATPYALYAIIFTTGNTMFIITQSIWMTGHVLRLLLIVEPCHGCILV
AKTTTQLVCKLLCLDLDKEVKKSLEFFMTYLAECQIRFTAYGFTKLHRGLLTTITGAVTTY
LVILFQFN
>TcasGR21
MTITISKELFHVLSPVLYLSRFFCLQPLKWTKTSAGNYIITKSRFYTIYTLAASCLLVITSITG
LSQVYQLDVIYLVRLGDTTRRFVTYSDIVVVLLPCVIGPVFALFKTNQTINYLSHLKQFDSL
QNQPTKSTKIFQITALTTFCTAFTLSMDLFLWLKLSHNYIFLLCLPYYISYWSTVVIELLFW
HFVHLIQIRISVINKKLAKMVVTGLNSVTTLKKPHAEVEDLVKGYEKLIEATNSINYCYGF
PILVIILGCLIHLLVTPYGLYSIIMSTGDSTSILSQTVWMTAHILRLFLIIEPCHECFIKTKETSQ
LICKLLCLSVNQEVKKSLEFFLTYLGECKIEFSVYGFTKINRELLTTIAGAITTYLVILFQFK
>TcasGR23
MTITISKTLFHVLSPVLHLSRLFCLLSVKWTKTSCGNYKITKSRFYTIYTLATSCLLVTTSIY
GLSQVYQLEVVYLIRLGDTTRRFVTFSDIVVVLSPCVTGPAFALFKTNQTITYLSHLKQFDS
LFNQPKKSPKIFFTTALAIFCTAFILSTDIVLWLKLSRNYIFLLCLPYYISYWSTMAIELLFWH
FVHLIRIRIGVMNEKLAEMVVTGLNSETTLKKLQDLVKGYEKLIGATNSVNDCYGFPILVII
LGCLVHLLVTPYDVYSIIMSTGDSTSILSQTVWMTAHILRLFLIIEPCHGCFVKAKETSQLVC
KLLCLSVNQEVRKSLEFFLMYLGECKIEFSAYGFTKINRGLLTTIAGAITTYLVVLFQFNKN
G
>TcasGR24
MHRTEIPVTSDLYHVLQPFIFVTRAFGFMPIAASKKGAHYVVVYLSRVYTAYSYALGSFMMF
WSLGGLHEDFHASNSVRMSNSTVKYVTLIDLGEVMVTVFVGIVVTPFKIKHLWRLLASFH
KIDAMVAPKKAQNERKIVIVSMVVAILCVLYVLTYELYLWGEVSSKNGQFWVFMKRYFIL
FSTYFMVFIEEMPYWNFIRLIRRRIEALNEVLGKGLDVFRNVTKLWITPVKLNKKNSGSFG
ILSYEKVVNLMKMYELIGDSVDAANDFCGVSILCIMFSCLIHLVVTPYFLLVEVANGGFFFF
IFLQFSWTMLHCGRLMVIVETCQYCLDEHQKTVELVFKLLSCELQEDVKDQMKLFALQL
SGRKVRFTSFSMMKFNRSLLTAIGGSITTFLVILFQFGSVGSDVYYSRD
>TcasGR25
MFRDITLDPKFFHCYQPIFTTTRFLGLNPLTCHRTPKSYLLKWSWTLYSFNCVLIAILTILGF
YGLESYIKAGTDGFVWSKFTQNHFLSILDFAELQLLGFYFVVVQPFGFKHFAEMMDGFGK
IDSLFPDFESENERRLCIIFGSGVFVVFSMVLVFDGIVVWRNVNGTELGLKDYTFYLVYFV

LIGFELQYWQLVGFIERRMRRINKNLKHSDGIFLIEENFSLFKIVENSHRLIFNAMIGYEHIY
DVINWINKIYNIQISLIISSCFLRIVLNLYVLILSIIHKDETVLLFLQVAWILLYFIRFVIILEVCH
SCELQHQKTLNLIFTLITRIVRDDTKDILKNFSLKLTQNKIRFCCFAIPRINRSLFVTICSSITT
FLIIFIQFTKL
>TcasGR28
MTSSKPLLINPNLFKTLRPLIKLSRIFGLCPITYKNSDKCLNLRIKWSWSLYILQLIAVTVITS
WAIWGFIRDMQIAEFLNLGFTSAIDLYIAIFDVNEIIITGFIFVAIIPSTFKHLPEIIDNFNKIDDT
NPQLPTNKIHRKILFLLSFVLVYMTVAYTCDLIMWSQDLLVGFIHDLPYYILYSVVVIHEVQ
YWFLVTLIRLRMSAMNGLVAETLNRNTDISSQEVCLLVETHSNILEAVSTVNRCFSGPTAII
LLSCYLHLVVCPYILYVVASSDNGLFCNTVYFLWISVHFSRLLVIIEVCNNCENECHKTRNL
IFKLLANTSKKGDVRRELKLFAYKNVGKKVRFSSYAVPKINRKLLISIATSISSYWMILYQF
GASTTGL
>TcasGR26
MYTVISPQLHYTNPNSFKMLRSITTNRSLFHTLSPLINPARILGLCPITYTKQKNFVTIRWSW
KIYMANCATALTLVCWGFWGFVHDMEIASFVSLGFTGSVDVVISSFDISDVLLSCLYFIVS
MPFKCAKLSIVFHNLNKVDAIITPVFCDRFYSNLVWFSRCWFVFLPVLYTLDVFMWGNTS
WLGVNNYFAYYVSYSIVVLHELQYYQVVKMAQLRVSGINKTVKENIKKDTSRIKLEFIFD
LIHCYNNTTDAIETINSSFNKTVTLMLFSCYVHLVTCPYQLFVMITSNETSILNYVYCLWVL
LQIFRLVLVVEVCHNCEEEIQNTRILVSQLLNCRLDKNVKKEANTFLFLMVKKKIKFSAYG
LPKVGRHLLLSVASSIGGYWMILLQFSSRTSKI
>TcasGR27
MTKFPAITENHFHIHNLCIHKMTNYKPGQKIHKSLGPIFKMSQIMGLCPVIYHKHENAFKW
SWKLYITSLVIITILACWSFWGFVRDMQVASFTALGFRGTADFVIACFDVNDVIVSAIFFVT
STPFKFKHFVQIVENFDRIDARISPILVEQIRKRSNIFVKVLVTFLPTLYVLDLFMWGKNNW
EGLNNYFAFYIMYSIVVVHELQYWHIMTMMYARILGLNKTLRDYFKNKTGFCEHEILVVT
QSFNSINDSVEEINKCFSYSTTTIIFSCYIHLVISPYQLFVVVSSTETSLFNYVYLLWISLHIMR
VLTIVEVCQKCENENRKTRSLVYQLLLCKLNEKVKNMVRVLFFLVTTRKILFSAYALPKIN
RRLIISILSSISTYWMILMQSTSRTIQVV
>DmelGR64c
LVIAQFFGVLPVAGVWPSCRPERVRFRWISLSLLAALILFVFSIVDCALSSKVVFDHGLKIY
TISLSFSVICIFCFGVFLLLSGRWPYINRRTAECEQIFLEPEYHCSYGRGSSTRLRLWGVCML
VAALCEHSTYVGSALYNNHLAIVECKLDANFWQNYFQRERQQLFLIMHFTAWWIPFIEWT
TLSMTFVWNFVDIFLILICRGMQMRFQKGNYADELYFWFCLSYVIIRVLNMMFAASSIPQE
AKEISYTLYEIPTEFWCVELRRLNEIFLSDHFALSGKGYFLLTRRLIFAVAATLMVYELVLIN
QMAGSEV
>DmelGR64d
MLRSHLSVHGLQMERSVQENTLHYTIGHVLIIARIFGVLPLAGINPNGKPENVRFRWFSPYI

LFFVVAFTFVIADFMLSTKIVLNDGLQLYTMGSLSFSVICIFCFGSFIKLSRRWPHIIRETALC
ERIFLKPCYANQEGLNFTRFLRRWALILLVAALCEHLTYVGSAAWSNYVQIRDCNLKVGFV
ENYFLRERQELFSVFEYRAWMVFFIEWNTMAMTFVWNFGDIFLFLMCRGLKIRFQQLHW
RIRQNLGKPMAKEFWQEIRSDFLDLDSLLKLYDKELSGLILVCCAHNMYFICVQVYHSFQ
VKGAFMDELYFWFCLLYVISRLMNMMLAASSIPQEIKDISNTLYEVRSSPWCDELGRLSE
MLRNETFALSGMGYFYVTRRLIFAMAGALMGYELVLFRQMQGAVVQKSICSRGPGSSMSI
FFS
>DmelGR64b
MPQGETFHRAVSNVLFISQIYGLLPVSNVRALDVADIRFRWCSPRILYSLLIGILNLSEFGAVI
NYVIKVTINFHTSSTLSLYIVCLLEHLFFWRLAIQWPRIMRTWHGVEQLFLRVPYRFYGEY
RIKRRIYIVFTIVMSSALVEHCLLLGNSFHLSNMERTQCKINVTYFESIYKWERPHLYMILP
YHFWMLPILEWVNQTIAYPRSFTDCFIMCIGIGLAARFHQLYRRIAAVHRKVMPAVFWTEV
REHYLALKRLVHLLDAAIAPLVLLAFGNNMSFICFQLFNSFKNIGVDFLVMLAFWYSLGFA
VVRTLLTIFVASSINDYERKIVTALRDVPSRAWSIEVQRFSEQLGNDTTALSGSGFFYLTRSL
VLAMGTTIITYELMISDVINQGSIRQKTQYCREY
>DmelGR64a
MKGPNLNFRKTPSKDNGVKQVESLARPETPPPKFVEDSNLEFNVLASEKLPNYTNLDLFH
RAVFPFMFLAQCVAIMPLVGIRESNPRRVRFAYKSIPMFVTLIFMIATSILFLSMFTHLLKIGI
TAKNFVGLVFFGCVLSAYVVFIRLAKKWPAVVRIWTRTEIPFTKPPYEIPKRNLSRRVQLAA
LAIIGLSLGEHALYQVSAILSYTRRIQMCANITTVPSFNNYMQTNYDYVFQLLPYSPIIAVLI
LLINGACTFVWNYMDLFIMMISKGLSYRFEQITTRIRKLEHEEVCESVFIQIREHYVKMCE
LLEFVDSAMSSLILLSCVNNLYFVCYQLLNVFNKLRWPINYIYFWYSLLYLIGRTAFVFLTA
ADINEESKRGLGVLRRVSSRSWCVEVERLIFQMTTQTVALSGKKFYFLTRRLLFGMAGTIV
TYELVLLQFDEPNRRKGLQPLCA
>DmelGR64f
MKILPKLERKLRRLKKRVTRTSLFRKLDLVHESARKKAFQESCETYKNQIENEYEIRNSLP
KLSRSDKEAFLSDGSFHQAVGRVLLVAEFFAMMPVKGVTGKHPSDLSFSWRNIRTCFSLLF
IASSLANFGLSLFKVLNNPISFNSIKPIIFRGSVLLVLIVALNLARQWPQLMMYWHTVEKDL
PQYKTQLTKWKMGHTISMVMLLGMMLSFAEHILSMVSAINYASFCNRTADPIQNYFLRTN
DEIFFVTSYSTTLALWGKFQNVFSTFIWNYMDLFVMIVSIGLASKFRQLNDDLRNFKGMN
MAPSYWSERRIQYRNICILCDKMDDAISLITMVSFSNNLYFICVQLLRSLNTMPSVAHAVY
FYFSLIFLIGRTLAVSLYSSSVHDESRLTLRYLRCVPKESWCPEVKRFTEEVISDEVALTGMK
FFHLTRKLVLSVAGTIVTYELVLIQFHEDNDLWDCDQSYYS
>DmelGR64e
MARTTGDPAKRRRCMSRIKFWRRSRVGSDATLGIIKYRVVEKDTKRFKLSLIKAWLLRIRQ
EDYKYSGSFQEAIKPVLIIAQIFALMPVRKVSSKFAEDLTFTWFSVRSYYALVTILFFGVSSG
YMVAFVTSVSFNFDSVETLVFYLSIFLISLSFFQLARKWPEIAQSWQLVEAKLPPLKLPKER

RSLAQHINMITIVATTCSLVEHIMSMLSMGYYVNSCPRWPDRPIDSFLYLSFSSVFYFVDYT
RFLGIVGKVVNVLSTFAWNFNDIFVMAVSVALAARFRQLNDYMMREARLPTTVDYWMQ
CRINFRNLCKLCEEVDDAISTITLLCFSNNLYFICGKILKSMQAKPSIWHALYFWFSLVYLL
GRTLILSLYSSSINDESKRPLVIFRLVPREYWCDELKRFSEEVQMDNVALTGMKFFRLTRGV
VISVAGTIVTYELILLQFNGEEKVPGCFEN
>DmelGR5a
MRQLKGRNRCNRAVRHLKVQGKMWLKNLKSGLEQIRESQVRGTRKNFLHDGSFHEAVA
PVLAVAQCFCLMPVCGISAPTYRGLSFNRRSWRFWYSSLYLCSTSVDLAFSIRRVAHSVLD
VRSVEPIVFHVSILIASWQFLNLAQLWPGLMRHWAAVERRLPGYTCCLQRARPARRLKLV
AFVLLVVSLMEHLLSIISVVYYDFCPRRSDPVESYLLGASAQLFEVFPYSNWLAWLGKIQN
VLLTFGWSYMDIFLMMLGMGLSEMLARLNRSLEQQVRQPMPEAYWTWSRTLYRSIVELI
REVDDAVSGIMLISFGSNLYFICLQLLKSINTMPSSAHAVYFYFSLLFLLSRSTAVLLFVSAIN
DQAREPLRLLRLVPLKGYHPEVFRFAAELASDQVALTGLKFFNVTRKLFLAMAGTVATYE
LVLIQFHEDKKTWDCSPFNLD
>DmelGR21a
MTFLDRTMSFWAVSRGLTPPSKVVPMLNPNQRQFLEDEVRYREKLKLMARGDAMEEVY
VRKQETVDDPLELDKHDSFYQTTKSLLVLFQIMGVMPIHRNPPEKNLPRTGYSWGSKQV
MWAIFIYSCQTTIVVLVLRERVKKFVTSPDKRFDEAIYNVIFISLLFTNFLLPVASWRHGPQ
VAIFKNMWTNYQYKFFKTTGSPIVFPNLYPLTWSLCVFSWLLSIAINLSQYFLQPDFRLWY
TFAYYPIIAMLNCFCSLWYINCNAFGTASRALSDALQTTIRGEKPAQKLTEYRHLWVDLSH
MMQQLGRAYSNMYGMYCLVIFFTTIIATYGSISEIIDHGATYKEVGLFVIVFYCMGLLYIIC
NEAHYASRKVGLDFQTKLLNINLTAVDAATQKEVEMLLVAINKNPPIMNLDGYANINRELI
TTNISFMATYLVVLLQFKITEQRRIGQQQA
>DmelGR63a
MANYYRRKKGDAVFLNAKPLNSANAQAYLYGVRKYSIGLAERLDADYEAPPLDRKKSS
DSTASNNPEFKPSVFYRNIDPINWFLRIIGVLPIVRHGPARAKFEMNSASFIYSVVFFVLLAC
YVGYVANNRIHIVRSLSGPFEEAVIAYLFLVNILPIMIIPILWYEARKIAKLFNDWDDFEVLY
YQISGHSLPLKLRQKAVYIAIVLPILSVLSVVITHVTMSDLNINQVVPYCILDNLTAMLGAW
WFLICEAMSITAHLLAERFQKALKHIGPAAMVADYRVLWLRLSKLTRDTGNALCYTFVFM
SLYLFFIITLSIYGLMSQLSEGFGIKDIGLTITALWNIGLLFYICDEAHYASVNVRTNFQKKLL
MVELNWMNSDAQTEINMFLRATEMNPSTINCGGFFDVNRTLFKGLLTTMVTYLVVLLQF
QISIPTDKGDSEGANNITVVDFVMDSLDNDMSLMGASTLSTTTVGTTLPPPIMKLKGRKG
>DmelGR28bB
MSALRRVRKYFISSQVYEALRPLFFLTFLYGLTPFHVVRRKMGESYLKMSCFGVFNIFIYIC
LCGFCYISSLRQGESIVGYFFRTEISTIGDRLQIFNGLIAGAVIYTSAILKRCKLLGTLTILHSL
DTNFSNIGVRVKYSRIFRYSLLVLIFKLLILGVYFVGVFRLLVSLDVTPSFCVCMTFFLQHSV
VSIAICLFCVIAFSFERRLSIINQVLKNLAHQWDTRSLKAVNQKQRSLQCLDSFSMYTIVTK

DPAEIIQESMEIHHLICEAAATANKYFTYQLLTIISIAFLIIVFDAYYVLETLLGKSKRESKFKT
VEFVTFFSCQMILYLIAIISIVEGSNRAIKKSEKTGGIVHSLLNKTKSAEVKEKLQQFSMQL
MHLKINFTAAGLFNIDRTLYFTISGALTTYLIILLQFTSNSPNNGYGNGSSCCETFNNMTNH
TL

>DmelGR28bC
MDIEMAKEPVNPTDTPDIEVTPGLCQPLRRRFRRFVTAKQLYECLRPVFHVTYIHGLTSFYI
SCDTKTGKKAIKKTIFGYINGIMHIAMFVFAYSLTIYNNCESVASYFFRSRITYFGDLMQIVS
GFIGVTVIYLTAFVPNHRLERCLQKFHTMDVQLQTVGVKIMYSKVLRFSYMVLISMFLVN
VLFTGGTFSVLYSSEVAPTMALHFTFLIQHTVIAIAIALFSCFTYLVEMRLVMVNKVLKNLA
HQWDTRSLKAVNQKQRSLQCLDSFSMYTIVTKDPAEIIQESMEIHHLICEAAATANKYFTY
QLLTIISIAFLIIVFDAYYVLETLLGKSKRESKFKTVEFVTFFSCQMILYLIAIISIVEGSNRAIK
KSEKTGGIVHSLLNKTKSAEVKEKLQQFSMQLMHLKINFTAAGLFNIDRTLYFTISGALTT
YLIILLQFTSNSPNNGYGNGSSCCETFNNMTNHTL

>DmelGR28bD
MSFYFCEIFKPRDAFGAEQTLLLYTYLLGLTPFRLRGQAGERQFHLSKIGYLNAFLQLSFFS
YCFLAALIEQQSIVGYFFKSEISQMGDSLQKFIGMTGMSILFLCSSIRVRLLIHIWDRISYIDD
RFLNLGVCFNYPAIMRLRLLQIFLINGVQLGYLISSNWMLLGNDVRPIYTAIVAFYVPQIFL
LSIVMLFNATLHRLWQHFTVLNQVLKNLAHQWDTRSLKAVNQKQRSLQCLDSFSMYTIV
TKDPAEIIQESMEIHHLICEAAATANKYFTYQLLTIISIAFLIIVFDAYYVLETLLGKSKRESKF
KTVEFVTFFSCQMILYLIAIISIVEGSNRAIKKSEKTGGIVHSLLNKTKSAEVKEKLQQFSMQ
LMHLKINFTAAGLFNIDRTLYFTISGALTTYLIILLQFTSNSPNNGYGNGSSCCETFNNMTN
HTL

>DmelGR28bE
MWLLRRSVGKSGNRPHDVYTCYRLTIFMALCLGIVPYYVSISSEGRGKLTSSYIGYINIIIR
MAIYMVNSFYGAVNRDTLMSNFFLTDISNVIDALQKINGMLGIFAILLISLLNRKELLKLLA
TFDRLETEAFPRVGVAMHQVAANKKMNRLVIILVGSMVAYITCSFLMISLRDTTTFSISAVIS
FFSPHFIVCAVSFLAGNVMIKLRIYLSALNEVLKNLAHQWDTRSLKAVNQKQRSLQCLDSF
SMYTIVTKDPAEIIQESMEIHHLICEAAATANKYFTYQLLTIISIAFLIIVFDAYYVLETLLGKS
KRESKFKTVEFVTFFSCQMILYLIAIISIVEGSNRAIKKSEKTGGIVHSLLNKTKSAEVKEKL
QQFSMQLMHLKINFTAAGLFNIDRTLYFTISGALTTYLIILLQFTSNSPNNGYGNGSSCCETF
NNMTNHTL

>DmelGR28a
MAFKLWERFSQADNVFQALRPLTFISLLGLAPFRLNLNPRKEVQTSKFSFFAGIVHFLFFVL
CFGISVKEGDSIIGYFFQTNITRFSDGTLRLTGILAMSTIFGFAMFKRQRLVSIIQNNIVVDEIF
VRLGMKLDYRRILLSSFLISLGMLLFNVIYLCVSYSLLVSATISPSFVTFTTFALPHINISLMV
FKFLCTTDLARSRFSMLNEILQDILDAHIEQLSALELSPMHSVVNHRRYSHRLRNLISTPMK
RYSVTSVIRLNPEYAIKQVSNIHNLLCDICQTIEEYFTYPLLGIIAISFLFILFDDFYILEAILNP

KRLDVFEADEFFAFFLMQLIWYIVIIVLIVEGSSRTILHSSYTAAIVHKILNITDDPELRDRLF
RLSLQLSHRKVLFTAAGLFRLDRTLIFTITGAATCYLIILIQFRFTHHMDDTSSNSTNNLHSI
HLGD
>DmelGR43a
MEISQPSIGIFYISKVLALAPYATVRNSKGRVEIGRSWLFTVYSATLTVVMVFLTYRGLLFD
ANSEIPVRMKSATSKVVTALDVSVVVMAIVSGVYCGLFSLNDTLELNDRLNKIDNTLNAY
NNFRRDRWRALGMAAVSLLAISILVGLDVGTWMRIAQDMNIAQSDTELNVHWYIPFYSL
YFILTGLQVNIANTAYGLGRRFGRLNRMLSSSFLAENNATSAIKPQKVSTVKNVSVNRPAM
PSALHASLTKLNGETLPSEAAGDKAAARSLILNVELLKLGYFPAKNKGLLLKSLADSHESL
GKCVHLLSNSFGIAVLFILVSCLLHLVATAYFLFLELLSKRDNGYLWVQMLWICFHFLRLL
MVVEPCHLAARESRKTIQIVCEIERKVHEPILAEAVKKFWQQLLVVDADFSACGLCRVNR
TILTSFASAIATYLVILIQFQRTNG
>DmelGR33a
MIQIMNWFSMVIGLIPLNRQQSETNFILDYAMMCIVPIFYVACYLLINLSHIIGLCLLDSCNS
VCKLSSHLFMHLGAFLYLTITLLSLYRRKEFFQQFDARLNDIDAVIQKCQRVAEMDKVKVT
AVKHSVAYHFTWLFLFCVFTFALYYDVRSLYLTFGNLAFIPFMVSSFPYLAGSIIQGEFIYHV
SVISQRFEQINMLLEKINQEARHRHAPLTVFDIESEGKKERKTVTPITVMDGRTTTGFGNEN
KFAGEMKRQEGQQKNDDDDLDTSNDEDEDDFDYDNATIAENTGNTSEANLPDLFKLHD
KILALSVITNGEFGPQCVPYMAACFVVSIFGIFLETKVNFIVGGKSRLLDYMTYLYVIWSFT
TMMVAYIVLRLCCNANNHSKQSAMIVHEIMQKKPAFMLSNDLFYNKMKSFTLQFLHWE
GFFQFNGVGLFALDYTFIFSTVSAATSYLIVLLQFDMTAILRNEGLMS
>DmelGR66a
MAQAEDAVQPLLQQFQQLFFISKIAGILPQDLEKFRSRNLLEKSRNGMIYMLSTLILYVVLY
NILIYSFGEEDRSLKASQSTLTFVIGLFLTYIGLIMMVSDQLTALRNQGRIGELYERIRLVDER
LYKEGCVMDNSTIGRRIRIMLIMTVIFELSILVSTYVKLVDYSQWMSLLWIVSAIPTFINTLD
KIWFAVSLYALKERFEAINATLEELVDTHEKHKLWLRGNQEVPPPLDSSQPPQYDSNLEYL
YKELGGMDIGSIGKSSVSGSGKNKVAPVAHSMNSFGEAIDAASRKPPPPPLATNMVHESEL
GNAAKVEEKLNNLCQVHDEICEIGKALNELWSYPILSLMAYGFLIFTAQLYFLYCATQYQSI
PSLFRSAKNPFITVIVLSYTSGKCVYLIYLSWKTSQASKRTGISLHKCGVVADDNLLYEIVN
HLSLKLLNHSVDFSACGFFTLDMETLYGVSGGITSYLIILIQFNLAAQQAKEAIQTFNSLND
TAGLVGAATDMDNISSTLRDFVTTTMTPAV
>DmelGR47b
MQRDDGFVYCYGNLYSLLLYWGLVTIRVRSPDRGGAFSNRWTVCYALFTRSFMVICFMA
TVMTKLRDPEMSAAMFGHLSPLVKAIFTWECLSCSVTYIEYCLSLDLQKDRHLKLVARMQ
EFDRSVLMVFPHVQWNYRRARLKYWYGTVIVGFCFFSFSISLIFDTTRCTCGIPSTLLMAF
TYTLLTSSVGLLGFVHIGIMDFIRVRLRLVQQLLHQLYQADDSSEVHERIAYLFEMSKRCSF
LLAELNGVFGFAAAAGIFYDFTIMTCFVYVICQKLLEREPWDPEYVYMLLHVAIHTYKVV

ITSTYGYLLLREKRNCMHLLSQYSRYFSGQDVARRKTEDFQHWRMHNRQAAMVGSTTL
LSVSTIYLVYNGMANYVIILVQLLFQQQQIKDHQLTSGKDVDIVGPMGPITHMD
>DmelGR57a
MAVLYFFREPETVFDCAAFICILQFLMGCNGFGIRRSTFRISWASRIYSMSVAIAAFCCLFGS
LSVLLAEEDIRERLAKADNLVLSISALELLMSTLVFGVTVISLQVFARRHLGIYQRLAALDA
RLMSDFGANLNYRKMLRKNIAVLGIVTTIYLMAINSAAVQVASGHRALFLLFALCYTIVTG
GPHFTGYVHMTLAEMLGIRFRLLQQLLQPEFLNWRFPQLHVQELRIRQVVSMIQELHYLI
QEINRVYALSLWAAMAHDLAMSTSELYILFGQSVGIGQQNEEENGSCYRMLGYLALVMIP
PLYKLLIAPFYCDRTIYEARRCLRLVEKLDDWFPQKSSLRPLVESLMSWRIQAKIQFTSGLD
VVLSRKVIGLFTSILVNYLLILIQFAMTQKMGEQIEQQKIALQEWIGF
>DmelGR23A
MKTLECLTRRFLEVIFSVLALVPLPPISQLGWLFLSLAIRCCWIVYFIYLLDVAISFSWVAIEN
VGNAVGTMLFVGNSVLGFALLLESVLKQKTHSQLEDLRVQTELQLQRLGMFGRSRHAAY
LLPLIGVQFTCDLVRLATNFGETVSPVFCISLPLMWLLRYRYVQLVQHVMDLNQRSIHLRR
SLLSMASGNDLWQPYGVQECLQLQTLRTTYERIFECYETFSDCYGWGMLGLHLLTSFQFV
TNAYWMIMGIYDGGNVRSLIFNGATGIDFGTPIATLFWHGDSGAENNQAGPVGRTDCVRG
LRSLVGLHCAGDAGSHFGSRVFGVLERARICLSYQDLSPENAAQFSGSAGADYILYPGGH
EGEHKGFVVTAKDFFSLNLHLLSSMFAAVVTYLVILIQFMFAERSSTRGSG
>DmelGR23aB
MFPPTRVQASSRVVLKIFHFILVAFSLRSRRLSRLVLWLQFLGWLTWFISMWTQSVIYAQTI
DCTLDCSLRHILTFFQTVSHAFIVVTSFLDGFRIKQDQLDEPIAFEDSDPWLAFTVLAMLVP
TLGVEYLVCSNAPEYAFRIRIYHLKTLPSFLALQVQIISFILEVMKVNIRVRQTKLQLLILAR
ELSCRWPQRKQKPQFSDQQAHRVKDLKRRYNDLHYLFVRINGYFGGSLLTIIIVHFAIFVS
NSYWLFVDIRTRPWRIYAILLNLGFIFNVALQMAAACWHCQQSYNLGRQIGCLISKLVKPQ
GSKLYNDLVSEFSLQTLHQRFVVTAKDFFSLNLHLLSSMFAAVVTYLVILIQFMFAERSSTR
GSG
>DmelGR23aC
MKTLECLTRRFLEVIFSVLALVPLPPISQLGWLFLSLAIRCCWIVYFIYLLDVAISFSWVAIEN
VGNAVGTMLFVGNSVLGFALLLESVLKQKTHSQLEDLRVQTELQLQRLGMFGRSRHAAY
LLPLIGVQFTCDLVRLATNFGETVSPVFCISLPLMWLLRYRYVQLVQHVMDLNQRSIHLRR
SLLSMASGNDLWQPYGVQECLQLQTLRTTYERIFECYETFSDCYGWGMLGLHLLTSFQFV
TNAYWMIMGIYDGGNVRSLIFNGATGIDFGTPIATLFWHGDSGAENGRQIGCLISKLVKPQ
GSKLYNDLVSEFSLQTLHQRFVVTAKDFFSLNLHLLSSMFAAVVTYLVILIQFMFAERSSTR
GSG
>DmelGR2a
MDTLRALEPLHRACQVCNLWPWRLAPPPDSEGILLRRSRWLELYGWTVLIAATSFTVYGL
FQESSVEEKQDSESTISSIGHTVDFIQLVGMRVAHLAALLEALWQRQAQRGFFAELGEIDRL

LSKALRVDVEAMRINMRRQTSRRAVWILWGYAVSQLLILGAKLLSRGDRFPIYWISYLLPL
LVCGLRYFQIFNATQLVRQRLDVLLVALQQLQLHQKGPAVDTVLEEQEDLEEAAMDRLIA
VRLVYQRVWALVALLNRCYGLSMLMQVGNDFLAITSNCYWMFLNFRQSAASPFDILQIVA
SGVWSAPHLGNVLVLSLLCDRTAQCASRLALCLHQVSVDLRNESHNALVGTLVRYCAPLI
ILVPLQITQFSLQLLHQRLHFSAAGFFNVDCTLLYTIVGATTTYLIILIQFHMSESTIGSDSNG
Q
>DmelGR8a
MSGHLGRVLQFHLRLYQVLGFHGLPLPGDGNPARTRRRLMAWSLFLLISLSALVLACLFS
GEEFLYRGDMFGCANDALKYVFAELGVLAIYLETLSSQRHLANFWWLHFKLGGQKTGLV
SLRSEFQQFCRYLIFLYAMMAAEVAIHLGLWQFQALTQHMLLFWSTYEPLVWLTYLRNLQ
FVLHLELLREQLTGLEREMGLLAEYSRFASETGRSFPGFESFLRRRLVQKQRIYSHVYDML
KCFQGAFNFSILAVLLTINIRIAVDCYFMYYSIYNNVINNDYYLIVPALLEIPAFIYASQSCMV
VVPRIAHQLHNIVTDSGCCSCPDLSLQIQNFSLQLLHQPIRIDCLGLTILDCSLLTRMACSVG
TYMIYSIQFIPKFSNTYM
>DmelGR39b
MLYSFHPYLKYFALLGLVPWSESCAQSKFVQKVYSAILIILNAVHFGISIYFPQSAELFLSLM
VNVIVFVARIVCVTVIILQVMVHYDDYFRFCREMKYLGLRLQCELKIHVGRLKWQSYAKI
LALGIGFLVTVLPSIYVALSGSLLYFWSSLLSILIIRMQFVLVLLNVELLGHHVSLLGIRLQN
VLECHLMGANCTLDGNANRLCSLEFLLALKQSHMQLHYLFTHFNDLFGWSILGTYVVLF
SDSTVNIYWTQQVLVEVYEYKYLYATFSVFVPSFFNILVFCRCGEFCQRQSVLIGSYLRNLS
CHPSIGRETSYKDLLMEFILQVEQNVLAINAEGFMSTDNSLLMSILAAKVTYLIVLMQFSS
V
>DmelGR98b
MVAQKSRLLARAFPYLDIFSVFALTPPPQSFGHTPHRRLRWYLMTGYVFYATAILATVFIVS
YFNIIAIDEEVLEYNVSDFTRVMGNIQKSLYSIMAIANHLNMLINYRRLGGIYKDIADLEMD
MDEASQCFGGQRQRFSFRFRMALCVGVWMILMVGSMPRLTMTAMGPFVSTLLKILTEFV
MIMQQLKSLEYCVFVLIIYELVLRLRRTLSQLQEEFQDCEQQDMLQALCVALKRNQLLLG
RIWRLEGDVGSYFTPTMLLLFLYNGLTILHMVNWAYINKFLYDSCCQYERFLVCSTLLVNL
LLPCLLSQRCINAYNCFPRILHKIRCTSADPNFAMLTRGLREYSLQMEHLKLRFTCGGLFDI
NLKYFGGLLVTIFGYIIILIQFKVQAIAANRYKKVVN
>DmelGR98c
MEMEAKRSRLLTTARPYLQVLSLFGLTPPAEFFTRTLRKRRRFCWMAGYSLYLIAILLMVF
YEFHANIVSLHLEIYKFHVEDFSKVMGRTQKFLIVAIATCNQLNILLNYGRLGLIYDEIANL
DLGIDKSSKNFCGKSHWWSFRLRLTLSIGLWMVIIIGVIPRLTLGRAGPFFHWVNQVLTQII
LIMLQLKGPEYCLFVLLVYELILRTRHVLEQLKDDLEDFDCGARIQELCVTLKQNQLLIGRI
WRLVDEIGAYFRWSMTLLFLYNGLTILHVVNWAIIRSIDPNDCCQLNRLGSITFLSFNLLLT
CFFSECCVKTYNSISYILHQIGCLPTAEEFQMLKMGLKEYILQMQHLKLLFTCGGLFDINIK

LFGGMLVTLCGYVIIIVQFKIQDFALIGYRQNTSDTS
>DmelGR98d
MEANRSRLLAAARPYIQIYSIFGLTPPIQFFTRTLHKRRRGIVILGYACYLISISLMVIYECYA
NIVALQKDIHKFHAEDSSKVMGNTQKVLVVAMFVWNQLNILLNFRRLARIYDDIADLEID
LNNASSGFVGQRHWWRFRFRLALSVGLWIVLLVGLTPRFTLVALGPYLHWTNKVLTEIILI
MLQLKCTEYCVFVLLIYELILRGRHILQQISVELEGNQSRDSVQELCVALKRNQLLAGRIW
GLVNEVSLYFTLSLTLLFLYNELTILQIVNWALIKSVNPNECCQYRRVGTCLLLSINIFLSCLY
SEFCIQTYNSISRVLHQMYCLSAAEDYLILKMGLREYSLQMEHLKLIFTCGGLFDINLKFFG
GMVVTLFGYIIILVQFKIQFFAQSNFMQNINSTELKAYTA
>DmelGR98a
MEQMSGELHAASLLYMRRLMKCLGMLPFGQNLFSKGFCYVLLFVSLGFSSYWRFSFDYE
FDYDFLNDRFSSTIDLSNFVALVLGHAIIVLELLWGNCSKDVDRQLQAIHSQIKLQLGTSNS
TDRVRRYCNWIYGSLIIRWLIFIVVTIYSNRALTINATYSELVFLARFSEFTLYCAVILFIYQEL
IVGGSNVLDELYRTRYEMWSIRRLSLQKLAKLQAIHNSLWQAIRCLECYFQLSLITLLMKF
FIDTSALPYWLYLSRVEHTRVAVQHYVATVECIKLLEIVVPCYLCTRCDAMQRKFLSMFYT
VTTDRRSSQLNAALRSLNLQLSQEKYKFSAGGMVDINTEMLGKFFFGMISYIVICIQFSINF
RAKKMSNEQMSQNITSTSAPI
>DmelGR39aA
MSKVCRDLRIYLRLLHIMGMMCWHFDSDHCQLVATSGSERYAVVYAGCILVSTTAGFIFAL
LHPSRFHIAIYNQTGNFYEAVIFRSTCVVLFLVYVILYAWRHRYRDLVQHILRLNRRCASSC
TNQQFLHNIILYGMLTILCFGNYLHGYTRAGLATLPLALCMLVYIFAFLVLCLLLMFFVSLK
QVMTAGLIHYNQQLCQGDLISGLRGRQQILKLCGGELNECFGLLMLPIVALVLLMAPSGPF
FLISTVLEGKFRPDECLIMLLTSSTWDTPWMIMLVLMLRTNGISEEANKTAKMLTKVPRTG
TGLDRMIEKFLLKNLRQKPILTAYGFFALDKSTLFKLFTAIFTYMVILVQFKEMENSTKSIN
KF
>DmelGR39aB
MGTRNRKLLFFLHYQRYLGLTNLDFSKSLHIYWLHGTWSSTAIQIVVVGVFMAALLGALA
ESLYYMETKSQTGNTFDNAVILTTSVTQLLANLWLRSQQKSQVNLLQRLSQVVELLQFEP
YAVPQFRWLYRIWLLVCLIYGAMVTHFGINWLTTMQISRVLTLIGFVYRCVLANFQFTCYT
GMVVILKKLLQVQVKQLEHLVSTTTISMAGVAGCLRTHDEILLLGQRELIAVYGGVILFLFI
YQVMQCILIFYISNLEGFHSSNDLVLIFCWLAPMLFYLILPLVVNDIHNQANKTAKMLTKV
PRTGTGLDRMIEKFLLKNLRQKPILTAYGFFALDKSTLFKLFTAIFTYMVILVQFKEMENST
KSINKF
>DmelGR39aC
MDFQPGELCAYYRLCRYLGIFCIDYNPTKKKFRLRRSVLCYIVHFALQAYLVGCISVMVTY
WRRCFKSELTTTGNHFDRLVMVIALGILVVQNAWLIWLQAPHLRIVRQIEFYRRNHLANV
RLLLPKRLLWLIIATNVVYMANFIKTCIFEWLTDASRLFVITSLGFPLRYLVTSFTMGTYFC

MVHIVRLVLDWNQSQINAIIDESADLKMTSPNRLRLRVCLEMHDRLMLLCNDEISLVYGFI
AWLSWMFASLDVTGVIYLTMVIQTKKSIVLKLITNVVWLSPTFMTCAASFMSNRVTIQAN
KTAKMLTKVPRTGTGLDRMIEKFLLKNLRQKPILTAYGFFALDKSTLFKLFTAIFTYMVILV
QFKEMENSTKSINKF
>DmelGR39aD
MKRNAFEELRVQLRTLKWLGVLRFTIDFNKCLVRENASEERSAWLYLIGVVGITCSLIVYS
TYFPSHFIMGKHNTTGNCYALINIRSCSIVTMLIYTQLYIQRFRFVALLQSILRFNQISGSHRE
EGRFAFYYYTHLSLLIICMLNYAYGYWTAGVRLTTIPIYLLQYGFSYLFLGQVVVLFACIQQ
ILLSILKYYNQVVLKNIKSSKESREFYYNFCKYNQVIWLSYTEINHCFGLLLLLVTGLILLIT
PSGPFYLVSTIFEGRFRQNWQFSLMSFTAILWSLPWIVLLVLAMGRNDVQKEANKTAKML
TKVPRTGTGLDRMIEKFLLKNLRQKPILTAYGFFALDKSTLFKLFTAIFTYMVILVQFKEME
NSTKSINKF
>DmelGR32a
MSPNTWVIEMPTQKTRSHPYPRRISPYRPPVLNRDAFSRDAPPMPARNHDHPVFEDIRTILS
VLKASGLMPIYEQVSDYEVGPPTKTNEFYSFFVRGVVHALTIFNVYSLFTPISAQLFFSYRE
TDNVNQWIELLLCILTYTLTVFVCAHNTTSMLRIMNEILQLDEEVRRQFGANLSQNFGFLV
KFLVGITACQAYIIVLKIYAVQGEITPTSYILLAFYGIQNGLTATYIVFASALLRIVYIRFHFIN
QLLNGYTYGQQHRRKEGGARARRQRGDVNPNVNPALMEHFPEDSLFIYRMHNKLLRIYK
GINDCCNLILVSFLGYSFYTVTTNCYNLFVQITGKGMVSPNILQWCFAWLCLHVSLLALLS
RSCGLTTTEANATSQILARVYAKSKEYQNIIDKFLTKSIKQEVQFTAYGFFAIDNSTLFKIFSA
VTTYLVILIQFKQLEDSKVEDPVPEQT
>DmelGR68a
MKIYQDIYPISKPSQIFAILPFYSGDVDDGFRFGGLGRWYGRLVALIILIGSLTLGEDVLFASK
EYRLVASAQGDTEEINRTIETLLCIISYTMVVLSSVQNASRHFRTLHDIAKIDEYLLANGFRE
TYSCRNLTILVTSAAGGVLAVAFYYIHYRSGIGAKRQIILLLIYFLQLLYSTLLALYLRTLMM
NLAQRIGFLNQKLDTFNLQDCGHMENWRELSNLIEVLCKFRYITENINCVAGVSLLFYFGF
SFYTVTNQSYLAFATLTAGSLSSKTEVADTIGLSCIWVLAETITMIVICSACDGLASEVNGTA
QILARIYGKSKQFQNLIDKFLTKSIKQDLQFTAYGFFSIDNSTLFKIFSAVTTYLVILIQFKQL
EDSKVEDISQA
>DmelGR36a
MFDWVGLLLKVLYYYGQIIGLINFEIDWQRGRVVAAQRGILFAIAINVLICMVLLLQISKKF
NLDVYFGRANQLHQYVIIVMVSLRMASGISAILNRWRQRAQLMRLVECVLRLFLKKPHV
KQMSRWAILVKFSVGVVSNFLQMAISMESLDRLGFNEFVGMASDFWMSAIINMAISQHYL
VILFVRAYYHLLKTEVRQAIHESQMLSEIYPRRAAFMTKCCYLADRIDNIAKLQNQLQSIV
TQLNQVFGIQGIMVYGGYYIFSVATTYITYSLAINGIEELHLSVRAAALVFSWFLFYYTSAI
LNLFVMLKLFDDHKEMERILEERTLFTSALDVRLEQSFESIQLQLIRNPLKIEVLDIFTITRSS
SAAMIGSIITNSIFLIQYDMEYF

>DmelGR36b
MVDWVVLLLKAVHIYCYLIGLSNFEFDCRTGRVFKSRRCTIYAFMANIFILITIIYNFTAHGD
TNLLFQSANKLHEYVIIIMSGLKIVAGLITVLNRWLQRGQMMQLVKDVIRLYMINPQLKS
MIRWGILLKAFISFAIELLQVTLSVDALDRQGTAEMMGLLVKLCVSFIMNLAISQHFLVILLI
RAQYRIMNAKLRMVIEESRRLSFLQLRNGAFMTRCCYLSDQLEDIGEVQSQLQSMVGQL
DEVFGMQGLMAYSEYYLSIVGTSYMSYSIYKYGPHNLKLSAKTSIIVCILITLFYLDALVNC
NNMLRVLDHHKDFLGLLEERTVFASSLDIRLEESFESLQLQLARNPLKINVMGMFPITRGS
TAAMCASVIVNSIFLIQFDMEFF
>DmelGR36c
MDLESFLLGAVYYYGLFIGLSNFEFDWNTGRVFTKKWSTLYAIALDSCIFALYIYHWTGNT
NIVNAIFGRANMLHEYVVAILTGLRIVTGLFTLILRWYQRCKMMDLASKVVRMYVARPQ
VRRMSRWGILTKFIFGSITDGLQMAMVLSAMGSVDSQFYLGLGLQYWMFVILNMAMMQ
QHMIMLFVRTQFQLINTELRQVIDEAKDLLLSPRHQGVFMTKCCSLADQIENIARIQSQLQ
TIMNQMEEVFGIQGAMTYGGYYLSSVGTCYLAYSILKHGYENLSMTLSTVILAYSWCFFY
YLDGMLNLSVMLHVQDDYWEMLQILGKRTIFVGLDVRLEEAFENLNLQLIRNPLKITVV
KLYDVTRSNTMAMFGNLITHSIFLIQYDIEHF
>DmelGR59c
MVDLVKTILLIAYWYGLAVGVSNFEVDWLTGEAIATRRTTIYAAVHNASLITLLILFNLGNN
SLKSEFISARYLHEYFFMLMTAVRISAVLLSLITRWYQRSRFIRIWNQILALVRDRPQVVRG
RWYRRSIILKFVFCVLSDSLHTISDVSAQRKRITADLIVKLSLLATLTTIFNMIVCQYYLAMV
QVIGLYKILLQDLRCLVRQAECICSIRNRRGGVYSIQCCSLADQLDLIAERHYFLKDRLDE
MSDLFQIQSLSMSLVYFFSTMGSIYFSVCSILYSSTGFGSTYWGLLLIVLSTASFYMDNWLS
VNIGFHIRDQQDELFRVLADRTLFYRELDNRLEAAFENFQLQLASNRHEFYVMGLFKMER
GRLIAMLSSVITHTMVLVQWEIQNDES
>DmelGR59d
MADLLKLCLRIAYAYGRLTGVINFKIDLKTGQALVTRGATLISVSTHLLIFALLLYQTMRKS
VVNVMWKYANSLHEYVFLVIAGFRVVCVFLELVSRWSQRRTFVRLFNSFRRLYQRNPDII
QYCRRSIVSKFFCVTMTETLHIIVTLAMMRNRLSIALALRIWAVLSLTAIINVIITQYYVATA
CVRGRYALLNKDLQAIVTESQSLVPNGGGVFVTKCCYLADRLERIAKSQSDLQELVENLS
TAYEGEVVCLVITYYLNMLGTSYLLFSISKYGNFGNNLLVIITLCGIVYFVFYVVDCWINAF
NVFYLLDAHDKMVKLLNKRTLFQPGLDHRLEMVFENFALNLVRNPLKLHMYGLFEFGR
GTSFAVFNSLLTHSLLLIQYDVQNF
>DmelGR58c
MNQYFLLHTYFQVSRLIGLCNLHYDSSNHRFILNHVPTVVYCVILNVVYLLVLPFALFVLT
GNIYHCPDAGMFGVVYNVVALTKLLTMLFLMSSVWIQRRRLYKLGNDLMKMLHKFRFN
LGNDCRNRCLCKGLLTSSRFVLLTQQLLTRDSVVNCESNSSLRQAMVPYQSAAIVYALIMI
LLMSYVDMTVYMVEVAGNWLLVNMTQGVREMVQDLEVLPERNGIPREMGLMQILAAW

RKLWRRCRRLDALLKQFVDIFQWQVLFNLLTTYIFSIAVLFRLWIYLEFDKNFHLWKGILY
AIIFLTHHVEIVMQFSIFEINRCKWLGLLEDVGNLWDINYSGRQCIKSSGTILSRKLEFSLLY
MNRKLQLNPKRVRRLHIVGLFDLSNLTVHNMTRSIITNVLVLCQIAYKKYG
>DmelGR58a
MLLKFMYIYGIGCGLMPAPLKKGQFLLGYKQRWYLIYTACLHGGLLTVLPFTFPHYMYD
DSYMSSNPVLKWTFNLTNITRIMAMFSGVLLMWFRRKRILNLGENLILHCLKCKTLDNRS
KKYSKLRKRVRNVLFQMLLVANLSILLGALILFRIHSVQRISKTAMIVAHITQFIYVVFMMT
GICVILLVLHWQSERLQIALKDLCSFLNHEERNSLTLSENKANRSLGKLAKLFKLFAENQR
LVREVFRTFDLPIALLLLKMFVTNVNLVYHGVQFGNDTIETSSYTRIVGQWVVISHYWSAV
LLMNVVDDVTRRSDLKMGDLLREFSHLELVKRDFHLQLELFSDHLRCHPSTYKVCGLFIF
NKQTSLAYFFYVLVQVLVLVQFDLKNKVEKRN
>DmelGR58b
MLHPKLGRVMNVVYYHSVVFALMSTTLRIRSCRKCLRLEKVSRTYTIYSFFVGIFLFLNLY
FMVPRIMEDGYMKYNIVLQWNFFVMLFLRAIAVVSCYGTLWLKRHKIIQLYKYSLIYWK
RFGHITRAIVDKKELLDLQESLARIMIRKIILLYSAFLCSTVLQYQLLSVINPQIFLAFCARLT
HFLHFLCVKMGFFGVLVLLNHQFLVIHLAINALHGRKARKKWKALRSVAAMHLKTLRLA
RRIFDMFDIANATVFINMFMTAINILYHAVQYSNSSIKSNGWGILFGNGLIVFNFWGTMAL
MEMLDSVVTSCNNTGQQLRQLSDLPKVGPKMQRELDVFTMQLRQNRLVYKICGIVELDK
PACLSYIGSILSNVIILMQFDLRRQRQPINDRQYLIHLMKNKTKV
>DmelGR59a
MKRIGQAYNVYAVFIGMTSYETMGGKFRQSRITRIYCLLINAIFLTLLPSAFWKSAKLLSTA
DWMPSYMRVTPYIMCTINYAAIAYTLISRCYRDAMLMDLQRIVLEVNREMLRTGKKMNS
LLRRMFFLKTFTLTYSCLSYILAVFIYQWKAQNWSNLCNGLLVNISLTILFVNTFFYFTSLW
HIARGYDFVNQQLNEIVACQSMDLERKSKELRGLWALHRNLSYTARRINKHYGPQMLAM
RFDYFIFSIINACIGTIYSTTDQEPSLEKIFGSLIYWVRSFDFFLNDYICDLVSEYQMQPKFFA
PESSMSNELSSYLIYESSTRLDLLVCGLYRVNKRKWLQMVGSIVVHSSMLFQFHLVMRGG
L
>DmelGR59b
MVYWMIKLYFRYSLAIGITSQQFSNRKFFSTLFSRTYALIANIVTLIMLPIVMWQVQLVFQQ
KKTFPKLILITNNVREAVSFLVILYTVLSRGFRDTAFKEMQPLLLTLFREEKRCGFKGIGGV
RRSLRILLFVKFFTLSWLCVTDVLFLLYSTDALIWVNVLRFFFKCNTNNILEMVPMGYFLA
LWHIARGFDCVNRRLDQIVKSKSTRKHRELQHLWLLHACLTKTALNINKIYAPQMLASRF
DNFVNGVIQAYWGAVFTFDLSTPFFWVVYGSVQYHVRCLDYYLIDNMCDVAVEYHDSA
KHSWSEVRWTKEISSYVIYANSTKLQLWSCGLFQANRSMWFAMISSVLYYILVLLQFHLV
MRK
>DmelGR22a
MSQPKRIHRICKGLARFTIRATLYGSWVLGLFPFTFDSRKRRLNRSKWLLAYGLVLNLTLL

VLSMLPSTDDHNSVKVEVFQRNPLVKQVEELVEVISLITTLVTHLRTFSRSSELVEILNELLV
LDKNHFSKLMLSECHTFNRYVIEKGLVIILEIGSSLVLYFGIPNSKIVVYEAVCIYIVQLEVL
MVVMHFHLAVIYIYRYLWIINGQLLDMASRLRRGDSVDPDRIQLLLWLYSRLLDLNHRLT
AIYDIQVTLFMATLFSVNIIVGHVLVICWINITRFSLLVIFLLFPQALIINFWDLWQGIAFCDL
AESTGKKTSMILKLFNDMENMDQETERRVTEFTLFCSHRRLKVCHLGLLDINYEMGFRMI
ITNILYVVFLVQFDYMNLKFKTD
>DmelGR22e
MFRPSGSGYRQKWTGLTLKGALYGSWILGVFPPFAYDSWTRTLRRSKWLIAYGFVLNAAFI
LLVVTNDTESETPLRMEVFHRNALAEQINGIHDIQSLSMVSIMLLRSFWKSGDIERTLNELE
DLQHRYFRNYSLEECISFDRFVLYKGFSVVLELVSMLVLELGMSPNYSAQFFIGLGSLCLM
LLAVLLGASHFHLAVVFVYRYVWIVNRELLKLVNKMAIGETVESERMDLLLYLYHRLLDL
GQRLASIYDYQMVMVMVSFLIANVLGIYFFIIYSISLNKSLDFKILVFVQALVINMLDFWLN
VEICELAERTGRQTSTILKLFNDIENIDEKLERSITDFALFCSHRRLRFHHCGLFYVNYEMG
FRMAITSFLYLLFLIQFDYWNL
>DmelGR22d
MFRPRCGLRQKFVYVILKSILYSSWLLGIFPFKYEPKKRRLRRSMWLILFGVVISSSLLILM
VKQSAEDREHGIMLDVFQRNALLYQISSLMGVVGVVSICTVHLRTLWRSKHLEEIYNGLM
LLEAKYFCSNAVECPAFDGYVIQKGVVIVVGLLAPWMVHFGMPDSKLPVLNVLVVSMVK
LGTLLLALHYHLGVVIIYRFVWLINRELLSLVCSLRGNHKGSSSRVRFLLKLYNKLVNLYS
KLADCYDCQTVLMMAIFLAANIIVCFYMIVYRISLSKMSFFVMLIMFPLAIANNFMDFWL
SMKVCDLLQKTGRQTSMILKLFNDIENMDKDLEISISDFALYCSHRRFKFLHCGLFHVNRE
MGFKMFVASVLYLLYLVQFDYMNL
>DmelGR22b
MFGSSREIRPYLARQMLKTTLYGSWLLGIFPFTLDSGKRIRQLRRSRCLTLYGLVLNYFLIF
TLIRLAFEYRKHKLEAFKRNPVLEMINVVIGIINVLSALIVHFMNFWGSRKVGEICNELLIL
EYQDFEGLNGRNCPNFNCFVIQKCLTILGQLLSFFTLNFALPGLEFHICLVLLSCLMEFSLNL
NIMHYHVGVLLIYRYVWLINEQLKDLVSQLKLNPETDFSRIHQFLSLYKRLLELNRKLVIA
YEYQMTLFIIAQLSGNIVVIYFLIVYGLSMRTYSIFLVAFPNSLLINIWDFWLCIAACDLTEK
AGDETAIILKIFSDLEHRDDKLEMSVNEFAWLCSHRKFRFQLCGLFSMNCRMGFKMIITTF
LYLVYLVQFDYMNL
>DmelGR22c
MFASRSDLQSRLCWIILKATLYSSWFLGVFPYRFDSRNGQLKRSRFLLFYGLILNFFLLLKM
VCSGGQKLGIPEAFARNSVLENTHYTTGMLAVFSCVVIHFLNFWGSTRVQDLANELLVLE
YQQFASLNETKCPKFNSFVIQKWLSVIGLLLSYLSIAYGLPGNNFSVEMVLINSLVQFSFNC
NIMHYYIGVLLIYRYLWLINGQLLEMVTNLKLDCSVDSSRIRKYLSLYRRLLELKGYMVAT
YEYHMTLVLTTGLASNFLAIYSWIVLDISMNINFIYLLIFPLFLLVNVWNLWLSIAASDLAE
NAGKSTQTVLKLFADLEVKDIELERSVNEFALLCGHCQFNFHVCGLFTINYKMGFQMIITS

FLYLIYMIQFDFMNL
>DmelGR22f
MKMFQPRRGFSCHLAWFMLQTTLYASWLLGLFPFTFDSRRKQLKRSRWLLLYGFVLHSL
AMCLAMSSHLASKQRRKYNAFERNPLLEKIYMQFQVTTFFTISVLLLMNVWKSNTVRKI
ANELLTLEGQVKDLLTLKNCPNFNCFVIKKHVAAIGQFVISIYFCLCQENSYPKILKILCCLP
SVGLQLIIMHFHTEIILVYRYVWLVNETLEDSHHLSSSRIHALASLYDRLLKLSELVVACND
LQLILMLIIYLIGNTVQIFFLIVLGVSMNKRYIYLVASPQLIINFWDFWLNIVVCDLAGKCGD
QTSKVLKLFTDLEHDDEELERSLNEFAWLCTHRKFRFQLCGLFSINHNMGFQMIITSFLYL
VYLLQFDFMNL
>DmelGR47a
MAFTSSQLCSLLTKFTALNGLNTYYFDTKTNAFRVSSKLKIYCAIHHALCVLALAHMSYST
ASNLRVSVTVLTIGGTMACCVKSCWEKAQGIRNLARGLVTMEQKYFAGRPSGLLLKCRY
YIKITFGSITLLRIHLIQPIYMRRLLPSQFYLNVGAYWLLYNMLLAAVLGFYFLLWEMCRIQ
KLINDQMTLILARSGQRNRLKKMQHCLRLYSKLLLLCDQFNSQLGHVAIWVLACKSWCQ
ITFGYEIFQMVAAPKSIDLTMSMRVFVIFTYIFDAMNLFLGTDISELFSTFRADSQRILRETSR
LDRLLSMFALKLALHPKRVVLLNVFTFDRKLTLTLLAKSTLYTICCLQNDYNKLKA
>DmelGR85a
MYSLIEAQLLGGKLVNRVMASLRRIIQRSLGYFCALNGILDFNTDIGTGNLRRYRVLFMYR
LLHNFAVISLTLKFLFDFTDHFKYIESSTLITVNFFTYFTLVFFALLSSMGSCYQWQNRILAV
LKELKHQRDLSRHMGYRVPRSKQNSIDYLLFALTVLLILRLSIHLATFTLSARMGFNHPCN
CFLPECMIFSMNYLLFAILAEITRCWWSLQSGLKMVLLNRQLSTVAFNLWEIERLHTRFQC
LIDLTSEVCSIFRYVTLAYMARNLWSGIVAGYLLVRFVIGNGLQDVELVYLVFSFITCIQPL
MLSLLVNSMTSTTGSLVEVTRDILKISHKKSVNLERSIEWLSLQLTWQHTHVTIFGVFRINR
SLAFRSASLILVHVLYMVQSDYISITN
>DmelGR59e
MDSSYWENLLLTINRFLGVYPSGRVGVLRWLHTLWSLFLLMYIWTGSIVKCLEFTVEIPTI
EKLLYLMEFPGNMATIAILVYYAVLNRPLAHGAELQIERIITGLKGKAKRLVYKRHGQRTL
HLMATTLVFHGLCVLVDVVNYDFEFWTTWSSNSVYNLPGLMMSLGVLQYAQPVHFLWL
VMDQMRMCLKELKLLQRPPQGSTKLDACYESAFAVLVDAGGGSALMIEEMRYTCNLIEQ
VHSQFLLRFGLYLVLNLLNSLVSICVELYLIFNFFETPLWEESVLLVYRLLWLAMHGGRIWF
ILSVNEQILEQKCNLCQLLNELEVCSSRLQRTINRFLLQLQRSIDQPLEACGIVTLDTRSLGG
FIGVLMAIVIFLIQIGLGNKSLMGVALNRSNWVYV
>DmelGR59f
MRSSATKGAKLKNSPRERLSSFNPQYAERYKELYRTLFWLLLISVLANTAPITILPGCPNRF
YRLVHLSWMILWYGLFVLGSYWEFVLVTTQRVSLDRYLNAIESAIYVVHIFSIMLLTWQC
RNWAPKLMTNIVTSDLNRAYTIDCNRTKRFIRLQLFLVGIFACLAIFFNIWTHKFVVYRSILS
INSYVMPNIISSISFAQYYLLLQGIAWRQRRLTEGLERELTHLHSPRISEVQKIRMHHANLID

FTKAVNRTFQYSILLLFVGCFLNFNLVLFLVYQGIENPSMADFTKWVCMLLWLAMHVGK
VCSILHFNQSIQNEHSTCLTLLSRVSYARKDIQDTITHFIIQMRTNVRQHVVCGVINLDLKFL
TTLLVASADFFIFLLQYDVTYEALSKSVQGNVTRYK
>DmelGR9a
MSLWLEHFLTGYFQLCGLVCGWSGSRLGRLLSSTFLVLILIELVGEIETYFTEENPDNESVP
AYFAKVIMGVNMAYKMIHAWIALSALFECRRFRYLLEELPPVKATSFIYRHLILEIILFACN
AFLVLSEYTIRGIYLENLRYAYSLQAVRARYLQMMVLVDRLDGKLEQLHHRVISGSSDYKT
LRLDYAHLAKVTRSLSHLFGLSLLLLNVLCLGDWIIVCNVYFMVAYLQVLPATLFLFGQV
MFVVCPTLIKIWSICAASHRCVSKSKHLQQQLKDLPGQTPVERSQIEGFALQIMQDPIQIDV
CGIYHLNLQTLAGMFFFILEALVIFLQFVSLVRT
>DmelGR77a
MPLPLGDPLALAVSPQLGYIRITAMPRWLQLPGMSALGILYSLTRVFGLMATANWSPRGIK
RVRQSLYLRIHGCVMLIFVGCFSPFAFWCIFQRMAFLRQNRILLMIGFNRYVLLLVCAFMT
LWIHCFKQAEIIGCLNRLLKCRRRLRRLMHTRKLKDSMDCLATKGHLLEVVVLLSSYLLS
MAQPIQILKDDPEVRRNFMYACSLVFVSVCQAILQLSLGMYTMAILFLGHLVRHSNLLLA
KILADAEHIFESSQKAGFWPNRQELYKGQQKWLALELWRLLHVHHQLLKLHRSICSLCAV
QAVCFLGFVPLECTIHLFFTYFMKYSKFILRKYGRSFPLNYFAIAFLVGLFTNLLLVILPTYY
SERRFNCTREIIKGGGLAFPSRITVKQLRHTMHFYGLYLKNVEHVFAVSACGLFKLNNAIL
FCIVGAILEYLMILIQFDKVLNK
>DmelGR10b
MRVGKLCRLALRFWMGLILVLGFSSHYYNPTRRRLVYSRILQTYDWLLMVINLGAFYLY
YRYAMTYFLEGMFRRQGFVNQVSTCNVFQQLLMAVTGTWLHFLFERHVCQTYNELSRIL
KHDLKLKEHSRFYCLAFLAKVYNFFHNFNFALSAIMHWGLRPFNVWDLLANLYFVVYNSL
ARDAILVAYVLLLLNLSEALRLNGQQEHDTYSDLMKQLRRRERLLRIGRRVHRMFAWLVA
IALIYLVFFNTATIYLGYTMFIQKHDALGLRGRGLKMLLTVVSFLVILWDVVLLQVICEKLL
AEENKICDCPEDVASSRTTYRQWEMSALRRAITRSSPENNVLGMFRMDMRCAFALISCSL
SYGIIIIQIGYIPG
>DmelGR89a
MLRFPHVCGLCLLLKYWQILALAPFRTSEPMVARCQRWMTLIAVFRWLLLTSMAPFVLW
KSAAMYEATNVRHSMVFKTIALATMTGDVCISLALLGNHLWNRRELANLVNDLARLHRR
RRLSWWSTLFLWLKLLLSLYDLLCSVPFLKGAGGRLPWSQLVAYGVQLYFQHVASVYGN
GIFGGILLMLECYNQLEREEPTNLARLLQKEYSWLRLIQRFVKLFQLGIFLLVLGSFVNIMV
NIYAFMSYYVSLHGVPLTISNNCLVLAIQLYAVILAAHLCQVRSAKLRKKCLQLEYVPEGLT
QEQAMASTPFPVLTPTGNVKFRILGVFILDNSFWLFLVSYAMNFIVVILQTSFEHINHGEI
>DmelGR92a
MFEFLHQMSAPKLSTSILRYIFRYAQFIGVIFFCLHTRKDDKTVFIRNWLKWLNVTHRIITF
TRFFWVYIASISIKTNRVLQVLHGMRLVLSIPNVAVILCYHIFRGPEIIDLINQFLRLFRQVSD

LFKTKTPGFGGRRELILILLNLISFAHEQTYLWFTIRKGFSWRFLIDWWCDFYLVSATNIFIH
INSIGYLSLGVLYSELNKYVYTNLRIQLQKLNTSGSKQKIRRVQNRLEKCISLYREIYHTSIM
FHKLFVPLLFLALIYKVLLIALIGFNVAVEFYLNSFIFWILLGKHVLDLFLVTVSVEGAVNQF
LNIGMQFGNVGDLSKFQTTLDTLFLHLRLGHFRVSILGLFDVTQMQYLQFLSALLSGLAFI
AQYRMQVGNG
>DmelGR93c
MIERLKKVSLPALSAFILFCSCHYGRILGVICFDIGQRTSDDSLVVRNRHQFKWFCLSCRLIS
VTAVCCFCAPYVADIEDPYERLLQCFRLSASLICGICIIVVQVCYEKELLRMIISFLRLFRRVR
RLSSLKRIGFGGKREFFLLLFKFICLVYELYSEICQLWHLPDSLSLFATLCEIFLEIGSLMIIHI
GFVGYLSVAALYSEVNSFARIELRRQLRSLERPVGGPVGRKQLRIVEYRVDECISVYDEIER
VGRTFHRLLELPVLIILLGKIFATTILSYEVIIRPELYARKIGMWGLVVKSFADVILLTLAVHE
AVSSSRMMRRLSLENFPITDHKAWHMKWEMFLSRLNFFEFRVRPLGLFEVSNEVILLFLSS
MITYFTYVVQYGIQTNRL
>DmelGR93b
MSGLLVMPRILRCLNVSRISAILLRSCFLYGTFFGVITFRIERKDSQLVAINRRGYLWICLVIR
LLASCFYGYSYDAWSGQYEDMYLRAFFGFRLIGCLICSVIILVMQFWFGEELINLVNRFLQ
LFRRMQSLTNSPKNRFGDRAEFLLMFSKVFSLLFVFMAFRLMLSPWFLLTLVCDLYTSVGT
GMITHLCFVGYLSIGVLYRDLNNYVDCQLRAQLRSLNGENNSFRNNPQPTRQAISNLDKC
LYLYDEIHQVSRSFQQLFDLPLFLSLAQSLLAMSMVSYHAILRRQYSFNLWGLVIKLLIDV
VLLTMSVHSAVNGSRLIRRLSFENFYVTDSQSYHQKLELFLGRLQHQELRVFPLGLFEVSN
ELTLFFLSAMVTYLVFLVQYGMQSQQI
>DmelGR93a
MFSSSSAMTGKRAESWSRLLLLWLYRCARGLLVLSSSLDRDKLQLKATKQGSRNRFLHIL
WRCIVVMIYAGLWPMLTSAVIGKRLESYADVLALAQSMSVSILAVISFVIQARGENQFREV
LNRYLALYQRICLTTRLRHLFPTKFVVFFLLKLFFTLCGCFHEIIPLFENSHFDDISQMVGTG
FGIYMWLGTLCVLDACFLGFLVSGILYEHMANNIIAMLKRMEPIESQDERYRMTKYRRMQ
LLCDFADELDECAAIYSELYHVTNSFRRILQWQILFYIYLNFINICLMLYQYILHFLNDDEV
VFVSIVMAFVKLANLVLLMMCADYTVRQSEVPKKLPLDIVCSDMDERWDKSVETFLGQL
QTQRLEIKVLGFFHLNNEFILLILSAIISYLFILIQFGITGGFEASEDIKNRFD
>DmelGR93d
MKATKYSVGILRFMSFYARFLSLVCFRLRKQKDNNVWLEEIWSNRSRWKWISVTLRIVPL
CIYAFTYAEWISNRMLITEKFLHSCSLVVSIPCYLSIIHLKICHGPEVTKLVNQYLHIFRLGTL
DIRRRSQFGGGRELFLLILSVCCQIHEYVFILVIASRLCGFQHIIWWVSYTYVFIICNSIMCFG
FIWHLSLGVLYAELNDNLRFESGFQTAFLRKQQRIRVQKSMALFKEISSVVTSLQDIFNVHL
FLSALLTLLQVLVVWYKMIIDLGFSDFRIWSFSLKNLIQTLLPVLAIQEAANQFKQTRERAL
DIFLVGKSKHWMKSVEIFVTHLNLSEFRVNLLGLFNVSNELFLIIVSAMFCYLVFVTQCVIV
YRRRYVI

>DmelGR94a
MDFTSDYAHRRMVKFLTIILIGFMTVFGLLANRYRAGRRERFRFSKANLAFASLWAIAFSL
VYGRQIYKEYQEGQINLKDATTLYSYMNITVAVINYVSQMIISDHVAKVLSKVPFFDTLKE
FRLDSRSLYISIVLALVKTVAFPLTIEVAFILQQRRQHPEMSLIWTLYRLFPLIISNFLNNCYFG
AMVVVKEILYALNRRLEAQLQEVNLLQRKDQLKLYTKYYRMQRFCALADELDQLAYRY
RLIYVHSGKYLTPMSLSMILSLICHLLGITVGFYSLYYAIADTLIMGKPYDGLGSLINLVFLSI
SLAEITLLTHLCNHLLVATRRSAVILQEMNLQHADSRYRQAVHGFTLLVTVTKYQIKPLGLY
ELDMRLISNVFSAVASFLLILVQADLSQRFKMQ
>DmelGR97a
MRFLRRQTRRLRSIWQRSLPVRFRRGKLHTQLVTICLYATVFLNILYGVYLGRFSFRRKKF
VFSKGLTIYSLFVATFFALFYIWNIYNEISTGQINLRDTIGIYCYMNVCVCLFNYVTQWEKT
LQIIRFQNSVPLFKVLDSLDISAMIVWRAFIYGLLKIVFCPLITYITLILYHRRSISESQWTSV
TTTKTMLPLIVSNQINNCFFGGLVLANLIFAAVNRKLHGIVKEANMLQSPVQMNLHKPYY
RMRRFCELADLLDELARKYGFTASRSKNYLRFTDWSMVLSMLMNLLGITMGCYNQYLAI
ADHYINEEPFDLFLAIVLVVFLAVPFLELVMVARISNQTLTRRTGELLQRFDLQHADARFKQ
VVNAFWLQVVTINYKLMPLGLLELNTSLVNKVFSSAIGSLLILIQSDLTLRFSLK
>DmelGR10a
MTSPDERKSFWERHEFKFYRYGHVYALIYGQVVIDYVPQRALKRGVKVLLIAYGHLFSM
LLIVVLPGYFCYHFRTLTDTLDRRLQLLFYVSFTNTAIKYATVIVTYVANTVHFEAINQRCT
MQRTHLEFEFKNAPQEPKRPFEFFMYFKFCLINLMMMIQVCGIFAQYGEVGKGSVSQVRV
HFAIYAFVLWNYTENMADYCYFINGSVLKYYRQFNLQLGSLRDEMDGLRPGGMLLHHC
CELSDRLEELRRRCREIHDLQRESFRMHQFQLIGLMLSTLINNLTNFYTLFHMLAKQSLEE
VSYPVVVGSVYATGFYIDTYIVALINEHIKLELEAVALTMRRFAEPREMDERLTREIEHLSLE
LLNYQPPMLCGLLHLDRRLVYLIAVTAFSYFITLVQFDLYLRKKS
SNMPs
>ItypSNMP2
MRFLQRVKFNLKTVFLCGISGVSLLVVALFLGFIIFPKVVNDQLLETKILREDTEQWAIFKKI
PFAFTFNVYLFTVENPEEILKGAKPVVKEKGPYVYKLYKWKEDIIWNYTTDEISYYEYEK
YVFDQEASGSLTEHDKVTLLNLPYLTFLYTAEANEATSGFLPLIDEALEFIFSGHNSPFLVNV
TVRDYLFEGVEICKNGCEDDGFVAKMACGKIKDNLKVAKQMRLHHKDILFATFHYRNNT
HQKYLTVNSGRQNHLEIGAITQLDNSSTMNVWNQFGCNQVSGLTGIFPINLGFKTTFQSFS
AEICRPVKLHFSTIKPFGSIKGYKYVALNTTFNTSMVENQCYCTGKIPNLDGNLGCLYDGV
LDLSTCLGAPIVVSFPHFLYADWRYVNNVKGLSPNETNHQIFVNLEPISGTPLEAATRIQFN
LFLRPVRNITSLDSVADALVPLFWIEELTYLPQKYQDVITGKLYRSIFILNAIKYVLLAIALVI
ITVCILIFLYTD
>ItypSNMP1
MPHPKNIAWAGGALAFGGVLFKVWLFDVLVRFGVKDQTALRYRNEVRGIYLKIPFPLNFK

IYFFNVTNPEEIQNGAKPVLNEVGPYWYDEYKERVDVIDNDTEDSLTYTPYDLFKFNPNM
STPLSDNDYVTIIHPVIVGMVNLLLRDSPMLLKVVSKAIPFIFNDPKTIFLTGRVKDILFDGV
VLNCTSKEFASTAVCGQMKGQVPGLKPTPGQPNLLLFSLLGPRNATRTGSLKVLRGIKHFQ
DLGRLLEVNGRKSIGIWAGDQCNRYDGTDSWIFPPLIQPESGLKSFSTDLCRNIKMKLVNE
TVVKKIPVGVFEPTWGVKVVTRRKSATVPTLPVXXXXXVFDLTKCMGVPLYATLPHFLDT
DPNYLKLVDGLKPDHEKHRIVVFFETMTGTPLKAAKRMQFNLELQQTNKLELFSKLPAAL
FPIFWLEEGMELEGYFLKKIQTVFMLLLFADVTIYVTIATGLSVCGAGFYQYWKNTKSLSI
TPLTKNNNGLSEPKLN
>PverSNMP1a
MQLPLKIAIGSLSALLLTIIVGFIAFPKLITSKVKGMINLGPGTDIRDMFLKVPFPLTYRVYIF
NVTNPDRIQKGDMPVVNEVGPFCYEEWKEKMNVEDMEADDTIAYDPKDTFLKKRWPGC
KTGKEIITVPHPMILGLVNTVARQKPGALSLANKAIKSIYANPSSIFITTEADNILFDGVIINC
GVTDFAGKAICSQLKSSGNLKLINGDQLLFSLLGPKNATLNTRMKAYRGKKHFQDVGRIV
EFGGAKNLDVWPTDECNEIKGTDGTIFPPFLKKEQGLVSYSPDLCRSLRATFVKDTVYDGI
PCAEFTATLGDMSKNEDEKCYCLTPDTCMKKGIMDLYKCAGVPVYASLPHFYGTDKSYL
DGVNGLTPNKSKHEIKILFESTTGSPLYARKRIQLSMPLEPIQKVELFMNFTPTVIPVLWIEE
GVELNRTYTGQLKSLFTMKKIVGAFKWVVLLSSLGGLAAAGYMFYKNNGKIEITPIHESK
RDGISTIHSLEGQVNHGMSENSIDKF
>PverSNMP2a
MLACSRFFSNKVLAVLTTILTLIFVGVLVLAFYGIPVIINKSIHNSVHLEKGTIQWDRFVDLP
VDILMKVFLYHVTNPDDVLNGAKPIVEERGPYCYKQNIHKNILSTSSSQDTVTYEQNFKIE
FDQEASGNLKESDKVVIVNPVMLTLYKLTSRLERLVVFGCLDKIFPKEYIGVFIEVDVKTV
MFDGFAFAQRSEDLGPACNIVRNQILDKTLPMKNVERITDDDGILELRFALLQYKIRGPDG
NYTINRGIDDITKLGHIIKWNDETELPFWGRMQSINNDTCKKVRGSDSTIYPPQVDKTRSF
DIFSTDICRVVEISFQRTDTYNGIDAYRFGITKNTFRSATTNPENDCYCIKQSAGIDGEPSCY
LDGVLDVYPCFGAPILLSFPHFLYADESYVDAIEGIGPSDPDIHELFLLIEPNTGTPLQGMKR
VQLNTVLMPMQNIPGTSKISPLVMPILWLEEGVSLPQNLIDELNSHYFQTVKLVEGIIYGLI
AVVAASVLISSGFLI
>PverSNMP2b
MVFNLKFNLSKKTLKILGCFGLFLACAGVYFGYKALPDIVTDKIWEMKVLKENTQQWD
MFKKMPFPFTFKVFIFDIKNPDEIMQGAKPTIKEIGPFVYKVYKWNSDIKWESPDDISYFAY
TRFEFDEEASGRFTEDYIVTILNTPYLGMLLKVADIQAAALPMVEGVLGDIFKENDGLFIK
VKVKDYLFQGLKMCENEGKDGDFAAGLVCKQVIAEAATSNNLRVENNTILFANLHYKNN
THLGRFTIKAGIQNHNEIAHLALYNNQSYISIWGEEKSICNKIEGLSTTVFPVNINKDMIFES
FAEDICRRMKLTYKMDETVKGLKGYKFTAANDSFSMKNENNTCYCNKKTTLMDGKLGC
VKDGITDLSTCTGGPVMVSFPHLLYADKEYLNSVEGLDPDSMKHESFVVLEPMSGFPLSL
AQRVQFNIFLRPIDESTILANVSRALFPLIWVEESLQLDDKFTDMLKNNLFKTLDMINILKW

VVIASGSACFLFAVSMAIYNDAS

>PverSNMP1b

MQLAIKVLVSGVGITISSVIFALVIYDPLIKYVIRDQTSLKKNNEIRDIYLKIPFPLDFRIYLFN

VSNPMEVQDGAKPVLKEVGPYCYNEYEEKVDVIDNEMEDSLTYNSYDIFRFNANKSIGLS

ENDYVTIIHPLIVAMAYQVNRDTPALLSFLNQAIVTIFKNPKSIYLTDTVKNILFDGFEINCN

VTEFAAKAVCTQIMNSNIPGLKTDPSRNNTLVFSLFGARNATLGHTMKVLRGIKRSEFVGK

VLEVDGKKEMNLWTSKACNRYRGTDGWIIPPLLEPGVGVWTHSVDMCRNVEAKYIKET

VLNGVNARLYEADLGDMQKNEDEKCYCLTPSTCSRKGTFDLTKCMGAPIIASLPHFLRAD

EIYRQQVDGMQPVHEKHIISIYLEGVTSAPLRATKRMQLNFPITTIPKLTLMTKLPEALHPL

LWLEEGVEVEGEFLKLITDKLMLLNVANYGRWLAVFGGLITTGVGVYLHNKNKNSVAIST

IHSGDIDREITRSTNELMDQMNRIQGNEKGHVNHVLSGHEFDRYM

>GdauSNMP1a

MKLQLSVKLAIGSFCTLFFIILVGFIMFPKMITSKVKAMVNLGPGMEIREMFLKVPFPLSFK

IYLFNVTNPMAIQSGDIPEVKEIGPFCFQEWKRKVDVTDEEENDIISYLSIDTFTRVSGPGCV

SGKVMVTIPHPMILGIVNAVSRAKPGALALINKAFKSVYDNPTSIFLTATADDILFDGVIIKC

GVTDFAGKAICSQLRDSGSLKIVNEKELAFSLIGPKNGTEQKRIKALRGTKNYHDVGRIVE

YDESPVMSTWPTDECNEISGTDGTVFPPMLLKEEGLVSFAPDLCRSLKAFWVRKTKYDGI

PVNEYTASLGDMSKNENEKCYCYTPETCLKKGLMDLYKCAGVPIYVSMPHFYDSDESYV

KGVKGLQPNKTQHQISILFEQLTGGPVSAKKRLQFSMPLEPNQKVELFKNFTPTVIPIFWV

EEGVDLNRTFTKPIKTLYTMKKVVKISKWLILLASTAGLITSGYLFFKSNQTVSITTVKDFK

KTAPASGISTVNGHINGSMIGNEVDKF

>GdauSNMP1b

MKVSLKYIFSGGFVFLATILVGFVAFRELVELAVKDQTSLRERNEIRGIYLKIPFPLNFKIYFF

NVTNPMEVQNGATPILQQVGPYYYDEYKEKINVIDNDAQDTLQYDSFDTYIFNKTLSGKL

SDEDYVTIIHPLLVGMVNAVTASMPALLSILNQAIPYIFHEPKSIYLTDKVKNIVFNGMELN

CQGSNFASKAVCTQLKSQIPGVKESTTQKNVLLYSLFGNRNATVGDTIKIMRGIKNNKDLG

RVLEVNGKSHLDLWSSDECNRFKGTDGWIIPPLLNPEDGIHCYSPQLCRNIALDYMKDDVI

KGINVRRYEANFGDQQTVEADKCFCPNPKPCLKKGVFDLSKCVGAPIMVTLPHFLYADET

LLQQVKGLKPIREEHILTVSIEPLTSAPLNVKMRIQMNLDIGPNQKITIMNNLTTALHPIFWL

EDSLDLEGPLLTKISSIFVLLKVTYVIKWILLVISIGLFAFGGYLHFKSRKSVKITPVHQRPEN

EVDALTRKTNEILSQITKVEKIGHTNSIMSGHEFDRYN

>AchiSNMP1

MILSHKLSMAGGSLIVFSVVFGFLAFEQILKFGIRDQTALRKRNAIRSLYLKLPFPLDFHVY

FFNVSNPMEVQTGSIPILEEIGPYCYDEYVEKVDVVDNDGDDSLTYSPYSVYKFNQEKSGI

LRDDDYVTVIHPLIIGMVNLVSRDMPALLPIVNKAIGLIFPDLESIYLTAKVKDILFDGMAIN

CKVTEFPAKAVCTQIKSKIPGIKTTGNEVYLFSLLGPRNATKGKRVKVLRGISKSKDLGKIL

EVDGKREIHLWGTPECNTLRGTDGWIFPPLLEPEEEISTYVSEMCKSVSAAPAGTTVLKGI

NVLHYDMDLGDMDHNEDEKCYCDTPKTCLKKGVFDLSKCMGVPIYVTLPHFLKTDEIYF
KQVKGMHPLPEKHKLEVFFEPMTAAPIAAYKRIQFNLPIAPNNKITLMKNLPEALHPLLWV
QESVDLEGPLLKKVKSIFVVVKVIKVARWFGIAVGIAIIGFAVFHYFNNKKEVKITPVHHPQ
SVSNSLTESNSNRASIKESNIDKRKVRSILNGHEFDRY
>AchiSNMP2
MRMVDNTVLFSNLYYKNVSHQGRFTINSGKDNSEETGVLKQFNGKTYISTWLGEKSICN
KIRGVTTVFPAQVKKSMVFESFAEDICRAMSLRFEKEKKVKGVLGYKFVAANDSFSVAND
DNSCYCVNKSKTLEGEFGCLRDGLGDLTTCTGAPVLVSFPHLLHADPEYISSVVGLQPNAS
KHETFLTLEPISGFPLELAQRVQFNMFIRPFEAISSLENVTKALIPLIWVEESTVLGDKYVDK
LKNELFKNLMILDVIKWGFIGIGVALVMSAFFLFIYVKSP
>AchiSNMP3
MSPVKRSSILSFIFPKAYLVRYVVDTIRRFYNLMVQSATKIKMKLPVKLGIGSSVLFVFIVLV
GFVVFPKMITSKIKSMVNLKPGTEIRDMFLKIPFGLEFRVYIFNVTNPMEVQRGQAPSLKE
VGPFCYEEWKEKVDVQDMEGDDTILYNAKDTFIQVMWPGCLSGTEVVTIPHPMILGMVN
TVVIQKPGALTLVNKAIKSIYSNPASIFLTAKANDILFDGVIINCDVKDFAGKAICSQLKEAP
TLRHVSENELAFALLAPKNATPGKRIKAARGVNNFKDVGRILEYDGVDKIDVWPTDECN
AIRGTDGTIFPPLLSEEEGLVSFAPDLCRSLVAEFQQKTKYDGIPVRKYSATLGDMSKNEDE
KCYCPTPETCLKKGIMDLYKCIGVPIYVSLPHFYETHESYLKGVKGLRPDKSKHEIIILFEG
MTGGPVYAKKRLQFNMPLQANPKVDIFNNFTESVLPIFWVEEGVELNNTFTKPLKDLFKI
QKIVKITTWTVLLGSLLGLSAAGYLFFKESGTADITPVHKVHPSDSRKTISTVSGNNLEGID
NHAMTKTDTD
>PmacSNMP1b
KEKINVIDNDAQDTLQYDSFDTYIFNKTLSGKLSDEDYVTIIHPLLVGIVNTVNATSPALLSI
VNQAINKLFKEPKSIYLTDKVKNILFDGMEINCRENDFASKAVCTQLKSQISDIKESTTQKN
VLLYSLFGQRNATIVDTIKIMRGIKNYRDLGRVLEVNGKSDINLWGSEHCNRFKGTDGWII
PPLLKPEEGIRCYATQLCRNIALDYVEDEIIKGFNVRRYEGNLGDQQLVEADKCYCPSPKR
CLRKGVFDLSKCLGAPIIASLPHFLYADEIFLSQVRGLEPIKENHVLAVTIDPISSAPLSLRMR
IQMNLEIGPNPKITVMNNLTDALHPIFWLEDSLDMEGPVLKKVSEIYVAIKVANVLKWFAL
AIFFVFFAFGIYLHFKNRKSAKITPVQRRPASDKDAFHKRTNGGQNNIMSGHEFDRYN
>PmacSNMP1a
MKLQLSVKLAIGSICAMFFIILVGFILFPRMITSKVKAMVNLGPGTDIRGMFLNVPFPLSFK
VYLFNVTNPMEIQTGAIPKLKEVGPFCFEEWKKKIDVTDEEEDDIITYLSVDTFLKKSGPG
CVSGKVIVTIPHPMILGLVNAVSRAKPGALALINKAIKSVYENPTSIFLTSTADEILFDGVIIK
CGVSDFAGKAICSQLRESGSLKIVNENDLAFSFLGMKNGTEQKRIKALRGTKNYHDVGRI
VEYDGSPVMSTWPTDECNKIAGTDGTVFPPMLLKEEGLVSFAPDLCRSLKAFWVQKTKY
DGIPVNEYTASLGDMSKNENEKCYCYTPDTCLKKGLMDLYKCAGVPIYVSMPHFYDSDE
SYLKGVKGLNPNKTSHEISILFEQLTGGPLSAKKRLQFSMPLEPIQKVDLFKNFSNNVIPLF

WVEEGVDLNNTFTKPIKMLYTMKKVVKISKWLILLSSIAGLVASGYLFFKSNQTISITTVK
DIKKGPMSGISTVNRSMSDNEVNKF
>PaenSNMP1b
TQLCRNIALDYVEDEIIKGINVRRYEGNLGDQQLVEADKCYCPSPKHCLKKGVFDLNKCL
GAPIIASLPHFLYADEIFLTQVKGLEPIKEDHVLAVSIDPISSAPLSLRMRIQMNLEIGPNPKIT
VMSNLTDALHPIFWLEDSLDMEGPVLKKVSEIYIAIKVANILKWFALVMFFVFFAFGIYLHF
KNRKSAKITPVQRRPASDKDTFYKSTNEGQNNIMSGHEFDRYN
>PaenSNMP1a
MKLRLSVKLAIGSICAMFFIILVGFILFPRMITSKVKAMVNLGPGMDIRGMFLNVPFPLSFK
VYLFNVTNPMEIQTGAIPKVEEVGPFCFEEWKKKVDVTDEEEDDIITYLSVDTFLKKSSPG
CVSGKVIVTIPHPMILGLVNAVSRAKPGALALINKAIKSVYENPTSIFLTSTADEILFDGVIIK
CGVTDFAGKAICSQLRESGSLKIVNENDLAFSFLGMKNGTEQKKIKALRGTKNYHDVGRI
VEYDGSPVMSTWPTDECNRIAGTDGTIFPPMLLKEEGLVSFAPDLCRSLKAFWVQKTKYD
GIPVNEYTASLGDMSKNENEKCYCYTPDTCLKKGLMDLYKCAGVPIYVSMPHFYDSDES
YLKGVKGLNPNKTSHEISILFEQLTGGPVSAKKRLQFSMPLEPIQKVDLFKNFSTTVIPLFW
VEEGVDLNNTFTKPIKMLYTMKKVVKISKWLILLSSIAGLVASGYLFFKSNQTISITTVKDI
KKGPTSGISTVNGHMNRSMSDNEVNKF
>DponSNMP1
MMLSNKIWSSSRFLYGSVILLVSSVLLKLWLFESMVKFVIRDQTALRKRNQVREVYLKIPF
PLNFKLYFFNVTNPEEIQTGSKPKLKEVGPFWYDEIKEKVQIIDNDTEDSLTYTPYDLFEYN
QNKSNQLREDDYVTIIHPAIVGMVNLVLRDSPVFLSIVSKAIPSIFNNPQTIFLTAKVKDILFD
GVELNCLGKDFGTTAVCSQMKSQIPGLKFKKDNENIFLFSLLGSRNGTLTRRLKVHRGIAH
AKDLGRLVELDGKKEINIWRQAECNRFHGTDGWIFPALSTPEEGLPSFSTDLCRSVNLRYI
NDTVLKKIPVRIYETDLGDQMTDENEKCYCRSADSCLKKGVFDLSKCMGVPIYATLPHFL
RTDPSYINLVDGLAPSELLHAIRVYFEPMTGTPLFAAKRMQFNLDLKPTNKIPLFSHLPTAL
FPMFWLEESVDLDGYLLKKVQTVFLLLHAVDIIQYLMIVIGCGCVTISMYFRLKNRKSVTI
TPATGSKKSAPPKPIDEMDVSHLSIAGILGDRPQKKAVVSQVMSGHEFDKY
>DponSNMP1a
MNFPMRLAIGSACSLLFIILVGFVGFPKMIKGKVKDMVNLKPGMEIREMFVKVPFPLSFNV
YIFSVLNPAEVQGGAKPHLKEMGPFCYNEWKTKINVEDNEGDDTISYDPVDTFENAKRPK
CLSVDTLVTIPHPMILGMVNTILRQKPGALTLANKAIKSIWSNPSSLFITVKAQDLLFDGVV
IHCGVSDFAGKAICTNLKAEPSLTHLGEDDLGFSLMGPKNGTAGKRIKAFRGTQDFHKVG
RIIEFDGKSKLDVWNNSKCDTIVGTDGTIFPPMLKKEEGLASFAPDLCRSLIAQFDKHDKY
DGIPVSSFFASLGDQSKNPAEKCFCTTPETCLKRGLMDLYRCAKIPLYVSLPHFYDSHESYL
KGVKGLKPDVEKHGIRIMFELLTGSPLSARKRLQFNMPLEPNPKVELFHNFTPTVLPIFWV
EEAVDLNSTFTKPLKTLFLTKKLVNIVKYLVLLMSIGGFCAAVYLYFKSDDSMNVTSVQKV
QPDQNGHRNIISTVFNGNHTAGQDNEAYEDKY

>DponSNMP2
MFRNCCSPRLVFLYNLLAVLLLIASLVLAFWGLPQIISKQIHKQTELTENTDQWDRFKELPF
PMEFNIRFFLVTNPADVLNGSMPILKESEPYKYKSTIKRTDIRFDDIEEDSVTYRRSFSFEFD
GSGTTREDDSITVINPLLMASFQLTNDIQRLAMAGCRKYILEPAGLDQVFLTTTVRKLLFD
GIYFGFQNATGKGVACEMVRKELGKIVANVRVVEHLNDTDCYRLAIFNYKTDNFLKNSPD
GIYTINRGRNNATALGSIMRWNGATTSTTYGTSTSINNLTCHSIKGTDSTIYSPELKAGENL
MIFNTDLCRTIQLVQVSSNEVFNGINAFRYSTGYTLFRPETILKENDCYCSHGTKGADGKP
SCFLDGLLDFRPCLGAPVLISQPHFLHADVKYIRAVSGLSPDEDKHDIYLLLEPNTGTPLEG
RKRVQMNSVLRRQPLLSMITPPNMYEAVVPLLWLDEGFTLPQKYLDDLNAKYFKTVRIAT
GFKFGFIAVALALLVGCLFVACRKMYFRNAK
>BmelSNMP1b
MLLHKKLFIIGGAIAASTIVFLFLAFTPILHAGIADQAALRKRNEIRQIYLKLPFPIDFSVYFF
NVTNPMEVHNGAKPVLKEVGPYTYDEYVEKVDVVDNEAEDSLSYLPFHTFYFNQEKSG
HHSDNDVVTIINPLIVGMVNLVTKESPALLSIINQAIAPLFRNPESIYLTARVKDILFDGMEID
CTSTEFAPKAVCLQLKSQIPGIEKLPDKNVYLFSLMGPRNATTGKRMKVSRGIRSIKELGSV
LEYDGKKEINLWNSRECNRFRGTDGWIVPPLLKQEEGIWIYSTDMCRNLHGYYVSDDVIK
GVFVNRYEGDLGDMTTNEEERKCYCSTPDTCMRKGVFDVSKCMRVPIYVSLPHFYGTDE
YYMNLVEGLQPNKSKHKIVLLLESLTSAPLIAAKRAQLNLLIAPNPKITIMTNLPEALHPIF
WMEESIILEGPILKKVQVVGLLLKITNIFRWILLVAGLGIIGFGGYLFIKSKNNSKILHINSAV
NGITTVKSAKKIQKPQNGLENGGFEFDDASTKEETDGNKKKGVMTGHEFDRFQ
>BmelSNMP1a
MKSVVLRAVWLSFQQLELFSSVRVILNKFSVDANRTKKGTMKLPTKLGIGCLFALVFIIAF
GFIGFPKLLTGKIKQMVNLGPDTEIRKMFVNVPFGIDFNIYIFNVTNPMEIQSGAKPRVQEV
GPFCYSEWIKKINVDDEEGDDTMSYNPVFTFYKRKGPGCVSGQEVVTIPHPLILGMVNAV
VRQKPGALSLVNKAIKSIYQNPTSIFLTASADDILFDGVPINCGVKDFAGKAICSQLKEADS
LRHIDDDTLAFALMAPKNGTPGKRFQVLRGTTNFHYVGKILNYDGSPVITTWPNEECNKI
DGTDGTIFPPFLTKEEGIASFSPELCRSLKARYVKNTRYNGVPVRLYTADLGDASQNEDEK
CFCYTPDTCLKKGLMDLYKCVGVPIYVSMPHFWNCHKSYLKGVDGLKPNKTAHEITILFE
SMTGGPVAAGRRLQFNMPLWPNEKVDLFKNFTDTVLPMFWVEESIKLNRTMTKPLKRLF
TVKRIMNKLKWLVLLSSIGGMGVAFYLHLRGEGKAGVTPIEKVKNEEKGISIVNSNSVNG
HDNPAMTTDESDKF
>BmelSNMP2b
MLNKVNCVVKQSTVLFLAVLGVFFVCAGSYFGFKIIPDYVTSKMWEMKVLRNSTEQWD
MFLKVPFPFAFKVYVFDIQNPNDILQGTKPIVKERGPYIYKEFKYNTDIEWNASRISFYQNR
RFYFDRKASMGYDDEDFITTLNTPYLNILYKIQDQQPGALGMIADALPAMFGEFNDLFIKV
KLRDYLFDGLKICEDGGKAGGFVAGMVCKQIAAKLSEAPAMRMIDNTILFATMHYKNTT
RVGKFTIESGIDNTPETAVLKEFEESENISVWEEHSTCNKVGGVTTVFPVSIDKSMVFEAFS

ADVCRTMALEYQREETIKDILGYRFVAKNNSFNYSVPENSCFCLNTTKTLSEEPACAKDG
VMDLSICTGGPVFLSFPHLLYADKEYSSTLEGLHPDPDEHETFVTLDPVSGTPLMLAQRVQ
FNMFTRPLEGFSVMENVSKALIPLFWLEESTTLEDKYTDMLKNQLFKNLRIINIVKWVVIG
SGAGCVLLALFLLVYVEEP
>BmelSNMP2a
MFIEIDIHTVVREGYVFASESMNPGPACRVVRDLLIKRASIIKNIEVIRDPSDNHVIELKFSFL
QYRVREPDGIFTVKRGIHDISHLGKTVKWNGKTELPYWGRINSINNETCNTVRGTDATIYP
PHVSTDDFFEIFSTDICRMMRIDYSGKSSYKDIDGYRFTADSNVLYSATTNSENDCYCSKSV
SDATGTPSCFLDGVVDVMPCFNAPVIFSWPHFLHADDYRNGLAGMEPADPSKHETYLLIE
PNTGTPLEGKKRVQLNSVLRPVKHMTMTTNMVRTLLPMIWLDEGVSLTDDLIDELKMKY
FDKVKLARGLKYALIAISAACFTISTIFLLR
>CbowSNMP3
MKFYSVLFVVKDRANMLNKFNITVSGKIIVILGVFGLFCIFAGFYVGFKAVPDVITDKIWD
MKVLKENTEQWGMFMKTPFPFTFKVYLFDVQNPQEILQGAKPVLRETGPFVYKVYKWK
SEVEWDTPDDISYFSYMRFEFDRKASGIFSEDMKVTLFNTAYYGMLQKIDETQPEVLSTVE
GVLPSIFGENHGLFIKVKVKDYLFDGLKICENEGKDGGFVAGMVCKQMIARLPESKNLRL
EDNSILFSNMHYKNNTHQGRFTVKSGGQNRTETATLTLFNGKSYISSWTGEKSMCNKIRG
ATTVFPVNIEKNMTFEAYSEDICRTIPLEYSAEETVKDIVGYKFSAMNDSFSSTKKENFCYC
TNTTRTLDGEYGCLKDGVTDLKTCIGSSILVSFPHLLYGDEEYLDSVIGLNPEKSKHETTVI
LEPISGFPLSVTQRIQFNTFLRPIDNVISLENVSKSLFPLLWVEESLILDDQYTDMLKNELFR
TIKIVDIVKWVTIGSGAACVLIALILRMSSKTT
>CbowSNMP2
MKMFGASRFCNVKILFVTTVVATVVLIGVLLLSFVGMPLIVNDQLAKKLRLENNTEQWD
RFVELPVPLNLNVFVFNVTNSDEVTNNKATPILQEIGPYCYEERITRKILSANSTEDSITYEQ
SFNITFDEKRSGQWKESDKIVMVNPLFLILSQITNVIERFVVMGCIDKLFPPKYSTMFFEVD
IKTIMLEGIEFGVASDDIGPACNIVRNKLLEKTLPMKNVERIPSPTDPSVINSLKFAFLQYKIR
GPDGQYTTNRGIDDITQLGHIMRWDHSAEIDVWGRGESTNNATCKEVKGSDSTIYPPHVT
KSTKLDIFSTDICRTVQIRYKGTGTYQGDSGYYFGIDENTFRPATPSPENDCYCIQQTMAPD
GEPSCFLDGVVDVYPCFGAPILLSFPHFLYADESYLDGVIGIDPPNSSIHEIFLLIEPNTGTPL
QGMKRIQLNVVLRPVEFVEYTANLPSTVLPLIWIEEGVNLSQDLLDKLDKMYFNVIKAAD
AAKYAAIGVLTAFVLISGGFFVRKRYFK
>CbowSNMP1b
MRLPLKLGVAGFLLILLSVIVGFIALNPIVRFGIRQQTALKRKSEIRNIYLKLPFPLDFRVYFF
NISNPMEVQKGATPILTEIGPYCYDEFKEKIDVLDNDAEDSLTYYPYDIYKFNAEKSGKLSD
TDYVTILHPALVGMVNQATRDSPALLSIVNKAIGPIFRDPESIYLTAKVKDILFDGVELNCK
VTEFAAKAVCTQIKSQIPGIKSDPEKSIFLFSLLGVKNATVGKSIKVSRGISNSRDLGKVLEF
DGKKVLKLWYEEQCNHFKGTDGWIIPPLLKPEEGLWSFSADLCRNVVAEYVEDSVTKGV

KTRRYEATLADMQNNEEDKCYCPTPKTCLRKGVFDLSKCMGVPILATLPHFLEADEIYLQ
QVKGLNPILDKHIIRIQLEPMTGTPIEARKRLQFNLPVSASEKITLMRNVSTSLHPIFWIEEG
VELDGALLEKVTEVFTFLGVFQVFRWLGLLIGFVSIAYAVYHHMKHSRSVHITPISGSSSSD
HVDINRSTNELVGKMKEVFQSDKGHTNPVMTGHEFDRYS
>CbowSNMP1a
MRFPVKLAIGSISAFIFIILVGFVLFPRMITSKVKGMVNLAPGNEIRDMFIKVPFALSFKIYLF
NVTNPMEIQSGEKPIVKEVGPFCYEEWKEKMNIEDKEEDDTISYNQKDTYLKKWWPGCR
NGQEEVTIPHPLILGIVNTVARQKPGALSLINKAIKSIYSDPSSIFLTAKVDDILFDGVVINCN
VSDFAGKALCGQLRTAEALTKVGEVEKFSLFSSKNATLQKRIKAYRGKKNHRDVGRIVEY
NSSKMMDVWPTEECNSIEGTDGTIFPPLTKPGEGLFMFSPDLCRSLIAFFVRKSTYDGIPCG
EFTADLGDMSKNEKEKCYCSTPETCLKKGMMDLYKCSGIPIYASFPHFYNSDTSYLKGVG
GLSPNKTKHEIKILFESITGSPLYARKRLQFSMPLESTQKVELFKNFTGTVLPIFWIEEGVGL
NRTYTGQLKSLFTLTKVVKVSKWLILIGSLGGLAAAGYLFFKVDGRADITPVHEIRRHESK
SGSTVNGAGGHVLSGNGLEKY
>OcomSNMP2
MKKILKSKYCSVKVLLICLAVLLVVLVGVLILSFYGIPKIIDVIVHQNVELKDNTEQWDRFI
DLPIPITLNAYIFNVTNSDAVLRGEKPILVEVGPYVYKEQIHRNVLSTDHDSITYQQSIGITF
DQELSGNLKETDEICIINPALLVLTQITSPLEQLAITGCLDKLFSPEYNKLFVKVNIKTVMFD
GIPFAQVSPDVGYACNVIRNQVIEKTKKIRNVQRIYNKVYNDVVDGLIFALLRYKSAAPDG
VYTINRGIDDVSQLGQIMRWNYSSHLPYWGRSQSKNIDSCQLVHGGDSTIYPPHVTKDDIL
SIFATDVCRNIDIFYTGKDTYKNIDGLKFEPKSSTFRSTLSNIEDDCFCIKLTKNAQGQLSCY
LDGVLDMYECFGVPILLSFPHFLYADESYINGVEGLTAADPQKHGIYLLIEPNTGTPLEGRK
RVQFNMITRPVEYMSFTKKIYSTVMPLVWIEEAVDLTDDLVEMVNNKYFKNVKIATGVKY
GLIAVSASLVIVVTTILVRKSLF
>OcomSNMP3
MVNFFVFNVSNKFLIPLGIVGSILLFGGVFVGFKIVPDTITDKLWETKILKENTQQWDIFLE
TPFPFTFKVYVYDVKNPEEILQGGKPVVQEVGPFVYKVFKWKKEVAWTTPDDIAYNLYTR
FHFDKDASGFYSDDTEVTILNTMYYSILLKIEETQPDAFNLIEGELPTIFGENDGLFIKVKV
KDYLFDGLKFCENEGADGGFASSLLCKRIIGKMNESRNLRLENKTVVFANMYYKNNTHL
GRFTVKSGQNNREEAGMLNLYNGKDYITSWAGDKSICNKIRGVTTVFPVGVRRNMIFESF
SEDICRAVALEYSSDETVKTIKGLKFVAKNDTFSSTKKDNSCFCLNKTRTFDSSLGCAKDGI
TDLSPCTGGPVMVSFPHLLYGDKEYQTSVQGMEPNKSKHESFIVLEPISGLPLYAAQRIQF
NMFIRPMEGMESLNNVSRALMPLIWIEETVVIGDQYIEMMKSQLFHTMNLANIITWVVIIS
GSGCLLLAVLIVIHKQAP
>OcomSNMP1b
MKLSLKFMIGGVVLLLGTIIIGFVEFQTLVEFVVKDQTALRKRNEIRDIYLKIPFPLNFKIYF
FNVTNPLEVNNGATPILQQIGPYYYDEYKEKINVIDNDAQDTLQYDSFDTYIFNKTLSGKL

SDEDFITIIHPLLVGMVNTVTSTSPALLSIVNQAITSIFKQPKSIYLTDKVKNILFKGMEINCQ
GSDFASKAVCTQLKSQIPDIKESTTQKNVLLYSLLGKRNASITDTVKILRGIKNSKDLGRVL
EVNGKSEIGLWGSENCNRFKGTDGWIIPPLLKPEEGIHCYTTNLCRNIALEYVKDDNLKGI
NVRRYEGNFGDQQSVAADKCYCPSPKPCLKKGIFDLNKCLGAPIIASLPHFLYADEIFLTQV
KGLKPVKEDHILTVSIDPITSAPISVKMRVQMNLEIAPNPKITVMNNLTTALHPIFWLEDSL
DLEGPLLSKISNIFLLLKIAYVIKWIILIASCGLFAYGGYLHFKSSKTVKITAVHQRSENTLNR
STNELVAQLRNEEKHGISNNIMSGHEFDRYN
>OcomSNMP1a
MNLVQFQKFLLSQNKFNNTIMKLRLPVKLAIGSFCALFFIILVGFIIFPKLITSKVKAMVNLG
PGMDIREMFLNVPFPLSFKVYIFNVTNPLEIQNGAKPEVKEVGPFCFQEWKKKVEVADDE
KSDIISYISVDTFTRTSGRKCVSGKVVVTIPHPMILGMVNTVSRAKPGALALINKAIKSIYE
NPTSIFLTDTVDNILFDGVIIKCGVTDFAGKAICSQLRDSGTLNIIDEKNLAFSLLGPKNATE
QKRIKALRGTRNYHDVGRIVEYDGSPVMTTWPTDECNLISGTDGTVFPPMLLKEEGLVSF
APDLCRSLKAFWVQKTKYDGIPVNKYTASLGDMSKNENEKCYCYTPDTCLKKGLMDLY
KCIGVPIYVSLPHFYDSDESYINGVIGLKPNKTEHEISILFEQLTGGPVSAKKRLQFSMPLEP
NPKVDLFKNFTNTVIPLFWVEEGVDLNNTFTKPIKMLNTMKTVVKVSKWIILVASLAGLA
VSGYLFLKNDQTISITTAKVIKKAPASGISTINGHINRSTSENEVN
>PstrSNMP1
MQLSVKLILGGAAMLGATVLVGFLGFQPLVNVVVKDQTSLRKRNQMRKLYLNIPFPLDTR
IYFFNVTNPMEVQNGSKPILQEVGPYCYDEHKNKINVADNDAEDSLRYDAFDVYRFNKN
RSGNLSDEDYVTIIHPLLVGMVNRVASDSPALLSILNQAFETIFKNPQSIYLTDKVKNILFDG
MELNCEGADFAAKAVCTQLKSLVPGIKEKPTNKNVLLYSLIGPRNATVASTIKVLRGTRNY
KDIGRVLEVDGKKQITLWGSDFCNRFRGTDGWIIPPLLDPADGIQSYTPHLCRNIDLKFVK
DDVIKKIQVRRYETTLGDQTNNTLDKCYCSPKRCLKKGVFDLTKCVGAPIMATLPHFLET
DQSYLSQVDGLHPNWEDHILNINIEPMTSAPLDVKIRIQMNLEIGPQPKISVMKNLPVALHP
IFWLEDGLELEGELYEKIANIFVLLKMAQILRWTFIVVSIAIIAYGYYLIMKNRKSVKITPVH
SASSYDSYDNEAFNNRSTNAIISQLKTEMNYPKNYRNVSNNNTNNNNVGGGHEFDRYS
>PstrSNMP2
MVNKNGLKLSNKVLIFVSVLGALMVIGGAYLGFKVVPDIIVNKIWETKILKENSEQWEAF
MKTPFPYSFKVFVFDVQNPDDVLQGAKPRVKEVGPFVYKVTKWKDDVQWTSPDEISYHS
YTKFEFDEASSGEYTENTEVTILNSPLYGILLKVEATKPEVFGLVEQAVPVAFAGHSQLFIK
VKVGDLLFKGIKFCENAGKNGGFATSIFCRNVMQKANESQSLRLENDAILFSNLHYKNNT
HLGRFTVKSGGKERKESATLTLYNGKPFLSTWPGENSSCNRIRGFTTVFPANIKTDMVFES
FSEDICRHVALEYDSKDAVKEIAGYKFVAKNDTFSSKTNKENSCFCSNRTKTFTTAEGCPE
DGIIDLTPCKGGPVMVSFPHLLYADEGYARSVEGLRPVKSRHEPFVILEPLSGLPLYGSQRIQ
FNMFLRPIEGMENPWNVSRSLLPLIWVEESFVIPDQFIGKLNDNLFSKLNMINIVKWIVIVS
GGAGLLLAGLTLVYRQAP

>DvirSNMP2

MKRPCICCSVKILAVITAVLVVVLAGILLLSFYGIPRIVNSQVDASVRLTENSDQWGRFLKL
PIPIYIGVYLFTVRNGPAVMNGAVPKLEEVGPYVFREDISKKVLSTNKDDDSVTYQKFTEQ
SFEQERSGQLTLDDNVTIVNPPLLVLTQITSVVEQLVVVGCLDKIFTPTYRDLFMTTTIRSV
MFEGFEFARSSSDVGYACEVIRQQIIEKTKKMKNIRRIYSKEYPGMVEALRFSFLGFKTESP
DGIYTLNRGIDDISKLGRIMQWNNSHYLPFWGTSQSINNDTCQLVEGGDSTMYPPHATKD
KGFVIFSTDICRRVEVSYSSTGTYKDIEGYRYEPKSDTFYSKLANVEEDCFCTGRTLNPNG
VRDCYLNGVIDVYDCFGAPLLLSFPHFLHADESYINGVQGVTSPDPEKHGIYLLIEPNTGT
PLQGRKRVQLNVVLRNIEYIQFTQKLKPTVLPVIWIEECADLTDDLINILNNQFFKVVKIAN
GVKYGLIAVSSLSVLVVAGILIRKQVISRRK

>CforSNMP1

MNLNSLLGISSAPSSLMQTLRGQLKKAPSRLTYFRQLPFFPFPYAVFLRRITGTASSGKMQY
PLKIIIASAGAMFFIVLFGFVVFPKIITSKVKGMVNLKPGSEIRDMFLKVPFPLEFRVYVFSV
LNPMDVQGGAIPNLQEIGPFCYEEWKTKINVQDNEGDDTISYNAVDTFQPASWPGCLTGE
EEVTVPHPMILGMVNTVVRMKPGAISLANKAIKSIYENPPSIFITVKAKDLLFDGVVIHCG
VKDFAGKAICTTLKAEPSLRQINEDDIAFSLFGPKNGTPSKTIVALRGVQESRSVGKIVTYD
GEKKQGVWNGTKCNQILGTDGTIFPPFLKKEEGLMSFSPDLCRSLGAVYEKKAKYEGIPV
SAYYASLGDQSKNADERCFCTTPETCMKKGLMDLFKCSGVPIYASLPHFYDSHDSYLKGV
KGLQPNKEKHGIRILFESTTGSPVYAKKRLMFSMPLEPNPKVDLFTNFTETIFPLFWVEEG
VELNNTYTKPLKDLFKIKKIVKVVKYLVLLGSLAGMGAGAYLHFTQTAQTSLEPVRKVKP
APDTKSPISTVYNGNSLEAANGNLSEKY

>CforSNMP2a

MFRKFWTLKVLWIINSAFLVLLGGVLLFGFWGLPRIISNQLHQQLELEKGTEQWDRFVDL
PFALNYTIRFWTVQNPYDIINLNQTPVLEESKPYNYFLHIKKKNITTNDSDDTVTYERELSF
EFDNSTDARETDVVTILNPVILSSLQLTNTVERLAWSGCVEKLYKRTETDQIFINKTVRDLL
FDGIIFAENDESVGVACSIVRERIIKLVENIRPLEYIEENNGTRSYIRFSFFNYKTGSYQKPRT
DGIYTISRGRDDLSLLGNILLYNGDSHLPTWGNSMSTNNETCNRVRGTDSTIFSPNRKEGD
TLDNFNSDICRIVNIKFVSSEESYLGINAYRYEATNTTFRPETSNPENDCFCSKSVTNINHDE
DCYLDGLFDFKQCLGAPVLVSFPHFLYADEKYLNGVKNLLPDPEKHKIFLLIEPNTGTPLQ
GRKRIQLNSILRREPLIPQTPENMTEAVFPLLWIEEGFDLPEEYVDKLKRQYFDKVNLATGV
KFGSIAVSSALLTISTFFLARKLFIRTRPSHKTK

>CforSNMP2b

MLWAKIKPPNYKLLFHVAVMGAVLLVSGALLGFKIIPDLIEDKIWEKKVLRMNTEQWDVF
LKMPFPVTFNVYLFDVQNPDEVMSGAKPVVKEKGPYTYKLNRWKSNITWDQKSDEISYF
EYERYQFDEDATKPLFETDTVTLLNIPYNSILLTTEHLMPSAMSMLDPALPSIFGELNDLFV
RVKVKDFLWAGLRFCRDGDSENFAAKMVCKQIKNKLKSTKQMRLEGEVIVFASLYYRND
SHHGYFTVKSGQHHETKTGKLTKVNNQTQMTTWLGEASACNQIQGLTSVFPNDVQKDF

NFDVFSEDICRTISMTFKSEEVVDDVAAYKFAAMGTTFSYSKDGGGDCFCINRTMNLDGT
YGCLFDGVGDLTTCTGGPVLVSFPHLLYGDERYLRGVEGMRPEEGLHETFVLLEPITGAPL
KVMKRVQFNMFIRSIDGITALENVTHSLMPVFWIEESLTLPQSYLDAIKYSAIKSVFILKIVR
WIVVAVGMSLVFISLFMFFYFK
>TcasSNMP1
MTSTARRRNIMKKVYKIMDRVYNITNSVNGVVKVGYCYDAKKIDVNGDSTYTYTYNDK
SGRTADDYVTVHIVGIVNTVSRDSIVDRAIKSIKDNIYITTKVRDDGMTINCKVDSATAVCT
KAIGIIKNVYKSIGRNGTNRYKVRGMKKWHGRVVNHKSTVWSTKKCNRRGTDGWIIDK
VGWTYSSDCRNMHVVTSHGVAKYYADGDMSSNDKCYCKTCKGMMDTRCMGVIYATHR
VDKVRRTVRGKITDHIVRVIIGTAKRMNIVKKISMKTAHIWIAIVGKMIKVVVAKVDVVK
YCAVCAVAGSYCYKRKKKAVTVSKTAKA
>TcasSNMP2
MGCSCCTIKVLLVCVVISVALLIVSLALAFKVFPDLLESEVNKAVRLEDGTKQYDRFVELP
FPVDFKVYLFNVSNPQQVLDGTEKPKLEEIGPFVYKQYRKKTILGKNEEEDTISYTQKETF
EFDAEASKPLTEESVVTVLNPALMSIYQLAEDLHLAGAADTCIKQTFENNQGKVFIEANVR
KLLFDGFSFCKNTSPGICGLVNDLICAIAATKRNSDLVLPDYSLIFSYLNYKRKPDDGKYTV
KRGLTNIEKLGHIVAWNDSLYTKFWGEGTTCSEVKGTDSTLYPPRVTTDSAFYIYSTDICRF
VKINYKGEESYKGIDGYLFETSEDTLRSSAPEEDCYCSKLSRDMEGKKSCFLDGVIDMQT
CFGVPVLFSFPHFLWADNKYLSAVEGLNPVEEKHKTYLVVEPNTGTPLKGMKRIQLNGVI
RPIVGIKSMLQTKRALLPLLWIEEGVSLPQKYVDELKSSYFDKVQIVDGVRYALIVISAILV
GAFGIIILRKRSHAKHHV
