# Supplementary material for: Identification of candidate chemosensory genes in the antennal transcriptome of Monolepta signata
Source: PLoS One. 2024 Jun 7;19(6):e0301177. doi: 10.1371/journal.pone.0301177 (PMC11161048; doi:10.1371/journal.pone.0301177)
Supplement: S4 File — (PDF) [file pone.0301177.s008.pdf]

File S4. Overview of transcriptome data from the antennae of *Monolepta signata*.

Statistics of RNA-Seq of antennae in *M. signata*

Sample	Raw reads	Clean reads	Clean bases (G)	Error rate (%)	Q20 (%)	Q30 (%)	GC content (%)
FA1	21,774,061	20,648,522	6.2	0.03	97.79	93.3	34.16
FA2	20,757,300	20,132,668	6.0	0.03	97.88	93.58	36.30
FA3	22,134,770	21,179,365	6.4	0.03	98.07	93.99	35.45
MA1	21,323,904	20,130,023	6.0	0.03	97.82	93.43	35.49
MA2	20,885,385	20,454,004	6.1	0.03	97.75	93.23	33.98
MA3	21,288,624	20,829,381	6.2	0.03	97.83	93.42	34.95

Summary of the antennal transcriptoms assembly in *M. signata*

Length range /bp	Transcripts		Unigenes	
	Number	Percentage (%)	Number	Percentage (%)
300-500 bp	53,300	33.39	29,009	39.71
500-1k bp	42,661	26.72	21,009	28.76
1k-2k bp	31,535	19.75	12,325	16.87
>2k bp	32,162	20.14	10,707	14.66
Total number	159,658		73,050	
Total length (bp)	216,704,872		83,267,839	
Mean length (bp)	1357		1140	
N50 length (bp)	2339		1896	
N90 length (bp)	516		440	

Successful annotation rate of genes in *M. signata*

Database	Number of unigenes	Percentage (%)
NR	29,404	40.25
NT	10,676	14.61
KEGG	10,014	13.70
Swiss-Prot	15,487	21.20
PFAM	19,137	26.19
GO	19,135	26.19
KOG	7554	10.34
Annotated in all databases	3475	4.75
Annotated in at least one database	34,233	46.86
Total unigenes	73,050	

Note: FA1, FA2 and FA3 indicate biological replicate samples of female antennae of *M. signata*; MA1, MA2 and MA3 indicate biological replicate samples of male antennae of *M. signata*.
