# Supplementary material for: Identification of candidate chemosensory genes in the antennal transcriptome of Monolepta signata
Source: PLoS One. 2024 Jun 7;19(6):e0301177. doi: 10.1371/journal.pone.0301177 (PMC11161048; doi:10.1371/journal.pone.0301177)
Supplement: S5 File — (DOCX) [file pone.0301177.s009.docx]

# Request for Change to Authorship

Check to confirm you have read [*PLOS ONE*’s authorship policy](http://journals.plos.org/plosone/s/authorship).

The authorship criteria for *PLOS ONE*, summarized below, are based on those outlined by the International Committee of Medical Journal Editors (ICMJE):

1. Conception and design of the work, acquisition of data, or analysis and interpretation of data
2. Drafting the article or revising it critically for important intellectual content
3. Final approval of the version to be published
4. Agreement to be accountable for all aspects of the work

Authors should meet all of the criteria; the contributions of all authors will be disclosed in the final publication. Any contributions that fall short of the criteria should be named in the Acknowledgments section of the manuscript. It is your responsibility to ensure that anyone named in the Acknowledgments consents to being named.

Check to confirm that all authors (including those to be added or removed) consent to the changes detailed below.

| **Reason for change in author list**  Please briefly describe the reason for adding/removing an author. | The added authors, Yu Zhang & Ge Zhang, not only provided help during the investigation, but also provided guidance during the revision. |
| --- | --- |

# Final manuscript information

| **Manuscript number**  e.g., PONE-D-17-00000 | PONE-D-23-25636R2 |
| --- | --- |
| **Complete author list, in correct order**  Please note any equal contributors with asterisks (*) or hashes (#) | Wanjie He, Hanying Meng, Yu Zhang, Ge Zhang, Mengting Zhi, Guangwei Li* and Jing Chen* |
| [**Financial Disclosure**](http://journals.plos.org/plosone/s/disclosure-of-funding-sources) – including any additions/deletions necessary due to the change in authorship | This study was funded by the Xinjiang Uygur Autonomous Region Key Research and Development Task Special (2022B02043-2), the National Natural Science Funds of China (No. 31960541 and NO. 31460473), Xinjiang Uygur Autonomous Region Key Science and Technology Task Special (2023A02009), and Xinjiang Production and Construction Corps Guiding Science and Technology Plan Project (2022ZD010). The funders had no role in study design, data collection and analysis, decision to publish, or preparation of the manuscript. |
| [**Competing Interests**](http://journals.plos.org/plosone/s/competing-interests) – including any additions/deletions necessary due to the change in authorship | Because the added authors, Yu Zhang & Ge Zhang, not only provided help during the investigation, but also provided guidance during the revision,we need to change the current manuscript’s author order and addresses, as listed below. Wanjie He, Hanying Meng, Yu Zhang, Ge Zhang, Mengting Zhi, Guangwei Li* and Jing Chen*. After consultations, all the authors agreed with the addition of authors in this paper, and all the authors agreed with the rearrangement of the names. In the final version of the article, Guangwei Li is tagged as Co-corresponding authors. |
| [**Acknowledgments statement**](http://journals.plos.org/plosone/s/submission-guidelines#loc-acknowledgments)  Please acknowledge any removed authors if they contributed to the study in any way, as well as members of any author groups who do not meet our authorship criteria. | Thank you to Professor Jianping Zhang, Dr. Zhiping Cai, Dr. Jie Su and Dr. Jie Zhao for their guidance in experimental design and methods. Thank you to academic Jing Chen from Shihezi University for their help in the experiment. We are grateful to the reviewer and the editor for comments and suggestions. |

# Adding authors

## Individual author addition #1

| **Full name** | Yu Zhang |
| --- | --- |
| **Email address** | zbzzhyu2002@163.com |
| **Full affiliation** | Plant Protection Station of Xinjiang Uygur Autonomous Region, Urumqi, Xinjiang, China 830049 |

| This person contributed to **all** of the following:   1. Conception and design of the work, acquisition of data, or analysis and interpretation of data 2. Drafting the article or revising it critically for important intellectual content 3. Final approval of the version to be published 4. Agreement to be accountable for all aspects of the work |  |
| --- | --- |
| **Specific contributions:** | |
| Conceptualization |  |
| Data Curation |  |
| Formal Analysis |  |
| Funding Acquisition |  |
| Investigation |  |
| Methodology |  |
| Project Administration |  |
| Resources |  |
| Software |  |
| Supervision |  |
| Validation |  |
| Visualization |  |
| Writing – Original Draft Preparation |  |
| Writing – Review & Editing |  |

## Individual author addition #2 (if applicable)

| **Full name** | Ge Zhang |
| --- | --- |
| **Email address** | mary_black@126.com |
| **Full affiliation** | Xinjiang Uygur Autonomous Region Science and Technology Development Strategy Research Institute, Urumqi, Xinjiang, China 830011 |

| This person contributed to **all** of the following:   1. Conception and design of the work, acquisition of data, or analysis and interpretation of data 2. Drafting the article or revising it critically for important intellectual content 3. Final approval of the version to be published 4. Agreement to be accountable for all aspects of the work |  |
| --- | --- |
| **Specific contributions:** | |
| Conceptualization |  |
| Data Curation |  |
| Formal Analysis |  |
| Funding Acquisition |  |
| Investigation |  |
| Methodology |  |
| Project Administration |  |
| Resources |  |
| Software |  |
| Supervision |  |
| Validation |  |
| Visualization |  |
| Writing – Original Draft Preparation |  |
| Writing – Review & Editing |  |

## Author group addition (if applicable)

| **Group or consortium name** |  |
| --- | --- |
| **Author who represents group** |  |

# Removing authors

## Author removal #1

| **Full name** |  |
| --- | --- |

| This person **did not** contribute to all of the following:   1. Conception and design of the work, acquisition of data, or analysis and interpretation of data 2. Drafting the article or revising it critically for important intellectual content 3. Final approval of the version to be published 4. Agreement to be accountable for all aspects of the work |  |
| --- | --- |
| This person consents to being acknowledged in the published paper. |  |

## Author removal #2 (if applicable)

| **Full name** |  |
| --- | --- |

| This person **did not** contribute to all of the following:   1. Conception and design of the work, acquisition of data, or analysis and interpretation of data 2. Drafting the article or revising it critically for important intellectual content 3. Final approval of the version to be published 4. Agreement to be accountable for all aspects of the work |  |
| --- | --- |
| This person consents to being acknowledged in the published paper. |  |
